# Network analysis as an alternative way to interpret constitutions

**DOI:** 10.1371/journal.pone.0259461

**Published:** 2021-11-01

**Authors:** Rafael Silveira e Silva

**Affiliations:** Department of Law and Public Administration, Brazilian Institute of Education, Development and Research (IDP), Brasília, Federal District, Brazil; Sapienza University of Rome, ITALY

## Abstract

This article aims to identify networks of constitutional text structured in an imperceptible way. Constitutions has the power to reveal how its devices “dialogue” with each other, forming their own communicative communities. With the help from the network analysis methodology, an interpretative model of textual mapping is proposed based on internal references between the provisions of one Constitution. We use the Brazilian Constitution, whose text is very detailed, analytical and deals with various issues. As a result, a network was identified that included 174 connections between 95 articles of the Constitution and the existence of normative communities that share codes or communicative standards. The study demonstrates the strong possibility to found structuring axis of this communities and relates to the other issues that have reached constitutional stature, showing that the complexity of some Constitutions can be observed beyond its logical sequential structure.

## Introduction

Constitutions are located at the apex of legal systems because they prescribe principles and procedures that validate laws and the entire normative set of actions and state intermediation. The constitutional valorisation movement concerns not only this hierarchy, acquired by a long process of valuation and social organization, but also historically and socially relevant thematic positions, from issues of an essentially constitutional nature to the social reality underlying these norms.

Some constitutional texts contain complex content. Such content includes the establishment of democratic, republican and pluralist principles, the guarantee of individual and collective freedoms and rights, and the limitation of state power; this content also provides programmatic devices and guidelines to be complied by the state, especially the broad list of social rights. Without disregarding the logic and organization of constitutional texts through various titles, chapters, sections and subsections, it is not always possible to establish a clear connection between its different parts or observe the content in a systemic way. In this sense, the nature and composition of the themes and subjects treated constitutionally may reveal relationships not directly perceptible, demonstrating possibilities of understanding that extend beyond the traditional methods of interpretation.

The objective of this study is to propose an exploratory and interpretive model of constitutional texts based on internal references to its own content, that is, through the citations that various articles of the constitution make in relation to other articles. Our intention is to identify the structural characteristics of the relationship network, with the help of computational and visual exploration tools, with an interest in interpreting shared mechanisms throughout various constitutional devices. This approach will allow the visualization of constitutional text citations as a map, revealing possible intrinsic communicative patterns and demonstrating a different way of observing the constitution as a structural coupling between the legal and political systems, in accordance with the theory of Niklas Luhmann. For the proposed empirical exercise, we will use the Brazilian Constitution [1], whose text is very detailed and analytical and addresses various topics of social interest.

Our goal is to visualize the constitution as a network of relationships based on an analysis of networks applied to the social sciences. In general, this study involves an eminently methodological approach that highlights the interactive processes between objects of analysis from their relationships, a method that is considered by many to be a central concept in the analysis of structuring processes of society and provides elements for understanding social phenomena.

Thus, we start from the assumption that constitutional rule has the ability to reveal to us, in an alternative way, how its provisions “dialogue” with each other. Furthermore, such dialogues or relationships may reveal the existence of internal mechanisms of constitutional text, gathered in an imperceptible way if we only take into account the mere organization through titles, subtitles and chapters. These mechanisms may have their own codes, in light of Luhmann’s theory, giving meaning and content to social agents. Thus, not only would the relationships between these agents be drivers of the creation of constitutional principles and rights, but intrinsic mechanisms of the constitution could also induce guiding realities of the actions of the agents, especially those who participate in the political and legal systems.

## Systems and the constitution as structural coupling

The concept of systems, developed by biologist Karl Ludwig von Bertalanffy in 1950, has application in several areas of human knowledge, providing a way to understand the generic functioning of any system, such as social relations. Systems are a set of interdependent elements that interact with common goals, forming a whole in view of external demand.

Luhmann [2] argues that society is a macrosystem of great complexity that incorporates several social systems that generate conditions for themselves and for others around them. This means that the definition of society can no longer be understood from a single dominant point of view but interpreted in light of its differentiation.

The social systems of society form a type of “environment” in which each system fulfils a specific function and, simultaneously, demands a function for the other systems. For this purpose, in Luhmann’s view, a social system is operationally closed to sustain its own self-reference, modifying itself from its own internal bases, and cognitively open when it offers the required responses [3]. It is through communication, that is, a manifestation with its own and unique code, that each system responds to the demands of the environment. Such communication promotes the phenomenon described by Luhmann as a “reduction in complexity” of information, resulting from a binary reasoning that the system produces: the acceptance or rejection of an idea. The greater the number of elements that constitute the interior of the system, the greater is the number of possible relationships and, consequently, the ability to sustain self-differentiation in subsystems to produce responses demanded by the environment.

In this sense, the communication of law taken as a system for its differentiation from the environment (i.e., everything that does not constitute the “legal system”) occurs through the control of the binary preference code “legal/illegal”, “licit/illicit”, “constitutional/unconstitutional”, depending on the hierarchy of the norm. These codes allow the self-differentiation of law into subsystems. The law as a system would have as its primary function to keep expectations stable, conformed by legal norms, whose stability is maintained, as the law system understands them, through "reductions in complexity".

Starting from the fact that law is a social subsystem, its existence depends on the coherence of its norms, and its complexity over time needs to be reduced through differentiation into other subsystems (economy, religion, politics, and education, for example). Thus, the legal system differs, in the public and private spheres and in the various branches of law (administrative, criminal, civil, commercial, etc.). This process reveals the evolution and, at the same time, the aforementioned self-reproduction from an autopoietic perspective.

In turn, political power, also constituted by a social system, allows finding its references with politically established foundations based on the sovereignty of the modern state, with the insertion of two poles applied to the same strand: the way in which powers are divided and the co-participation of civil society. This implies a set of specific codes, depending on the complexity inherent to this system, such as “legitimate/illegitimate”, “democratic/autocratic”, “authority/subordination”, and “government/opposition”.

Thus, the binary codes that shape the communicative process of complexity reduction reveal several facets of the internal operation of each system. From this point of view, Teubner [4] emphasizes that this process is not only the key to self-differentiation but also to the evolution of systems. When they reach a high level of complexity, systems produce relative autonomies and differentiate themselves to reduce this complexity. All differentiation implies, in addition to a reduction in complexity, the very evolution of the system. Thus, the variation in the content of each system in the selection and stabilization of their respective culture or *modus operandi* is perceived. Interestingly, the process of reducing complexity does not mean summarizing or simplifying it but rather making it more coherent and effective for the functioning of the system.

The reference to political and legal systems is not merely random. If the operational autonomy and the differentiation of functions of each of these systems are identified, the reciprocal link and influence are also clearly identified. For example, rights can only be established and defended through an organized political power that makes decisions on behalf of the collective and linked to democratic sovereignty. On the other hand, these same decisions owe their mandatory nature to the legal format they are in, as well as the relevance of their content to the current normative order.

Nevertheless, each system has a specific way of addressing a situation and changes in their environment and link this context to internal operations. Thus, the political environment does not determine the structures or operations of the legal system, nor the reverse; however, one system can be “provoked” by the other. They are not mere informational exchanges from one system to the other but, rather, the system’s ability to react and process external stimuli in accordance with its code and its operating programmes.

In this context, it is in the reaction to this idea of absolute differentiation that Luhmann [5] exposes his idea of a constitution as “structural coupling” between the legal and political systems, an evolutionary acquisition, both for the relationship itself and for its degree of differentiation.

When referring to the constitution, the author imagines a means by which the political constitution of a state is fixed, a space where the legal and political terminologies are interpenetrated at the time when a new legal fixation of the political order has to be dealt with and the political order is considered to be legal order. In addition, the idea of the constitution, as configured by Luhmann, constitutes a reaction to the total separation between law and politics and, simultaneously, the mediating mechanism that, par excellence, ensures the development of these two systems.

The meaning of this structural coupling evidence the constitution’s function as a mechanism of permanent and concentrated interpenetration between the legal and the political, further reducing the complexity of the information and, therefore, facilitating and disciplining reciprocal influence [6]. Considering the self-referencing of systems, the constitution is received by each system as an internal mechanism of control of their self-reproduction and of selective filtering of environmental influences. However, Luhmann emphasizes that the concept of structural coupling and, therefore, the role played by a constitution cannot eliminate the identity and autonomy of systems or integrate them into a hierarchical or asymmetric order.

From these ideas, we understand that the structural coupling embodied in the “constitutional mechanism” has its own logic and is also consistent with the evolution of political and legal systems. This is why Luhmann’s claim of evolutionary acquisition not only implies the evolution of the aforementioned systems but also the evolution of their coupling. We can understand, from the statements by Luhmann and Teubner, that it is not only social reality that produces law or politics; politics and law also create or seek to induce social reality from their codes.

In this sense, it would be interesting to understand constitutive elements, mechanisms or operating systems of constitutions, considering that coupling would also require a set of internal communicative operations. Consequently, such mechanisms would also require a development process. And this is the idea that we intend to achieve through the network of constitutional citations: discover mechanisms beyond the mere formal organization of the constitution, with communicative operations or own messages that help in understanding the evolutionary acquisition of the Constitution for the fulfilment of its social functions. Notably, amendments, textual changes or interpretative changes originating from the courts confirm the meaning advocated by Luhmann of the constitution as structural coupling and evolutionary acquisition between the political and legal systems. The changes do not detract from the relative stability, demonstrating an ability to adapt and accept policies as well as the adjustment in the relationship between powers. Constitutional flexibility and adaptability are evolutionary stratification processes [7] in which the core of the constitution is preserved while adjusting to remain in tune with changes in the normative, social and political environments.

Although technically elaborate with semantic structures and their own thematic organizations, the decision-making processes of analytical constitutions in young democracies were plural and participatory, leaving traces that are imperceptible under a common or merely hermeneutic reading. Thus, the internal self-references contained in constitutional texts can draw attention to the possibility of finding an additional interpretive layer that reveals internal explanatory mechanisms of the structural coupling of the political and legal systems. In other words, we believe it is possible to detect codes shared throughout different parts of constitutional texts.

Using the suggestion of “self-references,” we seek to answer these questions through the use of the methodological resource offered by network analysis applied to the textual content of constitutions.

## Visualizing the constitution through network analysis

The area of knowledge of networks focuses on the study of complex relationships. Computational advances in the big data era combined with the growth of social networks have provided the rise of this field. Studies that utilize this methodology have analysed topics ranging from the *internet* (sites connected by links) to chemical elements of a cell (connected by reactions). Thus, there has been an exponential increase in the science of networks, considering that the emergence and evolution of different networks obey common organizational principles [8].

Network analysis, in addition to being multidisciplinary, is a tool whose advantage is the possibility of graphical and quantitative formalization of concepts abstracted from properties and processes characteristic of social reality. A network is composed of different elements, the main ones being “nodes” and “edges”.

Traditional analyses interpret networks as a structure composed of the interaction of a set of social actors, i.e., nodes, that are interconnected through relatively stable and independent relationships, the edges (links between two nodes). Regarding the object of this study, we will treat the articles of the constitution as nodes, with each maintaining one or several edges with others, representing their relationships and thematic interactions. Here, the relationships will be materialized through the existing references between devices.

Network analyses have multiplied over the last few years, including studies referring to textual citations, many of which have important methodological details. Silva and Amancio [9] and Amancio et al [10] treating texts as complex networks, in which words are nodes, which are directionally linked according to the natural reading order, and show that the hierarchical connectivity and clustering of words to resolve the problem of supervised classification in the word disambiguation task. Lehmann et al [11] focuses on the topology of the network of citations of scientific and considers every paper is a node, and an edge arises when one paper is cited by another. In the research, the authors used the Stanford Physics Information Retrieval System (SPIRES). Another research, carried out by Silva et al [12], developed a detailed framework to combine network-based methodologies and text analytics to construct taxonomies and visualizations of a science field. They built citation networks using data from the Web of Science and used a community detection algorithm for partitioning to obtain science maps. The construction of network maps and the visualization of paper communities (which are the nodes of the network) is in a procedure that assigns each paper to a non-overleaping community.

A similar application was presented by Verrier for provisions of the French Civil Code [13] and Allison, that constructed a network graph of Canadian Legislation [14].

Verrier works on the development of a legal text visualization system. His research proposal was to seek a way to "translate" such texts (an article, a law, a decree or any other normative) into nodes, considering that they are connected when one quotes the other. The focus of the work were legal texts that make up the French Civil Code.

The analysis of the data sample generated 2928 nodes and 6574 edges. Data spatialization was obtained with the OpenOrd algorithm, a Gephi plug in, generating nodes and edges in different sizes to highlight the relevance by the number of established connections, as well as different colors to differentiate the clusters or communities of nodes detected by the modularity algorithm.

A similar experiment was produced by Allison, who created a network graph of Canadian legislation and the linkages between the Acts, through a data repository available on the internet (GitHub). Each instance where a piece of legislation references another Act creates a link between those acts. Where multiple references occur, the weight of the connection is strengthened. All of this is displayed visually through the network graph, composed of 550 Acts and 3782 edges. The visualization components are identical to the network produced by Verrier: (i) the sizes of the nodes represent their importance to the network based on their connections and centrality; (ii) the width of the edges is determined by the number of links; and (iii) the colors of the nodes represent communities identified through network analysis algorithms.

In this research we intend to do something similar to Verrier and Allison, but seeking to focus our attention only on the Constitution of Brazil. Although apparently less complex, the empirical study remains interesting due to the exhaustively analytical characteristics of the Brazilian text.

The text of the Brazilian Constitution is organized based on the following principles: (i) the basic unit of the text is the article, followed by ordinal numbering up to the ninth and cardinal thereafter; (ii) articles can be broken down into paragraphs or items; paragraphs are divided into items, items are divided into topics; (iii) items are represented by Roman numerals, topics by lowercase letters. In this research, I will consider all of these units to identify how each article cites or is cited by another.

In our exercise, a link between two articles of the Brazilian Constitution exists when one refers to the other. Let us look at an example, from the first connection observed.

The first article to make this type of reference is Article 5 (fundamental rights and guarantees), when referring to Article 84 (exclusive powers of the President).

Article 5. All persons are equal before the law, without any distinction whatsoever, Brazilians and foreigners residing in the country being ensured of inviolability of the right to life, to liberty, to equality, to security and to property, on the following terms:

……………………………………………………………………………………….

XLVII—there shall be no punishment:

a) of death, save in case of declared war, under the terms of article 84,

……………………………………………………………………………………….

Note that the link does not depend on which article is the source or the target of the link. Let us analyse another situation, such as that of Article 5, which is also linked to Article 15 (loss or suspension of political rights), but the remission starts from Article 15 for Article 5, not the other way around.

Article 15. Disfranchisement of political rights is forbidden, the loss or suspension of which rights shall apply only in the event of:

……………………………………………………………………………………….

IV—refusal to comply with an obligation imposed upon everyone or to render an alternative service, according to article 5, VIII;

……………………………………………………………………………………….

Notably, one article may refer to another on more than one occasion. For example, Article 39 (public servants) refers 14 times to items of Article 7 (workers’ rights).

Furthermore, our methodological adaptation for the construction of the network addresses only the cases in which the articles make explicit reference to each other. In the database, the indication of the cited articles will be enough to build the citation network.

Thus, for example, Article 5 ends up not being among the most central of the network because it is not as cited verbatim as Article 37. This does not mean that it is “less important” because the constituent Parliamentarian may have understood that the “radiant effect” of fundamental rights would make explicit citation unnecessary.

We had to carry out a pre-processing of steps applied to build networks from constitutional texts, since there was no database available on citations between articles. As a record, data collection was carried out when the 99th Constitutional Amendment had been enacted. This means that the database does not take into account new citations, given the fact that the Brazilian Constitution has already undergone modifications by the 111th Constitutional Amendment.

As a description of the database construction procedure, we will take Article 37 as an example, as the target of citations. The Table 1 details the mapping:

**Table 1:** Article 37 citation mapping process.

Article-target of citation	Target detail	Article-source of citation	Source Detail	Number of citations
37	Art. 37, Paragraph 4	15	Art. 15, V	1
	Art. 37, XXI	22	Art. 22, XXVII	1
	Art. 37, XI	28	Art. 28, Paragraph 2	1
	Art. 37, XI	29	Art. 29, V	1
	Art. 37, X	39	Art. 39, Paragraph 4	3
	Art. 37, XI		Art. 39, Paragraph 4	
	Art. 37, XI		Art. 39, Paragraph 5	
	Art. 37, XI	40	Art. 40, Paragraph 11	1
	Art. 37, XI	49	Art. 49, VII	2
	Art. 37, XI		Art. 49, VIII	
	Art. 37, XI	93	Art. 93, V	1
	Art. 37, X	95	Art. 95, III	2
	Art. 37, XI			
	Art. 37	103-B	Art. 103-B, Paragraph 4, II	1
	Art. 37, X	128	Art. 128, Paragraph 5, I,c	2
	Art. 37, XI		Art. 128, Paragraph 5, I,c	
	Art. 37	130-A	Art. 130-A, Paragraph 2, II	1
	Art. 37, XVI, c	142	Art. 142, Paragraph 3, II	7
	Art. 37, XVI, c		Art. 142, Paragraph 3, III	
	Art. 37, XI		Art. 142, Paragraph 3, VIII	
	Art. 37, XIII			
	Art. 37, XIV			
	Art. 37, XV			
	Art. 37, XVI, c			
	Art. 37, XXII	167	Art. 167, IV	1
	Art. 37, XI	248	Art. 248	1

With the database in hand, we decided to use the With the use of Gephi software and the layout algorithm ForceAtlas 2. ForceAtlas2 is a layout algorithm developed as a Gephi plugin that simulates a physical system in order to spatialize a network. With Gephi we have modularity algorithms capable of detecting communities. According to Jacomy et al [15] nodes repulse each other like charged particles, while links attract their nodes, like springs. These forces create a movement that converges to a balanced state. It creates a configured graph to help the interpretation of the data. Such algorithms calculate the layout of a graph using only information contained within the structure of the graph itself, rather than relying on domain-specific knowledge. Your goal is to draw a graphic that is more intelligible to the user. This algorithm positions nodes to avoid crossing-free layouts as much as possible.

ForceAtlas 2 works well with two concepts that are very applicable in this research. The first is the concept of “hubs”, which are nodes that have many *out-degrees*, that is, nodes from which a large number of links go to other nodes. In our research, nodes would be the articles that cite many other articles. On the other hand, in the ForceAtlas 2 layout, there is a parameter called “dissuade-hubs”, where nodes that have many *in-degree* connections stand out in the graph. These would be the articles that are most cited by other articles. While the hubs are more strongly directed to the peripheries of the clusters, the dissuade-hubs occupy the most central regions of the clusters formed within the network.

We will take as metrics the following parameters for the analysis of the generated network and the generation of its representative graph:

1. The closer the points are, the more related they are; that is, the closer the articles tend to be, the greater the reference to each other; and
2. The point size is proportional to its centrality; that is, the points representative of articles that refer more to others or are more referenced have a larger dimension in relation to others.

In addition to these aspects, network analysis also enables the identification of different levels of relationships, characterized by the observation of specific “communities” within the main structure. Barabási defines a community as a group of nodes that is more likely to connect to each other than to connect with other nodes [16].

For Patty and Penn [17], the notion of community considers how a network indicates differences and similarities between actors. There are several different ways to divide a network into communities—i.e., *“*community detection*”*. Patty and Penn see potential for many community detection applications. Note that while centrality allows classifying a node based on how connected it is, the concept of community allows classifying which group it belongs to.

In our construction of the network of articles of the constitution, communities are useful because they confront the formal division of constitutional text. That is, although the articles are divided in the constitution into chapters and sections, communities allow an alternative way of visualizing the provisions, as they are determined by mentions between articles in the text of the constitution. In addition to visualization, once detected, each community can be evaluated in terms of its “communicative codes”. In other words, we can relate each of the communities as a constitutional subsystem in terms of Luhmann’s theory.

## Results and discussion

Based on the construction parameters we adopted, a total of 174 edges between 95 articles were observed to form a complete network of citations. It was also possible to detect another 13 articles connected, but not showing any link to the network.

Next, we will highlight the characteristics of these communities and their connections based on the concepts of network analysis.

### Brazilian constitutional citations network

**Fig 1:**
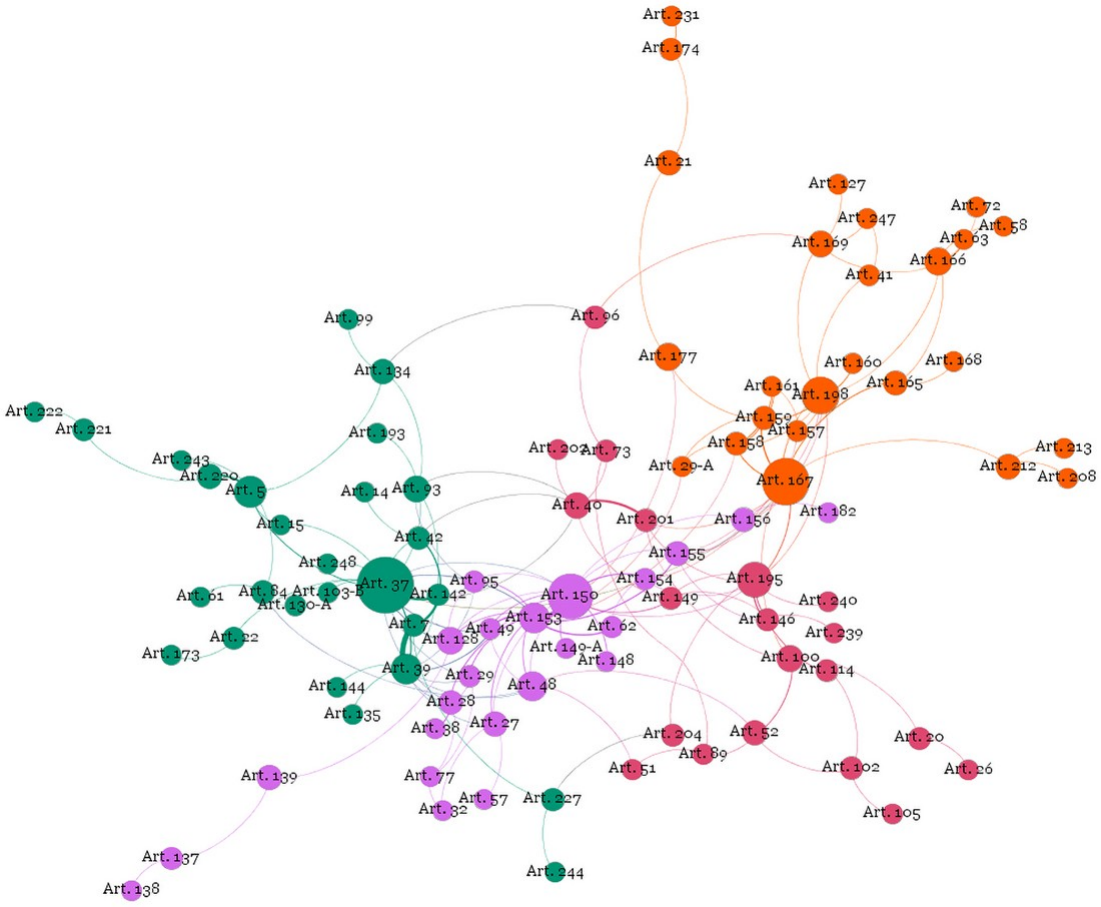
Network of cited articles: Communities of the constitution.

Fig 1 shows the network built after the described procedures. As noted above, each point is an article of the constitution, which connects with another if in some of its provisions the article refers to it or, alternatively, if it is referenced in any of the provisions of the other article.

To show how close articles tend to come closer in the graph, let us take as an example Article 150 (limitations on the power to tax) and Article 153 (Union powers to impose taxes). They are easily identifiable in Fig 1: they are two larger points close to each other in the middle of the network.

Article 150 refers eight times to Article 153, which explains their proximity in the network. However, note the wide distance between Article 222 (communication companies), in the lower left corner of the image, and Article 213 (education resources), in the right corner. Despite the topographic proximity of these articles to their numerical order in the constitution (separated by only eight articles, Article 214 to Article 221), in addition to not making reference to each other, they have no indirect connection in the network.

This explains the distance between them in the network and provides a didactic example of the intuition and logic of the algorithm. To “arrive” from Article 222, on the left, to 213, on the right, we go through the connection of Article 222 with Article 221 (principles of production and programming of radio and TV stations), Article 221 with Article 220 (communication), Article 220 with Article 5 (fundamental rights and guarantees), 5 with Article 37 (public administration), Article 37 with Article 167 (prohibitions in budgetary matters), Article 167 with Article 212 (education budget), and finally, Article 212 with Article 213.

The network, therefore, presents a configuration that makes sense because it actually brings together similar and related articles and creates distance between different and unrelated articles.

The connection between the articles of the constitution is perhaps the most interesting feature in terms of visualization. As an important airport that connects to other airports in different regions through flights, the points are more central, and more connections are generated through them. In addition, in the methodology used here, the points that connect with more diverse points, which concentrate the largest number of links and connections, are more central. Table 2 lists the 10 most central articles of the Constitution in the network.

**Table 2:** Articles with the highest number of connections in the network.

Article	Theme
37	General Provisions on Public Administration
167	Budget limitations
150	Limitations on the power to tax
198	Single Health System
195	Social Security Financing
5	Fundamental rights and guarantees
39	Public Servers
153	The Union’s powers to impose taxes
48	Powers of the National Congress
166	Budgetary legislative process

These are not necessarily issues of greater relevance relative to the normative weight but, rather, indicate the connection of themes. Keeping the analogy, for the same number of flights, an airport that connects different regions is more central than an airport that connects flights within the same region.

For example, Article 142 (armed forces) makes many references to Article 7 (workers’ rights) and Article 37 (public administration), all within the sphere of labour-related rights, such as paid vacations, whether for workers in the private sector, civil servants or the military. Conversely, Article 167 (budget prohibitions) is more central: it refers to 12 distinct articles, such as Article 37 (public administration), Article 62 (provisional measures), Article 198 (Unified Health System), Article 201 (social security), and Article 212 (education budget). Article 142 is not, therefore, a central article, while Article 167 appears to be one of the most central articles of this constitution scheme.

The algorithm that generated the network also established nine distinct communities. Some are very intuitive because, remembering, a community is a group of points that has more connections between themselves than with others. We highlight the four large communities represented by different colours in Fig 1.

The largest of the communities is composed of 27 articles and appears in a greenish tone on the left side of the network, with Article 37 (general provisions on public administration). The second largest, on the right, in an orange tone, with 25 articles, is centred by Article 167 (budgetary prohibitions). The third largest, with 23 articles, appears in the centre of the network in a purple tone and is centred on Article 150 (limitations on the power to tax). Additionally, with 20 articles, in a reddish tone, is the community around Article 195 (social security financing).

Residually, there are still communities of articles that are not connected to the described central web. These are the 13 articles that refer to or are referenced by other articles in the text of the constitution, but none of them are connected to the articles that compose the main network.

**Fig 2:**
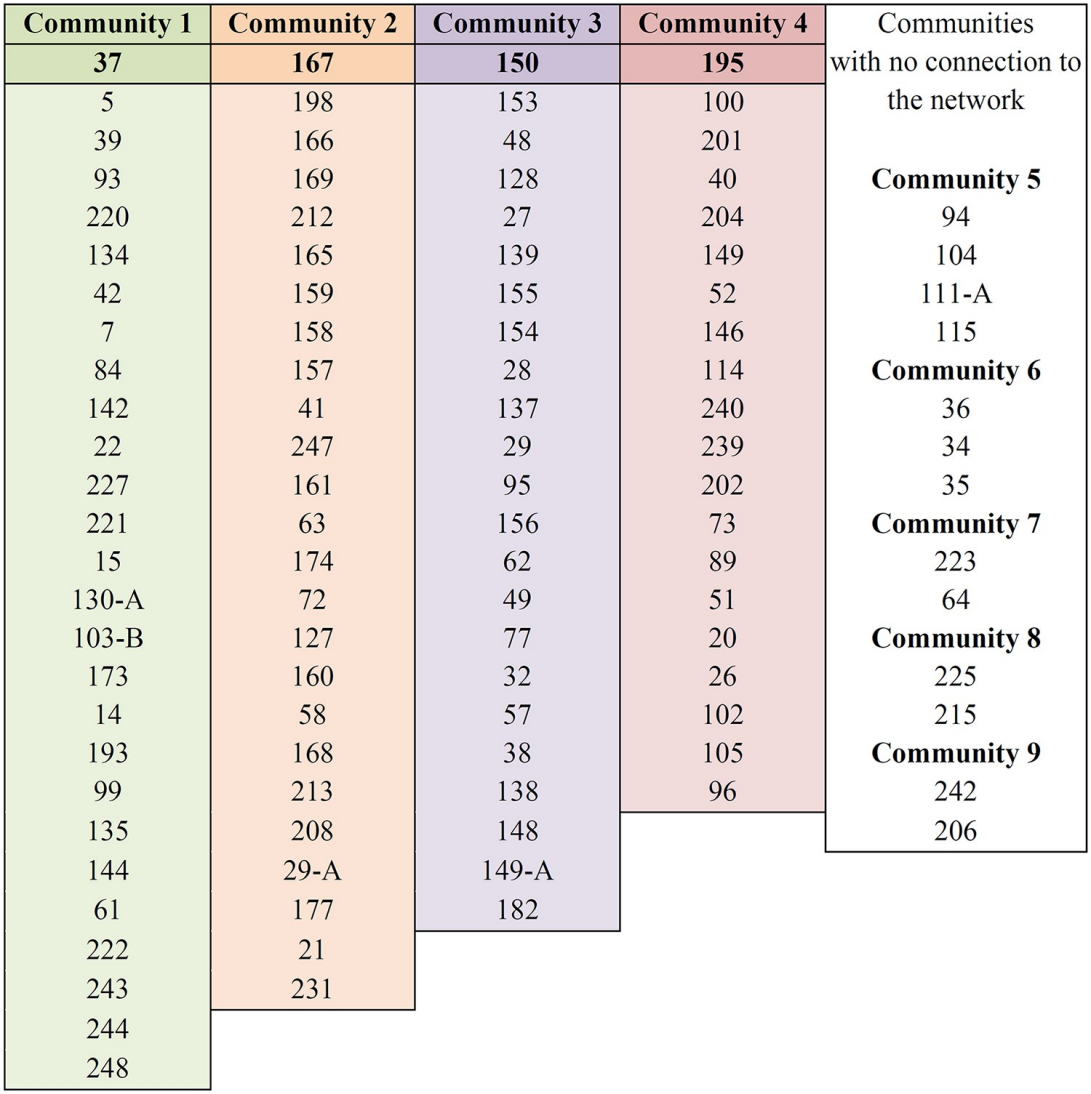
Mapped communities of articles (ranked by centrality).

We replicated the network through Fig 2, which lists the articles that make up each community, ranked by centrality.

## Discussion

Based on the visualization of the most central articles and their respective communities, we will proceed to their analysis, seeking to understand how each of them communicates and differentiates itself as an internal operating mechanism of the constitution.

Brazilian political-cultural heritage has given a central role to the state, the government and public administration, if not from the “topographic” textual point of view at least from the point of view of the number of remissions. In this sense, we emphasize that the first three central points address issues regarding the organization of the state and the powers (*lato sensu*), such as public administration (Article 37), public finances (Article 167) and taxation (Article 150).

### Public administration community

**Fig 3:**
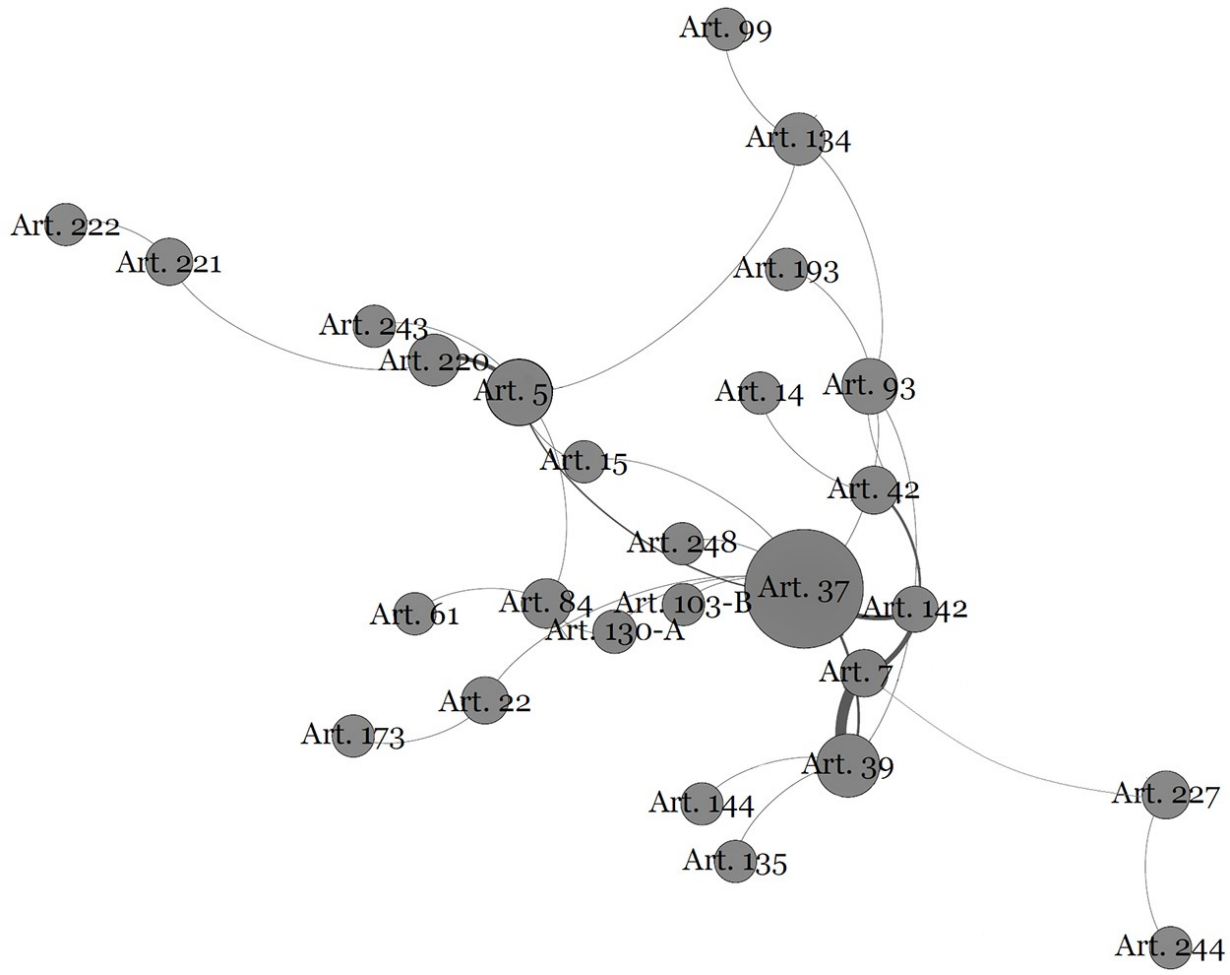
The public administration and its constitutional text connections.

The first point will be called the “Public Administration Community”. This is a normative subset that begins with Article 37. In the mapping performed, the references to this article are communicated throughout constitutional text. This community is highlighted in Fig 3 below:

There is a multiplicity of topics addressed in Article 37. It addresses the principles of public administration, rules for access to public offices, remuneration issues, bidding guidelines, and issues related to administrative misconduct, civil liability of the state and management contracts. This means that this provision is frequently cited in the most diverse parts of the constitution, making it very central to the analysis undertaken here.

The centrality of an article on public administration in the Brazilian Constitution is significant. Some arguments may explain this range of citations to so many provisions in the constitution. First, the strength of unions and other corporate entities in the preparation of the original text is recorded. The categorization of public servants is predominant in the article on public administration and is also explicit in another article that is among the ten most central, i.e., Article 39, which specifically addresses the rights of public servants.

Among the most notable textual devices, we highlight the annual review and the remuneration ceiling of public servants and public agents: Article 28 (governors), Article 29 (municipalities), Article 39 (public servants), Article 49 (subsidies for deputies, senators, the president and ministers), Article 93 (statute of the judiciary, in this case the subsidy of ministers of superior courts), Article 95 (guarantees for judges), Article 128 (public prosecutor) and 142 (armed forces). It is important to note that articles 28, 29, 49, 95 and 128, despite being connected to article 37, belong to another community (taxation limitation’s Community), which refers to the importance attributed to the limitation of the power to tax to defray expenses with actors elected politicians. These connections demonstrate the proximity of these two communities (see Fig 1). Another explanation is found in the changes to the constitution, resulting from the latest social security reforms and the interpretation capacity of the judiciary, resulting in the approximation between the rules for public servants and certain special categories, such as political agents, magistrates and members of the public ministry. This uniformity of the rules occurred by reference to Article 37. This is the case for articles dealing with the National Councils of Justice and Public Prosecutors (Articles 103-B and 130-A) as well as the application to the magistrates of the retirement rules of civil servants (Articles 93 and 40).

Finally, from the main text of Article 37 shows that the community created in its surroundings establishes its own communication, linked to the principles of “legality”, “impersonality”, “morality”, “publicity” and “efficiency”. Thus, such principles focus on the state when legislative bodies and entities are created and focus on any and all state power when exercising public administration activities.

Thus, the public administration community has its own identity and sufficient differentiation to be observed as a communicative mechanism that guides behaviours and self-referencing.

### Budgetary allocation community

**Fig 4:**
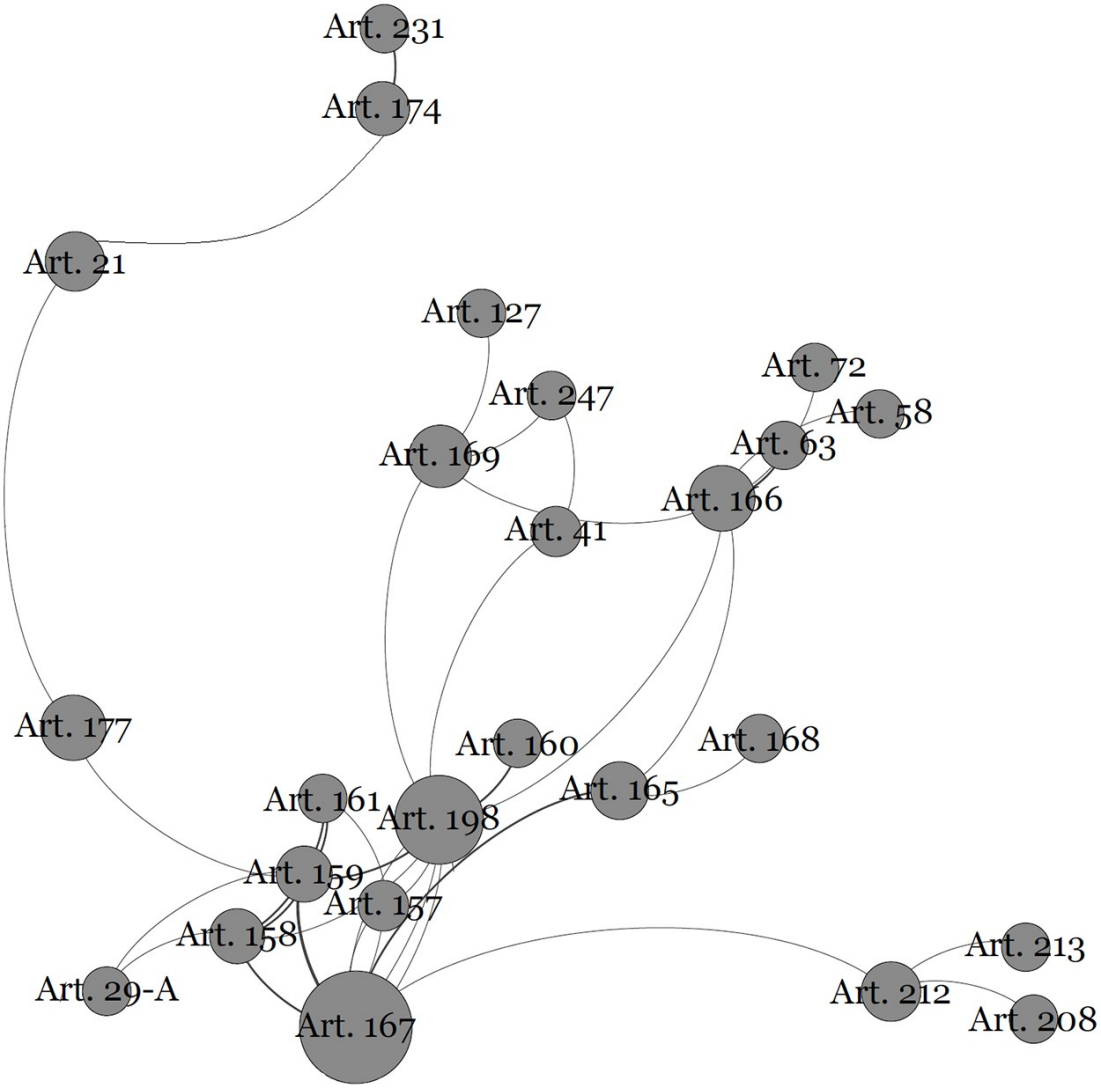
Allocation of budgetary resources and its constitutional text connections.

The second community centers around Article 167, which establishes prohibitions on budgetary matters, can be seen in detail in Fig 4:

Regarding this provision of law, several complementary explanations are designed to visualize it not only as a community of articles but also as an explanatory mechanism of Luhmannian constitutional structural coupling.

Article 167 connects directly with 12 other articles. It prohibits the binding of revenues, but it does not include health (Article 198), funds for the participation of states and municipalities (Articles 158 and 159) and revenue anticipation operations (Article 165). The scope of this community is even broader: it prohibits the use of social security contributions (Article 195) for the payment of expenses different from those of the benefits of the general social security regime (Article 201) and restricts the granting of extraordinary credit by the president in emergency situations (Article 62). Therefore, it communicates “prohibitions on the allocation and use of budgetary resources”. There are also links between different communities, in this case the close link with the Financing Social Security Community, which will be described below. It means to say that there is strong communication between these two communities, which is the need for budget allocation rules to guarantee the budget and proper financing of social security. On the network, the proximity of these two communities confirms the nature of the connection (see Fig 1).

First, there is a consequence of the centrality of norms on public finances as the positivity towards fundamental social rights grows. The demand for financial resources is quite intense for the realization of this category of rights when compared to that related to the implementation of other fundamental rights.

Thus, the Brazilian Constitution substantially expanded the predictions of rights to be ensured by the state to ensure the well-being of individuals, and thus, it is natural that the provisions on public finances are even more central. This hypothesis seems to be corroborated by the fact that the list of the ten most central articles includes Article 198 (right to health), Article 195 (social security financing), and Article 166 (budgetary legislative process). It is striking, in fact, to verify that the provision on the special budgetary legislative process can be more central than the provision that deals with the legislative process.

Another aspect that explains the centrality of Article 167 is the fact that budgetary issues are fundamental for both the organization of the state and the separation of powers.

Thus, the community that revolves around Article 167 presents a very unique communication: the need for budgetary legality that is established in the principle of the rule of law, amalgamating with the very idea of freedom by promoting the limitation of state power and, at the same time, directing administrative activities. for the realization of fundamental and social rights.

### Taxation limitations’ community

**Fig 5:**
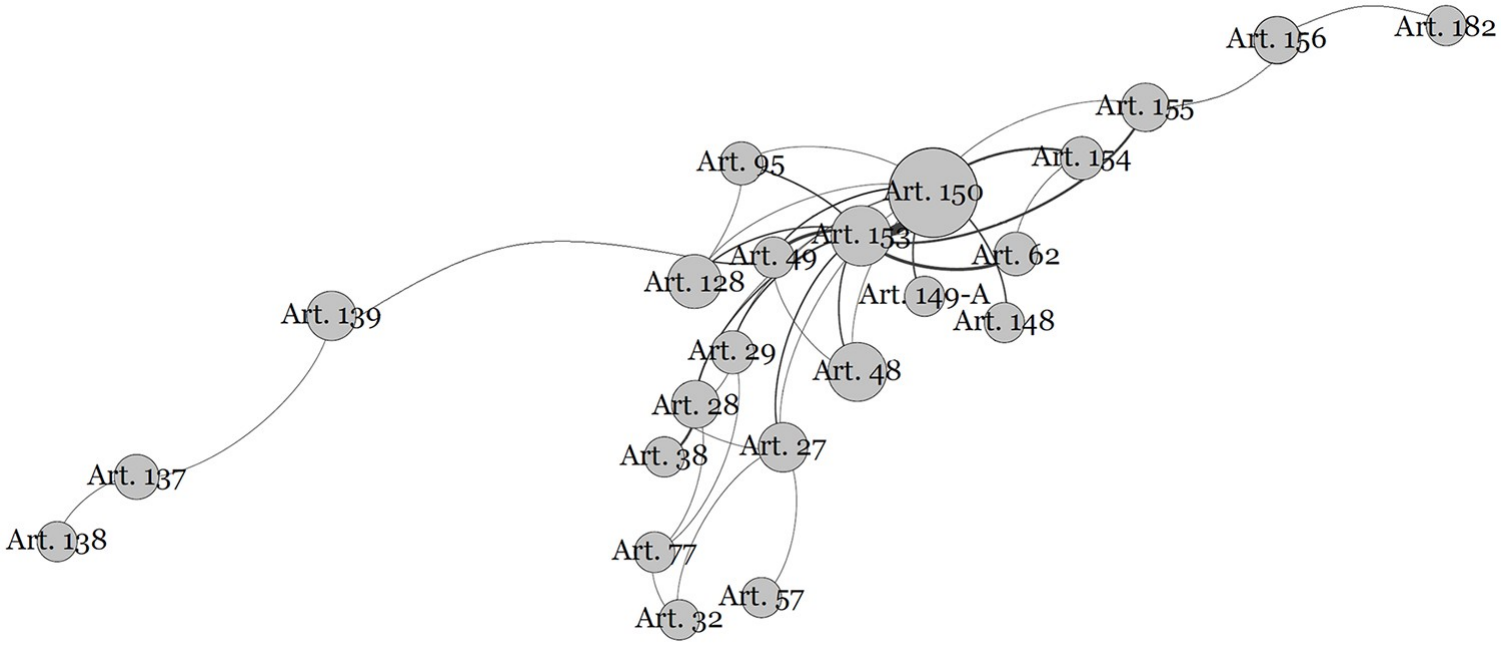
Limitations on the power to tax and its constitutional text connections. Item II of Article 150, which prohibits the differential treatment of taxpayers by profession, is referenced in other articles, especially for state deputies (Article 27); governors and secretaries (Article 28); mayors (Article 29); servants (Article 37); ministers of the federal supreme court (Article 48); deputies, senators, the president and ministers (Article 49); judges (Article 95); and prosecutors (Article 128).

The third community revolves around Article 150, on limitations to the power to tax, is the most central of the network, indicating that this theme dialogues directly with all the other mapped communities. In Fig 5 this community of articles is highlighted:

Item III of Article 150 prohibits the collection of taxes in the same year in which it was instituted or without 90 days’ notice (“noventena” principle). The principles are referenced by the provisions dealing with social contributions and intervention in the economic domain (Articles 149, 177 and 195), the municipal public lighting fee (Article 149-A), and the tax on the circulation of goods and services on fuels (Article 155).

Another important link is with federal themes. The division of powers in tax matters, for example, is within the ten most central articles, and the very provision on the limits to the power to tax plays a relevant role in this sector.

Furthermore, we believe that the most relevant factor is the strong connection between limitations on the power to tax and fundamental rights. In this context, such limitations come to be read as true institutional or personal guarantees of fundamental rights to property (principle of non-confiscation, Article 150, item IV), freedom (principle of strict legality, Article 150, item I), equality (prohibition of unequal treatment between taxpayers, Article 150, item II) and legal certainty (principles of anteriority and nonretroactivity, Article 150, item III).

There is an important link with the government’s actions (in line with Article 37), especially with regard to the recognition of legality as a relevant instrument that contributes to the security and certainty of the law. After all, legality is also an instrument of protection against attacks resulting from the exercise of power.

Not surprisingly, this subject is in a central position in the Brazilian Constitution, especially if we recognize that the National Tax System was one of the topics that gained the most attention.

### Financing social security community

The fourth community of articles deals with the financing of social security and social assistance. Observing the historical evolution of constitutional texts, the inclusion of the social security funding standard is unprecedented and has not appeared in any other constitutional or infraconstitutional provision that is known, only with regard to the cost of social security provided in the Law of Social Welfare (Law 3,807/1960) and the Consolidation of Social Welfare Laws (Decree 89.312/1984). Thus, the purpose was precisely to establish a type of safeguard for a broader concept of security of rights, incorporating health, welfare and social assistance.

**Fig 6:**
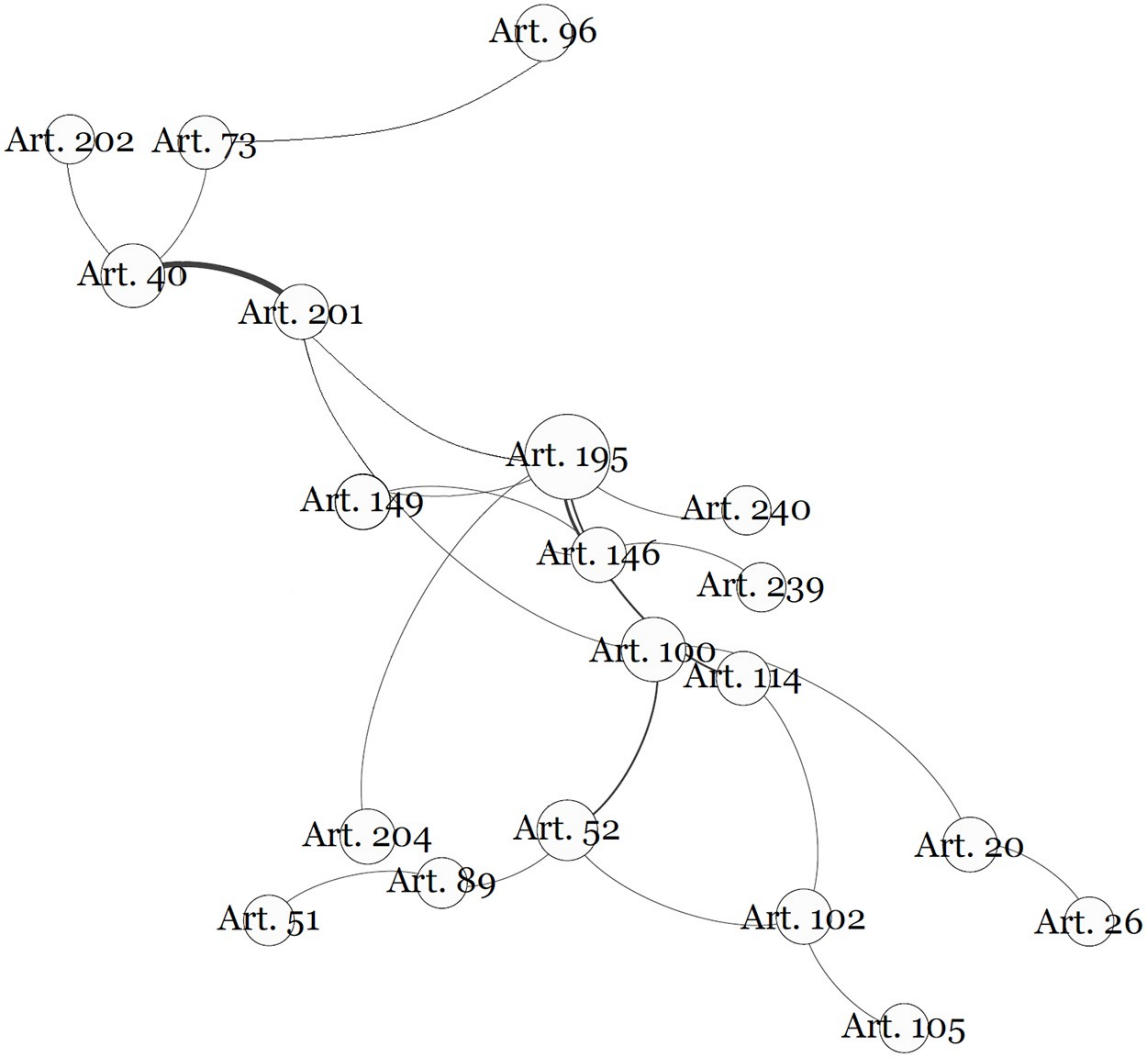
Financing social security and its constitutional text connections.

However, this mapped community still significantly reinforces an interconnection between different articles that emphasize specific communications and is especially focused on messages of safeguards in relation to the social security system. Fig 6 highlights the articles that make up this community:

We emphasize the set of specific rules pertaining to the set of rights established by social security (Article 201) and its intersection with the welfare of public servants (Article 40). Additionally, the principle of contributory solidarity is reinforced by this connection and defines in which terms the rights to benefits are structured and are ensured by their sources of funding. Notably, in this structure, the linkage of supplementary welfare (Article 202) is a relevant aspect to ensure that the funding of the social protection system can also be added to additional voluntary contributions.

Another message extracted from this community is the support offered to the social assistance system (Article 204). In addition to reinforcing the innovation of costing of this state activity, the aforementioned article is linked to Article 195 when it provides guidelines for organizations, with an emphasis on the political-administrative decentralization of social assistance and the participation of the population in the formulation of social assistance policy. An innovative mechanism of this text was introduced by Constitutional Amendment 42/2003, allowing the possibility of linking tax revenue to social assistance programmes. The message is clear: to induce governments to institutionalize their programmes by guaranteeing them a specific part of the government’s revenue.

The application of the “noventena” principle is clear; however, the exclusion of the annuity principle (Article 195, paragraph 6) for the effectiveness of social security funding, i.e., contributions that can be collected in the same year in which they were created by law, is noteworthy.

Finally, another message offered by this community is highlighted by the competence of the labour courts to judge and execute ex officio requests for the payment of social security contributions during employment contracts (Article 114, item VII). This initiative encouraged the fight against tax evasion and, consequently, an increase in the collection of social security contributions. Thus, the communication of this community addresses not only the forms of funding but also how it relates to the rights it has to guarantee and of some priorities that this form of funding has relative to other forms of revenue.

Interestingly, this community does not directly incorporate health (Article 198). Note that Article 198 is the fourth most “central” article from the point of view of the network methodology we adopted. However, this condition was not sufficient for this article to create in its surroundings a specific community of normative communication. The explanation seems to be linked to the fact that the textual conformation of the constitution regarding health is more easily circumscribed within the sequential logic established by the constituent, indicating less need for more diverse connections with other thematic parts.

## Conclusion

Among the various principles of constitutional interpretation, there is a strong trend that values criteria that favour political and legal integration when solving problems. Furthermore, this perspective does not require that constitutional provisions be treated monolithically for their proper interpretation.

In this work, strengthening the notion of the constitution as a structural coupling between the political and legal social systems, consisting of various internal mechanisms (or self-referential communities), we argue that it is possible to obtain relevant information not only from the formal organization of the Magna Carta, within a functional and thematic logic, but also from the synchronization and mapping of their internal references in the network methodology that we apply. Thus, far from establishing any hierarchy between the articles, the applied method allowed the visualization of more possibilities within a complex system such as that of the Brazilian Constitution.

One cannot fail to recognize a relationship between the articles themselves, for example, Article 37, Article 167, Article 150 and Article 195. All the communities they represent address, directly or indirectly, the concern with the sustainability of state actions, with emphasis on the balance between budget planning and the capacity to collect taxes, as well as ensuring that social security is sustainable. Therefore, of the ten articles that concentrate the most internal references, seven of them directly or indirectly address financial, tax or managerial issues. These three *hubs* are also interconnected for logical reasons and the chronology of public finances themselves: collection, expenditure and, within this, especially the administrative management of resources.

Furthermore, all of the most central articles have the characteristic of interdisciplinarity among themselves, i.e., they generally touch on themes of separation of powers, fundamental rights and federal issues, which explains the large number of connections that they establish but also the diversity of the thematic fields with which they establish relationships.

The proposed method does not eliminate other ways of viewing the constitution through other axiological axes or vectors, such as the defence of fundamental rights, or around principles, such as the dignity of the human person. These are certainly radiating elements that do not necessarily need to be verified by means of textual references between the articles of the text, as was the object of our analysis.

Importantly, the process of drafting the Brazilian Constitution, the historical moment and the intrinsic characteristics of our political and legal systems determine their own textual standards, which may coincide with constitutional articles from other countries, especially those of *civil law* tradition. Searching for elements of comparability through this method may be a direction for future studies.

The advantage of representing citation communities consists in finding structuring axis and relating to other issues that have reached constitutional status, showing that the complexity of some Constitutions can be observed beyond its logical sequential structure. When articles become self-referential, intrinsic values are self-reinforcing in an objectively imperceptible way, but which becomes evident with the mapping of communities.

The set of communities connected in the network extracted from the Brazilian constitutional text demonstrates the existence of communicative axes, in the Luhmannian sense, with a profile consistently associated with the determination of limits and processes for the action of the state and its agents, representing the relevance of liberal provisions. Such provisions of constitutional law, as observed in the analysis, are linked to the classical conception, which is concern with the defence of individual rights by limiting the power of the state. In other words, it means, ultimately, that such codes emanating from the mapped communities collaborate in the communication and differentiation between the system of law and the system of policy.

The structuring axis of these communities communicates and relates to other themes that have reached constitutional stature, such as other conceptions of the defence of the dignity of the human person and other fundamental rights of the contemporary constitutional order, showing that the complexity of Brazilian constitutional law can be observed beyond its sequential logical structure.

The network analysis carried out here can very well be applied to the constitutions of other countries and will be more appropriate the more analytical and detailed the texts. Once similar researches are undertaken, it will also be possible to carry out a comparative analysis, in order to perceive how each constitution presents intrinsic values that possible communities of citations reinforce in the constitutional texts.

## Supporting information

S1 Fig Network of cited articles in grayscale. (TIF)

S1 File Brazilian Constitution citation network database. Citations until 2017.
(XLSX)

S2 File Brazilian Constitution in Portuguese. Text used with update until 2017.
(PDF)

S3 File Brazilian Constitution in English version. Text update until 2020.
(PDF)
