# Supplementary figures and images for: Network analysis as an alternative way to interpret constitutions
Source: PLoS One. 2021 Nov 1;16(11):e0259461. doi: 10.1371/journal.pone.0259461 (PMC8559923; doi:10.1371/journal.pone.0259461)

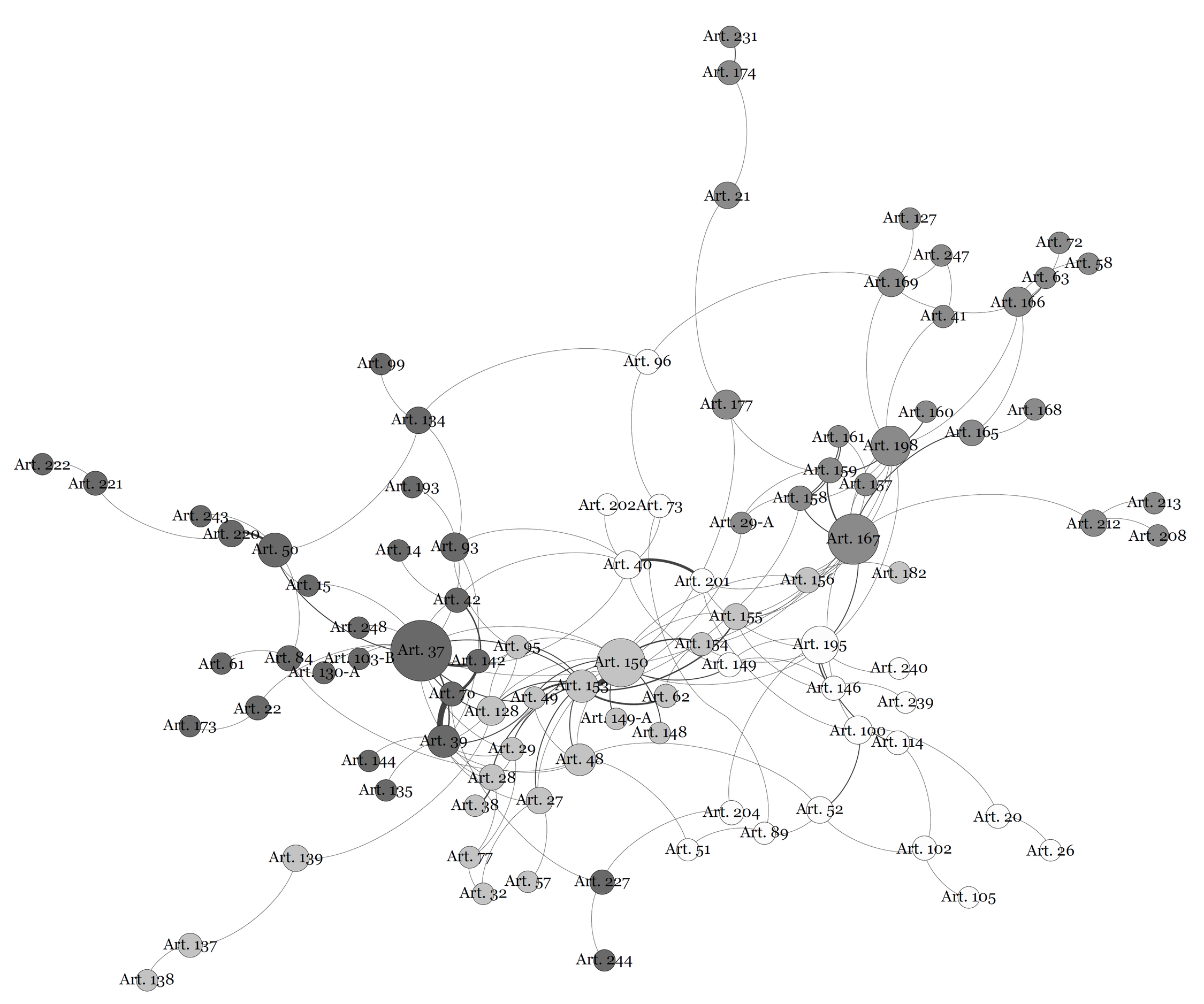

Supplement: S1 Fig — (TIF) [file pone.0259461.s001.tif]
