# Supplementary material for: Network analysis as an alternative way to interpret constitutions
Source: PLoS One. 2021 Nov 1;16(11):e0259461. doi: 10.1371/journal.pone.0259461 (PMC8559923; doi:10.1371/journal.pone.0259461)
Supplement: S2 File — Text used with update until 2017. (PDF) [file pone.0259461.s003.pdf]

CONSTITUIÇÃO DA REPÚBLICA FEDERATIVA DO BRASIL DE 1988

(Text updated until Constitutional Amendement nº 99/2017,

PREÂMBULO

Nós, representantes do povo brasileiro, reunidos em Assembléia Nacional Constituinte para instituir um Estado Democrático, destinado a assegurar o exercício dos direitos sociais e individuais, a liberdade, a segurança, o bem-estar, o desenvolvimento, a igualdade e a justiça como valores supremos de uma sociedade fraterna, pluralista e sem preconceitos, fundada na harmonia social e comprometida, na ordem interna e internacional, com a solução pacífica das controvérsias, promulgamos, sob a proteção de Deus, a seguinte CONSTITUIÇÃO DA REPÚBLICA FEDERATIVA DO BRASIL.

TÍTULO I
DOS PRINCÍPIOS FUNDAMENTAIS

Art. 1º A República Federativa do Brasil, formada pela união indissolúvel dos Estados e Municípios e do Distrito Federal, constitui-se em Estado Democrático de Direito e tem como fundamentos:

I - a soberania;

II - a cidadania;

III - a dignidade da pessoa humana;

IV - os valores sociais do trabalho e da livre iniciativa; (Vide Lei nº 13.874, de 2019)

V - o pluralismo político.

Parágrafo único. Todo o poder emana do povo, que o exerce por meio de representantes eleitos ou diretamente, nos termos desta Constituição.

Art. 2º São Poderes da União, independentes e harmônicos entre si, o Legislativo, o Executivo e o Judiciário.

Art. 3º Constituem objetivos fundamentais da República Federativa do Brasil:

I - construir uma sociedade livre, justa e solidária;

II - garantir o desenvolvimento nacional;

III - erradicar a pobreza e a marginalização e reduzir as desigualdades sociais e regionais;

IV - promover o bem de todos, sem preconceitos de origem, raça, sexo, cor, idade e quaisquer outras formas de discriminação.

Art. 4º A República Federativa do Brasil rege-se nas suas relações internacionais pelos seguintes princípios:

I - independência nacional;

II - prevalência dos direitos humanos;

III - autodeterminação dos povos;

IV - não-intervenção;

V - igualdade entre os Estados;

VI - defesa da paz;

VII - solução pacífica dos conflitos;

VIII - repúdio ao terrorismo e ao racismo;

IX - cooperação entre os povos para o progresso da humanidade;

X - concessão de asilo político.

Parágrafo único. A República Federativa do Brasil buscará a integração econômica, política, social e cultural dos povos da América Latina, visando à formação de uma comunidade latino-americana de nações.

TÍTULO II
DOS DIREITOS E GARANTIAS FUNDAMENTAIS

CAPÍTULO I
DOS DIREITOS E DEVERES INDIVIDUAIS E COLETIVOS

Art. 5º Todos são iguais perante a lei, sem distinção de qualquer natureza, garantindo-se aos brasileiros e aos estrangeiros residentes no País a inviolabilidade do direito à vida, à liberdade, à igualdade, à segurança e à propriedade, nos termos seguintes:

I - homens e mulheres são iguais em direitos e obrigações, nos termos desta Constituição;

II - ninguém será obrigado a fazer ou deixar de fazer alguma coisa senão em virtude de lei;

III - ninguém será submetido a tortura nem a tratamento desumano ou degradante;

IV - é livre a manifestação do pensamento, sendo vedado o anonimato;

V - é assegurado o direito de resposta, proporcional ao agravo, além da indenização por dano material, moral ou à imagem;

VI - é inviolável a liberdade de consciência e de crença, sendo assegurado o livre exercício dos cultos religiosos e garantida, na forma da lei, a proteção aos locais de culto e a suas liturgias;

VII - é assegurada, nos termos da lei, a prestação de assistência religiosa nas entidades civis e militares de internação coletiva;

VIII - ninguém será privado de direitos por motivo de crença religiosa ou de convicção filosófica ou política, salvo se as invocar para eximir-se de obrigação legal a todos imposta e recusar-se a cumprir prestação alternativa, fixada em lei;

IX - é livre a expressão da atividade intelectual, artística, científica e de comunicação, independentemente de censura ou licença;

X - são invioláveis a intimidade, a vida privada, a honra e a imagem das pessoas, assegurado o direito a indenização pelo dano material ou moral decorrente de sua violação;

XI - a casa é asilo inviolável do indivíduo, ninguém nela podendo penetrar sem consentimento do morador, salvo em caso de flagrante delito ou desastre, ou para prestar socorro, ou, durante o dia, por determinação judicial; (Vide Lei nº 13.105, de 2015) (Vigência)

XII - é inviolável o sigilo da correspondência e das comunicações telegráficas, de dados e das comunicações telefônicas, salvo, no último caso, por ordem judicial, nas hipóteses e na forma que a lei estabelecer para fins de investigação criminal ou instrução processual penal; (Vide Lei nº 9.296, de 1996)

XIII - é livre o exercício de qualquer trabalho, ofício ou profissão, atendidas as qualificações profissionais que a lei estabelecer;

XIV - é assegurado a todos o acesso à informação e resguardado o sigilo da fonte, quando necessário ao exercício profissional;

XV - é livre a locomoção no território nacional em tempo de paz, podendo qualquer pessoa, nos termos da lei, nele entrar, permanecer ou dele sair com seus bens;

XVI - todos podem reunir-se pacificamente, sem armas, em locais abertos ao público, independentemente de autorização, desde que não frustrem outra reunião anteriormente convocada para o mesmo local, sendo apenas exigido prévio aviso à autoridade competente;

XVII - é plena a liberdade de associação para fins lícitos, vedada a de caráter paramilitar;

XVIII - a criação de associações e, na forma da lei, a de cooperativas independem de autorização, sendo vedada a interferência estatal em seu funcionamento;

XIX - as associações só poderão ser compulsoriamente dissolvidas ou ter suas atividades suspensas por decisão judicial, exigindo-se, no primeiro caso, o trânsito em julgado;

XX - ninguém poderá ser compelido a associar-se ou a permanecer associado;

XXI - as entidades associativas, quando expressamente autorizadas, têm legitimidade para representar seus filiados judicial ou extrajudicialmente;

XXII - é garantido o direito de propriedade;

XXIII - a propriedade atenderá a sua função social;

XXIV - a lei estabelecerá o procedimento para desapropriação por necessidade ou utilidade pública, ou por interesse social, mediante justa e prévia indenização em dinheiro, ressalvados os casos previstos nesta Constituição;

XXV - no caso de iminente perigo público, a autoridade competente poderá usar de propriedade particular, assegurada ao proprietário indenização ulterior, se houver dano;

XXVI - a pequena propriedade rural, assim definida em lei, desde que trabalhada pela família, não será objeto de penhora para pagamento de débitos decorrentes de sua atividade produtiva, dispondo a lei sobre os meios de financiar o seu desenvolvimento;

XXVII - aos autores pertence o direito exclusivo de utilização, publicação ou reprodução de suas obras, transmissível aos herdeiros pelo tempo que a lei fixar;

XXVIII - são assegurados, nos termos da lei:

a) a proteção às participações individuais em obras coletivas e à reprodução da imagem e voz humanas, inclusive nas atividades desportivas;

b) o direito de fiscalização do aproveitamento econômico das obras que criarem ou de que participarem aos criadores, aos intérpretes e às respectivas representações sindicais e associativas;

XXIX - a lei assegurará aos autores de inventos industriais privilégio temporário para sua utilização, bem como proteção às criações industriais, à propriedade das marcas, aos nomes de empresas e a outros signos distintivos, tendo em vista o interesse social e o desenvolvimento tecnológico e econômico do País;

XXX - é garantido o direito de herança;

XXXI - a sucessão de bens de estrangeiros situados no País será regulada pela lei brasileira em benefício do cônjuge ou dos filhos brasileiros, sempre que não lhes seja mais favorável a lei pessoal do "de cujus";

XXXII - o Estado promoverá, na forma da lei, a defesa do consumidor;

XXXIII - todos têm direito a receber dos órgãos públicos informações de seu interesse particular, ou de interesse coletivo ou geral, que serão prestadas no prazo da lei, sob pena de responsabilidade, ressalvadas aquelas cujo sigilo seja imprescindível à segurança da sociedade e do Estado; (Regulamento) (Vide Lei nº 12.527, de 2011)

XXXIV - são a todos assegurados, independentemente do pagamento de taxas:

a) o direito de petição aos Poderes Públicos em defesa de direitos ou contra ilegalidade ou abuso de poder;

b) a obtenção de certidões em repartições públicas, para defesa de direitos e esclarecimento de situações de interesse pessoal;

XXXV - a lei não excluirá da apreciação do Poder Judiciário lesão ou ameaça a direito;

XXXVI - a lei não prejudicará o direito adquirido, o ato jurídico perfeito e a coisa julgada;

XXXVII - não haverá juízo ou tribunal de exceção;

XXXVIII - é reconhecida a instituição do júri, com a organização que lhe der a lei, assegurados:

a) a plenitude de defesa;

b) o sigilo das votações;

c) a soberania dos veredictos;

d) a competência para o julgamento dos crimes dolosos contra a vida;

XXXIX - não há crime sem lei anterior que o defina, nem pena sem prévia cominação legal;

XL - a lei penal não retroagirá, salvo para beneficiar o réu;

XLI - a lei punirá qualquer discriminação atentatória dos direitos e liberdades fundamentais;

XLII - a prática do racismo constitui crime inafiançável e imprescritível, sujeito à pena de reclusão, nos termos da lei;

XLIII - a lei considerará crimes inafiançáveis e insuscetíveis de graça ou anistia a prática da tortura , o tráfico ilícito de entorpecentes e drogas afins, o terrorismo e os definidos como crimes hediondos, por eles respondendo os mandantes, os executores e os que, podendo evitá-los, se omitirem; (Regulamento)

XLIV - constitui crime inafiançável e imprescritível a ação de grupos armados, civis ou militares, contra a ordem constitucional e o Estado Democrático;

XLV - nenhuma pena passará da pessoa do condenado, podendo a obrigação de reparar o dano e a decretação do perdimento de bens ser, nos termos da lei, estendidas aos sucessores e contra eles executadas, até o limite do valor do patrimônio transferido;

XLVI - a lei regulará a individualização da pena e adotará, entre outras, as seguintes:

a) privação ou restrição da liberdade;

b) perda de bens;

c) multa;

d) prestação social alternativa;

e) suspensão ou interdição de direitos;

XLVII - não haverá penas:

a) de morte, salvo em caso de guerra declarada, nos termos do art. 84, XIX;

b) de caráter perpétuo;

c) de trabalhos forçados;

d) de banimento;

e) cruéis;

XLVIII - a pena será cumprida em estabelecimentos distintos, de acordo com a natureza do delito, a idade e o sexo do apenado;

XLIX - é assegurado aos presos o respeito à integridade física e moral;

L - às presidiárias serão asseguradas condições para que possam permanecer com seus filhos durante o período de amamentação;

LI - nenhum brasileiro será extraditado, salvo o naturalizado, em caso de crime comum, praticado antes da naturalização, ou de comprovado envolvimento em tráfico ilícito de entorpecentes e drogas afins, na forma da lei;

LII - não será concedida extradição de estrangeiro por crime político ou de opinião;

LIII - ninguém será processado nem sentenciado senão pela autoridade competente;

LIV - ninguém será privado da liberdade ou de seus bens sem o devido processo legal;

LV - aos litigantes, em processo judicial ou administrativo, e aos acusados em geral são assegurados o contraditório e ampla defesa, com os meios e recursos a ela inerentes;

LVI - são inadmissíveis, no processo, as provas obtidas por meios ilícitos;

LVII - ninguém será considerado culpado até o trânsito em julgado de sentença penal condenatória;

LVIII - o civilmente identificado não será submetido a identificação criminal, salvo nas hipóteses previstas em lei; (Regulamento)

LIX - será admitida ação privada nos crimes de ação pública, se esta não for intentada no prazo legal;

LX - a lei só poderá restringir a publicidade dos atos processuais quando a defesa da intimidade ou o interesse social o exigirem;

LXI - ninguém será preso senão em flagrante delito ou por ordem escrita e fundamentada de autoridade judiciária competente, salvo nos casos de transgressão militar ou crime propriamente militar, definidos em lei;

LXII - a prisão de qualquer pessoa e o local onde se encontre serão comunicados imediatamente ao juiz competente e à família do preso ou à pessoa por ele indicada;

LXIII - o preso será informado de seus direitos, entre os quais o de permanecer calado, sendo-lhe assegurada a assistência da família e de advogado;

LXIV - o preso tem direito à identificação dos responsáveis por sua prisão ou por seu interrogatório policial;

LXV - a prisão ilegal será imediatamente relaxada pela autoridade judiciária;

LXVI - ninguém será levado à prisão ou nela mantido, quando a lei admitir a liberdade provisória, com ou sem fiança;

LXVII - não haverá prisão civil por dívida, salvo a do responsável pelo inadimplemento voluntário e inescusável de obrigação alimentícia e a do depositário infiel;

LXVIII - conceder-se-á *"habeas-corpus"* sempre que alguém sofrer ou se achar ameaçado de sofrer violência ou coação em sua liberdade de locomoção, por ilegalidade ou abuso de poder;

LXIX - conceder-se-á mandado de segurança para proteger direito líquido e certo, não amparado por *"habeas-corpus"* ou *"habeas-data"*, quando o responsável pela ilegalidade ou abuso de poder for autoridade pública ou agente de pessoa jurídica no exercício de atribuições do Poder Público;

LXX - o mandado de segurança coletivo pode ser impetrado por:

a) partido político com representação no Congresso Nacional;

b) organização sindical, entidade de classe ou associação legalmente constituída e em funcionamento há pelo menos um ano, em defesa dos interesses de seus membros ou associados;

LXXI - conceder-se-á mandado de injunção sempre que a falta de norma regulamentadora torne inviável o exercício dos direitos e liberdades constitucionais e das prerrogativas inerentes à nacionalidade, à soberania e à cidadania;

LXXII - conceder-se-á *"habeas-data":*

a) para assegurar o conhecimento de informações relativas à pessoa do impetrante, constantes de registros ou bancos de dados de entidades governamentais ou de caráter público;

b) para a retificação de dados, quando não se prefira fazê-lo por processo sigiloso, judicial ou administrativo;

LXXIII - qualquer cidadão é parte legítima para propor ação popular que vise a anular ato lesivo ao patrimônio público ou de entidade de que o Estado participe, à moralidade administrativa, ao meio ambiente e ao patrimônio histórico e cultural, ficando o autor, salvo comprovada má-fé, isento de custas judiciais e do ônus da sucumbência;

LXXIV - o Estado prestará assistência jurídica integral e gratuita aos que comprovarem insuficiência de recursos;

LXXV - o Estado indenizará o condenado por erro judiciário, assim como o que ficar preso além do tempo fixado na sentença;

LXXVI - são gratuitos para os reconhecidamente pobres, na forma da lei: (Vide Lei nº 7.844, de 1989)

a) o registro civil de nascimento;

b) a certidão de óbito;

LXXVII - são gratuitas as ações de *"habeas-corpus"* e *"habeas-data",* e, na forma da lei, os atos necessários ao exercício da cidadania. (Regulamento)

LXXVIII - a todos, no âmbito judicial e administrativo, são assegurados a razoável duração do processo e os meios que garantam a celeridade de sua tramitação. (Incluído pela Emenda Constitucional nº 45, de 2004) (Vide ADIN 3392)

§ 1º As normas definidoras dos direitos e garantias fundamentais têm aplicação imediata.

§ 2º Os direitos e garantias expressos nesta Constituição não excluem outros decorrentes do regime e dos princípios por ela adotados, ou dos tratados internacionais em que a República Federativa do Brasil seja parte.

§ 3º Os tratados e convenções internacionais sobre direitos humanos que forem aprovados, em cada Casa do Congresso Nacional, em dois turnos, por três quintos dos votos dos respectivos membros, serão equivalentes às emendas constitucionais. (Incluído pela Emenda Constitucional nº 45, de 2004) (Atos aprovados na forma deste parágrafo: DLG nº 186, de 2008, DEC 6.949, de 2009, DLG 261, de 2015, DEC 9.522, de 2018) (Vide ADIN 3392)

§ 4º O Brasil se submete à jurisdição de Tribunal Penal Internacional a cuja criação tenha manifestado adesão. (Incluído pela Emenda Constitucional nº 45, de 2004)

CAPÍTULO II
DOS DIREITOS SOCIAIS

Art. 6º São direitos sociais a educação, a saúde, a alimentação, o trabalho, a moradia, o transporte, o lazer, a segurança, a previdência social, a proteção à maternidade e à infância, a assistência aos desamparados, na forma desta Constituição. (Redação dada pela Emenda Constitucional nº 90, de 2015)

Art. 7º São direitos dos trabalhadores urbanos e rurais, além de outros que visem à melhoria de sua condição social:

I - relação de emprego protegida contra despedida arbitrária ou sem justa causa, nos termos de lei complementar, que preverá indenização compensatória, dentre outros direitos;

II - seguro-desemprego, em caso de desemprego involuntário;

III - fundo de garantia do tempo de serviço;

IV - salário mínimo, fixado em lei, nacionalmente unificado, capaz de atender a suas necessidades vitais básicas e às de sua família com moradia, alimentação, educação, saúde, lazer, vestuário, higiene, transporte e previdência social, com reajustes periódicos que lhe preservem o poder aquisitivo, sendo vedada sua vinculação para qualquer fim;

V - piso salarial proporcional à extensão e à complexidade do trabalho;

VI - irredutibilidade do salário, salvo o disposto em convenção ou acordo coletivo;

VII - garantia de salário, nunca inferior ao mínimo, para os que percebem remuneração variável;

VIII - décimo terceiro salário com base na remuneração integral ou no valor da aposentadoria;

IX - remuneração do trabalho noturno superior à do diurno;

X - proteção do salário na forma da lei, constituindo crime sua retenção dolosa;

XI - participação nos lucros, ou resultados, desvinculada da remuneração, e, excepcionalmente, participação na gestão da empresa, conforme definido em lei;

~~XII - salário-família para os seus dependentes;~~

XII - salário-família pago em razão do dependente do trabalhador de baixa renda nos termos da lei; (Redação dada pela Emenda Constitucional nº 20, de 1998)

XIII - duração do trabalho normal não superior a oito horas diárias e quarenta e quatro semanais, facultada a compensação de horários e a redução da jornada, mediante acordo ou convenção coletiva de trabalho; (Vide Decreto-Lei nº 5.452, de 1943)

XIV - jornada de seis horas para o trabalho realizado em turnos ininterruptos de revezamento, salvo negociação coletiva;

XV - repouso semanal remunerado, preferencialmente aos domingos;

XVI - remuneração do serviço extraordinário superior, no mínimo, em cinqüenta por cento à do normal; (Vide Del 5.452, art. 59 § 1º)

XVII - gozo de férias anuais remuneradas com, pelo menos, um terço a mais do que o salário normal;

XVIII - licença à gestante, sem prejuízo do emprego e do salário, com a duração de cento e vinte dias;

XIX - licença-paternidade, nos termos fixados em lei;

XX - proteção do mercado de trabalho da mulher, mediante incentivos específicos, nos termos da lei;

XXI - aviso prévio proporcional ao tempo de serviço, sendo no mínimo de trinta dias, nos termos da lei;

XXII - redução dos riscos inerentes ao trabalho, por meio de normas de saúde, higiene e segurança;

XXIII - adicional de remuneração para as atividades penosas, insalubres ou perigosas, na forma da lei;

XXIV - aposentadoria;

XXV - assistência gratuita aos filhos e dependentes desde o nascimento até 5 (cinco) anos de idade em creches e pré-escolas; (Redação dada pela Emenda Constitucional nº 53, de 2006)

XXVI - reconhecimento das convenções e acordos coletivos de trabalho;

XXVII - proteção em face da automação, na forma da lei;

XXVIII - seguro contra acidentes de trabalho, a cargo do empregador, sem excluir a indenização a que este está obrigado, quando incorrer em dolo ou culpa;

XXIX - ação, quanto aos créditos resultantes das relações de trabalho, com prazo prescricional de cinco anos para os trabalhadores urbanos e rurais, até o limite de dois anos após a extinção do contrato de trabalho; (Redação dada pela Emenda Constitucional nº 28, de 2000)

a) (Revogada). (Redação dada pela Emenda Constitucional nº 28, de 2000)

b) (Revogada). (Redação dada pela Emenda Constitucional nº 28, de 2000)

XXX - proibição de diferença de salários, de exercício de funções e de critério de admissão por motivo de sexo, idade, cor ou estado civil;

XXXI - proibição de qualquer discriminação no tocante a salário e critérios de admissão do trabalhador portador de deficiência;

XXXII - proibição de distinção entre trabalho manual, técnico e intelectual ou entre os profissionais respectivos;

~~XXXIII - proibição de trabalho noturno, perigoso ou insalubre aos menores de dezoito e de qualquer trabalho a menores de quatorze anos, salvo na condição de aprendiz~~ ;

XXXIII - proibição de trabalho noturno, perigoso ou insalubre a menores de dezoito e de qualquer trabalho a menores de dezesseis anos, salvo na condição de aprendiz, a partir de quatorze anos; (Redação dada pela Emenda Constitucional nº 20, de 1998)

XXXIV - igualdade de direitos entre o trabalhador com vínculo empregatício permanente e o trabalhador avulso

Parágrafo único. São assegurados à categoria dos trabalhadores domésticos os direitos previstos nos incisos IV, VI, VII, VIII, X, XIII, XV, XVI, XVII, XVIII, XIX, XXI, XXII, XXIV, XXVI, XXX, XXXI e XXXIII e, atendidas as condições estabelecidas em lei e observada a simplificação do cumprimento das obrigações tributárias, principais e acessórias, decorrentes da relação de trabalho e suas peculiaridades, os previstos nos incisos I, II, III, IX, XII, XXV e XXVIII, bem como a sua integração à previdência social. (Redação dada pela Emenda Constitucional nº 72, de 2013)

Art. 8º É livre a associação profissional ou sindical, observado o seguinte:

I - a lei não poderá exigir autorização do Estado para a fundação de sindicato, ressalvado o registro no órgão competente, vedadas ao Poder Público a interferência e a intervenção na organização sindical;

II - é vedada a criação de mais de uma organização sindical, em qualquer grau, representativa de categoria profissional ou econômica, na mesma base territorial, que será definida pelos trabalhadores ou empregadores interessados, não podendo ser inferior à área de um Município;

III - ao sindicato cabe a defesa dos direitos e interesses coletivos ou individuais da categoria, inclusive em questões judiciais ou administrativas;

IV - a assembléia geral fixará a contribuição que, em se tratando de categoria profissional, será descontada em folha, para custeio do sistema confederativo da representação sindical respectiva, independentemente da contribuição prevista em lei;

V - ninguém será obrigado a filiar-se ou a manter-se filiado a sindicato;

VI - é obrigatória a participação dos sindicatos nas negociações coletivas de trabalho;

VII - o aposentado filiado tem direito a votar e ser votado nas organizações sindicais;

VIII - é vedada a dispensa do empregado sindicalizado a partir do registro da candidatura a cargo de direção ou representação sindical e, se eleito, ainda que suplente, até um ano após o final do mandato, salvo se cometer falta grave nos termos da lei.

Parágrafo único. As disposições deste artigo aplicam-se à organização de sindicatos rurais e de colônias de pescadores, atendidas as condições que a lei estabelecer.

Art. 9º É assegurado o direito de greve, competindo aos trabalhadores decidir sobre a oportunidade de exercê-lo e sobre os interesses que devam por meio dele defender.

§ 1º A lei definirá os serviços ou atividades essenciais e disporá sobre o atendimento das necessidades inadiáveis da comunidade.

§ 2º Os abusos cometidos sujeitam os responsáveis às penas da lei.

Art. 10. É assegurada a participação dos trabalhadores e empregadores nos colegiados dos órgãos públicos em que seus interesses profissionais ou previdenciários sejam objeto de discussão e deliberação.

Art. 11. Nas empresas de mais de duzentos empregados, é assegurada a eleição de um representante destes com a finalidade exclusiva de promover-lhes o entendimento direto com os empregadores.

CAPÍTULO III
DA NACIONALIDADE

Art. 12. São brasileiros:

I - natos:

a) os nascidos na República Federativa do Brasil, ainda que de pais estrangeiros, desde que estes não estejam a serviço de seu país;

b) os nascidos no estrangeiro, de pai brasileiro ou mãe brasileira, desde que qualquer deles esteja a serviço da República Federativa do Brasil;

c) os nascidos no estrangeiro de pai brasileiro ou de mãe brasileira, desde que sejam registrados em repartição brasileira competente ou venham a residir na República Federativa do Brasil e optem, em qualquer tempo, depois de atingida a maioridade, pela nacionalidade brasileira; (Redação dada pela Emenda Constitucional nº 54, de 2007)

II - naturalizados:

a) os que, na forma da lei, adquiram a nacionalidade brasileira, exigidas aos originários de países de língua portuguesa apenas residência por um ano ininterrupto e idoneidade moral;

b) os estrangeiros de qualquer nacionalidade, residentes na República Federativa do Brasil há mais de quinze anos ininterruptos e sem condenação penal, desde que requeiram a nacionalidade brasileira. (Redação dada pela Emenda Constitucional de Revisão nº 3, de 1994)

§ 1º Aos portugueses com residência permanente no País, se houver reciprocidade em favor de brasileiros, serão atribuídos os direitos inerentes ao brasileiro, salvo os casos previstos nesta Constituição. (Redação dada pela Emenda Constitucional de Revisão nº 3, de 1994)

§ 2º A lei não poderá estabelecer distinção entre brasileiros natos e naturalizados, salvo nos casos previstos nesta Constituição.

§ 3º São privativos de brasileiro nato os cargos:

I - de Presidente e Vice-Presidente da República;

II - de Presidente da Câmara dos Deputados;

III - de Presidente do Senado Federal;

IV - de Ministro do Supremo Tribunal Federal;

V - da carreira diplomática;

VI - de oficial das Forças Armadas.

VII - de Ministro de Estado da Defesa. (Incluído pela Emenda Constitucional nº 23, de 1999)

§ 4º - Será declarada a perda da nacionalidade do brasileiro que:

I - tiver cancelada sua naturalização, por sentença judicial, em virtude de atividade nociva ao interesse nacional;

II - adquirir outra nacionalidade, salvo nos casos: (Redação dada pela Emenda Constitucional de Revisão nº 3, de 1994)

a) de reconhecimento de nacionalidade originária pela lei estrangeira; (Incluído pela Emenda Constitucional de Revisão nº 3, de 1994)

b) de imposição de naturalização, pela norma estrangeira, ao brasileiro residente em estado estrangeiro, como condição para permanência em seu território ou para o exercício de direitos civis; (Incluído pela Emenda Constitucional de Revisão nº 3, de 1994)

Art. 13. A língua portuguesa é o idioma oficial da República Federativa do Brasil.

§ 1º São símbolos da República Federativa do Brasil a bandeira, o hino, as armas e o selo nacionais.

§ 2º Os Estados, o Distrito Federal e os Municípios poderão ter símbolos próprios.

CAPÍTULO IV
DOS DIREITOS POLÍTICOS

Art. 14. A soberania popular será exercida pelo sufrágio universal e pelo voto direto e secreto, com valor igual para todos, e, nos termos da lei, mediante:

I - plebiscito;

II - referendo;

III - iniciativa popular.

§ 1º O alistamento eleitoral e o voto são:

I - obrigatórios para os maiores de dezoito anos;

II - facultativos para:

a) os analfabetos;

b) os maiores de setenta anos;

c) os maiores de dezesseis e menores de dezoito anos.

§ 2º Não podem alistar-se como eleitores os estrangeiros e, durante o período do serviço militar obrigatório, os conscritos.

§ 3º São condições de elegibilidade, na forma da lei:

I - a nacionalidade brasileira;

II - o pleno exercício dos direitos políticos;

III - o alistamento eleitoral;

IV - o domicílio eleitoral na circunscrição;

V - a filiação partidária; Regulamento

VI - a idade mínima de:

a) trinta e cinco anos para Presidente e Vice-Presidente da República e Senador;

b) trinta anos para Governador e Vice-Governador de Estado e do Distrito Federal;

c) vinte e um anos para Deputado Federal, Deputado Estadual ou Distrital, Prefeito, Vice-Prefeito e juiz de paz;

d) dezoito anos para Vereador.

§ 4º São inelegíveis os inalistáveis e os analfabetos.

§ 5º O Presidente da República, os Governadores de Estado e do Distrito Federal, os Prefeitos e quem os houver sucedido, ou substituído no curso dos mandatos poderão ser reeleitos para um único período subseqüente. (Redação dada pela Emenda Constitucional nº 16, de 1997)

§ 6º Para concorrerem a outros cargos, o Presidente da República, os Governadores de Estado e do Distrito Federal e os Prefeitos devem renunciar aos respectivos mandatos até seis meses antes do pleito.

§ 7º São inelegíveis, no território de jurisdição do titular, o cônjuge e os parentes consangüíneos ou afins, até o segundo grau ou por adoção, do Presidente da República, de Governador de Estado ou Território, do Distrito Federal, de Prefeito ou de quem os haja substituído dentro dos seis meses anteriores ao pleito, salvo se já titular de mandato eletivo e candidato à reeleição.

§ 8º O militar alistável é elegível, atendidas as seguintes condições:

I - se contar menos de dez anos de serviço, deverá afastar-se da atividade;

II - se contar mais de dez anos de serviço, será agregado pela autoridade superior e, se eleito, passará automaticamente, no ato da diplomação, para a inatividade.

§ 9º Lei complementar estabelecerá outros casos de inelegibilidade e os prazos de sua cessação, a fim de proteger a probidade administrativa, a moralidade para exercício de mandato considerada vida pregressa do candidato, e a normalidade e legitimidade das eleições contra a influência do poder econômico ou o abuso do exercício de função, cargo ou emprego na administração direta ou indireta. (Redação dada pela Emenda Constitucional de Revisão nº 4, de 1994)

§ 10 - O mandato eletivo poderá ser impugnado ante a Justiça Eleitoral no prazo de quinze dias contados da diplomação, instruída a ação com provas de abuso do poder econômico, corrupção ou fraude.

§ 11 - A ação de impugnação de mandato tramitará em segredo de justiça, respondendo o autor, na forma da lei, se temerária ou de manifesta má-fé.

Art. 15. É vedada a cassação de direitos políticos, cuja perda ou suspensão só se dará nos casos de:

I - cancelamento da naturalização por sentença transitada em julgado;

II - incapacidade civil absoluta;

III - condenação criminal transitada em julgado, enquanto durarem seus efeitos;

IV - recusa de cumprir obrigação a todos imposta ou prestação alternativa, nos termos do art. 5º, VIII;

V - improbidade administrativa, nos termos do art. 37, § 4º.

Art. 16. A lei que alterar o processo eleitoral entrará em vigor na data de sua publicação, não se aplicando à eleição que ocorra até um ano da data de sua vigência. (Redação dada pela Emenda Constitucional nº 4, de 1993)

CAPÍTULO V
DOS PARTIDOS POLÍTICOS

Art. 17. É livre a criação, fusão, incorporação e extinção de partidos políticos, resguardados a soberania nacional, o regime democrático, o pluripartidarismo, os direitos fundamentais da pessoa humana e observados os seguintes preceitos: Regulamento

I - caráter nacional;

II - proibição de recebimento de recursos financeiros de entidade ou governo estrangeiros ou de subordinação a estes;

III - prestação de contas à Justiça Eleitoral;

IV - funcionamento parlamentar de acordo com a lei.

§ 1º É assegurada aos partidos políticos autonomia para definir sua estrutura interna e estabelecer regras sobre escolha, formação e duração de seus órgãos permanentes e provisórios e sobre sua organização e funcionamento e para adotar os critérios de escolha e o regime de suas coligações nas eleições majoritárias, vedada a sua celebração nas eleições proporcionais, sem obrigatoriedade de vinculação entre as candidaturas em âmbito nacional, estadual, distrital ou municipal, devendo seus estatutos estabelecer normas de disciplina e fidelidade partidária. (Redação dada pela Emenda Constitucional nº 97, de 2017)

§ 2º Os partidos políticos, após adquirirem personalidade jurídica, na forma da lei civil, registrarão seus estatutos no Tribunal Superior Eleitoral.

§ 3º Somente terão direito a recursos do fundo partidário e acesso gratuito ao rádio e à televisão, na forma da lei, os partidos políticos que alternativamente: (Redação dada pela Emenda Constitucional nº 97, de 2017)

I - obtiverem, nas eleições para a Câmara dos Deputados, no mínimo, 3% (três por cento) dos votos válidos, distribuídos em pelo menos um terço das unidades da Federação, com um mínimo de 2% (dois por cento) dos votos válidos em cada uma delas; ou (Incluído pela Emenda Constitucional nº 97, de 2017)

II - tiverem elegido pelo menos quinze Deputados Federais distribuídos em pelo menos um terço das unidades da Federação. (Incluído pela Emenda Constitucional nº 97, de 2017)

§ 4º É vedada a utilização pelos partidos políticos de organização paramilitar.

§ 5º Ao eleito por partido que não preencher os requisitos previstos no § 3º deste artigo é assegurado o mandato e facultada a filiação, sem perda do mandato, a outro partido que os tenha atingido, não sendo essa filiação considerada para fins de distribuição dos recursos do fundo partidário e de acesso gratuito ao tempo de rádio e de televisão. (Incluído pela Emenda Constitucional nº 97, de 2017)

TÍTULO III
DA ORGANIZAÇÃO DO ESTADO
CAPÍTULO I
DA ORGANIZAÇÃO POLÍTICO-ADMINISTRATIVA

Art. 18. A organização político-administrativa da República Federativa do Brasil compreende a União, os Estados, o Distrito Federal e os Municípios, todos autônomos, nos termos desta Constituição.

§ 1º Brasília é a Capital Federal.

§ 2º Os Territórios Federais integram a União, e sua criação, transformação em Estado ou reintegração ao Estado de origem serão reguladas em lei complementar.

§ 3º Os Estados podem incorporar-se entre si, subdividir-se ou desmembrar-se para se anexarem a outros, ou formarem novos Estados ou Territórios Federais, mediante aprovação da população diretamente interessada, através de plebiscito, e do Congresso Nacional, por lei complementar.

§ 4º A criação, a incorporação, a fusão e o desmembramento de Municípios, far-se-ão por lei estadual, dentro do período determinado por Lei Complementar Federal, e dependerão de consulta prévia, mediante plebiscito, às populações dos Municípios envolvidos, após divulgação dos Estudos de Viabilidade Municipal, apresentados e publicados na forma da lei. (Redação dada pela Emenda Constitucional nº 15, de 1996) Vide art. 96 - ADCT

Art. 19. É vedado à União, aos Estados, ao Distrito Federal e aos Municípios:

I - estabelecer cultos religiosos ou igrejas, subvencioná-los, embaraçar-lhes o funcionamento ou manter com eles ou seus representantes relações de dependência ou aliança, ressalvada, na forma da lei, a colaboração de interesse público;

II - recusar fé aos documentos públicos;

III - criar distinções entre brasileiros ou preferências entre si.

CAPÍTULO II
DA UNIÃO

Art. 20. São bens da União:

I - os que atualmente lhe pertencem e os que lhe vierem a ser atribuídos;

II - as terras devolutas indispensáveis à defesa das fronteiras, das fortificações e construções militares, das vias federais de comunicação e à preservação ambiental, definidas em lei;

III - os lagos, rios e quaisquer correntes de água em terrenos de seu domínio, ou que banhem mais de um Estado, sirvam de limites com outros países, ou se estendam a território estrangeiro ou dele provenham, bem como os terrenos marginais e as praias fluviais;

IV as ilhas fluviais e lacustres nas zonas limítrofes com outros países; as praias marítimas; as ilhas oceânicas e as costeiras, excluídas, destas, as que contenham a sede de Municípios, exceto aquelas áreas afetadas ao serviço público e a unidade ambiental federal, e as referidas no art. 26, II; (Redação dada pela Emenda Constitucional nº 46, de 2005)

V - os recursos naturais da plataforma continental e da zona econômica exclusiva;

VI - o mar territorial;

VII - os terrenos de marinha e seus acrescidos;

VIII - os potenciais de energia hidráulica;

IX - os recursos minerais, inclusive os do subsolo;

X - as cavidades naturais subterrâneas e os sítios arqueológicos e pré-históricos;

XI - as terras tradicionalmente ocupadas pelos índios.

§ 2º A faixa de até cento e cinqüenta quilômetros de largura, ao longo das fronteiras terrestres, designada como faixa de fronteira, é considerada fundamental para defesa do território nacional, e sua ocupação e utilização serão reguladas em lei.

Art. 21. Compete à União:

I - manter relações com Estados estrangeiros e participar de organizações internacionais;

II - declarar a guerra e celebrar a paz;

III - assegurar a defesa nacional;

IV - permitir, nos casos previstos em lei complementar, que forças estrangeiras transitem pelo território nacional ou nele permaneçam temporariamente;

V - decretar o estado de sítio, o estado de defesa e a intervenção federal;

VI - autorizar e fiscalizar a produção e o comércio de material bélico;

VII - emitir moeda;

VIII - administrar as reservas cambiais do País e fiscalizar as operações de natureza financeira, especialmente as de crédito, câmbio e capitalização, bem como as de seguros e de previdência privada;

IX - elaborar e executar planos nacionais e regionais de ordenação do território e de desenvolvimento econômico e social;

X - manter o serviço postal e o correio aéreo nacional;

XI - explorar, diretamente ou mediante autorização, concessão ou permissão, os serviços de telecomunicações, nos termos da lei, que disporá sobre a organização dos serviços, a criação de um órgão regulador e outros aspectos institucionais; (Redação dada pela Emenda Constitucional nº 8, de 15/08/95:)

XII - explorar, diretamente ou mediante autorização, concessão ou permissão:

a) os serviços de radiodifusão sonora, e de sons e imagens; (Redação dada pela Emenda Constitucional nº 8, de 15/08/95:)

b) os serviços e instalações de energia elétrica e o aproveitamento energético dos cursos de água, em articulação com os Estados onde se situam os potenciais hidroenergéticos;

c) a navegação aérea, aeroespacial e a infra-estrutura aeroportuária;

d) os serviços de transporte ferroviário e aquaviário entre portos brasileiros e fronteiras nacionais, ou que transponham os limites de Estado ou Território;

e) os serviços de transporte rodoviário interestadual e internacional de passageiros;

f) os portos marítimos, fluviais e lacustres;

XIII - organizar e manter o Poder Judiciário, o Ministério Público do Distrito Federal e dos Territórios e a Defensoria Pública dos Territórios; (Redação dada pela Emenda Constitucional nº 69, de 2012) (Produção de efeito)

XIV - organizar e manter a polícia civil, a polícia militar e o corpo de bombeiros militar do Distrito Federal, bem como prestar assistência financeira ao Distrito Federal para a execução de serviços públicos, por meio de fundo próprio; (Redação dada pela Emenda Constitucional nº 19, de 1998)

XV - organizar e manter os serviços oficiais de estatística, geografia, geologia e cartografia de âmbito nacional;

XVI - exercer a classificação, para efeito indicativo, de diversões públicas e de programas de rádio e televisão;

XVII - conceder anistia;

XVIII - planejar e promover a defesa permanente contra as calamidades públicas, especialmente as secas e as inundações;

XIX - instituir sistema nacional de gerenciamento de recursos hídricos e definir critérios de outorga de direitos de seu uso; (Regulamento)

XX - instituir diretrizes para o desenvolvimento urbano, inclusive habitação, saneamento básico e transportes urbanos;

XXI - estabelecer princípios e diretrizes para o sistema nacional de viação;

XXII - executar os serviços de polícia marítima, aeroportuária e de fronteiras; (Redação dada pela Emenda Constitucional nº 19, de 1998)

XXIII - explorar os serviços e instalações nucleares de qualquer natureza e exercer monopólio estatal sobre a pesquisa, a lavra, o enriquecimento e reprocessamento, a industrialização e o comércio de minérios nucleares e seus derivados, atendidos os seguintes princípios e condições:

a) toda atividade nuclear em território nacional somente será admitida para fins pacíficos e mediante aprovação do Congresso Nacional;

b) sob regime de permissão, são autorizadas a comercialização e a utilização de radioisótopos para a pesquisa e usos médicos, agrícolas e industriais; (Redação dada pela Emenda Constitucional nº 49, de 2006)

c) sob regime de permissão, são autorizadas a produção, comercialização e utilização de radioisótopos de meia-vida igual ou inferior a duas horas; (Redação dada pela Emenda Constitucional nº 49, de 2006)

d) a responsabilidade civil por danos nucleares independe da existência de culpa; (Incluída pela Emenda Constitucional nº 49, de 2006)

XXIV - organizar, manter e executar a inspeção do trabalho;

XXV - estabelecer as áreas e as condições para o exercício da atividade de garimpagem, em forma associativa.

Art. 22. Compete privativamente à União legislar sobre:

I - direito civil, comercial, penal, processual, eleitoral, agrário, marítimo, aeronáutico, espacial e do trabalho;

II - desapropriação;

III - requisições civis e militares, em caso de iminente perigo e em tempo de guerra;

IV - águas, energia, informática, telecomunicações e radiodifusão;

V - serviço postal;

VI - sistema monetário e de medidas, títulos e garantias dos metais;

VII - política de crédito, câmbio, seguros e transferência de valores;

VIII - comércio exterior e interestadual;

IX - diretrizes da política nacional de transportes;

X - regime dos portos, navegação lacustre, fluvial, marítima, aérea e aeroespacial;

XI - trânsito e transporte;

XII - jazidas, minas, outros recursos minerais e metalurgia;

XIII - nacionalidade, cidadania e naturalização;

XIV - populações indígenas;

XV - emigração e imigração, entrada, extradição e expulsão de estrangeiros;

XVI - organização do sistema nacional de emprego e condições para o exercício de profissões;

XVII - organização judiciária, do Ministério Público do Distrito Federal e dos Territórios e da Defensoria Pública dos Territórios, bem como organização administrativa destes; (Redação dada pela Emenda Constitucional nº 69, de 2012) (Produção de efeito)

XVIII - sistema estatístico, sistema cartográfico e de geologia nacionais;

XIX - sistemas de poupança, captação e garantia da poupança popular;

XX - sistemas de consórcios e sorteios;

XXI - normas gerais de organização, efetivos, material bélico, garantias, convocação e mobilização das polícias militares e corpos de bombeiros militares;

XXII - competência da polícia federal e das polícias rodoviária e ferroviária federais;

XXIII - seguridade social;

XXIV - diretrizes e bases da educação nacional;

XXV - registros públicos;

XXVI - atividades nucleares de qualquer natureza;

XXVII - normas gerais de licitação e contratação, em todas as modalidades, para as administrações públicas diretas, autárquicas e fundacionais da União, Estados, Distrito Federal e Municípios, obedecido o disposto no art. 37, XXI, e para as empresas públicas e sociedades de economia mista, nos termos do art. 173, § 1°, III; (Redação dada pela Emenda Constitucional nº 19, de 1998)

XXVIII - defesa territorial, defesa aeroespacial, defesa marítima, defesa civil e mobilização nacional;

XXIX - propaganda comercial.

Parágrafo único. Lei complementar poderá autorizar os Estados a legislar sobre questões específicas das matérias relacionadas neste artigo.

Art. 23. É competência comum da União, dos Estados, do Distrito Federal e dos Municípios:

I - zelar pela guarda da Constituição, das leis e das instituições democráticas e conservar o patrimônio público;

II - cuidar da saúde e assistência pública, da proteção e garantia das pessoas portadoras de deficiência; (Vide ADPF 672)

III - proteger os documentos, as obras e outros bens de valor histórico, artístico e cultural, os monumentos, as paisagens naturais notáveis e os sítios arqueológicos;

IV - impedir a evasão, a destruição e a descaracterização de obras de arte e de outros bens de valor histórico, artístico ou cultural;

~~V - proporcionar os meios de acesso à cultura, à educação e à ciência;~~

V - proporcionar os meios de acesso à cultura, à educação, à ciência, à tecnologia, à pesquisa e à inovação; (Redação dada pela Emenda Constitucional nº 85, de 2015)

VI - proteger o meio ambiente e combater a poluição em qualquer de suas formas;

VII - preservar as florestas, a fauna e a flora;

VIII - fomentar a produção agropecuária e organizar o abastecimento alimentar;

IX - promover programas de construção de moradias e a melhoria das condições habitacionais e de saneamento básico; (Vide ADPF 672)

X - combater as causas da pobreza e os fatores de marginalização, promovendo a integração social dos setores desfavorecidos;

XI - registrar, acompanhar e fiscalizar as concessões de direitos de pesquisa e exploração de recursos hídricos e minerais em seus territórios;

XII - estabelecer e implantar política de educação para a segurança do trânsito.

Parágrafo único. Leis complementares fixarão normas para a cooperação entre a União e os Estados, o Distrito Federal e os Municípios, tendo em vista o equilíbrio do desenvolvimento e do bem-estar em âmbito nacional. (Redação dada pela Emenda Constitucional nº 53, de 2006)

Art. 24. Compete à União, aos Estados e ao Distrito Federal legislar concorrentemente sobre:

I - direito tributário, financeiro, penitenciário, econômico e urbanístico; (Vide Lei nº 13.874, de 2019)

II - orçamento;

III - juntas comerciais;

IV - custas dos serviços forenses;

V - produção e consumo;

VI - florestas, caça, pesca, fauna, conservação da natureza, defesa do solo e dos recursos naturais, proteção do meio ambiente e controle da poluição;

VII - proteção ao patrimônio histórico, cultural, artístico, turístico e paisagístico;

VIII - responsabilidade por dano ao meio ambiente, ao consumidor, a bens e direitos de valor artístico, estético, histórico, turístico e paisagístico;

~~IX - educação, cultura, ensino e desporto;~~

IX - educação, cultura, ensino, desporto, ciência, tecnologia, pesquisa, desenvolvimento e inovação; (Redação dada pela Emenda Constitucional nº 85, de 2015)

X - criação, funcionamento e processo do juizado de pequenas causas;

XI - procedimentos em matéria processual;

XII - previdência social, proteção e defesa da saúde; (Vide ADPF 672)

XIII - assistência jurídica e Defensoria pública;

XIV - proteção e integração social das pessoas portadoras de deficiência;

XV - proteção à infância e à juventude;

XVI - organização, garantias, direitos e deveres das polícias civis.

§ 1º No âmbito da legislação concorrente, a competência da União limitar-se-á a estabelecer normas gerais. (Vide Lei nº 13.874, de 2019)

§ 2º A competência da União para legislar sobre normas gerais não exclui a competência suplementar dos Estados. (Vide Lei nº 13.874, de 2019)

§ 3º Inexistindo lei federal sobre normas gerais, os Estados exercerão a competência legislativa plena, para atender a suas peculiaridades. (Vide Lei nº 13.874, de 2019)

§ 4º A superveniência de lei federal sobre normas gerais suspende a eficácia da lei estadual, no que lhe for contrário. (Vide Lei nº 13.874, de 2019)

CAPÍTULO III
DOS ESTADOS FEDERADOS

Art. 25. Os Estados organizam-se e regem-se pelas Constituições e leis que adotarem, observados os princípios desta Constituição.

§ 1º São reservadas aos Estados as competências que não lhes sejam vedadas por esta Constituição.

~~§ 2º Cabe aos Estados explorar diretamente, ou mediante concessão, a empresa estatal, com exclusividade de distribuição, os serviços locais de gás canalizado.~~

§ 2º Cabe aos Estados explorar diretamente, ou mediante concessão, os serviços locais de gás canalizado, na forma da lei, vedada a edição de medida provisória para a sua regulamentação. (Redação dada pela Emenda Constitucional nº 5, de 1995)

§ 3º Os Estados poderão, mediante lei complementar, instituir regiões metropolitanas, aglomerações urbanas e microrregiões, constituídas por agrupamentos de municípios limítrofes, para integrar a organização, o planejamento e a execução de funções públicas de interesse comum.

Art. 26. Incluem-se entre os bens dos Estados:

I - as águas superficiais ou subterrâneas, fluentes, emergentes e em depósito, ressalvadas, neste caso, na forma da lei, as decorrentes de obras da União;

II - as áreas, nas ilhas oceânicas e costeiras, que estiverem no seu domínio, excluídas aquelas sob domínio da União, Municípios ou terceiros;

III - as ilhas fluviais e lacustres não pertencentes à União;

IV - as terras devolutas não compreendidas entre as da União.

Art. 27. O número de Deputados à Assembléia Legislativa corresponderá ao triplo da representação do Estado na Câmara dos Deputados e, atingido o número de trinta e seis, será acrescido de tantos quantos forem os Deputados Federais acima de doze.

§ 1º Será de quatro anos o mandato dos Deputados Estaduais, aplicando- sê-lhes as regras desta Constituição sobre sistema eleitoral, inviolabilidade, imunidades, remuneração, perda de mandato, licença, impedimentos e incorporação às Forças Armadas.

§ 2º O subsídio dos Deputados Estaduais será fixado por lei de iniciativa da Assembléia Legislativa, na razão de, no máximo, setenta e cinco por cento daquele estabelecido, em espécie, para os Deputados Federais, observado o que dispõem os arts. 39, § 4º, 57, § 7º, 150, II, 153, III, e 153, § 2º, I. (Redação dada pela Emenda Constitucional nº 19, de 1998)

§ 3º Compete às Assembléias Legislativas dispor sobre seu regimento interno, polícia e serviços administrativos de sua secretaria, e prover os respectivos cargos.

§ 4º A lei disporá sobre a iniciativa popular no processo legislativo estadual.

Art. 28. A eleição do Governador e do Vice-Governador de Estado, para mandato de quatro anos, realizar-se-á no primeiro domingo de outubro, em primeiro turno, e no último domingo de outubro, em segundo turno, se houver, do ano anterior ao do término do mandato de seus

antecessores, e a posse ocorrerá em primeiro de janeiro do ano subseqüente, observado, quanto ao mais, o disposto no art. 77. (Redação dada pela Emenda Constitucional nº 16, de1997)

Parágrafo único. Perderá o mandato o Governador que assumir outro cargo ou função na administração pública direta ou indireta, ressalvada a posse em virtude de concurso público e observado o disposto no art. 38, I, IV e V.

§ 1º Perderá o mandato o Governador que assumir outro cargo ou função na administração pública direta ou indireta, ressalvada a posse em virtude de concurso público e observado o disposto no art. 38, I, IV e V. (Renumerado do parágrafo único, pela Emenda Constitucional nº 19, de 1998)

§ 2º Os subsídios do Governador, do Vice-Governador e dos Secretários de Estado serão fixados por lei de iniciativa da Assembléia Legislativa, observado o que dispõem os arts. 37, XI, 39, § 4º, 150, II, 153, III, e 153, § 2º, I. (Incluído pela Emenda Constitucional nº 19, de 1998)

CAPÍTULO IV
Dos Municípios

Art. 29. O Município reger-se-á por lei orgânica, votada em dois turnos, com o interstício mínimo de dez dias, e aprovada por dois terços dos membros da Câmara Municipal, que a promulgará, atendidos os princípios estabelecidos nesta Constituição, na Constituição do respectivo Estado e os seguintes preceitos:

I - eleição do Prefeito, do Vice-Prefeito e dos Vereadores, para mandato de quatro anos, mediante pleito direto e simultâneo realizado em todo o País;

II - eleição do Prefeito e do Vice-Prefeito realizada no primeiro domingo de outubro do ano anterior ao término do mandato dos que devam suceder, aplicadas as regras do art. 77, no caso de Municípios com mais de duzentos mil eleitores; (Redação dada pela Emenda Constitucional nº 16, de1997)

III - posse do Prefeito e do Vice-Prefeito no dia 1º de janeiro do ano subseqüente ao da eleição;

IV - para a composição das Câmaras Municipais, será observado o limite máximo de: (Redação dada pela Emenda Constituição Constitucional nº 58, de 2009) (Produção de efeito) (Vide ADIN 4307)

a) 9 (nove) Vereadores, nos Municípios de até 15.000 (quinze mil) habitantes; (Redação dada pela Emenda Constituição Constitucional nº 58, de 2009)

b) 11 (onze) Vereadores, nos Municípios de mais de 15.000 (quinze mil) habitantes e de até 30.000 (trinta mil) habitantes; (Redação dada pela Emenda Constituição Constitucional nº 58, de 2009)

c) 13 (treze) Vereadores, nos Municípios com mais de 30.000 (trinta mil) habitantes e de até 50.000 (cinquenta mil) habitantes; (Redação dada pela Emenda Constituição Constitucional nº 58, de 2009)

d) 15 (quinze) Vereadores, nos Municípios de mais de 50.000 (cinquenta mil) habitantes e de até 80.000 (oitenta mil) habitantes; (Incluída pela Emenda Constituição Constitucional nº 58, de 2009)

e) 17 (dezessete) Vereadores, nos Municípios de mais de 80.000 (oitenta mil) habitantes e de até 120.000 (cento e vinte mil) habitantes; (Incluída pela Emenda Constituição Constitucional nº 58, de 2009)

f) 19 (dezenove) Vereadores, nos Municípios de mais de 120.000 (cento e vinte mil) habitantes e de até 160.000 (cento sessenta mil) habitantes; (Incluída pela Emenda Constituição Constitucional nº 58, de 2009)

g) 21 (vinte e um) Vereadores, nos Municípios de mais de 160.000 (cento e sessenta mil) habitantes e de até 300.000 (trezentos mil) habitantes; (Incluída pela Emenda Constituição Constitucional nº 58, de 2009)

h) 23 (vinte e três) Vereadores, nos Municípios de mais de 300.000 (trezentos mil) habitantes e de até 450.000 (quatrocentos e cinquenta mil) habitantes; (Incluída pela Emenda Constituição Constitucional nº 58, de 2009)

i) 25 (vinte e cinco) Vereadores, nos Municípios de mais de 450.000 (quatrocentos e cinquenta mil) habitantes e de até 600.000 (seiscentos mil) habitantes; (Incluída pela Emenda Constituição Constitucional nº 58, de 2009)

j) 27 (vinte e sete) Vereadores, nos Municípios de mais de 600.000 (seiscentos mil) habitantes e de até 750.000 (setecentos cinquenta mil) habitantes; (Incluída pela Emenda Constituição Constitucional nº 58, de 2009)

k) 29 (vinte e nove) Vereadores, nos Municípios de mais de 750.000 (setecentos e cinquenta mil) habitantes e de até 900.000 (novecentos mil) habitantes; (Incluída pela Emenda Constituição Constitucional nº 58, de 2009)

l) 31 (trinta e um) Vereadores, nos Municípios de mais de 900.000 (novecentos mil) habitantes e de até 1.050.000 (um milhão e cinquenta mil) habitantes; (Incluída pela Emenda Constituição Constitucional nº 58, de 2009)

m) 33 (trinta e três) Vereadores, nos Municípios de mais de 1.050.000 (um milhão e cinquenta mil) habitantes e de até 1.200.000 (um milhão e duzentos mil) habitantes; (Incluída pela Emenda Constituição Constitucional nº 58, de 2009)

n) 35 (trinta e cinco) Vereadores, nos Municípios de mais de 1.200.000 (um milhão e duzentos mil) habitantes e de até 1.350.000 (um milhão e trezentos e cinquenta mil) habitantes; (Incluída pela Emenda Constituição Constitucional nº 58, de 2009)

o) 37 (trinta e sete) Vereadores, nos Municípios de 1.350.000 (um milhão e trezentos e cinquenta mil) habitantes e de até 1.500.000 (um milhão e quinhentos mil) habitantes; (Incluída pela Emenda Constituição Constitucional nº 58, de 2009)

p) 39 (trinta e nove) Vereadores, nos Municípios de mais de 1.500.000 (um milhão e quinhentos mil) habitantes e de até 1.800.000 (um milhão e oitocentos mil) habitantes; (Incluída pela Emenda Constituição Constitucional nº 58, de 2009)

q) 41 (quarenta e um) Vereadores, nos Municípios de mais de 1.800.000 (um milhão e oitocentos mil) habitantes e de até 2.400.000 (dois milhões e quatrocentos mil) habitantes; (Incluída pela Emenda Constituição Constitucional nº 58, de 2009)

r) 43 (quarenta e três) Vereadores, nos Municípios de mais de 2.400.000 (dois milhões e quatrocentos mil) habitantes e de até 3.000.000 (três milhões) de habitantes; (Incluída pela Emenda Constituição Constitucional nº 58, de 2009)

s) 45 (quarenta e cinco) Vereadores, nos Municípios de mais de 3.000.000 (três milhões) de habitantes e de até 4.000.000 (quatro milhões) de habitantes; (Incluída pela Emenda Constituição Constitucional nº 58, de 2009)

t) 47 (quarenta e sete) Vereadores, nos Municípios de mais de 4.000.000 (quatro milhões) de habitantes e de até 5.000.000 (cinco milhões) de habitantes; (Incluída pela Emenda Constituição Constitucional nº 58, de 2009)

u) 49 (quarenta e nove) Vereadores, nos Municípios de mais de 5.000.000 (cinco milhões) de habitantes e de até 6.000.000 (seis milhões) de habitantes; (Incluída pela Emenda Constituição Constitucional nº 58, de 2009)

v) 51 (cinquenta e um) Vereadores, nos Municípios de mais de 6.000.000 (seis milhões) de habitantes e de até 7.000.000 (sete milhões) de habitantes; (Incluída pela Emenda Constituição Constitucional nº 58, de 2009)

w) 53 (cinquenta e três) Vereadores, nos Municípios de mais de 7.000.000 (sete milhões) de habitantes e de até 8.000.000 (oito milhões) de habitantes; e (Incluída pela Emenda Constituição Constitucional nº 58, de 2009)

x) 55 (cinquenta e cinco) Vereadores, nos Municípios de mais de 8.000.000 (oito milhões) de habitantes; (Incluída pela Emenda Constituição Constitucional nº 58, de 2009)

V - subsídios do Prefeito, do Vice-Prefeito e dos Secretários Municipais fixados por lei de iniciativa da Câmara Municipal, observado o que dispõem os arts. 37, XI, 39, § 4º, 150, II, 153, III, e 153, § 2º, I; (Redação dada pela Emenda constitucional nº 19, de 1998)

VI - subsídio dos Vereadores fixado por lei de iniciativa da Câmara Municipal, na razão de, no máximo, setenta e cinco por cento daquele estabelecido, em espécie, para os Deputados Estaduais, observado o que dispõem os arts. 39, § 4º, 57, § 7º, 150, II, 153, III, e 153, § 2º, I; (Redação dada pela Emenda constitucional nº 19, de 1998)

VI - o subsídio dos Vereadores será fixado pelas respectivas Câmaras Municipais em cada legislatura para a subseqüente, observado o que dispõe esta Constituição, observados os critérios estabelecidos na respectiva Lei Orgânica e os seguintes limites máximos: (Redação dada pela Emenda Constitucional nº 25, de 2000)

a) em Municípios de até dez mil habitantes, o subsídio máximo dos Vereadores corresponderá a vinte por cento do subsídio dos Deputados Estaduais; (Incluído pela Emenda Constitucional nº 25, de 2000)

b) em Municípios de dez mil e um a cinqüenta mil habitantes, o subsídio máximo dos Vereadores corresponderá a trinta por cento do subsídio dos Deputados Estaduais; (Incluído pela Emenda Constitucional nº 25, de 2000)

c) em Municípios de cinqüenta mil e um a cem mil habitantes, o subsídio máximo dos Vereadores corresponderá a quarenta por cento do subsídio dos Deputados Estaduais; (Incluído pela Emenda Constitucional nº 25, de 2000)

d) em Municípios de cem mil e um a trezentos mil habitantes, o subsídio máximo dos Vereadores corresponderá a cinqüenta por cento do subsídio dos Deputados Estaduais; (Incluído pela Emenda Constitucional nº 25, de 2000)

e) em Municípios de trezentos mil e um a quinhentos mil habitantes, o subsídio máximo dos Vereadores corresponderá a sessenta por cento do subsídio dos Deputados Estaduais; (Incluído pela Emenda Constitucional nº 25, de 2000)

f) em Municípios de mais de quinhentos mil habitantes, o subsídio máximo dos Vereadores corresponderá a setenta e cinco por cento do subsídio dos Deputados Estaduais; (Incluído pela Emenda Constitucional nº 25, de 2000)

VII - o total da despesa com a remuneração dos Vereadores não poderá ultrapassar o montante de cinco por cento da receita do Município; (Incluído pela Emenda Constitucional nº 1, de 1992)

VIII - inviolabilidade dos Vereadores por suas opiniões, palavras e votos no exercício do mandato e na circunscrição do Município; (Renumerado do inciso VI, pela Emenda Constitucional nº 1, de 1992)

IX - proibições e incompatibilidades, no exercício da vereança, similares, no que couber, ao disposto nesta Constituição para os membros do Congresso Nacional e na Constituição do respectivo Estado para os membros da Assembléia Legislativa; (Renumerado do inciso VII, pela Emenda Constitucional nº 1, de 1992)

X - julgamento do Prefeito perante o Tribunal de Justiça; (Renumerado do inciso VIII, pela Emenda Constitucional nº 1, de 1992)

XI - organização das funções legislativas e fiscalizadoras da Câmara Municipal; (Renumerado do inciso IX, pela Emenda Constitucional nº 1, de 1992)

XII - cooperação das associações representativas no planejamento municipal; (Renumerado do inciso X, pela Emenda Constitucional nº 1, de 1992)

XIII - iniciativa popular de projetos de lei de interesse específico do Município, da cidade ou de bairros, através de manifestação de, pelo menos, cinco por cento do eleitorado; (Renumerado do inciso XI, pela Emenda Constitucional nº 1, de 1992)

XIV - perda do mandato do Prefeito, nos termos do art. 28, parágrafo único . (Renumerado do inciso XII, pela Emenda Constitucional nº 1, de 1992)

~~I - oito por cento para Municípios com população de até cem mil habitantes; (Incluído pela Emenda Constitucional nº 25, de 2000)~~

I - 7% (sete por cento) para Municípios com população de até 100.000 (cem mil) habitantes; (Redação dada pela Emenda Constituição Constitucional nº 58, de 2009) (Produção de efeito)

~~II - sete por cento para Municípios com população entre cem mil e um e trezentos mil habitantes; (Incluído pela Emenda Constitucional nº 25, de 2000)~~

II - 6% (seis por cento) para Municípios com população entre 100.000 (cem mil) e 300.000 (trezentos mil) habitantes; (Redação dada pela Emenda Constituição Constitucional nº 58, de 2009)

~~III - seis por cento para Municípios com população entre trezentos mil e um e quinhentos mil habitantes; (Incluído pela Emenda Constitucional nº 25, de 2000)~~

III - 5% (cinco por cento) para Municípios com população entre 300.001 (trezentos mil e um) e 500.000 (quinhentos mil) habitantes; (Redação dada pela Emenda Constituição Constitucional nº 58, de 2009)

~~IV - cinco por cento para Municípios com população acima de quinhentos mil habitantes. (Incluído pela Emenda Constitucional nº 25, de 2000)~~

IV - 4,5% (quatro inteiros e cinco décimos por cento) para Municípios com população entre 500.001 (quinhentos mil e um) e 3.000.000 (três milhões) de habitantes; (Redação dada pela Emenda Constituição Constitucional nº 58, de 2009)

V - 4% (quatro por cento) para Municípios com população entre 3.000.001 (três milhões e um) e 8.000.000 (oito milhões) de habitantes; (Incluído pela Emenda Constituição Constitucional nº 58, de 2009)

VI - 3,5% (três inteiros e cinco décimos por cento) para Municípios com população acima de 8.000.001 (oito milhões e um) habitantes. (Incluído pela Emenda Constituição Constitucional nº 58, de 2009)

§ 1º A Câmara Municipal não gastará mais de setenta por cento de sua receita com folha de pagamento, incluído o gasto com o subsídio de seus Vereadores. (Incluído pela Emenda Constitucional nº 25, de 2000)

§ 2º Constitui crime de responsabilidade do Prefeito Municipal: (Incluído pela Emenda Constitucional nº 25, de 2000)

I - efetuar repasse que supere os limites definidos neste artigo; (Incluído pela Emenda Constitucional nº 25, de 2000)

II - não enviar o repasse até o dia vinte de cada mês; ou (Incluído pela Emenda Constitucional nº 25, de 2000)

III - enviá-lo a menor em relação à proporção fixada na Lei Orçamentária. (Incluído pela Emenda Constitucional nº 25, de 2000)

§ 3º Constitui crime de responsabilidade do Presidente da Câmara Municipal o desrespeito ao § 1º deste artigo. (Incluído pela Emenda Constitucional nº 25, de 2000)

Art. 29-A. O total da despesa do Poder Legislativo Municipal, incluídos os subsídios dos Vereadores e excluídos os gastos com inativos, não poderá ultrapassar os seguintes percentuais, relativos ao somatório da receita tributária e das transferências previstas no § 5º do art. 153 e nos arts. 158 e 159, efetivamente realizado no exercício anterior: (Incluído pela Emenda Constitucional nº 25, de 2000) (Vide Emenda Constitucional nº 109, de 2021) (Vigência)

~~I - oito por cento para Municípios com população de até cem mil habitantes; (Incluído pela Emenda Constitucional nº 25, de 2000)~~

I - 7% (sete por cento) para Municípios com população de até 100.000 (cem mil) habitantes; (Redação dada pela Emenda Constituição Constitucional nº 58, de 2009) (Produção de efeito)

~~II - sete por cento para Municípios com população entre cem mil e um e trezentos mil habitantes; (Incluído pela Emenda Constitucional nº 25, de 2000)~~

II - 6% (seis por cento) para Municípios com população entre 100.000 (cem mil) e 300.000 (trezentos mil) habitantes; (Redação dada pela Emenda Constituição Constitucional nº 58, de 2009)

~~III - seis por cento para Municípios com população entre trezentos mil e um e quinhentos mil habitantes; (Incluído pela Emenda Constitucional nº 25, de 2000)~~

III - 5% (cinco por cento) para Municípios com população entre 300.001 (trezentos mil e um) e 500.000 (quinhentos mil) habitantes; (Redação dada pela Emenda Constituição Constitucional nº 58, de 2009)

~~IV - cinco por cento para Municípios com população acima de quinhentos mil habitantes. (Incluído pela Emenda Constitucional nº 25, de 2000)~~

IV - 4,5% (quatro inteiros e cinco décimos por cento) para Municípios com população entre 500.001 (quinhentos mil e um) e 3.000.000 (três milhões) de habitantes; (Redação dada pela Emenda Constituição Constitucional nº 58, de 2009)

V - 4% (quatro por cento) para Municípios com população entre 3.000.001 (três milhões e um) e 8.000.000 (oito milhões) de habitantes; (Incluído pela Emenda Constituição Constitucional nº 58, de 2009)

VI - 3,5% (três inteiros e cinco décimos por cento) para Municípios com população acima de 8.000.001 (oito milhões e um) habitantes. (Incluído pela Emenda Constituição Constitucional nº 58, de 2009)

§ 1 º A Câmara Municipal não gastará mais de setenta por cento de sua receita com folha de pagamento, incluído o gasto com o subsídio de seus Vereadores. (Incluído pela Emenda Constitucional nº 25, de 2000)

§ 2 º Constitui crime de responsabilidade do Prefeito Municipal: (Incluído pela Emenda Constitucional nº 25, de 2000)

I - efetuar repasse que supere os limites definidos neste artigo; (Incluído pela Emenda Constitucional nº 25, de 2000)

II - não enviar o repasse até o dia vinte de cada mês; ou (Incluído pela Emenda Constitucional nº 25, de 2000)

III - enviá-lo a menor em relação à proporção fixada na Lei Orçamentária. (Incluído pela Emenda Constitucional nº 25, de 2000)

§ 3 º Constitui crime de responsabilidade do Presidente da Câmara Municipal o desrespeito ao § 1 º deste artigo. (Incluído pela Emenda Constitucional nº 25, de 2000)

Art. 30. Compete aos Municípios:

I - legislar sobre assuntos de interesse local;

II - suplementar a legislação federal e a estadual no que couber; (Vide ADPF 672)

III - instituir e arrecadar os tributos de sua competência, bem como aplicar suas rendas, sem prejuízo da obrigatoriedade de prestar contas e publicar balancetes nos prazos fixados em lei;

IV - criar, organizar e suprimir distritos, observada a legislação estadual;

V - organizar e prestar, diretamente ou sob regime de concessão ou permissão, os serviços públicos de interesse local, incluído o de transporte coletivo, que tem caráter essencial;

~~VI - manter, com a cooperação técnica e financeira da União e do Estado, programas de educação pré-escolar e de ensino fundamental;~~

VI - manter, com a cooperação técnica e financeira da União e do Estado, programas de educação infantil e de ensino fundamental; (Redação dada pela Emenda Constitucional nº 53, de 2006)

VII - prestar, com a cooperação técnica e financeira da União e do Estado, serviços de atendimento à saúde da população;

VIII - promover, no que couber, adequado ordenamento territorial, mediante planejamento e controle do uso, do parcelamento e da ocupação do solo urbano;

IX - promover a proteção do patrimônio histórico-cultural local, observada a legislação e a ação fiscalizadora federal e estadual.

Art. 31. A fiscalização do Município será exercida pelo Poder Legislativo Municipal, mediante controle externo, e pelos sistemas de controle interno do Poder Executivo Municipal, na forma da lei.

§ 1º O controle externo da Câmara Municipal será exercido com o auxílio dos Tribunais de Contas dos Estados ou do Município ou dos Conselhos ou Tribunais de Contas dos Municípios, onde houver.

§ 2º O parecer prévio, emitido pelo órgão competente sobre as contas que o Prefeito deve anualmente prestar, só deixará de prevalecer por decisão de dois terços dos membros da Câmara Municipal.

§ 3º As contas dos Municípios ficarão, durante sessenta dias, anualmente, à disposição de qualquer contribuinte, para exame e apreciação, o qual poderá questionar-lhes a legitimidade, nos termos da lei.

§ 4º É vedada a criação de Tribunais, Conselhos ou órgãos de Contas Municipais.

CAPÍTULO V
DO DISTRITO FEDERAL E DOS TERRITÓRIOS

SEÇÃO I
DO DISTRITO FEDERAL

Art. 32. O Distrito Federal, vedada sua divisão em Municípios, reger- se-á por lei orgânica, votada em dois turnos com interstício mínimo de dez dias, e aprovada por dois terços da Câmara Legislativa, que a promulgará, atendidos os princípios estabelecidos nesta Constituição.

§ 1º Ao Distrito Federal são atribuídas as competências legislativas reservadas aos Estados e Municípios.

§ 2º A eleição do Governador e do Vice-Governador, observadas as regras do art. 77, e dos Deputados Distritais coincidirá com a dos Governadores e Deputados Estaduais, para mandato de igual duração.

§ 3º Aos Deputados Distritais e à Câmara Legislativa aplica-se o disposto no art. 27.

~~§ 4º Lei federal disporá sobre a utilização, pelo Governo do Distrito Federal, das polícias civil e militar e do corpo de bombeiros militar.~~

SEÇÃO II
DOS TERRITÓRIOS

Art. 33. A lei disporá sobre a organização administrativa e judiciária dos Territórios.

§ 1º Os Territórios poderão ser divididos em Municípios, aos quais se aplicará, no que couber, o disposto no Capítulo IV deste Título.

§ 2º As contas do Governo do Território serão submetidas ao Congresso Nacional, com parecer prévio do Tribunal de Contas da União.

§ 3º Nos Territórios Federais com mais de cem mil habitantes, além do Governador nomeado na forma desta Constituição, haverá órgãos judiciários de primeira e segunda instância, membros do Ministério Público e defensores públicos federais; a lei disporá sobre as eleições para a Câmara Territorial e sua competência deliberativa.

CAPÍTULO VI
DA INTERVENÇÃO

Art. 34. A União não intervirá nos Estados nem no Distrito Federal, exceto para:

I - manter a integridade nacional;

II - repelir invasão estrangeira ou de uma unidade da Federação em outra;

III - pôr termo a grave comprometimento da ordem pública;

IV - garantir o livre exercício de qualquer dos Poderes nas unidades da Federação;

V - reorganizar as finanças da unidade da Federação que:

a) suspender o pagamento da dívida fundada por mais de dois anos consecutivos, salvo motivo de força maior;

b) deixar de entregar aos Municípios receitas tributárias fixadas nesta Constituição, dentro dos prazos estabelecidos em lei;

VI - prover a execução de lei federal, ordem ou decisão judicial;

VII - assegurar a observância dos seguintes princípios constitucionais:

a) forma republicana, sistema representativo e regime democrático;

b) direitos da pessoa humana;

c) autonomia municipal;

d) prestação de contas da administração pública, direta e indireta.

e) aplicação do mínimo exigido da receita resultante de impostos estaduais, compreendida a proveniente de transferências, na manutenção e desenvolvimento do ensino e nas ações e serviços públicos de saúde. (Redação dada pela Emenda Constitucional nº 29, de 2000)

Art. 35. O Estado não intervirá em seus Municípios, nem a União nos Municípios localizados em Território Federal, exceto quando:

I - deixar de ser paga, sem motivo de força maior, por dois anos consecutivos, a dívida fundada;

II - não forem prestadas contas devidas, na forma da lei;

III - não tiver sido aplicado o mínimo exigido da receita municipal na manutenção e desenvolvimento do ensino e nas ações e serviços públicos de saúde; (Redação dada pela Emenda Constitucional nº 29, de 2000)

IV - o Tribunal de Justiça der provimento a representação para assegurar a observância de princípios indicados na Constituição Estadual, ou para prover a execução de lei, de ordem ou de decisão judicial.

Art. 36. A decretação da intervenção dependerá:

I - no caso do art. 34, IV, de solicitação do Poder Legislativo ou do Poder Executivo coacto ou impedido, ou de requisição do Supremo Tribunal Federal, se a coação for exercida contra o Poder Judiciário;

II - no caso de desobediência a ordem ou decisão judiciária, de requisição do Supremo Tribunal Federal, do Superior Tribunal de Justiça ou do Tribunal Superior Eleitoral;

III - de provimento, pelo Supremo Tribunal Federal, de representação do Procurador-Geral da República, na hipótese do art. 34, VII, e no caso de recusa à execução de lei federal. (Redação dada pela Emenda Constitucional nº 45, de 2004)

~~IV - de provimento, pelo Superior Tribunal de Justiça, de representação do Procurador-Geral da República, no caso de recusa à execução de lei federal~~. (Revogado pela Emenda Constitucional nº 45, de 2004)

§ 1º O decreto de intervenção, que especificará a amplitude, o prazo e as condições de execução e que, se couber, nomeará o interventor, será submetido à apreciação do Congresso Nacional ou da Assembléia Legislativa do Estado, no prazo de vinte e quatro horas.

§ 2º Se não estiver funcionando o Congresso Nacional ou a Assembléia Legislativa, far-se-á convocação extraordinária, no mesmo prazo de vinte e quatro horas.

§ 3º Nos casos do art. 34, VI e VII, ou do art. 35, IV, dispensada a apreciação pelo Congresso Nacional ou pela Assembléia Legislativa, o decreto limitar-se-á a suspender a execução do ato impugnado, se essa medida bastar ao restabelecimento da normalidade.

§ 4º Cessados os motivos da intervenção, as autoridades afastadas de seus cargos a estes voltarão, salvo impedimento legal.

CAPÍTULO VII
DA ADMINISTRAÇÃO PÚBLICA
SEÇÃO I
DISPOSIÇÕES GERAIS

Art. 37. A administração pública direta e indireta de qualquer dos Poderes da União, dos Estados, do Distrito Federal e dos Municípios obedecerá aos princípios de legalidade, impessoalidade, moralidade, publicidade e eficiência e, também, ao seguinte: (Redação dada pela Emenda Constitucional nº 19, de 1998)

I - os cargos, empregos e funções públicas são acessíveis aos brasileiros que preencham os requisitos estabelecidos em lei, assim como aos estrangeiros, na forma da lei; (Redação dada pela Emenda Constitucional nº 19, de 1998)

II - a investidura em cargo ou emprego público depende de aprovação prévia em concurso público de provas ou de provas e títulos, de acordo com a natureza e a complexidade do cargo ou emprego, na forma prevista em lei, ressalvadas as nomeações para cargo em comissão declarado em lei de livre nomeação e exoneração; (Redação dada pela Emenda Constitucional nº 19, de 1998)

III - o prazo de validade do concurso público será de até dois anos, prorrogável uma vez, por igual período;

IV - durante o prazo improrrogável previsto no edital de convocação, aquele aprovado em concurso público de provas ou de provas e títulos será convocado com prioridade sobre novos concursados para assumir cargo ou emprego, na carreira;

V - as funções de confiança, exercidas exclusivamente por servidores ocupantes de cargo efetivo, e os cargos em comissão, a serem preenchidos por servidores de carreira nos casos, condições e percentuais mínimos previstos em lei, destinam-se apenas às atribuições de direção, chefia e assessoramento; (Redação dada pela Emenda Constitucional nº 19, de 1998)

VI - é garantido ao servidor público civil o direito à livre associação sindical;

VII - o direito de greve será exercido nos termos e nos limites definidos em lei específica; (Redação dada pela Emenda Constitucional nº 19, de 1998)

VIII - a lei reservará percentual dos cargos e empregos públicos para as pessoas portadoras de deficiência e definirá os critérios de sua admissão;

X - a remuneração dos servidores públicos e o subsídio de que trata o § 4º do art. 39 somente poderão ser fixados ou alterados por lei específica, observada a iniciativa privativa em cada caso, assegurada revisão geral anual, sempre na mesma data e sem distinção de índices; (Redação dada pela Emenda Constitucional nº 19, de 1998) (Regulamento)

XI - a remuneração e o subsídio dos ocupantes de cargos, funções e empregos públicos da administração direta, autárquica e fundacional, dos membros de qualquer dos Poderes da União, dos Estados, do Distrito Federal e dos Municípios, dos detentores de mandato eletivo e dos demais agentes políticos e os proventos, pensões ou outra espécie remuneratória, percebidos cumulativamente ou não, incluídas as vantagens pessoais ou de qualquer outra natureza, não poderão exceder o subsídio mensal, em espécie, dos Ministros do Supremo Tribunal Federal, aplicando-se como limite, nos Municípios, o subsídio do Prefeito, e nos Estados e no Distrito Federal, o subsídio mensal do Governador no âmbito do Poder Executivo, o subsídio dos Deputados Estaduais e Distritais no âmbito do Poder Legislativo e o subsídio dos Desembargadores do Tribunal de Justiça, limitado a noventa inteiros e vinte e cinco centésimos por cento do subsídio mensal, em espécie, dos Ministros do Supremo Tribunal Federal, no âmbito do Poder Judiciário, aplicável este limite aos membros do Ministério Público, aos Procuradores e aos Defensores Públicos; (Redação dada pela Emenda Constitucional nº 41, 19.12.2003)

XII - os vencimentos dos cargos do Poder Legislativo e do Poder Judiciário não poderão ser superiores aos pagos pelo Poder Executivo;

XIII - é vedada a vinculação ou equiparação de quaisquer espécies remuneratórias para o efeito de remuneração de pessoal do serviço público; (Redação dada pela Emenda Constitucional nº 19, de 1998)

XIV - os acréscimos pecuniários percebidos por servidor público não serão computados nem acumulados para fins de concessão de acréscimos ulteriores; (Redação dada pela Emenda Constitucional nº 19, de 1998)

XV - o subsídio e os vencimentos dos ocupantes de cargos e empregos públicos são irredutíveis, ressalvado o disposto nos incisos XI e XIV deste artigo e nos arts. 39, § 4º, 150, II, 153, III, e 153, § 2º, I; (Redação dada pela Emenda Constitucional nº 19, de 1998)

XVI - é vedada a acumulação remunerada de cargos públicos, exceto, quando houver compatibilidade de horários, observado em qualquer caso o disposto no inciso XI: (Redação dada pela Emenda Constitucional nº 19, de 1998)

a) a de dois cargos de professor; (Redação dada pela Emenda Constitucional nº 19, de 1998)

b) a de um cargo de professor com outro técnico ou científico; (Redação dada pela Emenda Constitucional nº 19, de 1998)

~~c) a de dois cargos privativos de médico; (Redação dada pela Emenda Constitucional nº 19, de 1998)~~

c) a de dois cargos ou empregos privativos de profissionais de saúde, com profissões regulamentadas; (Redação dada pela Emenda Constitucional nº 34, de 2001)

XVII - a proibição de acumular estende-se a empregos e funções e abrange autarquias, fundações, empresas públicas, sociedades de economia mista, suas subsidiárias, e sociedades controladas, direta ou indiretamente, pelo poder público; (Redação dada pela Emenda Constitucional nº 19, de 1998)

XVIII - a administração fazendária e seus servidores fiscais terão, dentro de suas áreas de competência e jurisdição, precedência sobre os demais setores administrativos, na forma da lei;

XIX - somente por lei específica poderá ser criada autarquia e autorizada a instituição de empresa pública, de sociedade de economia mista e de fundação, cabendo à lei complementar, neste último caso, definir as áreas de sua atuação; (Redação dada pela Emenda Constitucional nº 19, de 1998)

XX - depende de autorização legislativa, em cada caso, a criação de subsidiárias das entidades mencionadas no inciso anterior, assim como a participação de qualquer delas em empresa privada;

XXI - ressalvados os casos especificados na legislação, as obras, serviços, compras e alienações serão contratados mediante processo de licitação pública que assegure igualdade de condições a todos os concorrentes, com cláusulas que estabeleçam obrigações de pagamento, mantidas as condições efetivas da proposta, nos termos da lei, o qual somente permitirá as exigências de qualificação técnica e econômica indispensáveis à garantia do cumprimento das obrigações. (Regulamento)

XXII - as administrações tributárias da União, dos Estados, do Distrito Federal e dos Municípios, atividades essenciais ao funcionamento do Estado, exercidas por servidores de carreiras específicas, terão recursos prioritários para a realização de suas atividades e atuarão de forma integrada, inclusive com o compartilhamento de cadastros e de informações fiscais, na forma da lei ou convênio. (Incluído pela Emenda Constitucional nº 42, de 19.12.2003)

§ 1º A publicidade dos atos, programas, obras, serviços e campanhas dos órgãos públicos deverá ter caráter educativo, informativo ou de orientação social, dela não podendo constar nomes, símbolos ou imagens que caracterizem promoção pessoal de autoridades ou servidores públicos.

§ 2º A não observância do disposto nos incisos II e III implicará a nulidade do ato e a punição da autoridade responsável, nos termos da lei.

§ 3º A lei disciplinará as formas de participação do usuário na administração pública direta e indireta, regulando especialmente: (Redação dada pela Emenda Constitucional nº 19, de 1998)

I - as reclamações relativas à prestação dos serviços públicos em geral, asseguradas a manutenção de serviços de atendimento ao usuário e a avaliação periódica, externa e interna, da qualidade dos serviços; (Incluído pela Emenda Constitucional nº 19, de 1998)

II - o acesso dos usuários a registros administrativos e a informações sobre atos de governo, observado o disposto no art. 5º, X e XXXIII; (Incluído pela Emenda Constitucional nº 19, de 1998) (Vide Lei nº 12.527, de 2011)

III - a disciplina da representação contra o exercício negligente ou abusivo de cargo, emprego ou função na administração pública. (Incluído pela Emenda Constitucional nº 19, de 1998)

§ 4º Os atos de improbidade administrativa importarão a suspensão dos direitos políticos, a perda da função pública, a indisponibilidade dos bens e o ressarcimento ao erário, na forma e gradação previstas em lei, sem prejuízo da ação penal cabível.

§ 5º A lei estabelecerá os prazos de prescrição para ilícitos praticados por qualquer agente, servidor ou não, que causem prejuízos ao erário, ressalvadas as respectivas ações de ressarcimento.

§ 6º As pessoas jurídicas de direito público e as de direito privado prestadoras de serviços públicos responderão pelos danos que seus agentes, nessa qualidade, causarem a terceiros, assegurado o direito de regresso contra o responsável nos casos de dolo ou culpa.

§ 7º A lei disporá sobre os requisitos e as restrições ao ocupante de cargo ou emprego da administração direta e indireta que possibilite o acesso a informações privilegiadas. (Incluído pela Emenda Constitucional nº 19, de 1998)

§ 8º A autonomia gerencial, orçamentária e financeira dos órgãos e entidades da administração direta e indireta poderá ser ampliada mediante contrato, a ser firmado entre seus administradores e o poder público, que tenha por objeto a fixação de metas de desempenho para o órgão ou entidade, cabendo à lei dispor sobre: (Incluído pela Emenda Constitucional nº 19, de 1998) (Regulamento) (Vigência)

I - o prazo de duração do contrato; (Incluído pela Emenda Constitucional nº 19, de 1998)

II - os controles e critérios de avaliação de desempenho, direitos, obrigações e responsabilidade dos dirigentes; (Incluído pela Emenda Constitucional nº 19, de 1998)

III - a remuneração do pessoal. (Incluído pela Emenda Constitucional nº 19, de 1998)

§ 9º O disposto no inciso XI aplica-se às empresas públicas e às sociedades de economia mista, e suas subsidiárias, que receberem recursos da União, dos Estados, do Distrito Federal ou dos Municípios para pagamento de despesas de pessoal ou de custeio em geral. (Incluído pela Emenda Constitucional nº 19, de 1998)

§ 10. É vedada a percepção simultânea de proventos de aposentadoria decorrentes do art. 40 ou dos arts. 42 e 142 com a remuneração de cargo, emprego ou função pública, ressalvados os cargos acumuláveis na forma desta Constituição, os cargos eletivos e os cargos em comissão declarados em lei de livre nomeação e exoneração. (Incluído pela Emenda Constitucional nº 20, de 1998) (Vide Emenda Constitucional nº 20, de 1998)

§ 11. Não serão computadas, para efeito dos limites remuneratórios de que trata o inciso XI do caput deste artigo, as parcelas de caráter indenizatório previstas em lei. (Incluído pela Emenda Constitucional nº 47, de 2005)

§ 12. Para os fins do disposto no inciso XI do caput deste artigo, fica facultado aos Estados e ao Distrito Federal fixar, em seu âmbito, mediante emenda às respectivas Constituições e Lei Orgânica, como limite único, o subsídio mensal dos Desembargadores do respectivo Tribunal de Justiça, limitado a noventa inteiros e vinte e cinco centésimos por cento do subsídio mensal dos Ministros do Supremo Tribunal Federal, não se aplicando o disposto neste parágrafo aos subsídios dos Deputados Estaduais e Distritais e dos Vereadores. (Incluído pela Emenda Constitucional nº 47, de 2005)

Art. 38. Ao servidor público da administração direta, autárquica e fundacional, no exercício de mandato eletivo, aplicam-se as seguintes disposições: (Redação dada pela Emenda Constitucional nº 19, de 1998)

I - tratando-se de mandato eletivo federal, estadual ou distrital, ficará afastado de seu cargo, emprego ou função;

II - investido no mandato de Prefeito, será afastado do cargo, emprego ou função, sendo-lhe facultado optar pela sua remuneração;

III - investido no mandato de Vereador, havendo compatibilidade de horários, perceberá as vantagens de seu cargo, emprego ou função, sem prejuízo da remuneração do cargo eletivo, e, não havendo compatibilidade, será aplicada a norma do inciso anterior;

IV - em qualquer caso que exija o afastamento para o exercício de mandato eletivo, seu tempo de serviço será contado para todos os efeitos legais, exceto para promoção por merecimento;

V - para efeito de benefício previdenciário, no caso de afastamento, os valores serão determinados como se no exercício estivesse.

SEÇÃO II

DOS SERVIDORES PÚBLICOS

(Redação dada pela Emenda Constitucional nº 18, de 1998)

~~Art. 39. A União, os Estados, o Distrito Federal e os Municípios instituirão, no âmbito de sua competência, regime jurídico único e planos de carreira para os servidores da administração pública direta, das autarquias e das fundações públicas.~~ (Vide ADI nº 2.135)

Art. 39. A União, os Estados, o Distrito Federal e os Municípios instituirão conselho de política de administração e remuneração de pessoal, integrado por servidores designados pelos respectivos Poderes. (Redação dada pela Emenda Constitucional nº 19, de 1998) (Vide ADI nº 2.135)

§ 1º A fixação dos padrões de vencimento e dos demais componentes do sistema remuneratório observará: (Redação dada pela Emenda Constitucional nº 19, de 1998)

I - a natureza, o grau de responsabilidade e a complexidade dos cargos componentes de cada carreira; (Incluído pela Emenda Constitucional nº 19, de 1998)

II - os requisitos para a investidura; (Incluído pela Emenda Constitucional nº 19, de 1998)

III - as peculiaridades dos cargos. (Incluído pela Emenda Constitucional nº 19, de 1998)

§ 2º A União, os Estados e o Distrito Federal manterão escolas de governo para a formação e o aperfeiçoamento dos servidores públicos, constituindo-se a participação nos cursos um dos requisitos para a promoção na carreira, facultada, para isso, a celebração de convênios ou contratos entre os entes federados. (Redação dada pela Emenda Constitucional nº 19, de 1998)

§ 3º Aplica-se aos servidores ocupantes de cargo público o disposto no art. 7º, IV, VII, VIII, IX, XII, XIII, XV, XVI, XVII, XVIII, XIX, XX, XXII e XXX, podendo a lei estabelecer requisitos diferenciados de admissão quando a natureza do cargo o exigir. (Incluído pela Emenda Constitucional nº 19, de 1998)

§ 4º O membro de Poder, o detentor de mandato eletivo, os Ministros de Estado e os Secretários Estaduais e Municipais serão remunerados exclusivamente por subsídio fixado em parcela única, vedado o acréscimo de qualquer gratificação, adicional, abono, prêmio, verba de

representação ou outra espécie remuneratória, obedecido, em qualquer caso, o disposto no art. 37, X e XI. (Incluído pela Emenda Constitucional nº 19, de 1998)

§ 5º Lei da União, dos Estados, do Distrito Federal e dos Municípios poderá estabelecer a relação entre a maior e a menor remuneração dos servidores públicos, obedecido, em qualquer caso, o disposto no art. 37, XI. (Incluído pela Emenda Constitucional nº 19, de 1998)

§ 6º Os Poderes Executivo, Legislativo e Judiciário publicarão anualmente os valores do subsídio e da remuneração dos cargos e empregos públicos. (Incluído pela Emenda Constitucional nº 19, de 1998)

§ 7º Lei da União, dos Estados, do Distrito Federal e dos Municípios disciplinará a aplicação de recursos orçamentários provenientes da economia com despesas correntes em cada órgão, autarquia e fundação, para aplicação no desenvolvimento de programas de qualidade e produtividade, treinamento e desenvolvimento, modernização, reaparelhamento e racionalização do serviço público, inclusive sob a forma de adicional ou prêmio de produtividade. (Incluído pela Emenda Constitucional nº 19, de 1998)

§ 8º A remuneração dos servidores públicos organizados em carreira poderá ser fixada nos termos do § 4º. (Incluído pela Emenda Constitucional nº 19, de 1998)

Art. 40. Aos servidores titulares de cargos efetivos da União, dos Estados, do Distrito Federal e dos Municípios, incluídas suas autarquias e fundações, é assegurado regime de previdência de caráter contributivo e solidário, mediante contribuição do respectivo ente público, dos servidores ativos e inativos e dos pensionistas, observados critérios que preservem o equilíbrio financeiro e atuarial e o disposto neste artigo. (Redação dada pela Emenda Constitucional nº 41, 19.12.2003)

§ 1º Os servidores abrangidos pelo regime de previdência de que trata este artigo serão aposentados, calculados os seus proventos a partir dos valores fixados na forma dos §§ 3º e 17: (Redação dada pela Emenda Constitucional nº 41, 19.12.2003)

I - por invalidez permanente, sendo os proventos proporcionais ao tempo de contribuição, exceto se decorrente de acidente em serviço, moléstia profissional ou doença grave, contagiosa ou incurável, na forma da lei; (Redação dada pela Emenda Constitucional nº 41, 19.12.2003)

II - compulsoriamente, com proventos proporcionais ao tempo de contribuição, aos 70 (setenta) anos de idade, ou aos 75 (setenta e cinco) anos de idade, na forma de lei complementar; (Redação dada pela Emenda Constitucional nº 88, de 2015) (Vide Lei Complementar nº 152, de 2015)

III - voluntariamente, desde que cumprido tempo mínimo de dez anos de efetivo exercício no serviço público e cinco anos no cargo efetivo em que se dará a aposentadoria, observadas as seguintes condições: (Redação dada pela Emenda Constitucional nº 20, de 1998)

a) sessenta anos de idade e trinta e cinco de contribuição, se homem, e cinqüenta e cinco anos de idade e trinta de contribuição, se mulher; (Redação dada pela Emenda Constitucional nº 20, de 1998) (Vide Emenda Constitucional nº 20, de 1998)

b) sessenta e cinco anos de idade, se homem, e sessenta anos de idade, se mulher, com proventos proporcionais ao tempo de contribuição (Redação dada pela Emenda Constitucional nº 20, de 1998)

§ 2º Os proventos de aposentadoria e as pensões, por ocasião de sua concessão, não poderão exceder a remuneração do respectivo servidor, no cargo efetivo em que se deu a aposentadoria ou que serviu de referência para a concessão da pensão. (Redação dada pela Emenda Constitucional nº 20, de 1998)

§ 3º Para o cálculo dos proventos de aposentadoria, por ocasião da sua concessão, serão consideradas as remunerações utilizadas como base para as contribuições do servidor aos

regimes de previdência de que tratam este artigo e o art. 201, na forma da lei. (Redação dada pela Emenda Constitucional nº 41, 19.12.2003)

§ 4º É vedada a adoção de requisitos e critérios diferenciados para a concessão de aposentadoria aos abrangidos pelo regime de que trata este artigo, ressalvados, nos termos definidos em leis complementares, os casos de servidores: (Redação dada pela Emenda Constitucional nº 47, de 2005)

I portadores de deficiência; (Incluído pela Emenda Constitucional nº 47, de 2005)

II que exerçam atividades de risco; (Incluído pela Emenda Constitucional nº 47, de 2005)

III cujas atividades sejam exercidas sob condições especiais que prejudiquem a saúde ou a integridade física. (Incluído pela Emenda Constitucional nº 47, de 2005)

§ 5º Os requisitos de idade e de tempo de contribuição serão reduzidos em cinco anos, em relação ao disposto no § 1º, III, "a", para o professor que comprove exclusivamente tempo de efetivo exercício das funções de magistério na educação infantil e no ensino fundamental e médio. (Redação dada pela Emenda Constitucional nº 20, de 15/12/98)

§ 6º Ressalvadas as aposentadorias decorrentes dos cargos acumuláveis na forma desta Constituição, é vedada a percepção de mais de uma aposentadoria à conta do regime de previdência previsto neste artigo. (Redação dada pela Emenda Constitucional nº 20, de 15/12/98)

§ 7º Lei disporá sobre a concessão do benefício de pensão por morte, que será igual: (Redação dada pela Emenda Constitucional nº 41, 19.12.2003)

I - ao valor da totalidade dos proventos do servidor falecido, até o limite máximo estabelecido para os benefícios do regime geral de previdência social de que trata o art. 201, acrescido de setenta por cento da parcela excedente a este limite, caso aposentado à data do óbito; ou (Incluído pela Emenda Constitucional nº 41, 19.12.2003) (Vide ADIN 3133)

II - ao valor da totalidade da remuneração do servidor no cargo efetivo em que se deu o falecimento, até o limite máximo estabelecido para os benefícios do regime geral de previdência social de que trata o art. 201, acrescido de setenta por cento da parcela excedente a este limite, caso em atividade na data do óbito. (Incluído pela Emenda Constitucional nº 41, 19.12.2003) (Vide ADIN 3133)

§ 8º É assegurado o reajustamento dos benefícios para preservar-lhes, em caráter permanente, o valor real, conforme critérios estabelecidos em lei. (Redação dada pela Emenda Constitucional nº 41, 19.12.2003)

§ 9º O tempo de contribuição federal, estadual ou municipal será contado para efeito de aposentadoria e o tempo de serviço correspondente para efeito de disponibilidade. (Incluído pela Emenda Constitucional nº 20, de 15/12/98)

§ 10 - A lei não poderá estabelecer qualquer forma de contagem de tempo de contribuição fictício. (Incluído pela Emenda Constitucional nº 20, de 15/12/98) (Vide Emenda Constitucional nº 20, de 1998)

§ 11 - Aplica-se o limite fixado no art. 37, XI, à soma total dos proventos de inatividade, inclusive quando decorrentes da acumulação de cargos ou empregos públicos, bem como de outras atividades sujeitas a contribuição para o regime geral de previdência social, e ao montante resultante da adição de proventos de inatividade com remuneração de cargo acumulável na forma desta Constituição, cargo em comissão declarado em lei de livre nomeação e exoneração, e de cargo eletivo. (Incluído pela Emenda Constitucional nº 20, de 15/12/98)

§ 12 - Além do disposto neste artigo, o regime de previdência dos servidores públicos titulares de cargo efetivo observará, no que couber, os requisitos e critérios fixados para o regime geral de previdência social. (Incluído pela Emenda Constitucional nº 20, de 15/12/98)

§ 13 - Ao servidor ocupante, exclusivamente, de cargo em comissão declarado em lei de livre nomeação e exoneração bem como de outro cargo temporário ou de emprego público,

aplica-se o regime geral de previdência social. (Incluído pela Emenda Constitucional nº 20, de 15/12/98)

§ 14 - A União, os Estados, o Distrito Federal e os Municípios, desde que instituam regime de previdência complementar para os seus respectivos servidores titulares de cargo efetivo, poderão fixar, para o valor das aposentadorias e pensões a serem concedidas pelo regime de que trata este artigo, o limite máximo estabelecido para os benefícios do regime geral de previdência social de que trata o art. 201. (Incluído pela Emenda Constitucional nº 20, de 15/12/98)

§ 15. O regime de previdência complementar de que trata o § 14 será instituído por lei de iniciativa do respectivo Poder Executivo, observado o disposto no art. 202 e seus parágrafos, no que couber, por intermédio de entidades fechadas de previdência complementar, de natureza pública, que oferecerão aos respectivos participantes planos de benefícios somente na modalidade de contribuição definida. (Redação dada pela Emenda Constitucional nº 41, 19.12.2003)

§ 16 - Somente mediante sua prévia e expressa opção, o disposto nos § § 14 e 15 poderá ser aplicado ao servidor que tiver ingressado no serviço público até a data da publicação do ato de instituição do correspondente regime de previdência complementar. (Incluído pela Emenda Constitucional nº 20, de 15/12/98)

§ 17. Todos os valores de remuneração considerados para o cálculo do benefício previsto no § 3° serão devidamente atualizados, na forma da lei. (Incluído pela Emenda Constitucional nº 41, 19.12.2003)

§ 18. Incidirá contribuição sobre os proventos de aposentadorias e pensões concedidas pelo regime de que trata este artigo que superem o limite máximo estabelecido para os benefícios do regime geral de previdência social de que trata o art. 201, com percentual igual ao estabelecido para os servidores titulares de cargos efetivos. (Incluído pela Emenda Constitucional nº 41, 19.12.2003) (Vide ADIN 3133) (Vide ADIN 3143) (Vide ADIN 3184)

§ 19. O servidor de que trata este artigo que tenha completado as exigências para aposentadoria voluntária estabelecidas no § 1º, III, a, e que opte por permanecer em atividade fará jus a um abono de permanência equivalente ao valor da sua contribuição previdenciária até completar as exigências para aposentadoria compulsória contidas no § 1º, II. (Incluído pela Emenda Constitucional nº 41, 19.12.2003)

§ 20. Fica vedada a existência de mais de um regime próprio de previdência social para os servidores titulares de cargos efetivos, e de mais de uma unidade gestora do respectivo regime em cada ente estatal, ressalvado o disposto no art. 142, § 3º, X. (Incluído pela Emenda Constitucional nº 41, 19.12.2003)

§ 21. A contribuição prevista no § 18 deste artigo incidirá apenas sobre as parcelas de proventos de aposentadoria e de pensão que superem o dobro do limite máximo estabelecido para os benefícios do regime geral de previdência social de que trata o art. 201 desta Constituição, quando o beneficiário, na forma da lei, for portador de doença incapacitante. (Incluído pela Emenda Constitucional nº 47, de 2005)(Revogado pela Emenda Constitucional nº 103, de 2019) (Vigência) (Vide Emenda Constitucional nº 103, de 2019)

Art. 41. São estáveis após três anos de efetivo exercício os servidores nomeados para cargo de provimento efetivo em virtude de concurso público. (Redação dada pela Emenda Constitucional nº 19, de 1998)

§ 1º O servidor público estável só perderá o cargo: (Redação dada pela Emenda Constitucional nº 19, de 1998)

I - em virtude de sentença judicial transitada em julgado; (Incluído pela Emenda Constitucional nº 19, de 1998)

II - mediante processo administrativo em que lhe seja assegurada ampla defesa; (Incluído pela Emenda Constitucional nº 19, de 1998)

III - mediante procedimento de avaliação periódica de desempenho, na forma de lei complementar, assegurada ampla defesa. (Incluído pela Emenda Constitucional nº 19, de 1998)

§ 2º Invalidada por sentença judicial a demissão do servidor estável, será ele reintegrado, e o eventual ocupante da vaga, se estável, reconduzido ao cargo de origem, sem direito a indenização, aproveitado em outro cargo ou posto em disponibilidade com remuneração proporcional ao tempo de serviço. (Redação dada pela Emenda Constitucional nº 19, de 1998)

§ 3º Extinto o cargo ou declarada a sua desnecessidade, o servidor estável ficará em disponibilidade, com remuneração proporcional ao tempo de serviço, até seu adequado aproveitamento em outro cargo. (Redação dada pela Emenda Constitucional nº 19, de 1998)

§ 4º Como condição para a aquisição da estabilidade, é obrigatória a avaliação especial de desempenho por comissão instituída para essa finalidade. (Incluído pela Emenda Constitucional nº 19, de 1998)

SEÇÃO III

~~DOS SERVIDORES PÚBLICOS MILITARES~~

DOS MILITARES DOS ESTADOS, DO DISTRITO FEDERAL E DOS TERRITÓRIOS

(Redação dada pela Emenda Constitucional nº 18, de 1998)

Art. 42 Os membros das Polícias Militares e Corpos de Bombeiros Militares, instituições organizadas com base na hierarquia e disciplina, são militares dos Estados, do Distrito Federal e dos Territórios. (Redação dada pela Emenda Constitucional nº 18, de 1998)

§ 1º Aplicam-se aos militares dos Estados, do Distrito Federal e dos Territórios, além do que vier a ser fixado em lei, as disposições do art. 14, § 8º; do art. 40, § 9º; e do art. 142, §§ 2º e 3º, cabendo a lei estadual específica dispor sobre as matérias do art. 142, § 3º, inciso X, sendo as patentes dos oficiais conferidas pelos respectivos governadores. (Redação dada pela Emenda Constitucional nº 20, de 15/12/98)

§ 2º Aos pensionistas dos militares dos Estados, do Distrito Federal e dos Territórios aplica-se o que for fixado em lei específica do respectivo ente estatal. (Redação dada pela Emenda Constitucional nº 41, 19.12.2003)

SEÇÃO IV
DAS REGIÕES

Art. 43. Para efeitos administrativos, a União poderá articular sua ação em um mesmo complexo geoeconômico e social, visando a seu desenvolvimento e à redução das desigualdades regionais.

§ 1º Lei complementar disporá sobre:

I - as condições para integração de regiões em desenvolvimento;

II - a composição dos organismos regionais que executarão, na forma da lei, os planos regionais, integrantes dos planos nacionais de desenvolvimento econômico e social, aprovados juntamente com estes.

§ 2º Os incentivos regionais compreenderão, além de outros, na forma da lei:

I - igualdade de tarifas, fretes, seguros e outros itens de custos e preços de responsabilidade do Poder Público;

II - juros favorecidos para financiamento de atividades prioritárias;

III - isenções, reduções ou diferimento temporário de tributos federais devidos por pessoas físicas ou jurídicas;

IV - prioridade para o aproveitamento econômico e social dos rios e das massas de água represadas ou represáveis nas regiões de baixa renda, sujeitas a secas periódicas.

§ 3º Nas áreas a que se refere o § 2º, IV, a União incentivará a recuperação de terras áridas e cooperará com os pequenos e médios proprietários rurais para o estabelecimento, em suas glebas, de fontes de água e de pequena irrigação.

~~TÍTULO IV~~
~~DA ORGANIZAÇÃO DOS PODERES~~

TÍTULO IV
DA ORGANIZAÇÃO DOS PODERES

(Redação dada pela Emenda Constitucional nº 80, de 2014)

CAPÍTULO I
DO PODER LEGISLATIVO

SEÇÃO I
DO CONGRESSO NACIONAL

Art. 44. O Poder Legislativo é exercido pelo Congresso Nacional, que se compõe da Câmara dos Deputados e do Senado Federal.

Parágrafo único. Cada legislatura terá a duração de quatro anos.

Art. 45. A Câmara dos Deputados compõe-se de representantes do povo, eleitos, pelo sistema proporcional, em cada Estado, em cada Território e no Distrito Federal.

§ 1º O número total de Deputados, bem como a representação por Estado e pelo Distrito Federal, será estabelecido por lei complementar, proporcionalmente à população, procedendo-se aos ajustes necessários, no ano anterior às eleições, para que nenhuma daquelas unidades da Federação tenha menos de oito ou mais de setenta Deputados. (Vide Lei Complementar nº 78, de 1993)

§ 2º Cada Território elegerá quatro Deputados.

Art. 46. O Senado Federal compõe-se de representantes dos Estados e do Distrito Federal, eleitos segundo o princípio majoritário.

§ 1º Cada Estado e o Distrito Federal elegerão três Senadores, com mandato de oito anos.

§ 2º A representação de cada Estado e do Distrito Federal será renovada de quatro em quatro anos, alternadamente, por um e dois terços.

§ 3º Cada Senador será eleito com dois suplentes.

Art. 47. Salvo disposição constitucional em contrário, as deliberações de cada Casa e de suas Comissões serão tomadas por maioria dos votos, presente a maioria absoluta de seus membros.

SEÇÃO II
DAS ATRIBUIÇÕES DO CONGRESSO NACIONAL

Art. 48. Cabe ao Congresso Nacional, com a sanção do Presidente da República, não exigida esta para o especificado nos arts. 49, 51 e 52, dispor sobre todas as matérias de competência da União, especialmente sobre:

I - sistema tributário, arrecadação e distribuição de rendas;

II - plano plurianual, diretrizes orçamentárias, orçamento anual, operações de crédito, dívida pública e emissões de curso forçado;

III - fixação e modificação do efetivo das Forças Armadas;

IV - planos e programas nacionais, regionais e setoriais de desenvolvimento;

V - limites do território nacional, espaço aéreo e marítimo e bens do domínio da União;

VI - incorporação, subdivisão ou desmembramento de áreas de Territórios ou Estados, ouvidas as respectivas Assembléias Legislativas;

VII - transferência temporária da sede do Governo Federal;

VIII - concessão de anistia;

~~IX - organização administrativa, judiciária, do Ministério Público e da Defensoria Pública da União e dos Territórios e organização judiciária, do Ministério Público e da Defensoria Pública do Distrito Federal;~~

IX - organização administrativa, judiciária, do Ministério Público e da Defensoria Pública da União e dos Territórios e organização judiciária e do Ministério Público do Distrito Federal; (Redação dada pela Emenda Constitucional nº 69, de 2012) (Produção de efeito)

X - criação, transformação e extinção de cargos, empregos e funções públicas, observado o que estabelece o art. 84, VI, *b* ; (Redação dada pela Emenda Constitucional nº 32, de 2001)

XI - criação e extinção de Ministérios e órgãos da administração pública; (Redação dada pela Emenda Constitucional nº 32, de 2001)

XII - telecomunicações e radiodifusão;

XIII - matéria financeira, cambial e monetária, instituições financeiras e suas operações;

XIV - moeda, seus limites de emissão, e montante da dívida mobiliária federal.

XV - fixação do subsídio dos Ministros do Supremo Tribunal Federal, observado o que dispõem os arts. 39, § 4º; 150, II; 153, III; e 153, § 2º, I. (Redação dada pela Emenda Constitucional nº 41, 19.12.2003)

Art. 49. É da competência exclusiva do Congresso Nacional:

I - resolver definitivamente sobre tratados, acordos ou atos internacionais que acarretem encargos ou compromissos gravosos ao patrimônio nacional;

II - autorizar o Presidente da República a declarar guerra, a celebrar a paz, a permitir que forças estrangeiras transitem pelo território nacional ou nele permaneçam temporariamente, ressalvados os casos previstos em lei complementar;

III - autorizar o Presidente e o Vice-Presidente da República a se ausentarem do País, quando a ausência exceder a quinze dias;

IV - aprovar o estado de defesa e a intervenção federal, autorizar o estado de sítio, ou suspender qualquer uma dessas medidas;

V - sustar os atos normativos do Poder Executivo que exorbitem do poder regulamentar ou dos limites de delegação legislativa;

VI - mudar temporariamente sua sede;

VII - fixar idêntico subsídio para os Deputados Federais e os Senadores, observado o que dispõem os arts. 37, XI, 39, § 4º, 150, II, 153, III, e 153, § 2º, I; (Redação dada pela Emenda Constitucional nº 19, de 1998)

VIII - fixar os subsídios do Presidente e do Vice-Presidente da República e dos Ministros de Estado, observado o que dispõem os arts. 37, XI, 39, § 4º, 150, II, 153, III, e 153, § 2º, I; (Redação dada pela Emenda Constitucional nº 19, de 1998)

IX - julgar anualmente as contas prestadas pelo Presidente da República e apreciar os relatórios sobre a execução dos planos de governo;

X - fiscalizar e controlar, diretamente, ou por qualquer de suas Casas, os atos do Poder Executivo, incluídos os da administração indireta;

XI - zelar pela preservação de sua competência legislativa em face da atribuição normativa dos outros Poderes;

XII - apreciar os atos de concessão e renovação de concessão de emissoras de rádio e televisão;

XIII - escolher dois terços dos membros do Tribunal de Contas da União;

XIV - aprovar iniciativas do Poder Executivo referentes a atividades nucleares;

XV - autorizar referendo e convocar plebiscito;

XVI - autorizar, em terras indígenas, a exploração e o aproveitamento de recursos hídricos e a pesquisa e lavra de riquezas minerais;

XVII - aprovar, previamente, a alienação ou concessão de terras públicas com área superior a dois mil e quinhentos hectares.

Art. 50. A Câmara dos Deputados e o Senado Federal, ou qualquer de suas Comissões, poderão convocar Ministro de Estado ou quaisquer titulares de órgãos diretamente subordinados à Presidência da República para prestarem, pessoalmente, informações sobre assunto previamente determinado, importando crime de responsabilidade a ausência sem justificação adequada. (Redação dada pela Emenda Constitucional de Revisão nº 2, de 1994)

§ 1º Os Ministros de Estado poderão comparecer ao Senado Federal, à Câmara dos Deputados, ou a qualquer de suas Comissões, por sua iniciativa e mediante entendimentos com a Mesa respectiva, para expor assunto de relevância de seu Ministério.

§ 2º As Mesas da Câmara dos Deputados e do Senado Federal poderão encaminhar pedidos escritos de informações a Ministros de Estado ou a qualquer das pessoas referidas no caput deste artigo, importando em crime de responsabilidade a recusa, ou o não - atendimento, no prazo de trinta dias, bem como a prestação de informações falsas. (Redação dada pela Emenda Constitucional de Revisão nº 2, de 1994)

SEÇÃO III
DA CÂMARA DOS DEPUTADOS

Art. 51. Compete privativamente à Câmara dos Deputados:

I - autorizar, por dois terços de seus membros, a instauração de processo contra o Presidente e o Vice-Presidente da República e os Ministros de Estado;

II - proceder à tomada de contas do Presidente da República, quando não apresentadas ao Congresso Nacional dentro de sessenta dias após a abertura da sessão legislativa;

III - elaborar seu regimento interno;

IV - dispor sobre sua organização, funcionamento, polícia, criação, transformação ou extinção dos cargos, empregos e funções de seus serviços, e a iniciativa de lei para fixação da respectiva remuneração, observados os parâmetros estabelecidos na lei de diretrizes orçamentárias; (Redação dada pela Emenda Constitucional nº 19, de 1998)

V - eleger membros do Conselho da República, nos termos do art. 89, VII.

SEÇÃO IV
DO SENADO FEDERAL

Art. 52. Compete privativamente ao Senado Federal:

I - processar e julgar o Presidente e o Vice-Presidente da República nos crimes de responsabilidade, bem como os Ministros de Estado e os Comandantes da Marinha, do Exército e da Aeronáutica nos crimes da mesma natureza conexos com aqueles; (Redação dada pela Emenda Constitucional nº 23, de 02/09/99)

II processar e julgar os Ministros do Supremo Tribunal Federal, os membros do Conselho Nacional de Justiça e do Conselho Nacional do Ministério Público, o Procurador-Geral da República e o Advogado-Geral da União nos crimes de responsabilidade; (Redação dada pela Emenda Constitucional nº 45, de 2004)

III - aprovar previamente, por voto secreto, após argüição pública, a escolha de:

a) Magistrados, nos casos estabelecidos nesta Constituição;

b) Ministros do Tribunal de Contas da União indicados pelo Presidente da República;

c) Governador de Território;

d) Presidente e diretores do banco central;

e) Procurador-Geral da República;

f) titulares de outros cargos que a lei determinar;

IV - aprovar previamente, por voto secreto, após argüição em sessão secreta, a escolha dos chefes de missão diplomática de caráter permanente;

V - autorizar operações externas de natureza financeira, de interesse da União, dos Estados, do Distrito Federal, dos Territórios e dos Municípios;

VI - fixar, por proposta do Presidente da República, limites globais para o montante da dívida consolidada da União, dos Estados, do Distrito Federal e dos Municípios;

VII - dispor sobre limites globais e condições para as operações de crédito externo e interno da União, dos Estados, do Distrito Federal e dos Municípios, de suas autarquias e demais entidades controladas pelo Poder Público federal;

VIII - dispor sobre limites e condições para a concessão de garantia da União em operações de crédito externo e interno;

IX - estabelecer limites globais e condições para o montante da dívida mobiliária dos Estados, do Distrito Federal e dos Municípios;

X - suspender a execução, no todo ou em parte, de lei declarada inconstitucional por decisão definitiva do Supremo Tribunal Federal;

XI - aprovar, por maioria absoluta e por voto secreto, a exoneração, de ofício, do Procurador-Geral da República antes do término de seu mandato;

XII - elaborar seu regimento interno;

XIII - dispor sobre sua organização, funcionamento, polícia, criação, transformação ou extinção dos cargos, empregos e funções de seus serviços, e a iniciativa de lei para fixação da respectiva remuneração, observados os parâmetros estabelecidos na lei de diretrizes orçamentárias; (Redação dada pela Emenda Constitucional nº 19, de 1998)

XIV - eleger membros do Conselho da República, nos termos do art. 89, VII.

XV - avaliar periodicamente a funcionalidade do Sistema Tributário Nacional, em sua estrutura e seus componentes, e o desempenho das administrações tributárias da União, dos Estados e do Distrito Federal e dos Municípios. (Incluído pela Emenda Constitucional nº 42, de 19.12.2003)

Parágrafo único. Nos casos previstos nos incisos I e II, funcionará como Presidente o do Supremo Tribunal Federal, limitando-se a condenação, que somente será proferida por dois terços dos votos do Senado Federal, à perda do cargo, com inabilitação, por oito anos, para o exercício de função pública, sem prejuízo das demais sanções judiciais cabíveis.

SEÇÃO V
DOS DEPUTADOS E DOS SENADORES

Art. 53. Os Deputados e Senadores são invioláveis, civil e penalmente, por quaisquer de suas opiniões, palavras e votos. (Redação dada pela Emenda Constitucional nº 35, de 2001)

§ 1º Os Deputados e Senadores, desde a expedição do diploma, serão submetidos a julgamento perante o Supremo Tribunal Federal. (Redação dada pela Emenda Constitucional nº 35, de 2001)

§ 2º Desde a expedição do diploma, os membros do Congresso Nacional não poderão ser presos, salvo em flagrante de crime inafiançável. Nesse caso, os autos serão remetidos dentro de vinte e quatro horas à Casa respectiva, para que, pelo voto da maioria de seus membros, resolva sobre a prisão. (Redação dada pela Emenda Constitucional nº 35, de 2001)

§ 3º Recebida a denúncia contra o Senador ou Deputado, por crime ocorrido após a diplomação, o Supremo Tribunal Federal dará ciência à Casa respectiva, que, por iniciativa de partido político nela representado e pelo voto da maioria de seus membros, poderá, até a decisão final, sustar o andamento da ação. (Redação dada pela Emenda Constitucional nº 35, de 2001)

§ 4º O pedido de sustação será apreciado pela Casa respectiva no prazo improrrogável de quarenta e cinco dias do seu recebimento pela Mesa Diretora. (Redação dada pela Emenda Constitucional nº 35, de 2001)

§ 5º A sustação do processo suspende a prescrição, enquanto durar o mandato. (Redação dada pela Emenda Constitucional nº 35, de 2001)

§ 6º Os Deputados e Senadores não serão obrigados a testemunhar sobre informações recebidas ou prestadas em razão do exercício do mandato, nem sobre as pessoas que lhes confiaram ou deles receberam informações. (Redação dada pela Emenda Constitucional nº 35, de 2001)

§ 7º A incorporação às Forças Armadas de Deputados e Senadores, embora militares e ainda que em tempo de guerra, dependerá de prévia licença da Casa respectiva. (Redação dada pela Emenda Constitucional nº 35, de 2001)

§ 8º As imunidades de Deputados ou Senadores subsistirão durante o estado de sítio, só podendo ser suspensas mediante o voto de dois terços dos membros da Casa respectiva, nos casos de atos praticados fora do recinto do Congresso Nacional, que sejam incompatíveis com a execução da medida. (Incluído pela Emenda Constitucional nº 35, de 2001)

Art. 54. Os Deputados e Senadores não poderão:

I - desde a expedição do diploma:

a) firmar ou manter contrato com pessoa jurídica de direito público, autarquia, empresa pública, sociedade de economia mista ou empresa concessionária de serviço público, salvo quando o contrato obedecer a cláusulas uniformes;

b) aceitar ou exercer cargo, função ou emprego remunerado, inclusive os de que sejam demissíveis "ad nutum", nas entidades constantes da alínea anterior;

II - desde a posse:

a) ser proprietários, controladores ou diretores de empresa que goze de favor decorrente de contrato com pessoa jurídica de direito público, ou nela exercer função remunerada;

b) ocupar cargo ou função de que sejam demissíveis "ad nutum", nas entidades referidas no inciso I, "a";

c) patrocinar causa em que seja interessada qualquer das entidades a que se refere o inciso I, "a";

d) ser titulares de mais de um cargo ou mandato público eletivo.

Art. 55. Perderá o mandato o Deputado ou Senador:

I - que infringir qualquer das proibições estabelecidas no artigo anterior;

II - cujo procedimento for declarado incompatível com o decoro parlamentar;

III - que deixar de comparecer, em cada sessão legislativa, à terça parte das sessões ordinárias da Casa a que pertencer, salvo licença ou missão por esta autorizada;

IV - que perder ou tiver suspensos os direitos políticos;

V - quando o decretar a Justiça Eleitoral, nos casos previstos nesta Constituição;

VI - que sofrer condenação criminal em sentença transitada em julgado.

§ 1º - É incompatível com o decoro parlamentar, além dos casos definidos no regimento interno, o abuso das prerrogativas asseguradas a membro do Congresso Nacional ou a percepção de vantagens indevidas.

§ 2º Nos casos dos incisos I, II e VI, a perda do mandato será decidida pela Câmara dos Deputados ou pelo Senado Federal, por maioria absoluta, mediante provocação da respectiva Mesa ou de partido político representado no Congresso Nacional, assegurada ampla defesa. (Redação dada pela Emenda Constitucional nº 76, de 2013)

§ 3º Nos casos previstos nos incisos III a V, a perda será declarada pela Mesa da Casa respectiva, de ofício ou mediante provocação de qualquer de seus membros, ou de partido político representado no Congresso Nacional, assegurada ampla defesa.

§ 4º A renúncia de parlamentar submetido a processo que vise ou possa levar à perda do mandato, nos termos deste artigo, terá seus efeitos suspensos até as deliberações finais de que tratam os §§ 2º e 3º. (Incluído pela Emenda Constitucional de Revisão nº 6, de 1994)

Art. 56. Não perderá o mandato o Deputado ou Senador:

I - investido no cargo de Ministro de Estado, Governador de Território, Secretário de Estado, do Distrito Federal, de Território, de Prefeitura de Capital ou chefe de missão diplomática temporária;

II - licenciado pela respectiva Casa por motivo de doença, ou para tratar, sem remuneração, de interesse particular, desde que, neste caso, o afastamento não ultrapasse cento e vinte dias por sessão legislativa.

§ 1º O suplente será convocado nos casos de vaga, de investidura em funções previstas neste artigo ou de licença superior a cento e vinte dias.

§ 2º Ocorrendo vaga e não havendo suplente, far-se-á eleição para preenchê-la se faltarem mais de quinze meses para o término do mandato.

§ 3º Na hipótese do inciso I, o Deputado ou Senador poderá optar pela remuneração do mandato.

SEÇÃO VI
DAS REUNIÕES

Art. 57. O Congresso Nacional reunir-se-á, anualmente, na Capital Federal, de 2 de fevereiro a 17 de julho e de 1º de agosto a 22 de dezembro. (Redação dada pela Emenda Constitucional nº 50, de 2006)

§ 1º As reuniões marcadas para essas datas serão transferidas para o primeiro dia útil subseqüente, quando recaírem em sábados, domingos ou feriados.

§ 2º A sessão legislativa não será interrompida sem a aprovação do projeto de lei de diretrizes orçamentárias.

§ 3º Além de outros casos previstos nesta Constituição, a Câmara dos Deputados e o Senado Federal reunir-se-ão em sessão conjunta para:

I - inaugurar a sessão legislativa;

II - elaborar o regimento comum e regular a criação de serviços comuns às duas Casas;

III - receber o compromisso do Presidente e do Vice-Presidente da República;

IV - conhecer do veto e sobre ele deliberar.

§ 4º Cada uma das Casas reunir-se-á em sessões preparatórias, a partir de 1º de fevereiro, no primeiro ano da legislatura, para a posse de seus membros e eleição das respectivas Mesas, para mandato de 2 (dois) anos, vedada a recondução para o mesmo cargo na eleição imediatamente subseqüente. (Redação dada pela Emenda Constitucional nº 50, de 2006)

§ 5º A Mesa do Congresso Nacional será presidida pelo Presidente do Senado Federal, e os demais cargos serão exercidos, alternadamente, pelos ocupantes de cargos equivalentes na Câmara dos Deputados e no Senado Federal.

~~§ 6º A convocação extraordinária do Congresso Nacional far-se-á:~~

§ 6º A convocação extraordinária do Congresso Nacional far-se-á: (Redação dada pela Emenda Constitucional nº 50, de 2006)

I - pelo Presidente do Senado Federal, em caso de decretação de estado de defesa ou de intervenção federal, de pedido de autorização para a decretação de estado de sítio e para o compromisso e a posse do Presidente e do Vice-Presidente da República;

II - pelo Presidente da República, pelos Presidentes da Câmara dos Deputados e do Senado Federal ou a requerimento da maioria dos membros de ambas as Casas, em caso de urgência ou interesse público relevante, em todas as hipóteses deste inciso com a aprovação da maioria absoluta de cada uma das Casas do Congresso Nacional. (Redação dada pela Emenda Constitucional nº 50, de 2006)

§ 7º Na sessão legislativa extraordinária, o Congresso Nacional somente deliberará sobre a matéria para a qual foi convocado, ressalvada a hipótese do § 8º deste artigo, vedado o pagamento de parcela indenizatória, em razão da convocação. (Redação dada pela Emenda Constitucional nº 50, de 2006)

§ 8º Havendo medidas provisórias em vigor na data de convocação extraordinária do Congresso Nacional, serão elas automaticamente incluídas na pauta da convocação. (Incluído pela Emenda Constitucional nº 32, de 2001)

SEÇÃO VII
DAS COMISSÕES

Art. 58. O Congresso Nacional e suas Casas terão comissões permanentes e temporárias, constituídas na forma e com as atribuições previstas no respectivo regimento ou no ato de que resultar sua criação.

§ 1º Na constituição das Mesas e de cada Comissão, é assegurada, tanto quanto possível, a representação proporcional dos partidos ou dos blocos parlamentares que participam da respectiva Casa.

§ 2º Às comissões, em razão da matéria de sua competência, cabe:

I - discutir e votar projeto de lei que dispensar, na forma do regimento, a competência do Plenário, salvo se houver recurso de um décimo dos membros da Casa;

II - realizar audiências públicas com entidades da sociedade civil;

III - convocar Ministros de Estado para prestar informações sobre assuntos inerentes a suas atribuições;

IV - receber petições, reclamações, representações ou queixas de qualquer pessoa contra atos ou omissões das autoridades ou entidades públicas;

V - solicitar depoimento de qualquer autoridade ou cidadão;

VI - apreciar programas de obras, planos nacionais, regionais e setoriais de desenvolvimento e sobre eles emitir parecer.

§ 3º As comissões parlamentares de inquérito, que terão poderes de investigação próprios das autoridades judiciais, além de outros previstos nos regimentos das respectivas Casas, serão criadas pela Câmara dos Deputados e pelo Senado Federal, em conjunto ou separadamente, mediante requerimento de um terço de seus membros, para a apuração de fato determinado e por prazo certo, sendo suas conclusões, se for o caso, encaminhadas ao Ministério Público, para que promova a responsabilidade civil ou criminal dos infratores.

§ 4º Durante o recesso, haverá uma Comissão representativa do Congresso Nacional, eleita por suas Casas na última sessão ordinária do período legislativo, com atribuições definidas no regimento comum, cuja composição reproduzirá, quanto possível, a proporcionalidade da representação partidária.

SEÇÃO VIII
DO PROCESSO LEGISLATIVO
SUBSEÇÃO I
DISPOSIÇÃO GERAL

Art. 59. O processo legislativo compreende a elaboração de:

I - emendas à Constituição;

II - leis complementares;

III - leis ordinárias;

IV - leis delegadas;

V - medidas provisórias;

VI - decretos legislativos;

VII - resoluções.

Parágrafo único. Lei complementar disporá sobre a elaboração, redação, alteração e consolidação das leis.

SUBSEÇÃO II
DA EMENDA À CONSTITUIÇÃO

Art. 60. A Constituição poderá ser emendada mediante proposta:

I - de um terço, no mínimo, dos membros da Câmara dos Deputados ou do Senado Federal;

II - do Presidente da República;

III - de mais da metade das Assembléias Legislativas das unidades da Federação, manifestando-se, cada uma delas, pela maioria relativa de seus membros.

§ 1º A Constituição não poderá ser emendada na vigência de intervenção federal, de estado de defesa ou de estado de sítio.

§ 2º A proposta será discutida e votada em cada Casa do Congresso Nacional, em dois turnos, considerando-se aprovada se obtiver, em ambos, três quintos dos votos dos respectivos membros.

§ 3º A emenda à Constituição será promulgada pelas Mesas da Câmara dos Deputados e do Senado Federal, com o respectivo número de ordem.

§ 4º Não será objeto de deliberação a proposta de emenda tendente a abolir:

I - a forma federativa de Estado;

II - o voto direto, secreto, universal e periódico;

III - a separação dos Poderes;

IV - os direitos e garantias individuais.

§ 5º A matéria constante de proposta de emenda rejeitada ou havida por prejudicada não pode ser objeto de nova proposta na mesma sessão legislativa.

SUBSEÇÃO III
DAS LEIS

Art. 61. A iniciativa das leis complementares e ordinárias cabe a qualquer membro ou Comissão da Câmara dos Deputados, do Senado Federal ou do Congresso Nacional, ao Presidente da República, ao Supremo Tribunal Federal, aos Tribunais Superiores, ao Procurador-Geral da República e aos cidadãos, na forma e nos casos previstos nesta Constituição.

§ 1º São de iniciativa privativa do Presidente da República as leis que:

I - fixem ou modifiquem os efetivos das Forças Armadas;

II - disponham sobre:

a) criação de cargos, funções ou empregos públicos na administração direta e autárquica ou aumento de sua remuneração;

b) organização administrativa e judiciária, matéria tributária e orçamentária, serviços públicos e pessoal da administração dos Territórios;

~~c) servidores públicos da União e Territórios, seu regime jurídico, provimento de cargos, estabilidade e aposentadoria de civis, reforma e transferência de militares para a inatividade;~~

c) servidores públicos da União e Territórios, seu regime jurídico, provimento de cargos, estabilidade e aposentadoria; (Redação dada pela Emenda Constitucional nº 18, de 1998)

d) organização do Ministério Público e da Defensoria Pública da União, bem como normas gerais para a organização do Ministério Público e da Defensoria Pública dos Estados, do Distrito Federal e dos Territórios;

~~e) criação, estruturação e atribuições dos Ministérios e órgãos da administração pública;~~

e) criação e extinção de Ministérios e órgãos da administração pública, observado o disposto no art. 84, VI; (Redação dada pela Emenda Constitucional nº 32, de 2001)

f) militares das Forças Armadas, seu regime jurídico, provimento de cargos, promoções, estabilidade, remuneração, reforma e transferência para a reserva. (Incluída pela Emenda Constitucional nº 18, de 1998)

§ 2º A iniciativa popular pode ser exercida pela apresentação à Câmara dos Deputados de projeto de lei subscrito por, no mínimo, um por cento do eleitorado nacional, distribuído pelo menos por cinco Estados, com não menos de três décimos por cento dos eleitores de cada um deles.

Art. 62. Em caso de relevância e urgência, o Presidente da República poderá adotar medidas provisórias, com força de lei, devendo submetê-las de imediato ao Congresso Nacional. (Redação dada pela Emenda Constitucional nº 32, de 2001)

§ 1º É vedada a edição de medidas provisórias sobre matéria: (Incluído pela Emenda Constitucional nº 32, de 2001)

I - relativa a: (Incluído pela Emenda Constitucional nº 32, de 2001)

a) nacionalidade, cidadania, direitos políticos, partidos políticos e direito eleitoral; (Incluído pela Emenda Constitucional nº 32, de 2001)

b) direito penal, processual penal e processual civil; (Incluído pela Emenda Constitucional nº 32, de 2001)

c) organização do Poder Judiciário e do Ministério Público, a carreira e a garantia de seus membros; (Incluído pela Emenda Constitucional nº 32, de 2001)

d) planos plurianuais, diretrizes orçamentárias, orçamento e créditos adicionais e suplementares, ressalvado o previsto no art. 167, § 3º; (Incluído pela Emenda Constitucional nº 32, de 2001)

II - que vise a detenção ou seqüestro de bens, de poupança popular ou qualquer outro ativo financeiro; (Incluído pela Emenda Constitucional nº 32, de 2001)

III - reservada a lei complementar; (Incluído pela Emenda Constitucional nº 32, de 2001)

IV - já disciplinada em projeto de lei aprovado pelo Congresso Nacional e pendente de sanção ou veto do Presidente da República. (Incluído pela Emenda Constitucional nº 32, de 2001)

§ 2º Medida provisória que implique instituição ou majoração de impostos, exceto os previstos nos arts. 153, I, II, IV, V, e 154, II, só produzirá efeitos no exercício financeiro seguinte se houver sido convertida em lei até o último dia daquele em que foi editada. (Incluído pela Emenda Constitucional nº 32, de 2001)

§ 3º As medidas provisórias, ressalvado o disposto nos §§ 11 e 12 perderão eficácia, desde a edição, se não forem convertidas em lei no prazo de sessenta dias, prorrogável, nos termos do § 7º, uma vez por igual período, devendo o Congresso Nacional disciplinar, por decreto legislativo, as relações jurídicas delas decorrentes. (Incluído pela Emenda Constitucional nº 32, de 2001)

§ 4º O prazo a que se refere o § 3º contar-se-á da publicação da medida provisória, suspendendo-se durante os períodos de recesso do Congresso Nacional. (Incluído pela Emenda Constitucional nº 32, de 2001)

§ 5º A deliberação de cada uma das Casas do Congresso Nacional sobre o mérito das medidas provisórias dependerá de juízo prévio sobre o atendimento de seus pressupostos constitucionais. (Incluído pela Emenda Constitucional nº 32, de 2001)

§ 6º Se a medida provisória não for apreciada em até quarenta e cinco dias contados de sua publicação, entrará em regime de urgência, subseqüentemente, em cada uma das Casas do Congresso Nacional, ficando sobrestadas, até que se ultime a votação, todas as demais deliberações legislativas da Casa em que estiver tramitando. (Incluído pela Emenda Constitucional nº 32, de 2001)

§ 7º Prorrogar-se-á uma única vez por igual período a vigência de medida provisória que, no prazo de sessenta dias, contado de sua publicação, não tiver a sua votação encerrada nas duas Casas do Congresso Nacional. (Incluído pela Emenda Constitucional nº 32, de 2001)

§ 8º As medidas provisórias terão sua votação iniciada na Câmara dos Deputados. (Incluído pela Emenda Constitucional nº 32, de 2001)

§ 9º Caberá à comissão mista de Deputados e Senadores examinar as medidas provisórias e sobre elas emitir parecer, antes de serem apreciadas, em sessão separada, pelo plenário de cada uma das Casas do Congresso Nacional. (Incluído pela Emenda Constitucional nº 32, de 2001)

§ 10. É vedada a reedição, na mesma sessão legislativa, de medida provisória que tenha sido rejeitada ou que tenha perdido sua eficácia por decurso de prazo. (Incluído pela Emenda Constitucional nº 32, de 2001)

§ 11. Não editado o decreto legislativo a que se refere o § 3º até sessenta dias após a rejeição ou perda de eficácia de medida provisória, as relações jurídicas constituídas e decorrentes de atos praticados durante sua vigência conservar-se-ão por ela regidas. (Incluído pela Emenda Constitucional nº 32, de 2001)

§ 12. Aprovado projeto de lei de conversão alterando o texto original da medida provisória, esta manter-se-á integralmente em vigor até que seja sancionado ou vetado o projeto. (Incluído pela Emenda Constitucional nº 32, de 2001)

Art. 63. Não será admitido aumento da despesa prevista:

I - nos projetos de iniciativa exclusiva do Presidente da República, ressalvado o disposto no art. 166, § 3º e § 4º;

II - nos projetos sobre organização dos serviços administrativos da Câmara dos Deputados, do Senado Federal, dos Tribunais Federais e do Ministério Público.

Art. 64. A discussão e votação dos projetos de lei de iniciativa do Presidente da República, do Supremo Tribunal Federal e dos Tribunais Superiores terão início na Câmara dos Deputados.

§ 1º - O Presidente da República poderá solicitar urgência para apreciação de projetos de sua iniciativa.

§ 2º Se, no caso do § 1º, a Câmara dos Deputados e o Senado Federal não se manifestarem sobre a proposição, cada qual sucessivamente, em até quarenta e cinco dias, sobrestar-se-ão todas as demais deliberações legislativas da respectiva Casa, com exceção das que tenham prazo constitucional determinado, até que se ultime a votação. (Redação dada pela Emenda Constitucional nº 32, de 2001)

§ 3º A apreciação das emendas do Senado Federal pela Câmara dos Deputados far-se-á no prazo de dez dias, observado quanto ao mais o disposto no parágrafo anterior.

§ 4º Os prazos do § 2º não correm nos períodos de recesso do Congresso Nacional, nem se aplicam aos projetos de código.

Art. 65. O projeto de lei aprovado por uma Casa será revisto pela outra, em um só turno de discussão e votação, e enviado à sanção ou promulgação, se a Casa revisora o aprovar, ou arquivado, se o rejeitar.

Parágrafo único. Sendo o projeto emendado, voltará à Casa iniciadora.

Art. 66. A Casa na qual tenha sido concluída a votação enviará o projeto de lei ao Presidente da República, que, aquiescendo, o sancionará.

§ 1º Se o Presidente da República considerar o projeto, no todo ou em parte, inconstitucional ou contrário ao interesse público, vetá-lo-á total ou parcialmente, no prazo de quinze dias úteis, contados da data do recebimento, e comunicará, dentro de quarenta e oito horas, ao Presidente do Senado Federal os motivos do veto.

§ 2º O veto parcial somente abrangerá texto integral de artigo, de parágrafo, de inciso ou de alínea.

§ 3º Decorrido o prazo de quinze dias, o silêncio do Presidente da República importará sanção.

§ 4º O veto será apreciado em sessão conjunta, dentro de trinta dias a contar de seu recebimento, só podendo ser rejeitado pelo voto da maioria absoluta dos Deputados e Senadores. (Redação dada pela Emenda Constitucional nº 76, de 2013)

§ 5º Se o veto não for mantido, será o projeto enviado, para promulgação, ao Presidente da República.

§ 6º Esgotado sem deliberação o prazo estabelecido no § 4º, o veto será colocado na ordem do dia da sessão imediata, sobrestadas as demais proposições, até sua votação final. (Redação dada pela Emenda Constitucional nº 32, de 2001)

§ 7º Se a lei não for promulgada dentro de quarenta e oito horas pelo Presidente da República, nos casos dos § 3º e § 5º, o Presidente do Senado a promulgará, e, se este não o fizer em igual prazo, caberá ao Vice-Presidente do Senado fazê-lo.

Art. 67. A matéria constante de projeto de lei rejeitado somente poderá constituir objeto de novo projeto, na mesma sessão legislativa, mediante proposta da maioria absoluta dos membros de qualquer das Casas do Congresso Nacional.

Art. 68. As leis delegadas serão elaboradas pelo Presidente da República, que deverá solicitar a delegação ao Congresso Nacional.

§ 1º Não serão objeto de delegação os atos de competência exclusiva do Congresso Nacional, os de competência privativa da Câmara dos Deputados ou do Senado Federal, a matéria reservada à lei complementar, nem a legislação sobre:

I - organização do Poder Judiciário e do Ministério Público, a carreira e a garantia de seus membros;

II - nacionalidade, cidadania, direitos individuais, políticos e eleitorais;

III - planos plurianuais, diretrizes orçamentárias e orçamentos.

§ 2º A delegação ao Presidente da República terá a forma de resolução do Congresso Nacional, que especificará seu conteúdo e os termos de seu exercício.

§ 3º Se a resolução determinar a apreciação do projeto pelo Congresso Nacional, este a fará em votação única, vedada qualquer emenda.

Art. 69. As leis complementares serão aprovadas por maioria absoluta.

SEÇÃO IX
DA FISCALIZAÇÃO CONTÁBIL, FINANCEIRA E ORÇAMENTÁRIA

Art. 70. A fiscalização contábil, financeira, orçamentária, operacional e patrimonial da União e das entidades da administração direta e indireta, quanto à legalidade, legitimidade, economicidade, aplicação das subvenções e renúncia de receitas, será exercida pelo Congresso Nacional, mediante controle externo, e pelo sistema de controle interno de cada Poder.

Parágrafo único. Prestará contas qualquer pessoa física ou jurídica, pública ou privada, que utilize, arrecade, guarde, gerencie ou administre dinheiros, bens e valores públicos ou pelos quais a União responda, ou que, em nome desta, assuma obrigações de natureza pecuniária. (Redação dada pela Emenda Constitucional nº 19, de 1998)

Art. 71. O controle externo, a cargo do Congresso Nacional, será exercido com o auxílio do Tribunal de Contas da União, ao qual compete:

I - apreciar as contas prestadas anualmente pelo Presidente da República, mediante parecer prévio que deverá ser elaborado em sessenta dias a contar de seu recebimento;

II - julgar as contas dos administradores e demais responsáveis por dinheiros, bens e valores públicos da administração direta e indireta, incluídas as fundações e sociedades instituídas e mantidas pelo Poder Público federal, e as contas daqueles que derem causa a perda, extravio ou outra irregularidade de que resulte prejuízo ao erário público;

III - apreciar, para fins de registro, a legalidade dos atos de admissão de pessoal, a qualquer título, na administração direta e indireta, incluídas as fundações instituídas e mantidas pelo Poder Público, excetuadas as nomeações para cargo de provimento em comissão, bem como a das concessões de aposentadorias, reformas e pensões, ressalvadas as melhorias posteriores que não alterem o fundamento legal do ato concessório;

IV - realizar, por iniciativa própria, da Câmara dos Deputados, do Senado Federal, de Comissão técnica ou de inquérito, inspeções e auditorias de natureza contábil, financeira, orçamentária, operacional e patrimonial, nas unidades administrativas dos Poderes Legislativo, Executivo e Judiciário, e demais entidades referidas no inciso II;

V - fiscalizar as contas nacionais das empresas supranacionais de cujo capital social a União participe, de forma direta ou indireta, nos termos do tratado constitutivo;

VI - fiscalizar a aplicação de quaisquer recursos repassados pela União mediante convênio, acordo, ajuste ou outros instrumentos congêneres, a Estado, ao Distrito Federal ou a Município;

VII - prestar as informações solicitadas pelo Congresso Nacional, por qualquer de suas Casas, ou por qualquer das respectivas Comissões, sobre a fiscalização contábil, financeira, orçamentária, operacional e patrimonial e sobre resultados de auditorias e inspeções realizadas;

VIII - aplicar aos responsáveis, em caso de ilegalidade de despesa ou irregularidade de contas, as sanções previstas em lei, que estabelecerá, entre outras cominações, multa proporcional ao dano causado ao erário;

IX - assinar prazo para que o órgão ou entidade adote as providências necessárias ao exato cumprimento da lei, se verificada ilegalidade;

X - sustar, se não atendido, a execução do ato impugnado, comunicando a decisão à Câmara dos Deputados e ao Senado Federal;

XI - representar ao Poder competente sobre irregularidades ou abusos apurados.

§ 1º No caso de contrato, o ato de sustação será adotado diretamente pelo Congresso Nacional, que solicitará, de imediato, ao Poder Executivo as medidas cabíveis.

§ 2º Se o Congresso Nacional ou o Poder Executivo, no prazo de noventa dias, não efetivar as medidas previstas no parágrafo anterior, o Tribunal decidirá a respeito.

§ 3º As decisões do Tribunal de que resulte imputação de débito ou multa terão eficácia de título executivo.

§ 4º O Tribunal encaminhará ao Congresso Nacional, trimestral e anualmente, relatório de suas atividades.

Art. 72. A Comissão mista permanente a que se refere o art. 166, §1º, diante de indícios de despesas não autorizadas, ainda que sob a forma de investimentos não programados ou de subsídios não aprovados, poderá solicitar à autoridade governamental responsável que, no prazo de cinco dias, preste os esclarecimentos necessários.

§ 1º Não prestados os esclarecimentos, ou considerados estes insuficientes, a Comissão solicitará ao Tribunal pronunciamento conclusivo sobre a matéria, no prazo de trinta dias.

§ 2º Entendendo o Tribunal irregular a despesa, a Comissão, se julgar que o gasto possa causar dano irreparável ou grave lesão à economia pública, proporá ao Congresso Nacional sua sustação.

Art. 73. O Tribunal de Contas da União, integrado por nove Ministros, tem sede no Distrito Federal, quadro próprio de pessoal e jurisdição em todo o território nacional, exercendo, no que couber, as atribuições previstas no art. 96.

§ 1º Os Ministros do Tribunal de Contas da União serão nomeados dentre brasileiros que satisfaçam os seguintes requisitos:

I - mais de trinta e cinco e menos de sessenta e cinco anos de idade;

II - idoneidade moral e reputação ilibada;

III - notórios conhecimentos jurídicos, contábeis, econômicos e financeiros ou de administração pública;

IV - mais de dez anos de exercício de função ou de efetiva atividade profissional que exija os conhecimentos mencionados no inciso anterior.

§ 2º Os Ministros do Tribunal de Contas da União serão escolhidos:

I - um terço pelo Presidente da República, com aprovação do Senado Federal, sendo dois alternadamente dentre auditores e membros do Ministério Público junto ao Tribunal, indicados em lista tríplice pelo Tribunal, segundo os critérios de antigüidade e merecimento;

II - dois terços pelo Congresso Nacional.

§ 3° Os Ministros do Tribunal de Contas da União terão as mesmas garantias, prerrogativas, impedimentos, vencimentos e vantagens dos Ministros do Superior Tribunal de Justiça, aplicando-se-lhes, quanto à aposentadoria e pensão, as normas constantes do art. 40. (Redação dada pela Emenda Constitucional nº 20, de 1998)

§ 4º O auditor, quando em substituição a Ministro, terá as mesmas garantias e impedimentos do titular e, quando no exercício das demais atribuições da judicatura, as de juiz de Tribunal Regional Federal.

Art. 74. Os Poderes Legislativo, Executivo e Judiciário manterão, de forma integrada, sistema de controle interno com a finalidade de:

I - avaliar o cumprimento das metas previstas no plano plurianual, a execução dos programas de governo e dos orçamentos da União;

II - comprovar a legalidade e avaliar os resultados, quanto à eficácia e eficiência, da gestão orçamentária, financeira e patrimonial nos órgãos e entidades da administração federal, bem como da aplicação de recursos públicos por entidades de direito privado;

III - exercer o controle das operações de crédito, avais e garantias, bem como dos direitos e haveres da União;

IV - apoiar o controle externo no exercício de sua missão institucional.

§ 1º Os responsáveis pelo controle interno, ao tomarem conhecimento de qualquer irregularidade ou ilegalidade, dela darão ciência ao Tribunal de Contas da União, sob pena de responsabilidade solidária.

§ 2º Qualquer cidadão, partido político, associação ou sindicato é parte legítima para, na forma da lei, denunciar irregularidades ou ilegalidades perante o Tribunal de Contas da União.

Art. 75. As normas estabelecidas nesta seção aplicam-se, no que couber, à organização, composição e fiscalização dos Tribunais de Contas dos Estados e do Distrito Federal, bem como dos Tribunais e Conselhos de Contas dos Municípios.

Parágrafo único. As Constituições estaduais disporão sobre os Tribunais de Contas respectivos, que serão integrados por sete Conselheiros.

CAPÍTULO II
DO PODER EXECUTIVO
SEÇÃO I
DO PRESIDENTE E DO VICE-PRESIDENTE DA REPÚBLICA

Art. 76. O Poder Executivo é exercido pelo Presidente da República, auxiliado pelos Ministros de Estado.

Art. 77. A eleição do Presidente e do Vice-Presidente da República realizar-se-á, simultaneamente, no primeiro domingo de outubro, em primeiro turno, e no último domingo de outubro, em segundo turno, se houver, do ano anterior ao do término do mandato presidencial vigente. (Redação dada pela Emenda Constitucional nº 16, de 1997)

§ 1º A eleição do Presidente da República importará a do Vice-Presidente com ele registrado.

§ 2º Será considerado eleito Presidente o candidato que, registrado por partido político, obtiver a maioria absoluta de votos, não computados os em branco e os nulos.

§ 3º Se nenhum candidato alcançar maioria absoluta na primeira votação, far-se-á nova eleição em até vinte dias após a proclamação do resultado, concorrendo os dois candidatos mais votados e considerando-se eleito aquele que obtiver a maioria dos votos válidos.

§ 4º Se, antes de realizado o segundo turno, ocorrer morte, desistência ou impedimento legal de candidato, convocar-se-á, dentre os remanescentes, o de maior votação.

§ 5º Se, na hipótese dos parágrafos anteriores, remanescer, em segundo lugar, mais de um candidato com a mesma votação, qualificar-se-á o mais idoso.

Art. 78. O Presidente e o Vice-Presidente da República tomarão posse em sessão do Congresso Nacional, prestando o compromisso de manter, defender e cumprir a Constituição, observar as leis, promover o bem geral do povo brasileiro, sustentar a união, a integridade e a independência do Brasil.

Parágrafo único. Se, decorridos dez dias da data fixada para a posse, o Presidente ou o Vice-Presidente, salvo motivo de força maior, não tiver assumido o cargo, este será declarado vago.

Art. 79. Substituirá o Presidente, no caso de impedimento, e suceder-lhe-á, no de vaga, o Vice-Presidente.

Parágrafo único. O Vice-Presidente da República, além de outras atribuições que lhe forem conferidas por lei complementar, auxiliará o Presidente, sempre que por ele convocado para missões especiais.

Art. 80. Em caso de impedimento do Presidente e do Vice-Presidente, ou vacância dos respectivos cargos, serão sucessivamente chamados ao exercício da Presidência o Presidente da Câmara dos Deputados, o do Senado Federal e o do Supremo Tribunal Federal.

Art. 81. Vagando os cargos de Presidente e Vice-Presidente da República, far-se-á eleição noventa dias depois de aberta a última vaga.

§ 1º Ocorrendo a vacância nos últimos dois anos do período presidencial, a eleição para ambos os cargos será feita trinta dias depois da última vaga, pelo Congresso Nacional, na forma da lei.

§ 2º Em qualquer dos casos, os eleitos deverão completar o período de seus antecessores.

Art. 82. O mandato do Presidente da República é de quatro anos e terá início em primeiro de janeiro do ano seguinte ao da sua eleição. (Redação dada pela Emenda Constitucional nº 16, de 1997)

Art. 83. O Presidente e o Vice-Presidente da República não poderão, sem licença do Congresso Nacional, ausentar-se do País por período superior a quinze dias, sob pena de perda do cargo.

SEÇÃO II
DAS ATRIBUIÇÕES DO PRESIDENTE DA REPÚBLICA

Art. 84. Compete privativamente ao Presidente da República:

I - nomear e exonerar os Ministros de Estado;

II - exercer, com o auxílio dos Ministros de Estado, a direção superior da administração federal;

III - iniciar o processo legislativo, na forma e nos casos previstos nesta Constituição;

IV - sancionar, promulgar e fazer publicar as leis, bem como expedir decretos e regulamentos para sua fiel execução;

V - vetar projetos de lei, total ou parcialmente;

VI - dispor, mediante decreto, sobre: (Redação dada pela Emenda Constitucional nº 32, de 2001)

a) organização e funcionamento da administração federal, quando não implicar aumento de despesa nem criação ou extinção de órgãos públicos; (Incluída pela Emenda Constitucional nº 32, de 2001)

b) extinção de funções ou cargos públicos, quando vagos; (Incluída pela Emenda Constitucional nº 32, de 2001)

VII - manter relações com Estados estrangeiros e acreditar seus representantes diplomáticos;

VIII - celebrar tratados, convenções e atos internacionais, sujeitos a referendo do Congresso Nacional;

IX - decretar o estado de defesa e o estado de sítio;

X - decretar e executar a intervenção federal;

XI - remeter mensagem e plano de governo ao Congresso Nacional por ocasião da abertura da sessão legislativa, expondo a situação do País e solicitando as providências que julgar necessárias;

XII - conceder indulto e comutar penas, com audiência, se necessário, dos órgãos instituídos em lei;

XIII - exercer o comando supremo das Forças Armadas, nomear os Comandantes da Marinha, do Exército e da Aeronáutica, promover seus oficiais-generais e nomeá-los para os cargos que lhes são privativos; (Redação dada pela Emenda Constitucional nº 23, de 02/09/99)

XIV - nomear, após aprovação pelo Senado Federal, os Ministros do Supremo Tribunal Federal e dos Tribunais Superiores, os Governadores de Territórios, o Procurador-Geral da

República, o presidente e os diretores do banco central e outros servidores, quando determinado em lei;

XV - nomear, observado o disposto no art. 73, os Ministros do Tribunal de Contas da União;

XVI - nomear os magistrados, nos casos previstos nesta Constituição, e o Advogado-Geral da União;

XVII - nomear membros do Conselho da República, nos termos do art. 89, VII;

XVIII - convocar e presidir o Conselho da República e o Conselho de Defesa Nacional;

XIX - declarar guerra, no caso de agressão estrangeira, autorizado pelo Congresso Nacional ou referendado por ele, quando ocorrida no intervalo das sessões legislativas, e, nas mesmas condições, decretar, total ou parcialmente, a mobilização nacional;

XX - celebrar a paz, autorizado ou com o referendo do Congresso Nacional;

XXI - conferir condecorações e distinções honoríficas;

XXII - permitir, nos casos previstos em lei complementar, que forças estrangeiras transitem pelo território nacional ou nele permaneçam temporariamente;

XXIII - enviar ao Congresso Nacional o plano plurianual, o projeto de lei de diretrizes orçamentárias e as propostas de orçamento previstos nesta Constituição;

XXIV - prestar, anualmente, ao Congresso Nacional, dentro de sessenta dias após a abertura da sessão legislativa, as contas referentes ao exercício anterior;

XXV - prover e extinguir os cargos públicos federais, na forma da lei;

XXVI - editar medidas provisórias com força de lei, nos termos do art. 62;

XXVII - exercer outras atribuições previstas nesta Constituição.

Parágrafo único. O Presidente da República poderá delegar as atribuições mencionadas nos incisos VI, XII e XXV, primeira parte, aos Ministros de Estado, ao Procurador-Geral da República ou ao Advogado-Geral da União, que observarão os limites traçados nas respectivas delegações.

SEÇÃO III
DA RESPONSABILIDADE DO PRESIDENTE DA REPÚBLICA

Art. 85. São crimes de responsabilidade os atos do Presidente da República que atentem contra a Constituição Federal e, especialmente, contra:

I - a existência da União;

II - o livre exercício do Poder Legislativo, do Poder Judiciário, do Ministério Público e dos Poderes constitucionais das unidades da Federação;

III - o exercício dos direitos políticos, individuais e sociais;

IV - a segurança interna do País;

V - a probidade na administração;

VI - a lei orçamentária;

VII - o cumprimento das leis e das decisões judiciais.

Parágrafo único. Esses crimes serão definidos em lei especial, que estabelecerá as normas de processo e julgamento.

Art. 86. Admitida a acusação contra o Presidente da República, por dois terços da Câmara dos Deputados, será ele submetido a julgamento perante o Supremo Tribunal Federal, nas infrações penais comuns, ou perante o Senado Federal, nos crimes de responsabilidade.

§ 1º O Presidente ficará suspenso de suas funções:

I - nas infrações penais comuns, se recebida a denúncia ou queixa-crime pelo Supremo Tribunal Federal;

II - nos crimes de responsabilidade, após a instauração do processo pelo Senado Federal.

§ 2º Se, decorrido o prazo de cento e oitenta dias, o julgamento não estiver concluído, cessará o afastamento do Presidente, sem prejuízo do regular prosseguimento do processo.

§ 3º Enquanto não sobrevier sentença condenatória, nas infrações comuns, o Presidente da República não estará sujeito a prisão.

§ 4º O Presidente da República, na vigência de seu mandato, não pode ser responsabilizado por atos estranhos ao exercício de suas funções.

SEÇÃO IV
DOS MINISTROS DE ESTADO

Art. 87. Os Ministros de Estado serão escolhidos dentre brasileiros maiores de vinte e um anos e no exercício dos direitos políticos.

Parágrafo único. Compete ao Ministro de Estado, além de outras atribuições estabelecidas nesta Constituição e na lei:

I - exercer a orientação, coordenação e supervisão dos órgãos e entidades da administração federal na área de sua competência e referendar os atos e decretos assinados pelo Presidente da República;

II - expedir instruções para a execução das leis, decretos e regulamentos;

III - apresentar ao Presidente da República relatório anual de sua gestão no Ministério;

IV - praticar os atos pertinentes às atribuições que lhe forem outorgadas ou delegadas pelo Presidente da República.

Art. 88. A lei disporá sobre a criação e extinção de Ministérios e órgãos da administração pública. (Redação dada pela Emenda Constitucional nº 32, de 2001)

SEÇÃO V
DO CONSELHO DA REPÚBLICA E DO CONSELHO DE DEFESA NACIONAL
SUBSEÇÃO I
DO CONSELHO DA REPÚBLICA

Art. 89. O Conselho da República é órgão superior de consulta do Presidente da República, e dele participam:

I - o Vice-Presidente da República;

II - o Presidente da Câmara dos Deputados;

III - o Presidente do Senado Federal;

IV - os líderes da maioria e da minoria na Câmara dos Deputados;

V - os líderes da maioria e da minoria no Senado Federal;

VI - o Ministro da Justiça;

VII - seis cidadãos brasileiros natos, com mais de trinta e cinco anos de idade, sendo dois nomeados pelo Presidente da República, dois eleitos pelo Senado Federal e dois eleitos pela Câmara dos Deputados, todos com mandato de três anos, vedada a recondução.

Art. 90. Compete ao Conselho da República pronunciar-se sobre:

I - intervenção federal, estado de defesa e estado de sítio;

II - as questões relevantes para a estabilidade das instituições democráticas.

§ 1º O Presidente da República poderá convocar Ministro de Estado para participar da reunião do Conselho, quando constar da pauta questão relacionada com o respectivo Ministério.

§ 2º A lei regulará a organização e o funcionamento do Conselho da República. (Vide Lei nº 8.041, de 1990)

SUBSEÇÃO II
DO CONSELHO DE DEFESA NACIONAL

Art. 91. O Conselho de Defesa Nacional é órgão de consulta do Presidente da República nos assuntos relacionados com a soberania nacional e a defesa do Estado democrático, e dele participam como membros natos:

I - o Vice-Presidente da República;

II - o Presidente da Câmara dos Deputados;

III - o Presidente do Senado Federal;

IV - o Ministro da Justiça;

V - o Ministro de Estado da Defesa; (Redação dada pela Emenda Constitucional nº 23, de 1999)

VI - o Ministro das Relações Exteriores;

VII - o Ministro do Planejamento.

VIII - os Comandantes da Marinha, do Exército e da Aeronáutica. (Incluído pela Emenda Constitucional nº 23, de 1999)

§ 1º Compete ao Conselho de Defesa Nacional:

I - opinar nas hipóteses de declaração de guerra e de celebração da paz, nos termos desta Constituição;

II - opinar sobre a decretação do estado de defesa, do estado de sítio e da intervenção federal;

III - propor os critérios e condições de utilização de áreas indispensáveis à segurança do território nacional e opinar sobre seu efetivo uso, especialmente na faixa de fronteira e nas relacionadas com a preservação e a exploração dos recursos naturais de qualquer tipo;

IV - estudar, propor e acompanhar o desenvolvimento de iniciativas necessárias a garantir a independência nacional e a defesa do Estado democrático.

§ 2º A lei regulará a organização e o funcionamento do Conselho de Defesa Nacional. (Vide Lei nº 8.183, de 1991)

CAPÍTULO III
DO PODER JUDICIÁRIO

SEÇÃO I
DISPOSIÇÕES GERAIS

Art. 92. São órgãos do Poder Judiciário:

I - o Supremo Tribunal Federal;

I-A o Conselho Nacional de Justiça; (Incluído pela Emenda Constitucional nº 45, de 2004)

II - o Superior Tribunal de Justiça;

II-A - o Tribunal Superior do Trabalho; (Incluído pela Emenda Constitucional nº 92, de 2016)

III - os Tribunais Regionais Federais e Juízes Federais;

IV - os Tribunais e Juízes do Trabalho;

V - os Tribunais e Juízes Eleitorais;

VI - os Tribunais e Juízes Militares;

VII - os Tribunais e Juízes dos Estados e do Distrito Federal e Territórios.

§ 1º O Supremo Tribunal Federal, o Conselho Nacional de Justiça e os Tribunais Superiores têm sede na Capital Federal. (Incluído pela Emenda Constitucional nº 45, de 2004) (Vide ADIN 3392)

§ 2º O Supremo Tribunal Federal e os Tribunais Superiores têm jurisdição em todo o território nacional. (Incluído pela Emenda Constitucional nº 45, de 2004)

Art. 93. Lei complementar, de iniciativa do Supremo Tribunal Federal, disporá sobre o Estatuto da Magistratura, observados os seguintes princípios:

I - ingresso na carreira, cujo cargo inicial será o de juiz substituto, mediante concurso público de provas e títulos, com a participação da Ordem dos Advogados do Brasil em todas as fases, exigindo-se do bacharel em direito, no mínimo, três anos de atividade jurídica e obedecendo-se, nas nomeações, à ordem de classificação; (Redação dada pela Emenda Constitucional nº 45, de 2004)

II - promoção de entrância para entrância, alternadamente, por antigüidade e merecimento, atendidas as seguintes normas:

a) é obrigatória a promoção do juiz que figure por três vezes consecutivas ou cinco alternadas em lista de merecimento;

b) a promoção por merecimento pressupõe dois anos de exercício na respectiva entrância e integrar o juiz a primeira quinta parte da lista de antigüidade desta, salvo se não houver com tais requisitos quem aceite o lugar vago;

c) aferição do merecimento conforme o desempenho e pelos critérios objetivos de produtividade e presteza no exercício da jurisdição e pela freqüência e aproveitamento em cursos oficiais ou reconhecidos de aperfeiçoamento; (Redação dada pela Emenda Constitucional nº 45, de 2004)

d) na apuração de antigüidade, o tribunal somente poderá recusar o juiz mais antigo pelo voto fundamentado de dois terços de seus membros, conforme procedimento próprio, e assegurada ampla defesa, repetindo-se a votação até fixar-se a indicação; (Redação dada pela Emenda Constitucional nº 45, de 2004)

e) não será promovido o juiz que, injustificadamente, retiver autos em seu poder além do prazo legal, não podendo devolvê-los ao cartório sem o devido despacho ou decisão; (Incluída pela Emenda Constitucional nº 45, de 2004)

III o acesso aos tribunais de segundo grau far-se-á por antigüidade e merecimento, alternadamente, apurados na última ou única entrância; (Redação dada pela Emenda Constitucional nº 45, de 2004) (Vide ADIN 3392)

IV previsão de cursos oficiais de preparação, aperfeiçoamento e promoção de magistrados, constituindo etapa obrigatória do processo de vitaliciamento a participação em curso oficial ou reconhecido por escola nacional de formação e aperfeiçoamento de magistrados; (Redação dada pela Emenda Constitucional nº 45, de 2004)

V - o subsídio dos Ministros dos Tribunais Superiores corresponderá a noventa e cinco por cento do subsídio mensal fixado para os Ministros do Supremo Tribunal Federal e os subsídios dos demais magistrados serão fixados em lei e escalonados, em nível federal e estadual, conforme as respectivas categorias da estrutura judiciária nacional, não podendo a diferença entre uma e outra ser superior a dez por cento ou inferior a cinco por cento, nem exceder a noventa e cinco por cento do subsídio mensal dos Ministros dos Tribunais Superiores, obedecido, em qualquer caso, o disposto nos arts. 37, XI, e 39, § 4º; (Redação dada pela Emenda Constitucional nº 19, de 1998)

VI - a aposentadoria dos magistrados e a pensão de seus dependentes observarão o disposto no art. 40; (Redação dada pela Emenda Constitucional nº 20, de 1998)

VII o juiz titular residirá na respectiva comarca, salvo autorização do tribunal; (Redação dada pela Emenda Constitucional nº 45, de 2004)

VIII - o ato de remoção, disponibilidade e aposentadoria do magistrado, por interesse público, fundar-se-á em decisão por voto da maioria absoluta do respectivo tribunal ou do Conselho Nacional de Justiça, assegurada ampla defesa; (Redação dada pela Emenda Constitucional nº 45, de 2004)

VIII-A a remoção a pedido ou a permuta de magistrados de comarca de igual entrância atenderá, no que couber, ao disposto nas alíneas a , b , c e e do inciso II; (Incluído pela Emenda Constitucional nº 45, de 2004)

IX todos os julgamentos dos órgãos do Poder Judiciário serão públicos, e fundamentadas todas as decisões, sob pena de nulidade, podendo a lei limitar a presença, em determinados atos, às próprias partes e a seus advogados, ou somente a estes, em casos nos quais a preservação do direito à intimidade do interessado no sigilo não prejudique o interesse público à informação; (Redação dada pela Emenda Constitucional nº 45, de 2004)

X as decisões administrativas dos tribunais serão motivadas e em sessão pública, sendo as disciplinares tomadas pelo voto da maioria absoluta de seus membros; (Redação dada pela Emenda Constitucional nº 45, de 2004)

XI nos tribunais com número superior a vinte e cinco julgadores, poderá ser constituído órgão especial, com o mínimo de onze e o máximo de vinte e cinco membros, para o exercício das atribuições administrativas e jurisdicionais delegadas da competência do tribunal pleno, provendo-se metade das vagas por antigüidade e a outra metade por eleição pelo tribunal pleno; (Redação dada pela Emenda Constitucional nº 45, de 2004)

XII a atividade jurisdicional será ininterrupta, sendo vedado férias coletivas nos juízos e tribunais de segundo grau, funcionando, nos dias em que não houver expediente forense normal, juízes em plantão permanente; (Incluído pela Emenda Constitucional nº 45, de 2004)

XIII o número de juízes na unidade jurisdicional será proporcional à efetiva demanda judicial e à respectiva população; (Incluído pela Emenda Constitucional nº 45, de 2004)

XIV os servidores receberão delegação para a prática de atos de administração e atos de mero expediente sem caráter decisório; (Incluído pela Emenda Constitucional nº 45, de 2004)

XV a distribuição de processos será imediata, em todos os graus de jurisdição. (Incluído pela Emenda Constitucional nº 45, de 2004)

Art. 94. Um quinto dos lugares dos Tribunais Regionais Federais, dos Tribunais dos Estados, e do Distrito Federal e Territórios será composto de membros, do Ministério Público, com mais de dez anos de carreira, e de advogados de notório saber jurídico e de reputação ilibada, com mais de dez anos de efetiva atividade profissional, indicados em lista sêxtupla pelos órgãos de representação das respectivas classes.

Parágrafo único. Recebidas as indicações, o tribunal formará lista tríplice, enviando-a ao Poder Executivo, que, nos vinte dias subseqüentes, escolherá um de seus integrantes para nomeação.

Art. 95. Os juízes gozam das seguintes garantias:

I - vitaliciedade, que, no primeiro grau, só será adquirida após dois anos de exercício, dependendo a perda do cargo, nesse período, de deliberação do tribunal a que o juiz estiver vinculado, e, nos demais casos, de sentença judicial transitada em julgado;

II - inamovibilidade, salvo por motivo de interesse público, na forma do art. 93, VIII;

III - irredutibilidade de subsídio, ressalvado o disposto nos arts. 37, X e XI, 39, § 4º, 150, II, 153, III, e 153, § 2º, I. (Redação dada pela Emenda Constitucional nº 19, de 1998)

Parágrafo único. Aos juízes é vedado:

I - exercer, ainda que em disponibilidade, outro cargo ou função, salvo uma de magistério;

II - receber, a qualquer título ou pretexto, custas ou participação em processo;

III - dedicar-se à atividade político-partidária.

IV - receber, a qualquer título ou pretexto, auxílios ou contribuições de pessoas físicas, entidades públicas ou privadas, ressalvadas as exceções previstas em lei; (Incluído pela Emenda Constitucional nº 45, de 2004)

V - exercer a advocacia no juízo ou tribunal do qual se afastou, antes de decorridos três anos do afastamento do cargo por aposentadoria ou exoneração. (Incluído pela Emenda Constitucional nº 45, de 2004)

Art. 96. Compete privativamente:

I - aos tribunais:

a) eleger seus órgãos diretivos e elaborar seus regimentos internos, com observância das normas de processo e das garantias processuais das partes, dispondo sobre a competência e o funcionamento dos respectivos órgãos jurisdicionais e administrativos;

b) organizar suas secretarias e serviços auxiliares e os dos juízos que lhes forem vinculados, velando pelo exercício da atividade correicional respectiva;

c) prover, na forma prevista nesta Constituição, os cargos de juiz de carreira da respectiva jurisdição;

d) propor a criação de novas varas judiciárias;

e) prover, por concurso público de provas, ou de provas e títulos, obedecido o disposto no art. 169, parágrafo único, os cargos necessários à administração da Justiça, exceto os de confiança assim definidos em lei;

f) conceder licença, férias e outros afastamentos a seus membros e aos juízes e servidores que lhes forem imediatamente vinculados;

II - ao Supremo Tribunal Federal, aos Tribunais Superiores e aos Tribunais de Justiça propor ao Poder Legislativo respectivo, observado o disposto no art. 169:

a) a alteração do número de membros dos tribunais inferiores;

b) a criação e a extinção de cargos e a remuneração dos seus serviços auxiliares e dos juízos que lhes forem vinculados, bem como a fixação do subsídio de seus membros e dos juízes, inclusive dos tribunais inferiores, onde houver; (Redação dada pela Emenda Constitucional nº 41, 19.12.2003)

c) a criação ou extinção dos tribunais inferiores;

d) a alteração da organização e da divisão judiciárias;

III - aos Tribunais de Justiça julgar os juízes estaduais e do Distrito Federal e Territórios, bem como os membros do Ministério Público, nos crimes comuns e de responsabilidade, ressalvada a competência da Justiça Eleitoral.

Art. 97. Somente pelo voto da maioria absoluta de seus membros ou dos membros do respectivo órgão especial poderão os tribunais declarar a inconstitucionalidade de lei ou ato normativo do Poder Público. (Vide Lei nº 13.105, de 2015) (Vigência)

Art. 98. A União, no Distrito Federal e nos Territórios, e os Estados criarão:

I - juizados especiais, providos por juízes togados, ou togados e leigos, competentes para a conciliação, o julgamento e a execução de causas cíveis de menor complexidade e infrações penais de menor potencial ofensivo, mediante os procedimentos oral e sumaríssimo , permitidos, nas hipóteses previstas em lei, a transação e o julgamento de recursos por turmas de juízes de primeiro grau;

II - justiça de paz, remunerada, composta de cidadãos eleitos pelo voto direto, universal e secreto, com mandato de quatro anos e competência para, na forma da lei, celebrar casamentos, verificar, de ofício ou em face de impugnação apresentada, o processo de habilitação e exercer atribuições conciliatórias, sem caráter jurisdicional, além de outras previstas na legislação.

§ 1º Lei federal disporá sobre a criação de juizados especiais no âmbito da Justiça Federal. (Renumerado pela Emenda Constitucional nº 45, de 2004) (Vide ADIN 3392)

§ 2º As custas e emolumentos serão destinados exclusivamente ao custeio dos serviços afetos às atividades específicas da Justiça. (Incluído pela Emenda Constitucional nº 45, de 2004)

Art. 99. Ao Poder Judiciário é assegurada autonomia administrativa e financeira.

§ 1º Os tribunais elaborarão suas propostas orçamentárias dentro dos limites estipulados conjuntamente com os demais Poderes na lei de diretrizes orçamentárias.

§ 2º O encaminhamento da proposta, ouvidos os outros tribunais interessados, compete:

I - no âmbito da União, aos Presidentes do Supremo Tribunal Federal e dos Tribunais Superiores, com a aprovação dos respectivos tribunais;

II - no âmbito dos Estados e no do Distrito Federal e Territórios, aos Presidentes dos Tribunais de Justiça, com a aprovação dos respectivos tribunais.

§ 3º Se os órgãos referidos no § 2º não encaminharem as respectivas propostas orçamentárias dentro do prazo estabelecido na lei de diretrizes orçamentárias, o Poder Executivo considerará, para fins de consolidação da proposta orçamentária anual, os valores aprovados na lei orçamentária vigente, ajustados de acordo com os limites estipulados na forma do § 1º deste artigo. (Incluído pela Emenda Constitucional nº 45, de 2004)

§ 4º Se as propostas orçamentárias de que trata este artigo forem encaminhadas em desacordo com os limites estipulados na forma do § 1º, o Poder Executivo procederá aos ajustes necessários para fins de consolidação da proposta orçamentária anual. (Incluído pela Emenda Constitucional nº 45, de 2004)

§ 5º Durante a execução orçamentária do exercício, não poderá haver a realização de despesas ou a assunção de obrigações que extrapolem os limites estabelecidos na lei de diretrizes orçamentárias, exceto se previamente autorizadas, mediante a abertura de créditos suplementares ou especiais. (Incluído pela Emenda Constitucional nº 45, de 2004)

Art. 100. Os pagamentos devidos pelas Fazendas Públicas Federal, Estaduais, Distrital e Municipais, em virtude de sentença judiciária, far-se-ão exclusivamente na ordem cronológica de apresentação dos precatórios e à conta dos créditos respectivos, proibida a designação de casos ou de pessoas nas dotações orçamentárias e nos créditos adicionais abertos para este

fim. (Redação dada pela Emenda Constitucional nº 62, de 2009). (Vide Emenda Constitucional nº 62, de 2009) (Vide ADI 4425)

§ 1º Os débitos de natureza alimentícia compreendem aqueles decorrentes de salários, vencimentos, proventos, pensões e suas complementações, benefícios previdenciários e indenizações por morte ou por invalidez, fundadas em responsabilidade civil, em virtude de sentença judicial transitada em julgado, e serão pagos com preferência sobre todos os demais débitos, exceto sobre aqueles referidos no § 2º deste artigo. (Redação dada pela Emenda Constitucional nº 62, de 2009).

§ 2º Os débitos de natureza alimentícia cujos titulares, originários ou por sucessão hereditária, tenham 60 (sessenta) anos de idade, ou sejam portadores de doença grave, ou pessoas com deficiência, assim definidos na forma da lei, serão pagos com preferência sobre todos os demais débitos, até o valor equivalente ao triplo fixado em lei para os fins do disposto no § 3º deste artigo, admitido o fracionamento para essa finalidade, sendo que o restante será pago na ordem cronológica de apresentação do precatório. (Redação dada pela Emenda Constitucional nº 94, de 2016)

§ 3º O disposto no caput deste artigo relativamente à expedição de precatórios não se aplica aos pagamentos de obrigações definidas em leis como de pequeno valor que as Fazendas referidas devam fazer em virtude de sentença judicial transitada em julgado. (Redação dada pela Emenda Constitucional nº 62, de 2009).

§ 4º Para os fins do disposto no § 3º, poderão ser fixados, por leis próprias, valores distintos às entidades de direito público, segundo as diferentes capacidades econômicas, sendo o mínimo igual ao valor do maior benefício do regime geral de previdência social. (Redação dada pela Emenda Constitucional nº 62, de 2009).

§ 5º É obrigatória a inclusão, no orçamento das entidades de direito público, de verba necessária ao pagamento de seus débitos, oriundos de sentenças transitadas em julgado, constantes de precatórios judiciários apresentados até 1º de julho, fazendo-se o pagamento até o final do exercício seguinte, quando terão seus valores atualizados monetariamente. (Redação dada pela Emenda Constitucional nº 62, de 2009).

§ 6º As dotações orçamentárias e os créditos abertos serão consignados diretamente ao Poder Judiciário, cabendo ao Presidente do Tribunal que proferir a decisão exequenda determinar o pagamento integral e autorizar, a requerimento do credor e exclusivamente para os casos de preterimento de seu direito de precedência ou de não alocação orçamentária do valor necessário à satisfação do seu débito, o sequestro da quantia respectiva. (Redação dada pela Emenda Constitucional nº 62, de 2009).

§ 7º O Presidente do Tribunal competente que, por ato comissivo ou omissivo, retardar ou tentar frustrar a liquidação regular de precatórios incorrerá em crime de responsabilidade e responderá, também, perante o Conselho Nacional de Justiça. (Incluído pela Emenda Constitucional nº 62, de 2009).

§ 8º É vedada a expedição de precatórios complementares ou suplementares de valor pago, bem como o fracionamento, repartição ou quebra do valor da execução para fins de enquadramento de parcela do total ao que dispõe o § 3º deste artigo. (Incluído pela Emenda Constitucional nº 62, de 2009).

§ 9º No momento da expedição dos precatórios, independentemente de regulamentação, deles deverá ser abatido, a título de compensação, valor correspondente aos débitos líquidos e certos, inscritos ou não em dívida ativa e constituídos contra o credor original pela Fazenda Pública devedora, incluídas parcelas vincendas de parcelamentos, ressalvados aqueles cuja execução esteja suspensa em virtude de contestação administrativa ou judicial. (Incluído pela Emenda Constitucional nº 62, de 2009). (Vide ADI 4425)

§ 10. Antes da expedição dos precatórios, o Tribunal solicitará à Fazenda Pública devedora, para resposta em até 30 (trinta) dias, sob pena de perda do direito de abatimento, informação sobre os débitos que preencham as condições estabelecidas no § 9º, para os fins nele previstos. (Incluído pela Emenda Constitucional nº 62, de 2009). (Vide ADI 4425)

§ 11. É facultada ao credor, conforme estabelecido em lei da entidade federativa devedora, a entrega de créditos em precatórios para compra de imóveis públicos do respectivo ente federado. (Incluído pela Emenda Constitucional nº 62, de 2009).

§ 12. A partir da promulgação desta Emenda Constitucional, a atualização de valores de requisitórios, após sua expedição, até o efetivo pagamento, independentemente de sua natureza, será feita pelo índice oficial de remuneração básica da caderneta de poupança, e, para fins de compensação da mora, incidirão juros simples no mesmo percentual de juros incidentes sobre a caderneta de poupança, ficando excluída a incidência de juros compensatórios. (Incluído pela Emenda Constitucional nº 62, de 2009). (Vide ADI 4425)

§ 13. O credor poderá ceder, total ou parcialmente, seus créditos em precatórios a terceiros, independentemente da concordância do devedor, não se aplicando ao cessionário o disposto nos §§ 2º e 3º. (Incluído pela Emenda Constitucional nº 62, de 2009).

§ 14. A cessão de precatórios somente produzirá efeitos após comunicação, por meio de petição protocolizada, ao tribunal de origem e à entidade devedora. (Incluído pela Emenda Constitucional nº 62, de 2009).

§ 15. Sem prejuízo do disposto neste artigo, lei complementar a esta Constituição Federal poderá estabelecer regime especial para pagamento de crédito de precatórios de Estados, Distrito Federal e Municípios, dispondo sobre vinculações à receita corrente líquida e forma e prazo de liquidação. (Incluído pela Emenda Constitucional nº 62, de 2009).

§ 16. A seu critério exclusivo e na forma de lei, a União poderá assumir débitos, oriundos de precatórios, de Estados, Distrito Federal e Municípios, refinanciando-os diretamente. (Incluído pela Emenda Constitucional nº 62, de 2009).

§ 17. A União, os Estados, o Distrito Federal e os Municípios aferirão mensalmente, em base anual, o comprometimento de suas respectivas receitas correntes líquidas com o pagamento de precatórios e obrigações de pequeno valor. (Incluído pela Emenda Constitucional nº 94, de 2016)

§ 18. Entende-se como receita corrente líquida, para os fins de que trata o § 17, o somatório das receitas tributárias, patrimoniais, industriais, agropecuárias, de contribuições e de serviços, de transferências correntes e outras receitas correntes, incluindo as oriundas do § 1º do art. 20 da Constituição Federal, verificado no período compreendido pelo segundo mês imediatamente anterior ao de referência e os 11 (onze) meses precedentes, excluídas as duplicidades, e deduzidas: (Incluído pela Emenda Constitucional nº 94, de 2016)

I - na União, as parcelas entregues aos Estados, ao Distrito Federal e aos Municípios por determinação constitucional; (Incluído pela Emenda Constitucional nº 94, de 2016)

II - nos Estados, as parcelas entregues aos Municípios por determinação constitucional; (Incluído pela Emenda Constitucional nº 94, de 2016)

III - na União, nos Estados, no Distrito Federal e nos Municípios, a contribuição dos servidores para custeio de seu sistema de previdência e assistência social e as receitas provenientes da compensação financeira referida no § 9º do art. 201 da Constituição Federal. (Incluído pela Emenda Constitucional nº 94, de 2016)

§ 19. Caso o montante total de débitos decorrentes de condenações judiciais em precatórios e obrigações de pequeno valor, em período de 12 (doze) meses, ultrapasse a média do

comprometimento percentual da receita corrente líquida nos 5 (cinco) anos imediatamente anteriores, a parcela que exceder esse percentual poderá ser financiada, excetuada dos limites de endividamento de que tratam os incisos VI e VII do art. 52 da Constituição Federal e de quaisquer outros limites de endividamento previstos, não se aplicando a esse financiamento a vedação de vinculação de receita prevista no inciso IV do art. 167 da Constituição Federal. (Incluído pela Emenda Constitucional nº 94, de 2016)

§ 20. Caso haja precatório com valor superior a 15% (quinze por cento) do montante dos precatórios apresentados nos termos do § 5º deste artigo, 15% (quinze por cento) do valor deste precatório serão pagos até o final do exercício seguinte e o restante em parcelas iguais nos cinco exercícios subsequentes, acrescidas de juros de mora e correção monetária, ou mediante acordos diretos, perante Juízos Auxiliares de Conciliação de Precatórios, com redução máxima de 40% (quarenta por cento) do valor do crédito atualizado, desde que em relação ao crédito não penda recurso ou defesa judicial e que sejam observados os requisitos definidos na regulamentação editada pelo ente federado. (Incluído pela Emenda Constitucional nº 94, de 2016)

SEÇÃO II
DO SUPREMO TRIBUNAL FEDERAL

Art. 101. O Supremo Tribunal Federal compõe-se de onze Ministros, escolhidos dentre cidadãos com mais de trinta e cinco e menos de sessenta e cinco anos de idade, de notável saber jurídico e reputação ilibada.

Parágrafo único. Os Ministros do Supremo Tribunal Federal serão nomeados pelo Presidente da República, depois de aprovada a escolha pela maioria absoluta do Senado Federal.

Art. 102. Compete ao Supremo Tribunal Federal, precipuamente, a guarda da Constituição, cabendo-lhe:

I - processar e julgar, originariamente:

a) a ação direta de inconstitucionalidade de lei ou ato normativo federal ou estadual e a ação declaratória de constitucionalidade de lei ou ato normativo federal; (Redação dada pela Emenda Constitucional nº 3, de 1993)

b) nas infrações penais comuns, o Presidente da República, o Vice-Presidente, os membros do Congresso Nacional, seus próprios Ministros e o Procurador-Geral da República;

c) nas infrações penais comuns e nos crimes de responsabilidade, os Ministros de Estado e os Comandantes da Marinha, do Exército e da Aeronáutica, ressalvado o disposto no art. 52, I, os membros dos Tribunais Superiores, os do Tribunal de Contas da União e os chefes de missão diplomática de caráter permanente; (Redação dada pela Emenda Constitucional nº 23, de 1999)

d) o *habeas corpus* , sendo paciente qualquer das pessoas referidas nas alíneas anteriores; o mandado de segurança e o *habeas data* contra atos do Presidente da República, das Mesas da Câmara dos Deputados e do Senado Federal, do Tribunal de Contas da União, do Procurador-Geral da República e do próprio Supremo Tribunal Federal;

e) o litígio entre Estado estrangeiro ou organismo internacional e a União, o Estado, o Distrito Federal ou o Território;

f) as causas e os conflitos entre a União e os Estados, a União e o Distrito Federal, ou entre uns e outros, inclusive as respectivas entidades da administração indireta;

g) a extradição solicitada por Estado estrangeiro;

h) a homologação das sentenças estrangeiras e a concessão do "exequatur" às cartas rogatórias, que podem ser conferidas pelo regimento interno a seu Presidente; (Revogado pela Emenda Constitucional nº 45, de 2004)

i) o ***habeas corpus*** , quando o coator for Tribunal Superior ou quando o coator ou o paciente for autoridade ou funcionário cujos atos estejam sujeitos diretamente à jurisdição do Supremo Tribunal Federal, ou se trate de crime sujeito à mesma jurisdição em uma única instância; (Redação dada pela Emenda Constitucional nº 22, de 1999)

j) a revisão criminal e a ação rescisória de seus julgados;

l) a reclamação para a preservação de sua competência e garantia da autoridade de suas decisões;

m) a execução de sentença nas causas de sua competência originária, facultada a delegação de atribuições para a prática de atos processuais;

n) a ação em que todos os membros da magistratura sejam direta ou indiretamente interessados, e aquela em que mais da metade dos membros do tribunal de origem estejam impedidos ou sejam direta ou indiretamente interessados;

o) os conflitos de competência entre o Superior Tribunal de Justiça e quaisquer tribunais, entre Tribunais Superiores, ou entre estes e qualquer outro tribunal;

p) o pedido de medida cautelar das ações diretas de inconstitucionalidade;

q) o mandado de injunção, quando a elaboração da norma regulamentadora for atribuição do Presidente da República, do Congresso Nacional, da Câmara dos Deputados, do Senado Federal, das Mesas de uma dessas Casas Legislativas, do Tribunal de Contas da União, de um dos Tribunais Superiores, ou do próprio Supremo Tribunal Federal;

r) as ações contra o Conselho Nacional de Justiça e contra o Conselho Nacional do Ministério Público; (Incluída pela Emenda Constitucional nº 45, de 2004)

II - julgar, em recurso ordinário:

a) o *habeas corpus* , o mandado de segurança, o *habeas data* e o mandado de injunção decididos em única instância pelos Tribunais Superiores, se denegatória a decisão;

b) o crime político;

III - julgar, mediante recurso extraordinário, as causas decididas em única ou última instância, quando a decisão recorrida:

a) contrariar dispositivo desta Constituição;

b) declarar a inconstitucionalidade de tratado ou lei federal;

c) julgar válida lei ou ato de governo local contestado em face desta Constituição.

d) julgar válida lei local contestada em face de lei federal. (Incluída pela Emenda Constitucional nº 45, de 2004)

§ 1º A argüição de descumprimento de preceito fundamental, decorrente desta Constituição, será apreciada pelo Supremo Tribunal Federal, na forma da lei. (Transformado em § 1º pela Emenda Constitucional nº 3, de 17/03/93)

§ 2º As decisões definitivas de mérito, proferidas pelo Supremo Tribunal Federal, nas ações diretas de inconstitucionalidade e nas ações declaratórias de constitucionalidade produzirão eficácia contra todos e efeito vinculante, relativamente aos demais órgãos do Poder Judiciário e à administração pública direta e indireta, nas esferas federal, estadual e municipal. (Redação dada pela Emenda Constitucional nº 45, de 2004) (Vide ADIN 3392)

§ 3º No recurso extraordinário o recorrente deverá demonstrar a repercussão geral das questões constitucionais discutidas no caso, nos termos da lei, a fim de que o Tribunal examine a admissão do recurso, somente podendo recusá-lo pela manifestação de dois terços de seus membros. (Incluída pela Emenda Constitucional nº 45, de 2004)

Art. 103. Podem propor a ação direta de inconstitucionalidade e a ação declaratória de constitucionalidade: (Redação dada pela Emenda Constitucional nº 45, de 2004) (Vide Lei nº 13.105, de 2015) (Vigência)

I - o Presidente da República;

II - a Mesa do Senado Federal;

III - a Mesa da Câmara dos Deputados;

IV - a Mesa de Assembléia Legislativa ou da Câmara Legislativa do Distrito Federal; (Redação dada pela Emenda Constitucional nº 45, de 2004)

V - o Governador de Estado ou do Distrito Federal; (Redação dada pela Emenda Constitucional nº 45, de 2004)

VI - o Procurador-Geral da República;

VII - o Conselho Federal da Ordem dos Advogados do Brasil;

VIII - partido político com representação no Congresso Nacional;

IX - confederação sindical ou entidade de classe de âmbito nacional.

§ 1º O Procurador-Geral da República deverá ser previamente ouvido nas ações de inconstitucionalidade e em todos os processos de competência do Supremo Tribunal Federal.

§ 2º Declarada a inconstitucionalidade por omissão de medida para tornar efetiva norma constitucional, será dada ciência ao Poder competente para a adoção das providências necessárias e, em se tratando de órgão administrativo, para fazê-lo em trinta dias.

§ 3º Quando o Supremo Tribunal Federal apreciar a inconstitucionalidade, em tese, de norma legal ou ato normativo, citará, previamente, o Advogado-Geral da União, que defenderá o ato ou texto impugnado.

Art. 103-A. O Supremo Tribunal Federal poderá, de ofício ou por provocação, mediante decisão de dois terços dos seus membros, após reiteradas decisões sobre matéria constitucional, aprovar súmula que, a partir de sua publicação na imprensa oficial, terá efeito vinculante em relação aos demais órgãos do Poder Judiciário e à administração pública direta e indireta, nas esferas federal, estadual e municipal, bem como proceder à sua revisão ou cancelamento, na forma estabelecida em lei. (Incluído pela Emenda Constitucional nº 45, de 2004) (Vide Lei nº 11.417, de 2006).

§ 1º A súmula terá por objetivo a validade, a interpretação e a eficácia de normas determinadas, acerca das quais haja controvérsia atual entre órgãos judiciários ou entre esses e

a administração pública que acarrete grave insegurança jurídica e relevante multiplicação de processos sobre questão idêntica. (Incluído pela Emenda Constitucional nº 45, de 2004)

§ 2º Sem prejuízo do que vier a ser estabelecido em lei, a aprovação, revisão ou cancelamento de súmula poderá ser provocada por aqueles que podem propor a ação direta de inconstitucionalidade. (Incluído pela Emenda Constitucional nº 45, de 2004)

§ 3º Do ato administrativo ou decisão judicial que contrariar a súmula aplicável ou que indevidamente a aplicar, caberá reclamação ao Supremo Tribunal Federal que, julgando-a procedente, anulará o ato administrativo ou cassará a decisão judicial reclamada, e determinará que outra seja proferida com ou sem a aplicação da súmula, conforme o caso. (Incluído pela Emenda Constitucional nº 45, de 2004)

Art. 103-B. O Conselho Nacional de Justiça compõe-se de 15 (quinze) membros com mandato de 2 (dois) anos, admitida 1 (uma) recondução, sendo: (Redação dada pela Emenda Constitucional nº 61, de 2009)

~~I - um Ministro do Supremo Tribunal Federal, indicado pelo respectivo tribunal; (Incluído pela Emenda Constitucional nº 45, de 2004)~~

I - o Presidente do Supremo Tribunal Federal; (Redação dada pela Emenda Constitucional nº 61, de 2009)

II - um Ministro do Superior Tribunal de Justiça, indicado pelo respectivo tribunal; (Incluído pela Emenda Constitucional nº 45, de 2004)

III - um Ministro do Tribunal Superior do Trabalho, indicado pelo respectivo tribunal; (Incluído pela Emenda Constitucional nº 45, de 2004)

IV - um desembargador de Tribunal de Justiça, indicado pelo Supremo Tribunal Federal; (Incluído pela Emenda Constitucional nº 45, de 2004)

V - um juiz estadual, indicado pelo Supremo Tribunal Federal; (Incluído pela Emenda Constitucional nº 45, de 2004)

VI - um juiz de Tribunal Regional Federal, indicado pelo Superior Tribunal de Justiça; (Incluído pela Emenda Constitucional nº 45, de 2004)

VII - um juiz federal, indicado pelo Superior Tribunal de Justiça; (Incluído pela Emenda Constitucional nº 45, de 2004)

VIII - um juiz de Tribunal Regional do Trabalho, indicado pelo Tribunal Superior do Trabalho; (Incluído pela Emenda Constitucional nº 45, de 2004)

IX - um juiz do trabalho, indicado pelo Tribunal Superior do Trabalho; (Incluído pela Emenda Constitucional nº 45, de 2004)

X - um membro do Ministério Público da União, indicado pelo Procurador-Geral da República; (Incluído pela Emenda Constitucional nº 45, de 2004)

XI um membro do Ministério Público estadual, escolhido pelo Procurador-Geral da República dentre os nomes indicados pelo órgão competente de cada instituição estadual; (Incluído pela Emenda Constitucional nº 45, de 2004)

XII - dois advogados, indicados pelo Conselho Federal da Ordem dos Advogados do Brasil; (Incluído pela Emenda Constitucional nº 45, de 2004)

XIII - dois cidadãos, de notável saber jurídico e reputação ilibada, indicados um pela Câmara dos Deputados e outro pelo Senado Federal. (Incluído pela Emenda Constitucional nº 45, de 2004)

§ 1º O Conselho será presidido pelo Presidente do Supremo Tribunal Federal e, nas suas ausências e impedimentos, pelo Vice-Presidente do Supremo Tribunal Federal. (Redação dada pela Emenda Constitucional nº 61, de 2009)

§ 2º Os demais membros do Conselho serão nomeados pelo Presidente da República, depois de aprovada a escolha pela maioria absoluta do Senado Federal. (Redação dada pela Emenda Constitucional nº 61, de 2009)

§ 3º Não efetuadas, no prazo legal, as indicações previstas neste artigo, caberá a escolha ao Supremo Tribunal Federal. (Incluído pela Emenda Constitucional nº 45, de 2004)

§ 4º Compete ao Conselho o controle da atuação administrativa e financeira do Poder Judiciário e do cumprimento dos deveres funcionais dos juízes, cabendo-lhe, além de outras atribuições que lhe forem conferidas pelo Estatuto da Magistratura: (Incluído pela Emenda Constitucional nº 45, de 2004)

I - zelar pela autonomia do Poder Judiciário e pelo cumprimento do Estatuto da Magistratura, podendo expedir atos regulamentares, no âmbito de sua competência, ou recomendar providências; (Incluído pela Emenda Constitucional nº 45, de 2004)

II - zelar pela observância do art. 37 e apreciar, de ofício ou mediante provocação, a legalidade dos atos administrativos praticados por membros ou órgãos do Poder Judiciário, podendo desconstituí-los, revê-los ou fixar prazo para que se adotem as providências necessárias ao exato cumprimento da lei, sem prejuízo da competência do Tribunal de Contas da União; (Incluído pela Emenda Constitucional nº 45, de 2004)

III - receber e conhecer das reclamações contra membros ou órgãos do Poder Judiciário, inclusive contra seus serviços auxiliares, serventias e órgãos prestadores de serviços notariais e de registro que atuem por delegação do poder público ou oficializados, sem prejuízo da competência disciplinar e correicional dos tribunais, podendo avocar processos disciplinares em curso e determinar a remoção, a disponibilidade ou a aposentadoria com subsídios ou proventos proporcionais ao tempo de serviço e aplicar outras sanções administrativas, assegurada ampla defesa; (Incluído pela Emenda Constitucional nº 45, de 2004)

IV - representar ao Ministério Público, no caso de crime contra a administração pública ou de abuso de autoridade; (Incluído pela Emenda Constitucional nº 45, de 2004)

V - rever, de ofício ou mediante provocação, os processos disciplinares de juízes e membros de tribunais julgados há menos de um ano; (Incluído pela Emenda Constitucional nº 45, de 2004)

VI - elaborar semestralmente relatório estatístico sobre processos e sentenças prolatadas, por unidade da Federação, nos diferentes órgãos do Poder Judiciário; (Incluído pela Emenda Constitucional nº 45, de 2004)

VII - elaborar relatório anual, propondo as providências que julgar necessárias, sobre a situação do Poder Judiciário no País e as atividades do Conselho, o qual deve integrar mensagem do Presidente do Supremo Tribunal Federal a ser remetida ao Congresso Nacional, por ocasião da abertura da sessão legislativa. (Incluído pela Emenda Constitucional nº 45, de 2004)

§ 5º O Ministro do Superior Tribunal de Justiça exercerá a função de Ministro-Corregedor e ficará excluído da distribuição de processos no Tribunal, competindo-lhe, além das atribuições

que lhe forem conferidas pelo Estatuto da Magistratura, as seguintes: (Incluído pela Emenda Constitucional nº 45, de 2004)

I receber as reclamações e denúncias, de qualquer interessado, relativas aos magistrados e aos serviços judiciários; (Incluído pela Emenda Constitucional nº 45, de 2004)

II exercer funções executivas do Conselho, de inspeção e de correição geral; (Incluído pela Emenda Constitucional nº 45, de 2004)

III requisitar e designar magistrados, delegando-lhes atribuições, e requisitar servidores de juízos ou tribunais, inclusive nos Estados, Distrito Federal e Territórios. (Incluído pela Emenda Constitucional nº 45, de 2004)

§ 6º Junto ao Conselho oficiarão o Procurador-Geral da República e o Presidente do Conselho Federal da Ordem dos Advogados do Brasil. (Incluído pela Emenda Constitucional nº 45, de 2004)

§ 7º A União, inclusive no Distrito Federal e nos Territórios, criará ouvidorias de justiça, competentes para receber reclamações e denúncias de qualquer interessado contra membros ou órgãos do Poder Judiciário, ou contra seus serviços auxiliares, representando diretamente ao Conselho Nacional de Justiça. (Incluído pela Emenda Constitucional nº 45, de 2004)

SEÇÃO III
DO SUPERIOR TRIBUNAL DE JUSTIÇA

Art. 104. O Superior Tribunal de Justiça compõe-se de, no mínimo, trinta e três Ministros.

Parágrafo único. Os Ministros do Superior Tribunal de Justiça serão nomeados pelo Presidente da República, dentre brasileiros com mais de trinta e cinco e menos de sessenta e cinco anos, de notável saber jurídico e reputação ilibada, depois de aprovada a escolha pela maioria absoluta do Senado Federal, sendo: (Redação dada pela Emenda Constitucional nº 45, de 2004)

I - um terço dentre juízes dos Tribunais Regionais Federais e um terço dentre desembargadores dos Tribunais de Justiça, indicados em lista tríplice elaborada pelo próprio Tribunal;

II - um terço, em partes iguais, dentre advogados e membros do Ministério Público Federal, Estadual, do Distrito Federal e Territórios, alternadamente, indicados na forma do art. 94.

Art. 105. Compete ao Superior Tribunal de Justiça:

I - processar e julgar, originariamente:

a) nos crimes comuns, os Governadores dos Estados e do Distrito Federal, e, nestes e nos de responsabilidade, os desembargadores dos Tribunais de Justiça dos Estados e do Distrito Federal, os membros dos Tribunais de Contas dos Estados e do Distrito Federal, os dos Tribunais Regionais Federais, dos Tribunais Regionais Eleitorais e do Trabalho, os membros dos Conselhos ou Tribunais de Contas dos Municípios e os do Ministério Público da União que oficiem perante tribunais;

b) os mandados de segurança e os ***habeas data*** contra ato de Ministro de Estado, dos Comandantes da Marinha, do Exército e da Aeronáutica ou do próprio Tribunal; (Redação dada pela Emenda Constitucional nº 23, de 1999)

c) os ***habeas corpus*** , quando o coator ou paciente for qualquer das pessoas mencionadas na alínea "a", ou quando o coator for tribunal sujeito à sua jurisdição, Ministro de Estado ou

Comandante da Marinha, do Exército ou da Aeronáutica, ressalvada a competência da Justiça Eleitoral; (Redação dada pela Emenda Constitucional nº 23, de 1999)

d) os conflitos de competência entre quaisquer tribunais, ressalvado o disposto no art. 102, I, "o", bem como entre tribunal e juízes a ele não vinculados e entre juízes vinculados a tribunais diversos;

e) as revisões criminais e as ações rescisórias de seus julgados;

f) a reclamação para a preservação de sua competência e garantia da autoridade de suas decisões;

g) os conflitos de atribuições entre autoridades administrativas e judiciárias da União, ou entre autoridades judiciárias de um Estado e administrativas de outro ou do Distrito Federal, ou entre as deste e da União;

h) o mandado de injunção, quando a elaboração da norma regulamentadora for atribuição de órgão, entidade ou autoridade federal, da administração direta ou indireta, excetuados os casos de competência do Supremo Tribunal Federal e dos órgãos da Justiça Militar, da Justiça Eleitoral, da Justiça do Trabalho e da Justiça Federal;

i) a homologação de sentenças estrangeiras e a concessão de exequatur às cartas rogatórias; (Incluída pela Emenda Constitucional nº 45, de 2004)

II - julgar, em recurso ordinário:

a) os habeas corpus decididos em única ou última instância pelos Tribunais Regionais Federais ou pelos tribunais dos Estados, do Distrito Federal e Territórios, quando a decisão for denegatória;

b) os mandados de segurança decididos em única instância pelos Tribunais Regionais Federais ou pelos tribunais dos Estados, do Distrito Federal e Territórios, quando denegatória a decisão;

c) as causas em que forem partes Estado estrangeiro ou organismo internacional, de um lado, e, do outro, Município ou pessoa residente ou domiciliada no País;

III - julgar, em recurso especial, as causas decididas, em única ou última instância, pelos Tribunais Regionais Federais ou pelos tribunais dos Estados, do Distrito Federal e Territórios, quando a decisão recorrida:

a) contrariar tratado ou lei federal, ou negar-lhes vigência;

b) julgar válido ato de governo local contestado em face de lei federal; (Redação dada pela Emenda Constitucional nº 45, de 2004)

c) der a lei federal interpretação divergente da que lhe haja atribuído outro tribunal.

Parágrafo único. Funcionarão junto ao Superior Tribunal de Justiça: (Redação dada pela Emenda Constitucional nº 45, de 2004)

I - a Escola Nacional de Formação e Aperfeiçoamento de Magistrados, cabendo-lhe, dentre outras funções, regulamentar os cursos oficiais para o ingresso e promoção na carreira; (Incluído pela Emenda Constitucional nº 45, de 2004)

II - o Conselho da Justiça Federal, cabendo-lhe exercer, na forma da lei, a supervisão administrativa e orçamentária da Justiça Federal de primeiro e segundo graus, como órgão

central do sistema e com poderes correicionais, cujas decisões terão caráter vinculante. (Incluído pela Emenda Constitucional nº 45, de 2004)

SEÇÃO IV
DOS TRIBUNAIS REGIONAIS FEDERAIS E DOS JUÍZES FEDERAIS

Art. 106. São órgãos da Justiça Federal:

I - os Tribunais Regionais Federais;

II - os Juízes Federais.

Art. 107. Os Tribunais Regionais Federais compõem-se de, no mínimo, sete juízes, recrutados, quando possível, na respectiva região e nomeados pelo Presidente da República dentre brasileiros com mais de trinta e menos de sessenta e cinco anos, sendo:

I - um quinto dentre advogados com mais de dez anos de efetiva atividade profissional e membros do Ministério Público Federal com mais de dez anos de carreira;

II - os demais, mediante promoção de juízes federais com mais de cinco anos de exercício, por antigüidade e merecimento, alternadamente.

§ 1º A lei disciplinará a remoção ou a permuta de juízes dos Tribunais Regionais Federais e determinará sua jurisdição e sede. (Renumerado pela Emenda Constitucional nº 45, de 2004)

§ 2º Os Tribunais Regionais Federais instalarão a justiça itinerante, com a realização de audiências e demais funções da atividade jurisdicional, nos limites territoriais da respectiva jurisdição, servindo-se de equipamentos públicos e comunitários. (Incluído pela Emenda Constitucional nº 45, de 2004)

§ 3º Os Tribunais Regionais Federais poderão funcionar descentralizadamente, constituindo Câmaras regionais, a fim de assegurar o pleno acesso do jurisdicionado à justiça em todas as fases do processo. (Incluído pela Emenda Constitucional nº 45, de 2004)

Art. 108. Compete aos Tribunais Regionais Federais:

I - processar e julgar, originariamente:

a) os juízes federais da área de sua jurisdição, incluídos os da Justiça Militar e da Justiça do Trabalho, nos crimes comuns e de responsabilidade, e os membros do Ministério Público da União, ressalvada a competência da Justiça Eleitoral;

b) as revisões criminais e as ações rescisórias de julgados seus ou dos juízes federais da região;

c) os mandados de segurança e os *habeas data* contra ato do próprio Tribunal ou de juiz federal;

d) os habeas corpus, quando a autoridade coatora for juiz federal;

e) os conflitos de competência entre juízes federais vinculados ao Tribunal;

II - julgar, em grau de recurso, as causas decididas pelos juízes federais e pelos juízes estaduais no exercício da competência federal da área de sua jurisdição.

Art. 109. Aos juízes federais compete processar e julgar:

I - as causas em que a União, entidade autárquica ou empresa pública federal forem interessadas na condição de autoras, rés, assistentes ou oponentes, exceto as de falência, as de acidentes de trabalho e as sujeitas à Justiça Eleitoral e à Justiça do Trabalho;

II - as causas entre Estado estrangeiro ou organismo internacional e Município ou pessoa domiciliada ou residente no País;

III - as causas fundadas em tratado ou contrato da União com Estado estrangeiro ou organismo internacional;

IV - os crimes políticos e as infrações penais praticadas em detrimento de bens, serviços ou interesse da União ou de suas entidades autárquicas ou empresas públicas, excluídas as contravenções e ressalvada a competência da Justiça Militar e da Justiça Eleitoral;

V - os crimes previstos em tratado ou convenção internacional, quando, iniciada a execução no País, o resultado tenha ou devesse ter ocorrido no estrangeiro, ou reciprocamente;

V-A as causas relativas a direitos humanos a que se refere o § 5º deste artigo; (Incluído pela Emenda Constitucional nº 45, de 2004)

VI - os crimes contra a organização do trabalho e, nos casos determinados por lei, contra o sistema financeiro e a ordem econômico-financeira;

VII - os *habeas corpus* , em matéria criminal de sua competência ou quando o constrangimento provier de autoridade cujos atos não estejam diretamente sujeitos a outra jurisdição;

VIII - os mandados de segurança e os *habeas data* contra ato de autoridade federal, excetuados os casos de competência dos tribunais federais;

IX - os crimes cometidos a bordo de navios ou aeronaves, ressalvada a competência da Justiça Militar;

X - os crimes de ingresso ou permanência irregular de estrangeiro, a execução de carta rogatória, após o "exequatur", e de sentença estrangeira, após a homologação, as causas referentes à nacionalidade, inclusive a respectiva opção, e à naturalização;

XI - a disputa sobre direitos indígenas.

§ 1º As causas em que a União for autora serão aforadas na seção judiciária onde tiver domicílio a outra parte.

§ 2º As causas intentadas contra a União poderão ser aforadas na seção judiciária em que for domiciliado o autor, naquela onde houver ocorrido o ato ou fato que deu origem à demanda ou onde esteja situada a coisa, ou, ainda, no Distrito Federal.

§ 3º Serão processadas e julgadas na justiça estadual, no foro do domicílio dos segurados ou beneficiários, as causas em que forem parte instituição de previdência social e segurado, sempre que a comarca não seja sede de vara do juízo federal, e, se verificada essa condição, a lei poderá permitir que outras causas sejam também processadas e julgadas pela justiça estadual.

§ 4º Na hipótese do parágrafo anterior, o recurso cabível será sempre para o Tribunal Regional Federal na área de jurisdição do juiz de primeiro grau.

§ 5º Nas hipóteses de grave violação de direitos humanos, o Procurador-Geral da República, com a finalidade de assegurar o cumprimento de obrigações decorrentes de tratados internacionais de direitos humanos dos quais o Brasil seja parte, poderá suscitar, perante o Superior Tribunal de Justiça, em qualquer fase do inquérito ou processo, incidente de deslocamento de competência para a Justiça Federal. (Incluído pela Emenda Constitucional nº 45, de 2004)

Art. 110. Cada Estado, bem como o Distrito Federal, constituirá uma seção judiciária que terá por sede a respectiva Capital, e varas localizadas segundo o estabelecido em lei.

Parágrafo único. Nos Territórios Federais, a jurisdição e as atribuições cometidas aos juízes federais caberão aos juízes da justiça local, na forma da lei.

Seção V

(Redação dada pela Emenda Constitucional nº 92, de 2016)

Do Tribunal Superior do Trabalho, dos Tribunais Regionais do Trabalho e dos Juízes do Trabalho

Art. 111. São órgãos da Justiça do Trabalho:

I - o Tribunal Superior do Trabalho;

II - os Tribunais Regionais do Trabalho;

~~III - as Juntas de Conciliação e Julgamento.~~

III - Juizes do Trabalho. (Redação dada pela Emenda Constitucional nº 24, de 1999)

§ 1º. O Tribunal Superior do Trabalho compor-se-á de dezessete Ministros, togados e vitalícios, escolhidos dentre brasileiros com mais de trinta e cinco e menos de sessenta e cinco anos, nomeados pelo Presidente da República, após aprovação pelo Senado Federal, dos quais onze escolhidos dentre juizes dos Tribunais Regionais do Trabalho, integrantes da carreira da magistratura trabalhista, três dentre advogados e três dentre membros do Ministério Público do Trabalho. (Redação dada pela Emenda Constitucional nº 24, de 1999) (Revogado pela Emenda Constitucional nº 45, de 2004)

I - dezessete togados e vitalícios, dos quais onze escolhidos dentre juízes de carreira da magistratura trabalhista, três dentre advogados e três dentre membros do Ministério Público do Trabalho;

II - dez classistas temporários, com representação paritária dos trabalhadores e empregadores. (Revogado pela Emenda Constitucional nº 24, de 1999)

§ 2º. O Tribunal encaminhará ao Presidente da República listas tríplices, observando-se, quanto às vagas destinadas aos advogados e aos membros do Ministério Público, o disposto no art. 94; as listas tríplices para o provimento de cargos destinados aos juízes da magistratura trabalhista de carreira deverão ser elaboradas pelos Ministros togados e vitalícios. (Redação dada pela Emenda Constitucional nº 24, de 1999) (Revogado pela Emenda Constitucional nº 45, de 2004)

§ 3º A lei disporá sobre a competência do Tribunal Superior do Trabalho. (Revogado pela Emenda Constitucional nº 45, de 2004)

Art. 111-A. O Tribunal Superior do Trabalho compor-se-á de vinte e sete Ministros, escolhidos dentre brasileiros com mais de trinta e cinco anos e menos de sessenta e cinco anos, de notável saber jurídico e reputação ilibada, nomeados pelo Presidente da República após aprovação pela maioria absoluta do Senado Federal, sendo: (Redação dada pela Emenda Constitucional nº 92, de 2016)

I um quinto dentre advogados com mais de dez anos de efetiva atividade profissional e membros do Ministério Público do Trabalho com mais de dez anos de efetivo exercício, observado o disposto no art. 94; (Incluído pela Emenda Constitucional nº 45, de 2004)

II os demais dentre juízes dos Tribunais Regionais do Trabalho, oriundos da magistratura da carreira, indicados pelo próprio Tribunal Superior. (Incluído pela Emenda Constitucional nº 45, de 2004)

§ 1º A lei disporá sobre a competência do Tribunal Superior do Trabalho. (Incluído pela Emenda Constitucional nº 45, de 2004)

§ 2º Funcionarão junto ao Tribunal Superior do Trabalho: (Incluído pela Emenda Constitucional nº 45, de 2004)

I a Escola Nacional de Formação e Aperfeiçoamento de Magistrados do Trabalho, cabendo-lhe, dentre outras funções, regulamentar os cursos oficiais para o ingresso e promoção na carreira; (Incluído pela Emenda Constitucional nº 45, de 2004)

II o Conselho Superior da Justiça do Trabalho, cabendo-lhe exercer, na forma da lei, a supervisão administrativa, orçamentária, financeira e patrimonial da Justiça do Trabalho de primeiro e segundo graus, como órgão central do sistema, cujas decisões terão efeito vinculante. (Incluído pela Emenda Constitucional nº 45, de 2004)

§ 3º Compete ao Tribunal Superior do Trabalho processar e julgar, originariamente, a reclamação para a preservação de sua competência e garantia da autoridade de suas decisões. (Incluído pela Emenda Constitucional nº 92, de 2016)

Art. 112. A lei criará varas da Justiça do Trabalho, podendo, nas comarcas não abrangidas por sua jurisdição, atribuí-la aos juízes de direito, com recurso para o respectivo Tribunal Regional do Trabalho. (Redação dada pela Emenda Constitucional nº 45, de 2004)

Art. 113. A lei disporá sobre a constituição, investidura, jurisdição, competência, garantias e condições de exercício dos órgãos da Justiça do Trabalho. (Redação dada pela Emenda Constitucional nº 24, de 1999)

Art. 114. Compete à Justiça do Trabalho processar e julgar: (Redação dada pela Emenda Constitucional nº 45, de 2004) (Vide ADIN 3392) (Vide ADIN 3432)

I as ações oriundas da relação de trabalho, abrangidos os entes de direito público externo e da administração pública direta e indireta da União, dos Estados, do Distrito Federal e dos Municípios; (Incluído pela Emenda Constitucional nº 45, de 2004)

II as ações que envolvam exercício do direito de greve; (Incluído pela Emenda Constitucional nº 45, de 2004)

III as ações sobre representação sindical, entre sindicatos, entre sindicatos e trabalhadores, e entre sindicatos e empregadores; (Incluído pela Emenda Constitucional nº 45, de 2004)

IV os mandados de segurança, *habeas corpus* e *habeas data* , quando o ato questionado envolver matéria sujeita à sua jurisdição; (Incluído pela Emenda Constitucional nº 45, de 2004)

V os conflitos de competência entre órgãos com jurisdição trabalhista, ressalvado o disposto no art. 102, I, o; (Incluído pela Emenda Constitucional nº 45, de 2004)

VI as ações de indenização por dano moral ou patrimonial, decorrentes da relação de trabalho; (Incluído pela Emenda Constitucional nº 45, de 2004)

VII as ações relativas às penalidades administrativas impostas aos empregadores pelos órgãos de fiscalização das relações de trabalho; (Incluído pela Emenda Constitucional nº 45, de 2004)

VIII a execução, de ofício, das contribuições sociais previstas no art. 195, I, a , e II, e seus acréscimos legais, decorrentes das sentenças que proferir; (Incluído pela Emenda Constitucional nº 45, de 2004)

IX outras controvérsias decorrentes da relação de trabalho, na forma da lei. (Incluído pela Emenda Constitucional nº 45, de 2004)

§ 1º Frustrada a negociação coletiva, as partes poderão eleger árbitros.

§ 2º Recusando-se qualquer das partes à negociação coletiva ou à arbitragem, é facultado às mesmas, de comum acordo, ajuizar dissídio coletivo de natureza econômica, podendo a Justiça do Trabalho decidir o conflito, respeitadas as disposições mínimas legais de proteção ao trabalho, bem como as convencionadas anteriormente. (Redação dada pela Emenda Constitucional nº 45, de 2004) (Vide ADI nº 3423) (Vide ADI nº 3423) (Vide ADI nº 3423) (Vide ADI nº 3431) (Vide ADI nº 3432) (Vide ADI nº 3520) (Vide ADIN 3392) (Vide ADIN 3432)

§ 3º Em caso de greve em atividade essencial, com possibilidade de lesão do interesse público, o Ministério Público do Trabalho poderá ajuizar dissídio coletivo, competindo à Justiça do Trabalho decidir o conflito. (Redação dada pela Emenda Constitucional nº 45, de 2004) (Vide ADI nº 3423) (Vide ADI nº 3431) (Vide ADI nº 3520) (Vide ADIN 3392) (Vide ADIN 3432)

Art. 115. Os Tribunais Regionais do Trabalho compõem-se de, no mínimo, sete juízes, recrutados, quando possível, na respectiva região, e nomeados pelo Presidente da República dentre brasileiros com mais de trinta e menos de sessenta e cinco anos, sendo: (Redação dada pela Emenda Constitucional nº 45, de 2004)

I um quinto dentre advogados com mais de dez anos de efetiva atividade profissional e membros do Ministério Público do Trabalho com mais de dez anos de efetivo exercício, observado o disposto no art. 94; (Redação dada pela Emenda Constitucional nº 45, de 2004)

II os demais, mediante promoção de juízes do trabalho por antigüidade e merecimento, alternadamente. (Redação dada pela Emenda Constitucional nº 45, de 2004)

§ 1º Os Tribunais Regionais do Trabalho instalarão a justiça itinerante, com a realização de audiências e demais funções de atividade jurisdicional, nos limites territoriais da respectiva jurisdição, servindo-se de equipamentos públicos e comunitários. (Incluído pela Emenda Constitucional nº 45, de 2004)

§ 2º Os Tribunais Regionais do Trabalho poderão funcionar descentralizadamente, constituindo Câmaras regionais, a fim de assegurar o pleno acesso do jurisdicionado à justiça em todas as fases do processo. (Incluído pela Emenda Constitucional nº 45, de 2004)

Art. 116. Nas Varas do Trabalho, a jurisdição será exercida por um juiz singular. (Redação dada pela Emenda Constitucional nº 24, de 1999)

SEÇÃO VI
DOS TRIBUNAIS E JUÍZES ELEITORAIS

Art. 118. São órgãos da Justiça Eleitoral:

I - o Tribunal Superior Eleitoral;

II - os Tribunais Regionais Eleitorais;

III - os Juízes Eleitorais;

IV - as Juntas Eleitorais.

Art. 119. O Tribunal Superior Eleitoral compor-se-á, no mínimo, de sete membros, escolhidos:

I - mediante eleição, pelo voto secreto:

a) três juízes dentre os Ministros do Supremo Tribunal Federal;

b) dois juízes dentre os Ministros do Superior Tribunal de Justiça;

II - por nomeação do Presidente da República, dois juízes dentre seis advogados de notável saber jurídico e idoneidade moral, indicados pelo Supremo Tribunal Federal.

Parágrafo único. O Tribunal Superior Eleitoral elegerá seu Presidente e o Vice-Presidente dentre os Ministros do Supremo Tribunal Federal, e o Corregedor Eleitoral dentre os Ministros do Superior Tribunal de Justiça.

Art. 120. Haverá um Tribunal Regional Eleitoral na Capital de cada Estado e no Distrito Federal.

§ 1º - Os Tribunais Regionais Eleitorais compor-se-ão:

I - mediante eleição, pelo voto secreto:

a) de dois juízes dentre os desembargadores do Tribunal de Justiça;

b) de dois juízes, dentre juízes de direito, escolhidos pelo Tribunal de Justiça;

II - de um juiz do Tribunal Regional Federal com sede na Capital do Estado ou no Distrito Federal, ou, não havendo, de juiz federal, escolhido, em qualquer caso, pelo Tribunal Regional Federal respectivo;

III - por nomeação, pelo Presidente da República, de dois juízes dentre seis advogados de notável saber jurídico e idoneidade moral, indicados pelo Tribunal de Justiça.

§ 2º - O Tribunal Regional Eleitoral elegerá seu Presidente e o Vice-Presidente- dentre os desembargadores.

Art. 121. Lei complementar disporá sobre a organização e competência dos tribunais, dos juízes de direito e das juntas eleitorais.

§ 1º - Os membros dos tribunais, os juízes de direito e os integrantes das juntas eleitorais, no exercício de suas funções, e no que lhes for aplicável, gozarão de plenas garantias e serão inamovíveis.

§ 2º - Os juízes dos tribunais eleitorais, salvo motivo justificado, servirão por dois anos, no mínimo, e nunca por mais de dois biênios consecutivos, sendo os substitutos escolhidos na mesma ocasião e pelo mesmo processo, em número igual para cada categoria.

§ 3º - São irrecorríveis as decisões do Tribunal Superior Eleitoral, salvo as que contrariarem esta Constituição e as denegatórias de *habeas corpus* ou mandado de segurança.

§ 4º - Das decisões dos Tribunais Regionais Eleitorais somente caberá recurso quando:

I - forem proferidas contra disposição expressa desta Constituição ou de lei;

II - ocorrer divergência na interpretação de lei entre dois ou mais tribunais eleitorais;

III - versarem sobre inelegibilidade ou expedição de diplomas nas eleições federais ou estaduais;

IV - anularem diplomas ou decretarem a perda de mandatos eletivos federais ou estaduais;

V - denegarem *habeas corpus* , mandado de segurança, *habeas data* ou mandado de injunção.

SEÇÃO VII
DOS TRIBUNAIS E JUÍZES MILITARES

Art. 122. São órgãos da Justiça Militar:

I - o Superior Tribunal Militar;

II - os Tribunais e Juízes Militares instituídos por lei.

Art. 123. O Superior Tribunal Militar compor-se-á de quinze Ministros vitalícios, nomeados pelo Presidente da República, depois de aprovada a indicação pelo Senado Federal, sendo três dentre oficiais-generais da Marinha, quatro dentre oficiais-generais do Exército, três dentre oficiais-generais da Aeronáutica, todos da ativa e do posto mais elevado da carreira, e cinco dentre civis.

Parágrafo único. Os Ministros civis serão escolhidos pelo Presidente da República dentre brasileiros maiores de trinta e cinco anos, sendo:

I - três dentre advogados de notório saber jurídico e conduta ilibada, com mais de dez anos de efetiva atividade profissional;

II - dois, por escolha paritária, dentre juízes auditores e membros do Ministério Público da Justiça Militar.

Art. 124. À Justiça Militar compete processar e julgar os crimes militares definidos em lei.

Parágrafo único. A lei disporá sobre a organização, o funcionamento e a competência da Justiça Militar.

SEÇÃO VIII
DOS TRIBUNAIS E JUÍZES DOS ESTADOS

Art. 125. Os Estados organizarão sua Justiça, observados os princípios estabelecidos nesta Constituição.

§ 1º A competência dos tribunais será definida na Constituição do Estado, sendo a lei de organização judiciária de iniciativa do Tribunal de Justiça.

§ 2º Cabe aos Estados a instituição de representação de inconstitucionalidade de leis ou atos normativos estaduais ou municipais em face da Constituição Estadual, vedada a atribuição da legitimação para agir a um único órgão.

§ 3º A lei estadual poderá criar, mediante proposta do Tribunal de Justiça, a Justiça Militar estadual, constituída, em primeiro grau, pelos juízes de direito e pelos Conselhos de Justiça e,

em segundo grau, pelo próprio Tribunal de Justiça, ou por Tribunal de Justiça Militar nos Estados em que o efetivo militar seja superior a vinte mil integrantes. (Redação dada pela Emenda Constitucional nº 45, de 2004)

§ 4º Compete à Justiça Militar estadual processar e julgar os militares dos Estados, nos crimes militares definidos em lei e as ações judiciais contra atos disciplinares militares, ressalvada a competência do júri quando a vítima for civil, cabendo ao tribunal competente decidir sobre a perda do posto e da patente dos oficiais e da graduação das praças. (Redação dada pela Emenda Constitucional nº 45, de 2004)

§ 5º Compete aos juízes de direito do juízo militar processar e julgar, singularmente, os crimes militares cometidos contra civis e as ações judiciais contra atos disciplinares militares, cabendo ao Conselho de Justiça, sob a presidência de juiz de direito, processar e julgar os demais crimes militares. (Incluído pela Emenda Constitucional nº 45, de 2004)

§ 6º O Tribunal de Justiça poderá funcionar descentralizadamente, constituindo Câmaras regionais, a fim de assegurar o pleno acesso do jurisdicionado à justiça em todas as fases do processo. (Incluído pela Emenda Constitucional nº 45, de 2004)

§ 7º O Tribunal de Justiça instalará a justiça itinerante, com a realização de audiências e demais funções da atividade jurisdicional, nos limites territoriais da respectiva jurisdição, servindo-se de equipamentos públicos e comunitários. (Incluído pela Emenda Constitucional nº 45, de 2004)

Art. 126. Para dirimir conflitos fundiários, o Tribunal de Justiça proporá a criação de varas especializadas, com competência exclusiva para questões agrárias. (Redação dada pela Emenda Constitucional nº 45, de 2004)

Parágrafo único. Sempre que necessário à eficiente prestação jurisdicional, o juiz far-se-á presente no local do litígio.

CAPÍTULO IV
DAS FUNÇÕES ESSENCIAIS À JUSTIÇA
(Redação dada pela Emenda Constitucional nº 80, de 2014)

SEÇÃO I
DO MINISTÉRIO PÚBLICO

Art. 127. O Ministério Público é instituição permanente, essencial à função jurisdicional do Estado, incumbindo-lhe a defesa da ordem jurídica, do regime democrático e dos interesses sociais e individuais indisponíveis.

§ 1º - São princípios institucionais do Ministério Público a unidade, a indivisibilidade e a independência funcional.

§ 2º Ao Ministério Público é assegurada autonomia funcional e administrativa, podendo, observado o disposto no art. 169, propor ao Poder Legislativo a criação e extinção de seus cargos e serviços auxiliares, provendo-os por concurso público de provas ou de provas e títulos, a política remuneratória e os planos de carreira; a lei disporá sobre sua organização e funcionamento. (Redação dada pela Emenda Constitucional nº 19, de 1998)

§ 3º O Ministério Público elaborará sua proposta orçamentária dentro dos limites estabelecidos na lei de diretrizes orçamentárias.

§ 4º Se o Ministério Público não encaminhar a respectiva proposta orçamentária dentro do prazo estabelecido na lei de diretrizes orçamentárias, o Poder Executivo considerará, para fins de consolidação da proposta orçamentária anual, os valores aprovados na lei orçamentária vigente, ajustados de acordo com os limites estipulados na forma do § 3º. (Incluído pela Emenda Constitucional nº 45, de 2004)

§ 5º Se a proposta orçamentária de que trata este artigo for encaminhada em desacordo com os limites estipulados na forma do § 3º, o Poder Executivo procederá aos ajustes necessários para fins de consolidação da proposta orçamentária anual. (Incluído pela Emenda Constitucional nº 45, de 2004)

§ 6º Durante a execução orçamentária do exercício, não poderá haver a realização de despesas ou a assunção de obrigações que extrapolem os limites estabelecidos na lei de diretrizes orçamentárias, exceto se previamente autorizadas, mediante a abertura de créditos suplementares ou especiais. (Incluído pela Emenda Constitucional nº 45, de 2004)

Art. 128. O Ministério Público abrange:

I - o Ministério Público da União, que compreende:

a) o Ministério Público Federal;

b) o Ministério Público do Trabalho;

c) o Ministério Público Militar;

d) o Ministério Público do Distrito Federal e Territórios;

II - os Ministérios Públicos dos Estados.

§ 1º O Ministério Público da União tem por chefe o Procurador-Geral da República, nomeado pelo Presidente da República dentre integrantes da carreira, maiores de trinta e cinco anos, após a aprovação de seu nome pela maioria absoluta dos membros do Senado Federal, para mandato de dois anos, permitida a recondução.

§ 2º A destituição do Procurador-Geral da República, por iniciativa do Presidente da República, deverá ser precedida de autorização da maioria absoluta do Senado Federal.

§ 3º Os Ministérios Públicos dos Estados e o do Distrito Federal e Territórios formarão lista tríplice dentre integrantes da carreira, na forma da lei respectiva, para escolha de seu Procurador-Geral, que será nomeado pelo Chefe do Poder Executivo, para mandato de dois anos, permitida uma recondução.

§ 4º Os Procuradores-Gerais nos Estados e no Distrito Federal e Territórios poderão ser destituídos por deliberação da maioria absoluta do Poder Legislativo, na forma da lei complementar respectiva.

§ 5º Leis complementares da União e dos Estados, cuja iniciativa é facultada aos respectivos Procuradores-Gerais, estabelecerão a organização, as atribuições e o estatuto de cada Ministério Público, observadas, relativamente a seus membros:

I - as seguintes garantias:

a) vitaliciedade, após dois anos de exercício, não podendo perder o cargo senão por sentença judicial transitada em julgado;

b) inamovibilidade, salvo por motivo de interesse público, mediante decisão do órgão colegiado competente do Ministério Público, pelo voto da maioria absoluta de seus membros, assegurada ampla defesa; (Redação dada pela Emenda Constitucional nº 45, de 2004)

c) irredutibilidade de subsídio, fixado na forma do art. 39, § 4º, e ressalvado o disposto nos arts. 37, X e XI, 150, II, 153, III, 153, § 2º, I; (Redação dada pela Emenda Constitucional nº 19, de 1998)

II - as seguintes vedações:

a) receber, a qualquer título e sob qualquer pretexto, honorários, percentagens ou custas processuais;

b) exercer a advocacia;

c) participar de sociedade comercial, na forma da lei;

d) exercer, ainda que em disponibilidade, qualquer outra função pública, salvo uma de magistério;

e) exercer atividade político-partidária; (Redação dada pela Emenda Constitucional nº 45, de 2004)

f) receber, a qualquer título ou pretexto, auxílios ou contribuições de pessoas físicas, entidades públicas ou privadas, ressalvadas as exceções previstas em lei. (Incluída pela Emenda Constitucional nº 45, de 2004)

§ 6º Aplica-se aos membros do Ministério Público o disposto no art. 95, parágrafo único, V. (Incluído pela Emenda Constitucional nº 45, de 2004)

Art. 129. São funções institucionais do Ministério Público:

I - promover, privativamente, a ação penal pública, na forma da lei;

II - zelar pelo efetivo respeito dos Poderes Públicos e dos serviços de relevância pública aos direitos assegurados nesta Constituição, promovendo as medidas necessárias a sua garantia;

III - promover o inquérito civil e a ação civil pública, para a proteção do patrimônio público e social, do meio ambiente e de outros interesses difusos e coletivos;

IV - promover a ação de inconstitucionalidade ou representação para fins de intervenção da União e dos Estados, nos casos previstos nesta Constituição;

V - defender judicialmente os direitos e interesses das populações indígenas;

VI - expedir notificações nos procedimentos administrativos de sua competência, requisitando informações e documentos para instruí-los, na forma da lei complementar respectiva;

VII - exercer o controle externo da atividade policial, na forma da lei complementar mencionada no artigo anterior;

VIII - requisitar diligências investigatórias e a instauração de inquérito policial, indicados os fundamentos jurídicos de suas manifestações processuais;

IX - exercer outras funções que lhe forem conferidas, desde que compatíveis com sua finalidade, sendo-lhe vedada a representação judicial e a consultoria jurídica de entidades públicas.

§ 1º - A legitimação do Ministério Público para as ações civis previstas neste artigo não impede a de terceiros, nas mesmas hipóteses, segundo o disposto nesta Constituição e na lei.

§ 2º As funções do Ministério Público só podem ser exercidas por integrantes da carreira, que deverão residir na comarca da respectiva lotação, salvo autorização do chefe da instituição. (Redação dada pela Emenda Constitucional nº 45, de 2004)

§ 3º O ingresso na carreira do Ministério Público far-se-á mediante concurso público de provas e títulos, assegurada a participação da Ordem dos Advogados do Brasil em sua realização, exigindo-se do bacharel em direito, no mínimo, três anos de atividade jurídica e observando-se, nas nomeações, a ordem de classificação. (Redação dada pela Emenda Constitucional nº 45, de 2004)

§ 4º Aplica-se ao Ministério Público, no que couber, o disposto no art. 93. (Redação dada pela Emenda Constitucional nº 45, de 2004)

§ 5º A distribuição de processos no Ministério Público será imediata. (Incluído pela Emenda Constitucional nº 45, de 2004)

Art. 130. Aos membros do Ministério Público junto aos Tribunais de Contas aplicam-se as disposições desta seção pertinentes a direitos, vedações e forma de investidura.

Art. 130-A. O Conselho Nacional do Ministério Público compõe-se de quatorze membros nomeados pelo Presidente da República, depois de aprovada a escolha pela maioria absoluta do Senado Federal, para um mandato de dois anos, admitida uma recondução, sendo: (Incluído pela Emenda Constitucional nº 45, de 2004)

I o Procurador-Geral da República, que o preside; (Incluído pela Emenda Constitucional nº 45, de 2004)

II quatro membros do Ministério Público da União, assegurada a representação de cada uma de suas carreiras; (Incluído pela Emenda Constitucional nº 45, de 2004)

III três membros do Ministério Público dos Estados; (Incluído pela Emenda Constitucional nº 45, de 2004)

IV dois juízes, indicados um pelo Supremo Tribunal Federal e outro pelo Superior Tribunal de Justiça; (Incluído pela Emenda Constitucional nº 45, de 2004)

V dois advogados, indicados pelo Conselho Federal da Ordem dos Advogados do Brasil; (Incluído pela Emenda Constitucional nº 45, de 2004)

VI dois cidadãos de notável saber jurídico e reputação ilibada, indicados um pela Câmara dos Deputados e outro pelo Senado Federal. (Incluído pela Emenda Constitucional nº 45, de 2004)

§ 1º Os membros do Conselho oriundos do Ministério Público serão indicados pelos respectivos Ministérios Públicos, na forma da lei. (Incluído pela Emenda Constitucional nº 45, de 2004)

§ 2º Compete ao Conselho Nacional do Ministério Público o controle da atuação administrativa e financeira do Ministério Público e do cumprimento dos deveres funcionais de seus membros, cabendo lhe: (Incluído pela Emenda Constitucional nº 45, de 2004)

I zelar pela autonomia funcional e administrativa do Ministério Público, podendo expedir atos regulamentares, no âmbito de sua competência, ou recomendar providências; (Incluído pela Emenda Constitucional nº 45, de 2004)

II zelar pela observância do art. 37 e apreciar, de ofício ou mediante provocação, a legalidade dos atos administrativos praticados por membros ou órgãos do Ministério Público da União e dos

Estados, podendo desconstituí-los, revê-los ou fixar prazo para que se adotem as providências necessárias ao exato cumprimento da lei, sem prejuízo da competência dos Tribunais de Contas; (Incluído pela Emenda Constitucional nº 45, de 2004)

III receber e conhecer das reclamações contra membros ou órgãos do Ministério Público da União ou dos Estados, inclusive contra seus serviços auxiliares, sem prejuízo da competência disciplinar e correicional da instituição, podendo avocar processos disciplinares em curso, determinar a remoção, a disponibilidade ou a aposentadoria com subsídios ou proventos proporcionais ao tempo de serviço e aplicar outras sanções administrativas, assegurada ampla defesa; (Incluído pela Emenda Constitucional nº 45, de 2004)

IV rever, de ofício ou mediante provocação, os processos disciplinares de membros do Ministério Público da União ou dos Estados julgados há menos de um ano; (Incluído pela Emenda Constitucional nº 45, de 2004)

V elaborar relatório anual, propondo as providências que julgar necessárias sobre a situação do Ministério Público no País e as atividades do Conselho, o qual deve integrar a mensagem prevista no art. 84, XI. (Incluído pela Emenda Constitucional nº 45, de 2004)

§ 3º O Conselho escolherá, em votação secreta, um Corregedor nacional, dentre os membros do Ministério Público que o integram, vedada a recondução, competindo-lhe, além das atribuições que lhe forem conferidas pela lei, as seguintes: (Incluído pela Emenda Constitucional nº 45, de 2004)

I receber reclamações e denúncias, de qualquer interessado, relativas aos membros do Ministério Público e dos seus serviços auxiliares; (Incluído pela Emenda Constitucional nº 45, de 2004)

II exercer funções executivas do Conselho, de inspeção e correição geral; (Incluído pela Emenda Constitucional nº 45, de 2004)

III requisitar e designar membros do Ministério Público, delegando-lhes atribuições, e requisitar servidores de órgãos do Ministério Público. (Incluído pela Emenda Constitucional nº 45, de 2004)

§ 4º O Presidente do Conselho Federal da Ordem dos Advogados do Brasil oficiará junto ao Conselho. (Incluído pela Emenda Constitucional nº 45, de 2004)

§ 5º Leis da União e dos Estados criarão ouvidorias do Ministério Público, competentes para receber reclamações e denúncias de qualquer interessado contra membros ou órgãos do Ministério Público, inclusive contra seus serviços auxiliares, representando diretamente ao Conselho Nacional do Ministério Público. (Incluído pela Emenda Constitucional nº 45, de 2004)

SEÇÃO II

~~DA ADVOCACIA-GERAL DA UNIÃO~~

DA ADVOCACIA PÚBLICA
(Redação dada pela Emenda Constitucional nº 19, de 1998)

Art. 131. A Advocacia-Geral da União é a instituição que, diretamente ou através de órgão vinculado, representa a União, judicial e extrajudicialmente, cabendo-lhe, nos termos da lei complementar que dispuser sobre sua organização e funcionamento, as atividades de consultoria e assessoramento jurídico do Poder Executivo.

§ 1º - A Advocacia-Geral da União tem por chefe o Advogado-Geral da União, de livre nomeação pelo Presidente da República dentre cidadãos maiores de trinta e cinco anos, de notável saber jurídico e reputação ilibada.

§ 2º - O ingresso nas classes iniciais das carreiras da instituição de que trata este artigo far-se-á mediante concurso público de provas e títulos.

§ 3º - Na execução da dívida ativa de natureza tributária, a representação da União cabe à Procuradoria-Geral da Fazenda Nacional, observado o disposto em lei.

Art. 132. Os Procuradores dos Estados e do Distrito Federal, organizados em carreira, na qual o ingresso dependerá de concurso público de provas e títulos, com a participação da Ordem dos Advogados do Brasil em todas as suas fases, exercerão a representação judicial e a consultoria jurídica das respectivas unidades federadas. (Redação dada pela Emenda Constitucional nº 19, de 1998)

Parágrafo único. Aos procuradores referidos neste artigo é assegurada estabilidade após três anos de efetivo exercício, mediante avaliação de desempenho perante os órgãos próprios, após relatório circunstanciado das corregedorias. (Incluído pela Emenda Constitucional nº 19, de 1998)

SEÇÃO III
DA ADVOCACIA
(Redação dada pela Emenda Constitucional nº 80, de 2014)

Art. 133. O advogado é indispensável à administração da justiça, sendo inviolável por seus atos e manifestações no exercício da profissão, nos limites da lei.

SEÇÃO IV
DA DEFENSORIA PÚBLICA
(Redação dada pela Emenda Constitucional nº 80, de 2014)

Art. 134. A Defensoria Pública é instituição permanente, essencial à função jurisdicional do Estado, incumbindo-lhe, como expressão e instrumento do regime democrático, fundamentalmente, a orientação jurídica, a promoção dos direitos humanos e a defesa, em todos os graus, judicial e extrajudicial, dos direitos individuais e coletivos, de forma integral e gratuita, aos necessitados, na forma do inciso LXXIV do art. 5º desta Constituição Federal . (Redação dada pela Emenda Constitucional nº 80, de 2014)

§ 1º Lei complementar organizará a Defensoria Pública da União e do Distrito Federal e dos Territórios e prescreverá normas gerais para sua organização nos Estados, em cargos de carreira, providos, na classe inicial, mediante concurso público de provas e títulos, assegurada a seus integrantes a garantia da inamovibilidade e vedado o exercício da advocacia fora das atribuições institucionais. (Renumerado do parágrafo único pela Emenda Constitucional nº 45, de 2004)

§ 2º Às Defensorias Públicas Estaduais são asseguradas autonomia funcional e administrativa e a iniciativa de sua proposta orçamentária dentro dos limites estabelecidos na lei de diretrizes orçamentárias e subordinação ao disposto no art. 99, § 2º . (Incluído pela Emenda Constitucional nº 45, de 2004)

§ 3º Aplica-se o disposto no § 2º às Defensorias Públicas da União e do Distrito Federal. (Incluído pela Emenda Constitucional nº 74, de 2013)

§ 4º São princípios institucionais da Defensoria Pública a unidade, a indivisibilidade e a independência funcional, aplicando-se também, no que couber, o disposto no art. 93 e no inciso II do art. 96 desta Constituição Federal. (Incluído pela Emenda Constitucional nº 80, de 2014)

Art. 135. Os servidores integrantes das carreiras disciplinadas nas Seções II e III deste Capítulo serão remunerados na forma do art. 39, § 4º. (Redação dada pela Emenda Constitucional nº 19, de 1998)

TÍTULO V
DA DEFESA DO ESTADO E DAS INSTITUIÇÕES DEMOCRÁTICAS
CAPÍTULO I
DO ESTADO DE DEFESA E DO ESTADO DE SÍTIO
SEÇÃO I
DO ESTADO DE DEFESA

Art. 136. O Presidente da República pode, ouvidos o Conselho da República e o Conselho de Defesa Nacional, decretar estado de defesa para preservar ou prontamente restabelecer, em locais restritos e determinados, a ordem pública ou a paz social ameaçadas por grave e iminente instabilidade institucional ou atingidas por calamidades de grandes proporções na natureza.

§ 1º O decreto que instituir o estado de defesa determinará o tempo de sua duração, especificará as áreas a serem abrangidas e indicará, nos termos e limites da lei, as medidas coercitivas a vigorarem, dentre as seguintes:

I - restrições aos direitos de:

a) reunião, ainda que exercida no seio das associações;

b) sigilo de correspondência;

c) sigilo de comunicação telegráfica e telefônica;

II - ocupação e uso temporário de bens e serviços públicos, na hipótese de calamidade pública, respondendo a União pelos danos e custos decorrentes.

§ 2º O tempo de duração do estado de defesa não será superior a trinta dias, podendo ser prorrogado uma vez, por igual período, se persistirem as razões que justificaram a sua decretação.

§ 3º Na vigência do estado de defesa:

I - a prisão por crime contra o Estado, determinada pelo executor da medida, será por este comunicada imediatamente ao juiz competente, que a relaxará, se não for legal, facultado ao preso requerer exame de corpo de delito à autoridade policial;

II - a comunicação será acompanhada de declaração, pela autoridade, do estado físico e mental do detido no momento de sua autuação;

III - a prisão ou detenção de qualquer pessoa não poderá ser superior a dez dias, salvo quando autorizada pelo Poder Judiciário;

IV - é vedada a incomunicabilidade do preso.

§ 4º Decretado o estado de defesa ou sua prorrogação, o Presidente da República, dentro de vinte e quatro horas, submeterá o ato com a respectiva justificação ao Congresso Nacional, que decidirá por maioria absoluta.

§ 5º Se o Congresso Nacional estiver em recesso, será convocado, extraordinariamente, no prazo de cinco dias.

§ 6º O Congresso Nacional apreciará o decreto dentro de dez dias contados de seu recebimento, devendo continuar funcionando enquanto vigorar o estado de defesa.

§ 7º Rejeitado o decreto, cessa imediatamente o estado de defesa.

SEÇÃO II
DO ESTADO DE SÍTIO

Art. 137. O Presidente da República pode, ouvidos o Conselho da República e o Conselho de Defesa Nacional, solicitar ao Congresso Nacional autorização para decretar o estado de sítio nos casos de:

I - comoção grave de repercussão nacional ou ocorrência de fatos que comprovem a ineficácia de medida tomada durante o estado de defesa;

II - declaração de estado de guerra ou resposta a agressão armada estrangeira.

Parágrafo único. O Presidente da República, ao solicitar autorização para decretar o estado de sítio ou sua prorrogação, relatará os motivos determinantes do pedido, devendo o Congresso Nacional decidir por maioria absoluta.

Art. 138. O decreto do estado de sítio indicará sua duração, as normas necessárias a sua execução e as garantias constitucionais que ficarão suspensas, e, depois de publicado, o Presidente da República designará o executor das medidas específicas e as áreas abrangidas.

§ 1º O estado de sítio, no caso do art. 137, I, não poderá ser decretado por mais de trinta dias, nem prorrogado, de cada vez, por prazo superior; no do inciso II, poderá ser decretado por todo o tempo que perdurar a guerra ou a agressão armada estrangeira.

§ 2º Solicitada autorização para decretar o estado de sítio durante o recesso parlamentar, o Presidente do Senado Federal, de imediato, convocará extraordinariamente o Congresso Nacional para se reunir dentro de cinco dias, a fim de apreciar o ato.

§ 3º O Congresso Nacional permanecerá em funcionamento até o término das medidas coercitivas.

Art. 139. Na vigência do estado de sítio decretado com fundamento no art. 137, I, só poderão ser tomadas contra as pessoas as seguintes medidas:

I - obrigação de permanência em localidade determinada;

II - detenção em edifício não destinado a acusados ou condenados por crimes comuns;

III - restrições relativas à inviolabilidade da correspondência, ao sigilo das comunicações, à prestação de informações e à liberdade de imprensa, radiodifusão e televisão, na forma da lei;

IV - suspensão da liberdade de reunião;

V - busca e apreensão em domicílio;

VI - intervenção nas empresas de serviços públicos;

VII - requisição de bens.

Parágrafo único. Não se inclui nas restrições do inciso III a difusão de pronunciamentos de parlamentares efetuados em suas Casas Legislativas, desde que liberada pela respectiva Mesa.

SEÇÃO III
DISPOSIÇÕES GERAIS

Art. 140. A Mesa do Congresso Nacional, ouvidos os líderes partidários, designará Comissão composta de cinco de seus membros para acompanhar e fiscalizar a execução das medidas referentes ao estado de defesa e ao estado de sítio.

Art. 141. Cessado o estado de defesa ou o estado de sítio, cessarão também seus efeitos, sem prejuízo da responsabilidade pelos ilícitos cometidos por seus executores ou agentes.

Parágrafo único. Logo que cesse o estado de defesa ou o estado de sítio, as medidas aplicadas em sua vigência serão relatadas pelo Presidente da República, em mensagem ao Congresso Nacional, com especificação e justificação das providências adotadas, com relação nominal dos atingidos e indicação das restrições aplicadas.

CAPÍTULO II
DAS FORÇAS ARMADAS

Art. 142. As Forças Armadas, constituídas pela Marinha, pelo Exército e pela Aeronáutica, são instituições nacionais permanentes e regulares, organizadas com base na hierarquia e na disciplina, sob a autoridade suprema do Presidente da República, e destinam-se à defesa da Pátria, à garantia dos poderes constitucionais e, por iniciativa de qualquer destes, da lei e da ordem.

§ 1º Lei complementar estabelecerá as normas gerais a serem adotadas na organização, no preparo e no emprego das Forças Armadas.

§ 2º Não caberá *habeas corpus* em relação a punições disciplinares militares.

§ 3º Os membros das Forças Armadas são denominados militares, aplicando-se-lhes, além das que vierem a ser fixadas em lei, as seguintes disposições: (Incluído pela Emenda Constitucional nº 18, de 1998)

I - as patentes, com prerrogativas, direitos e deveres a elas inerentes, são conferidas pelo Presidente da República e asseguradas em plenitude aos oficiais da ativa, da reserva ou reformados, sendo-lhes privativos os títulos e postos militares e, juntamente com os demais membros, o uso dos uniformes das Forças Armadas; (Incluído pela Emenda Constitucional nº 18, de 1998)

II - o militar em atividade que tomar posse em cargo ou emprego público civil permanente, ressalvada a hipótese prevista no art. 37, inciso XVI, alínea "c", será transferido para a reserva, nos termos da lei; (Redação dada pela Emenda Constitucional nº 77, de 2014)

III - o militar da ativa que, de acordo com a lei, tomar posse em cargo, emprego ou função pública civil temporária, não eletiva, ainda que da administração indireta, ressalvada a hipótese prevista no art. 37, inciso XVI, alínea "c", ficará agregado ao respectivo quadro e somente poderá, enquanto permanecer nessa situação, ser promovido por antiguidade, contando-se-lhe o tempo de serviço apenas para aquela promoção e transferência para a reserva, sendo depois de dois anos de afastamento, contínuos ou não, transferido para a reserva, nos termos da lei; (Redação dada pela Emenda Constitucional nº 77, de 2014)

IV - ao militar são proibidas a sindicalização e a greve; (Incluído pela Emenda Constitucional nº 18, de 1998)

V - o militar, enquanto em serviço ativo, não pode estar filiado a partidos políticos; (Incluído pela Emenda Constitucional nº 18, de 1998)

VI - o oficial só perderá o posto e a patente se for julgado indigno do oficialato ou com ele incompatível, por decisão de tribunal militar de caráter permanente, em tempo de paz, ou de tribunal especial, em tempo de guerra; (Incluído pela Emenda Constitucional nº 18, de 1998)

VII - o oficial condenado na justiça comum ou militar a pena privativa de liberdade superior a dois anos, por sentença transitada em julgado, será submetido ao julgamento previsto no inciso anterior; (Incluído pela Emenda Constitucional nº 18, de 1998)

VIII - aplica-se aos militares o disposto no art. 7º, incisos VIII, XII, XVII, XVIII, XIX e XXV, e no art. 37, incisos XI, XIII, XIV e XV, bem como, na forma da lei e com prevalência da atividade militar, no art. 37, inciso XVI, alínea "c"; (Redação dada pela Emenda Constitucional nº 77, de 2014)

IX - aplica-se aos militares e a seus pensionistas o disposto no art. 40, §§ 7º e 8º; (Redação dada pela Emenda Constitucional nº 20, de 1998) (Revogado pela Emenda Constitucional nº 41, de 19.12.2003)

X - a lei disporá sobre o ingresso nas Forças Armadas, os limites de idade, a estabilidade e outras condições de transferência do militar para a inatividade, os direitos, os deveres, a remuneração, as prerrogativas e outras situações especiais dos militares, consideradas as peculiaridades de suas atividades, inclusive aquelas cumpridas por força de compromissos internacionais e de guerra. (Incluído pela Emenda Constitucional nº 18, de 1998)

Art. 143. O serviço militar é obrigatório nos termos da lei.

§ 1º Às Forças Armadas compete, na forma da lei, atribuir serviço alternativo aos que, em tempo de paz, após alistados, alegarem imperativo de consciência, entendendo-se como tal o decorrente de crença religiosa e de convicção filosófica ou política, para se eximirem de atividades de caráter essencialmente militar. (Regulamento)

§ 2º - As mulheres e os eclesiásticos ficam isentos do serviço militar obrigatório em tempo de paz, sujeitos, porém, a outros encargos que a lei lhes atribuir. (Regulamento)

CAPÍTULO III
DA SEGURANÇA PÚBLICA

Art. 144. A segurança pública, dever do Estado, direito e responsabilidade de todos, é exercida para a preservação da ordem pública e da incolumidade das pessoas e do patrimônio, através dos seguintes órgãos:

I - polícia federal;

II - polícia rodoviária federal;

III - polícia ferroviária federal;

IV - polícias civis;

V - polícias militares e corpos de bombeiros militares.

§ 1º A polícia federal, instituída por lei como órgão permanente, organizado e mantido pela União e estruturado em carreira, destina-se a: (Redação dada pela Emenda Constitucional nº 19, de 1998)

I - apurar infrações penais contra a ordem política e social ou em detrimento de bens, serviços e interesses da União ou de suas entidades autárquicas e empresas públicas, assim

como outras infrações cuja prática tenha repercussão interestadual ou internacional e exija repressão uniforme, segundo se dispuser em lei;

II - prevenir e reprimir o tráfico ilícito de entorpecentes e drogas afins, o contrabando e o descaminho, sem prejuízo da ação fazendária e de outros órgãos públicos nas respectivas áreas de competência;

III - exercer as funções de polícia marítima, aeroportuária e de fronteiras; (Redação dada pela Emenda Constitucional nº 19, de 1998)

IV - exercer, com exclusividade, as funções de polícia judiciária da União.

§ 2º A polícia rodoviária federal, órgão permanente, organizado e mantido pela União e estruturado em carreira, destina-se, na forma da lei, ao patrulhamento ostensivo das rodovias federais. (Redação dada pela Emenda Constitucional nº 19, de 1998)

§ 3º A polícia ferroviária federal, órgão permanente, organizado e mantido pela União e estruturado em carreira, destina-se, na forma da lei, ao patrulhamento ostensivo das ferrovias federais. (Redação dada pela Emenda Constitucional nº 19, de 1998)

§ 4º Às polícias civis, dirigidas por delegados de polícia de carreira, incumbem, ressalvada a competência da União, as funções de polícia judiciária e a apuração de infrações penais, exceto as militares.

§ 5º Às polícias militares cabem a polícia ostensiva e a preservação da ordem pública; aos corpos de bombeiros militares, além das atribuições definidas em lei, incumbe a execução de atividades de defesa civil.

§ 6º As polícias militares e corpos de bombeiros militares, forças auxiliares e reserva do Exército, subordinam-se, juntamente com as polícias civis, aos Governadores dos Estados, do Distrito Federal e dos Territórios.

§ 7º A lei disciplinará a organização e o funcionamento dos órgãos responsáveis pela segurança pública, de maneira a garantir a eficiência de suas atividades.

§ 8º Os Municípios poderão constituir guardas municipais destinadas à proteção de seus bens, serviços e instalações, conforme dispuser a lei.

§ 9º A remuneração dos servidores policiais integrantes dos órgãos relacionados neste artigo será fixada na forma do § 4º do art. 39. (Incluído pela Emenda Constitucional nº 19, de 1998)

§ 10. A segurança viária, exercida para a preservação da ordem pública e da incolumidade das pessoas e do seu patrimônio nas vias públicas: (Incluído pela Emenda Constitucional nº 82, de 2014)

I - compreende a educação, engenharia e fiscalização de trânsito, além de outras atividades previstas em lei, que assegurem ao cidadão o direito à mobilidade urbana eficiente; e (Incluído pela Emenda Constitucional nº 82, de 2014)

II - compete, no âmbito dos Estados, do Distrito Federal e dos Municípios, aos respectivos órgãos ou entidades executivos e seus agentes de trânsito, estruturados em Carreira, na forma da lei. (Incluído pela Emenda Constitucional nº 82, de 2014)

TÍTULO VI
DA TRIBUTAÇÃO E DO ORÇAMENTO
CAPÍTULO I
DO SISTEMA TRIBUTÁRIO NACIONAL

SEÇÃO I
DOS PRINCÍPIOS GERAIS

Art. 145. A União, os Estados, o Distrito Federal e os Municípios poderão instituir os seguintes tributos:

I - impostos;

II - taxas, em razão do exercício do poder de polícia ou pela utilização, efetiva ou potencial, de serviços públicos específicos e divisíveis, prestados ao contribuinte ou postos a sua disposição;

III - contribuição de melhoria, decorrente de obras públicas.

§ 1º Sempre que possível, os impostos terão caráter pessoal e serão graduados segundo a capacidade econômica do contribuinte, facultado à administração tributária, especialmente para conferir efetividade a esses objetivos, identificar, respeitados os direitos individuais e nos termos da lei, o patrimônio, os rendimentos e as atividades econômicas do contribuinte.

§ 2º As taxas não poderão ter base de cálculo própria de impostos.

Art. 146. Cabe à lei complementar:

I - dispor sobre conflitos de competência, em matéria tributária, entre a União, os Estados, o Distrito Federal e os Municípios;

II - regular as limitações constitucionais ao poder de tributar;

III - estabelecer normas gerais em matéria de legislação tributária, especialmente sobre:

a) definição de tributos e de suas espécies, bem como, em relação aos impostos discriminados nesta Constituição, a dos respectivos fatos geradores, bases de cálculo e contribuintes;

b) obrigação, lançamento, crédito, prescrição e decadência tributários;

c) adequado tratamento tributário ao ato cooperativo praticado pelas sociedades cooperativas.

d) definição de tratamento diferenciado e favorecido para as microempresas e para as empresas de pequeno porte, inclusive regimes especiais ou simplificados no caso do imposto previsto no art. 155, II, das contribuições previstas no art. 195, I e §§ 12 e 13, e da contribuição a que se refere o art. 239. (Incluído pela Emenda Constitucional nº 42, de 19.12.2003)

Parágrafo único. A lei complementar de que trata o inciso III, d, também poderá instituir um regime único de arrecadação dos impostos e contribuições da União, dos Estados, do Distrito Federal e dos Municípios, observado que: (Incluído pela Emenda Constitucional nº 42, de 19.12.2003)

I - será opcional para o contribuinte; (Incluído pela Emenda Constitucional nº 42, de 19.12.2003)

II - poderão ser estabelecidas condições de enquadramento diferenciadas por Estado; (Incluído pela Emenda Constitucional nº 42, de 19.12.2003)

III - o recolhimento será unificado e centralizado e a distribuição da parcela de recursos pertencentes aos respectivos entes federados será imediata, vedada qualquer retenção ou condicionamento; (Incluído pela Emenda Constitucional nº 42, de 19.12.2003)

IV - a arrecadação, a fiscalização e a cobrança poderão ser compartilhadas pelos entes federados, adotado cadastro nacional único de contribuintes. (Incluído pela Emenda Constitucional nº 42, de 19.12.2003)

Art. 146-A. Lei complementar poderá estabelecer critérios especiais de tributação, com o objetivo de prevenir desequilíbrios da concorrência, sem prejuízo da competência de a União, por lei, estabelecer normas de igual objetivo. (Incluído pela Emenda Constitucional nº 42, de 19.12.2003)

Art. 147. Competem à União, em Território Federal, os impostos estaduais e, se o Território não for dividido em Municípios, cumulativamente, os impostos municipais; ao Distrito Federal cabem os impostos municipais.

Art. 148. A União, mediante lei complementar, poderá instituir empréstimos compulsórios:

I - para atender a despesas extraordinárias, decorrentes de calamidade pública, de guerra externa ou sua iminência;

II - no caso de investimento público de caráter urgente e de relevante interesse nacional, observado o disposto no art. 150, III, "b".

Parágrafo único. A aplicação dos recursos provenientes de empréstimo compulsório será vinculada à despesa que fundamentou sua instituição.

Art. 149. Compete exclusivamente à União instituir contribuições sociais, de intervenção no domínio econômico e de interesse das categorias profissionais ou econômicas, como instrumento de sua atuação nas respectivas áreas, observado o disposto nos arts. 146, III, e 150, I e III, e sem prejuízo do previsto no art. 195, § 6º, relativamente às contribuições a que alude o dispositivo.

§ 1º Os Estados, o Distrito Federal e os Municípios instituirão contribuição, cobrada de seus servidores, para o custeio, em benefício destes, do regime previdenciário de que trata o art. 40, cuja alíquota não será inferior à da contribuição dos servidores titulares de cargos efetivos da União. (Redação dada pela Emenda Constitucional nº 41, 19.12.2003) (Vide ADIN 3133)

§ 2º As contribuições sociais e de intervenção no domínio econômico de que trata o *caput* deste artigo: (Incluído pela Emenda Constitucional nº 33, de 2001)

I - não incidirão sobre as receitas decorrentes de exportação; (Incluído pela Emenda Constitucional nº 33, de 2001)

II - incidirão também sobre a importação de produtos estrangeiros ou serviços; (Redação dada pela Emenda Constitucional nº 42, de 19.12.2003)

III - poderão ter alíquotas: (Incluído pela Emenda Constitucional nº 33, de 2001)

a) *ad valorem* , tendo por base o faturamento, a receita bruta ou o valor da operação e, no caso de importação, o valor aduaneiro; (Incluído pela Emenda Constitucional nº 33, de 2001)

b) específica, tendo por base a unidade de medida adotada. (Incluído pela Emenda Constitucional nº 33, de 2001)

§ 3º A pessoa natural destinatária das operações de importação poderá ser equiparada a pessoa jurídica, na forma da lei. (Incluído pela Emenda Constitucional nº 33, de 2001)

§ 4º A lei definirá as hipóteses em que as contribuições incidirão uma única vez. (Incluído pela Emenda Constitucional nº 33, de 2001)

Art. 149-A Os Municípios e o Distrito Federal poderão instituir contribuição, na forma das respectivas leis, para o custeio do serviço de iluminação pública, observado o disposto no art. 150, I e III. (Incluído pela Emenda Constitucional nº 39, de 2002)

Parágrafo único. É facultada a cobrança da contribuição a que se refere o caput, na fatura de consumo de energia elétrica. (Incluído pela Emenda Constitucional nº 39, de 2002)

SEÇÃO II
DAS LIMITAÇÕES DO PODER DE TRIBUTAR

Art. 150. Sem prejuízo de outras garantias asseguradas ao contribuinte, é vedado à União, aos Estados, ao Distrito Federal e aos Municípios:

I - exigir ou aumentar tributo sem lei que o estabeleça;

II - instituir tratamento desigual entre contribuintes que se encontrem em situação equivalente, proibida qualquer distinção em razão de ocupação profissional ou função por eles exercida, independentemente da denominação jurídica dos rendimentos, títulos ou direitos;

III - cobrar tributos:

a) em relação a fatos geradores ocorridos antes do início da vigência da lei que os houver instituído ou aumentado;

b) no mesmo exercício financeiro em que haja sido publicada a lei que os instituiu ou aumentou; (Vide Emenda Constitucional nº 3, de 1993)

c) antes de decorridos noventa dias da data em que haja sido publicada a lei que os instituiu ou aumentou, observado o disposto na alínea b; (Incluído pela Emenda Constitucional nº 42, de 19.12.2003)

IV - utilizar tributo com efeito de confisco;

V - estabelecer limitações ao tráfego de pessoas ou bens, por meio de tributos interestaduais ou intermunicipais, ressalvada a cobrança de pedágio pela utilização de vias conservadas pelo Poder Público;

VI - instituir impostos sobre: (Vide Emenda Constitucional nº 3, de 1993)

a) patrimônio, renda ou serviços, uns dos outros;

b) templos de qualquer culto;

c) patrimônio, renda ou serviços dos partidos políticos, inclusive suas fundações, das entidades sindicais dos trabalhadores, das instituições de educação e de assistência social, sem fins lucrativos, atendidos os requisitos da lei;

d) livros, jornais, periódicos e o papel destinado a sua impressão.

e) fonogramas e videofonogramas musicais produzidos no Brasil contendo obras musicais ou literomusicais de autores brasileiros e/ou obras em geral interpretadas por artistas brasileiros bem como os suportes materiais ou arquivos digitais que os contenham, salvo na etapa de replicação industrial de mídias ópticas de leitura a laser. (Incluída pela Emenda Constitucional nº 75, de 15.10.2013)

§ 1º A vedação do inciso III, *b,* não se aplica aos tributos previstos nos arts. 148, I, 153, I, II, IV e V; e 154, II; e a vedação do inciso III, *c,* não se aplica aos tributos previstos nos arts. 148, I, 153, I, II, III e V; e 154, II, nem à fixação da base de cálculo dos impostos previstos nos arts. 155, III, e 156, I. (Redação dada pela Emenda Constitucional nº 42, de 19.12.2003)

§ 2º - A vedação do inciso VI, "a", é extensiva às autarquias e às fundações instituídas e mantidas pelo Poder Público, no que se refere ao patrimônio, à renda e aos serviços, vinculados a suas finalidades essenciais ou às delas decorrentes.

§ 3º - As vedações do inciso VI, "a", e do parágrafo anterior não se aplicam ao patrimônio, à renda e aos serviços, relacionados com exploração de atividades econômicas regidas pelas normas aplicáveis a empreendimentos privados, ou em que haja contraprestação ou pagamento de preços ou tarifas pelo usuário, nem exonera o promitente comprador da obrigação de pagar imposto relativamente ao bem imóvel.

§ 4º - As vedações expressas no inciso VI, alíneas "b" e "c", compreendem somente o patrimônio, a renda e os serviços, relacionados com as finalidades essenciais das entidades nelas mencionadas.

§ 5º - A lei determinará medidas para que os consumidores sejam esclarecidos acerca dos impostos que incidam sobre mercadorias e serviços.

§ 6º Qualquer subsídio ou isenção, redução de base de cálculo, concessão de crédito presumido, anistia ou remissão, relativos a impostos, taxas ou contribuições, só poderá ser concedido mediante lei específica, federal, estadual ou municipal, que regule exclusivamente as matérias acima enumeradas ou o correspondente tributo ou contribuição, sem prejuízo do disposto no art. 155, § 2.º, XII, g. (Redação dada pela Emenda Constitucional nº 3, de 1993)

§ 7º A lei poderá atribuir a sujeito passivo de obrigação tributária a condição de responsável pelo pagamento de imposto ou contribuição, cujo fato gerador deva ocorrer posteriormente, assegurada a imediata e preferencial restituição da quantia paga, caso não se realize o fato gerador presumido. (Incluído pela Emenda Constitucional nº 3, de 1993)

Art. 151. É vedado à União:

I - instituir tributo que não seja uniforme em todo o território nacional ou que implique distinção ou preferência em relação a Estado, ao Distrito Federal ou a Município, em detrimento de outro, admitida a concessão de incentivos fiscais destinados a promover o equilíbrio do desenvolvimento sócio-econômico entre as diferentes regiões do País;

II - tributar a renda das obrigações da dívida pública dos Estados, do Distrito Federal e dos Municípios, bem como a remuneração e os proventos dos respectivos agentes públicos, em níveis superiores aos que fixar para suas obrigações e para seus agentes;

III - instituir isenções de tributos da competência dos Estados, do Distrito Federal ou dos Municípios.

Art. 152. É vedado aos Estados, ao Distrito Federal e aos Municípios estabelecer diferença tributária entre bens e serviços, de qualquer natureza, em razão de sua procedência ou destino.

SEÇÃO III
DOS IMPOSTOS DA UNIÃO

Art. 153. Compete à União instituir impostos sobre:

I - importação de produtos estrangeiros;

II - exportação, para o exterior, de produtos nacionais ou nacionalizados;

III - renda e proventos de qualquer natureza;

IV - produtos industrializados;

V - operações de crédito, câmbio e seguro, ou relativas a títulos ou valores mobiliários;

VI - propriedade territorial rural;

VII - grandes fortunas, nos termos de lei complementar.

§ 1º É facultado ao Poder Executivo, atendidas as condições e os limites estabelecidos em lei, alterar as alíquotas dos impostos enumerados nos incisos I, II, IV e V.

§ 2º O imposto previsto no inciso III:

I - será informado pelos critérios da generalidade, da universalidade e da progressividade, na forma da lei;

II - não incidirá, nos termos e limites fixados em lei, sobre rendimentos provenientes de aposentadoria e pensão, pagos pela previdência social da União, dos Estados, do Distrito Federal e dos Municípios, a pessoa com idade superior a sessenta e cinco anos, cuja renda total seja constituída, exclusivamente, de rendimentos do trabalho . (Revogado pela Emenda Constitucional nº 20, de 1998)

§ 3º O imposto previsto no inciso IV:

I - será seletivo, em função da essencialidade do produto;

II - será não-cumulativo, compensando-se o que for devido em cada operação com o montante cobrado nas anteriores;

III - não incidirá sobre produtos industrializados destinados ao exterior.

IV - terá reduzido seu impacto sobre a aquisição de bens de capital pelo contribuinte do imposto, na forma da lei. (Incluído pela Emenda Constitucional nº 42, de 19.12.2003)

§ 4º O imposto previsto no inciso VI do *capu* t: (Redação dada pela Emenda Constitucional nº 42, de 19.12.2003)

I - será progressivo e terá suas alíquotas fixadas de forma a desestimular a manutenção de propriedades improdutivas; (Incluído pela Emenda Constitucional nº 42, de 19.12.2003)

II - não incidirá sobre pequenas glebas rurais, definidas em lei, quando as explore o proprietário que não possua outro imóvel; (Incluído pela Emenda Constitucional nº 42, de 19.12.2003)

III - será fiscalizado e cobrado pelos Municípios que assim optarem, na forma da lei, desde que não implique redução do imposto ou qualquer outra forma de renúncia fiscal. (Incluído pela Emenda Constitucional nº 42, de 19.12.2003) (Regulamento)

§ 5º O ouro, quando definido em lei como ativo financeiro ou instrumento cambial, sujeita-se exclusivamente à incidência do imposto de que trata o inciso V do "caput" deste artigo, devido na operação de origem; a alíquota mínima será de um por cento, assegurada a transferência do montante da arrecadação nos seguintes termos: (Vide Emenda Constitucional nº 3, de 1993)

I - trinta por cento para o Estado, o Distrito Federal ou o Território, conforme a origem;

II - setenta por cento para o Município de origem.

Art. 154. A União poderá instituir:

I - mediante lei complementar, impostos não previstos no artigo anterior, desde que sejam não-cumulativos e não tenham fato gerador ou base de cálculo próprios dos discriminados nesta Constituição;

II - na iminência ou no caso de guerra externa, impostos extraordinários, compreendidos ou não em sua competência tributária, os quais serão suprimidos, gradativamente, cessadas as causas de sua criação.

SEÇÃO IV
DOS IMPOSTOS DOS ESTADOS E DO DISTRITO FEDERAL

Art. 155. Compete aos Estados e ao Distrito Federal instituir impostos sobre: (Redação dada pela Emenda Constitucional nº 3, de 1993)

I - transmissão causa mortis e doação, de quaisquer bens ou direitos; (Redação dada pela Emenda Constitucional nº 3, de 1993)

II - operações relativas à circulação de mercadorias e sobre prestações de serviços de transporte interestadual e intermunicipal e de comunicação, ainda que as operações e as prestações se iniciem no exterior; (Redação dada pela Emenda Constitucional nº 3, de 1993)

III - propriedade de veículos automotores. (Redação dada pela Emenda Constitucional nº 3, de 1993)

§ 1º O imposto previsto no inciso I: (Redação dada pela Emenda Constitucional nº 3, de 1993)

I - relativamente a bens imóveis e respectivos direitos, compete ao Estado da situação do bem, ou ao Distrito Federal

II - relativamente a bens móveis, títulos e créditos, compete ao Estado onde se processar o inventário ou arrolamento, ou tiver domicílio o doador, ou ao Distrito Federal;

III - terá competência para sua instituição regulada por lei complementar:

a) se o doador tiver domicilio ou residência no exterior;

b) se o de cujus possuía bens, era residente ou domiciliado ou teve o seu inventário processado no exterior;

IV - terá suas alíquotas máximas fixadas pelo Senado Federal;

§ 2º ~~O imposto previsto no inciso I, b, atenderá ao seguinte:~~

§ 2º O imposto previsto no inciso II atenderá ao seguinte: (Redação dada pela Emenda Constitucional nº 3, de 1993)

I - será não-cumulativo, compensando-se o que for devido em cada operação relativa à circulação de mercadorias ou prestação de serviços com o montante cobrado nas anteriores pelo mesmo ou outro Estado ou pelo Distrito Federal;

II - a isenção ou não-incidência, salvo determinação em contrário da legislação:

a) não implicará crédito para compensação com o montante devido nas operações ou prestações seguintes;

b) acarretará a anulação do crédito relativo às operações anteriores;

III - poderá ser seletivo, em função da essencialidade das mercadorias e dos serviços;

IV - resolução do Senado Federal, de iniciativa do Presidente da República ou de um terço dos Senadores, aprovada pela maioria absoluta de seus membros, estabelecerá as alíquotas aplicáveis às operações e prestações, interestaduais e de exportação;

V - é facultado ao Senado Federal:

a) estabelecer alíquotas mínimas nas operações internas, mediante resolução de iniciativa de um terço e aprovada pela maioria absoluta de seus membros;

b) fixar alíquotas máximas nas mesmas operações para resolver conflito específico que envolva interesse de Estados, mediante resolução de iniciativa da maioria absoluta e aprovada por dois terços de seus membros;

VI - salvo deliberação em contrário dos Estados e do Distrito Federal, nos termos do disposto no inciso XII, "g", as alíquotas internas, nas operações relativas à circulação de mercadorias e nas prestações de serviços, não poderão ser inferiores às previstas para as operações interestaduais;

VII - nas operações e prestações que destinem bens e serviços a consumidor final, contribuinte ou não do imposto, localizado em outro Estado, adotar-se-á a alíquota interestadual e caberá ao Estado de localização do destinatário o imposto correspondente à diferença entre a alíquota interna do Estado destinatário e a alíquota interestadual; (Redação dada pela Emenda Constitucional nº 87, de 2015) (Produção de efeito)

a) (revogada); (Redação dada pela Emenda Constitucional nº 87, de 2015)

b) (revogada); (Redação dada pela Emenda Constitucional nº 87, de 2015)

VIII - a responsabilidade pelo recolhimento do imposto correspondente à diferença entre a alíquota interna e a interestadual de que trata o inciso VII será atribuída: (Redação dada pela Emenda Constitucional nº 87, de 2015) (Produção de efeito)

a) ao destinatário, quando este for contribuinte do imposto; (Incluído pela Emenda Constitucional nº 87, de 2015)

b) ao remetente, quando o destinatário não for contribuinte do imposto; (Incluído pela Emenda Constitucional nº 87, de 2015)

IX - incidirá também:

a) sobre a entrada de bem ou mercadoria importados do exterior por pessoa física ou jurídica, ainda que não seja contribuinte habitual do imposto, qualquer que seja a sua finalidade, assim

como sobre o serviço prestado no exterior, cabendo o imposto ao Estado onde estiver situado o domicílio ou o estabelecimento do destinatário da mercadoria, bem ou serviço; (Redação dada pela Emenda Constitucional nº 33, de 2001)

b) sobre o valor total da operação, quando mercadorias forem fornecidas com serviços não compreendidos na competência tributária dos Municípios;

X - não incidirá:

a) sobre operações que destinem mercadorias para o exterior, nem sobre serviços prestados a destinatários no exterior, assegurada a manutenção e o aproveitamento do montante do imposto cobrado nas operações e prestações anteriores; (Redação dada pela Emenda Constitucional nº 42, de 19.12.2003)

b) sobre operações que destinem a outros Estados petróleo, inclusive lubrificantes, combustíveis líquidos e gasosos dele derivados, e energia elétrica;

c) sobre o ouro, nas hipóteses definidas no art. 153, § 5º;

d) nas prestações de serviço de comunicação nas modalidades de radiodifusão sonora e de sons e imagens de recepção livre e gratuita; (Incluído pela Emenda Constitucional nº 42, de 19.12.2003)

XI - não compreenderá, em sua base de cálculo, o montante do imposto sobre produtos industrializados, quando a operação, realizada entre contribuintes e relativa a produto destinado à industrialização ou à comercialização, configure fato gerador dos dois impostos;

XII - cabe à lei complementar:

a) definir seus contribuintes;

b) dispor sobre substituição tributária;

c) disciplinar o regime de compensação do imposto;

d) fixar, para efeito de sua cobrança e definição do estabelecimento responsável, o local das operações relativas à circulação de mercadorias e das prestações de serviços;

e) excluir da incidência do imposto, nas exportações para o exterior, serviços e outros produtos além dos mencionados no inciso X, "a"

f) prever casos de manutenção de crédito, relativamente à remessa para outro Estado e exportação para o exterior, de serviços e de mercadorias;

g) regular a forma como, mediante deliberação dos Estados e do Distrito Federal, isenções, incentivos e benefícios fiscais serão concedidos e revogados.

h) definir os combustíveis e lubrificantes sobre os quais o imposto incidirá uma única vez, qualquer que seja a sua finalidade, hipótese em que não se aplicará o disposto no inciso X, *b* ; (Incluída pela Emenda Constitucional nº 33, de 2001) (Vide Emenda Constitucional nº 33, de 2001)

i) fixar a base de cálculo, de modo que o montante do imposto a integre, também na importação do exterior de bem, mercadoria ou serviço. (Incluída pela Emenda Constitucional nº 33, de 2001)

§ 3º À exceção dos impostos de que tratam o inciso II do *caput* deste artigo e o art. 153, I e II, nenhum outro imposto poderá incidir sobre operações relativas a energia elétrica, serviços de telecomunicações, derivados de petróleo, combustíveis e minerais do País. (Redação dada pela Emenda Constitucional nº 33, de 2001)

§ 4º Na hipótese do inciso XII, *h* , observar-se-á o seguinte: (Incluído pela Emenda Constitucional nº 33, de 2001)

I - nas operações com os lubrificantes e combustíveis derivados de petróleo, o imposto caberá ao Estado onde ocorrer o consumo; (Incluído pela Emenda Constitucional nº 33, de 2001)

II - nas operações interestaduais, entre contribuintes, com gás natural e seus derivados, e lubrificantes e combustíveis não incluídos no inciso I deste parágrafo, o imposto será repartido entre os Estados de origem e de destino, mantendo-se a mesma proporcionalidade que ocorre nas operações com as demais mercadorias; (Incluído pela Emenda Constitucional nº 33, de 2001)

III - nas operações interestaduais com gás natural e seus derivados, e lubrificantes e combustíveis não incluídos no inciso I deste parágrafo, destinadas a não contribuinte, o imposto caberá ao Estado de origem; (Incluído pela Emenda Constitucional nº 33, de 2001)

IV - as alíquotas do imposto serão definidas mediante deliberação dos Estados e Distrito Federal, nos termos do § 2º, XII, *g* , observando-se o seguinte: (Incluído pela Emenda Constitucional nº 33, de 2001)

a) serão uniformes em todo o território nacional, podendo ser diferenciadas por produto; (Incluído pela Emenda Constitucional nº 33, de 2001)

b) poderão ser específicas, por unidade de medida adotada, ou *ad valorem* , incidindo sobre o valor da operação ou sobre o preço que o produto ou seu similar alcançaria em uma venda em condições de livre concorrência; (Incluído pela Emenda Constitucional nº 33, de 2001)

c) poderão ser reduzidas e restabelecidas, não se lhes aplicando o disposto no art. 150, III, *b* . (Incluído pela Emenda Constitucional nº 33, de 2001)

§ 5º As regras necessárias à aplicação do disposto no § 4º, inclusive as relativas à apuração e à destinação do imposto, serão estabelecidas mediante deliberação dos Estados e do Distrito Federal, nos termos do § 2º, XII, *g* . (Incluído pela Emenda Constitucional nº 33, de 2001)

§ 6º O imposto previsto no inciso III: (Incluído pela Emenda Constitucional nº 42, de 19.12.2003)

I - terá alíquotas mínimas fixadas pelo Senado Federal; (Incluído pela Emenda Constitucional nº 42, de 19.12.2003)

II - poderá ter alíquotas diferenciadas em função do tipo e utilização. (Incluído pela Emenda Constitucional nº 42, de 19.12.2003)

SEÇÃO V
DOS IMPOSTOS DOS MUNICÍPIOS

Art. 156. Compete aos Municípios instituir impostos sobre:

I - propriedade predial e territorial urbana;

II - transmissão "inter vivos", a qualquer título, por ato oneroso, de bens imóveis, por natureza ou acessão física, e de direitos reais sobre imóveis, exceto os de garantia, bem como cessão de direitos a sua aquisição;

III - serviços de qualquer natureza, não compreendidos no art. 155, II, definidos em lei complementar. (Redação dada pela Emenda Constitucional nº 3, de 1993)

IV - serviços de qualquer natureza, não compreendidos no art. 155, I, b, definidos em lei complementar. (Revogado pela Emenda Constitucional nº 3, de 1993)

§ 1º Sem prejuízo da progressividade no tempo a que se refere o art. 182, § 4º, inciso II, o imposto previsto no inciso I poderá: (Redação dada pela Emenda Constitucional nº 29, de 2000)

I - ser progressivo em razão do valor do imóvel; e (Incluído pela Emenda Constitucional nº 29, de 2000)

II - ter alíquotas diferentes de acordo com a localização e o uso do imóvel. (Incluído pela Emenda Constitucional nº 29, de 2000)

§ 2º O imposto previsto no inciso II:

I - não incide sobre a transmissão de bens ou direitos incorporados ao patrimônio de pessoa jurídica em realização de capital, nem sobre a transmissão de bens ou direitos decorrente de fusão, incorporação, cisão ou extinção de pessoa jurídica, salvo se, nesses casos, a atividade preponderante do adquirente for a compra e venda desses bens ou direitos, locação de bens imóveis ou arrendamento mercantil;

II - compete ao Município da situação do bem.

§ 3º Em relação ao imposto previsto no inciso III do *caput* deste artigo, cabe à lei complementar: (Redação dada pela Emenda Constitucional nº 37, de 2002)

I - fixar as suas alíquotas máximas e mínimas; (Redação dada pela Emenda Constitucional nº 37, de 2002)

II - excluir da sua incidência exportações de serviços para o exterior. (Incluído pela Emenda Constitucional nº 3, de 1993)

III - regular a forma e as condições como isenções, incentivos e benefícios fiscais serão concedidos e revogados. (Incluído pela Emenda Constitucional nº 3, de 1993)

SEÇÃO VI
DA REPARTIÇÃO DAS RECEITAS TRIBUTÁRIAS

Art. 157. Pertencem aos Estados e ao Distrito Federal:

I - o produto da arrecadação do imposto da União sobre renda e proventos de qualquer natureza, incidente na fonte, sobre rendimentos pagos, a qualquer título, por eles, suas autarquias e pelas fundações que instituírem e mantiverem;

II - vinte por cento do produto da arrecadação do imposto que a União instituir no exercício da competência que lhe é atribuída pelo art. 154, I.

Art. 158. Pertencem aos Municípios:

I - o produto da arrecadação do imposto da União sobre renda e proventos de qualquer natureza, incidente na fonte, sobre rendimentos pagos, a qualquer título, por eles, suas autarquias e pelas fundações que instituírem e mantiverem;

II - cinqüenta por cento do produto da arrecadação do imposto da União sobre a propriedade territorial rural, relativamente aos imóveis neles situados, cabendo a totalidade na hipótese da opção a que se refere o art. 153, § 4º, III; (Redação dada pela Emenda Constitucional nº 42, de 19.12.2003) (Regulamento)

III - cinqüenta por cento do produto da arrecadação do imposto do Estado sobre a propriedade de veículos automotores licenciados em seus territórios;

IV - vinte e cinco por cento do produto da arrecadação do imposto do Estado sobre operações relativas à circulação de mercadorias e sobre prestações de serviços de transporte interestadual e intermunicipal e de comunicação.

Parágrafo único. As parcelas de receita pertencentes aos Municípios, mencionadas no inciso IV, serão creditadas conforme os seguintes critérios:

I - três quartos, no mínimo, na proporção do valor adicionado nas operações relativas à circulação de mercadorias e nas prestações de serviços, realizadas em seus territórios;

II - até um quarto, de acordo com o que dispuser lei estadual ou, no caso dos Territórios, lei federal.

Art. 159. A União entregará: (Vide Emenda Constitucional nº 55, de 2007)

I - do produto da arrecadação dos impostos sobre renda e proventos de qualquer natureza e sobre produtos industrializados, 49% (quarenta e nove por cento), na seguinte forma: (Redação dada pela Emenda Constitucional nº 84, de 2014)

a) vinte e um inteiros e cinco décimos por cento ao Fundo de Participação dos Estados e do Distrito Federal; (Vide Lei Complementar nº 62, de 1989) (Regulamento)

b) vinte e dois inteiros e cinco décimos por cento ao Fundo de Participação dos Municípios; (Vide Lei Complementar nº 62, de 1989) (Regulamento)

c) três por cento, para aplicação em programas de financiamento ao setor produtivo das Regiões Norte, Nordeste e Centro-Oeste, através de suas instituições financeiras de caráter regional, de acordo com os planos regionais de desenvolvimento, ficando assegurada ao semi-árido do Nordeste a metade dos recursos destinados à Região, na forma que a lei estabelecer; (Regulamento)

d) um por cento ao Fundo de Participação dos Municípios, que será entregue no primeiro decêndio do mês de dezembro de cada ano; (Incluído pela Emenda Constitucional nº 55, de 2007)

e) 1% (um por cento) ao Fundo de Participação dos Municípios, que será entregue no primeiro decêndio do mês de julho de cada ano; (Incluída pela Emenda Constitucional nº 84, de 2014)

II - do produto da arrecadação do imposto sobre produtos industrializados, dez por cento aos Estados e ao Distrito Federal, proporcionalmente ao valor das respectivas exportações de produtos industrializados. (Regulamento)

III - do produto da arrecadação da contribuição de intervenção no domínio econômico prevista no art. 177, § 4º, 29% (vinte e nove por cento) para os Estados e o Distrito Federal,

distribuídos na forma da lei, observada a destinação a que se refere o inciso II, *c* , do referido parágrafo. (Redação dada pela Emenda Constitucional nº 44, de 2004)

§ 1º Para efeito de cálculo da entrega a ser efetuada de acordo com o previsto no inciso I, excluir-se-á a parcela da arrecadação do imposto de renda e proventos de qualquer natureza pertencente aos Estados, ao Distrito Federal e aos Municípios, nos termos do disposto nos arts. 157, I, e 158, I.

§ 2º A nenhuma unidade federada poderá ser destinada parcela superior a vinte por cento do montante a que se refere o inciso II, devendo o eventual excedente ser distribuído entre os demais participantes, mantido, em relação a esses, o critério de partilha nele estabelecido.

§ 3º Os Estados entregarão aos respectivos Municípios vinte e cinco por cento dos recursos que receberem nos termos do inciso II, observados os critérios estabelecidos no art. 158, parágrafo único, I e II.

§ 4º Do montante de recursos de que trata o inciso III que cabe a cada Estado, vinte e cinco por cento serão destinados aos seus Municípios, na forma da lei a que se refere o mencionado inciso. (Incluído pela Emenda Constitucional nº 42, de 19.12.2003)

Art. 160. É vedada a retenção ou qualquer restrição à entrega e ao emprego dos recursos atribuídos, nesta seção, aos Estados, ao Distrito Federal e aos Municípios, neles compreendidos adicionais e acréscimos relativos a impostos.

Parágrafo único. A vedação prevista neste artigo não impede a União e os Estados de condicionarem a entrega de recursos: (Redação dada pela Emenda Constitucional nº 29, de 2000)

I - ao pagamento de seus créditos, inclusive de suas autarquias; (Incluído pela Emenda Constitucional nº 29, de 2000)

II - ao cumprimento do disposto no art. 198, § 2º, incisos II e III. (Incluído pela Emenda Constitucional nº 29, de 2000)

Art. 161. Cabe à lei complementar:

I - definir valor adicionado para fins do disposto no art. 158, parágrafo único, I;

II - estabelecer normas sobre a entrega dos recursos de que trata o art. 159, especialmente sobre os critérios de rateio dos fundos previstos em seu inciso I, objetivando promover o equilíbrio sócio-econômico entre Estados e entre Municípios;

III - dispor sobre o acompanhamento, pelos beneficiários, do cálculo das quotas e da liberação das participações previstas nos arts. 157, 158 e 159.

Parágrafo único. O Tribunal de Contas da União efetuará o cálculo das quotas referentes aos fundos de participação a que alude o inciso II.

Art. 162. A União, os Estados, o Distrito Federal e os Municípios divulgarão, até o último dia do mês subseqüente ao da arrecadação, os montantes de cada um dos tributos arrecadados, os recursos recebidos, os valores de origem tributária entregues e a entregar e a expressão numérica dos critérios de rateio.

Parágrafo único. Os dados divulgados pela União serão discriminados por Estado e por Município; os dos Estados, por Município.

CAPÍTULO II

DAS FINANÇAS PÚBLICAS

SEÇÃO I
NORMAS GERAIS

Art. 163. Lei complementar disporá sobre:

I - finanças públicas;

II - dívida pública externa e interna, incluída a das autarquias, fundações e demais entidades controladas pelo Poder Público;

III - concessão de garantias pelas entidades públicas;

IV - emissão e resgate de títulos da dívida pública;

~~V - fiscalização das instituições financeiras;~~

V - fiscalização financeira da administração pública direta e indireta; (Redação dada pela Emenda Constitucional nº 40, de 2003)

VI - operações de câmbio realizadas por órgãos e entidades da União, dos Estados, do Distrito Federal e dos Municípios;

VII - compatibilização das funções das instituições oficiais de crédito da União, resguardadas as características e condições operacionais plenas das voltadas ao desenvolvimento regional.

Art. 164. A competência da União para emitir moeda será exercida exclusivamente pelo banco central.

§ 1º É vedado ao banco central conceder, direta ou indiretamente, empréstimos ao Tesouro Nacional e a qualquer órgão ou entidade que não seja instituição financeira.

§ 2º O banco central poderá comprar e vender títulos de emissão do Tesouro Nacional, com o objetivo de regular a oferta de moeda ou a taxa de juros.

§ 3º As disponibilidades de caixa da União serão depositadas no banco central; as dos Estados, do Distrito Federal, dos Municípios e dos órgãos ou entidades do Poder Público e das empresas por ele controladas, em instituições financeiras oficiais, ressalvados os casos previstos em lei.

SEÇÃO II
DOS ORÇAMENTOS

Art. 165. Leis de iniciativa do Poder Executivo estabelecerão:

I - o plano plurianual;

II - as diretrizes orçamentárias;

III - os orçamentos anuais.

§ 1º A lei que instituir o plano plurianual estabelecerá, de forma regionalizada, as diretrizes, objetivos e metas da administração pública federal para as despesas de capital e outras delas decorrentes e para as relativas aos programas de duração continuada.

§ 2º A lei de diretrizes orçamentárias compreenderá as metas e prioridades da administração pública federal, incluindo as despesas de capital para o exercício financeiro subseqüente, orientará a elaboração da lei orçamentária anual, disporá sobre as alterações na legislação tributária e estabelecerá a política de aplicação das agências financeiras oficiais de fomento.

§ 4º Os planos e programas nacionais, regionais e setoriais previstos nesta Constituição serão elaborados em consonância com o plano plurianual e apreciados pelo Congresso Nacional.

§ 5º A lei orçamentária anual compreenderá:

I - o orçamento fiscal referente aos Poderes da União, seus fundos, órgãos e entidades da administração direta e indireta, inclusive fundações instituídas e mantidas pelo Poder Público;

II - o orçamento de investimento das empresas em que a União, direta ou indiretamente, detenha a maioria do capital social com direito a voto;

III - o orçamento da seguridade social, abrangendo todas as entidades e órgãos a ela vinculados, da administração direta ou indireta, bem como os fundos e fundações instituídos e mantidos pelo Poder Público.

§ 6º O projeto de lei orçamentária será acompanhado de demonstrativo regionalizado do efeito, sobre as receitas e despesas, decorrente de isenções, anistias, remissões, subsídios e benefícios de natureza financeira, tributária e creditícia.

§ 7º Os orçamentos previstos no § 5º, I e II, deste artigo, compatibilizados com o plano plurianual, terão entre suas funções a de reduzir desigualdades inter-regionais, segundo critério populacional.

§ 8º A lei orçamentária anual não conterá dispositivo estranho à previsão da receita e à fixação da despesa, não se incluindo na proibição a autorização para abertura de créditos suplementares e contratação de operações de crédito, ainda que por antecipação de receita, nos termos da lei.

§ 9º Cabe à lei complementar:

I - dispor sobre o exercício financeiro, a vigência, os prazos, a elaboração e a organização do plano plurianual, da lei de diretrizes orçamentárias e da lei orçamentária anual;

II - estabelecer normas de gestão financeira e patrimonial da administração direta e indireta bem como condições para a instituição e funcionamento de fundos.

III - dispor sobre critérios para a execução equitativa, além de procedimentos que serão adotados quando houver impedimentos legais e técnicos, cumprimento de restos a pagar e limitação das programações de caráter obrigatório, para a realização do disposto no § 11 do art. 166. (Incluído pela Emenda Constitucional nº 86, de 2015)

I - subordina-se ao cumprimento de dispositivos constitucionais e legais que estabeleçam metas fiscais ou limites de despesas e não impede o cancelamento necessário à abertura de créditos adicionais;

II - não se aplica nos casos de impedimentos de ordem técnica devidamente justificados;

III - aplica-se exclusivamente às despesas primárias discricionárias.

Art. 166. Os projetos de lei relativos ao plano plurianual, às diretrizes orçamentárias, ao orçamento anual e aos créditos adicionais serão apreciados pelas duas Casas do Congresso Nacional, na forma do regimento comum.

§ 1º Caberá a uma Comissão mista permanente de Senadores e Deputados:

I - examinar e emitir parecer sobre os projetos referidos neste artigo e sobre as contas apresentadas anualmente pelo Presidente da República;

II - examinar e emitir parecer sobre os planos e programas nacionais, regionais e setoriais previstos nesta Constituição e exercer o acompanhamento e a fiscalização orçamentária, sem prejuízo da atuação das demais comissões do Congresso Nacional e de suas Casas, criadas de acordo com o art. 58.

§ 2º As emendas serão apresentadas na Comissão mista, que sobre elas emitirá parecer, e apreciadas, na forma regimental, pelo Plenário das duas Casas do Congresso Nacional.

§ 3º As emendas ao projeto de lei do orçamento anual ou aos projetos que o modifiquem somente podem ser aprovadas caso:

I - sejam compatíveis com o plano plurianual e com a lei de diretrizes orçamentárias;

II - indiquem os recursos necessários, admitidos apenas os provenientes de anulação de despesa, excluídas as que incidam sobre:

a) dotações para pessoal e seus encargos;

b) serviço da dívida;

c) transferências tributárias constitucionais para Estados, Municípios e Distrito Federal; ou

III - sejam relacionadas:

a) com a correção de erros ou omissões; ou

b) com os dispositivos do texto do projeto de lei.

§ 4º As emendas ao projeto de lei de diretrizes orçamentárias não poderão ser aprovadas quando incompatíveis com o plano plurianual.

§ 5º O Presidente da República poderá enviar mensagem ao Congresso Nacional para propor modificação nos projetos a que se refere este artigo enquanto não iniciada a votação, na Comissão mista, da parte cuja alteração é proposta.

§ 6º Os projetos de lei do plano plurianual, das diretrizes orçamentárias e do orçamento anual serão enviados pelo Presidente da República ao Congresso Nacional, nos termos da lei complementar a que se refere o art. 165, § 9º.

§ 7º Aplicam-se aos projetos mencionados neste artigo, no que não contrariar o disposto nesta seção, as demais normas relativas ao processo legislativo.

§ 8º Os recursos que, em decorrência de veto, emenda ou rejeição do projeto de lei orçamentária anual, ficarem sem despesas correspondentes poderão ser utilizados, conforme o caso, mediante créditos especiais ou suplementares, com prévia e específica autorização legislativa.

§ 9º As emendas individuais ao projeto de lei orçamentária serão aprovadas no limite de 1,2% (um inteiro e dois décimos por cento) da receita corrente líquida prevista no projeto encaminhado pelo Poder Executivo, sendo que a metade deste percentual será destinada a ações e serviços públicos de saúde. (Incluído pela Emenda Constitucional nº 86, de 2015)

§ 10. A execução do montante destinado a ações e serviços públicos de saúde previsto no § 9º, inclusive custeio, será computada para fins do cumprimento do inciso I do § 2º do art. 198, vedada a destinação para pagamento de pessoal ou encargos sociais. (Incluído pela Emenda Constitucional nº 86, de 2015)

§ 11. É obrigatória a execução orçamentária e financeira das programações a que se refere o § 9º deste artigo, em montante correspondente a 1,2% (um inteiro e dois décimos por cento) da receita corrente líquida realizada no exercício anterior, conforme os critérios para a execução equitativa da programação definidos na lei complementar prevista no § 9º do art. 165. (Incluído pela Emenda Constitucional nº 86, de 2015)

~~§ 12. As programações orçamentárias previstas no § 9º deste artigo não serão de execução obrigatória nos casos dos impedimentos de ordem técnica. (Incluído pela Emenda Constitucional nº 86, de 2015)~~

§ 13. Quando a transferência obrigatória da União, para a execução da programação prevista no §11 deste artigo, for destinada a Estados, ao Distrito Federal e a Municípios, independerá da adimplência do ente federativo destinatário e não integrará a base de cálculo da receita corrente líquida para fins de aplicação dos limites de despesa de pessoal de que trata o caput do art. 169. (Incluído pela Emenda Constitucional nº 86, de 2015)

§ 14. No caso de impedimento de ordem técnica, no empenho de despesa que integre a programação, na forma do § 11 deste artigo, serão adotadas as seguintes medidas: (Incluído pela Emenda Constitucional nº 86, de 2015)

I - até 120 (cento e vinte) dias após a publicação da lei orçamentária, o Poder Executivo, o Poder Legislativo, o Poder Judiciário, o Ministério Público e a Defensoria Pública enviarão ao Poder Legislativo as justificativas do impedimento; (Incluído pela Emenda Constitucional nº 86, de 2015)

II - até 30 (trinta) dias após o término do prazo previsto no inciso I, o Poder Legislativo indicará ao Poder Executivo o remanejamento da programação cujo impedimento seja insuperável; (Incluído pela Emenda Constitucional nº 86, de 2015)

III - até 30 de setembro ou até 30 (trinta) dias após o prazo previsto no inciso II, o Poder Executivo encaminhará projeto de lei sobre o remanejamento da programação cujo impedimento seja insuperável; (Incluído pela Emenda Constitucional nº 86, de 2015)

IV - se, até 20 de novembro ou até 30 (trinta) dias após o término do prazo previsto no inciso III, o Congresso Nacional não deliberar sobre o projeto, o remanejamento será implementado por ato do Poder Executivo, nos termos previstos na lei orçamentária.

§ 15. Após o prazo previsto no inciso IV do § 14, as programações orçamentárias previstas no § 11 não serão de execução obrigatória nos casos dos impedimentos justificados na notificação prevista no inciso I do § 14. (Incluído pela Emenda Constitucional nº 86, de 2015)

§ 16. Os restos a pagar poderão ser considerados para fins de cumprimento da execução financeira prevista no § 11 deste artigo, até o limite de 0,6% (seis décimos por cento) da receita corrente líquida realizada no exercício anterior. (Incluído pela Emenda Constitucional nº 86, de 2015)

§ 17. Se for verificado que a reestimativa da receita e da despesa poderá resultar no não cumprimento da meta de resultado fiscal estabelecida na lei de diretrizes orçamentárias, o montante previsto no § 11 deste artigo poderá ser reduzido em até a mesma proporção da limitação incidente sobre o conjunto das despesas discricionárias. (Incluído pela Emenda Constitucional nº 86, de 2015)

§ 18. Considera-se equitativa a execução das programações de caráter obrigatório que atenda de forma igualitária e impessoal às emendas apresentadas, independentemente da autoria. (Incluído pela Emenda Constitucional nº 86, de 2015)

Art. 167. São vedados:

I - o início de programas ou projetos não incluídos na lei orçamentária anual;

II - a realização de despesas ou a assunção de obrigações diretas que excedam os créditos orçamentários ou adicionais;

IV - a vinculação de receita de impostos a órgão, fundo ou despesa, ressalvadas a repartição do produto da arrecadação dos impostos a que se referem os arts. 158 e 159, a destinação de recursos para as ações e serviços públicos de saúde, para manutenção e desenvolvimento do ensino e para realização de atividades da administração tributária, como determinado, respectivamente, pelos arts. 198, § 2º, 212 e 37, XXII, e a prestação de garantias às operações de crédito por antecipação de receita, previstas no art. 165, § 8º, bem como o disposto no § 4º deste artigo; (Redação dada pela Emenda Constitucional nº 42, de 19.12.2003)

V - a abertura de crédito suplementar ou especial sem prévia autorização legislativa e sem indicação dos recursos correspondentes;

VI - a transposição, o remanejamento ou a transferência de recursos de uma categoria de programação para outra ou de um órgão para outro, sem prévia autorização legislativa;

VII - a concessão ou utilização de créditos ilimitados;

VIII - a utilização, sem autorização legislativa específica, de recursos dos orçamentos fiscal e da seguridade social para suprir necessidade ou cobrir déficit de empresas, fundações e fundos, inclusive dos mencionados no art. 165, § 5º;

IX - a instituição de fundos de qualquer natureza, sem prévia autorização legislativa.

X - a transferência voluntária de recursos e a concessão de empréstimos, inclusive por antecipação de receita, pelos Governos Federal e Estaduais e suas instituições financeiras, para pagamento de despesas com pessoal ativo, inativo e pensionista, dos Estados, do Distrito Federal e dos Municípios. (Incluído pela Emenda Constitucional nº 19, de 1998)

XI - a utilização dos recursos provenientes das contribuições sociais de que trata o art. 195, I, a, e II, para a realização de despesas distintas do pagamento de benefícios do regime geral de previdência social de que trata o art. 201. (Incluído pela Emenda Constitucional nº 20, de 1998)

§ 1º Nenhum investimento cuja execução ultrapasse um exercício financeiro poderá ser iniciado sem prévia inclusão no plano plurianual, ou sem lei que autorize a inclusão, sob pena de crime de responsabilidade.

§ 2º Os créditos especiais e extraordinários terão vigência no exercício financeiro em que forem autorizados, salvo se o ato de autorização for promulgado nos últimos quatro meses daquele exercício, caso em que, reabertos nos limites de seus saldos, serão incorporados ao orçamento do exercício financeiro subseqüente.

§ 3º A abertura de crédito extraordinário somente será admitida para atender a despesas imprevisíveis e urgentes, como as decorrentes de guerra, comoção interna ou calamidade pública, observado o disposto no art. 62.

§ 4.º É permitida a vinculação de receitas próprias geradas pelos impostos a que se referem os arts. 155 e 156, e dos recursos de que tratam os arts. 157, 158 e 159, I, a e b, e II, para a prestação de garantia ou contragarantia à União e para pagamento de débitos para com esta. (Incluído pela Emenda Constitucional nº 3, de 1993)

§ 5º A transposição, o remanejamento ou a transferência de recursos de uma categoria de programação para outra poderão ser admitidos, no âmbito das atividades de ciência, tecnologia e inovação, com o objetivo de viabilizar os resultados de projetos restritos a essas funções, mediante ato do Poder Executivo, sem necessidade da prévia autorização legislativa prevista no inciso VI deste artigo. (Incluído pela Emenda Constitucional nº 85, de 2015)

Art. 168. Os recursos correspondentes às dotações orçamentárias, compreendidos os créditos suplementares e especiais, destinados aos órgãos dos Poderes Legislativo e Judiciário, do Ministério Público e da Defensoria Pública, ser-lhes-ão entregues até o dia 20 de cada mês, em duodécimos, na forma da lei complementar a que se refere o art. 165, § 9º. (Redação dada pela Emenda Constitucional nº 45, de 2004)

Art. 169. A despesa com pessoal ativo e inativo da União, dos Estados, do Distrito Federal e dos Municípios não poderá exceder os limites estabelecidos em lei complementar. (Redação dada pela pela Emenda Constitucional nº 19, de 1998)

I - se houver prévia dotação orçamentária suficiente para atender às projeções de despesa de pessoal e aos acréscimos dela decorrentes; (Incluído pela Emenda Constitucional nº 19, de 1998)

II - se houver autorização específica na lei de diretrizes orçamentárias, ressalvadas as empresas públicas e as sociedades de economia mista. (Incluído pela Emenda Constitucional nº 19, de 1998)

§ 2º Decorrido o prazo estabelecido na lei complementar referida neste artigo para a adaptação aos parâmetros ali previstos, serão imediatamente suspensos todos os repasses de verbas federais ou estaduais aos Estados, ao Distrito Federal e aos Municípios que não observarem os referidos limites. (Incluído pela Emenda Constitucional nº 19, de 1998)

§ 3º Para o cumprimento dos limites estabelecidos com base neste artigo, durante o prazo fixado na lei complementar referida no caput, a União, os Estados, o Distrito Federal e os Municípios adotarão as seguintes providências: (Incluído pela Emenda Constitucional nº 19, de 1998)

I - redução em pelo menos vinte por cento das despesas com cargos em comissão e funções de confiança; (Incluído pela Emenda Constitucional nº 19, de 1998)

II - exoneração dos servidores não estáveis. (Incluído pela Emenda Constitucional nº 19, de 1998) (Vide Emenda Constitucional nº 19, de 1998)

§ 4º Se as medidas adotadas com base no parágrafo anterior não forem suficientes para assegurar o cumprimento da determinação da lei complementar referida neste artigo, o servidor estável poderá perder o cargo, desde que ato normativo motivado de cada um dos Poderes especifique a atividade funcional, o órgão ou unidade administrativa objeto da redução de pessoal. (Incluído pela Emenda Constitucional nº 19, de 1998)

§ 5º O servidor que perder o cargo na forma do parágrafo anterior fará jus a indenização correspondente a um mês de remuneração por ano de serviço. (Incluído pela Emenda Constitucional nº 19, de 1998)

§ 6º O cargo objeto da redução prevista nos parágrafos anteriores será considerado extinto, vedada a criação de cargo, emprego ou função com atribuições iguais ou assemelhadas pelo prazo de quatro anos. (Incluído pela Emenda Constitucional nº 19, de 1998)

§ 7º Lei federal disporá sobre as normas gerais a serem obedecidas na efetivação do disposto no § 4º. (Incluído pela Emenda Constitucional nº 19, de 1998)

TÍTULO VII

DA ORDEM ECONÔMICA E FINANCEIRA

CAPÍTULO I

DOS PRINCÍPIOS GERAIS DA ATIVIDADE ECONÔMICA

Art. 170. A ordem econômica, fundada na valorização do trabalho humano e na livre iniciativa, tem por fim assegurar a todos existência digna, conforme os ditames da justiça social, observados os seguintes princípios:

I - soberania nacional;

II - propriedade privada;

III - função social da propriedade;

IV - livre concorrência;

V - defesa do consumidor;

VI - defesa do meio ambiente, inclusive mediante tratamento diferenciado conforme o impacto ambiental dos produtos e serviços e de seus processos de elaboração e prestação; (Redação dada pela Emenda Constitucional nº 42, de 19.12.2003)

VII - redução das desigualdades regionais e sociais;

VIII - busca do pleno emprego;

IX - tratamento favorecido para as empresas de pequeno porte constituídas sob as leis brasileiras e que tenham sua sede e administração no País. (Redação dada pela Emenda Constitucional nº 6, de 1995)

Parágrafo único. É assegurado a todos o livre exercício de qualquer atividade econômica, independentemente de autorização de órgãos públicos, salvo nos casos previstos em lei. (Vide Lei nº 13.874, de 2019)

Art. 171. São consideradas: (Revogado pela Emenda Constitucional nº 6, de 1995)
I - empresa brasileira a constituída sob as leis brasileiras e que tenha sua sede e administração no País; (Revogado pela Emenda Constitucional nº 6, de 1995)
II - empresa brasileira de capital nacional aquela cujo controle efetivo esteja em caráter permanente sob a titularidade direta ou indireta de pessoas físicas domiciliadas e residentes no País ou de entidades de direito público interno, entendendo-se por controle efetivo da empresa a titularidade da maioria de seu capital votante e o exercício, de fato e de direito, do poder decisório para gerir suas atividades. (Revogado pela Emenda Constitucional nº 6, de 1995)
§ 1º - A lei poderá, em relação à empresa brasileira de capital nacional: (Revogado pela Emenda Constitucional nº 6, de 1995)
I - conceder proteção e benefícios especiais temporários para desenvolver atividades consideradas estratégicas para a defesa nacional ou imprescindíveis ao desenvolvimento do País; (Revogado pela Emenda Constitucional nº 6, de 1995)
II - estabelecer, sempre que considerar um setor imprescindível ao desenvolvimento tecnológico nacional, entre outras condições e requisitos: (Revogado pela Emenda Constitucional nº 6, de 1995)
a) a exigência de que o controle referido no inciso II do "caput" se estenda às atividades tecnológicas da empresa, assim entendido o exercício, de fato e de direito, do poder decisório para desenvolver ou absorver tecnologia; (Revogado pela Emenda Constitucional nº 6, de 1995)
b) percentuais de participação, no capital, de pessoas físicas domiciliadas e residentes no País ou entidades de direito público interno. (Revogado pela Emenda Constitucional nº 6, de 1995)

§ 2º - Na aquisição de bens e serviços, o Poder Público dará tratamento preferencial, nos termos da lei, à empresa brasileira de capital nacional . (Revogado pela Emenda Constitucional nº 6, de 1995)

Art. 172. A lei disciplinará, com base no interesse nacional, os investimentos de capital estrangeiro, incentivará os reinvestimentos e regulará a remessa de lucros.

Art. 173. Ressalvados os casos previstos nesta Constituição, a exploração direta de atividade econômica pelo Estado só será permitida quando necessária aos imperativos da segurança nacional ou a relevante interesse coletivo, conforme definidos em lei.

§ 1º A lei estabelecerá o estatuto jurídico da empresa pública, da sociedade de economia mista e de suas subsidiárias que explorem atividade econômica de produção ou comercialização de bens ou de prestação de serviços, dispondo sobre: (Redação dada pela Emenda Constitucional nº 19, de 1998)

I - sua função social e formas de fiscalização pelo Estado e pela sociedade; (Incluído pela Emenda Constitucional nº 19, de 1998)

II - a sujeição ao regime jurídico próprio das empresas privadas, inclusive quanto aos direitos e obrigações civis, comerciais, trabalhistas e tributários; (Incluído pela Emenda Constitucional nº 19, de 1998)

III - licitação e contratação de obras, serviços, compras e alienações, observados os princípios da administração pública; (Incluído pela Emenda Constitucional nº 19, de 1998)

IV - a constituição e o funcionamento dos conselhos de administração e fiscal, com a participação de acionistas minoritários; (Incluído pela Emenda Constitucional nº 19, de 1998)

V - os mandatos, a avaliação de desempenho e a responsabilidade dos administradores. (Incluído pela Emenda Constitucional nº 19, de 1998)

§ 2º As empresas públicas e as sociedades de economia mista não poderão gozar de privilégios fiscais não extensivos às do setor privado.

§ 3º A lei regulamentará as relações da empresa pública com o Estado e a sociedade.

§ 4º A lei reprimirá o abuso do poder econômico que vise à dominação dos mercados, à eliminação da concorrência e ao aumento arbitrário dos lucros.

§ 5º A lei, sem prejuízo da responsabilidade individual dos dirigentes da pessoa jurídica, estabelecerá a responsabilidade desta, sujeitando-a às punições compatíveis com sua natureza, nos atos praticados contra a ordem econômica e financeira e contra a economia popular.

Art. 174. Como agente normativo e regulador da atividade econômica, o Estado exercerá, na forma da lei, as funções de fiscalização, incentivo e planejamento, sendo este determinante para o setor público e indicativo para o setor privado. (Vide Lei nº 13.874, de 2019)

§ 1º A lei estabelecerá as diretrizes e bases do planejamento do desenvolvimento nacional equilibrado, o qual incorporará e compatibilizará os planos nacionais e regionais de desenvolvimento.

§ 2º A lei apoiará e estimulará o cooperativismo e outras formas de associativismo.

§ 3º O Estado favorecerá a organização da atividade garimpeira em cooperativas, levando em conta a proteção do meio ambiente e a promoção econômico-social dos garimpeiros.

§ 4º As cooperativas a que se refere o parágrafo anterior terão prioridade na autorização ou concessão para pesquisa e lavra dos recursos e jazidas de minerais garimpáveis, nas áreas onde estejam atuando, e naquelas fixadas de acordo com o art. 21, XXV, na forma da lei.

Art. 175. Incumbe ao Poder Público, na forma da lei, diretamente ou sob regime de concessão ou permissão, sempre através de licitação, a prestação de serviços públicos.

Parágrafo único. A lei disporá sobre:

I - o regime das empresas concessionárias e permissionárias de serviços públicos, o caráter especial de seu contrato e de sua prorrogação, bem como as condições de caducidade, fiscalização e rescisão da concessão ou permissão;

II - os direitos dos usuários;

III - política tarifária;

IV - a obrigação de manter serviço adequado.

Art. 176. As jazidas, em lavra ou não, e demais recursos minerais e os potenciais de energia hidráulica constituem propriedade distinta da do solo, para efeito de exploração ou aproveitamento, e pertencem à União, garantida ao concessionário a propriedade do produto da lavra.

§ 1º A pesquisa e a lavra de recursos minerais e o aproveitamento dos potenciais a que se refere o "caput" deste artigo somente poderão ser efetuados mediante autorização ou concessão da União, no interesse nacional, por brasileiros ou empresa constituída sob as leis brasileiras e que tenha sua sede e administração no País, na forma da lei, que estabelecerá as condições específicas quando essas atividades se desenvolverem em faixa de fronteira ou terras indígenas. (Redação dada pela Emenda Constitucional nº 6, de 1995)

§ 2º É assegurada participação ao proprietário do solo nos resultados da lavra, na forma e no valor que dispuser a lei.

§ 3º A autorização de pesquisa será sempre por prazo determinado, e as autorizações e concessões previstas neste artigo não poderão ser cedidas ou transferidas, total ou parcialmente, sem prévia anuência do poder concedente.

§ 4º Não dependerá de autorização ou concessão o aproveitamento do potencial de energia renovável de capacidade reduzida.

Art. 177. Constituem monopólio da União:

I - a pesquisa e a lavra das jazidas de petróleo e gás natural e outros hidrocarbonetos fluidos; (Vide Emenda Constitucional nº 9, de 1995)

II - a refinação do petróleo nacional ou estrangeiro;

III - a importação e exportação dos produtos e derivados básicos resultantes das atividades previstas nos incisos anteriores;

IV - o transporte marítimo do petróleo bruto de origem nacional ou de derivados básicos de petróleo produzidos no País, bem assim o transporte, por meio de conduto, de petróleo bruto, seus derivados e gás natural de qualquer origem;

V - a pesquisa, a lavra, o enriquecimento, o reprocessamento, a industrialização e o comércio de minérios e minerais nucleares e seus derivados, com exceção dos radioisótopos cuja

produção, comercialização e utilização poderão ser autorizadas sob regime de permissão, conforme as alíneas b e c do inciso XXIII do caput do art. 21 desta Constituição Federal. (Redação dada pela Emenda Constitucional nº 49, de 2006)

§ 1º A União poderá contratar com empresas estatais ou privadas a realização das atividades previstas nos incisos I a IV deste artigo observadas as condições estabelecidas em lei. (Redação dada pela Emenda Constitucional nº 9, de 1995) (Vide Emenda Constitucional nº 9, de 1995)

§ 2º A lei a que se refere o § 1º disporá sobre: (Incluído pela Emenda Constitucional nº 9, de 1995) (Vide Emenda Constitucional nº 9, de 1995)

I - a garantia do fornecimento dos derivados de petróleo em todo o território nacional; (Incluído pela Emenda Constitucional nº 9, de 1995)

II - as condições de contratação; (Incluído pela Emenda Constitucional nº 9, de 1995)

III - a estrutura e atribuições do órgão regulador do monopólio da União; (Incluído pela Emenda Constitucional nº 9, de 1995)

§ 3º A lei disporá sobre o transporte e a utilização de materiais radioativos no território nacional. (Renumerado de § 2º para 3º pela Emenda Constitucional nº 9, de 1995)

§ 4º A lei que instituir contribuição de intervenção no domínio econômico relativa às atividades de importação ou comercialização de petróleo e seus derivados, gás natural e seus derivados e álcool combustível deverá atender aos seguintes requisitos: (Incluído pela Emenda Constitucional nº 33, de 2001)

I - a alíquota da contribuição poderá ser: (Incluído pela Emenda Constitucional nº 33, de 2001)

a) diferenciada por produto ou uso; (Incluído pela Emenda Constitucional nº 33, de 2001)

b)reduzida e restabelecida por ato do Poder Executivo, não se lhe aplicando o disposto no art. 150,III, *b* ; (Incluído pela Emenda Constitucional nº 33, de 2001)

II - os recursos arrecadados serão destinados: (Incluído pela Emenda Constitucional nº 33, de 2001)

a) ao pagamento de subsídios a preços ou transporte de álcool combustível, gás natural e seus derivados e derivados de petróleo; (Incluído pela Emenda Constitucional nº 33, de 2001)

b) ao financiamento de projetos ambientais relacionados com a indústria do petróleo e do gás; (Incluído pela Emenda Constitucional nº 33, de 2001)

c) ao financiamento de programas de infra-estrutura de transportes. (Incluído pela Emenda Constitucional nº 33, de 2001)

Art. 178. A lei disporá sobre a ordenação dos transportes aéreo, aquático e terrestre, devendo, quanto à ordenação do transporte internacional, observar os acordos firmados pela União, atendido o princípio da reciprocidade. (Redação dada pela Emenda Constitucional nº 7, de 1995)

Parágrafo único. Na ordenação do transporte aquático, a lei estabelecerá as condições em que o transporte de mercadorias na cabotagem e a navegação interior poderão ser feitos por embarcações estrangeiras. (Incluído pela Emenda Constitucional nº 7, de 1995)

Art. 179. A União, os Estados, o Distrito Federal e os Municípios dispensarão às microempresas e às empresas de pequeno porte, assim definidas em lei, tratamento jurídico diferenciado, visando a incentivá-las pela simplificação de suas obrigações administrativas, tributárias, previdenciárias e creditícias, ou pela eliminação ou redução destas por meio de lei.

Art. 180. A União, os Estados, o Distrito Federal e os Municípios promoverão e incentivarão o turismo como fator de desenvolvimento social e econômico.

Art. 181. O atendimento de requisição de documento ou informação de natureza comercial, feita por autoridade administrativa ou judiciária estrangeira, a pessoa física ou jurídica residente ou domiciliada no País dependerá de autorização do Poder competente.

CAPÍTULO II
DA POLÍTICA URBANA

Art. 182. A política de desenvolvimento urbano, executada pelo Poder Público municipal, conforme diretrizes gerais fixadas em lei, tem por objetivo ordenar o pleno desenvolvimento das funções sociais da cidade e garantir o bem- estar de seus habitantes. (Regulamento) (Vide Lei nº 13.311, de 11 de julho de 2016)

§ 1º O plano diretor, aprovado pela Câmara Municipal, obrigatório para cidades com mais de vinte mil habitantes, é o instrumento básico da política de desenvolvimento e de expansão urbana.

§ 2º A propriedade urbana cumpre sua função social quando atende às exigências fundamentais de ordenação da cidade expressas no plano diretor.

§ 3º As desapropriações de imóveis urbanos serão feitas com prévia e justa indenização em dinheiro.

§ 4º É facultado ao Poder Público municipal, mediante lei específica para área incluída no plano diretor, exigir, nos termos da lei federal, do proprietário do solo urbano não edificado, subutilizado ou não utilizado, que promova seu adequado aproveitamento, sob pena, sucessivamente, de:

I - parcelamento ou edificação compulsórios;

II - imposto sobre a propriedade predial e territorial urbana progressivo no tempo;

III - desapropriação com pagamento mediante títulos da dívida pública de emissão previamente aprovada pelo Senado Federal, com prazo de resgate de até dez anos, em parcelas anuais, iguais e sucessivas, assegurados o valor real da indenização e os juros legais.

Art. 183. Aquele que possuir como sua área urbana de até duzentos e cinqüenta metros quadrados, por cinco anos, ininterruptamente e sem oposição, utilizando-a para sua moradia ou de sua família, adquirir-lhe-á o domínio, desde que não seja proprietário de outro imóvel urbano ou rural. (Regulamento)

§ 1º O título de domínio e a concessão de uso serão conferidos ao homem ou à mulher, ou a ambos, independentemente do estado civil.

§ 2º Esse direito não será reconhecido ao mesmo possuidor mais de uma vez.

§ 3º Os imóveis públicos não serão adquiridos por usucapião.

CAPÍTULO III
DA POLÍTICA AGRÍCOLA E FUNDIÁRIA E DA REFORMA AGRÁRIA

Regulamento

Art. 184. Compete à União desapropriar por interesse social, para fins de reforma agrária, o imóvel rural que não esteja cumprindo sua função social, mediante prévia e justa indenização em títulos da dívida agrária, com cláusula de preservação do valor real, resgatáveis no prazo de até vinte anos, a partir do segundo ano de sua emissão, e cuja utilização será definida em lei.

§ 1º As benfeitorias úteis e necessárias serão indenizadas em dinheiro.

§ 2º O decreto que declarar o imóvel como de interesse social, para fins de reforma agrária, autoriza a União a propor a ação de desapropriação.

§ 3º Cabe à lei complementar estabelecer procedimento contraditório especial, de rito sumário, para o processo judicial de desapropriação.

§ 4º O orçamento fixará anualmente o volume total de títulos da dívida agrária, assim como o montante de recursos para atender ao programa de reforma agrária no exercício.

§ 5º São isentas de impostos federais, estaduais e municipais as operações de transferência de imóveis desapropriados para fins de reforma agrária.

Art. 185. São insuscetíveis de desapropriação para fins de reforma agrária:

I - a pequena e média propriedade rural, assim definida em lei, desde que seu proprietário não possua outra;

II - a propriedade produtiva.

Parágrafo único. A lei garantirá tratamento especial à propriedade produtiva e fixará normas para o cumprimento dos requisitos relativos a sua função social.

Art. 186. A função social é cumprida quando a propriedade rural atende, simultaneamente, segundo critérios e graus de exigência estabelecidos em lei, aos seguintes requisitos:

I - aproveitamento racional e adequado;

II - utilização adequada dos recursos naturais disponíveis e preservação do meio ambiente;

III - observância das disposições que regulam as relações de trabalho;

IV - exploração que favoreça o bem-estar dos proprietários e dos trabalhadores.

Art. 187. A política agrícola será planejada e executada na forma da lei, com a participação efetiva do setor de produção, envolvendo produtores e trabalhadores rurais, bem como dos setores de comercialização, de armazenamento e de transportes, levando em conta, especialmente:

I - os instrumentos creditícios e fiscais;

II - os preços compatíveis com os custos de produção e a garantia de comercialização;

III - o incentivo à pesquisa e à tecnologia;

IV - a assistência técnica e extensão rural;

V - o seguro agrícola;

VI - o cooperativismo;

VII - a eletrificação rural e irrigação;

VIII - a habitação para o trabalhador rural.

§ 1º Incluem-se no planejamento agrícola as atividades agro-industriais, agropecuárias, pesqueiras e florestais.

§ 2º Serão compatibilizadas as ações de política agrícola e de reforma agrária.

Art. 188. A destinação de terras públicas e devolutas será compatibilizada com a política agrícola e com o plano nacional de reforma agrária.

§ 1º A alienação ou a concessão, a qualquer título, de terras públicas com área superior a dois mil e quinhentos hectares a pessoa física ou jurídica, ainda que por interposta pessoa, dependerá de prévia aprovação do Congresso Nacional.

§ 2º Excetuam-se do disposto no parágrafo anterior as alienações ou as concessões de terras públicas para fins de reforma agrária.

Art. 189. Os beneficiários da distribuição de imóveis rurais pela reforma agrária receberão títulos de domínio ou de concessão de uso, inegociáveis pelo prazo de dez anos.

Parágrafo único. O título de domínio e a concessão de uso serão conferidos ao homem ou à mulher, ou a ambos, independentemente do estado civil, nos termos e condições previstos em lei.

Art. 190. A lei regulará e limitará a aquisição ou o arrendamento de propriedade rural por pessoa física ou jurídica estrangeira e estabelecerá os casos que dependerão de autorização do Congresso Nacional.

Art. 191. Aquele que, não sendo proprietário de imóvel rural ou urbano, possua como seu, por cinco anos ininterruptos, sem oposição, área de terra, em zona rural, não superior a cinqüenta hectares, tornando-a produtiva por seu trabalho ou de sua família, tendo nela sua moradia, adquirir-lhe-á a propriedade.

Parágrafo único. Os imóveis públicos não serão adquiridos por usucapião.

CAPÍTULO IV
DO SISTEMA FINANCEIRO NACIONAL

Art. 192. O sistema financeiro nacional, estruturado de forma a promover o desenvolvimento equilibrado do País e a servir aos interesses da coletividade, em todas as partes que o compõem, abrangendo as cooperativas de crédito, será regulado por leis complementares que disporão, inclusive, sobre a participação do capital estrangeiro nas instituições que o integram. (Redação dada pela Emenda Constitucional nº 40, de 2003) (Vide Lei nº 8.392, de 1991)

I - (Revogado). (Redação dada pela Emenda Constitucional nº 40, de 2003)

II - (Revogado). (Redação dada pela Emenda Constitucional nº 40, de 2003)

III - (Revogado) (Redação dada pela Emenda Constitucional nº 40, de 2003)

a) (Revogado) (Redação dada pela Emenda Constitucional nº 40, de 2003)

b) (Revogado) (Redação dada pela Emenda Constitucional nº 40, de 2003)

IV - (Revogado) (Redação dada pela Emenda Constitucional nº 40, de 2003)

V - (Revogado) (Redação dada pela Emenda Constitucional nº 40, de 2003)

VI - (Revogado) (Redação dada pela Emenda Constitucional nº 40, de 2003)

VII - (Revogado) (Redação dada pela Emenda Constitucional nº 40, de 2003)

VIII - (Revogado) (Redação dada pela Emenda Constitucional nº 40, de 2003)

§ 1° (Revogado) (Redação dada pela Emenda Constitucional nº 40, de 2003)

§ 2° (Revogado) (Redação dada pela Emenda Constitucional nº 40, de 2003)

§ 3° (Revogado) (Redação dada pela Emenda Constitucional nº 40, de 2003)

TÍTULO VIII
DA ORDEM SOCIAL
CAPÍTULO I
DISPOSIÇÃO GERAL

Art. 193. A ordem social tem como base o primado do trabalho, e como objetivo o bem-estar e a justiça sociais.

CAPÍTULO II
DA SEGURIDADE SOCIAL
SEÇÃO I
DISPOSIÇÕES GERAIS

Art. 194. A seguridade social compreende um conjunto integrado de ações de iniciativa dos Poderes Públicos e da sociedade, destinadas a assegurar os direitos relativos à saúde, à previdência e à assistência social.

Parágrafo único. Compete ao Poder Público, nos termos da lei, organizar a seguridade social, com base nos seguintes objetivos:

I - universalidade da cobertura e do atendimento;

II - uniformidade e equivalência dos benefícios e serviços às populações urbanas e rurais;

III - seletividade e distributividade na prestação dos benefícios e serviços;

IV - irredutibilidade do valor dos benefícios;

V - eqüidade na forma de participação no custeio;

~~VI - diversidade da base de financiamento;~~

VII - caráter democrático e descentralizado da administração, mediante gestão quadripartite, com participação dos trabalhadores, dos empregadores, dos aposentados e do Governo nos órgãos colegiados. (Redação dada pela Emenda Constitucional nº 20, de 1998)

Art. 195. A seguridade social será financiada por toda a sociedade, de forma direta e indireta, nos termos da lei, mediante recursos provenientes dos orçamentos da União, dos Estados, do Distrito Federal e dos Municípios, e das seguintes contribuições sociais: (Vide Emenda Constitucional nº 20, de 1998)

I - do empregador, da empresa e da entidade a ela equiparada na forma da lei, incidentes sobre: (Redação dada pela Emenda Constitucional nº 20, de 1998)

a) a folha de salários e demais rendimentos do trabalho pagos ou creditados, a qualquer título, à pessoa física que lhe preste serviço, mesmo sem vínculo empregatício; (Incluído pela Emenda Constitucional nº 20, de 1998)

b) a receita ou o faturamento; (Incluído pela Emenda Constitucional nº 20, de 1998)

c) o lucro; (Incluído pela Emenda Constitucional nº 20, de 1998)

II - dos trabalhadores;
II - do trabalhador e dos demais segurados da previdência social, não incidindo contribuição sobre aposentadoria e pensão concedidas pelo regime geral de previdência social de que trata o art. 201; (Redação dada pela Emenda Constitucional nº 20, de 1998)

III - sobre a receita de concursos de prognósticos.

IV - do importador de bens ou serviços do exterior, ou de quem a lei a ele equiparar. (Incluído pela Emenda Constitucional nº 42, de 19.12.2003)

§ 1º As receitas dos Estados, do Distrito Federal e dos Municípios destinadas à seguridade social constarão dos respectivos orçamentos, não integrando o orçamento da União.

§ 2º A proposta de orçamento da seguridade social será elaborada de forma integrada pelos órgãos responsáveis pela saúde, previdência social e assistência social, tendo em vista as metas e prioridades estabelecidas na lei de diretrizes orçamentárias, assegurada a cada área a gestão de seus recursos.

§ 4º A lei poderá instituir outras fontes destinadas a garantir a manutenção ou expansão da seguridade social, obedecido o disposto no art. 154, I.

§ 5º Nenhum benefício ou serviço da seguridade social poderá ser criado, majorado ou estendido sem a correspondente fonte de custeio total.

§ 6º As contribuições sociais de que trata este artigo só poderão ser exigidas após decorridos noventa dias da data da publicação da lei que as houver instituído ou modificado, não se lhes aplicando o disposto no art. 150, III, "b".

§ 7º São isentas de contribuição para a seguridade social as entidades beneficentes de assistência social que atendam às exigências estabelecidas em lei.

§ 8º O produtor, o parceiro, o meeiro e o arrendatário rurais e o pescador artesanal, bem como os respectivos cônjuges, que exerçam suas atividades em regime de economia familiar, sem empregados permanentes, contribuirão para a seguridade social mediante a aplicação de uma alíquota sobre o resultado da comercialização da produção e farão jus aos benefícios nos termos da lei. (Redação dada pela Emenda Constitucional nº 20, de 1998)

§ 9º As contribuições sociais previstas no inciso I do caput deste artigo poderão ter alíquotas ou bases de cálculo diferenciadas, em razão da atividade econômica, da utilização intensiva de mão-de-obra, do porte da empresa ou da condição estrutural do mercado de trabalho. (Redação dada pela Emenda Constitucional nº 47, de 2005)

§ 10. A lei definirá os critérios de transferência de recursos para o sistema único de saúde e ações de assistência social da União para os Estados, o Distrito Federal e os Municípios, e dos Estados para os Municípios, observada a respectiva contrapartida de recursos. (Incluído pela Emenda Constitucional nº 20, de 1998)

§ 11. É vedada a concessão de remissão ou anistia das contribuições sociais de que tratam os incisos I, a, e II deste artigo, para débitos em montante superior ao fixado em lei complementar. (Incluído pela Emenda Constitucional nº 20, de 1998)

§ 12. A lei definirá os setores de atividade econômica para os quais as contribuições incidentes na forma dos incisos I, b; e IV do *capu* t, serão não-cumulativas. (Incluído pela Emenda Constitucional nº 42, de 19.12.2003)

SEÇÃO II
DA SAÚDE

Art. 196. A saúde é direito de todos e dever do Estado, garantido mediante políticas sociais e econômicas que visem à redução do risco de doença e de outros agravos e ao acesso universal e igualitário às ações e serviços para sua promoção, proteção e recuperação.

Art. 197. São de relevância pública as ações e serviços de saúde, cabendo ao Poder Público dispor, nos termos da lei, sobre sua regulamentação, fiscalização e controle, devendo sua execução ser feita diretamente ou através de terceiros e, também, por pessoa física ou jurídica de direito privado.

Art. 198. As ações e serviços públicos de saúde integram uma rede regionalizada e hierarquizada e constituem um sistema único, organizado de acordo com as seguintes diretrizes: (Vide ADPF 672)

I - descentralização, com direção única em cada esfera de governo;

II - atendimento integral, com prioridade para as atividades preventivas, sem prejuízo dos serviços assistenciais;

III - participação da comunidade.

§ 1º. O sistema único de saúde será financiado, nos termos do art. 195, com recursos do orçamento da seguridade social, da União, dos Estados, do Distrito Federal e dos Municípios, além de outras fontes. (Parágrafo único renumerado para § 1º pela Emenda Constitucional nº 29, de 2000)

§ 2º A União, os Estados, o Distrito Federal e os Municípios aplicarão, anualmente, em ações e serviços públicos de saúde recursos mínimos derivados da aplicação de percentuais calculados sobre: (Incluído pela Emenda Constitucional nº 29, de 2000)

I - no caso da União, a receita corrente líquida do respectivo exercício financeiro, não podendo ser inferior a 15% (quinze por cento); (Redação dada pela Emenda Constitucional nº 86, de 2015)

II - no caso dos Estados e do Distrito Federal, o produto da arrecadação dos impostos a que se refere o art. 155 e dos recursos de que tratam os arts. 157 e 159, inciso I, alínea a, e inciso II,

deduzidas as parcelas que forem transferidas aos respectivos Municípios; (Incluído pela Emenda Constitucional nº 29, de 2000)

III - no caso dos Municípios e do Distrito Federal, o produto da arrecadação dos impostos a que se refere o art. 156 e dos recursos de que tratam os arts. 158 e 159, inciso I, alínea b e § 3º. (Incluído pela Emenda Constitucional nº 29, de 2000)

§ 3º Lei complementar, que será reavaliada pelo menos a cada cinco anos, estabelecerá: (Incluído pela Emenda Constitucional nº 29, de 2000) Regulamento

I - os percentuais de que tratam os incisos II e III do § 2º; (Redação dada pela Emenda Constitucional nº 86, de 2015)

II - os critérios de rateio dos recursos da União vinculados à saúde destinados aos Estados, ao Distrito Federal e aos Municípios, e dos Estados destinados a seus respectivos Municípios, objetivando a progressiva redução das disparidades regionais; (Incluído pela Emenda Constitucional nº 29, de 2000)

III - as normas de fiscalização, avaliação e controle das despesas com saúde nas esferas federal, estadual, distrital e municipal; (Incluído pela Emenda Constitucional nº 29, de 2000)

IV - (revogado) . (Redação dada pela Emenda Constitucional nº 86, de 2015)

§ 4º Os gestores locais do sistema único de saúde poderão admitir agentes comunitários de saúde e agentes de combate às endemias por meio de processo seletivo público, de acordo com a natureza e complexidade de suas atribuições e requisitos específicos para sua atuação. (Incluído pela Emenda Constitucional nº 51, de 2006)

§ 5º Lei federal disporá sobre o regime jurídico, o piso salarial profissional nacional, as diretrizes para os Planos de Carreira e a regulamentação das atividades de agente comunitário de saúde e agente de combate às endemias, competindo à União, nos termos da lei, prestar assistência financeira complementar aos Estados, ao Distrito Federal e aos Municípios, para o cumprimento do referido piso salarial. (Redação dada pela Emenda Constitucional nº 63, de 2010) Regulamento

§ 6º Além das hipóteses previstas no § 1º do art. 41 e no § 4º do art. 169 da Constituição Federal, o servidor que exerça funções equivalentes às de agente comunitário de saúde ou de agente de combate às endemias poderá perder o cargo em caso de descumprimento dos requisitos específicos, fixados em lei, para o seu exercício. (Incluído pela Emenda Constitucional nº 51, de 2006)

Art. 199. A assistência à saúde é livre à iniciativa privada.

§ 1º - As instituições privadas poderão participar de forma complementar do sistema único de saúde, segundo diretrizes deste, mediante contrato de direito público ou convênio, tendo preferência as entidades filantrópicas e as sem fins lucrativos.

§ 2º É vedada a destinação de recursos públicos para auxílios ou subvenções às instituições privadas com fins lucrativos.

§ 3º É vedada a participação direta ou indireta de empresas ou capitais estrangeiros na assistência à saúde no País, salvo nos casos previstos em lei.

§ 4º A lei disporá sobre as condições e os requisitos que facilitem a remoção de órgãos, tecidos e substâncias humanas para fins de transplante, pesquisa e tratamento, bem como a coleta, processamento e transfusão de sangue e seus derivados, sendo vedado todo tipo de comercialização.

Art. 200. Ao sistema único de saúde compete, além de outras atribuições, nos termos da lei:

I - controlar e fiscalizar procedimentos, produtos e substâncias de interesse para a saúde e participar da produção de medicamentos, equipamentos, imunobiológicos, hemoderivados e outros insumos;

II - executar as ações de vigilância sanitária e epidemiológica, bem como as de saúde do trabalhador;

III - ordenar a formação de recursos humanos na área de saúde;

IV - participar da formulação da política e da execução das ações de saneamento básico;

V - incrementar, em sua área de atuação, o desenvolvimento científico e tecnológico e a inovação; (Redação dada pela Emenda Constitucional nº 85, de 2015)

VI - fiscalizar e inspecionar alimentos, compreendido o controle de seu teor nutricional, bem como bebidas e águas para consumo humano;

VII - participar do controle e fiscalização da produção, transporte, guarda e utilização de substâncias e produtos psicoativos, tóxicos e radioativos;

VIII - colaborar na proteção do meio ambiente, nele compreendido o do trabalho.

SEÇÃO III
DA PREVIDÊNCIA SOCIAL

Art. 201. A previdência social será organizada sob a forma de regime geral, de caráter contributivo e de filiação obrigatória, observados critérios que preservem o equilíbrio financeiro e atuarial, e atenderá, nos termos da lei, a: (Redação dada pela Emenda Constitucional nº 20, de 1998) (Vide Emenda Constitucional nº 20, de 1998)

I - cobertura dos eventos de doença, invalidez, morte e idade avançada; (Redação dada pela Emenda Constitucional nº 20, de 1998)

II - proteção à maternidade, especialmente à gestante; (Redação dada pela Emenda Constitucional nº 20, de 1998)

III - proteção ao trabalhador em situação de desemprego involuntário; (Redação dada pela Emenda Constitucional nº 20, de 1998)

IV - salário-família e auxílio-reclusão para os dependentes dos segurados de baixa renda; (Redação dada pela Emenda Constitucional nº 20, de 1998)

V - pensão por morte do segurado, homem ou mulher, ao cônjuge ou companheiro e dependentes, observado o disposto no § 2º. (Redação dada pela Emenda Constitucional nº 20, de 1998)

§ 1º É vedada a adoção de requisitos e critérios diferenciados para a concessão de aposentadoria aos beneficiários do regime geral de previdência social, ressalvados os casos de atividades exercidas sob condições especiais que prejudiquem a saúde ou a integridade física e quando se tratar de segurados portadores de deficiência, nos termos definidos em lei complementar. (Redação dada pela Emenda Constitucional nº 47, de 2005) (Regulamento) (Vigência)

§ 2º Nenhum benefício que substitua o salário de contribuição ou o rendimento do trabalho do segurado terá valor mensal inferior ao salário mínimo. (Redação dada pela Emenda Constitucional nº 20, de 1998)

§ 3º Todos os salários de contribuição considerados para o cálculo de benefício serão devidamente atualizados, na forma da lei. (Redação dada pela Emenda Constitucional nº 20, de 1998)

§ 4º É assegurado o reajustamento dos benefícios para preservar-lhes, em caráter permanente, o valor real, conforme critérios definidos em lei. (Redação dada pela Emenda Constitucional nº 20, de 1998)

§ 5º É vedada a filiação ao regime geral de previdência social, na qualidade de segurado facultativo, de pessoa participante de regime próprio de previdência. (Redação dada pela Emenda Constitucional nº 20, de 1998)

§ 6º A gratificação natalina dos aposentados e pensionistas terá por base o valor dos proventos do mês de dezembro de cada ano. (Redação dada pela Emenda Constitucional nº 20, de 1998)

§ 7º É assegurada aposentadoria no regime geral de previdência social, nos termos da lei, obedecidas as seguintes condições: (Redação dada pela Emenda Constitucional nº 20, de 1998)

I - trinta e cinco anos de contribuição, se homem, e trinta anos de contribuição, se mulher; (Incluído dada pela Emenda Constitucional nº 20, de 1998)

II - sessenta e cinco anos de idade, se homem, e sessenta anos de idade, se mulher, reduzido em cinco anos o limite para os trabalhadores rurais de ambos os sexos e para os que exerçam suas atividades em regime de economia familiar, nestes incluídos o produtor rural, o garimpeiro e o pescador artesanal. (Incluído dada pela Emenda Constitucional nº 20, de 1998)

§ 8º Os requisitos a que se refere o inciso I do parágrafo anterior serão reduzidos em cinco anos, para o professor que comprove exclusivamente tempo de efetivo exercício das funções de magistério na educação infantil e no ensino fundamental e médio. (Redação dada pela Emenda Constitucional nº 20, de 1998)

§ 9º Para efeito de aposentadoria, é assegurada a contagem recíproca do tempo de contribuição na administração pública e na atividade privada, rural e urbana, hipótese em que os diversos regimes de previdência social se compensarão financeiramente, segundo critérios estabelecidos em lei. (Incluído dada pela Emenda Constitucional nº 20, de 1998)

§ 10. Lei disciplinará a cobertura do risco de acidente do trabalho, a ser atendida concorrentemente pelo regime geral de previdência social e pelo setor privado. (Incluído dada pela Emenda Constitucional nº 20, de 1998)

§ 11. Os ganhos habituais do empregado, a qualquer título, serão incorporados ao salário para efeito de contribuição previdenciária e conseqüente repercussão em benefícios, nos casos e na forma da lei. (Incluído dada pela Emenda Constitucional nº 20, de 1998)

§ 12. Lei disporá sobre sistema especial de inclusão previdenciária para atender a trabalhadores de baixa renda e àqueles sem renda própria que se dediquem exclusivamente ao trabalho doméstico no âmbito de sua residência, desde que pertencentes a famílias de baixa renda, garantindo-lhes acesso a benefícios de valor igual a um salário-mínimo. (Redação dada pela Emenda Constitucional nº 47, de 2005)

§ 13. O sistema especial de inclusão previdenciária de que trata o § 12 deste artigo terá alíquotas e carências inferiores às vigentes para os demais segurados do regime geral de previdência social. (Incluído pela Emenda Constitucional nº 47, de 2005)

Art. 202. O regime de previdência privada, de caráter complementar e organizado de forma autônoma em relação ao regime geral de previdência social, será facultativo, baseado na constituição de reservas que garantam o benefício contratado, e regulado por lei complementar. (Redação dada pela Emenda Constitucional nº 20, de 1998) (Vide Emenda Constitucional nº 20, de 1998)

§ 1° A lei complementar de que trata este artigo assegurará ao participante de planos de benefícios de entidades de previdência privada o pleno acesso às informações relativas à gestão de seus respectivos planos. (Redação dada pela Emenda Constitucional nº 20, de 1998)

§ 2° As contribuições do empregador, os benefícios e as condições contratuais previstas nos estatutos, regulamentos e planos de benefícios das entidades de previdência privada não integram o contrato de trabalho dos participantes, assim como, à exceção dos benefícios concedidos, não integram a remuneração dos participantes, nos termos da lei. (Redação dada pela Emenda Constitucional nº 20, de 1998)

§ 3º É vedado o aporte de recursos a entidade de previdência privada pela União, Estados, Distrito Federal e Municípios, suas autarquias, fundações, empresas públicas, sociedades de economia mista e outras entidades públicas, salvo na qualidade de patrocinador, situação na qual, em hipótese alguma, sua contribuição normal poderá exceder a do segurado. (Incluído pela Emenda Constitucional nº 20, de 1998) (Vide Emenda Constitucional nº 20, de 1998)

§ 4º Lei complementar disciplinará a relação entre a União, Estados, Distrito Federal ou Municípios, inclusive suas autarquias, fundações, sociedades de economia mista e empresas controladas direta ou indiretamente, enquanto patrocinadoras de entidades fechadas de previdência privada, e suas respectivas entidades fechadas de previdência privada. (Incluído pela Emenda Constitucional nº 20, de 1998)

§ 5º A lei complementar de que trata o parágrafo anterior aplicar-se-á, no que couber, às empresas privadas permissionárias ou concessionárias de prestação de serviços públicos, quando patrocinadoras de entidades fechadas de previdência privada. (Incluído pela Emenda Constitucional nº 20, de 1998)

§ 6º A lei complementar a que se refere o § 4° deste artigo estabelecerá os requisitos para a designação dos membros das diretorias das entidades fechadas de previdência privada e disciplinará a inserção dos participantes nos colegiados e instâncias de decisão em que seus interesses sejam objeto de discussão e deliberação. (Incluído pela Emenda Constitucional nº 20, de 1998)

SEÇÃO IV
DA ASSISTÊNCIA SOCIAL

Art. 203. A assistência social será prestada a quem dela necessitar, independentemente de contribuição à seguridade social, e tem por objetivos:

I - a proteção à família, à maternidade, à infância, à adolescência e à velhice;

II - o amparo às crianças e adolescentes carentes;

III - a promoção da integração ao mercado de trabalho;

IV - a habilitação e reabilitação das pessoas portadoras de deficiência e a promoção de sua integração à vida comunitária;

V - a garantia de um salário mínimo de benefício mensal à pessoa portadora de deficiência e ao idoso que comprovem não possuir meios de prover à própria manutenção ou de tê-la provida por sua família, conforme dispuser a lei.

Art. 204. As ações governamentais na área da assistência social serão realizadas com recursos do orçamento da seguridade social, previstos no art. 195, além de outras fontes, e organizadas com base nas seguintes diretrizes:

I - descentralização político-administrativa, cabendo a coordenação e as normas gerais à esfera federal e a coordenação e a execução dos respectivos programas às esferas estadual e municipal, bem como a entidades beneficentes e de assistência social;

II - participação da população, por meio de organizações representativas, na formulação das políticas e no controle das ações em todos os níveis.

Parágrafo único. É facultado aos Estados e ao Distrito Federal vincular a programa de apoio à inclusão e promoção social até cinco décimos por cento de sua receita tributária líquida, vedada a aplicação desses recursos no pagamento de: (Incluído pela Emenda Constitucional nº 42, de 19.12.2003)

I - despesas com pessoal e encargos sociais; (Incluído pela Emenda Constitucional nº 42, de 19.12.2003)

II - serviço da dívida; (Incluído pela Emenda Constitucional nº 42, de 19.12.2003)

III - qualquer outra despesa corrente não vinculada diretamente aos investimentos ou ações apoiados. (Incluído pela Emenda Constitucional nº 42, de 19.12.2003)

CAPÍTULO III
DA EDUCAÇÃO, DA CULTURA E DO DESPORTO
SEÇÃO I
DA EDUCAÇÃO

Art. 205. A educação, direito de todos e dever do Estado e da família, será promovida e incentivada com a colaboração da sociedade, visando ao pleno desenvolvimento da pessoa, seu preparo para o exercício da cidadania e sua qualificação para o trabalho.

Art. 206. O ensino será ministrado com base nos seguintes princípios:

I - igualdade de condições para o acesso e permanência na escola;

II - liberdade de aprender, ensinar, pesquisar e divulgar o pensamento, a arte e o saber;

III - pluralismo de idéias e de concepções pedagógicas, e coexistência de instituições públicas e privadas de ensino;

IV - gratuidade do ensino público em estabelecimentos oficiais;

V - valorização dos profissionais da educação escolar, garantidos, na forma da lei, planos de carreira, com ingresso exclusivamente por concurso público de provas e títulos, aos das redes públicas; (Redação dada pela Emenda Constitucional nº 53, de 2006)

VI - gestão democrática do ensino público, na forma da lei;

VII - garantia de padrão de qualidade.

VIII - piso salarial profissional nacional para os profissionais da educação escolar pública, nos termos de lei federal. (Incluído pela Emenda Constitucional nº 53, de 2006)

Parágrafo único. A lei disporá sobre as categorias de trabalhadores considerados profissionais da educação básica e sobre a fixação de prazo para a elaboração ou adequação de seus planos de carreira, no âmbito da União, dos Estados, do Distrito Federal e dos Municípios. (Incluído pela Emenda Constitucional nº 53, de 2006)

Art. 207. As universidades gozam de autonomia didático-científica, administrativa e de gestão financeira e patrimonial, e obedecerão ao princípio de indissociabilidade entre ensino, pesquisa e extensão.

§ 1º É facultado às universidades admitir professores, técnicos e cientistas estrangeiros, na forma da lei. (Incluído pela Emenda Constitucional nº 11, de 1996)

§ 2º O disposto neste artigo aplica-se às instituições de pesquisa científica e tecnológica. (Incluído pela Emenda Constitucional nº 11, de 1996)

Art. 208. O dever do Estado com a educação será efetivado mediante a garantia de:

I - educação básica obrigatória e gratuita dos 4 (quatro) aos 17 (dezessete) anos de idade, assegurada inclusive sua oferta gratuita para todos os que a ela não tiveram acesso na idade própria; (Redação dada pela Emenda Constitucional nº 59, de 2009) (Vide Emenda Constitucional nº 59, de 2009)

II - progressiva universalização do ensino médio gratuito; (Redação dada pela Emenda Constitucional nº 14, de 1996)

III - atendimento educacional especializado aos portadores de deficiência, preferencialmente na rede regular de ensino;

IV - educação infantil, em creche e pré-escola, às crianças até 5 (cinco) anos de idade; (Redação dada pela Emenda Constitucional nº 53, de 2006)

V - acesso aos níveis mais elevados do ensino, da pesquisa e da criação artística, segundo a capacidade de cada um;

VI - oferta de ensino noturno regular, adequado às condições do educando;

VII - atendimento ao educando, em todas as etapas da educação básica, por meio de programas suplementares de material didáticoescolar, transporte, alimentação e assistência à saúde. (Redação dada pela Emenda Constitucional nº 59, de 2009)

§ 1º O acesso ao ensino obrigatório e gratuito é direito público subjetivo.

§ 2º O não-oferecimento do ensino obrigatório pelo Poder Público, ou sua oferta irregular, importa responsabilidade da autoridade competente.

§ 3º Compete ao Poder Público recensear os educandos no ensino fundamental, fazer-lhes a chamada e zelar, junto aos pais ou responsáveis, pela freqüência à escola.

Art. 209. O ensino é livre à iniciativa privada, atendidas as seguintes condições:

I - cumprimento das normas gerais da educação nacional;

II - autorização e avaliação de qualidade pelo Poder Público.

Art. 210. Serão fixados conteúdos mínimos para o ensino fundamental, de maneira a assegurar formação básica comum e respeito aos valores culturais e artísticos, nacionais e regionais.

§ 1º O ensino religioso, de matrícula facultativa, constituirá disciplina dos horários normais das escolas públicas de ensino fundamental.

§ 2º O ensino fundamental regular será ministrado em língua portuguesa, assegurada às comunidades indígenas também a utilização de suas línguas maternas e processos próprios de aprendizagem.

Art. 211. A União, os Estados, o Distrito Federal e os Municípios organizarão em regime de colaboração seus sistemas de ensino.

§ 1º A União organizará o sistema federal de ensino e o dos Territórios, financiará as instituições de ensino públicas federais e exercerá, em matéria educacional, função redistributiva e supletiva, de forma a garantir equalização de oportunidades educacionais e padrão mínimo de qualidade do ensino mediante assistência técnica e financeira aos Estados, ao Distrito Federal e aos Municípios; (Redação dada pela Emenda Constitucional nº 14, de 1996)

§ 2º Os Municípios atuarão prioritariamente no ensino fundamental e na educação infantil. (Redação dada pela Emenda Constitucional nº 14, de 1996)

§ 3º Os Estados e o Distrito Federal atuarão prioritariamente no ensino fundamental e médio. (Incluído pela Emenda Constitucional nº 14, de 1996)

§ 4º Na organização de seus sistemas de ensino, a União, os Estados, o Distrito Federal e os Municípios definirão formas de colaboração, de modo a assegurar a universalização do ensino obrigatório. (Redação dada pela Emenda Constitucional nº 59, de 2009)

§ 5º A educação básica pública atenderá prioritariamente ao ensino regular. (Incluído pela Emenda Constitucional nº 53, de 2006)

Art. 212. A União aplicará, anualmente, nunca menos de dezoito, e os Estados, o Distrito Federal e os Municípios vinte e cinco por cento, no mínimo, da receita resultante de impostos, compreendida a proveniente de transferências, na manutenção e desenvolvimento do ensino.

§ 1º A parcela da arrecadação de impostos transferida pela União aos Estados, ao Distrito Federal e aos Municípios, ou pelos Estados aos respectivos Municípios, não é considerada, para efeito do cálculo previsto neste artigo, receita do governo que a transferir.

§ 2º Para efeito do cumprimento do disposto no "caput" deste artigo, serão considerados os sistemas de ensino federal, estadual e municipal e os recursos aplicados na forma do art. 213.

§ 3º A distribuição dos recursos públicos assegurará prioridade ao atendimento das necessidades do ensino obrigatório, no que se refere a universalização, garantia de padrão de qualidade e equidade, nos termos do plano nacional de educação. (Redação dada pela Emenda Constitucional nº 59, de 2009)

§ 4º Os programas suplementares de alimentação e assistência à saúde previstos no art. 208, VII, serão financiados com recursos provenientes de contribuições sociais e outros recursos orçamentários.

§ 5º A educação básica pública terá como fonte adicional de financiamento a contribuição social do salário-educação, recolhida pelas empresas na forma da lei. (Redação dada pela Emenda Constitucional nº 53, de 2006) (Vide Decreto nº 6.003, de 2006)

§ 6º As cotas estaduais e municipais da arrecadação da contribuição social do salário-educação serão distribuídas proporcionalmente ao número de alunos matriculados na educação básica nas respectivas redes públicas de ensino. (Incluído pela Emenda Constitucional nº 53, de 2006)

Art. 213. Os recursos públicos serão destinados às escolas públicas, podendo ser dirigidos a escolas comunitárias, confessionais ou filantrópicas, definidas em lei, que:

I - comprovem finalidade não-lucrativa e apliquem seus excedentes financeiros em educação;

II - assegurem a destinação de seu patrimônio a outra escola comunitária, filantrópica ou confessional, ou ao Poder Público, no caso de encerramento de suas atividades.

§ 1º Os recursos de que trata este artigo poderão ser destinados a bolsas de estudo para o ensino fundamental e médio, na forma da lei, para os que demonstrarem insuficiência de recursos, quando houver falta de vagas e cursos regulares da rede pública na localidade da residência do educando, ficando o Poder Público obrigado a investir prioritariamente na expansão de sua rede na localidade.

§ 2º As atividades de pesquisa, de extensão e de estímulo e fomento à inovação realizadas por universidades e/ou por instituições de educação profissional e tecnológica poderão receber apoio financeiro do Poder Público. (Redação dada pela Emenda Constitucional nº 85, de 2015)

Art. 214. A lei estabelecerá o plano nacional de educação, de duração decenal, com o objetivo de articular o sistema nacional de educação em regime de colaboração e definir diretrizes, objetivos, metas e estratégias de implementação para assegurar a manutenção e desenvolvimento do ensino em seus diversos níveis, etapas e modalidades por meio de ações integradas dos poderes públicos das diferentes esferas federativas que conduzam a: (Redação dada pela Emenda Constitucional nº 59, de 2009)

I - erradicação do analfabetismo;

II - universalização do atendimento escolar;

III - melhoria da qualidade do ensino;

IV - formação para o trabalho;

V - promoção humanística, científica e tecnológica do País.

VI - estabelecimento de meta de aplicação de recursos públicos em educação como proporção do produto interno bruto. (Incluído pela Emenda Constitucional nº 59, de 2009)

SEÇÃO II
DA CULTURA

Art. 215. O Estado garantirá a todos o pleno exercício dos direitos culturais e acesso às fontes da cultura nacional, e apoiará e incentivará a valorização e a difusão das manifestações culturais.

§ 1º O Estado protegerá as manifestações das culturas populares, indígenas e afro-brasileiras, e das de outros grupos participantes do processo civilizatório nacional.

§ 2º A lei disporá sobre a fixação de datas comemorativas de alta significação para os diferentes segmentos étnicos nacionais.

§ 3º A lei estabelecerá o Plano Nacional de Cultura, de duração plurianual, visando ao desenvolvimento cultural do País e à integração das ações do poder público que conduzem à: (Incluído pela Emenda Constitucional nº 48, de 2005)

I defesa e valorização do patrimônio cultural brasileiro; (Incluído pela Emenda Constitucional nº 48, de 2005)

II produção, promoção e difusão de bens culturais; (Incluído pela Emenda Constitucional nº 48, de 2005)

III formação de pessoal qualificado para a gestão da cultura em suas múltiplas dimensões; (Incluído pela Emenda Constitucional nº 48, de 2005)

IV democratização do acesso aos bens de cultura; (Incluído pela Emenda Constitucional nº 48, de 2005)

V valorização da diversidade étnica e regional. (Incluído pela Emenda Constitucional nº 48, de 2005)

Art. 216. Constituem patrimônio cultural brasileiro os bens de natureza material e imaterial, tomados individualmente ou em conjunto, portadores de referência à identidade, à ação, à memória dos diferentes grupos formadores da sociedade brasileira, nos quais se incluem:

I - as formas de expressão;

II - os modos de criar, fazer e viver;

III - as criações científicas, artísticas e tecnológicas;

IV - as obras, objetos, documentos, edificações e demais espaços destinados às manifestações artístico-culturais;

V - os conjuntos urbanos e sítios de valor histórico, paisagístico, artístico, arqueológico, paleontológico, ecológico e científico.

§ 1º O Poder Público, com a colaboração da comunidade, promoverá e protegerá o patrimônio cultural brasileiro, por meio de inventários, registros, vigilância, tombamento e desapropriação, e de outras formas de acautelamento e preservação.

§ 2º Cabem à administração pública, na forma da lei, a gestão da documentação governamental e as providências para franquear sua consulta a quantos dela necessitem. (Vide Lei nº 12.527, de 2011)

§ 3º A lei estabelecerá incentivos para a produção e o conhecimento de bens e valores culturais.

§ 4º Os danos e ameaças ao patrimônio cultural serão punidos, na forma da lei.

§ 5º Ficam tombados todos os documentos e os sítios detentores de reminiscências históricas dos antigos quilombos.

§ 6 º É facultado aos Estados e ao Distrito Federal vincular a fundo estadual de fomento à cultura até cinco décimos por cento de sua receita tributária líquida, para o financiamento de programas e projetos culturais, vedada a aplicação desses recursos no pagamento de: (Incluído pela Emenda Constitucional nº 42, de 19.12.2003)

I - despesas com pessoal e encargos sociais; (Incluído pela Emenda Constitucional nº 42, de 19.12.2003)

II - serviço da dívida; (Incluído pela Emenda Constitucional nº 42, de 19.12.2003)

III - qualquer outra despesa corrente não vinculada diretamente aos investimentos ou ações apoiados. (Incluído pela Emenda Constitucional nº 42, de 19.12.2003)

Art. 216-A. O Sistema Nacional de Cultura, organizado em regime de colaboração, de forma descentralizada e participativa, institui um processo de gestão e promoção conjunta de políticas públicas de cultura, democráticas e permanentes, pactuadas entre os entes da Federação e a sociedade, tendo por objetivo promover o desenvolvimento humano, social e econômico com pleno exercício dos direitos culturais. (Incluído pela Emenda Constitucional nº 71, de 2012)

§ 1º O Sistema Nacional de Cultura fundamenta-se na política nacional de cultura e nas suas diretrizes, estabelecidas no Plano Nacional de Cultura, e rege-se pelos seguintes princípios: (Incluído pela Emenda Constitucional nº 71, de 2012)

I - diversidade das expressões culturais; (Incluído pela Emenda Constitucional nº 71, de 2012)

II - universalização do acesso aos bens e serviços culturais; (Incluído pela Emenda Constitucional nº 71, de 2012)

III - fomento à produção, difusão e circulação de conhecimento e bens culturais; (Incluído pela Emenda Constitucional nº 71, de 2012)

IV - cooperação entre os entes federados, os agentes públicos e privados atuantes na área cultural; (Incluído pela Emenda Constitucional nº 71, de 2012)

V - integração e interação na execução das políticas, programas, projetos e ações desenvolvidas; (Incluído pela Emenda Constitucional nº 71, de 2012)

VI - complementaridade nos papéis dos agentes culturais; (Incluído pela Emenda Constitucional nº 71, de 2012)

VII - transversalidade das políticas culturais; (Incluído pela Emenda Constitucional nº 71, de 2012)

VIII - autonomia dos entes federados e das instituições da sociedade civil; (Incluído pela Emenda Constitucional nº 71, de 2012)

IX - transparência e compartilhamento das informações; (Incluído pela Emenda Constitucional nº 71, de 2012)

X - democratização dos processos decisórios com participação e controle social; (Incluído pela Emenda Constitucional nº 71, de 2012)

XI - descentralização articulada e pactuada da gestão, dos recursos e das ações; (Incluído pela Emenda Constitucional nº 71, de 2012)

XII - ampliação progressiva dos recursos contidos nos orçamentos públicos para a cultura. (Incluído pela Emenda Constitucional nº 71, de 2012)

§ 2º Constitui a estrutura do Sistema Nacional de Cultura, nas respectivas esferas da Federação: (Incluído pela Emenda Constitucional nº 71, de 2012)

I - órgãos gestores da cultura; (Incluído pela Emenda Constitucional nº 71, de 2012)

II - conselhos de política cultural; (Incluído pela Emenda Constitucional nº 71, de 2012)

III - conferências de cultura; (Incluído pela Emenda Constitucional nº 71, de 2012)

IV - comissões intergestores; (Incluído pela Emenda Constitucional nº 71, de 2012)

V - planos de cultura; (Incluído pela Emenda Constitucional nº 71, de 2012)

VI - sistemas de financiamento à cultura; (Incluído pela Emenda Constitucional nº 71, de 2012)

VII - sistemas de informações e indicadores culturais; (Incluído pela Emenda Constitucional nº 71, de 2012)

VIII - programas de formação na área da cultura; e (Incluído pela Emenda Constitucional nº 71, de 2012)

IX - sistemas setoriais de cultura. (Incluído pela Emenda Constitucional nº 71, de 2012)

§ 3º Lei federal disporá sobre a regulamentação do Sistema Nacional de Cultura, bem como de sua articulação com os demais sistemas nacionais ou políticas setoriais de governo. (Incluído pela Emenda Constitucional nº 71, de 2012)

§ 4º Os Estados, o Distrito Federal e os Municípios organizarão seus respectivos sistemas de cultura em leis próprias. (Incluído pela Emenda Constitucional nº 71, de 2012)

SEÇÃO III
DO DESPORTO

Art. 217. É dever do Estado fomentar práticas desportivas formais e não-formais, como direito de cada um, observados:

I - a autonomia das entidades desportivas dirigentes e associações, quanto a sua organização e funcionamento;

II - a destinação de recursos públicos para a promoção prioritária do desporto educacional e, em casos específicos, para a do desporto de alto rendimento;

III - o tratamento diferenciado para o desporto profissional e o não- profissional;

IV - a proteção e o incentivo às manifestações desportivas de criação nacional.

§ 1º O Poder Judiciário só admitirá ações relativas à disciplina e às competições desportivas após esgotarem-se as instâncias da justiça desportiva, regulada em lei.

§ 2º A justiça desportiva terá o prazo máximo de sessenta dias, contados da instauração do processo, para proferir decisão final.

§ 3º O Poder Público incentivará o lazer, como forma de promoção social.

CAPÍTULO IV
DA CIÊNCIA, TECNOLOGIA E INOVAÇÃO
(Redação dada pela Emenda Constitucional nº 85, de 2015)

Art. 218. O Estado promoverá e incentivará o desenvolvimento científico, a pesquisa, a capacitação científica e tecnológica e a inovação. (Redação dada pela Emenda Constitucional nº 85, de 2015)

§ 1º A pesquisa científica básica e tecnológica receberá tratamento prioritário do Estado, tendo em vista o bem público e o progresso da ciência, tecnologia e inovação. (Redação dada pela Emenda Constitucional nº 85, de 2015)

§ 2º A pesquisa tecnológica voltar-se-á preponderantemente para a solução dos problemas brasileiros e para o desenvolvimento do sistema produtivo nacional e regional.

§ 3º O Estado apoiará a formação de recursos humanos nas áreas de ciência, pesquisa, tecnologia e inovação, inclusive por meio do apoio às atividades de extensão tecnológica, e concederá aos que delas se ocupem meios e condições especiais de trabalho. (Redação dada pela Emenda Constitucional nº 85, de 2015)

§ 4º A lei apoiará e estimulará as empresas que invistam em pesquisa, criação de tecnologia adequada ao País, formação e aperfeiçoamento de seus recursos humanos e que pratiquem sistemas de remuneração que assegurem ao empregado, desvinculada do salário, participação nos ganhos econômicos resultantes da produtividade de seu trabalho.

§ 5º É facultado aos Estados e ao Distrito Federal vincular parcela de sua receita orçamentária a entidades públicas de fomento ao ensino e à pesquisa científica e tecnológica.

§ 6º O Estado, na execução das atividades previstas no caput , estimulará a articulação entre entes, tanto públicos quanto privados, nas diversas esferas de governo. (Incluído pela Emenda Constitucional nº 85, de 2015)

§ 7º O Estado promoverá e incentivará a atuação no exterior das instituições públicas de ciência, tecnologia e inovação, com vistas à execução das atividades previstas no caput. (Incluído pela Emenda Constitucional nº 85, de 2015)

Art. 219. O mercado interno integra o patrimônio nacional e será incentivado de modo a viabilizar o desenvolvimento cultural e sócio-econômico, o bem-estar da população e a autonomia tecnológica do País, nos termos de lei federal.

Parágrafo único. O Estado estimulará a formação e o fortalecimento da inovação nas empresas, bem como nos demais entes, públicos ou privados, a constituição e a manutenção de parques e polos tecnológicos e de demais ambientes promotores da inovação, a atuação dos inventores independentes e a criação, absorção, difusão e transferência de tecnologia. (Incluído pela Emenda Constitucional nº 85, de 2015)

Art. 219-A. A União, os Estados, o Distrito Federal e os Municípios poderão firmar instrumentos de cooperação com órgãos e entidades públicos e com entidades privadas, inclusive para o compartilhamento de recursos humanos especializados e capacidade instalada, para a execução de projetos de pesquisa, de desenvolvimento científico e tecnológico e de inovação, mediante contrapartida financeira ou não financeira assumida pelo ente beneficiário, na forma da lei. (Incluído pela Emenda Constitucional nº 85, de 2015)

Art. 219-B. O Sistema Nacional de Ciência, Tecnologia e Inovação (SNCTI) será organizado em regime de colaboração entre entes, tanto públicos quanto privados, com vistas a promover o desenvolvimento científico e tecnológico e a inovação. (Incluído pela Emenda Constitucional nº 85, de 2015)

§ 1º Lei federal disporá sobre as normas gerais do SNCTI. (Incluído pela Emenda Constitucional nº 85, de 2015)

§ 2º Os Estados, o Distrito Federal e os Municípios legislarão concorrentemente sobre suas peculiaridades. (Incluído pela Emenda Constitucional nº 85, de 2015)

CAPÍTULO V
DA COMUNICAÇÃO SOCIAL

Art. 220. A manifestação do pensamento, a criação, a expressão e a informação, sob qualquer forma, processo ou veículo não sofrerão qualquer restrição, observado o disposto nesta Constituição.

§ 1º Nenhuma lei conterá dispositivo que possa constituir embaraço à plena liberdade de informação jornalística em qualquer veículo de comunicação social, observado o disposto no art. 5º, IV, V, X, XIII e XIV.

§ 2º É vedada toda e qualquer censura de natureza política, ideológica e artística.

§ 3º Compete à lei federal:

I - regular as diversões e espetáculos públicos, cabendo ao Poder Público informar sobre a natureza deles, as faixas etárias a que não se recomendem, locais e horários em que sua apresentação se mostre inadequada;

II - estabelecer os meios legais que garantam à pessoa e à família a possibilidade de se defenderem de programas ou programações de rádio e televisão que contrariem o disposto no art. 221, bem como da propaganda de produtos, práticas e serviços que possam ser nocivos à saúde e ao meio ambiente.

§ 4º A propaganda comercial de tabaco, bebidas alcoólicas, agrotóxicos, medicamentos e terapias estará sujeita a restrições legais, nos termos do inciso II do parágrafo anterior, e conterá, sempre que necessário, advertência sobre os malefícios decorrentes de seu uso.

§ 5º Os meios de comunicação social não podem, direta ou indiretamente, ser objeto de monopólio ou oligopólio.

§ 6º A publicação de veículo impresso de comunicação independe de licença de autoridade.

Art. 221. A produção e a programação das emissoras de rádio e televisão atenderão aos seguintes princípios:

I - preferência a finalidades educativas, artísticas, culturais e informativas;

II - promoção da cultura nacional e regional e estímulo à produção independente que objetive sua divulgação;

III - regionalização da produção cultural, artística e jornalística, conforme percentuais estabelecidos em lei;

IV - respeito aos valores éticos e sociais da pessoa e da família.

Art. 222. A propriedade de empresa jornalística e de radiodifusão sonora e de sons e imagens é privativa de brasileiros natos ou naturalizados há mais de dez anos, ou de pessoas jurídicas constituídas sob as leis brasileiras e que tenham sede no País. (Redação dada pela Emenda Constitucional nº 36, de 2002)

§ 1º Em qualquer caso, pelo menos setenta por cento do capital total e do capital votante das empresas jornalísticas e de radiodifusão sonora e de sons e imagens deverá pertencer, direta ou indiretamente, a brasileiros natos ou naturalizados há mais de dez anos, que exercerão

obrigatoriamente a gestão das atividades e estabelecerão o conteúdo da programação. (Redação dada pela Emenda Constitucional nº 36, de 2002)

§ 2º A responsabilidade editorial e as atividades de seleção e direção da programação veiculada são privativas de brasileiros natos ou naturalizados há mais de dez anos, em qualquer meio de comunicação social. (Redação dada pela Emenda Constitucional nº 36, de 2002)

§ 3º Os meios de comunicação social eletrônica, independentemente da tecnologia utilizada para a prestação do serviço, deverão observar os princípios enunciados no art. 221, na forma de lei específica, que também garantirá a prioridade de profissionais brasileiros na execução de produções nacionais. (Incluído pela Emenda Constitucional nº 36, de 2002)

§ 4º Lei disciplinará a participação de capital estrangeiro nas empresas de que trata o § 1º. (Incluído pela Emenda Constitucional nº 36, de 2002)

§ 5º As alterações de controle societário das empresas de que trata o § 1º serão comunicadas ao Congresso Nacional. (Incluído pela Emenda Constitucional nº 36, de 2002)

Art. 223. Compete ao Poder Executivo outorgar e renovar concessão, permissão e autorização para o serviço de radiodifusão sonora e de sons e imagens, observado o princípio da complementaridade dos sistemas privado, público e estatal.

§ 1º O Congresso Nacional apreciará o ato no prazo do art. 64, § 2º e § 4º, a contar do recebimento da mensagem.

§ 2º A não renovação da concessão ou permissão dependerá de aprovação de, no mínimo, dois quintos do Congresso Nacional, em votação nominal.

§ 3º O ato de outorga ou renovação somente produzirá efeitos legais após deliberação do Congresso Nacional, na forma dos parágrafos anteriores.

§ 4º O cancelamento da concessão ou permissão, antes de vencido o prazo, depende de decisão judicial.

§ 5º O prazo da concessão ou permissão será de dez anos para as emissoras de rádio e de quinze para as de televisão.

Art. 224. Para os efeitos do disposto neste capítulo, o Congresso Nacional instituirá, como seu órgão auxiliar, o Conselho de Comunicação Social, na forma da lei.

CAPÍTULO VI
DO MEIO AMBIENTE

Art. 225. Todos têm direito ao meio ambiente ecologicamente equilibrado, bem de uso comum do povo e essencial à sadia qualidade de vida, impondo-se ao Poder Público e à coletividade o dever de defendê-lo e preservá- lo para as presentes e futuras gerações.

§ 1º Para assegurar a efetividade desse direito, incumbe ao Poder Público:

I - preservar e restaurar os processos ecológicos essenciais e prover o manejo ecológico das espécies e ecossistemas; (Regulamento)

II - preservar a diversidade e a integridade do patrimônio genético do País e fiscalizar as entidades dedicadas à pesquisa e manipulação de material genético; (Regulamento) (Regulamento) (Regulamento) (Regulamento)

III - definir, em todas as unidades da Federação, espaços territoriais e seus componentes a serem especialmente protegidos, sendo a alteração e a supressão permitidas somente através de lei, vedada qualquer utilização que comprometa a integridade dos atributos que justifiquem sua proteção; (Regulamento)

IV - exigir, na forma da lei, para instalação de obra ou atividade potencialmente causadora de significativa degradação do meio ambiente, estudo prévio de impacto ambiental, a que se dará publicidade; (Regulamento)

V - controlar a produção, a comercialização e o emprego de técnicas, métodos e substâncias que comportem risco para a vida, a qualidade de vida e o meio ambiente; (Regulamento)

VI - promover a educação ambiental em todos os níveis de ensino e a conscientização pública para a preservação do meio ambiente;

VII - proteger a fauna e a flora, vedadas, na forma da lei, as práticas que coloquem em risco sua função ecológica, provoquem a extinção de espécies ou submetam os animais a crueldade. (Regulamento)

§ 2º Aquele que explorar recursos minerais fica obrigado a recuperar o meio ambiente degradado, de acordo com solução técnica exigida pelo órgão público competente, na forma da lei.

§ 3º As condutas e atividades consideradas lesivas ao meio ambiente sujeitarão os infratores, pessoas físicas ou jurídicas, a sanções penais e administrativas, independentemente da obrigação de reparar os danos causados.

§ 4º A Floresta Amazônica brasileira, a Mata Atlântica, a Serra do Mar, o Pantanal Mato-Grossense e a Zona Costeira são patrimônio nacional, e sua utilização far-se-á, na forma da lei, dentro de condições que assegurem a preservação do meio ambiente, inclusive quanto ao uso dos recursos naturais. (Regulamento) (Regulamento)

§ 5º São indisponíveis as terras devolutas ou arrecadadas pelos Estados, por ações discriminatórias, necessárias à proteção dos ecossistemas naturais.

§ 6º As usinas que operem com reator nuclear deverão ter sua localização definida em lei federal, sem o que não poderão ser instaladas.

§ 7º Para fins do disposto na parte final do inciso VII do § 1º deste artigo, não se consideram cruéis as práticas desportivas que utilizem animais, desde que sejam manifestações culturais, conforme o § 1º do art. 215 desta Constituição Federal, registradas como bem de natureza imaterial integrante do patrimônio cultural brasileiro, devendo ser regulamentadas por lei específica que assegure o bem-estar dos animais envolvidos. (Incluído pela Emenda Constitucional nº 96, de 2017)

CAPÍTULO VII
Da Família, da Criança, do Adolescente, do Jovem e do Idoso

(Redação dada Pela Emenda Constitucional nº 65, de 2010)

Art. 226. A família, base da sociedade, tem especial proteção do Estado.

§ 1º O casamento é civil e gratuita a celebração.

§ 2º O casamento religioso tem efeito civil, nos termos da lei.

§ 3º Para efeito da proteção do Estado, é reconhecida a união estável entre o homem e a mulher como entidade familiar, devendo a lei facilitar sua conversão em casamento. (Regulamento)

§ 4º Entende-se, também, como entidade familiar a comunidade formada por qualquer dos pais e seus descendentes.

§ 5º Os direitos e deveres referentes à sociedade conjugal são exercidos igualmente pelo homem e pela mulher.

§ 6º O casamento civil pode ser dissolvido pelo divórcio. (Redação dada Pela Emenda Constitucional nº 66, de 2010)

§ 7º Fundado nos princípios da dignidade da pessoa humana e da paternidade responsável, o planejamento familiar é livre decisão do casal, competindo ao Estado propiciar recursos educacionais e científicos para o exercício desse direito, vedada qualquer forma coercitiva por parte de instituições oficiais ou privadas. Regulamento

§ 8º O Estado assegurará a assistência à família na pessoa de cada um dos que a integram, criando mecanismos para coibir a violência no âmbito de suas relações.

Art. 227. É dever da família, da sociedade e do Estado assegurar à criança, ao adolescente e ao jovem, com absoluta prioridade, o direito à vida, à saúde, à alimentação, à educação, ao lazer, à profissionalização, à cultura, à dignidade, ao respeito, à liberdade e à convivência familiar e comunitária, além de colocá-los a salvo de toda forma de negligência, discriminação, exploração, violência, crueldade e opressão. (Redação dada Pela Emenda Constitucional nº 65, de 2010)

§ 1º O Estado promoverá programas de assistência integral à saúde da criança, do adolescente e do jovem, admitida a participação de entidades não governamentais, mediante políticas específicas e obedecendo aos seguintes preceitos: (Redação dada Pela Emenda Constitucional nº 65, de 2010)

I - aplicação de percentual dos recursos públicos destinados à saúde na assistência materno-infantil;

II - criação de programas de prevenção e atendimento especializado para as pessoas portadoras de deficiência física, sensorial ou mental, bem como de integração social do adolescente e do jovem portador de deficiência, mediante o treinamento para o trabalho e a convivência, e a facilitação do acesso aos bens e serviços coletivos, com a eliminação de obstáculos arquitetônicos e de todas as formas de discriminação. (Redação dada Pela Emenda Constitucional nº 65, de 2010)

§ 2º A lei disporá sobre normas de construção dos logradouros e dos edifícios de uso público e de fabricação de veículos de transporte coletivo, a fim de garantir acesso adequado às pessoas portadoras de deficiência.

§ 3º O direito a proteção especial abrangerá os seguintes aspectos:

I - idade mínima de quatorze anos para admissão ao trabalho, observado o disposto no art. 7º, XXXIII;

II - garantia de direitos previdenciários e trabalhistas;

III - garantia de acesso do trabalhador adolescente e jovem à escola; (Redação dada Pela Emenda Constitucional nº 65, de 2010)

IV - garantia de pleno e formal conhecimento da atribuição de ato infracional, igualdade na relação processual e defesa técnica por profissional habilitado, segundo dispuser a legislação tutelar específica;

V - obediência aos princípios de brevidade, excepcionalidade e respeito à condição peculiar de pessoa em desenvolvimento, quando da aplicação de qualquer medida privativa da liberdade;

VI - estímulo do Poder Público, através de assistência jurídica, incentivos fiscais e subsídios, nos termos da lei, ao acolhimento, sob a forma de guarda, de criança ou adolescente órfão ou abandonado;

VII - programas de prevenção e atendimento especializado à criança, ao adolescente e ao jovem dependente de entorpecentes e drogas afins. (Redação dada Pela Emenda Constitucional nº 65, de 2010)

§ 4º A lei punirá severamente o abuso, a violência e a exploração sexual da criança e do adolescente.

§ 5º A adoção será assistida pelo Poder Público, na forma da lei, que estabelecerá casos e condições de sua efetivação por parte de estrangeiros.

§ 6º Os filhos, havidos ou não da relação do casamento, ou por adoção, terão os mesmos direitos e qualificações, proibidas quaisquer designações discriminatórias relativas à filiação.

§ 7º No atendimento dos direitos da criança e do adolescente levar-se- á em consideração o disposto no art. 204.

§ 8º A lei estabelecerá: (Incluído Pela Emenda Constitucional nº 65, de 2010)

I - o estatuto da juventude, destinado a regular os direitos dos jovens; (Incluído Pela Emenda Constitucional nº 65, de 2010)

II - o plano nacional de juventude, de duração decenal, visando à articulação das várias esferas do poder público para a execução de políticas públicas. (Incluído Pela Emenda Constitucional nº 65, de 2010)

Art. 228. São penalmente inimputáveis os menores de dezoito anos, sujeitos às normas da legislação especial.

Art. 229. Os pais têm o dever de assistir, criar e educar os filhos menores, e os filhos maiores têm o dever de ajudar e amparar os pais na velhice, carência ou enfermidade.

Art. 230. A família, a sociedade e o Estado têm o dever de amparar as pessoas idosas, assegurando sua participação na comunidade, defendendo sua dignidade e bem-estar e garantindo-lhes o direito à vida.

§ 1º Os programas de amparo aos idosos serão executados preferencialmente em seus lares.

§ 2º Aos maiores de sessenta e cinco anos é garantida a gratuidade dos transportes coletivos urbanos.

CAPÍTULO VIII
DOS ÍNDIOS

Art. 231. São reconhecidos aos índios sua organização social, costumes, línguas, crenças e tradições, e os direitos originários sobre as terras que tradicionalmente ocupam, competindo à União demarcá-las, proteger e fazer respeitar todos os seus bens.

§ 1º São terras tradicionalmente ocupadas pelos índios as por eles habitadas em caráter permanente, as utilizadas para suas atividades produtivas, as imprescindíveis à preservação dos recursos ambientais necessários a seu bem-estar e as necessárias a sua reprodução física e cultural, segundo seus usos, costumes e tradições.

§ 2º As terras tradicionalmente ocupadas pelos índios destinam-se a sua posse permanente, cabendo-lhes o usufruto exclusivo das riquezas do solo, dos rios e dos lagos nelas existentes.

§ 3º O aproveitamento dos recursos hídricos, incluídos os potenciais energéticos, a pesquisa e a lavra das riquezas minerais em terras indígenas só podem ser efetivados com autorização do Congresso Nacional, ouvidas as comunidades afetadas, ficando-lhes assegurada participação nos resultados da lavra, na forma da lei.

§ 4º As terras de que trata este artigo são inalienáveis e indisponíveis, e os direitos sobre elas, imprescritíveis.

§ 5º É vedada a remoção dos grupos indígenas de suas terras, salvo, "ad referendum" do Congresso Nacional, em caso de catástrofe ou epidemia que ponha em risco sua população, ou no interesse da soberania do País, após deliberação do Congresso Nacional, garantido, em qualquer hipótese, o retorno imediato logo que cesse o risco.

§ 6º São nulos e extintos, não produzindo efeitos jurídicos, os atos que tenham por objeto a ocupação, o domínio e a posse das terras a que se refere este artigo, ou a exploração das riquezas naturais do solo, dos rios e dos lagos nelas existentes, ressalvado relevante interesse público da União, segundo o que dispuser lei complementar, não gerando a nulidade e a extinção direito a indenização ou a ações contra a União, salvo, na forma da lei, quanto às benfeitorias derivadas da ocupação de boa fé.

§ 7º Não se aplica às terras indígenas o disposto no art. 174, § 3º e § 4º.

Art. 232. Os índios, suas comunidades e organizações são partes legítimas para ingressar em juízo em defesa de seus direitos e interesses, intervindo o Ministério Público em todos os atos do processo.

TÍTULO IX
DAS DISPOSIÇÕES CONSTITUCIONAIS GERAIS

~~Art. 233. Para efeito do art. 7º, XXIX, o empregador rural comprovará, de cinco em cinco anos, perante a Justiça do Trabalho, o cumprimento das suas obrigações trabalhistas para com o empregado rural, na presença deste e de seu representante sindical.~~ (Revogado pela Emenda Constitucional nº 28, de 25/05/2000)

~~§ 1º Uma vez comprovado o cumprimento das obrigações mencionadas neste artigo, fica o empregador isento de qualquer ônus decorrente daquelas obrigações no período respectivo. Caso o empregado e seu representante não concordem com a comprovação do empregador, caberá à Justiça do Trabalho a solução da controvérsia.~~ (Revogado pela Emenda Constitucional nº 28, de 25/05/2000)

~~§ 2º Fica ressalvado ao empregado, em qualquer hipótese, o direito de postular, judicialmente, os créditos que entender existir, relativamente aos últimos cinco anos.~~ (Revogado pela Emenda Constitucional nº 28, de 25/05/2000)

~~§ 3º A comprovação mencionada neste artigo poderá ser feita em prazo inferior a cinco anos, a critério do empregador.~~ (Revogado pela Emenda Constitucional nº 28, de 25/05/2000)

Art. 234. É vedado à União, direta ou indiretamente, assumir, em decorrência da criação de Estado, encargos referentes a despesas com pessoal inativo e com encargos e amortizações da dívida interna ou externa da administração pública, inclusive da indireta.

Art. 235. Nos dez primeiros anos da criação de Estado, serão observadas as seguintes normas básicas:

I - a Assembléia Legislativa será composta de dezessete Deputados se a população do Estado for inferior a seiscentos mil habitantes, e de vinte e quatro, se igual ou superior a esse número, até um milhão e quinhentos mil;

II - o Governo terá no máximo dez Secretarias;

III - o Tribunal de Contas terá três membros, nomeados, pelo Governador eleito, dentre brasileiros de comprovada idoneidade e notório saber;

IV - o Tribunal de Justiça terá sete Desembargadores;

V - os primeiros Desembargadores serão nomeados pelo Governador eleito, escolhidos da seguinte forma:

a) cinco dentre os magistrados com mais de trinta e cinco anos de idade, em exercício na área do novo Estado ou do Estado originário;

b) dois dentre promotores, nas mesmas condições, e advogados de comprovada idoneidade e saber jurídico, com dez anos, no mínimo, de exercício profissional, obedecido o procedimento fixado na Constituição;

VI - no caso de Estado proveniente de Território Federal, os cinco primeiros Desembargadores poderão ser escolhidos dentre juízes de direito de qualquer parte do País;

VII - em cada Comarca, o primeiro Juiz de Direito, o primeiro Promotor de Justiça e o primeiro Defensor Público serão nomeados pelo Governador eleito após concurso público de provas e títulos;

VIII - até a promulgação da Constituição Estadual, responderão pela Procuradoria-Geral, pela Advocacia-Geral e pela Defensoria-Geral do Estado advogados de notório saber, com trinta e cinco anos de idade, no mínimo, nomeados pelo Governador eleito e demissíveis "ad nutum";

IX - se o novo Estado for resultado de transformação de Território Federal, a transferência de encargos financeiros da União para pagamento dos servidores optantes que pertenciam à Administração Federal ocorrerá da seguinte forma:

a) no sexto ano de instalação, o Estado assumirá vinte por cento dos encargos financeiros para fazer face ao pagamento dos servidores públicos, ficando ainda o restante sob a responsabilidade da União;

b) no sétimo ano, os encargos do Estado serão acrescidos de trinta por cento e, no oitavo, dos restantes cinqüenta por cento;

X - as nomeações que se seguirem às primeiras, para os cargos mencionados neste artigo, serão disciplinadas na Constituição Estadual;

XI - as despesas orçamentárias com pessoal não poderão ultrapassar cinqüenta por cento da receita do Estado.

Art. 236. Os serviços notariais e de registro são exercidos em caráter privado, por delegação do Poder Público. (Regulamento)

§ 1º Lei regulará as atividades, disciplinará a responsabilidade civil e criminal dos notários, dos oficiais de registro e de seus prepostos, e definirá a fiscalização de seus atos pelo Poder Judiciário.

§ 2º Lei federal estabelecerá normas gerais para fixação de emolumentos relativos aos atos praticados pelos serviços notariais e de registro. (Regulamento)

§ 3º O ingresso na atividade notarial e de registro depende de concurso público de provas e títulos, não se permitindo que qualquer serventia fique vaga, sem abertura de concurso de provimento ou de remoção, por mais de seis meses.

Art. 237. A fiscalização e o controle sobre o comércio exterior, essenciais à defesa dos interesses fazendários nacionais, serão exercidos pelo Ministério da Fazenda.

Art. 238. A lei ordenará a venda e revenda de combustíveis de petróleo, álcool carburante e outros combustíveis derivados de matérias-primas renováveis, respeitados os princípios desta Constituição.

Art. 239. A arrecadação decorrente das contribuições para o Programa de Integração Social, criado pela Lei Complementar nº 7, de 7 de setembro de 1970 , e para o Programa de Formação do Patrimônio do Servidor Público, criado pela Lei Complementar nº 8, de 3 de dezembro de 1970 , passa, a partir da promulgação desta Constituição, a financiar, nos termos que a lei dispuser, o programa do seguro-desemprego e o abono de que trata o § 3º deste artigo. (Regulamento)

§ 1º Dos recursos mencionados no "caput" deste artigo, pelo menos quarenta por cento serão destinados a financiar programas de desenvolvimento econômico, através do Banco Nacional de Desenvolvimento Econômico e Social, com critérios de remuneração que lhes preservem o valor.

§ 2º Os patrimônios acumulados do Programa de Integração Social e do Programa de Formação do Patrimônio do Servidor Público são preservados, mantendo-se os critérios de saque nas situações previstas nas leis específicas, com exceção da retirada por motivo de casamento, ficando vedada a distribuição da arrecadação de que trata o "caput" deste artigo, para depósito nas contas individuais dos participantes.

§ 3º Aos empregados que percebam de empregadores que contribuem para o Programa de Integração Social ou para o Programa de Formação do Patrimônio do Servidor Público, até dois salários mínimos de remuneração mensal, é assegurado o pagamento de um salário mínimo anual, computado neste valor o rendimento das contas individuais, no caso daqueles que já participavam dos referidos programas, até a data da promulgação desta Constituição.

§ 4º O financiamento do seguro-desemprego receberá uma contribuição adicional da empresa cujo índice de rotatividade da força de trabalho superar o índice médio da rotatividade do setor, na forma estabelecida por lei.

Art. 240. Ficam ressalvadas do disposto no art. 195 as atuais contribuições compulsórias dos empregadores sobre a folha de salários, destinadas às entidades privadas de serviço social e de formação profissional vinculadas ao sistema sindical.

Art. 241. A União, os Estados, o Distrito Federal e os Municípios disciplinarão por meio de lei os consórcios públicos e os convênios de cooperação entre os entes federados, autorizando a gestão associada de serviços públicos, bem como a transferência total ou parcial de encargos, serviços, pessoal e bens essenciais à continuidade dos serviços transferidos. (Redação dada pela Emenda Constitucional nº 19, de 1998)

Art. 242. O princípio do art. 206, IV, não se aplica às instituições educacionais oficiais criadas por lei estadual ou municipal e existentes na data da promulgação desta Constituição, que não sejam total ou preponderantemente mantidas com recursos públicos.

§ 1º O ensino da História do Brasil levará em conta as contribuições das diferentes culturas e etnias para a formação do povo brasileiro.

§ 2º O Colégio Pedro II, localizado na cidade do Rio de Janeiro, será mantido na órbita federal.

Art. 243. As propriedades rurais e urbanas de qualquer região do País onde forem localizadas culturas ilegais de plantas psicotrópicas ou a exploração de trabalho escravo na forma da lei serão expropriadas e destinadas à reforma agrária e a programas de habitação popular, sem qualquer indenização ao proprietário e sem prejuízo de outras sanções previstas em lei, observado, no que couber, o disposto no art. 5º. (Redação dada pela Emenda Constitucional nº 81, de 2014)

Parágrafo único. Todo e qualquer bem de valor econômico apreendido em decorrência do tráfico ilícito de entorpecentes e drogas afins e da exploração de trabalho escravo será confiscado e reverterá a fundo especial com destinação específica, na forma da lei. (Redação dada pela Emenda Constitucional nº 81, de 2014)

Art. 244. A lei disporá sobre a adaptação dos logradouros, dos edifícios de uso público e dos veículos de transporte coletivo atualmente existentes a fim de garantir acesso adequado às pessoas portadoras de deficiência, conforme o disposto no art. 227, § 2º.

Art. 245. A lei disporá sobre as hipóteses e condições em que o Poder Público dará assistência aos herdeiros e dependentes carentes de pessoas vitimadas por crime doloso, sem prejuízo da responsabilidade civil do autor do ilícito.

Art. 246. É vedada a adoção de medida provisória na regulamentação de artigo da Constituição cuja redação tenha sido alterada por meio de emenda promulgada entre 1º de janeiro de 1995 até a promulgação desta emenda, inclusive. (Redação dada pela Emenda Constitucional nº 32, de 2001)

Art. 247. As leis previstas no inciso III do § 1º do art. 41 e no § 7º do art. 169 estabelecerão critérios e garantias especiais para a perda do cargo pelo servidor público estável que, em decorrência das atribuições de seu cargo efetivo, desenvolva atividades exclusivas de Estado. (Incluído pela Emenda Constitucional nº 19, de 1998)

Parágrafo único. Na hipótese de insuficiência de desempenho, a perda do cargo somente ocorrerá mediante processo administrativo em que lhe sejam assegurados o contraditório e a ampla defesa. (Incluído pela Emenda Constitucional nº 19, de 1998)

Art. 248. Os benefícios pagos, a qualquer título, pelo órgão responsável pelo regime geral de previdência social, ainda que à conta do Tesouro Nacional, e os não sujeitos ao limite máximo de valor fixado para os benefícios concedidos por esse regime observarão os limites fixados no art. 37, XI. (Incluído pela Emenda Constitucional nº 20, de 1998)

Art. 249. Com o objetivo de assegurar recursos para o pagamento de proventos de aposentadoria e pensões concedidas aos respectivos servidores e seus dependentes, em adição aos recursos dos respectivos tesouros, a União, os Estados, o Distrito Federal e os Municípios poderão constituir fundos integrados pelos recursos provenientes de contribuições e por bens, direitos e ativos de qualquer natureza, mediante lei que disporá sobre a natureza e administração desses fundos. (Incluído pela Emenda Constitucional nº 20, de 1998)

Art. 250. Com o objetivo de assegurar recursos para o pagamento dos benefícios concedidos pelo regime geral de previdência social, em adição aos recursos de sua arrecadação,

a União poderá constituir fundo integrado por bens, direitos e ativos de qualquer natureza, mediante lei que disporá sobre a natureza e administração desse fundo. (Incluído pela Emenda Constitucional nº 20, de 1998)

Brasília, 5 de outubro de 1988.
