# Supplementary material for: Network analysis as an alternative way to interpret constitutions
Source: PLoS One. 2021 Nov 1;16(11):e0259461. doi: 10.1371/journal.pone.0259461 (PMC8559923; doi:10.1371/journal.pone.0259461)
Supplement: S3 File — Text update until 2020. (PDF) [file pone.0259461.s004.pdf]

FEDERAL SUPREME COURT

CONSTITUTION

of the Federative Republic of Brazil

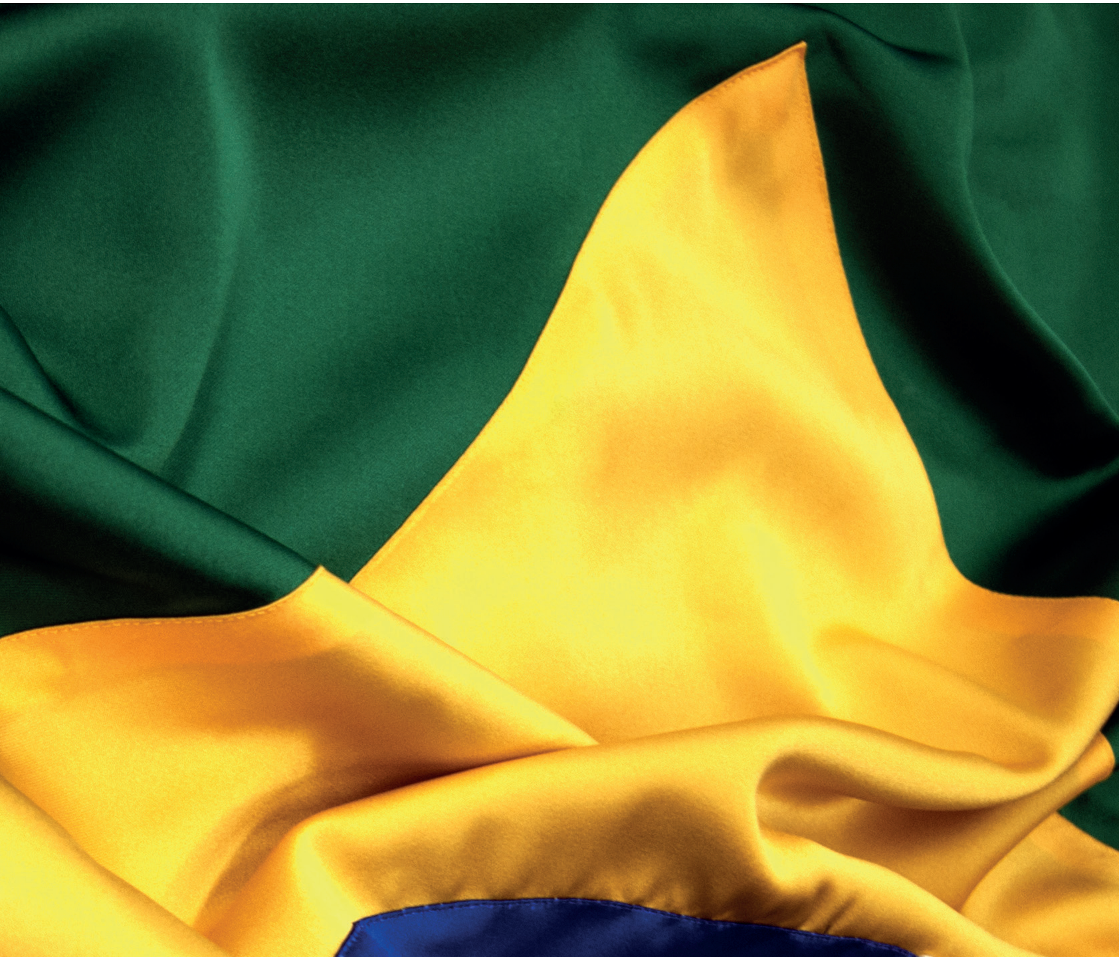

FEDERAL SUPREME COURT

Constitution of the Federative Republic of Brazil

Constitutional text enacted on October 5, 1988, with the alterations established by Revision Constitutional Amendments 1, 1994 through 6, 1994, and by Constitutional Amendments 1, 1992 through 106, 2020.

Brasília
2020

Office of the General Secretary to the Presidency
Daiane Nogueira de Lira

Office of Documentation
Naiara Cabeleira de Araújo Pichler

Office of Caselaw Analysis
Cícero Antônio Cavalcante de Araújo

Office of Institutional Memory and Document Management
Ana Paula Alencar Oliveira

Office of the Secretary to the Court
Eduardo Silva Toledo

International Affairs Office
Joel Souza Pinto Sampaio
André Nogueira Rangel Varanda Wollman

Office of Caselaw Dissemination
Flávia Carvalho Coelho Arlant

Proofreading
Ana Valéria de Oliveira Teixeira, Flávia Trigueiro Mendes Patriota, Joel Souza Pinto Sampaio e Milena Negrão de Miranda

Graphic Design Project
Eduardo Franco Dias

Layout
Camila Penha Soares

The English version of the Constitution of the Federative Republic of Brazil was extracted from the website of the Chamber of Deputies. The text of the amendments was translated by Felipe Justino de Farias and Flávia Trigueiro Mendes Patriota.

Dados Internacionais de Catalogação na Publicação (CIP)
(Supremo Tribunal Federal – Biblioteca Ministro Victor Nunes Leal)

Brasil.
[Constituição (1988)].
Constitution of the Federative Republic of Brazil. -- Brasília : STF, Secretaria de Documentação, 2020.

261 p.

Constitutional text enacted on October 5, 1988, with the alterations established by Revision Constitutional Amendments 1,1994 through 6, 1994, and Constitutional Amendments 1, 1992 through 106, 2020.

Disponível também em formato eletrônico: <http://www.stf.jus.br/arquivo/cms/legislacaoConstituicao/anexo/CF_ingles.pdf>

ISBN: 978-65-87125-02-2

1. Direito constitucional, legislação, Brasil. 2. Constituição, Brasil, 1988. 3. Emenda constitucional, Brasil. I. Título

CDDir-341.2481

FEDERAL SUPREME COURT

Justice José Antonio DIAS TOFFOLI (10-23-2009), Chief Justice

Justice LUIZ FUX (3-3-2011), Deputy Chief Justice

Justice José CELSO DE MELLO Filho (8-17-1989), Senior

Justice MARCO AURÉLIO Mendes de Farias Mello (6-13-1990)

Justice GILMAR Ferreira MENDES (6-20-2002)

Justice Enrique RICARDO LEWANDOWSKI (3-16-2006)

Justice CÁRMEN LÚCIA Antunes Rocha (6-21-2006)

Justice ROSA Maria Pires WEBER (12-19-2011)

Justice Luís ROBERTO BARROSO (6-26-2013)

Justice Luiz EDSON FACHIN (6-16-2015)

Justice ALEXANDRE DE MORAES (3-22-2017)

TABLE OF CONTENTS

Preamble 9

Title I – Fundamental Principles (Articles 1 to 4) 11

Title II – Fundamental Rights and Guarantees (Articles 5 to 17) 13
Chapter I – Individual and Collective Rights and Duties (Article 5) 13
Chapter II – Social Rights (Articles 6 to 11) 19
Chapter III – Nationality (Articles 12 and 13) 22
Chapter IV – Political Rights (Articles 14 to 16) 24
Chapter V – Political Parties (Article 17) 26

Title III – The Organization of the State (Articles 18 to 43) 28
Chapter I – The Political and Administrative Organization (Articles 18 and 19) 28
Chapter II – The Union (Articles 20 to 24) 29
Chapter III – The Federated States (Articles 25 to 28) 34
Chapter IV – The Municipalities (Articles 29 to 31) 36
Chapter V – The Federal District and the Territories (Articles 32 and 33) 41
Section I – The Federal District (Article 32) 41
Section II – The Territories (Article 33) 42
Chapter VI – Intervention (Articles 34 to 36) 42
Chapter VII – Public Administration (Articles 37 to 43) 44
Section I – General Provisions (Articles 37 and 38) 44
Section II – Government Employees (Articles 39 to 41) 49
Section III – The Military of the States, of the Federal District and of the Territories (Article 42) 55
Section IV – The Regions (Article 43) 55

Title IV – Organization of the Branches (Articles 44 to 135) 57
Chapter I – The Legislative Branch (Articles 44 to 75) 57
Section I – The National Congress (Articles 44 to 47) 57
Section II – Powers of the National Congress (Articles 48 to 50) 58
Section III – The Chamber of Deputies (Article 51) 60

Section IV – The Federal Senate (Article 52) 61
Section V – Deputies and Senators (Articles 53 to 56) 62
Section VI – The Sessions (Article 57) 65
Section VII – The Committees (Article 58) 66
Section VIII – The Legislative Process (Articles 59 to 69) 67
Subsection I – General Provision (Article 59) 67
Subsection II – Amendments to the Constitution (Article 60) 67
Subsection III – The Laws (Articles 61 to 69) 68
Section IX – Accounting, Financial and Budget Control (Articles 70 to 75) 72
Chapter II – The Executive Branch (Articles 76 to 91) 76
Section I – The President and the Vice-President of the Republic (Articles 76 to 83) 76
Section II – Duties of the President of the Republic (Article 84) 77
Section III – Liability of the President of the Republic (Articles 85 and 86) 79
Section IV – The Ministers of State (Articles 87 and 88) 80
Section V – The Council of the Republic and the National Defense Council (Articles 89 to 91) 81
Subsection I – The Council of the Republic (Articles 89 and 90) 81
Subsection II – The National Defense Council (Article 91) 81
Chapter III – The Judicial Branch (Articles 92 to 126) 82
Section I – General Provisions (Articles 92 to 100) 82
Section II – The Federal Supreme Court (Articles 101 to 103-B) 91
Section III – The Superior Court of Justice (Articles 104 and 105) 97
Section IV – The Federal Regional Courts and the Federal Judges (Articles 106 to 110) 99
Section V – Labor Courts and Judges (Articles 111 to 117) 101
Section VI – Electoral Courts and Judges (Articles 118 to 121) 104
Section VII – Military Courts and Judges (Articles 122 to 124) 105
Section VIII – Courts and Judges of the States (Articles 125 and 126) 106
Chapter IV – The Functions Essential to Justice (Articles 127 to 135) 107
Section I – The Prosecution Office (Articles 127 to 130-A) 107
Section II – The Public Advocacy (Articles 131 and 132) 112
Section III – The Legal Profession (Article 133) 113

Section IV – The Office of the Public Defender (Articles 134 and 135) 113

Title V – The Defense of the State and of the Democratic Institutions (Articles 136 to 144) 115
Chapter I – The State of Defense and the State of Siege (Articles 136 to 141) 115
Section I – The State of Defense (Article 136) 115
Section II – The State of Siege (Articles 137 to 139) 116
Section III – General Provisions (Articles 140 and 141) 117
Chapter II – The Armed Forces (Articles 142 and 143) 118
Chapter III – Public Security (Article 144) 120

Title VI – Taxation and Budget (Articles 145 to 169) 122
Chapter I – The National Tax System (Articles 145 to 162) 122
Section I – General Principles (Articles 145 to 149-A) 122
Section II – Limitations on the Taxing Power (Articles 150 to 152) 125
Section III – Federal Taxes (Articles 153 and 154) 127
Section IV – State and Federal District Taxes (Article 155) 128
Section V – Municipal Taxes (Article 156) 132
Section VI – Sharing of Tax Revenue (Articles 157 to 162) 133
Chapter II – Public Finances (Articles 163 to 169) 135
Section I – General Rules (Articles 163 and 164) 135
Section II – Budgets (Articles 165 to 169) 136

Title VII – The Economic and Financial Order (Articles 170 to 192) 145
Chapter I – The General Principles of the Economic Activity (Articles 170 to 181) 145
Chapter II – Urban Policy (Articles 182 and 183) 149
Chapter III – Agricultural and Land Policy and Agrarian Reform (Articles 184 to 191) 150
Chapter IV – The National Financial System (Article 192) 152

Title VIII – The Social Order (Articles 193 to 232) 154
Chapter I – General Provision (Article 193) 154
Chapter II – Social Welfare (Articles 194 to 204) 154

Section I – General Provisions (Articles 194 and 195) 154
Section II – Health (Articles 196 to 200) 157
Section III – Social Security (Articles 201 and 202) 159
Section IV – Social Assistance (Articles 203 and 204) 162
Chapter III – Education, Culture and Sports (Articles 205 to 217) 163
Section I – Education (Articles 205 to 214) 163
Section II – Culture (Articles 215 to 216-A) 167
Section III – Sports (Article 217) 170
Chapter IV – Science, Technology, and Innovation (Articles 218 to 219-B) 170
Chapter V – Social Communication (Articles 220 to 224) 172
Chapter VI – Environment (Article 225) 174
Chapter VII – Family, Children, Adolescents, Young People and Senior Citizens (Articles 226 to 230) 175
Chapter VIII – Indigenous People (Articles 231 and 232) 178

Title IX – General Constitutional Provisions (Articles 233 to 250) 180

Temporary Constitutional Provisions Act 188
Revision Constitutional Amendments 244
Constitutional Amendments 245

PREAMBLE

We, the representatives of the Brazilian people, convened in the National Constituent Assembly to institute a democratic State aimed at ensuring the exercise of social and individual rights, liberty, security, well-being, development, equality and justice as supreme values of a fraternal, pluralist and unprejudiced society, founded on social harmony and committed, in the domestic and international orders, to the peaceful settlement of disputes, promulgate, under the protection of God, the following CONSTITUTION OF THE FEDERATIVE REPUBLIC OF BRAZIL.

Editor's note: The alterations deriving from the Constitutional Amendments (CA) and from the Revision Constitutional Amendments (RCA) have already been incorporated into the main text. The modifying amendments are mentioned in parentheses at the end of each provision that they have altered.

TITLE I
Fundamental Principles

Article 1. The Federative Republic of Brazil, formed by the permanent union of the states, the municipalities, and the Federal District, is a democratic State ruled by the law and founded on:

I – sovereignty;

II – citizenship;

III – human dignity;

IV – social values of labor and free enterprise;

V – political pluralism.

Single paragraph. All power emanates from the people, who exercise it by means of elected representatives or directly, as provided under this Constitution.

Article 2. The powers of the Union are vested in the legislative, the executive, and the judicial branch, which are independent and harmonious with each other.

Article 3. The fundamental objectives of the Federative Republic of Brazil are:

I – to build a free, fair and solidary society;

II – to guarantee national development;

III – to eradicate poverty and marginalization and to reduce social and regional inequalities;

IV – to promote the well-being of all, without prejudice as to origin, race, sex, color, age and any other forms of discrimination.

Article 4. The Federative Republic of Brazil governs its international relations by the following principles:

I – national independence;

II – prevalence of human rights;

III – self-determination of the people;

IV – nonintervention;

V – equality among nations;

VI – defense of peace;

VII – peaceful settlement of conflicts;

VIII – repudiation of terrorism and racism;

IX – cooperation among people for the progress of humanity;

X – granting of political asylum.

Single paragraph. The Federative Republic of Brazil shall pursue the economic, political, social, and cultural integration of the people of Latin America, with a view to form a Latin-American community of nations.

TITLE II

Fundamental Rights and Guarantees

CHAPTER I

Individual and Collective Rights and Duties

Article 5. All people are equal before the law, without any distinction whatsoever. Brazilians and foreigners residing in the country are ensured the inviolability of their right to life, liberty, equality, security, and property, under the following terms:

I – men and women have equal rights and duties, as provided by this Constitution;

II – no one shall be compelled to do or refrain from doing something except by reason of law;

III – no one shall be subjected to torture or to inhuman or degrading treatment;

IV – expression of thought is free and anonymity is forbidden;

V – the right of reply equivalent to the grievance is ensured, in addition to compensation for pecuniary loss, emotional distress or damages to reputation;

VI – freedom of conscience and of belief is inviolable; the free exercise of religious services is ensured as well as, as provided by law, the protection of places of worship and their liturgies;

VII – religious assistance in civil and military establishments of collective confinement is guaranteed;

VIII – no person shall be deprived of rights due to religious belief or philosophical or political convictions, unless the person claims it to be exempted from legal obligations imposed on everyone and refuses to comply with an alternative provision established by law;

IX – the expression of intellectual, artistic, scientific, and communication activity is free, irrespective of censorship or license;

X – personal intimacy, private life, honor and reputation are inviolable; the right to compensation for pecuniary loss or emotional distress due to their breach is ensured;

XI – the home is the inviolable refuge of individuals; no one may enter therein without the resident's consent, except in the event of *flagrante delicto* or disaster, or to provide help, or, during the day, with a court order;

XII – the secrecy of correspondence and of telegraphic, data and telephone communications is inviolable; except, in the latter case, by a court order, in the cases and as provided by law for the purposes of criminal investigation or finding of evidence in criminal proceedings;

XIII – all people are free to perform any occupation, as long as they comply with the professional qualifications the law shall establish;

XIV – access to information of public interest is ensured to everyone; the confidentiality of the source shall be safeguarded whenever necessary for the professional practice;

XV – all people are free to move within the national territory during peacetime; they may enter it, remain therein or leave it with their possessions, as provided by law;

XVI – all people may hold peaceful meetings in public places regardless of authorization, as long as they bear no weapons, do not hinder another meeting previously convened for the same place, and issue prior notice to the competent authority;

XVII – freedom of association for lawful purposes is fully guaranteed; paramilitary groups are forbidden;

XVIII – the establishment of associations and, according to law, that of cooperatives, requires no authorization; the State is forbidden to intervene in their operation;

XIX – only by a court order may associations be compulsorily dissolved or have their activities suspended; in the former case, a final and unappealable decision is required;

XX – no one shall be compelled to join an association or to remain associated;

XXI – associations expressly authorized have standing to represent their affiliates in court or out of it;

XXII – the right of property is guaranteed;

XXIII – property shall comply with its social purpose;

XXIV – the law shall establish the expropriation procedure for public need or interest, or for social interest, by means of a fair and prior pecuniary compensation, except in the cases provided otherwise in this Constitution;

XXV – the competent authority may use private property in the event of imminent public danger; the owner is ensured subsequent compensation in case of damage;

XXVI – small rural property, as defined by law, whenever used by families, shall not be subject to attachment for payment of debts incurred by reason of its productive activity; the law shall establish the means to finance its development;

XXVII – authors own the exclusive rights to use, publish or reproduce their works; such rights are transmissible only to the heirs for as long as the law establishes;

XXVIII – under the terms of law, the following are ensured:

a) the protection of rights of individuals who participate in collective works and of the human image and voice reproduction, including in sports activities;

b) the right of authors, interpreters, and their respective unions and associations to monitor the economic exploitation of the works which they create or participate in;

XXIX – the law shall ensure the inventors of industrial inventions a temporary privilege for their use, in addition to protection of industrial creations, ownership of trademarks, company names and other distinctive signs, taking into account the interest of society and the technological and economic development of the country;

XXX – the right of inheritance is guaranteed;

XXXI – the inheritance of foreigners' assets situated in Brazil shall be determined by Brazilian law in favor of the Brazilian spouse or children, whenever the law applicable to the deceased does not benefit them the most;

XXXII – the State shall provide for consumer protection, as set forth by law;

XXXIII – all people are entitled to receive information concerning their private, collective or general interest from government bodies, which shall be provided within the period established by law, under penalty of liability, except for those information whose secrecy is essential to the security of society and of the State;

XXXIV – the following are guaranteed to everyone, without having to pay any fee:

a) to submit petitions to the government for the defense of rights, against illegal acts or abuse of power;

b) to obtain certificates issued by government offices for the defense of rights and to clarify situations of personal interest;

XXXV – the law shall not exclude any injury or threat to a right from review by the judiciary;

XXXVI – the law shall not obstruct vested rights, perfect legal acts or *res judicata*;

XXXVII – there shall be no trial or court *ad hoc*;

XXXVIII – the institution of the jury is recognized; the law shall provide its organization and ensure the:

a) full defense;

b) secret voting;

c) sovereignty of verdicts;

d) jurisdiction to try willful crimes against life;

XXXIX – there is no crime unless a prior law defines it, nor is there a punishment unless a prior law so provides;

XL – criminal law shall not retroact, except to benefit the defendant;

XLI – the law shall punish any discrimination that may attempt against fundamental rights and liberties;

XLII – the practice of racism is a non-bailable crime, with no statute of limitations and subject to prison sentence, under the terms of law;

XLIII – acts of torture, illicit trafficking in narcotics and similar drugs, terrorism, and crimes regarded as heinous shall not be bailable or subject to grace or amnesty by law; those who order, commit or omit themselves while being able to prevent such crimes shall be held liable;

XLIV – actions of civilian or military armed groups against the constitutional order and the democratic State are crimes neither bailable nor subject to the statute of limitations;

XLV – no punishment shall extend beyond the person convicted; according to law, the compensation for damage and the loss of assets may be ordered and enforced against the successors up to the amount of assets that were transferred to them;

XLVI – the law shall regulate individualization of punishment and shall provide for, *inter alia*, the following:

a) imprisonment or restraint of freedom;

b) loss of assets;

c) fine;

d) community service;

e) suspension or interdiction of rights;

XLVII – there shall be no punishment of:

a) death, except in case of declared war, under the terms of Article 84, item XIX;

b) perpetual nature;

c) forced labor;

d) banishment;

e) cruel nature;

XLVIII – the sentence shall be served in separate establishments, according to the nature of the offense, and age and sex of the convict;

XLIX – prisoners are ensured respect for their physical and moral integrity;

L – female prisoners shall be ensured conditions that allow them to remain with their children during the nursing period;

LI – no Brazilian shall be extradited, except naturalized Brazilians, if they committed a common crime prior to naturalization or if their participation in unlawful traffic in narcotics and similar drugs is proven, under the terms of law;

LII – no foreigner shall be extradited for a political or ideological offense;

LIII – no one shall be tried or sentenced other than by the competent authority;

LIV – no one shall be deprived of freedom or property without due process of law;

LV – parties in judicial or administrative proceedings and defendants in general are ensured an adversary system and a full defense, with the means and resources inherent therein;

LVI – evidence obtained through unlawful means is unacceptable in proceedings;

LVII – no one shall be considered guilty before the criminal conviction becomes final and unappealable;

LVIII – the holder of a civil identity document shall not be submitted to criminal identification, except for the cases provided by law;

LIX – private lawsuits for crimes subject to public prosecution shall be admitted, whenever the latter is not filed within the period established by law;

LX – the law may only restrict the disclosure of proceedings if the restriction is required to protect privacy or the interest of society;

LXI – no one shall be arrested unless in *flagrante delicto* or by a written and grounded order of a competent judicial authority, except for military transgression or specific military crime, as defined under the law;

LXII – the arrest of any person as well as the place where one is being held shall be immediately communicated to the competent judge and to the arrestee's family or to a person the arrestee designates;

LXIII – arrested people shall be informed of their rights, including the right to remain silent; they are ensured assistance by their family and by a lawyer;

LXIV – arrested people are entitled to identification of those responsible for their arrest or their police interrogation;

LXV – courts shall immediately release the people illegally arrested;

LXVI – no one shall be taken to prison or held therein when the law authorizes release prior to trial whether subject or not to bail;

LXVII – there shall be no civil imprisonment for debt, except for a person who is voluntarily and inexcusably in default on alimony obligation or for the unjustifiably unfaithful bailee;

LXVIII – *habeas corpus* shall be granted whenever a person suffers or is threatened with suffering violence or coercion in their freedom of movement as a result of illegal actions or abuse of power;

LXIX – a *writ of mandamus* shall be issued to protect a clear and certain right, when such right is not protected by *habeas corpus* or *habeas data*, whenever the party responsible for the illegal action or abuse of power is a public authority or an agent of a legal entity performing governmental duties;

LXX – a collective *writ of mandamus* may be filed by:

a) a political party represented in the National Congress;

b) a union, a professional association or an association legally established and in operation for at least one year, to defend the interests of its members or associates;

LXXI – a *writ of injunction* shall be granted whenever the lack of regulatory provisions hinders the exercise of constitutional rights and liberties in addition to the prerogatives inherent in nationality, sovereignty and citizenship;

LXXII – *habeas data* shall be granted to:

a) secure the information related to the petitioner contained in records or databases of governmental entities or of agencies of a public character;

b) rectify data whenever the petitioner does not prefer to do so through a confidential judicial or administrative proceeding;

LXXIII – any citizen has standing to file a popular action to annul an act injurious to the public property or to the property of an entity in which the State participates, to administrative morality, to the environment and to the historic and cultural heritage; except in the case of proven bad faith, the plaintiff is exempt from court costs and from the burden of paying the prevailing party's attorneys' fees and costs;

LXXIV – the State shall provide full and free of charge legal assistance to any person who proves insufficiency of funds;

LXXV – the State shall compensate anyone convicted by judicial error, as well as any person who remains imprisoned for a period longer than that established by the sentence;

LXXVI – the following are free of charge for any person who proves the condition of poverty, under the terms of law:

a) civil birth certificate;

b) death certificate;

LXXVII – *habeas corpus* and *habeas data* proceedings and, under the terms of law, the acts necessary to the exercise of citizenship, are free of charge;

LXXVIII – everyone is ensured a trial within a reasonable time and the remedies that guarantee the expeditious processing of judicial and administrative proceeding; (CA 45, 2004)

Paragraph 1. The provisions defining fundamental rights and guarantees apply immediately.

Paragraph 2. The rights and guarantees established in this Constitution do not exclude others deriving from the regime and principles adopted by it, or from the international treaties to which the Federative Republic of Brazil is a party.

Paragraph 3. International human rights treaties and conventions on human rights approved by both Houses of the National Congress, in two different voting sessions, by vote of three-fifths of their respective members, shall be equivalent to Constitutional Amendments. (CA 45, 2004)

Paragraph 4. Brazil submits itself to the jurisdiction of an International Criminal Court to whose creation it has expressed adhesion. (CA 45, 2004)

CHAPTER II
Social Rights

Article 6. Education, health, food, work, housing, transportation, leisure, security, social security, protection of motherhood and childhood, and assistance to the destitute, are social rights, as set forth by this Constitution. (CA 90, 2015)

Article 7. The following are rights of urban and rural workers, among others that aim to improve their social conditions:

I – employment protected against arbitrary dismissal or against dismissal without cause, under the terms of a supplementary law that shall establish severance pay, among other rights;

II – unemployment insurance, in the event of involuntary unemployment;

III – Guarantee Fund for the Length of Service;

IV – national uniform minimum wage, established by law, capable of meeting workers' basic living needs and those of their family for housing, nourishment, education, health, leisure, clothing, hygiene, transportation, and social security, with periodic adjustments to maintain its purchasing power; it is forbidden to use it as an index for any purpose;

V – salary floor in proportion to the laboriousness and complexity of the work;

VI – salary cut ban, except when established in a collective labor agreement or collective-bargaining agreement;

VII – guarantee that the salary will never fall below the minimum wage, for those receiving variable pay;

VIII – thirteenth salary based on full pay or the amount of the pension;

IX – pay rate for nightshift work higher than that for daytime work;

X – salary protection under the terms of law; the intentional withholding of salary shall be considered a crime;

XI – participation in the profits or results, independent of wages, and, exceptionally, participation in the management of the company, as provided by law;

XII – family allowance paid to each dependent of low-income workers, under the terms of law; (CA 20, 1998)

XIII – normal working hours not exceeding eight hours per day and forty-four hours per week, having the option to compensate working hours and reduce the length of workday through a collective labor agreement or collective-bargaining agreement;

XIV – workday of six hours for work carried out in continuous shifts, unless otherwise established under collective-bargaining agreement;

XV – paid weekly rest, preferably on Sundays;

XVI – overtime pay rate at least fifty percent higher than the regular pay rate;

XVII – annual vacation payed at least one third higher than the regular salary;

XVIII – maternity leave without loss of job and of salary, for a period of one hundred and twenty days;

XIX – paternity leave, under the terms established by law;

XX – protection of the labor market for women through specific incentives, under the terms of law;

XXI – advance notice of dismissal in proportion to the length of service of at least thirty days, under the terms of law;

XXII – reduction of employment related risks by means of health, hygiene and safety rules;

XXIII – additional remuneration for strenuous, unhealthy or dangerous work, as established by law;

XXIV – retirement pension;

XXV – free assistance for children and dependents of up to five years of age, in daycare centers and pre-school facilities; (CA 53, 2006)

XXVI – recognition of collective labor agreement or collective bargaining agreement;

XXVII – protection from effects of automation, as established by law;

XXVIII – occupational accident insurance coverage by employers, without excluding employers' liability to compensate for damage in case of fraud or fault;

XXIX – legal action, concerning credits from the employment relation, with a limitation of five years for urban and rural workers, up to the limit of two years after the end of the employment contract; (CA 28, 2000)

a) (Repealed) (CA 28, 2000)

b) (Repealed) (CA 28, 2000)

XXX – prohibition of any difference in wages, duties and employment decisions based on sex, age, color or marital status;

XXXI – prohibition of any discrimination with respect to wages and hiring criteria of handicapped workers;

XXXII – prohibition of any distinction between manual, technical, and intellectual work or among the respective professionals;

XXXIII – prohibition of night, dangerous, or unhealthy work for minors under eighteen years of age, and of any work for minors under sixteen years of age, except as an apprentice, for minors above fourteen years of age; (CA 20, 1998)

XXXIV – equal rights for workers with a permanent employment relationship and for temporary workers.

Single paragraph. Domestic workers are ensured the rights set forth in items IV, VI, VII, VIII, X, XIII, XV, XVI, XVII, XVIII, XIX, XXI, XXII, XXIV, XXVI, XXX, XXXI, and XXXIII, and, observing the conditions established by law and with due regard for simplified compliance with both primary and ancillary tax obligations arising from labor relations and from their peculiarities, also those rights set forth in items I, II, III, IX, XII, XXV, and XXVIII, as well as integration into social security. (CA 72, 2013)

Article 8. Professional or union association is free, with regard to the following:

I – the law may not require authorization of the State for a union to be founded, except for authorization for registration with the competent agency; the government is forbidden to interfere in the union;

II – it is forbidden to create more than one union, at any level, representing a professional or economic category, in the same territorial base, which shall be defined by the workers or employers concerned, which base may not cover less than the area of one municipality;

III – the Union is responsible for defending the collective or individual rights and interests of the category, including legal or administrative disputes;

IV – the general assembly shall establish the contribution which, in the case of a professional category, shall be discounted from the payroll, to support the confederative system of the respective union representation, regardless of the contribution set forth by law;

V – no one shall be required to join or to remain a member of a union;

VI – collective labor bargaining must be held with the participation of unions;

VII – retired members shall be entitled to vote and be voted in elections held by unions;

VIII – the dismissal of unionized employees is forbidden from the moment they register their candidacy to a position of union direction or representation and, if elected, even if as a substitute, up to one year after the end of the office term, unless committed a serious fault as established by law.

Single paragraph. The provisions of this Article apply to the organization of rural unions and those of fishing communities, with due regard for the conditions established by law.

Article 9. The right to strike is guaranteed; workers are empowered to decide on the advisability of exercising it and on the interests to be defended thereby.

Paragraph 1. The law shall define essential services or activities and shall provide for the satisfaction of the community's pressing needs.

Paragraph 2. The abuses committed shall subject those responsible to the penalties defined by law.

Article 10. The participation of workers and employers is ensured in the collegial bodies of government bodies in which their professional or social security interests are subject to discussion and deliberation.

Article 11. In companies with more than 200 employees, the workers are ensured the election of a representative for the exclusive purpose of furthering direct negotiations with the employers.

CHAPTER III

Nationality

Article 12. The following are Brazilians:

I – by birth:

a) those born in the Federative Republic of Brazil, even if to foreign parents, as long as they are not in the service of their country;

b) those born abroad, to a Brazilian father or a Brazilian mother, if either of them is in the service of the Federative Republic of Brazil;

c) those born abroad, to a Brazilian father or a Brazilian mother, as long as they are registered with a competent Brazilian authority, or come to reside in the Federative Republic of Brazil, and opt for the Brazilian nationality at any time after reaching age of majority; (CA 54, 2007)

II – by naturalization:

a) those who, as set forth by law, acquire Brazilian nationality; residing in the country for one uninterrupted year and having moral integrity are the only requirements for people from Portuguese-speaking countries;

b) foreigners of any nationality, with no criminal conviction, residing in the Federative Republic of Brazil for over fifteen uninterrupted years, if they so request. (RCA 3, 1994)

Paragraph 1. The rights inherent to Brazilians shall be granted to Portuguese citizens with permanent residence in Brazil, if there is reciprocity in favor of Brazilians, except for the cases provided otherwise under this Constitution. (RCA 3, 1994)

Paragraph 2. The law may not establish any distinction between natural born citizen and naturalized citizens, except for the cases provided under this Constitution.

Paragraph 3. The following positions are restricted to natural born citizens of Brazil:

I – president and vice-president of the Republic;

II – president of the Chamber of Deputies;

III – president of the Federal Senate;

IV – justice of the Federal Supreme Court;

V – in the diplomatic career;

VI – officers of the Armed Forces;

VII – Minister of Defense. (CA 23, 1999)

Paragraph 4. Loss of nationality shall be declared for a Brazilians who:

I – have their naturalization cancelled by a court decision on account of an activity harmful to the national interest;

II – acquire another nationality, except in the cases: (RCA 3, 1994)

a) of recognition of the original nationality by foreign law; (RCA 3, 1994)

b) of imposition of naturalization, under foreign rules, to a Brazilian resident in a foreign State, as a condition for remaining in its territory, or for the exercise of civil rights. (RCA 3, 1994)

Article 13. Portuguese is the official language of the Federative Republic of Brazil.

Paragraph 1. The national flag, anthem, coat of arms and seal are the symbols of the Federative Republic of Brazil.

Paragraph 2. The state, Federal District and municipalities may have symbols of their own.

CHAPTER IV
Political Rights

Article 14. The sovereignty of the people shall be exercised by universal suffrage and by direct and secret voting, with equal value for all and according to the law, by means of:

I – plebiscite;

II – *referendum*;

III – popular initiative.

Paragraph 1. Electoral enrollment and voting are:

I – mandatory for people over eighteen years of age;

II – optional for:

a) the illiterate;

b) those over seventy years of age;

c) those over sixteen and under eighteen years of age.

Paragraph 2. Foreigners, and conscripts during their period of compulsory military service, are forbidden to register as voters.

Paragraph 3. The conditions for eligibility, according to law, are:

I – Brazilian nationality;

II – full exercise of the political rights;

III – electoral enrollment;

IV – electoral domicile in the electoral district;

V – membership in a political party;

VI – minimum age of:

a) thirty-five years for president and vice-president of the Republic and senator;

b) thirty years for governor and vice-governor of a state and of the Federal District;

c) twenty-one years for federal deputy, state or district deputy, mayor, vice-mayor, and justice of the peace;

d) eighteen years for municipal councilor.

Paragraph 4. The illiterate and those who cannot be registered as voters are not eligible.

Paragraph 5. The president of the Republic, state and Federal District governors, mayors and those who have succeeded or replaced them during their terms of office may be reelected for only one subsequent term. (CA 16, 1997)

Paragraph 6. In order to run for other offices, the president of the Republic, state and Federal District governors and mayors must resign from their respective offices at least six months in advance of the election.

Paragraph 7. The spouse and relatives by blood or marriage, up to the second degree or by adoption, of the president of the Republic, governor of a state or Territory or Federal District, mayor or of those who have replaced them within the six months preceding the election, are not eligible in the jurisdiction of the incumbent, unless they already hold an elective office and are candidates for reelection.

Paragraph 8. Members of the Armed Forces who can be registered as voters are eligible if the following conditions are met:

I – if they have served for less than ten years, they shall be on leave from military activities;

II – if they have served for more than ten years, they shall be discharged from military duties by the superior and, if elected, shall be automatically retired upon issuance of the official certificate of electoral victory.

Paragraph 9. Supplementary law shall establish other cases of ineligibility and the periods for such ineligibilities to cease in order to protect administrative probity, morality for the exercise of the office, considering the past life of the candidate, and normality and legitimacy of elections from the influence of the economic power or abuse while holding an office, position or job in the government bodies or associated entities. (RCA 4, 1994)

Paragraph 10. The exercise of an elective office may be contested before the Electoral Courts within a period of fifteen days after the date of issuance of the official certificate of electoral victory, substantiating the suit with evidence of abuse of economic power, corruption or fraud.

Paragraph 11. The suit procedure contesting the office shall be secret; the plaintiff shall be held liable under the law in case of presenting a reckless suit or acting in bad faith.

Article 15. Disfranchisement of political rights is forbidden; the loss or suspension of such rights shall apply only in the event of:

I – cancellation of naturalization by a final and unappealable decision;

II – absolute civil incapacity;

III – final and unappealable criminal sentence, for as long as its effects last;

IV – refusal to comply with an obligation imposed upon everyone or to render alternative service, according to Article 5, item VIII;

V – administrative improbity, according to Article 37, paragraph 4.

Article 16. The law that alters the electoral procedure shall come into force on the date of its publication and shall not apply to the elections that take place within one year of it being in force. (CA 4, 1993)

CHAPTER V
Political Parties

Article 17. The creation, fusion, merger and extinction of political parties is free, with due regard for national sovereignty, the democratic regime, the plurality of political parties, the fundamental rights of the individual, and observing the following precepts:

I – nationwide reach;

II – prohibition against receiving financial support either from the government or from a foreign entity, and prohibition against being subject to them;

III – rendering of accounts to the Electoral Courts;

IV – operation in the National Congress in accordance with the law.

Paragraph 1. Political parties are ensured autonomy to define their internal structure and establish rules on the choice, formation and period of their permanent and temporary bodies and on their organization and operation. They are also ensured autonomy to adopt criteria for choosing coalitions and its regime in majority elections, it being forbidden to celebrate them in the proportional elections, without requiring linkage among candidacies in the national, state, district or municipal level. Party by-laws shall establish rules for party discipline and loyalty. (CA 97, 2017)

Paragraph 2. After acquiring corporate legal status under civil law, political parties shall register their bylaws at the Superior Electoral Court.

Paragraph 3. Political party funds and free of charge access to radio and television shall only be awarded, according to law, to political parties that alternatively: (CA 97, 2017)

I – obtain, in the elections to the Chamber of Deputies, a minimum of 3% (three percent) of the valid votes, distributed in at least one third of the federal entities, with a minimum of 2% of the valid votes in each of them; or (CA 97, 2017)

II – have elected at least fifteen federal deputies distributed in at least one third of the federal entities. (CA 97, 2017)

Paragraph 4. Political parties are forbidden to use paramilitary organizations.

Paragraph 5. The elected party candidate who do not fulfill the requirements set forth under paragraph 3 of this Article has the term guaranteed and may choose to affiliate, without losing office, to another party that has reached them, and this affiliation shall not be considered for the distribution purposes of party funds or free of charge access to radio and television. (CA 97, 2017)

TITLE III

The Organization of the State

CHAPTER I

The Political and Administrative Organization

Article 18. The political and administrative organization of the Federative Republic of Brazil comprises the Union, states, Federal District, and municipalities, all of them autonomous, under the terms of this Constitution.

Paragraph 1. Brasilia is the federal capital.

Paragraph 2. The Federal Territories are part of the Union and their establishment, transformation into states or reintegration into the state of origin shall be regulated by a supplementary law.

Paragraph 3. The states may merge, subdivide or dismember to be annexed to others or to form new states or Federal Territories, subject to the approval of the population directly concerned, by means of a plebiscite, and of the National Congress, by means of a supplementary law.

Paragraph 4. The establishment, merger, fusion and dismemberment of municipalities shall be implemented through a state law, within the period provided by a supplementary federal law, and shall depend on prior consultation of the municipalities population concerned by means of a plebiscite, after a municipal feasibility study is released and published as set forth by law. (CA 15, 1996)

Article 19. The Union, states, Federal District, and municipalities are forbidden to:

I – establish religious sects or churches, subsidize them, hinder their activities, or maintain relationships of dependence or alliance with them or their representatives, without prejudice to collaboration in the public interest in the manner set forth by law;

II – refuse to honor public documents;

III – create distinctions between Brazilians or preferences favoring some.

CHAPTER II
The Union

Article 20. The Union owns the following properties:

I – the property that currently belongs to it as well as that which may be assigned to it;

II – the unoccupied lands essential to the defense of the boundaries, the fortifications and military constructions, the federal routes of communication and the preservation of the environment, as defined by law;

III – the lakes, rivers and any watercourses in lands within its domain, or that flow through more than one State, that serve as boundaries with other countries, or that extend into foreign territory or proceed therefrom, as well as riverbanks and river beaches;

IV – the river and lake islands in zones bordering with other countries; sea beaches; ocean and offshore islands, excluding those which are the seat of Municipalities, with the exception of areas assigned to public services and to federal environmental units, and those referred to in Article 26, item II; (CA 46, 2005)

V – the natural resources of the continental shelf and of the exclusive economic zone;

VI – the territorial sea;

VII – tidal lands and those added to them;

VIII – hydraulic energy potentials;

IX – mineral resources, including those of the subsoil;

X – natural underground caves and the archaeological and pre-historic sites;

XI – those lands traditionally occupied by Indigenous.

Paragraph 1. In accordance with the law, the participation in the results of the exploitation of petroleum or natural gas, hydric resources for the purpose of generation of electric power and other mineral resources in the respective territory, continental shelf, territorial sea or exclusive economic zone, or financial compensation for the exploitation thereof, is assured to the Union, states, Federal District and municipalities. (CA 102, 2019)

Paragraph 2. The strip of land up to a hundred and fifty kilometers in width alongside the terrestrial boundaries, designated as boundary zone, is considered essential to the defense of the national territory and its occupation and utilization shall be regulated by law.

Article 21. The Union shall have the power to:

I – maintain relations with foreign States and participate in international organizations;

II – declare war and make peace;

III – ensure national defense;

IV – allow foreign forces, in the cases provided for in a supplementary law, to pass through the national territory or to remain therein temporarily;

V – declare a state of siege, a state of defense and a federal intervention;

VI – authorize and control the production and trade of military material;

VII – issue currency;

VIII – manage the foreign exchange reserves of the country and control financial operations, especially those of credit, exchange and capitalization, as well as insurance and private security;

IX – prepare and implement the national and regional planning for organizing the territory and for economic and social progress;

X – maintain the postal service and the national air mail;

XI – operate, directly or through authorization, concession or permission, the telecommunications services, as set forth by law, which shall provide for the organization of the services, the establishment of a regulatory agency and other institutional issues; (CA 8, 1995)

XII – operate, directly or through authorization, concession or permission:

a) the services of sound broadcasting and of sound and image broadcasting; (CA 8, 1995)

b) the electric power services and facilities and the exploitation to generate energy of watercourses, jointly with the states wherein those hydro-energetic potentials are located;

c) air and aerospace navigation and airport infrastructure;

d) railway and waterway services between seaports and national borders or which cross the boundary of a state or Territory;

e) interstate and international highway passenger transportation services;

f) sea, river and lake ports;

XIII – organize and maintain the judicial branch, the Prosecution Office of the Federal District and of the Territories, and the Office of the Public Defender of the Territories; (CA 69, 2012)

XIV – organize and maintain the civil police, the correctional police, the military police, and the military fire brigade of the Federal District, as well as provide financial support to the Federal District to perform public services using a specific fund; (CA 104, 2019)

XV – organize and maintain the official services of statistics, geography, geology and cartography of national scope;

XVI – classify, for guidance purposes, public entertainment and radio and television programs;

XVII – grant amnesty;

XVIII – plan and promote permanent defense against public disasters, especially droughts and floods;

XIX – establish a national system for the management of hydro resources and define criteria for the concession of the right to their use;

XX – establish directives for urban development, including housing, basic sanitation and urban transportation;

XXI – establish principles and directives for the national transportation system;

XXII – perform the services of maritime, airport, and border police; (CA 19, 1998)

XXIII – operate nuclear energy services and facilities of any nature and exercise State monopoly over research, mining, enrichment and reprocessing, industrialization and trade in nuclear ores and their by-products, taking into account the following principles and conditions:

a) all nuclear activity within the national territory shall only be admitted for peaceful purposes and subject to approval by the National Congress;

b) under a permission, authorization is granted for the sale and use of radioisotopes in research and for medical, agricultural, and industrial purposes; (CA 49, 2006)

c) under a permission, authorization is granted for the production, sale, and use of radioisotopes with a half-life lower than two hours; (CA 49, 2006)

d) civil liability for nuclear damage does not depend on the existence of fault; (CA 49, 2006)

XXIV – organize, maintain and carry out inspection of working conditions;

XXV – establish the areas and conditions for the exercise of placer mining activities in associative form.

Article 22. The Union has the exclusive power to legislate on:

I – civil, commercial, criminal, procedural, electoral, agrarian, maritime, aeronautical, space and labor law;

II – expropriation;

III – civil and military requisitioning, in case of imminent danger or in times of war;

IV – waters, energy, information technology, telecommunications and radio broadcasting;

V – the postal service;

VI – the monetary and measures systems, metal certificates and guarantees;

VII – policies for credit, foreign exchange, insurance and transfer of values;

VIII – foreign and interstate trade;

IX – guidelines for the national transportation policy;

X – the regime of the ports and lake, river, ocean, air and aerospace navigation;

XI – traffic and transportation;

XII – beds of ore, mines, other mineral resources and metallurgy;

XIII – nationality, citizenship and naturalization;

XIV – Indigenous;

XV – emigration, immigration, entry, extradition and expulsion of foreigners;

XVI – the organization of the national employment system and conditions for the practice of professions;

XVII – the judicial organization, the organization of the Prosecution Office of the Federal District and of the Territories and of the Office of the Public Defender of the Territories, as well as their administrative organization; (CA 69, 2012)

XVIII – the national statistical, cartographic and geological systems;

XIX – systems of savings, as well as of obtaining and guaranteeing popular savings;

XX – consortium and lottery systems;

XXI – general organization rules, troops, ordnance, guarantees, drafting, mobilization, retirement and pensions of the military polices and military fire brigades; (CA 103, 2019)

XXII – the jurisdiction of the federal police and of the federal highway and military polices;

XXIII – social security;

XXIV – directives and bases of the national education;

XXV – public records;

XXVI – nuclear activities of any nature;

XXVII – general rules for all types of bidding and contracting for government bodies, agencies and foundations of the Union, states, Federal District, and municipalities, in accordance with Article 37, item XXI, and for state-owned and mixed-capital companies, under the terms of Article 173, paragraph 1, item III; (CA 19,1998)

XXVIII – territorial defense, aerospace defense, maritime defense, civil defense, and national mobilization;

XXIX – commercial advertising.

Single paragraph. A supplementary law may authorize the states to legislate upon specific topics related to the matters listed in this Article.

Article 23. The Union, states, Federal District and municipalities have the collective power:

I – to ensure that the Constitution, the laws and the democratic institutions are respected, and that public property is preserved;

II – to provide for health and public assistance, for the protection and safeguard of handicapped people;

III – to protect the documents, works and other assets of historical, artistic or cultural value, monuments, remarkable landscapes and the archaeological sites;

IV – to prevent works of art and other assets of historical, artistic and cultural value from being taken out of the country, destroyed or from being stripped of their original characteristics;

V – to provide the means of access to culture, education, science, technology, research, and innovation; (CA 85, 2015)

VI – to protect the environment and to fight pollution in any of its forms;

VII – to preserve the forests, fauna and flora;

VIII – to promote agriculture and organize the supply of foodstuff;

IX – to promote housing construction programs and the improvement of housing and basic sanitation conditions;

X – to fight the causes of poverty and the factors leading to substandard living conditions, promoting the social integration of the underprivileged sectors of the population;

XI – to register, monitor and control the concessions of rights to research and exploit hydro and mineral resources within their territories;

XII – to establish and to implement an educational policy for traffic safety.

Single paragraph. Supplementary laws shall establish rules for the cooperation between the Union and the states, Federal District, and municipalities, aiming at the attainment of a balanced development and well-being on a nationwide scope. (CA 53, 2006)

Article 24. The Union, states and Federal District have the power to legislate concurrently on:

I – tax, financial, penitentiary, economic and urbanistic law;

II – budget;

III – trade boards;

IV – costs of forensic services;

V – production and consumption;

VI – forests, hunting, fishing, fauna, preservation of nature, defense of land and natural resources, protection of the environment and control of pollution;

VII – protection of historic, cultural and artistic heritage, as well as of assets of touristic interest and landscapes of outstanding beauty;

VIII – liability for damages to the environment, to consumers, to assets and rights of artistic, aesthetic, historical, and touristic value, as well as to remarkable landscapes;

IX – education, culture, teaching, sports, science, technology, research, development, and innovation; (CA 85, 2015)

X – establishment, operation and procedures of small claims courts;

XI – judicial procedures;

XII – social security, protection and defense of health;

XIII – legal assistance and public defense;

XIV – protection and social integration of handicapped people;

XV – protection of childhood and youth;

XVI – organization, guarantees, rights and duties of the civil polices.

Paragraph 1. Within the scope of concurrent legislation, the competence of the Union shall be limited to establish general rules.

Paragraph 2. The competence of the Union to legislate upon general rules does not exclude the supplementary competence of the states.

Paragraph 3. If there is no federal law on general rules, the states shall exercise full legislative competence to provide for their peculiarities.

Paragraph 4. The subsequent enactment of a federal law over general rules suspends the effectiveness of a state law to the extent that the two are contrary.

CHAPTER III

The Federated States

Article 25. The states are organized and governed by the Constitutions and laws they may adopt, in accordance with the principles of this Constitution.

Paragraph 1. All powers that this Constitution does not prohibit the states from exercising shall be conferred upon them.

Paragraph 2. The states shall have the power to operate, directly or by means of concession, the local services of piped gas, as provided by law; governors are forbidden to issue any provisional decree for its regulation. (CA 5, 1995)

Paragraph 3. The states may, by means of a supplementary law, establish metropolitan regions, urban agglomerations and micro regions, formed by the grouping of adjacent municipalities, in order to integrate the organization, the planning and the operation of public functions of common interest.

Article 26. The property of the states includes:

I – surface or subterranean waters, flowing, emerging or in deposit, with the exception, in this case, of those resulting from work carried out by the Union, as provided by law;

II – the areas, on ocean and coastal islands, which are within their domain, excluding those under the domain of the Union, the municipalities or third parties;

III – the river and lake islands which do not belong to the Union;

IV – the unoccupied lands not included among those belonging to the Union.

Article 27. The number of deputies in the State Legislature shall correspond to three times the representation of the state in the Chamber of Deputies and, when the number of thirty-six has been reached, it shall be increased by as many members as the number of federal deputies exceeding twelve.

Paragraph 1. The state deputies shall have a four-year term of office and the provisions of this Constitution related to electoral system, inviolability, immunity, remuneration, loss of office, leave of absence, impediments, and incorporation into the Armed Forces shall apply to them.

Paragraph 2. The compensation of state deputies shall be established by an act of the State Legislature, in the proportion of seventy-five percent, at most, of the compensation established, in specie, for federal deputies, as provided by Articles 39, paragraph 4; 57, paragraph 7; 150, item II; 153, item III; and 153, paragraph 2, item I. (CA 19, 1998)

Paragraph 3. State Legislatures shall have the power to provide upon their internal regulations, police, and administrative services of their secretariat and to fill in the respective offices.

Paragraph 4. The law shall provide for popular initiative proposals in the State Legislature process.

Article 28. The election of the governor and vice-governor of a state, for a term of office of four years , shall be held on the first Sunday of October, in the first round, and on the last Sunday of October, in the second round, as the case may be, of the year preceding the one in which the term of office of their predecessors ends, and they shall take office on January 1 of the following year, in accordance, otherwise, with the provisions of Article 77. (CA 16, 1997)

Paragraph 1. The governor who takes another post or function in the government bodies or associated entities shall lose office, with the exception of the taking of office due to a competitive civil-servant examination, and with due regard for the provisions in Article 38, items I, IV, and V. (Numbered paragraph 1 by CA 19, 1998)

Paragraph 2. The compensation of the governor, vice-governor, and state cabinet members shall be established by an act of the State Legislature, as provided by Articles 37, item XI, 39, paragraph 4, 150, item II, 153, item III, and 153, paragraph 2, item I. (CA 19, 1998)

CHAPTER IV

The Municipalities

Article 29. Municipalities shall be governed by an organic law, voted in two rounds, with a minimum interval of ten days between the rounds, and approved by two thirds of the members of the municipal council, which shall promulgate it, observing the principles established in this Constitution, in the Constitution of the respective state and the following precepts:

I – election of the mayor, vice-mayor and municipal councilors for a term of office of four years, by means of direct election held simultaneously throughout the country;

II – election of the mayor and vice-mayor on the first Sunday of October of the year preceding the end of the term of office of those they are to succeed, subject, in the case of municipalities with over two hundred thousand voters, to the provisions set forth in Article 77; (CA 16, 1997)

III – swearing in of the mayor and vice-mayor on January 1 of the year subsequent to the year of the election;

IV – the following limits shall apply to the composition of municipal councils: (CA 58, 2009)

a) 9 (nine) municipal councilors, in municipalities with up to 15,000 (fifteen thousand) inhabitants; (CA 58, 2009)

b) 11 (eleven) municipal councilors, in municipalities with over 15,000 (fifteen thousand) inhabitants and with up to 30,000 (thirty thousand) inhabitants; (CA 58, 2009)

c) 13 (thirteen) municipal councilors, in municipalities with over 30,000 (thirty thousand) inhabitants and with up to 50,000 (fifty thousand) inhabitants; (CA 58, 2009)

d) 15 (fifteen) municipal councilors, in municipalities with over 50,000 (fifty thousand) inhabitants and with up to 80,000 (eighty thousand) inhabitants; (CA 58, 2009)

e) 17 (seventeen) municipal councilors, in municipalities with over 80,000 (eighty thousand) inhabitants and with up to 120,000 (one hundred and twenty thousand) inhabitants; (CA 58, 2009)

f) 19 (nineteen) municipal councilors, in municipalities with over 120,000 (one hundred and twenty thousand) inhabitants and with up to 160,000 (one hundred and sixty thousand) inhabitants; (CA 58, 2009)

g) 21 (twenty-one) Municipal councilors, in Municipalities with over 160,000 (one hundred and sixty thousand) inhabitants and with up to 300,000 (three hundred thousand) inhabitants; (CA 58, 2009)

h) 23 (twenty-three) municipal councilors, in municipalities with over 300,000 (three hundred thousand) inhabitants and with up to 450,000 (four hundred and fifty thousand) inhabitants; (CA 58, 2009)

i) 25 (twenty-five) municipal councilors, in municipalities with over 450,000 (four hundred and fifty thousand) inhabitants and with up to 600,000 (six hundred thousand) inhabitants; (CA 58, 2009)

j) 27 (twenty-seven) municipal councilors, in municipalities with over 600,000 (six hundred thousand) inhabitants and with up to 750,000 (seven hundred and fifty thousand) inhabitants; (CA 58, 2009)

k) 29 (twenty-nine) municipal councilors, in municipalities with over 750,000 (seven hundred and fifty thousand) inhabitants and with up to 900,000 (nine hundred thousand) inhabitants; (CA 58, 2009)

l) 31 (thirty-one) municipal councilors, in municipalities with over 900,000 (nine hundred thousand) inhabitants and with up to 1,050,000 (one million and fifty thousand) inhabitants; (CA 58, 2009)

m) 33 (thirty-three) municipal councilors, in municipalities with over 1,050,000 (one million and fifty thousand) inhabitants and with up to 1,200,000 (one million and two hundred thousand) inhabitants; (CA 58, 2009)

n) 35 (thirty-five) municipal councilors, in municipalities with over 1,200,000 (one million and two hundred thousand) inhabitants and with up to 1,350,000 (one million three hundred and fifty thousand) inhabitants; (CA 58, 2009)

o) 37 (thirty-seven) municipal councilors, in municipalities with 1,350,000 (one million three hundred and fifty thousand) inhabitants and with up to 1,500,000 (one million five hundred thousand) inhabitants; (CA 58, 2009)

p) 39 (thirty-nine) municipal councilors, in municipalities with over 1,500,000 (one million five hundred thousand) inhabitants and with up to 1,800,000 (one million eight hundred thousand) inhabitants; (CA 58, 2009)

q) 41 (forty-one) municipal councilors, in municipalities with over 1,800,000 (one million eight hundred thousand) inhabitants and with up to 2,400,000 (two million four hundred thousand) inhabitants; (CA 58, 2009)

r) 43 (forty-three) municipal councilors, in municipalities with over 2,400,000 (two million four hundred thousand) inhabitants and with up to 3,000,000 (three million) inhabitants; (CA 58, 2009)

s) 45 (forty-five) municipal councilors, in municipalities with over 3,000,000 (three million) inhabitants and with up to 4,000,000 (four million) inhabitants; (CA 58, 2009)

t) 47 (forty-seven) municipal councilors, in municipalities with over 4,000,000 (four million) inhabitants and with up to 5,000,000 (five million) inhabitants; (CA 58, 2009)

u) 49 (forty-nine) municipal councilors, in municipalities with over 5,000,000 (five million) inhabitants and with up to 6,000,000 (six million) inhabitants; (CA 58, 2009)

v) 51 (fifty-one) municipal councilors, in municipalities with over 6,000,000 (six million) inhabitants and with up to 7,000,000 (seven million) inhabitants; (CA 58, 2009)

w) 53 (fifty-three) municipal councilors, in municipalities with over 7,000,000 (seven million) inhabitants and with up to 8,000,000 (eight million) inhabitants; and (CA 58, 2009)

x) 55 (fifty-five) municipal councilors, in municipalities with over 8,000,000 (eight million) inhabitants; (CA 58, 2009)

V – compensation of the mayor, vice-mayor, and local cabinet members established by an act of the municipal council, as provided by Articles 37, item XI, 39, paragraph 4, 150, item II, 153, item III, and 153, paragraph 2, item I; (CA 19, 1998)

VI – the compensation of municipal councilors shall be stipulated by their respective municipal councils in each legislative term for the subsequent one, with due regard for the provisions of this Constitution, in accordance with the criteria set forth in the respective Organic Law and the following maximum limits: (CA 25, 2000)

a) in municipalities having up to ten thousand inhabitants, the compensation of municipal councilors shall correspond, at the most, to twenty percent of the compensation of state deputies; (CA 25, 2000)

b) in municipalities having between ten thousand and one inhabitants and fifty thousand inhabitants, the compensation of municipal councilors shall correspond, at the most, to thirty percent of the compensation of state deputies; (CA 25, 2000)

c) in municipalities having between fifty thousand and one inhabitants and one hundred thousand inhabitants, the compensation of municipal councilors shall correspond, at the most, to forty percent of the compensation of state deputies; (CA 25, 2000)

d) in municipalities having between one hundred thousand and one inhabitants and three hundred thousand inhabitants, the compensation of municipal councilors shall correspond, at the most, to fifty percent of the compensation of state deputies; (CA 25, 2000)

e) in municipalities having between three hundred thousand and one inhabitants and five hundred thousand inhabitants, the compensation of Municipal councilors shall correspond, at the most, to sixty percent of the compensation of state deputies; (CA 25, 2000)

f) in municipalities having over five hundred thousand inhabitants, the compensation of municipal councilors shall correspond, at the most, to seventy-five percent of the compensation of state deputies; (CA 25, 2000)

VII – the total expenditure with the remuneration of the municipal councilors may not exceed the amount of five percent of the revenue of the municipality; (CA 1, 1992)

VIII – inviolability of the municipal councilors on account of their opinions, words and votes while in office and within the jurisdiction of the municipality; (Numbered from item VI by the CA 1, 1992)

IX – prohibitions and incompatibilities, while in the exercise of office of municipal councilor, similar, where applicable, to the provisions of this Constitution for the members of the National Congress and of the Constitution of the respective state for the members of the State Legislature; (Numbered from item VII by the CA 1, 1992)

X – trial of the mayor before the Court of Appeals; (Numbered from item VIII by the CA 1, 1992)

XI – organization of the legislative and supervisory functions of the municipal council; (Numbered from item IX by the CA 1, 1992)

XII – cooperation of the representative associations in municipal planning; (Numbered from item X by the CA 1, 1992)

XIII – popular initiative in the presentation of bills of specific interest to the municipality, the city or the neighborhoods, by means of the manifestation of at least five percent of the electorate; (Numbered from item XI by the CA 1, 1992)

XIV – loss of office of the mayor, as provided in Article 28, single paragraph. (Numbered from subitem XII by the CA 1, 1992)

Article 29-A. The total expenditures of the municipal legislative branch, including the compensation of municipal councilors and excluding expenses on retired personnel, may not exceed the following percentages, related to the total amount, effectively realized in

the prior year, of tax revenues and the transfers set forth in paragraph 5 of Article 153, and in Articles 158 and 159: (CA 25, 2000)

I – 7% (seven percent) in the case of municipalities having up to 100,000 (one hundred thousand) inhabitants; (CA 58, 2009)

II – 6% (six percent) in the case of municipalities having between 100,000 (one hundred thousand) and 300,000 (three hundred thousand) inhabitants; (CA 58, 2009)

III – 5% (five percent) in the case of municipalities having between 300,001 (three hundred thousand and one) inhabitants and 500,000 (five hundred thousand) inhabitants; (CA 58, 2009)

IV – 4.5% (four and five tenths percent) in the case of municipalities having between 500,001 (five hundred thousand and one) and 3,000,000 (three million) inhabitants; (CA 58, 2009)

V – 4% (four percent) in the case of municipalities having between 3,000,001 (three million and one) and 8,000,000 (eight million) inhabitants; (CA 58, 2009)

VI – 3.5% (three and five tenths percent) in the case of municipalities having over 8,000,001 (eight million and one) inhabitants. (CA 58, 2009)

Paragraph 1. The municipal council shall not spend more than seventy percent of its allocation on payroll, including expenses on the compensation of its member municipal councilors. (CA 25, 2000)

Paragraph 2. The following acts of the mayor are crimes of responsibility: (CA 25, 2000)

I – to make a remittance payment in excess of the limits stipulated in this Article; (CA 25, 2000)

II – not make a remittance payment before the twentieth day of each month; (CA 25, 2000)

III – to make a remittance below the proportion stipulated in the Budget Law. (CA 25, 2000)

Paragraph 3. It shall be a crime of responsibility for the president of the municipal council to disobey paragraph 1 of this Article. (CA 25, 2000)

Article 30. The municipalities have the power to:

I – legislate upon matters of local interest;

II – supplement federal and state legislations where pertinent;

III – enact and levy the taxation within their jurisdiction, as well as apply their revenues, without prejudice to the obligation of rendering accounts and publishing balance sheets within the periods established by law;

IV – create, organize and suppress districts, with due regard for the state legislation;

V – organize and render, directly or by concession or permission, the public services of local interest, including mass-transportation, which is of essential nature;

VI – maintain, with the technical and financial cooperation of the Union and the state, programs of early childhood and elementary school education; (CA 53, 2006)

VII – provide, with the technical and financial cooperation of the Union and the state, health services to the population;

VIII – promote, wherever pertinent, adequate territorial organization, by means of planning and control of use, apportionment and occupation of the urban land;

IX – promote the protection of the local historic and cultural heritage, with due regard for federal and state legislation and supervision.

Article 31. Supervision of the municipality shall be exercised by the municipal legislative branch through external control and by the internal control systems of the municipal executive branch, as provided by law.

Paragraph 1. External control of the municipal council shall be exercised with the assistance of the State or Municipal Accounting Court, or of the Municipal Accounting Councils or Courts, where they exist.

Paragraph 2. The preliminary report, issued by the competent agency, on the accounts the mayor must render annually, shall only be dismissed by a two-third decision of the members of the municipal council.

Paragraph 3. Accounts of the municipalities shall remain available each year to any taxpayer for sixty days for examination and evaluation, and any taxpayer may question their legitimacy, as provided by law.

Paragraph 4. Creation of Municipal Accounting Courts, Accounting Councils or agencies is forbidden.

CHAPTER V
The Federal District and the Territories

SECTION I
The Federal District

Article 32. The Federal District, which may not be divided into municipalities, shall be governed by an organic law, voted in two rounds, with a minimum interval of ten days, and approved by two-thirds of the Legislative Chamber, which shall enact it, in accordance with the principles set forth in this Constitution.

Paragraph 1. The Federal District shall have the legislative powers reserved to the states and municipalities.

Paragraph 2. Election of the governor and vice-governor, complying with the rules of Article 77, and of the district deputies shall coincide with that of the state governors and deputies, for terms of office of the same duration.

Paragraph 3. The provisions of Article 27 apply to the district deputies and the Legislative Chamber.

Paragraph 4. A federal law shall provide for the use of the civil police, the correctional police, the military police, and the military fire brigade by the government of the Federal District. (CA 104, 2019)

SECTION II

The Territories

Article 33. The law shall provide for the administrative and judicial organization of the Territories.

Paragraph 1. The Territories may be divided into municipalities, to which the provisions of Chapter IV of this Title shall apply, insofar as pertinent.

Paragraph 2. The accounts of a territorial government shall be submitted to the National Congress, with the prior opinion of the Federal Accounting Court.

Paragraph 3. Federal Territories with over one hundred thousand inhabitants shall have, in addition to a governor appointed according to this Constitution, courts of first and second instances, members of the Prosecution Office and Federal Public Defenders; the law shall provide for the elections to the Territory Chamber and its decision-making powers.

CHAPTER VI

Intervention

Article 34. The Union shall not intervene in the states or in the Federal District, except to:

I – maintain national integrity;

II – repel foreign invasion or that of one federal entity into another;

III – put an end to serious threat to public order;

IV – guarantee the free exercise of any of the government branches in the federal entities;

V – reorganize the finances of a federal entity that:

a) suspends the payment of its funded debt for more than two consecutive years, except for reasons of *force majeure*;

b) fails to deliver to the municipalities the tax revenues established in this Constitution within the periods of time set forth by law;

VI – enforce a federal law, judicial order or decision;

VII – ensure compliance with the following constitutional principles:

a) republican form, representative system and democratic regime;

b) rights of the human person;

c) municipal autonomy;

d) rendering of accounts of government bodies and associated entities;

e) application of the required minimum amount of the revenues from state taxes, including those stemming from transfers, for maintenance and development of education and for public health activities and services. (CA 29, 2000)

Article 35. The state shall not intervene in its municipalities, neither the Union in the municipalities located in a Federal Territory, except when:

I – a funded debt is not paid for two consecutive years, without reasons of *force majeure*;

II – required accounts are not rendered in the manner prescribed by law;

III – the required minimum amount of the municipal revenues has not been applied to maintain and develop education and public health activities and services; (CA 29, 2000)

IV – the Court of Appeals grants the request in the representation suit to ensure compliance with principles established in the State Constitution or to provide for law enforcement, court order or decision.

Article 36. A decree of intervention shall depend:

I – in the case of Article 34, item IV, on a request from the coerced or impeded legislative or executive branch, or on a requirement from the Federal Supreme Court, if the coercion is exercised against the judicial branch;

II – in the case of disobedience of a court order or decision, on a requirement from the Federal Supreme Court, the Superior Court of Justice or the Superior Electoral Court;

III – on the Federal Supreme Court decision granting the request in the representation suit filed by the federal attorney general, in the case of Article 34, item VII, and in the case of refusal to enforce a federal law. (CA 45, 2004)

IV – (Repealed) (CA 45, 2004)

Paragraph 1. The decree of intervention, which shall specify the extent, period and conditions of enforcement and which, if pertinent, shall appoint the intervener, shall be submitted for consideration by the National Congress or the State Legislature within twenty-four hours.

Paragraph 2. If the National Congress or the State Legislature are not in session, a special session shall be called within the same twenty-four hours.

Paragraph 3. In the case of Article 34, items VI and VII, or Article 35, item IV, when the consideration by the National Congress or the State Legislature may be waived, the decree shall be limited to suspending the enforcement of the challenged act, if such measure suffices to restore normality.

Paragraph 4. Upon cessation of the reasons that caused the intervention, authorities removed from offices shall return to them, unless there is some legal impediment.

CHAPTER VII
Public Administration

SECTION I
General Provisions

Article 37. The government bodies and its associated entities in any of the branches of the Union, states, Federal District and municipalities shall obey the principles of legality, impersonality, morality, publicity, and efficiency, and also the following: (CA 19, 1998)

I – public offices, positions and functions are accessible to all Brazilians who meet the requirements established by law, as well as to foreigners, as provided by law; (CA 19, 1998)

II – investiture in public office or position depends on prior approval in a competitive civil-service examination consisting of tests or tests and presentation of academic and professional credentials, according to the nature and complexity of the office or position, as provided by law, except for appointment to a commission office declared by law as being of free appointment and discharge; (CA 19, 1998)

III – a competitive civil-service examination shall be valid up to two years, extendable once for an equal period;

IV – during the inextensible period established in the public call notice, a person who has passed a competitive civil-service examination of tests, or of tests and presentation of

academic and professional credentials, shall be called with priority over newly approved applicants, to take an office or position in the career;

V – positions of trust, exercised exclusively by civil servants holding an effective post, and commission offices, to be filled by career employees in the cases, conditions and minimum percentages established by law, are reserved exclusively for the duties of directors, chiefs of staff, and assistants; (CA 19, 1998)

VI – the right to free union association is guaranteed to civil servants;

VII – the right to strike shall be exercised in the manner and within the limits defined by a specific law; (CA 19, 1998)

VIII – the law shall reserve a percentage of public offices and positions for handicapped people and shall define the criteria for their admittance;

IX – the law shall establish the cases to hire for a fixed period to meet a temporary need of exceptional public interest;

X – the remuneration of civil servants and the compensation referred to in paragraph 4 of Article 39 may only be established or altered by means of a specific law, with due regard for the exclusive capacity to introduce a law in each case, an annual general review being ensured, always on the same date and without distinction with respect to the indexes; (CA 19, 1998)

XI – remuneration and compensation of holders of public offices, functions, and positions in the government bodies, agencies, and foundations; of the members of any of the branches of the Union, states, Federal District, and municipalities; of the holders of elective offices, and of any other political agent, as well as the salary, pension, or other type of remuneration, earned on a cumulative basis or not, including personal advantages or those of any other nature, shall not exceed the monthly compensation, in specie, of the justices of the Federal Supreme Court, and the following limits shall be applied: in municipalities, the compensation of the mayor; in the states and the Federal District, in the executive branch, the monthly compensation of the governor; in the legislative branch, the compensation of state and Federal District deputies; and in the judicial branch, the compensation of the judges of the Court of Appeals, limited to ninety and twenty-five hundredths percent of the monthly compensation, in specie, of the justices of the Federal Supreme Court, in the judicial branch, this limit being also applicable to the members of the Prosecution Office, state attorneys, and public defenders; (CA 41, 2003)

XII – the salaries for positions of the legislative and judicial branches may not be higher than those paid by the executive branch;

XIII – linking or equating any kind of remuneration is prohibited for purposes of compensating public service personnel; (CA 19, 1998)

XIV – pecuniary raises received by civil servants shall not be computed or accumulated for purposes of granting subsequent raises; (CA 19, 1998)

XV – compensation and salaries of holders of public offices and positions may not be reduced, except for the provisions of items XI and XIV of this Article and of Articles 39, paragraph 4; 150, item II; 153, item III; and 153, paragraph 2, item I; (CA 19, 1998)

XVI – accumulation of remunerated public offices is forbidden, except for the following cases, if working hours are compatible, and with due regard, in any case, for the provision of item XI: (CA 19, 1998)

a) of two teaching positions; (CA 19, 1998)

b) of one teaching position with another technical or scientific position; (CA 19, 1998)

c) of two positions or jobs which are exclusive for health professionals, with regulated professions; (CA 34, 2001)

XVII – the prohibition against accumulation extends to positions and functions and includes government agencies, foundations, state-owned companies, mixed-capital companies, their subsidiary companies, and companies controlled, either directly or indirectly, by the government; (CA 19, 1998)

XVIII – the tax authority and its revenue officers shall, within their spheres of authority and jurisdiction, have the right of precedence over other administrative sectors, as provided by law;

XIX – creation of government agencies and authorization to establish state-owned companies, mixed-capital companies, and foundations may only be accomplished by a specific law; in the latter case, a supplementary law shall specify the areas they may operate; (CA 19, 1998)

XX – in each case creation of subsidiaries of the entities mentioned in the preceding item depends on legislative authorization, as well as the participation by any of them in a private company;

XXI – with the exception of the cases specified in law, public works, services, purchases and disposals shall be contracted by public bidding proceedings that ensure equal conditions to all bidders, with clauses that establish payment obligations, maintaining the effective conditions of the bid, as the law provides, which shall only allow the requirements of technical and economic qualifications indispensable to guarantee the fulfillment of the obligations;

XXII – the tax authorities of the Union, states, Federal District, and municipalities, whose activities are essential for the operation of the State and are exercised by civil servants of specific careers, shall have priority funds to perform their activities and shall work in an integrated manner, including sharing tax records and fiscal information, as provided by law or covenant. (CA 42, 2003)

Paragraph 1. The publicity of the acts, programs, public works, services and campaigns of government bodies shall be of educational, informative or social orientation character, and shall not contain names, symbols or images that characterize personal propaganda of government authorities or employees.

Paragraph 2. Non-compliance with the provisions of items II and III shall result in the nullity of the act and punishment of the responsible authority, as provided by law.

Paragraph 3. The law shall regulate the forms of user participation in the government bodies and associated entities, regarding especially: (CA 19, 1998)

I – claims relating to the provision of public services in general, the provision of user services being ensured, as well as periodical assessment, both external and internal, of the quality of services; (CA 19, 1998)

II – user access to administrative records and to information about government initiatives, with due regard for Article 5, items X and XXXIII; (CA 19, 1998)

III – the rules of a complaint against negligence or abuse in the exercise of an office, position or function in government services. (CA 19, 1998)

Paragraph 4. Acts of administrative improbity shall result in the suspension of political rights, loss of public function, prohibition to transfer personal property and reimbursement to the Treasury, in the manner and grading established by law, without prejudice to the applicable criminal action.

Paragraph 5. The law shall establish the statute of limitations for unlawful acts performed by any agent, whether a civil servant, which may damage the Treasury, without prejudice to the respective claims for reimbursement.

Paragraph 6. Public legal entities and private legal entities rendering public services shall be liable for damage that any of their agents, acting as such, cause to third parties, ensuring the right of recourse against the liable agent in cases of fraud or fault.

Paragraph 7. The law shall establish the requirements and restrictions regarding the holder of an office or position in the government bodies and associated entities which provide access to inside information. (CA 19, 1998)

Paragraph 8. The managerial, budget and financial autonomy of the government bodies and associated entities may be extended by means of a contract, to be entered into by their

administrators and the government, with a view to establish the performance goals for the government bodies or associated entities, and the law shall provide for: (CA 19, 1998)

I – the term of the contract; (CA 19, 1998)

II – controls and criteria for the appraisal of performance, rights, duties, and liability of managing officers; (CA 19, 1998)

III – the remuneration of the employees. (CA 19, 1998)

Paragraph 9. The provision of item XI applies to the state-owned and mixed-capital companies and their subsidiaries which receive funds from the Union, states, Federal District, or municipalities for payment of personnel expenditures or general expenses. (CA 19, 1998)

Paragraph 10. Receiving retirement benefits arising from Article 40 or from Articles 42 and 142, while at the same time receiving the remuneration of a public office, position or function is forbidden, with the exception of offices that may be accumulated according to this Constitution, elective offices, and commission offices declared by law as being of free appointment and discharge. (CA 20, 1998)

Paragraph 11. The compensatory amounts set forth in law shall not be computed for the purposes of the remuneration limits referred to in item XI of the head provision. (CA 47, 2005)

Paragraph 12. For the purposes provided by item XI of the head provision , the states and the Federal District may stipulate, within their jurisdiction, by means of an Amendment to their respective Constitutions and Organic Law, as a single limit, the monthly compensation of the judges of the respective Court of Appeals, limited to ninety and twenty-five hundredths percent of the monthly compensation of the justices of the Federal Supreme Court, and the provision of this paragraph shall not be applied to the compensation of the state and Federal District deputies and municipal councilors. (CA 47, 2005)

Paragraph 13. Civil servants holding an effective post may be reassigned to a position holding duties and responsibilities that meet the physical or mental impairment they have suffered, while remaining in this condition, as long as they qualify and hold the required educational level for such a job; the remuneration from the prior position must be preserved. (CA 103, 2019)

Paragraph 14. The retirement granted on grounds of the period of contribution due to a public position, job or office, including the general social security regime, shall terminate the bond that provided the mentioned period of contribution. (CA 103, 2019)

Paragraph 15. It is forbidden to supplement retirement benefits of civil servants and death pensions to their dependents in cases other than the one established under

paragraphs 14 to 16 of Article 40 or in case they are not provided for under a law that extinguishes the special social security scheme. (CA 103, 2019)

Article 38. The following provisions are applicable to civil servants holding elective offices in government bodies, agencies and foundations: (CA 19, 1998)

I – in the case of a federal, state or district elective office, they shall leave their office, position or function;

II – if sworn into office as mayor, they shall take leave from post, position or function and may opt for the corresponding remuneration;

III – if sworn into office as municipal councilor, where the working hours are compatible, they shall receive the benefits of their post, position or function, without prejudice to the remuneration of the elective office and where there is no such compatibility, the provisions of the preceding item shall be applied;

IV – in any case requiring leave of absence due to exercise of an elective office, their time of service shall be counted in full, for all legal effects, except for promotion by merit;

V – in the event of being insured by the special social security scheme, the civil servant shall remain affiliated to such scheme in the respective federal entity. (CA 103, 2019)

SECTION II

Government Employees (CA 18, 1998)

Article 39. The Union, states, Federal District and municipalities shall institute a board of administration policy and personnel remuneration policy, composed of civil servants appointed by the respective branches.

Paragraph 1. Stipulation of pay levels and other components of the remuneration system shall comply with: (CA 19, 1998)

I – the nature, level of responsibility, and complexity of the posts of each career; (CA 19, 1998)

II – the requirements for holding the post; (CA 19, 1998)

III – the specific characteristics of each post. (CA 19, 1998)

Paragraph 2. The Union, states, and Federal District shall maintain government schools for the education and further development of civil servants, and participation in such courses shall be one of the requirements for promotion in the career. For this purpose, entry into agreements or contracts among federal entities is allowed. (CA 19, 1998)

Paragraph 3. The provisions of Article 7, items IV, VII, VIII, IX, XII, XIII, XV, XVI, XVII, XVIII, XIX, XX, XXII, and XXX, shall apply to employees holding public offices, and the

law may stipulate differentiated requirements for admission when the nature of the office so demands. (CA 19, 1998)

Paragraph 4. A member of one of the branches, holder of an elective office, ministers of State, and secretary of a state or municipality shall be remunerated exclusively by means of a compensation consisting of one sole item; the addition of any extra benefit, additional pay, bonus, award, representation allowance, or other type of remuneration being forbidden, with due regard, in any of the cases, for the provisions of Article 37, items X and XI. (CA 19, 1998)

Paragraph 5. The legislation of the Union, states, Federal District, and municipalities may establish the proportion between the highest and lowest remuneration of civil servants, with due regard, in any case, for the provision of Article 37, item XI. (CA 19, 1998)

Paragraph 6. The executive, legislative and judicial branches shall disclosure annually the compensation and remuneration of public offices and positions. (CA 19, 1998)

Paragraph 7. The legislation of the Union, states, Federal District, and municipalities shall regulate the utilization of the budget funds deriving from savings in current expenditures in each government body, agency and foundation, to be used in the development of programs of quality and productivity, training and development, modernization, re-equipping and rationalization of public services, including as additional pay or productivity bonus. (CA 19, 1998)

Paragraph 8. The remuneration of civil servants organized in a career may be established under the terms of paragraph 4. (CA 19, 1998)

Paragraph 9. It is forbidden to incorporate into the remuneration of the effective post benefits arising from the exercise of positions of trust or commission offices, as well as temporary benefits. (CA 103, 2019)

Article 40. The special social security scheme of employees holding effective post shall have a contributory and solidary basis, with contributions from the respective federal entity, from the current employees, retired personnel and pensioners, with due regard for criteria that preserve financial and actuarial balance. (CA 103, 2019)

Paragraph 1. The employees covered by the special social security scheme set forth under this Article shall retire: (CA 103, 2019)

I – due to permanent disability for work, in the position which they are assigned to, when unsuitable for reassignment, in which case periodic evaluations will be required to assess whether the conditions that enabled the retirement are still present, as provided for by the law of the respective federal entity; (CA 103, 2019)

II – compulsorily, with a pension proportional to the period of contribution, at the age of 70 (seventy), or at the age of 75 (seventy-five), as provided in supplementary law; (CA 88, 2015)

III – within the Union, at the age of 62 (sixty-two), if female, and at 65 (sixty-five), if male, and within the states, Federal District, and municipalities, at the minimum age established by Amendment to the respective Constitutions and Organic Laws, subject to the period of contribution and other requirements established by a supplementary law of the respective federal entity. (CA 103, 2019)

Paragraph 2. The retirement pensions may not be lower than the minimum value referred to in paragraph 2 of Article 201 or above the maximum limit established for the general social security regime, provided for in paragraphs 14 to 16. (CA 103, 2019)

Paragraph 3. The rules for calculating retirement benefits shall be regulated by the law of the respective federal entity. (CA 103, 2019)

Paragraph 4. It is forbidden to adopt different requirements or criteria to grant benefits under the special social security scheme, except for the cases under paragraphs 4-A, 4-B, 4-C and 5. (CA 103, 2019)

Paragraph 4-A. The respective federal entity may establish under supplementary law a different age and period of contribution for the employees with disabilities to retire, if they submit to a biopsychosocial assessment performed by a multi professional and interdisciplinary team. (CA 103, 2019)

Paragraph 4-B. The respective federal entity may establish, under supplementary law, a different age and period of contribution for the retirement of penitentiary agents, socio-educational agents or police officers of the bodies mentioned in item IV of the head provision of Article 51, in item XIII of the head provision of Article 52 and in items I to IV of the head provision of Article 144. (CA 103, 2019)

Paragraph 4-C. The respective federal entity may establish under a supplementary law a differentiated age and period of contribution for the retirement of employees whose activities are performed with effective exposure to chemical, physical and biological agents harmful to health, or association to these agents; the characterization by professional or occupation category, in that case, is forbidden. (CA 103, 2019)

Paragraph 5. The employees in the position of a teacher shall have the minimum age reduced by five (5) years in relation to the ages of the provisions of item III of paragraph 1, as long as they document effective time of exercising teaching functions in early childhood education and in elementary and secondary education, according to the supplementary law of the respective federal entity. (CA 103, 2019)

Paragraph 6. Except for retirements from posts that may be accumulated under the provisions of this Constitution, it is forbidden to pay more than one retirement pension under the special social security scheme, which is also subject to other rules, conditions and prohibitions against accumulation of social security benefits established under the general social security regime. (CA 103, 2019)

Paragraph 7. With due regard to the provisions of paragraph 2 of Article 201, when it is the only formal source of income earned by the dependent, the death benefit pension will be granted in accordance with the law of the respective federal entity, which will differentiate the cases of death of employees referred to in paragraph 4-B due to aggression suffered while performing their duties or due to their office. (CA 103, 2019)

Paragraph 8. Readjustment of the benefits is ensured, to the end that their real value is permanently maintained, in accordance with criteria established by law. (CA 41, 2003)

Paragraph 9. The period of contribution in a federal, state, district or municipal post shall count for retirement purposes, subject to the provision of paragraphs 9 and 9-A of Article 201, and the corresponding period of service shall count for the purpose of placement on paid availability. (CA 103, 2019)

Paragraph 10. The law may not establish any method of computation of fictitious periods of contribution. (CA 20, 1998)

Paragraph 11. The limit set forth in Article 37, item XI, applies to the total amount of the retirement pension and other pensions, including those resulting from the accumulation of public posts or positions, as well as from other activities which must contribute to the general social security regime, and to the amount resulting from the addition of pensions and the remuneration of a post which may be accumulated under the terms of this Constitution, a commission office declared by law as being of free appointment and discharge, and an elective office. (CA 20, 1998)

Paragraph 12. In addition to the provisions of this Article, the special social security scheme shall comply, whenever appropriate, with the requirements and criteria stipulated for the general social security regime. (CA 103, 2019)

Paragraph 13. The general social security regime covers employees who hold exclusively commission offices declared by law as being of free appointment and discharge, as well as other temporary posts, including an elective office, or public positions. (CA 103, 2019)

Paragraph 14. The Union, states, Federal District, and municipalities will establish by a law proposed by each respective executive branch, a supplementary social security scheme for their respective employees who hold effective posts; the retirement and pension benefits of such special social security scheme shall be subject to the cap of retirement

pensions and other pensions established under the general social security regime, except for the case under paragraph 16. (CA 103, 2019)

Paragraph 15. The supplementary social security scheme referred to in paragraph 14 will offer benefit plans exclusively in the defined contribution mode, with due regard for the provisions of Article 202, and will be offered either by closed complementary pension fund or open complementary pension fund entities. (CA 103, 2019)

Paragraph 16. Only by prior and express option may the provisions of paragraphs 14 and 15 be applied to an employee who has entered public administration by the publication date of the act which instituted the corresponding complementary social security scheme. (CA 20, 1998)

Paragraph 17. All remuneration amounts considered in the calculation of the benefit established under paragraph 3 shall be duly updated, as provided by law. (CA 41, 2003)

Paragraph 18. A contribution shall be levied on retirement pensions and other pensions granted by the scheme referred to in this Article if such pensions exceed the maximum limit established for the benefits of the general social security regime mentioned in Article 201, at a percentage equal to the one established for employees holding effective posts. (CA 41, 2003)

Paragraph 19. Subject to the criteria to be established by law of the respective federal entity, the employees who hold effective posts who have fulfilled the requirements for voluntary retirement and who choose to remain working shall be entitled to a continuous activity bonus equivalent to, at most, the amount of their social security contribution, until they reach the age of compulsory retirement. (CA 103, 2019)

Paragraph 20. It is forbidden to establish more than one special social security scheme and more than one body or unit to manage the respective scheme in each federal entity, including all the branches, government bodies, agencies and foundations that will be responsible for its financing, subject to the criteria, parameters and legal nature determined in the supplementary law referred to in paragraph 22. (CA 103, 2019)

Paragraph 21. (Repealed) (CA 103, 2019)

Paragraph 22. It is forbidden to establish new special social security schemes; a federal supplementary law will provide for the general rules of organization, operation and management liability to the ones already in force, and will set forth, among other aspects: (CA 103, 2019)

I – requirements for its extinction and consequent migration to the general social security regime; (CA 103, 2019)

II – model of collection, application and use of resources; (CA 103, 2019)

III – federal inspection and external and social control; (CA 103, 2019)

IV – definition of financial and actuarial balance; (CA 103, 2019)

V – conditions to establish the fund with social security purposes referred to in Article 249 and for earmarking contributions and goods, rights and assets of any kind to it funds; (CA 103, 2019)

VI – actuarial deficit equation mechanisms; (CA 103, 2019)

VII – structuring of the scheme management body or entity, complying with the principles related to governance, internal control and transparency; (CA 103, 2019)

VIII – conditions and assumptions for the accountability of those who perform duties related, directly or indirectly, to the scheme management; (CA 103, 2019)

IX – conditions for joining a public consortium; (CA 103, 2019)

X – parameters to determine the tax base and the rate definition of ordinary and extraordinary contributions. (CA 103, 2019)

Article 41. Civil servants who, as a result of a competitive civil-service examination, are appointed to effective posts, acquire tenure after three years of actual service. (CA 19, 1998)

Paragraph 1. A tenured civil servant shall only lose office: (CA 19, 1998)

I – as a result of a final and unappealable judicial decision; (CA 19, 1998)

II – by means of an administrative proceeding, in which full defense is ensured; (CA 19, 1998)

III – by means of a procedure of periodical appraisal of performance, under the terms of a supplementary law, full defense being ensured. (CA 19, 1998)

Paragraph 2. If the dismissal of a tenured civil servant is voided by a judicial decision, the servant shall be reinstated, and the occupant of the vacancy, when tenured, shall be led back to the original office, with no right to indemnity, taken to another office or placed on paid availability with a remuneration proportional to the length of employment. (CA 19, 1998)

Paragraph 3. If the office is declared extinct or unnecessary, a tenured civil servant shall remain on availability, with remuneration proportional to the length of employment, until being placed in another office. (CA 19, 1998)

Paragraph 4. As a requirement to acquire tenure, a special appraisal of performance by a committee created for this purpose is mandatory. (CA 19, 1998)

SECTION III

The Military of the States, of the Federal District and of the Territories (CA 18, 1998)

Article 42. The members of the military police and of the military fire brigades, institutions whose organization is based on hierarchy and discipline, are military of the states, Federal District and Territories. (CA 18, 1998)

Paragraph 1. The provisions of Article 14, paragraph 8; Article 40, paragraph 9; and of Article 142, paragraphs 2 and 3, apply to the military of the states, Federal District, and Territories, in addition to other provisions that the law may establish, it being incumbent upon a specific state legislation to provide for the matters of Article 142, paragraph 3, item X, the ranks of the officers being awarded by the respective state governors. (CA 20, 1998)

Paragraph 2. The provisions that may be established by a specific act of the respective state shall apply to the pensioners of the military of the states, Federal District, and Territories. (CA 41, 2003)

Paragraph 3. The provisions of Article 37, item XVI, with the prevalence of military activity, shall apply to the military of the states, Federal District and Territories. (CA 101, 2019)

SECTION IV

The Regions

Article 43. For administrative purposes, the Union may coordinate its action in one same social and geo-economic complex, seeking to attain its development and to reduce regional inequalities.

Paragraph 1. A supplementary law shall provide for:

I – the conditions for the integration of developing regions;

II – the composition of the regional agencies which shall carry out, as provided by law, the regional plans included in the national social and economic development plans approved concurrently.

Paragraph 2. The regional incentives shall include, inter alia, as provided by law:

I – equality of tariffs, freight rates, insurance and other cost and price items which are within the responsibility of the government;

II – favored interest rates for the financing of priority activities;

III – exemptions, reductions or temporary deferment of federal taxation owed by individuals or by legal entities;

IV – priority in the economic and social use of rivers and dammed water masses or areas with a potential to form water reservoirs in low-income regions subject to periodical droughts.

Paragraph 3. In the areas referred to in paragraph 2, item IV, the Union shall grant incentives to recover the arid lands and shall cooperate with small and medium-size rural landowners to implement water sources and small-scale irrigation in their tracts of land.

TITLE IV

Organization of the Branches (CA 80, 2014)

CHAPTER I

The Legislative Branch

SECTION I

The National Congress

Article 44. The legislative power is vested in the National Congress, which is composed of the Chamber of Deputies and the Federal Senate.

Single paragraph. Each legislative term shall have the duration of four years.

Article 45. The Chamber of Deputies is composed of representatives of the people, elected, by the proportional system, in each state, Territory and in the Federal District.

Paragraph 1. The total number of deputies, as well as the representation of the states and of the Federal District shall be established by a supplementary law, in proportion to the population, and the necessary adjustments shall be made in the year preceding the elections, so that none of those federal entities has less than eight or more than seventy deputies.

Paragraph 2. Each Territory shall elect four deputies.

Article 46. The Federal Senate is composed of representatives of the states and of the Federal District, elected by a majority vote.

Paragraph 1. Each state and the Federal District shall elect three senators for an eight-year term of office.

Paragraph 2. One-third and two-thirds of the representation of each state and of the Federal District shall be renewed every four years, alternately.

Paragraph 3. Each senator shall be elected with two substitutes.

Article 47. Except where there is a constitutional provision to the contrary, the decisions of each House and of their committees shall be taken by a majority vote, when an absolute majority of its members is present.

SECTION II

Powers of the National Congress

Article 48. The National Congress shall have the power, with the sanction of the president of the Republic, which shall not be required for the matters specified in Articles 49, 51 and 52, to provide for all the matters within the competence of the Union and especially on:

I – system of taxation, collection of taxes and income distribution;

II – pluriannual plan, budget directives, annual budget, credit transactions, public debt and issuance of currency;

III – establishment and modification of Armed Forces troops;

IV – national, regional and sectorial plans and programs of development;

V – boundaries of the national territory, air and maritime space and property of the Union;

VI – incorporation, subdivision or dismemberment of areas of Territories or states, after consulting with the respective State Legislatures;

VII – temporary transference of the seat of the Union;

VIII – granting of amnesty;

IX – administrative and judicial organization, organization of the Prosecution Office and of the Office of the Public Defender of the Union and of the Territories, and judicial organization as well as organization of the Prosecution Office of the Federal District; (CA 69, 2012)

X – creation, change, and elimination of public offices, positions and functions, with due regard for Article 84, item VI, subitem *b*; (CA 32, 2001)

XI – creation and extinction of Ministries and government bodies; (CA 32, 2001)

XII – telecommunications and radio broadcasting;

XIII – financial, foreign exchange and monetary matters, financial institutions and their operations;

XIV – currency, currency issuance limits, and amount of federal indebtedness;

XV – stipulation of the compensation for the justices of the Federal Supreme Court, with due regard for Articles 39, paragraph 4; 150, item II; 153, item III; and 153, paragraph 2, item I. (CA 41, 2003)

Article 49. The National Congress has exclusive power:

I – to decide conclusively on international treaties, agreements or acts that result in charges or commitments that are onerous to the national property;

II – to authorize the president of the Republic to declare war, to make peace and to permit foreign forces to pass through the national territory or remain therein temporarily, with the exception of the cases provided by a supplementary law;

III – to authorize the president and the vice-president of the Republic to leave the country, when such absence exceeds fifteen days;

IV – to approve a state of defense and federal intervention, to authorize a state of siege or suspend any of these measures;

V – to stop the normative acts of the executive branch which exceed their regulatory power or the limits of legislative delegation;

VI – to transfer its seat temporarily;

VII – to establish identical compensation for federal deputies and senators, taking into account the provisions of Articles 37, item XI, 39, paragraph 4, 150, item II, 153, item III, and 153, paragraph 2, item I; (CA 19, 1998)

VIII – to establish the compensation of the president and the vice-president of the Republic and of the ministers of State, taking into account the provisions of Articles 37, item XI, 39, paragraph 4, 150, item II, 153, item III, and 153, paragraph 2, item I; (CA 19, 1998)

IX – to examine each year the accounts rendered by the president of the Republic and to consider the reports on the execution of government plans;

X – to supervise and control directly or through either of its Houses, the acts of the executive branch, including those of the direct bodies and associated entities;

XI – to ensure the preservation of legislative competence in the face of the normative incumbency of the other branches;

XII – to consider the acts of concession and renewal of concession of radio and television stations;

XIII – to choose two-thirds of the members of the Federal Accounting Court;

XIV – to approve initiatives of the executive branch referring to nuclear activities;

XV – to authorize a *referendum* and to call a plebiscite;

XVI – to authorize, in indigenous lands, the exploitation and use of hydric resources and the prospecting and mining of mineral resources;

XVII – to give prior approval to the disposal or concession of public lands with an area of over two thousand and five hundred hectares.

Article 50. The Chamber of Deputies and the Federal Senate, or any of their committees, may summon a minister of State or any chief officers of agencies directly subordinate to the Presidency of the Republic to personally render information on a previously determined matter, and this absence without adequate justification shall constitute a crime of responsibility. (RCA 2, 1994)

Paragraph 1. The ministers of State may attend the Federal Senate, the Chamber of Deputies or any of their committees, on their own initiative and by agreement with the respective Directing Board, to report on a matter of relevance to their Ministry.

Paragraph 2. The Directing Boards of the Chamber of Deputies and of the Federal Senate may forward to the ministers of State, or any of the people mentioned in the head provision , written requests for information to be rendered within a period of thirty days, and refusal or non-compliance, as well as the rendering of false information, shall constitute crime of responsibility. (RCA 2, 1994)

SECTION III

The Chamber of Deputies

Article 51. The Chamber of Deputies has exclusive power:

I – to authorize the commencement of impeachment proceedings against the president, the vice-president of the Republic and the minister of State, by vote of two-thirds of its members;

II – to proceed to take the accounts of the president of the Republic, when they are not presented to the National Congress within sixty days of the opening of the legislative session;

III – to elaborate its internal regulations;

IV – to provide for its organization, functioning, police, creation, change or elimination of offices, positions and functions of its services, and the introduction of a law for the establishment of their respective remuneration, taking into account the guidelines set forth in the budget directives law; (CA 19, 1998)

V – to elect the members of the Council of the Republic, in the manner prescribed by Article 89, item VII.

SECTION IV
The Federal Senate

Article 52. The Federal Senate has exclusive power:

I – to preside over and try the president and the vice-president of the Republic for crime of responsibility, and the ministers of State and the commanders of the Navy, Army, and Air Force for crimes of the same nature relating to those; (CA 23, 1999)

II – to preside over and try the justices of the Federal Supreme Court, members of the National Council of Justice and National Council of the Prosecution Office, the federal attorney general, and the general counsel to the federal government charged with crimes of responsibility; (CA 45, 2004)

III – to give prior consent, by secret voting, after public hearing, on the selection of:

a) judges, in the cases established under this Constitution;

b) justices of the Federal Accounting Court appointed by the president of the Republic;

c) governor of a Territory;

d) president and directors of the Central Bank;

e) federal attorney general;

f) holders of other offices, as the law may provide;

IV – to give prior approval, by secret voting, after closed hearing, on the selection of heads of permanent diplomatic missions;

V – to authorize foreign transactions of a financial nature, of the interest of the Union, states, Federal District, Territories and municipalities;

VI – to establish, as proposed by the president of the Republic, total limits for the entire amount of the consolidated debt of the Union, states, Federal District and municipalities;

VII – to provide for the total limits and conditions for foreign and domestic credit transactions of the Union, states, Federal District and municipalities, of their agencies and other entities controlled by the Union;

VIII – to provide for limits and conditions for the concession of a guarantee by the Union in foreign and domestic credit transaction;

IX – to establish total limits and conditions for the entire amount of the government-bond debt of the states, Federal District and municipalities;

X – to suspend the application, in full or in part, of a law declared unconstitutional by final decision of the Federal Supreme Court;

XI – to approve, by an absolute majority and by secret voting, the removal from office of the federal attorney general before the term of office ends;

XII – to elaborate its internal regulations;

XIII – to provide for its organization, functioning, police, creation, change or elimination of offices, positions and functions of its services, and the introduction of a law for the establishment of their respective remuneration, taking into account the guidelines set forth in the budget directives law; (CA 19, 1998)

XIV – to elect the members of the Council of the Republic, as established in Article 89, item VII;

XV – to carry out a regular assessment of the functionality of the national tax system, its structure and components, as well as the performance of the tax authorities of the Union, states, Federal District, and municipalities. (CA 42, 2003)

Single paragraph. In the cases provided for in items I and II, the chief justice of the Federal Supreme Court shall preside and a conviction, which may only be rendered by two-thirds vote of the Federal Senate, shall be limited to the loss of office with a ban to hold any public office for a period of eight years, without prejudice to any other judicial sanctions that may be applicable.

SECTION V

Deputies and Senators

Article 53. Deputies and senators enjoy civil and criminal immunity on account of any of their opinions, words and votes. (CA 35, 2001)

Paragraph 1. Deputies and senators, from the date of issuance of the certificate of election victory, shall be tried by the Federal Supreme Court. (CA 35, 2001)

Paragraph 2. From the date of issuance of the certificate of election victory, the members of the National Congress may not be arrested, except in flagrante delicto of an offense not subject to bail. In such event, the case records shall be sent within twenty-four hours to the respective House, which, by a majority vote of its members, shall decide on the arrest. (CA 35, 2001)

Paragraph 3. Upon receiving an accusation against a senator or deputy, for an offense committed after the issuance of the certificate of election victory, the Federal Supreme Court shall inform the respective House, which, by the initiative of a political party therein represented and by a majority vote of those House members, may, until a final decision is issued, suspend the proceeding. (CA 35, 2001)

Paragraph 4. The request for suspension shall be examined by the respective House within the non-extendable period of forty-five days from its receipt by the Directing Board. (CA 35, 2001)

Paragraph 5. The suspension of proceedings shall suspend the statute of limitation for the duration of the term of office. (CA 35, 2001)

Paragraph 6. Deputies and senators shall not be compelled to render testimony on information received or given due to the exercise of their office, nor on people who rendered them information or received information from them. (CA 35, 2001)

Paragraph 7. Incorporation into the Armed Forces of deputies and senators, even if they hold military rank and even in time of war shall depend upon the prior granting of permission by the respective House. (CA 35, 2001)

Paragraph 8. The immunities of deputies and senators shall be maintained during a state of siege and may only be suspended by vote of two-thirds of the members of the respective House, in cases of acts committed outside the premises of the National Congress, which are not compatible with the implementation of such a measure. (CA 35, 2001)

Article 54. Deputies and senators may not:

I – after the issuance of their certificate of electoral victory:

a) sign or maintain a contract with a public legal entity, agency, state-owned company, mixed-capital company or public-utility company, unless the contract is in accordance with uniform clauses;

b) accept or hold a paid office, function or position including those from which they may be dismissed *ad nutum* in the entities mentioned in the preceding subitem;

II – after taking office:

a) be the owners, controllers or directors of a company which enjoys benefits arising from a contract with a public legal entity or perform a remunerated position therein;

b) hold an office or function from which they may be dismissed *ad nutum*, in the entities mentioned in item I, subitem *a*;

c) act as a lawyer in a cause in which any of the entities referred to in item I, subitem *a*, has an interest;

d) be the holders of more than one public elective position or office.

Article 55. A deputy or senator shall lose office:

I – if they violate any of the prohibitions established in the preceding Article;

II – if their conduct is declared incompatible with parliamentary decorum;

III – if they fail to appear, in each legislative session, at one-third of the regular sessions of the House to which they belong, except for a leave of absence or a mission authorized by the House concerned;

IV – if they have been stripped of their political rights or if these rights have been suspended;

V – whenever decreed by the Electoral Courts, in the cases established in this Constitution;

VI – if they are criminally convicted by a final and unappealable sentence.

Paragraph 1. Abuse of the prerogatives ensured to a congress member or the gaining of undue advantages, in addition to the cases defined in the internal regulations, is incompatible with parliamentary decorum.

Paragraph 2. In the cases of items I, II and VI, loss of office shall be declared by the Chamber of Deputies or the Federal Senate, by an absolute majority, on the initiative of the respective Directing Board or of a political party represented in the National Congress, full defense being ensured. (CA 76, 2013)

Paragraph 3. In the cases set forth in items III to V, the loss shall be declared by the Directing Board of the respective House, on its own initiative or upon the initiative of any of its members, or of a political party represented in the National Congress, full defense being ensured.

Paragraph 4. The resignation of a congress member submitted to a legal suit that aims at or may lead to loss of office, under the provisions of this Article, will have its effects suspended until the final deliberations mentioned in paragraphs 2 and 3. (RCA 6, 1994)

Article 56. A deputy or senator shall not lose office:

I – if vested with the office of minister of State, governor of a Territory, secretary of a member state, of the Federal District, of a Territory, of a state capital or head of a temporary diplomatic mission;

II – if on leave of absence from the respective House, by virtue of illness or, without remuneration, to attend private matters, provided that, in this case, the absence does not exceed one hundred and twenty days per legislative session.

Paragraph 1. The substitute shall be called in cases of vacancy, of investiture in the functions set forth in this Article or of leave of absence exceeding one hundred and twenty days.

Paragraph 2. Upon the occurrence of a vacancy and there being no substitute, if more than fifteen months remain before the end of the term of office, an election shall be held to fill it.

Paragraph 3. In the event of item I, the deputy or senator may opt for the remuneration of the elective office.

SECTION VI
The Sessions

Article 57. The National Congress shall meet each year in the Federal Capital, from February 2 to July 17 and from August 1 to December 22. (CA 50, 2006)

Paragraph 1. If sessions scheduled for these dates fall on a Saturday, Sunday or a holiday, they shall be transferred to the subsequent workday.

Paragraph 2. The legislative session shall not be interrupted before the approval of the bill of budget directives.

Paragraph 3. In addition to other cases provided for in this Constitution, the Chamber of Deputies and the Federal Senate shall meet in a joint session to:

I – inaugurate the legislative session;

II – elaborate the common regulations and regulate the creation of services common to both Houses;

III – take the oath of the president and vice-president of the Republic;

IV – acknowledge a veto and resolve thereon.

Paragraph 4. Both Houses shall meet in a preparatory session, beginning February 1 of the first year of the legislative term, for the induction of its members and the election of the respective Directing Boards, for a two-year term of office, the re-election to the same office in the immediately subsequent election being prohibited. (CA 50, 2006)

Paragraph 5. The Directing Board of the National Congress shall be presided by the president of the Federal Senate and the remaining offices shall be held, alternately, by the holders of equivalent offices in the Chamber of Deputies and in the Federal Senate.

Paragraph 6. Special sessions of the National Congress shall be called: (CA 50, 2006)

I – by the president of the Federal Senate, in the event of a decree of a state of defense or of federal intervention, of a demand for the authorization to decree a state of siege and the taking of oath and inauguration of the president and vice-president of the Republic;

II – by the president of the Republic, by the presidents of the Chamber of Deputies and of the Federal Senate, or by request of the majority of the members of both Houses, in the event of urgency or important public interest, approval by absolute majority of each House of the National Congress being required in all cases referred to in this item. (CA 50, 2006)

Paragraph 7. In a special legislative session, the National Congress shall deliberate only upon the matter for which it was called, exception being made for the event mentioned in paragraph 8 of this Article, the payment of a compensatory amount for the special session being forbidden. (CA 50, 2006)

Paragraph 8. If there are provisional presidential decrees in effect on the date a special session of the National Congress is called, they shall be automatically included in the agenda of the session. (CA 32, 2001)

SECTION VII
The Committees

Article 58. The National Congress and both its Houses shall have permanent and temporary committees, established in the manner and with the incumbencies set forth in the respective regulations or in the act from which their creation resulted.

Paragraph 1. In the composition of the Directing Boards and of each committee, the proportional representation of the parties or the parliamentary groups which participate in the respective House shall be ensured to the extent possible.

Paragraph 2. The committees have the power, on account of the matter under their authority:

I – to debate and vote on bills of law which, in accordance with the regulations, are exempt from being submitted to the Plenary Assembly, except in the event of an appeal from one-tenth of the members of the respective House;

II – to hold public audiences with entities of civil society;

III – to summon ministers of State to render information on matters inherent to their duties;

IV – to receive petitions, claims, statements or complaints from any person against acts or omissions of government authorities or entities;

V – to request the testimony of any authority or citizen;

VI – to examine construction work programs and national, regional and sectorial development plans and to report thereupon.

Paragraph 3. Parliamentary inquiry committees, which shall have the powers of investigation inherent in the judicial authorities, in addition to other powers set forth in the regulations of the respective Houses, shall be created by the Chamber of Deputies and by the Federal Senate, jointly or separately, upon the request of one-third of its members, to investigate a given fact and for a certain period of time, and their conclusions shall,

if applicable, be forwarded to the Prosecution Office to determine the civil or criminal liability of the offenders.

Paragraph 4. During recess there shall be a committee to represent the National Congress, elected by both its Houses in the last regular session of the legislative session, with incumbencies defined in the common regulations, the composition of which shall repeat, to the extent possible, the proportional representation of the political parties.

SECTION VIII
The Legislative Process

SUBSECTION I
General Provision

Article 59. The legislative process comprises the preparation of:

I – Amendments to the Constitution;

II – supplementary laws;

III – ordinary laws;

IV – delegated laws;

V – provisional presidential decrees;

VI – legislative decrees;

VII – resolutions.

Single paragraph. A supplementary law shall provide for the preparation, drafting, amendment and consolidation of laws.

SUBSECTION II
Amendments to the Constitution

Article 60. The Constitution may be amended on the proposal of:

I – at least one-third of the members of the Chamber of Deputies or Federal Senate;

II – the president of the Republic;

III – more than one half of the State Legislatures of the federal entities, each of them expressing itself by a simple majority of its members.

Paragraph 1. The Constitution shall not be amended while federal intervention, state of defense or state of siege is in force.

Paragraph 2. The proposal shall be discussed and voted upon in each House of the National Congress, in two rounds, and it shall be considered approved if it obtains in both rounds, three-fifths of the votes of the respective members.

Paragraph 3. An Amendment to the Constitution shall be promulgated by the Directing Boards of the Chamber of Deputies and the Federal Senate with its respective sequence number.

Paragraph 4. No proposal of Constitutional Amendment shall be subject to deliberation if it has the tendency towards abolishing the:

I – federalist form of the State;

II – direct, secret, universal and periodic suffrage;

III – separation of powers;

IV – individual rights and guarantees.

Paragraph 5. The matter dealt with in a proposal of Amendment that is rejected or considered impaired shall not be the subject of another proposal in the same legislative session.

SUBSECTION III

The Laws

Article 61. The initiative of supplementary and ordinary laws is within the competence of any member or committee of the Chamber of Deputies or Federal Senate or National Congress, the president of the Republic, the Federal Supreme Court, the Superior Courts, the federal attorney general and citizens, in the manner and cases provided for in this Constitution.

Paragraph 1. The president of the Republic has the exclusive power to initiative the legislative proceeding to:

I – determine or modify the number of Armed Forces troops;

II – provide for:

a) creation of public offices, functions or positions in the direct bodies of the administration and in its associated entities or increases in their salaries;

b) administrative and judicial organization, tax and budget matters, public services and administrative personnel of the Territories;

c) civil servants of the Union and Territories, their legal statute, appointment to offices, tenure and retirement; (CA 18, 1998)

d) organization of the Prosecution Office and of the Office of the Public Defender of the Union, as well as general rules for the organization of the Prosecution Office and the Office of the Public Defender of the states, the Federal District and the Territories;

e) creation and extinction of Ministries and government bodies, with due regard for the provision of Article 84, item VI; (CA 32, 2001)

f) military of the Armed Forces, their legal statute, appointment to offices, promotions, tenure, remuneration, retirement, and transfer to the reserve. (CA 18, 1998)

Paragraph 2. The popular initiative proposal may be exercised by means of the presentation to the Chamber of Deputies of a bill of law subscribed by at least one percent of the national electorate, distributed throughout at least five states, with not less than three tenths of one percent of the voters in each of them.

Article 62. In important and urgent cases, the president of the Republic may adopt provisional decrees with the force of law and shall submit them to the National Congress immediately. (CA 32, 2001)

Paragraph 1. The issuance of provisional presidential decrees is forbidden when the matter involved: (CA 32, 2001)

I – deals with: (CA 32, 2001)

a) nationality, citizenship, political rights, political parties, and election law; (CA 32, 2001)

b) criminal law, criminal procedural law, and civil procedural law; (CA 32, 2001)

c) organization of the judiciary and Prosecution Office, the career and guarantees of their members; (CA 32, 2001)

d) pluriannual plans, budget directives, budgets, and additional and supplementary credits, with the exception of the provision mentioned in Article 167, paragraph 3; (CA 32, 2001)

II – aims at the detention or seizure of goods, people's savings, or any other financial asset; (CA 32, 2001)

III – is reserved for a supplementary law; (CA 32, 2001)

IV – has already been regulated by a bill of law passed by the National Congress that is awaiting sanction or veto by the president of the Republic. (CA 32, 2001)

Paragraph 2. A provisional presidential decree to institute or increase taxes, with the exception of the taxes mentioned in Articles 153, items I, II, IV, V, and 154, item II, shall only produce effects in the subsequent fiscal year if it has been converted into law before or on the last day of the fiscal year in which it was issued. (CA 32, 2001)

Paragraph 3. With the exception of the provisions mentioned in paragraphs 11 and 12, provisional presidential decrees shall lose effectiveness from the day of their issuance if they are not converted into law within a period of sixty days, which may be extended once

for an identical period under the terms of paragraph 7, and the National Congress shall issue a legislative decree to regulate the legal relations arising therefrom. (CA 32, 2001)

Paragraph 4. The period mentioned in paragraph 3 shall be counted from the date of publication of the provisional presidential decree and shall be suspended while the National Congress is in recess. (CA 32, 2001)

Paragraph 5. Deliberation by each House of the National Congress upon the merits of provisional presidential decrees shall depend on prior determination of their compliance with the constitutional requirements. (CA 32, 2001)

Paragraph 6. If a provisional presidential decree is not examined within forty-five days as of its date of publication, it shall subsequently be forwarded to urgent consideration in each House of the National Congress, and the deliberation of all other legislative matters shall be suspended in the House where it is under consideration, until voting is concluded. (CA 32, 2001)

Paragraph 7. If the voting of a provisional presidential decree is not concluded in both Houses of the National Congress within sixty days as of its date of publication, its period of effectiveness may be extended once for an identical period. (CA 32, 2001)

Paragraph 8. The voting of a provisional presidential decree shall start in the Chamber of Deputies. (CA 32, 2001)

Paragraph 9. It is incumbent upon the joint committee of deputies and senators to examine a provisional presidential decree and issue an opinion thereon, before they are submitted to the floor in each House of the National Congress in a separate session. (CA 32, 2001)

Paragraph 10. It is forbidden to reissue a provisional presidential decree in the same legislative session in which it has been rejected or that has lost its effectiveness due to a lapse of time. (CA 32, 2001)

Paragraph 11. If the legislative decree mentioned in paragraph 3 is not issued within sixty days as of the date the provisional presidential decree was rejected or lost its effectiveness, the legal relations constituted and arising from acts performed during its period of effectiveness shall still be regulated by such provisional decree. (CA 32, 2001)

Paragraph 12. Should a bill of law be passed that alters the original text of a provisional presidential decree, the latter will remain effective in full until such date as the bill is sanctioned or vetoed. (CA 32, 2001)

Article 63. An increase in expenditure proposals shall not be admitted:

I – in bills of the exclusive initiative of the president of the Republic, except for the provisions of Article 166, paragraphs 3 and 4;

II – in bills concerning the organization of the administrative services of the Chamber of Deputies, Federal Senate, Federal Courts and Prosecution Office.

Article 64. The discussion and voting of the bills of law that are the initiative of the president of the Republic, Federal Supreme Court and Superior Courts shall start in the Chamber of Deputies.

Paragraph 1. The president of the Republic may request urgency in the examination of bills of their own initiative.

Paragraph 2. If, in the event of paragraph 1, the Chamber of Deputies and the Federal Senate fail to act, each one, successively, on the proposition, within the period of forty-five days, deliberation on all other legislative matters shall be suspended in the respective House, except for those which must be considered within a stipulated constitutional period, in order that the voting may be concluded. (CA 32, 2001)

Paragraph 3. Amendments of the Federal Senate shall be examined by the Chamber of Deputies within a period of ten days, in accordance with the provisions of the preceding paragraph.

Paragraph 4. The periods of time referred to in paragraph 2 shall not be counted while the Congress is in recess and shall not apply to the legal drafting of codes.

Article 65. A bill of law approved by one House shall be reviewed by the other in a single round of discussion and voting and sent for sanctioning or promulgation, if approved by the reviewing House, or it shall be dismissed, if rejected.

Single paragraph. If the bill is amended, it shall return to the House where it was proposed.

Article 66. The House in which voting is concluded shall send the bill of law to the president of the Republic, who, upon concurring, shall sanction it.

Paragraph 1. If the president of the Republic considers the bill of law, wholly or in part, unconstitutional or contrary to public interest, the president shall veto it, wholly or in part, within fifteen work days, counted from the date of receipt and he shall, within forty-eight hours, inform the president of the Senate of the reasons of his veto.

Paragraph 2. A partial veto shall only comprise the full text of an Article, paragraph, item or subitem.

Paragraph 3. After a period of fifteen days, the lack of decision by the president of the Republic shall be considered as sanctioning.

Paragraph 4. The veto shall be examined in a joint session, within thirty days, counted from the date of receipt, and may only be rejected by an absolute majority of the deputies and senators. (CA 76, 2013)

Paragraph 5. If the veto is not upheld, the bill shall be sent to the president of the Republic for promulgation.

Paragraph 6. If the period established in paragraph 4 elapses without a decision being reached, the veto shall be included in the order of the day of the subsequent session, and all other propositions shall be suspended until its final voting. (CA 32, 2001)

Paragraph 7. If, in the cases of paragraphs 3 and 5, the law is not promulgated within forty-eight hours by the president of the Republic, the president of the Senate shall enact it and if the latter fails to do so within the same period, the vice-president of the Senate shall do so.

Article 67. The matter dealt with in a rejected bill of law may only be the subject of a new bill during the same legislative session, upon proposal of an absolute majority of the members of either House of the National Congress.

Article 68. Delegated laws shall be drawn up by the president of the Republic, who shall request delegation from the National Congress.

Paragraph 1. There shall be no delegation of acts falling within the exclusive power of the National Congress, of those within the exclusive power of the Chamber of Deputies or Federal Senate, of matters reserved for supplementary laws and of legislation on:

I – the organization of the judicial branch and of the Prosecution Office, the career and guarantees of their members;

II – nationality, citizenship, individual, political and electoral rights;

III – pluriannual plans, budget directives and budgets.

Paragraph 2. The delegation to the president of the Republic shall take the form of a resolution of the National Congress, which shall specify its contents and the terms of its exercise.

Paragraph 3. If the resolution calls for consideration of the bill by the National Congress, the latter shall do so in a single voting, any amendment being forbidden.

Article 69. Supplementary laws shall be approved by absolute majority.

SECTION IX

Accounting, Financial and Budget Control

Article 70. Control of accounts, finances, budget, operations and property of the Union and of the government bodies and associated entities, as to lawfulness, legitimacy, economic efficiency, application of subsidies and waiver of revenues, shall be exercised by

the National Congress, by means of external control and of the internal control system of each branch.

Single paragraph. Accounts shall be rendered by any individual or corporation, public or private, which uses, collects, keeps, manages, or administers public resources, assets or values, or those for which the Union is responsible or which, on behalf of the Union, assumes obligations of a pecuniary nature. (CA 19, 1998)

Article 71. External control, incumbent on the National Congress, shall be exercised with the aid of the Federal Accounting Court, which shall:

I – examine the accounts rendered annually by the president of the Republic, by means of a prior report which shall be prepared in sixty days counted from receipt;

II – evaluate the accounts of the administrators and other people responsible for public resources, assets and values of the administration direct bodies and its associated entities, including foundations and companies instituted and maintained by the Union as well as the accounts of those who have caused a loss, misplacement or other irregularity resulting in losses to the Treasury;

III – examine, for the purpose of registration, the lawfulness of acts of admission of personnel, on any account, in the administration direct bodies and its associated entities, including the foundations instituted and maintained by the Union, with the exception of the appointments to commission offices, as well as the granting of civil and military retirement and pensions, except for subsequent improvements which do not alter the legal fundaments of the conceding act;

IV – carry out, on its own initiative or on that of the Chamber of Deputies, of the Federal Senate, or of a technical or inquiry committee, inspection and audits of an accounting, financial, budget, operational or property nature in the administrative units of the legislative, executive and judicial branches and other entities referred to in item II;

V – control the national accounts of supranational companies in whose capital stock the Union holds a direct or indirect interest, as set forth in the acts of incorporation;

VI – control the use of any funds transferred by the Union, by means of an agreement, arrangement, adjustment or any other similar instrument, to a state, the Federal District or a municipality;

VII – render the information requested by the National Congress, by either of its Houses or by any of the respective committees concerning accounting, financial, budget, operational and property control and the results of audits and inspections made;

VIII – in case of illegal expenses or irregular accounts, apply to the responsible parties the penalties provided by law, which shall establish, among other penalties, a fine proportional to the damage caused to the Treasury;

IX – determine a period for the body or entity to take the necessary steps for the strict compliance with the law, if an illegality is established;

X – if not heeded, suspend the enforcement of the act challenged, notifying the Chamber of Deputies and the Federal Senate of such decision;

XI – present a formal charge to the competent branch on any irregularities or abuses verified.

Paragraph 1. In the case of a contract, the restraining act shall be adopted directly by the National Congress, which shall immediately request the executive branch to take the applicable measures.

Paragraph 2. If the National Congress or the executive branch, within ninety days, do not take the measures provided for in the preceding paragraph, the Court shall decide on the matter.

Paragraph 3. Decisions of the Court resulting in the imposition of a debt or fine shall have the effectiveness of an enforceable instrument.

Paragraph 4. The Court shall, quarterly and annually, forward a report on its activities to the National Congress.

Article 72. In view of indications of unauthorized expenditure, even if in the form of non-programmed investments or non-approved subsidies, the permanent joint committee referred to in Article 166, paragraph 1, may request the incumbent government authority to render the necessary explanation, within five days.

Paragraph 1. If the explanations are not rendered or are considered insufficient, the committee shall request the Court to make a conclusive statement on the matter within thirty days.

Paragraph 2. If the Court deems the expense to be irregular, the committee shall, if it considers that the expenditure may cause irreparable damage or serious injury to the public economy, propose to the National Congress that it be suspended.

Article 73. The Federal Accounting Court, formed by nine justices, shall have its seat in the Federal District, its own staff and jurisdiction throughout the national territory, and shall exercise, insofar as pertinent, the incumbencies provided for in Article 96.

Paragraph 1. The justices of the Federal Accounting Court shall be appointed from among Brazilians who meet the following requirements:

I – more than thirty-five and less than sixty-five years of age;

II – moral integrity and the highest moral reputation;

III – renowned knowledge in legal matters, accounting, economics and finances or of public administration;

IV – more than ten years of exercise of office or of actual professional activity which requires the knowledge mentioned in the preceding item.

Paragraph 2. The justices of the Federal Accounting Court shall be chosen:

I – one third by the president of the Republic with the approval of the Federal Senate, two of them being alternately chosen from among auditors and members of the Accounting Court Prosecution Office, as indicated in a triple list by the Court, in accordance with criteria of seniority and merit;

II – two-thirds by the National Congress.

Paragraph 3. The justices of the Federal Accounting Court shall have the same guarantees, prerogatives, impediments, remuneration, and benefits as the justices of the Superior Court of Justice, their retirement benefits and other pensions being ruled by the provisions of Article 40. (CA 20, 1998)

Paragraph 4. The auditor, when substituting for a justice, shall have the same guarantees and impediments as the incumbent justice, and, when performing the other duties of the judicature, those of a judge of a Federal Regional Court.

Article 74. The legislative, executive and judicial branches shall maintain an integrated system of internal control for the purpose of:

I – evaluating the attainment of the goals established in the pluriannual plan, the implementation of government programs and of the budgets of the Union;

II – verifying the lawfulness and evaluating the results, as to effectiveness and efficiency, of the budget, financial and property management in the government bodies and entities of the federal administration, as well as the use of public funds by private legal entities;

III – exercising control over credit transactions, collateral signatures and guarantees, as well as over the rights and assets of the Union;

IV – supporting external control in the exercise of its institutional mission.

Paragraph 1. People in charge of internal control shall, upon learning of any irregularity or illegality, inform the Federal Accounting Court about it, subject to joint and several liability.

Paragraph 2. Any citizen, political party, association or labor union has standing under the law to denounce irregularities or illegalities to the Federal Accounting Court.

Article 75. The rules set forth in this section shall apply, where appropriate, to the organization, composition and control of the Accounting Courts of the states and of the Federal District, as well as the municipal Accounting Courts and Councils.

Single paragraph. The state Constitutions shall provide for the respective Accounting Courts, which shall be formed by seven counselors.

CHAPTER II
The Executive Branch

SECTION I
The President and the Vice-President of the Republic

Article 76. The executive power is vested in the president of the Republic, who is assisted by the ministers of State.

Article 77. The election of the president and vice-president of the Republic shall take place simultaneously, on the first Sunday of October, in the first round, and on the last Sunday of October, in the second round, as the case may be, of the year preceding the one in which the current presidential term of office ends. (CA 16, 1997)

Paragraph 1. The election of the president of the Republic shall imply the election of the vice-president registered with him.

Paragraph 2. The candidate who, being registered by a political party, obtains an absolute majority of votes, not counting blank or void votes, shall be considered elected president.

Paragraph 3. If no candidate attains an absolute majority in the first voting, another election shall be held within twenty days from the announcement of the results, the competition being between the two candidates with the highest number of votes, and being considered elected the candidate with the majority of valid votes.

Paragraph 4. If, before the runoff election is held, a candidate dies, withdraws or is legally impaired, the candidate with the greatest number of votes among the remaining candidates shall be called.

Paragraph 5. If in the event of the preceding paragraphs, more than one candidate with an equal number of votes remain in second place, the senior one shall qualify.

Article 78. The president and the vice-president of the Republic shall take office in a session of the National Congress, pledging to maintain, defend and comply with the

Constitution, obey the laws, promote the general well-being of the Brazilian people, sustain the union, the integrity and the independence of Brazil.

Single paragraph. If, after ten days from the date scheduled for the inauguration, the president or the vice-president, except by reason of *force majeure*, has not taken office, the office shall be declared vacant.

Article 79. The vice-president shall replace the president in the event of impediment and shall succeed him in the event of vacancy.

Single paragraph. In addition to other duties attributed to him by a supplementary law, the vice-president shall assist the president whenever summoned by him for special missions.

Article 80. In the event of impediment of the president and vice-president, or of vacancy of the respective offices, the president of the Chamber of Deputies, the president of the Senate and the chief justice of the Federal Supreme Court shall be called successively to exercise the Presidency.

Article 81. In the event of vacancy of the offices of president and vice-president of the Republic, elections shall be held ninety days after the occurrence of the last vacancy.

Paragraph 1. If the vacancy occurs during the last two years of the president's term of office, the National Congress shall hold elections for both offices thirty days after the last vacancy, as established by law.

Paragraph 2. In any of the cases, those elected shall complete the term of office of their predecessors.

Article 82. The president of the Republic has a four-year term of office, and it shall commence on January 1 following the year of his election. (CA 16, 1997)

Article 83. The president and the vice-president of the Republic may not, without authorization from the National Congress, leave the country for a period of more than fifteen days, subject to loss of office.

SECTION II

Duties of the President of the Republic

Article 84. The president of the Republic shall have the exclusive power to:

I – appoint and dismiss the ministers of State;

II – exercise, with the assistance of the ministers of State, the higher management of the federal administration;

III – start the legislative procedure, in the manner and in the cases set forth in this Constitution;

IV – sanction, promulgate and order the publication of laws, as well as issue decrees and regulations for the true enforcement thereof;

V – veto bills, wholly or in part;

VI – provide for the following, by means of a decree: (CA 32, 2001)

a) organization and operation of the federal administration, whenever no increase of expenditures or creation or abolishment of government bodies is involved; (CA 32, 2001)

b) elimination of public positions or posts, if vacant; (CA 32, 2001)

VII – maintain relations with foreign States and accredit their diplomatic representatives;

VIII – conclude international treaties, conventions and acts, *ad referendum* of the National Congress;

IX – decree the state of defense and the state of siege;

X – decree and enforce federal intervention;

XI – upon the opening of the legislative session, send a government message and plan to the National Congress, describing the state of the nation and requesting any action deemed necessary;

XII – grant pardons and reduce sentences, after hearing the entities instituted by law, if necessary;

XIII – exercise the supreme command of the Armed Forces, appoint the commanders of the Navy, Army, and Air Force, promote general officers and appoint them to the offices held exclusively by them; (CA 23, 1999)

XIV – appoint, after approval by the Senate, the justices of the Federal Supreme Court and those of the Superior Courts, the governors of the Territories, the federal attorney general, the president and the directors of the Central Bank and other civil servants, when established by law;

XV – appoint, with due regard for the provisions of Article 73, the justices of the Federal Accounting Court;

XVI – appoint judges in the events established by this Constitution and the general counsel to the federal government;

XVII – appoint members of the Council of the Republic, in accordance with Article 89, item VII;

XVIII – call and preside over the Council of the Republic and the National Defense Council;

XIX – declare war, in the event of foreign aggression, authorized by the National Congress or confirmed by it, whenever it occurs between legislative sessions and, under the same conditions, to decree full or partial national mobilization;

XX – make peace, authorized or confirmed by the National Congress;

XXI – award decorations and honorary distinctions;

XXII – permit, in the cases set forth by supplementary law, foreign forces to pass through the national territory, or remain temporarily therein;

XXIII – submit to the National Congress the multiyear plan, the bill of budget directives and the budget proposals set forth in this Constitution;

XXIV – render, each year, accounts to the National Congress concerning the previous fiscal year, within sixty days of the opening of the legislative session;

XXV – fill and abolish federal government positions, as set forth by law;

XXVI – issue presidential provisional decrees, with force of law, according to Article 62;

XXVII – perform other duties set forth in this Constitution.

Single paragraph. The president of the Republic may delegate the duties mentioned in items VI, XII and XXV, first part, to the ministers of State, to the federal attorney general or to the general counsel to the federal government, who shall observe the limits established in the respective delegations.

SECTION III

Liability of the President of the Republic

Article 85. The acts of the president of the Republic that attempt against the Federal Constitution are crimes of responsibility, especially those against the:

I – existence of the Union;

II – free exercise of the powers of the legislative and judicial branch, of the Prosecution Office and the constitutional powers of the federal entities;

III – exercise of political, individual and social rights;

IV – internal security of the country;

V – probity in the administration;

VI – budget law;

VII – compliance with the laws and court decisions.

Single paragraph. These crimes shall be defined in a special law, which shall establish the rules of procedure and trial.

Article 86. If charges against the president of the Republic are accepted by two thirds of the Chamber of Deputies, he shall be submitted to trial before the Federal Supreme Court for common criminal offenses or before the Federal Senate for crimes of responsibility.

Paragraph 1. The president shall be suspended from office:

I – in common criminal offenses, if the Federal Supreme Court accepts the information or the complaint;

II – in the event of crimes of responsibility, after the Senate commences the proceeding.

Paragraph 2. If the trial has not been concluded after a period of one hundred and eighty days, the president shall return to office notwithstanding the regular progress of the proceeding.

Paragraph 3. In the event of common offenses, the president of the Republic shall not be subject to arrest before a criminal conviction is issued.

Paragraph 4. During his term of office, the president of the Republic may not be held liable for acts not related to the performance of the functions of the public office.

SECTION IV

The Ministers of State

Article 87. The ministers of State shall be chosen from among Brazilians over twenty-one years of age and in possession of their political rights.

Single paragraph. The minister of State, in addition to other duties established in this Constitution and in the law, has the power to:

I – exercise guidance, coordination and supervision of the agencies and entities of the federal administration in the area of their authority and endorse acts and decrees signed by the president of the Republic;

II – issue instructions for the enforcement of laws, decrees and regulations;

III – submit to the president of the Republic an annual report on the administration of the Ministry;

IV – perform the acts pertinent to the duties assigned or delegated by the president of the Republic.

Article 88. The law shall provide for the creation and extinction of ministries and government bodies. (CA 32, 2001)

SECTION V
The Council of the Republic and the National Defense Council

SUBSECTION I
The Council of the Republic

Article 89. The Council of the Republic is a higher body for consultation by the president of the Republic, and its members are the:

I – vice-president of the Republic;

II – president of the Chamber of Deputies;

III – president of the Federal Senate;

IV – majority and minority leaders in the Chamber of Deputies;

V – majority and minority leaders in the Federal Senate;

VI – minister of justice;

VII – six born Brazilian citizens, with over thirty-five years of age, two of which appointed by the president of the Republic, two elected by the Federal Senate and two elected by the Chamber of Deputies, all with a three-year term of office, the reappointment being prohibited.

Article 90. The Council of the Republic has the power to express opinion on:

I – federal intervention, state of defense and state of siege;

II – matters relevant to the stability of the democratic institutions.

Paragraph 1. The president of the Republic may summon a minister of State to participate in the Council meeting, when the agenda includes a matter related to the respective Ministry.

Paragraph 2. The organization and operation of the Council of the Republic shall be regulated by law.

SUBSECTION II
The National Defense Council

Article 91. The National Defense Council is a consultation body of the president of the Republic on matters related to national sovereignty and defense of the democratic State ruled by the law, and the following participate in it as natural members the:

I – vice-president of the Republic;

II – president of the Chamber of Deputies;

III – president of the Federal Senate;

IV – minister of justice;

V – minister of defense; (CA 23, 1999)

VI – foreign minister;

VII – minister of planning;

VIII – the commanders of the Navy, the Army, and the Air Force. (CA 23, 1999)

Paragraph 1. It is the competence of the National Defense Council:

I – to express opinion in the event of declaration of war and making peace, as established in this Constitution;

II – to express opinion on the decreeing of state of defense, state of siege and federal intervention;

III – to propose the criteria and conditions for the use of areas which are indispensable to the security of the national territory and to express opinion on their actual use, especially on the frontier zone and on those related to the preservation and exploitation of natural resources of any kind;

IV – to study, propose and monitor the development of initiatives required to guarantee national independence and the defense of the democratic State.

Paragraph 2. The organization and the operation of the National Defense Council shall be regulated by law.

CHAPTER III
The Judicial Branch

SECTION I
General Provisions

Article 92. The judicial branch consists of the following bodies:

I – the Federal Supreme Court;

I-A – the National Council of Justice; (CA 45, 2004)

II – the Superior Court of Justice;

II-A – the Superior Labor Court; (CA 92, 2016)

III – the Regional Federal Courts of Appeals and federal judges;

IV – the Labor Courts of Appeals and labor judges;

V – the Electoral Courts and judges;

VI – the Military Courts and judges;

VII – the Courts of Appeals and judges of the states and of the Federal District and Territories.

Paragraph 1. The Federal Supreme Court, the National Council of Justice, and the Superior Courts have their seat in the Federal Capital. (CA 45, 2004)

Paragraph 2. The Federal Supreme Court and the Superior Courts have jurisdiction over the entire Brazilian territory. (CA 45, 2004)

Article 93. A supplementary law, proposed by the Federal Supreme Court, shall provide for the statute of the judicature, observing the following principles:

I – admission into the career, with the initial post of substitute judge, by means of a competitive civil-service entrance examination of tests and presentation of academic and professional credentials, with the participation of the Brazilian Bar Association in all phases, at least three years of legal practice being required of holders of a B.A. in law, and obeying the order of classification for appointments; (CA 45, 2004)

II – promotion from level to level, based on seniority and merit, alternately, observing the following rules:

a) mandatory promotion of judges who have appeared in a merit list for three consecutive times or for five alternate times;

b) merit promotion requires two years in office in the respective level and that the judge appears in the top fifth part of the seniority list of such level, unless no one satisfying such requirements is willing to accept the vacant post;

c) evaluation of merit according to performance and to the objective criteria of productivity and promptness in exercising jurisdiction and according to attendance and achievement in official or recognized professional development courses; (CA 45, 2004)

d) in determining seniority, the court may only reject the judge with the longest service by the justified vote of two-thirds of its members, according to a specific procedure, full defense being ensured, the voting being repeated until the selection is concluded; (CA 45, 2004)

e) promotion shall not be granted to judges who unjustifiably withhold case records beyond the legal deadline, they may not return them to the court archives without providing the necessary disposition thereof or decision thereon; (CA 45, 2004)

III – access to the courts of second instance shall obey seniority and merit, alternately, as determined at the last or single level; (CA 45, 2004)

IV – provision of official courses for preparation, professional development, and promotion of judges, while the participation in an official course or in a course recognized by a national school for the education and further development of judges, shall constitute a mandatory stage of the tenure acquisition process; (CA 45, 2004)

V – the compensation of the justices of the Superior Courts shall correspond to ninety-five percent of the monthly compensation stipulated for the justices of the Federal Supreme Court, and the compensation of the other judges shall be stipulated by law and distributed, at federal and state levels, according to the respective categories of the national structure of the judiciary, and the difference between categories may not be higher than ten percent or lower than five percent, nor higher than ninety-five percent of the monthly compensation of the justices of the Superior Courts, with due regard, in any of the cases, for the provisions of Articles 37, item XI, and 39, paragraph 4; (CA 19, 1998)

VI – the retirement of judges as well as the granting of pensions for their dependents shall comply with the provisions of Article 40; (CA 20, 1998)

VII – a permanent judge shall reside in the respective judicial district, except when otherwise authorized by the court; (CA 45, 2004)

VIII – the acts of removal and of placement on paid availability of a judge, for public interest, shall be based on a decision by the vote of an absolute majority of the respective court or of the National Council of Justice; the right of full defense is ensured; (CA 103, 2019)

VIII-A – the removal upon request or the exchange of judges of same-level judicial districts shall obey, insofar as pertinent, the provisions of subitems *a*, *b*, *c*, and *e* of item II; (CA 45, 2004)

IX – all judgements of the bodies of the judicial branch shall be public, and all decisions shall present grounds, under penalty of nullity, but the law may limit attendance, in given acts, to the interested parties and to their lawyers, or only to the latter, whenever preservation of the right to privacy of the party interested in confidentiality will not harm the right of the public interest to information; (CA 45, 2004)

X – courts shall present grounds in the administrative decisions, which shall be entered in public sessions; the disciplinary trials shall be taken by vote of an absolute majority of its members; (CA 45, 2004)

XI – in courts with more than twenty-five judges, a special body may be constituted, with a minimum of eleven and a maximum of twenty-five members, to exercise delegated administrative and jurisdictional duties which are under the powers of the full court, half of the positions being filled according to seniority and the other half through election by the full court; (CA 45, 2004)

XII – courts will operate continuously, without interruption, collective vacation being forbidden for first instance judges and courts of second instance, and there must be judges on duty at all times on days in which courts are closed; (CA 45, 2004)

XIII – the number of judges in each court shall be proportional to the effective judicial demand and to the respective population; (CA 45, 2004)

XIV – court employees will receive delegation to carry out administrative acts and acts aimed at the mere disposition of matters, without a decisional nature; (CA 45, 2004)

XV – proceedings will be assigned immediately upon filing, at all levels of jurisdiction. (CA 45, 2004)

Article 94. One fifth of the seats of the Regional Federal Courts of Appeal, of the Courts of Appeal of the states, and of the Court of Appeal of the Federal District and the Territories shall be occupied by members of the Prosecution Office, with over ten years of office, and by lawyers of renowned legal knowledge and the highest moral reputation, with over ten years of effective professional activity, nominated in a list of six names by the entities representing the respective classes.

Single paragraph. Upon receiving the nominations, the court shall organize a list of three names and shall send it to the executive branch, which shall, within the subsequent twenty days, select one of the listed names for appointment.

Article 95. Judges enjoy the following guarantees:

I – life-tenure office, which, at first instance, shall only be acquired after two years in office, loss of office being dependent, during this period, on deliberation of the court to which the judge is subject, and, in other cases, on a final and unappealable judicial decision;

II –non-removability, except for reason of public interest, under the terms of Article 93, item VIII;

III – irreducibility of compensation, except for the provisions of Articles 37, items X and XI, 39, paragraph 4, 150, item II, 153, item III, and 153, paragraph 2, item I. (CA 19, 1998)

Single paragraph. Judges are forbidden to:

I – hold, even when on paid availability, another office or position, except for a teaching position;

II – receive, on any account or for any reason, court costs or participation in a lawsuit;

III – engage in political or party activities;

IV – receive, on any account or for any reason, financial aid or contribution from individuals, and from public or private institutions, except for the exceptions set forth in law; (CA 45, 2004)

V – practice law in the court on which they served as judges, for a period of three years following their retirement or discharge. (CA 45, 2004)

Article 96. It is of the exclusive competence of:

I – the courts:

a) to elect their directive bodies and to draw up their internal regulations, in compliance with the rules of proceedings and the procedural guarantees of the parties, providing the competence and the operation of the respective jurisdictional and administrative bodies;

b) to organize their secretariats and auxiliary services, as well as those of the courts connected with them, guaranteeing the exercise of the respective inspection activities;

c) to fill, as provided by this Constitution, offices of career judges within their respective jurisdiction;

d) to propose the creation of new courts of first instance;

e) to fill, by means of a competitive civil-service examination of tests, or of tests and presentation of academic and professional credentials, according to the provisions of Article 169, single paragraph, the offices required for the administration of justice, except for the positions of trust as defined in law;

f) to grant leave, vacations and other authorized absences to their members and to the judges and employees who are immediately subordinated to them;

II – the Federal Supreme Court, the Superior Courts and the Courts of Appeal, to propose to the respective legislative branch, with due regard for the provisions of Article 169:

a) alteration in the number of members of the lower courts;

b) creation and elimination of offices and the remuneration of the auxiliary services and of the courts connected with them, as well as the establishment of the compensation for their members and for the judges, including those of the lower courts, if existing; (CA 41, 2003)

c) creation or extinction of lower courts;

d) alteration of the judicial organization and division;

III – the Courts of Appeals, to try judges of the states, Federal District and Territories, as well as members of the Prosecution Office, for common crimes and crimes of responsibility, except in the cases within the jurisdiction of the Electoral Courts.

Article 97. Courts may declare a law or a normative act of the government unconstitutional only by the vote of an absolute majority of their members or of the members of the respective special body.

Article 98. The Union shall create in the Federal District and in the Territories, and the states shall create within their limits:

I – special courts, filled by professional judges, or by professional and lay judges, with power for conciliation, judgment and execution of civil suits of lesser complexity and criminal offenses of lower offensive potential, by oral and summary proceedings, allowing, in the cases established in law, the settlement and judgment of appeals by panels of judges of first instance;

II – remunerated justice of the peace, formed by citizens elected by direct, universal and secret vote, with a four-year term of office and competence to, as provided by law, perform marriages, examine qualification proceedings, on their own initiative or in view of the presentation of a challenge, and exercise conciliatory functions, of a non-jurisdictional nature, besides others established by law.

Paragraph 1. Federal legislation shall provide for the establishment of special courts within the Federal Justice. (CA 45, 2004)

Paragraph 2. Judicial costs and fees shall be assigned exclusively to fund services related to activities which are specific of the judiciary. (CA 45, 2004)

Article 99. The judicial branch is ensured of administrative and financial autonomy.

Paragraph 1. Courts shall prepare their budget proposals, within the limits stipulated jointly with the other branches in the budget directives law.

Paragraph 2. The proposal shall, after hearing the other interested courts, be forwarded:

I – at the federal level, by the chief justices of the Federal Supreme Court and of the Superior Courts, with the approval of the respective courts;

II – at the level of the states and of the Federal District and the Territories, by the chief appellate judges of the Courts of Appeal, with the approval of the respective courts.

Paragraph 3. If the government bodies referred to in paragraph 2 do not forward their respective budget proposals within the time period stipulated in the budget directives law, the executive branch shall, with a view to engrossing the annual budget proposal, take into account the figures approved in the current budget law, adjusted in accordance with the limits stipulated according to paragraph 1 of this Article. (CA 45, 2004)

Paragraph 4. If the budget proposals referred to in this Article and thus forwarded do not obey the limits stipulated under paragraph 1, the executive branch shall make the necessary adjustments to consolidate the annual budget proposal. (CA 45, 2004)

Paragraph 5. In the implementation of the budget of a specific fiscal year, no expenses may be incurred, and no obligations may be assumed that exceed the limits stipulated in

the budget directives law, except when previously authorized, by opening supplementary or special credits. (CA 45, 2004)

Article 100. Payments owed by the federal, state, Federal District, or municipal Treasuries, as a result of a court decision, shall be made exclusively in chronological order of submission of court orders and charged to the respective credits, it being forbidden to designate cases or people in the budget appropriations and in the additional credits opened for such purpose. (CA 62, 2009)

Paragraph 1. Support-related debts include those arising from wages, salaries, pay, pensions, and their supplementations, social security benefits and compensation for death and disability, such compensation being based on civil liability, as a result of a final and unappealable judicial decision, and shall be paid before any other debts, except those referred to in paragraph 2 of this Article. (CA 62, 2009)

Paragraph 2. Support-related debts whose owners, either original or through inheritance, are 60 (sixty) years of age, or suffer from a serious disease, or have a disability, as defined by law, shall be paid with preference over all other debts, up to an amount equivalent to three times the one established by law for the purposes of the provision in paragraph 3 of this Article. Payment of a fractional amount is permitted for such end, whereas the remaining shall be paid in chronological order of submission of the respective court order. (CA 94, 2016)

Paragraph 3. The provision contained in the head provision, regarding the issuance of court orders, does not apply to obligations defined by law as small amounts, which must be paid by the treasuries herein referred to as a result of a final and unappealable court decision. (CA 62, 2009)

Paragraph 4. For the purposes of the provision of paragraph 3, different amounts may be stipulated for the federal entities through their own legislation and according to their various economic capabilities, whereas the minimum amount shall be equal to the amount of the highest benefit paid by the general social security regime. (CA 62, 2009)

Paragraph 5. It is mandatory for the budgets of the federal entities to include the funds required for payment of debts arising from final and unappealable judicial decisions, stated in court orders submitted by July 1st, and payment shall be made before the end of the subsequent fiscal year, on which date their amounts shall be adjusted for inflation. (CA 62, 2009)

Paragraph 6. The budget allocations and the credits opened shall be assigned to the Judicial branch, it being within the competence of the presiding judge of the court which rendered the decision of execution to determine full payment and to authorize upon peti-

tion of a creditor and exclusively in the event that his right of precedence is not respected or that the amount necessary to satisfy the debt has not been set aside attachment of the respective amount. (CA 62, 2009)

Paragraph 7. The presiding judge of the competent court who, by means of an act or omission, delays or attempts to frustrate the regular payment of a court-ordered debt shall be liable to crime of responsibility and shall also appear before the National Council of Justice. (CA 62, 2009)

Paragraph 8. The issuance of a court order as a supplementation to or in addition to an amount already paid, as well as the parceling, apportionment, or reduction of the amount being enforced aiming at placing a share of the entire debt under paragraph 3 of this article is forbidden. (CA 62, 2009)

Paragraph 9. At the time a court order is issued, irrespective of the relevant regulation, there shall be deducted from such court order, for the purpose of a setoff, an amount corresponding to clear legal debts, either registered or not under debts in execution and attributed to the original creditor by the Treasury in debt, including future accruing instalments of parceling, except for those whose execution has been suspended as a result of administrative or judicial challenge. (CA 62, 2009)

Paragraph 10. Before a court order is issued, the relevant court shall request that the Treasury in debt must provide, within 30 (thirty) days, otherwise subject to loss of the right to offset, information on the debits which meet the conditions stipulated in paragraph 9, for the purposes set forth in said paragraph. (CA 62, 2009)

Paragraph 11. In accordance with legislation of the federal entity in debt, a creditor may employ court order credits to purchase public property belonging to the respective federal entity. (CA 62, 2009)

Paragraph 12. As from the date the Constitutional Amendment 62 is enacted, the amounts stated in court orders, after such court orders are issued up until effective payment, irrespective of their nature, shall be adjusted according to the official rate applied to savings accounts, whereas, for the purpose of compensation of delay in the payment, simple interest will be applied at the same percentage of interest applied to savings accounts, being excluded the employment of compensatory interest. (CA 62, 2009)

Paragraph 13. Creditors may assign their court order credits, in whole or in part, to third parties, irrespective of consent by the debtor, and the provisions of paragraphs 2 and 3 shall not be applied to the assignee. (CA 62, 2009)

Paragraph 14. Assignment of court order credits shall produce effects only after communication with the court of origin and to the federal entity in debt by filing a relevant petition. (CA 62, 2009)

Paragraph 15. Without prejudice to the provisions of this Article, a supplementary law to this Federal Constitution may establish a special regime for payment of court-ordered debts owed by states, the Federal District, and municipalities, providing for earmarked net current revenues and for payment term and methods. (CA 62, 2009)

Paragraph 16. The Union may, at its own discretion and as provided by law, take on debts resulting from court orders issued against a state, the Federal District, or a Municipality, and refinance them directly. (CA 62, 2009)

Paragraph 17. The Union, states, Federal District and municipalities shall compare monthly, on an annual basis, their respective net current revenue committed to pay court-ordered debts and small amount obligations. (CA 94, 2016)

Paragraph 18. For the purposes of paragraph 17, net current revenue means the sum of revenues from taxes, assets, industry, agriculture, and livestock; from contributions and services; from current transfers and other current revenues, including those from paragraph 1 of Article 20 of the Federal Constitution, assessed in the period including the second month immediately prior to the referred month and the 11 (eleven) preceding months, excluding duplicities and deductions: (CA 94, 2016)

I – from the Union, the amount transferred to the states, Federal District, and municipalities by constitutional requirement; (CA 94, 2016)

II – from the states, the amount transferred to the municipalities by constitutional requirement; (CA 94, 2016)

III – from the Union, states, Federal District and municipalities, the employees' contribution for the cost of their system of social security and social assistance and the receipts from the financial compensation referred to in paragraph 9 of Article 201 of the Federal Constitution. (CA 94, 2016)

Paragraph 19. In the event that the total amount of court-ordered debts and of small amount obligations in a 12 (twelve) month period exceeds the average of the committed of percentage of current net receipts percentage in the immediately preceding 5 (five) years, the amount that exceeds this percentage may be financed, exempted from the debt limits dealt with in items VI and VII of Article 52 of the Federal Constitution and from any other debt limitations provided for by law. The prohibition to earmark tax revenues under Article 167, item IV, of the Federal Constitution shall not apply to such funding mechanism. (CA 94, 2016)

Paragraph 20. In the event of a court-ordered debt amounting over 15% (fifteen percent) of the court-ordered debts value submitted under paragraph 5 of this Article, 15% (fifteen percent) of the value of such court-ordered debt shall be paid by the end of the following fiscal year and the remaining in equal installments in the five subsequent fiscal years, increased by interest for the delay and inflation adjustment, or through direct settlements, before Auxiliaries Courts for Settlement of Judgement Debt of the government, reducing 40% (forty percent) of the updated credit value at most, so long as no appeal or judicial defense is pending with respect to the credit and that the requirements set forth in the regulations issued by the federal entity are observed. (CA 94, 2016)

SECTION II

The Federal Supreme Court

Article 101. The Federal Supreme Court shall be composed of eleven justices, chosen from citizens between thirty-five and sixty-five years of age, with renowned legal knowledge and the highest moral reputation.

Single paragraph. The justices of the Federal Supreme Court shall be appointed by the president of the Republic, after their nomination has been approved by an absolute majority of the Federal Senate.

Article 102. The Federal Supreme Court has, essentially, responsibility for safeguarding the Constitution with the power:

I – to preside over and try under its original jurisdiction:

a) direct actions of unconstitutionality of federal or state laws or normative acts, and declaratory actions of constitutionality of federal laws or normative acts; (CA 3, 1993)

b) common criminal offenses committed by the president of the Republic, the vice-president, the members of the National Congress, its own justices and the federal attorney general;

c) common criminal offenses and crimes of responsibility committed by the ministers of State and the commanders of the Navy, the Army, and the Air Force, except as provided in Article 52, item I, the members of the Superior Courts, those of the Federal Accounting Court and the heads of permanent diplomatic missions; (CA 23, 1999)

d) *habeas corpus*, when the petitioner is any one of the people referred to in the preceding subitems; *writ of mandamus* and *habeas data* against acts of the president of the Republic, of the Directing Boards of the Chamber of Deputies and of the Federal Senate,

of the Federal Accounting Court, of the federal attorney general and of the Federal Supreme Court itself;

e) litigation between a foreign State or an international organization and the Union, a state, the Federal District or a Territory;

f) disputes and conflicts between the Union and the states, the Union and the Federal District, or with one another, including the respective associated entities;

g) extradition requested by a foreign State;

h) (Repealed) (CA 45, 2004)

i) *habeas corpus*, when the constraining party is a Superior Court, or when the constraining party or the petitioner is an authority or employee whose acts are directly subject to the jurisdiction of the Federal Supreme Court, or in the case of a crime, subject to the same jurisdiction in one sole instance; (CA 22, 1999)

j) criminal review and actions to vacate its final decisions;

l) claims for the preservation of its powers and guarantee of the enforcement of its decisions;

m) enforcement of court decisions in the cases where it has original competence; the court may delegate duties of procedural acts;

n) suits in which all members of the judicature are directly or indirectly involved, and suits in which more than half of the members of the court of origin are disqualified or have a direct or indirect interest;

o) conflicts of powers between the Superior Court of Justice and any other courts, between Superior Courts, or between the latter and any other court;

p) petitions of provisional remedy in direct actions of unconstitutionality;

q) *writs* of injunction, when the regulation is the president of the Republic's responsibility, of the National Congress, of the Chamber of Deputies, of the Federal Senate, of the Directing Boards of one of these legislative Houses, of the Federal Accounting Court, of one of the Superior Courts, or of the Federal Supreme Court itself;

r) lawsuits against the National Council of Justice and against the National Council of the Prosecution Office; (CA 45, 2004)

II – to try on ordinary appeal:

a) *habeas corpus*, *writs of mandamus*, *habeas data* and *writs of injunction* decided in a sole instance by the Superior Courts, in the event of a denial;

b) political crimes;

III – to try, on extraordinary appeal, cases decided in a sole or last instance, when the decision challenged:

a) is contrary to a provision of this Constitution;

b) declares a treaty or a federal law unconstitutional;

c) considers valid a law or an act of a local government contested in the light of this Constitution;

d) considers valid a local law challenged in the light of a federal law. (CA 45, 2004)

Paragraph 1. A claim of non-compliance with a fundamental precept arising from this Constitution shall be reviewed by the Federal Supreme Court, as provided by law. (Numbered paragraph 1 by CA 3, 1993)

Paragraph 2. Final decisions on merits, entered by the Federal Supreme Court in direct actions of unconstitutionality and declaratory actions of constitutionality shall be binding and effective against all, as regards the other bodies of the judicial branch and the government bodies and associated entities in the federal, state, and local levels. (CA 45, 2004)

Paragraph 3. In an extraordinary appeal, the appellant must demonstrate the general repercussion of the constitutional issues discussed in the case, as provided by law, so the Court may examine the possibility of hearing the appeal, which may be rejected only by the opinion of two-thirds of its members. (CA 45, 2004)

Article 103. The following may file direct actions of unconstitutionality and declaratory actions of constitutionality: (CA 45, 2004)

I – the president of the Republic;

II – the Directing Board of the Federal Senate;

III – the Directing Board of the Chamber of Deputies;

IV – the Directing Board of a State Legislative Assembly or of the Federal District Legislative Chamber; (CA 45, 2004)

V – a state governor or the Federal District governor; (CA 45, 2004)

VI – the federal attorney general;

VII – the Federal Council of the Brazilian Bar Association;

VIII – a political party represented in the National Congress;

IX – a confederation of labor unions or a professional association of a nationwide nature.

Paragraph 1. The federal attorney general shall be previously heard in actions of unconstitutionality and in all suits under the jurisdiction of the Federal Supreme Court.

Paragraph 2. When unconstitutionality is declared on account of lack of a measure to render a constitutional provision effective, the competent branch shall be notified for the adoption of the necessary actions and, in the case of an administrative body, to do so within thirty days.

Paragraph 3. When the Federal Supreme Court examines the unconstitutionality in the abstract of a legal provision or normative act, it shall first summon the general counsel to the federal government, who shall defend the challenged act or text.

Paragraph 4. (Repealed) (CA 45, 2004)

Article 103-A. The Federal Supreme Court may, on its own initiative or upon request, by decision of two thirds of its members, and following reiterated judicial decisions on constitutional matter, issue a *summula* which, as from publication in the official press, shall have a binding effect upon the lower courts of the Judicial branch and the government bodies and associated entities, in the federal, state, and local levels, and which may also be reviewed or repealed, as set forth in law. (CA 45, 2004)

Paragraph 1. The purpose of a *summula* is to validate, construe, and impart effectiveness to some rules about which there is a current controversy among judicial bodies or among such bodies and the public administration, and such controversy brings about serious legal uncertainty and the filing of multiple lawsuits involving similar issues. (CA 45, 2004)

Paragraph 2. Without prejudice to the provisions, the law may establish that the issuance, review, or revocation of a *summula* may be requested by those who may file a direct action of unconstitutionality. (CA 45, 2004)

Paragraph 3. An administrative act or judicial decision which contradicts the applicable *summula* or which unduly applies a *summula* may be appealed to the Federal Supreme Court, and if the appeal is granted, such Court shall declare the administrative act null and void or overrule the appealed judicial decision, ordering a new judicial decision to be issued, with or without applying the *summula*, as the case may be. (CA 45, 2004)

Article 103-B. The National Council of Justice shall be composed of 15 (fifteen) members appointed for a two-year term of office, being permitted one reappointment, according to the following criteria: (CA 61, 2009)

I – the chief justice of the Federal Supreme Court; (CA 61, 2009)

II – a justice of the Superior Court of Justice, nominated by said Court; (CA 45, 2004)

III – a justice of the Superior Labor Court, nominated by said Court; (CA 45, 2004)

IV – a judge of a state Court of Appeal, nominated by the Federal Supreme Court; (CA 45, 2004)

V – a state judge, nominated by the Federal Supreme Court; (CA 45, 2004)

VI – a judge of a Regional Federal Court of Appeal, nominated by the Superior Court of Justice; (CA 45, 2004)

VII – a federal judge, nominated by the Superior Court of Justice; (CA 45, 2004)

VIII – a judge of a Regional Labor Court of Appeal, nominated by the Superior Labor Court; (CA 45, 2004)

IX – a labor judge, nominated by the Superior Labor Court; (CA 45, 2004)

X – a member of the Prosecution Office of the Union, nominated by the federal attorney general; (CA 45, 2004)

XI – a member of a state Prosecution Office, chosen by the federal attorney general from among the names indicated by the competent body of each state institution; (CA 45, 2004)

XII – two lawyers, nominated by the federal board of the Brazilian Bar Association; (CA 45, 2004)

XIII – two citizens of renowned legal knowledge and the highest moral reputation, one of whom nominated by the Chamber of Deputies and the other one by the Federal Senate. (CA 45, 2004)

Paragraph 1. The Council shall be presided over by the chief justice of the Federal Supreme Court and, in the event of his absence or impediment, by the most senior associate justice of the Federal Supreme Court. (CA 61, 2009)

Paragraph 2. The other members of the Council shall be appointed by the president of the Republic, after their nomination has been approved by an absolute majority of the Federal Senate. (CA 61, 2009)

Paragraph 3. If the nominations set forth in this Article are not made within the legal deadline, selection shall be incumbent upon the Federal Supreme Court. (CA 45, 2004)

Paragraph 4. It is incumbent upon the Council to control the administrative and financial operation of the Judicial branch and the proper discharge of official duties by judges, and it shall, in addition to other duties that the statute of the judicature may confer upon it: (CA 45, 2004)

I – to ensure that the judicial branch is autonomous, and that the statute of the judicature is complied with; it may issue regulatory acts within its jurisdiction, or recommend measures; (CA 45, 2004)

II – to ensure that Article 37 is complied with, and examine, on its own initiative or upon request, the legality of administrative acts carried out by members or bodies of the judicial branch; being able to repeal or review them, or stipulate a deadline for the adoption of the necessary measures to achieve due enforcement of the law, without prejudice to the jurisdiction of the Federal Accounting Court; (CA 45, 2004)

III – to receive and examine complaints against members or bodies of the judicial branch, including against its ancillary services, clerical offices, and bodies in charge of notary and registration services which operate due to government delegation or have

been made official, without prejudice to the courts' disciplinary competence and their power to correct administrative acts; it may order that pending disciplinary proceedings be forwarded to the National Council of Justice, determine the removal, placement on paid availability, or retirement with compensation or pension in proportion to the length of service, and enforce other administrative sanctions, full defense being ensured; (CA 103, 2019)

IV – to present a formal charge to the Prosecution Office, in case of crime against public administration or abuse of authority; (CA 45, 2004)

V – to review, on its own initiative or upon request, disciplinary proceedings against judges and members of courts tried in the preceding twelve months; (CA 45, 2004)

VI – to prepare a half-year statistical report on proceedings and judgements rendered per federal entity in the various bodies of the judicial branch; (CA 45, 2004)

VII – to prepare a yearly report, including the measures it deems necessary, on the state of the judicial branch in the country and on the Council's activities, which report must be an integral part of a message to be forwarded by the chief justice of the Federal Supreme Court to the National Congress upon the opening of the legislative session. (CA 45, 2004)

Paragraph 5. The justice of the Superior Court of Justice shall occupy the position of head of the Disciplinary Board of the Court, in charge of internal affairs, and he shall be excluded from the assignment of proceedings in said Court, the following duties being incumbent upon him, in addition to those that may be conferred upon him by the statute of the judicature: (CA 45, 2004)

I – to receive complaints and accusations from any interested party regarding judges and judiciary services; (CA 45, 2004)

II – to exercise executive functions of the Council concerning inspection and general correction; (CA 45, 2004)

III – to request and appoint judges, delegating powers to them, and to request court employees, including in the states, Federal District, and Territories. (CA 45, 2004)

Paragraph 6. The federal attorney general and the chairman of the federal board of the Brazilian Bar Association shall be competent to petition before the Council. (CA 45, 2004)

Paragraph 7. The Union shall establish justice ombudsman's offices, including in the Federal District and in the Territories, with jurisdiction to receive complaints and accusations from any interested party against members or bodies of the judicial branch, or against their ancillary services, thus presenting formal charges directly to the National Council of Justice. (CA 45, 2004)

SECTION III

The Superior Court of Justice

Article 104. The Superior Court of Justice shall be composed of thirty-three justices at minimum.

Single paragraph. The justices of the Superior Court of Justice shall be appointed by the president of the Republic chosen from Brazilians between thirty-five and sixty-five years of age, with renowned legal knowledge and the highest moral reputation, after their nomination has been approved by an absolute majority of the Federal Senate, as follows: (CA 45, 2004)

I – one-third from the judges of the Regional Federal Courts of Appeal and one-third from the judges of the Courts of Appeal of the states, nominated in a list of three names prepared by the court itself;

II – one-third, in equal parts, from the lawyers and members of the Federal, state, Federal District and Territorial Prosecution Office, nominated alternately, according to Article 94.

Article 105. The Superior Court of Justice has the power to:

I – preside over and try under its original jurisdiction:

a) for common crimes, the governors of the states and of the Federal District, and, for such crimes and for crimes of responsibility, the judges of the Courts of Appeal of the states and of the Federal District, the members of the Accounting Courts of the states and of the Federal District, those of the Regional Federal Courts of Appeal, of the Regional Electoral and Labor Courts of Appeal, the members of Accounting Councils or Courts of the municipalities and the members of the Prosecution Office of the Union who act before second instance or superior courts;

b) *writs of mandamus* and *habeas data* against an act of a minister of State, of the commanders of the Navy, the Army, and the Air Force, or of the court itself; (CA 23,1999)

c) *habeas corpus*, when the constraining party or the petitioner is any of the people mentioned in subitem *a*, or when the constraining party is a court subject to its jurisdiction, a minister of State or commander of the Navy, the Army, or the Air Force, except for the competence of the Electoral Courts; (CA 23, 1999)

d) conflicts of competence between any courts, except for the provision in Article 102, item I, subitem *o*, as well as between a court and the judges not subject to it and between judges subject to different courts;

e) criminal review of and actions to vacate its final decisions;

f) claims to preserve its competence and guarantee the enforcement of its decisions;

g) conflicts of duties between administrative and judicial authorities of the Union, or between judicial authorities of one state and administrative authorities of another or of the Federal District, or between those of the latter and those of the Union;

h) *writs of injunction*, when the elaboration of a regulation is the responsibility of a federal body, entity, or authority, of the government bodies or associated entities, with the exception of the cases within the competence of the Federal Supreme Court and of the bodies of the Military Justice, of the Electoral Justice, of the Labor Justice and of the Federal Justice;

i) the homologation of foreign court decisions and the granting of *exequatur* to letters rogatory; (CA 45, 2004)

II – to try on ordinary appeal:

a) *habeas corpus* decided in a sole or last instance by Regional Federal Courts of Appeal or by Courts of Appeal of the states, of the Federal District and the Territories, in the event of a denial;

b) *writs of mandamus* decided in a sole instance by Regional Federal Courts of Appeal or by Courts of Appeal of the states, of the Federal District and the Territories, in the event of a denial;

c) cases in which the parties are a foreign State or international organization, on the one part, and a municipality or a person residing or domiciled in the country, on the other part;

III – to try, on special appeal, the cases decided, in a sole or last instance, by the Regional Federal Courts of Appeal or by the Courts of Appeal of states, of the Federal District and the Territories, when the decision appealed:

a) is contrary to a treaty or a federal law, or denies it effectiveness;

b) considers valid an act of local government challenged in the light of a federal law; (CA 45, 2004)

c) confers upon a federal law an interpretation different from that which has been conferred upon it by another court.

Single paragraph. The following shall operate in conjunction with the Superior Court of Justice: (CA 45, 2004)

I – the Brazilian National Judicial School for Formation and Development, which shall be in charge, among other duties, of regulating the official courses for admission into and promotion in the judicial career; (CA 45, 2004)

II – the Council of Federal Justice, which shall, under the terms of law, exercise administrative and budget supervision over the Federal Courts of first and second instances, in the quality of the main body of the system, having powers to correct administrative acts, and whose decisions shall have a binding nature. (CA 45, 2004)

SECTION IV
The Federal Regional Courts and the Federal Judges

Article 106. The following are bodies of the Federal Justice:

I – Regional Federal Courts of Appeal;

II – federal judges.

Article 107. Regional Federal Courts of Appeal shall be composed of a minimum of seven judges, selected, whenever possible, in the respective region and nominated by the president of the Republic from Brazilians between thirty and sixty-five years of age, as follows:

I – one-fifth from lawyers effectively practicing their professional activity for more than ten years and from members of the Federal Prosecution Office, with over ten years of service;

II – the remainder, by means of promotion of federal judges with over five years in office, alternating seniority and merit.

Paragraph 1. A law shall regulate removal or exchange of judges of the Regional Federal Courts of Appeal and shall determine their jurisdiction and judicial district of seat. (CA 45, 2004)

Paragraph 2. Regional Federal Courts of Appeal shall install an itinerant justice system, carrying out hearings and other functions typical of the operation of justice, within the territorial limits of the respective jurisdiction, and making use of public and community facilities. (CA 45, 2004)

Paragraph 3. Regional Federal Courts of Appeal may operate in a decentralized mode, by creating regional divisions, with a view to affording claimants full access to justice in all stages of the judicial proceedings. (CA 45, 2004)

Article 108. Regional Federal Courts of Appeal have the power to:

I – preside over and try under its original jurisdiction:

a) federal judges within the area of their jurisdiction, including those of the Military and Labor Courts, for common crimes and crimes of responsibility, and the members of the Prosecution Office of the Union, except for the jurisdiction of the Electoral Courts;

b) criminal reviews and actions to vacate their final decisions or those of the federal judges of the region;

c) *writs of mandamus* and *habeas data* against an act of the court itself or of a federal judge;

d) *habeas corpus*, when the defendant is a federal judge;

e) conflicts of competence between federal judges subject to the court;

II – try, on appeal, cases decided by federal and state judges exercising federal competence within the area of their jurisdiction.

Article 109. Federal judges have jurisdiction to preside over and try:

I – cases in which the Union, agencies or federal state-owned companies have an interest as plaintiffs, defendants, assistants or opponents, with the exception of bankruptcy cases, of job-related accidents, and of those subject to the Electoral and Labor Courts;

II – cases between a foreign State or international organization and a municipality or a person domiciled or residing in the country;

III – cases based on a treaty or a contract between the Union and a foreign State or international organization;

IV – political crimes and criminal offenses committed against the assets, services or interests of the Union or its agencies or companies, excluding misdemeanors with the exception of the jurisdiction of the Military and Electoral Courts;

V – crimes covered by an international treaty or convention, when, the prosecution having started in the country, the result has taken place or should have taken place abroad, or conversely;

V-A – cases regarding human rights referred to in paragraph 5 of this Article; (CA 45, 2004)

VI – crimes against the organization of labor and, in the cases determined by law, those against the financial system and the economic and financial order;

VII – *habeas corpus*, for criminal matters within their jurisdiction or when the coercion is exercised by an authority whose acts are not directly subject to another jurisdiction;

VIII – *writs of mandamus* and *habeas data* against an act of a federal authority, except for the cases within the jurisdiction of the Federal Courts;

IX – crimes committed aboard ships or aircrafts, with the exception of the jurisdiction of the Military Courts;

X – crimes or irregular entry or stay of a foreigner, execution of letters rogatory, after *exequatur*, and of foreign court decisions, after homologation, cases related to nationality, including the respective option, and to naturalization;

XI – disputes over indigenous rights.

Paragraph 1. Cases in which the Union is the plaintiff shall be instituted in the judicial section where the other party is domiciled.

Paragraph 2. Cases brought against the Union may be filed in the judicial section where the plaintiff is domiciled, or where the act or fact giving rise to the suit occurred or where the item is located, or even, in the Federal District.

Paragraph 3. The law may grant the jurisdiction to the states to preside over and try cases under the federal jurisdiction in which the parties are a social security institution and its insured whenever the district of the insured has no federal court of first instance. (CA 103, 2019)

Paragraph 4. In the event of the preceding paragraph, the appropriate appeal shall always be taken to the Regional Federal Court of Appeal within the jurisdiction of the first instance court.

Paragraph 5. In cases of serious human rights violations, and with a view to ensuring compliance with obligations arising from international human rights treaties to which Brazil is a party, the federal attorney general may request the removal of the action or the inquiry to the Federal Justice before the Superior Court of Justice upon an incident of competence in the course of any of their stages. (CA 45, 2004)

Article 110. Each state, as well as the Federal District, shall be a judicial section, which shall have its seat in the respective capital, and there shall be courts of first instance located where established by law.

Single paragraph. In the Federal Territories, the jurisdiction and duties attributed to federal judges shall be within the competence of the judges of the local justice, as provided by law.

SECTION V

Labor Courts and Judges (CA 92, 2016)

Article 111. The following are bodies of the Labor Justice:

I – the Superior Labor Court;

II – the Regional Labor Courts of Appeal;

III – labor judges. (CA 24, 1999)

Paragraph 1. (Repealed) (CA 45, 2004)

Paragraph 2. (Repealed) (CA 45, 2004)

Paragraph 3. (Repealed) (CA 45, 2004)

Article 111-A. The Superior Labor Court shall be composed of twenty-seven justices, chosen from Brazilians between thirty-five and sixty-five years of age, with renowned legal knowledge and the highest moral reputation, appointed by the president of the Republic upon approval by an absolute majority of the Federal Senate, as follows: (CA 92, 2016)

I – one-fifth from lawyers effectively practicing their professional activity for more than ten years and from members of the Labor Prosecution Office with over ten years of effective exercise, with due regard for the provisions of Article 94; (CA 45, 2004)

II – the remainder, from career judges of the Regional Labor Courts of Appeal, nominated by the Superior Labor Court. (CA 45, 2004)

Paragraph 1. The law shall provide for the powers of the Superior Labor Court. (CA 45, 2004)

Paragraph 2. The following shall operate in conjunction with the Superior Labor Court: (CA 45, 2004)

I – the National School for the Education and Further Development of Labor Judges, which shall have the duty, inter alia, to regulate the official courses for admission into and promotion in the career; (CA 45, 2004)

II – the Higher Council of Labor Justice, which shall, as provided by law, exercise administrative, budget, financial, and property supervision over Labor Courts of first and second instances, in the quality of central body of the system, whose decisions shall have a binding effect. (CA 45, 2004)

Paragraph 3. The Superior Labor Court has the original jurisdiction to preside over and try cases to preserve its competence and to guarantee the authority of its decisions. (CA 92, 2016)

Article 112. The law shall establish Labor Courts of first instance, allowing, in districts not covered by their jurisdiction, for the attribution of such jurisdiction to judges, appeals being admissible to the respective Regional Labor Court of Appeal. (CA 45, 2004)

Article 113. The law shall regulate the constitution, installation, jurisdiction, powers, guarantees, and conditions of exercise of the bodies of the Labor Justice. (CA 24, 1999)

Article 114. The Labor Justice has jurisdiction to preside over and try: (CA 45, 2004)

I – judicial actions arising from labor relations, comprising entities of public international law and of the government bodies and associated entities of the Union, states, Federal District, and municipalities; (CA 45, 2004)

II – judicial actions involving the exercise of the right to strike; (CA 45, 2004)

III – judicial actions regarding union representation, when the opposing parties are trade unions, or trade unions and workers, or trade unions and employers; (CA 45, 2004)

IV – *writs of mandamus*, *habeas corpus*, and *habeas data*, when the action being challenged involves matter under the Labor Justice jurisdiction; (CA 45, 2004)

V – conflicts of powers between bodies having jurisdiction over labor issues, except as provided under Article 102, item I, subitem *o*; (CA 45, 2004)

VI – judicial actions arising from labor relations which seek compensation for pecuniary loss or emotional distress; (CA 45, 2004)

VII – judicial actions regarding administrative penalties imposed upon employers by the bodies charged with supervising labor relations; (CA 45, 2004)

VIII – enforcement on its own initiative of the social-security contributions set forth in Article 195, items I, subitem *a*, and II, and their legal increments, arising from the decisions it enters; (CA 45, 2004)

IX – other disagreements arising from labor relations, as provided by law. (CA 45, 2004)

Paragraph 1. If collective negotiations are unsuccessful, the parties may elect arbitrators.

Paragraph 2. If any of the parties refuses collective negotiation or arbitration, they may file a collective labor suit of economic nature, by mutual agreement, and Labor Courts may settle the conflict, respecting the minimum legal provisions for the protection of labor, as well as any provisions previously agreed upon. (CA 45, 2004)

Paragraph 3. In the event of a strike in an essential activity that may possibly harm the public interest, the Labor Prosecution Office may file a collective labor suit, and it is incumbent upon Labor Courts to settle the conflict. (CA 45, 2004)

Article 115. Regional Labor Courts of Appeal shall be composed of a minimum of seven judges, selected, whenever possible, from the respective region and appointed by the president of the Republic from Brazilians between thirty and sixty-five years of age, as follows: (CA 45, 2004)

I – one-fifth from lawyers effectively practicing their professional activity for more than ten years and from members of the Labor Prosecution Office with over ten years of effective service, with due regard for the provisions of Article 94; (CA 45, 2004)

II – the remainder, by means of promotion of labor judges, alternatively for seniority and merit. (CA 45, 2004)

Paragraph 1. Regional Labor Courts of Appeal shall install an itinerant justice system, carrying out hearings and other functions typical of the operation of justice, within the territorial limits of the respective jurisdiction, and making use of public and community facilities. (CA 45, 2004)

Paragraph 2. Regional Labor Courts of Appeal may operate in a decentralized mode, by creating regional divisions, with a view to affording claimants full access to justice in all stages of the judicial proceeding. (CA 45, 2004)

Article 116. In the Labor Courts of first instance, jurisdiction shall be exercised by a single judge. (CA 24, 1999)

Single paragraph. (Repealed) (CA 24, 1999)

Article 117. (Repealed) (CA 24, 1999)

SECTION VI

Electoral Courts and Judges

Article 118. The following are bodies of the Electoral Justice:

I – the Superior Electoral Court;

II – the Regional Electoral Courts of Appeal;

III – the electoral judges;

IV – the Electoral Boards.

Article 119. The Superior Electoral Court shall be composed of seven members at minimum, chosen:

I – through election, by secret vote:

a) three judges from the justices of the Federal Supreme Court;

b) two judges from the justices of the Superior Court of Justice;

II – through appointment by the president of the Republic, two judges from six lawyers with renowned legal knowledge and the highest moral reputation, nominated by the Federal Supreme Court.

Single paragraph. The Superior Electoral Court shall elect its chief justice and deputy chief justice from the justices of the Federal Supreme Court, and the head of the Electoral Disciplinary Board from the justices of the Superior Court of Justice.

Article 120. There shall be a Regional Electoral Court of Appeal in the capital of each state and in the Federal District.

Paragraph 1. Regional Electoral Courts of Appeal shall consist of:

I – through election, by secret vote:

a) two judges from the Court of Appeal;

b) two state judges chosen by the Court of Appeal;

II – a judge of the Regional Federal Court of Appeal with its seat in the capital of a state or in the Federal District, or, in the absence thereof, of a federal judge chosen in any case by the respective Regional Federal Court of Appeal;

III – two judges appointed by the president of the Republic, nominated by the Court of Appeal from six lawyers with renowned legal knowledge and the highest moral reputation.

Paragraph 2. The Regional Electoral Court of Appeal shall elect its judges and deputy chief appellate judge from its judges.

Article 121. A supplementary law shall provide for the organization and jurisdiction of the electoral courts, judges and boards.

Paragraph 1. The members of the courts, the court judges and the members of the electoral boards, while in office and insofar as applicable to them, shall enjoy full guarantees and shall not be removed.

Paragraph 2. The judges of the Electoral Courts, except for a justified reason, shall serve for a minimum of two years, and never for more than two consecutive two-year periods, and their substitutes shall be chosen at the same time and through the same procedure, in equal numbers for each category.

Paragraph 3. The decisions of the Superior Electoral Court are unappealable, except for those which are contrary to this Constitution and those denying *habeas corpus* or *writs of mandamus*.

Paragraph 4. Decisions of the Regional Electoral Courts of Appeal may only be appealed against when:

I – they are rendered against an express provision of this Constitution or of a law;

II – there is a divergence in the interpretation of a law between two or more Electoral Courts;

III – they are related to ineligibility or issuance of certificates of electoral victory in federal or state elections;

IV – they annul certificates of electoral victory or decree the loss of federal or state elective offices;

V – they deny *habeas corpus*, *writs of mandamus*, *habeas data* or *writs of injunction*.

SECTION VII

Military Courts and Judges

Article 122. The following are bodies of the Military Justice:

I – the Superior Military Court;

II – the Military Courts and Judges instituted by law.

Article 123. The Superior Military Court shall be composed of fifteen life-tenured justices, appointed by the president of the Republic, after their nomination has been approved by the Federal Senate, three of which shall be chosen from general officers of the Navy, four from general officers of the Army, three from general officers of the Air Force, all of them in active service and in the highest rank of the career, and five from civilians.

Single paragraph. The civil justices shall be chosen by the president of the Republic from Brazilians between over thirty-five years of age, as follows:

I – three from lawyers with renowned legal knowledge and the highest moral reputation, with over ten years of effective professional activity;

II – two, by equal choice, from auditor judges and members of the Prosecution Office of the Military Justice.

Article 124. The Military Courts have jurisdiction to preside over and try military crimes defined by law.

Single paragraph. The law shall make provisions for the organization, operation and competence of the Military Courts.

SECTION VIII

Courts and Judges of the States

Article 125. The states shall organize their judicial system, observing the principles established in this Constitution.

Paragraph 1. The jurisdiction of the courts shall be defined in the Constitution of the state, and the law of judicial organization shall be proposed by the Court of Appeal.

Paragraph 2. The states have jurisdiction to preside over actions of unconstitutionality of state or municipal laws or normative acts in the light of the Constitution of the state, it being forbidden to grant standing to a sole body to act.

Paragraph 3. By proposal of the Court of Appeal, a state law may create the State Military Justice, constituted, at first instance, by judges and by the Councils of Justice and, at second instance, by the Court of Appeal itself, or by the Military Court of Appeal in those states in which the military troops count more than twenty thousand members. (CA 45, 2004)

Paragraph 4. The State Military Justice has jurisdiction to preside over and try members of the state military for military crimes defined by law, in addition to hear and try cases on acts of military discipline, preserving the jurisdiction of the jury when the victim is a

civilian. It shall be the responsibility of the appropriate court to decide upon the loss of post or rank for officers and the loss of grade for servicemen. (CA 45, 2004)

Paragraph 5. The judges of the military justice system have jurisdiction, performing as single judge courts, to preside over and try military crimes committed against civilians and to hear and try judicial cases on acts of military discipline. The Council of Justice, presided over by a judge, has jurisdiction to try other military crimes. (CA 45, 2004)

Paragraph 6. The Court of Appeal may operate in a decentralized mode, by creating regional divisions, with a view to ensuring claimants full access to justice in all stages of the judicial proceeding. (CA 45, 2004)

Paragraph 7. The Court of Appeal shall install an itinerant justice system, carrying out hearings and other functions typical of the operation of justice, within the territorial limits of the respective jurisdiction, and making use of public and community facilities. (CA 45, 2004)

Article 126. For the settlement of conflicts related to land property, the Court of Appeal shall propose the creation of specialized single-judge courts, with exclusive competence for agrarian matters. (CA 45, 2004)

Single paragraph. Whenever efficient jurisdictional service requires it, the judge shall go personally to the site of the litigation.

CHAPTER IV

The Functions Essential to Justice (CA 80, 2014)

SECTION I

The Prosecution Office

Article 127. The Prosecution Office is a permanent institution, essential to the jurisdictional function of the State, and it is its duty to defend the legal order, the democratic regime and the inalienable social and individual interests.

Paragraph 1. Unity, indivisibility and functional independence are institutional principles of the Prosecution Office.

Paragraph 2. The Prosecution Office is ensured functional and administrative autonomy, and it may, observing the provisions of Article 169, propose to the legislative branch the creation and elimination of its offices and auxiliary services, filling them through a competitive civil-service examination of tests or of tests and presentation of

academic and professional credentials, the remuneration policies, and the career plans; the law shall provide for its organization and operation. (CA 19, 1998)

Paragraph 3. The Prosecution Office shall prepare its budget proposal within the limits established in the budget directives law.

Paragraph 4. If the Prosecution Office does not forward its respective budget proposal within the period stipulated in the budget directives law, the executive branch shall, with a view to consolidating the annual budget proposal, take into account the figures approved in the current budget Law, adjusted in accordance with the limits stipulated as provided by paragraph 3. (CA 45, 2004)

Paragraph 5. If the budget proposal referred to in this Article and thus forwarded does not obey the limits stipulated under paragraph 3, the executive branch shall make the necessary adjustments with a view to consolidating the annual budget proposal. (CA 45, 2004)

Paragraph 6. In the implementation of the budget of a specific fiscal year, no expenses may be incurred, and no obligations may be assumed that exceed the limits stipulated in the budget directives law, except when previously authorized, by opening supplementary or special credits. (CA 45, 2004)

Article 128. The Prosecution Office comprises:

I – the Prosecution Office of the Union, which includes:

a) the Federal Prosecution Office;

b) the Labor Prosecution Office;

c) the Military Prosecution Office;

d) the Prosecution Office of the Federal District and the Territories;

II – the Prosecution Offices of the states.

Paragraph 1. The head of the Prosecution Office of the Union is the federal attorney general, appointed by the president of the Republic from career members over thirty-five years of age, upon being approved by an absolute majority of the members of the Federal Senate, for a two-year term of office, reappointment being allowed.

Paragraph 2. The removal of the federal attorney general, on the initiative of the president of the Republic, shall be subject to prior authorization by an absolute majority of the Federal Senate.

Paragraph 3. The Prosecution Offices of the states, of the Federal District and the Territories shall prepare a list of three names from career members, as provided by the respective law, for the selection of their attorney general, who shall be appointed by the head of the executive branch for a two-year term of office, one reappointment being allowed.

Paragraph 4. The attorneys general in the states, in the Federal District and the Territories may be removed from office by deliberation of an absolute majority of the legislative branch, as provided by the respective supplementary law.

Paragraph 5. Supplementary laws of the Union and of the states, which may be proposed by the respective attorneys general, shall establish the organization, the duties and the statute of each Prosecution Office, observing, regarding their members:

I – the following guarantees:

a) of life-tenure office, after two years in office, with loss of office only by a final and unappealable judicial decision;

b) non-removability, except for reason of public interest, through decision of the competent collegiate body of the Prosecution Office, by vote of an absolute majority of its members, full defense being ensured; (CA 45, 2004)

c) irreducibility of compensation, stipulated according to Article 39, paragraph 4, and with due regard for the provisions of Articles 37, items X and XI, 150, item II, 153, item III, 153, paragraph 2, item I; (CA 19, 1998)

II – the following prohibitions:

a) receiving, on any account or for any reason, fees, percentages or court costs;

b) practicing the legal profession;

c) participating in a commercial company, as provided by law;

d) exercising, even when on paid availability, any other public function, except for a teaching position;

e) engaging in political or party activities; (CA 45, 2004)

f) receiving, on any account or for any reason, financial aid or contribution from individuals, and from public or private institutions, except for the cases otherwise provided by law. (CA 45, 2004)

Paragraph 6. The provisions of Article 95, single paragraph, item V, shall apply to the members of the Prosecution Office. (CA 45, 2004)

Article 129. The following are institutional functions of the Prosecution Office:

I – to file public criminal prosecutions, as its exclusive power, according to the law;

II – to safeguard respect by the public authorities and by the services of public relevance for the rights guaranteed in this Constitution, taking the measures required to guarantee such rights;

III – to institute civil investigation and public civil suit to protect public and social property, the environment and other diffuse and collective interests;

IV – to file action of unconstitutionality or representation for purposes of intervention by the Union or by the states, in the cases established in this Constitution;

V – to defend the rights and interests of the indigenous populations in courts;

VI – to issue notifications in administrative procedures within its competence, requesting information and documents to support them, as provided by the respective supplementary law;

VII – to exercise external control over police activities, as provided by the supplementary law mentioned in the previous Article;

VIII – to request investigatory procedures and the institution of police investigation, indicating the legal grounds of its procedural acts;

IX – to exercise other functions which may be conferred upon it, if they are compatible with its purpose, with judicial representation and judicial consultation for public entities being forbidden.

Paragraph 1. Legitimation by the Prosecution Office for the civil actions set forth in this Article shall not preclude those of third parties in the same cases, according to the provisions of this Constitution and of the law.

Paragraph 2. The functions of the Prosecution Office may only be exercised by career members, who must reside in the judicial district of their respective assignment, except when otherwise authorized by the head of the institution. (CA 45, 2004)

Paragraph 3. Admission into the career of the Prosecution Office shall take place by means of a competitive civil-service examination of tests and presentation of academic and professional credentials, ensuring participation by the Brazilian Bar Association in such examination, at least three years of legal practice being required of holders of a B.A. in law, and observing, for appointment, the order of classification. (CA 45, 2004)

Paragraph 4. The provisions of Article 93 shall apply to the Prosecution Office, where appropriate. (CA 45, 2004)

Paragraph 5. In the Prosecution Office, proceedings will be assigned immediately upon filing. (CA 45, 2004)

Article 130. The provisions of this section concerning rights, prohibitions and form of investiture apply to the members of the Accounting Court Prosecution Office.

Article 130-A. The National Council of the Prosecution Office is composed of fourteen members appointed by the president of the Republic, after their nomination has been approved by an absolute majority of the Federal Senate, for a two-year term of office, one reappointment being permitted, as follows: (CA 45, 2004)

I – the federal attorney general, who chairs the Council;

II – four members of the Prosecution Office of the Union, representing each one of its careers;

III – three members of the Prosecution Office of the states;

IV – two judges, one of whom nominated by the Federal Supreme Court and the other one by the Superior Court of Justice;

V – two lawyers, nominated by the federal board of the Brazilian Bar Association;

VI – two citizens with renowned legal knowledge and the highest moral reputation, one of whom nominated by the Chamber of Deputies and the other one by the Federal Senate.

Paragraph 1. The members of the Council who are members of the Prosecution Office shall be nominated by their respective bodies, as provided by law.

Paragraph 2. It is incumbent upon the National Council of the Prosecution Office to control the administrative and financial operation of the Prosecution Office and the proper discharge of official duties by its members, and it shall:

I – ensure that the Prosecution Office is autonomous in its operation and administration, and it may issue regulatory acts within its jurisdiction, or recommend measures;

II – ensure that Article 37 is complied with, and examine, on its own initiative or upon request, the legality of administrative acts carried out by members or bodies of the Prosecution Office of the Union and of the states, and it may revoke or review them, or stipulate a deadline for the adoption of the necessary measures to achieve due execution of the law, without prejudice to the powers of Accounting Courts;

III – receive and examine complaints against members or bodies of the Federal and state Prosecution Office, including against their ancillary services, without prejudice to the institutions' disciplinary competence and their power to correct administrative acts, and it may order that pending disciplinary proceedings be forwarded to the National Council of the Prosecution Office, determine the removal, placement on paid availability, and enforce other administrative sanctions, with the right of full defense being ensured; (CA 103, 2019)

IV – review, on its own initiative or upon request, disciplinary proceedings against members of the Prosecution Office of the Union or of the states tried in the preceding twelve months;

V – prepare a yearly report, including the measures it deems necessary, on the state of the Prosecution Office in the country and on the Council's activities, which report must be an integral part of the message referred to in Article 84, item XI.

Paragraph 3. The Council shall, by means of secret voting, choose a national head of the Disciplinary Board, from the members of the Prosecution Office who compose the

Council, reappointment being forbidden, and the following duties shall be incumbent upon him, in addition to those that may be conferred by law:

I – to receive complaints and accusations from any interested party regarding members of the Prosecution Office and its ancillary services;

II – to exercise executive functions of the Council concerning inspection and general correction;

III – to requisition and to appoint members of the Prosecution Office, delegating specific duties to such members, and to requisition employees of Prosecution Office bodies.

Paragraph 4. The head of the federal board of the Brazilian Bar Association shall be competent to petition before the Council.

Paragraph 5. Federal and state legislation shall establish ombudsman's offices for the Prosecution Office, with power to receive complaints and accusations from any interested party against members or bodies of the Prosecution Office, including against their ancillary services, thus presenting formal charges directly to the National Council of the Prosecution Office.

SECTION II

The Public Advocacy (CA 19, 1998)

Article 131. The Office of the General Counsel to the Federal Government is the institution that, either directly or through a subordinated agency, represents the Union judicially or extra judicially, and it is responsible, under the terms of the supplementary law that provides for its organization and operation, for the activities of judicial consultation and assistance to the executive branch .

Paragraph 1. The Office of the General Counsel to the Federal Government is headed by the general counsel to the federal government, freely appointed by the president of the Republic from citizens over thirty-five years of age, with renowned legal knowledge and the highest moral reputation.

Paragraph 2. Admission into the initial classes of the careers of the institution dealt with in this Article shall take place by means of a competitive civil-service examination of tests and presentation of academic and professional credentials.

Paragraph 3. In the execution of overdue tax liabilities, the Union shall be represented by the Office of the General Counsel to the National Treasury, observing the provisions of the law.

Article 132. The state and the Federal District prosecutors, organized in a career, admission into which shall depend on a competitive civil-service examination of tests and presentation of academic and professional credentials, with the participation of the Brazilian Bar Association in all of its stages, shall exercise judicial representation and judicial consultation for their respective federal entities. (CA 19, 1998)

Single paragraph. The prosecutors referred to in this Article are entitled to tenure after three years of effective exercise, by means of a performance appraisal carried out by the relevant agencies, following a detailed report issued by the Disciplinary Boards. (CA 19, 1998)

SECTION III

The Legal Profession (CA 80, 2014)

Article 133. Lawyers are indispensable to the administration of justice and immune for their acts or manifestations in the practice of their profession, within the limits of the law.

SECTION IV

The Office of the Public Defender (CA 80, 2014)

Article 134. The Office of the Public Defender is a permanent institution, essential to the jurisdictional function of the State, and is responsible primarily, as an expression and an instrument of the democratic regime, for the judicial guidance, the promotion of human rights, and the full and free of charge defense, at all levels, both judicially and extra judicially, of individual and collective rights of the needy, as provided by item LXXIV of Article 5 of the Federal Constitution. (CA 80, 2014)

Paragraph 1. A supplementary law shall organize the Office of the Federal Public Defender and that of the Federal District and Territories and shall provide general rules for their organization in the states, into career offices filled, in the initial class, by means of a competitive civil-service examination of tests and presentation of academic and professional credentials, with the guarantee of non-removability being ensured to its members and the practice of the legal profession beyond the institutional attributions being forbidden. (CA 45, 2004)

Paragraph 2. Offices of the state Public Defenders shall be ensured functional and administrative autonomy, as well as the prerogative to present its budget proposal within

the limits set forth in the budget directives law and in due compliance with the provisions of Article 99, paragraph 2. (CA 45, 2004)

Paragraph 3. The provisions of paragraph 2 shall apply to the Office of the Federal Public Defenders and to that of the Federal District. (CA 74, 2013)

Paragraph 4. Unity, indivisibility, and functional independence are institutional principles of the Offices of Public Defender, and the provisions of Article 93 and of item II of Article 96 of this Federal Constitution shall also apply, insofar as pertinent. (CA 80, 2014)

Article 135. Servants in the careers regulated in Sections II and III of this Chapter shall be remunerated according to Article 39, paragraph 4. (CA 19, 1998)

TITLE V
The Defense of the State and of the Democratic Institutions

CHAPTER I
The State of Defense and the State of Siege

SECTION I
The State of Defense

Article 136. The president of the Republic may, after consulting with the Council of the Republic and the National Defense Council, decree a state of defense to preserve or to promptly re-establish, in specific and restricted locations, the public order or the social peace threatened by serious and imminent institutional instability or affected by major natural calamities.

Paragraph 1. The decree instituting the state of defense shall determine the period of its duration, shall specify the areas to be encompassed and shall indicate, within the terms and limitations of the law, the coercive measures to be in force from the following:

I – restrictions to the rights of:

a) assembly, even if held within associations;

b) secrecy of correspondence;

c) secrecy of telegraph and telephone communication;

II – in the event of a public calamity, occupation and temporary use of public property and services, the Union being liable for the resulting damage and costs arising.

Paragraph 2. The state of defense shall not exceed thirty days and it may be extended once for an identical period if the reasons that justified its declaration persist.

Paragraph 3. During the period in which the state of defense is in force:

I – arrest for a crime against the State, determined by the party enforcing the measure, shall be immediately communicated by such party to the competent judge, who shall release the arrested person if it is illegal, it being the arrested person's choice to request examination of *corpus delicti* from the police authority;

II – the communication shall be accompanied by a statement by the authority as to the physical and mental state of the arrested person at the time of the filing of the charges;

III – the imprisonment or detention of any person shall not exceed ten days, unless authorized by the judicial branch;

IV – the arrested person shall not be forbidden to communicate with the outside world.

Paragraph 4. Upon decreeing a state of defense or extension thereof, the president of the Republic shall, within twenty-four hours, submit the act with the respective justification to the National Congress, which shall decide by an absolute majority.

Paragraph 5. If the National Congress is in recess, it shall be called into extraordinary session within five days.

Paragraph 6. The National Congress shall examine the decree within ten days as from receipt thereof and shall remain in operation for as long as the state of defense is in force.

Paragraph 7. If the decree is rejected, the state of defense shall cease immediately.

SECTION II

The State of Siege

Article 137. The president of the Republic may, after consulting with the Council of the Republic and the National Defense Council, request authorization from the National Congress to decree the state of siege in the event of:

I – serious disturbance with nationwide effects or occurrence of facts that evidence the ineffectiveness of a measure taken during the state of defense;

II – declaration of state of war or response to foreign armed aggression.

Single paragraph. The president of the Republic shall, when requesting authorization to decree the state of siege or to extend it, submit the reasons that determine such request, and the National Congress shall decide by an absolute majority.

Article 138. The decree of the state of siege shall specify the period of its duration, the rules required to implement it and the constitutional guarantees that are to be suspended

and, after it is published, the president of the Republic shall assign the person responsible to enforce the specific measures and the areas encompassed.

Paragraph 1. In the event of Article 137, item I, the state of siege may not be decreed for more than thirty days, nor may each extension exceed such period; in the event of item II, it may be decreed for as long as the war or foreign armed aggression lasts.

Paragraph 2. If authorization to decree the state of siege is requested during parliamentary recess, the president of the Federal Senate shall immediately summon an extraordinary session of the National Congress to convene within five days in order to examine the act.

Paragraph 3. The National Congress shall remain in session until the end of the coercive measures.

Article 139. During the period in which the state of siege decreed under Article 137, item I, is in force, only the following measures may be taken against people:

I – obligation to remain at a specific place;

II – detention in a building not intended for people accused of or convicted for common crimes;

III – restrictions regarding the inviolability of correspondence, the secrecy of communications, the rendering of information and the freedom of press, radio broadcasting and television, as established by law;

IV – suspension of freedom of assembly;

V – home search and seizure;

VI – intervention in public utility companies;

VII – requisitioning of property.

Single paragraph. The broadcasting of speeches made by congress members in their Legislative Houses is not included in the restrictions of item III, if authorized by the respective Directing Board.

SECTION III

General Provisions

Article 140. The Directing Board of the National Congress shall, after consulting with the party leaders, designate a Committee comprised of five of its members to monitor and supervise the implementation of the measures concerning the state of defense and the state of siege.

Article 141. Once the state of defense or the state of siege ceases, its effects shall also cease, without prejudice to liability for illicit acts performed by the enforcers or agents thereof.

Single paragraph. As soon as the state of defense or the state of siege ceases, the measures applied during the period while it is in force shall be reported by the president of the Republic in a message to the National Congress, with specification and justification of the actions taken, with the listing of the names of those affected and indication of the restrictions applied.

CHAPTER II
The Armed Forces

Article 142. The Armed Forces, comprised of the Navy, the Army and the Air Force, are permanent and regular national institutions, organized on the basis of hierarchy and discipline, under the supreme authority of the president of the Republic, and are intended for the defense of the Country, for the guarantee of the constitutional powers, and, on the initiative of any of these, of law and order.

Paragraph 1. A supplementary law shall establish the general rules to be adopted in the organization, training and use of the Armed Forces.

Paragraph 2. *Habeas corpus* shall not apply to military discipline punishments.

Paragraph 3. The members of the Armed Forces are called military, and the following provisions apply to them, in addition to other provisions that the law may establish: (CA 18, 1998)

I – the ranks, with the prerogatives, rights and duties inherent to them, are awarded by the president of the Republic and are guaranteed in full to officers in active service, those in the reserve corps or in retirement, and such officers have exclusive rights to military titles and posts, and, together with the other members, to the use of the uniforms of the Armed Forces; (CA 18, 1998)

II – militaries in active service who take office in a permanent civil public position or job, except in the case provided for in Article 37, item XVI, subitem *c*, shall be transferred to the reserve, as provided by law; (CA 77, 2014)

III – militaries in active service who, under the terms of law, take office in a nonelective, temporary civil public position, job or function, even if in the government associated entities, except in the case provided for in Article 37, item XVI, subitem *c*, shall be put on leave and, as long as remaining in this situation, they may only be promoted by seniority

and their period of service shall be counted only for that promotion and for transfer to the reserve, and after two years, whether continuous or not, away from active service, they shall be transferred to the reserve, as provided by law; (CA 77, 2014)

IV – militaries are forbidden to enlist in a trade union or to go on strike; (CA 18, 1998)

V – while in active service, militaries are forbidden to belong to political parties; (CA 18, 1998)

VI – officers shall only lose post and rank if they are judged unworthy of or incompatible with the dignity of officership by decision of a permanent military court, in times of peace, or of a special court, in times of war; (CA 18, 1998)

VII – officers sentenced with unappealable decision by a common or military court to imprisonment for more than two years shall be submitted to trial as provided in the preceding item; (CA 18, 1998)

VIII – the provisions of Article 7, items VIII, XII, XVII, XVIII, XIX and XXV, and of Article 37, items XI, XIII, XIV and XV, as well as, under the terms of law and priority being given to military activities, the provisions of Article 37, item XVI, subitem *c*, apply to militaries; (CA 77, 2014)

IX – (Repealed) (CA 41, 2003)

X – the law shall provide for admission to the Armed Forces, age limits, tenure, and other conditions for militaries to retire, the rights, duties, remuneration, prerogatives and other circumstances which are specific to them, the special characteristics of their activities being taken into account, including those carried out as a result of international agreements and of war. (CA 18, 1998)

Article 143. Military service is compulsory as set forth by law.

Paragraph 1. It is within the competence of the Armed Forces, according to the law, to assign an alternative service to those who, in times of peace, after being enlisted, claim imperative of conscience, which shall be understood as originating in religious creed and philosophical or political belief, for exemption from essentially military activities.

Paragraph 2. Women and clergymen are exempt from compulsory military service in times of peace but are subject to other duties assigned to them by law.

CHAPTER III
Public Security

Article 144. Public security, the duty of the State and the right and responsibility of all, is exercised to preserve public order and the safety of people and property, by means of the following agencies:

I – Federal Police;

II – Federal Highway Police;

III – Federal Railway Police;

IV – civil polices;

V – military polices and military fire brigades;

VI – federal, state and district correctional police. (CA 104, 2019)

Paragraph 1. The Federal Police, instituted by law as a permanent body, organized and maintained by the Union and structured into a career, are intended to: (CA 19, 1998)

I – investigate criminal offenses against the political and the social order or damaging to property, services and interests of the Union and its entities and companies, as well as other offenses with interstate or international effects that require uniform repression as the law shall establish;

II – to prevent and repress the illegal traffic in narcotics and similar drugs, as well as smuggling, without prejudice to action by tax authorities and other government entities in their respective areas of competence;

III – to perform the functions of maritime, airport and border police; (CA 19, 1998)

IV – to perform exclusively the functions of the judicial police of the Union.

Paragraph 2. The Federal Highway Police are a permanent body organized and maintained by the Union, structured into a career, and intended, according to the law, to patrol overtly the federal highways. (CA 19, 1998)

Paragraph 3. The Federal Railway Police are a permanent body organized and maintained by the Union, structured into a career, and intended, according to the law, to patrol overtly the federal railways. (CA 19, 1998)

Paragraph 4. Excluding the jurisdiction of the Union, the civil police, directed by career police chiefs, have the competence to act as judicial police and to investigate criminal offenses, except for the military ones.

Paragraph 5. The military police are responsible for overtly policing and preserving the public order; the military fire brigades, in addition to the duties defined by law, are responsible for carrying out activities of civil defense.

Paragraph 5-A. The correctional police are responsible for maintaining prison facilities safe and are subordinated to the criminal system body of the federal entity they are placed at. (CA 104, 2019)

Paragraph 6. The military police and military fire brigades, ancillary forces, and reserve corps of the Army are subordinated, together with the civil police and the state and district correctional police, to the governors of the states, Federal District, and Territories. (CA 104, 2019)

Paragraph 7. The law shall regulate the organization and operation of the bodies responsible for public security in such a manner as to guarantee the efficiency of their activities.

Paragraph 8. The municipalities may organize municipal guards to protect their properties, services and facilities, as the law shall establish.

Paragraph 9. The remuneration of the policemen who are members of the bodies mentioned in this Article shall be stipulated according to paragraph 4 of Article 39. (CA 19, 1998)

Paragraph 10. Road safety, carried out with a view to preserving public order and the safety of people and of their property on public roads: (CA 82, 2014)

I – comprises traffic education, engineering, and supervision, as well as other activities set forth in law, aimed at affording citizens the right to efficient urban mobility; and (CA 82, 2014)

II – is incumbent, to the states, Federal District, and municipalities, on the respective executive bodies or entities and their traffic officers, all organized in a career, as provided by law. (CA 82, 2014)

TITLE VI
Taxation and Budget

CHAPTER I
The National Tax System

SECTION I
General Principles

Article 145. The Union, states, Federal District and municipalities may enact the following taxation:

I – taxes;

II – fees, by reason of the exercise of police power or for the effective or potential use of specific and divisible public services, rendered to taxpayers or made available to them;

III – special assessment, resulting from public works.

Paragraph 1. Whenever possible, taxes shall be charged on individual basis and graded according to the taxpayer's ability to pay; tax authorities may identify the property, incomes and economic activities of taxpayers, especially to achieve effectiveness upon such objectives, respecting personal rights and complying with the provision of the law.

Paragraph 2. Fees shall not have the tax base of taxes.

Article 146. A supplementary law shall:

I – provide for conflicts of competence concerning tax matters among the Union, states, Federal District and municipalities;

II – regulate the constitutional limitations on taxing power;

III – establish general rules for tax legislation, particularly to:

a) definition of taxation and their types, as well as, regarding the taxes specified in this Constitution, the definition of the respective taxable events, tax bases and taxpayers;

b) tax liability, assessment, credit, statute of limitation and laches;

c) adequate tax treatment for the cooperative acts of cooperative associations;

d) the definition of a differentiated and favorable tax treatment to be given to micro and small-sized enterprises, including special or simplified tax regimes in the case of the tax set forth in Article 155, item II, the contributions set forth in Article 195, item I, and paragraphs 12 and 13, and the contribution referred to in Article 239. (CA 42, 2003)

Single paragraph. The supplementary law referred to in item III, subitem *d*, may also establish a single regime for collection of taxes and contributions owed to the Union, states, Federal District, and municipalities, with due regard for the following: (CA 42, 2003)

I – it shall be optional for the taxpayer; (CA 42, 2003)

II – different eligibility requirements may be established for each state; (CA 42, 2003)

III – collection shall be unified and centralized, and the distribution of the share of the funds belonging to the respective federal entities shall be immediate, any withholding or establishment of conditions being forbidden; (CA 42, 2003)

IV – collection, supervision, and levying be shared by the federal entities, a single national roster of taxpayers being adopted. (CA 42, 2003)

Article 146-A. A supplementary law may establish special criteria for taxation, with a view to preventing imbalances in competition, without prejudice to the power of the Union to establish, by law, rules for the same purpose. (CA 42, 2003)

Article 147. In Federal Territories, the Union has the power to levy state taxes and, if the Territory is not divided into municipalities, municipal taxes as well. The Federal District has the power to levy municipal taxes.

Article 148. The Union may, by means of a supplementary law, levy compulsory loans:

I – to meet extraordinary expenses resulting from public calamity, foreign war or imminence thereof;

II – in the case of public investment of urgent nature and relevant national interest, observing the provisions of Article 150, item III, subitem *b*.

Single paragraph. Application of the funds arising from a compulsory loan shall be earmarked to the expense that justified its institution.

Article 149. The Union shall have the exclusive power to institute social contributions, regarding intervention in the economic domain and the interest of categories of employees or employers, as instrument of its activity in the respective areas, observing the provisions

of Articles 146, item III, and 150, items I and III, and without prejudice to the provisions of Article 195, paragraph 6, as regards the contributions mentioned in the latter Article.

Paragraph 1. The Union, the states, the Federal District and the municipalities will enact, by statutory law, contributions to fund their own social security regime, charged to active employees, retirees and pensioners, which may have progressive rates according to the amount of the tax base or of the retirement and pension benefits. (CA 103, 2019)

Paragraph 1-A. In the event of actuarial deficit, the ordinary contribution of retirees and pensioners may be levied on the amount of retirement and pension benefits that exceeds the minimum wage. (CA 103, 2019)

Paragraph 1-B. Once the insufficiency of the measure provided for in paragraph 1-A is demonstrated to settle the actuarial deficit, an extraordinary contribution may be enacted for active civil servants, retirees and pensioners in the federal level. (CA 103, 2019)

Paragraph 1-C. The extraordinary contribution referred to in paragraph 1b must be enacted simultaneously with other measures to balance the deficit and shall be in force for a specified period counting from the date of its institution. (CA 103, 2019)

Paragraph 2. The social contributions and the contribution for the intervention in the economic domain dealt with in the head provision: (CA 33, 2001)

I – shall not be levied on export proceeds; (CA 33, 2001)

II – shall also be levied on the importation of foreign products or services; (CA 42, 2003)

III – may have the following rates: (CA 33, 2001)

a) *ad valorem* rates, based upon the proceeds, gross revenues, or the value of the transaction, and, in the case of importation, the customs value; (CA 33, 2001)

b) specific rates based upon the unit of measurement adopted. (CA 33, 2001)

Paragraph 3. An individual who imports may be treated as the equivalent of a legal entity, as provided by law. (CA 33, 2001)

Paragraph 4. The law shall establish the cases in which contributions will be levied only once. (CA 33, 2001)

Article 149-A. The municipalities and the Federal District may establish a contribution, as provided by their respective laws, to finance the public lighting service, with due regard for the provisions of Article 150, items I and III. (CA 39, 2002)

Single paragraph. The contribution mentioned in the head provision may be assessed on the bill for the consumption of electricity energy. (CA 39, 2002)

SECTION II
Limitations on the Taxing Power

Article 150. Without prejudice to any other guarantees ensured to taxpayers, the Union, states, Federal District and municipalities are forbidden to:

I – levy or increase taxation without a law to establish it;

II – institute unequal treatment for taxpayers who are in an equivalent situation, it being forbidden to establish any distinction by reason of professional occupation or function performed by them, notwithstanding the legal designation of their incomes, titles or rights;

III – levy taxation:

a) for taxable events that occurred before the law which instituted or increased them went into force;

b) in the same fiscal year in which the law that instituted or increased them was published;

c) within the period of ninety days from the date of publication of the law which instituted or raised them, with due regard for the provision of subitem *b*; (CA 42, 2003)

IV – use taxation for the purpose of confiscation;

V – establish limitations on the movement of people or goods, by means of interstate or inter-municipal taxations, except for collection of toll fees for use of highways maintained by the government;

VI – enact taxes on:

a) property, income or services of one another;

b) temples of any denomination;

c) property, income or services of political parties, including their foundations, labor unions, non-profit education and social assistance institutions, observing the requirements of the law;

d) books, newspapers, periodicals and paper intended for the printing thereof;

e) musical phonograms and videophonograms produced in Brazil containing musical works or literary-musical works by Brazilian authors and/or works in general interpreted by Brazilian artists, as well as the physical media or digital files containing such works, except in the phase of industrial replication of laser-readable optical media. (CA 75,2013)

Paragraph 1. The prohibition set forth in item III, subitem *b*, shall not apply to the taxations provided in Articles 148, item I, 153, items I, II, IV, and V; and 154, item II; and the prohibition set forth in item III, subitem *c*, shall not apply to the taxations provided

in Articles 148, item I, 153, items I, II, III, and V; and 154, item II, nor to the stipulation of the tax base of the taxes provided in Articles 155, item III, and 156, item I. (CA 42, 2003)

Paragraph 2. The prohibition set forth in item VI, subitem *a*, extends to the government agencies and to the foundations instituted and maintained by the government, regarding the property, income and services related to their essential purposes or resulting therefrom.

Paragraph 3. The prohibitions set forth in item VI, subitem *a*, and in the preceding paragraph do not apply to the property, income and services related to the exploitation of economic activities governed by the regulations which apply to private undertakings, or in which users pay consideration or prices or tariffs, nor exempt a promisor purchaser of real estate from the obligation to pay tax thereon.

Paragraph 4. The prohibitions set forth in item VI, subitems *b* and *c*, encompass only the property, income and services related to the essential purposes of the entities mentioned therein.

Paragraph 5. The law shall determine measures for consumers to be informed about taxes levied on goods and services.

Paragraph 6. Any subsidy or exemption, reduction of tax base, granting of offset credits from previous transactions, amnesty or remission, related to taxes, fees or contributions, may only be granted by means of a specific federal, state or municipal law, which provides exclusively for the above-enumerated matters or the corresponding tax, fee or contribution, without prejudice to the provisions of Article 155, paragraph 2, item XII, subitem *g*. (CA 3, 1993)

Paragraph 7. The law may impose liability upon taxpayers for payment of a tax or contribution whose taxable event will occur afterwards; if the assumed taxable event does not occur, the taxpayer is ensured immediate and preferential restitution of the amount paid. (CA 3, 1993)

Article 151. The Union is forbidden:

I – to enact taxation that is not uniform throughout the entire national territory or that imply a distinction or preference regarding a state, Federal District or municipality over another, except for the granting of tax incentives for the purpose of promoting the balanced social and economic development of the various regions of the country;

II – to tax income from overdue tax liabilities of the states, Federal District and municipalities, as well as the remuneration and earnings of the respective public agents, at higher levels than those established for its own obligations and agents;

III – to grant exemptions from taxation within the powers of the states, Federal District or municipalities.

Article 152. The states, Federal District and municipalities are forbidden to establish a tax difference between goods and services of any nature by reason of their origin or destination.

SECTION III
Federal Taxes

Article 153. The Union has the power to enact taxes on:

I – importation of foreign products;

II – exportation to other countries of national or nationalized products;

III – income and earnings of any nature;

IV – industrialized products;

V – credit, foreign exchange and insurance transactions, or transactions related to bonds or securities;

VI – rural property;

VII – wealth, as provided by a supplementary law.

Paragraph 1. The executive branch may, observing the conditions and the limits established by law, alter the rates of the taxes listed in items I, II, IV and V.

Paragraph 2. The tax established in item III:

I – shall be based on the criteria of general application, universality and progressive taxation, as provided by law.

II – (Repealed) (CA 20, 1998)

Paragraph 3. The tax established in item IV:

I – shall be selective, based on the essentiality of the product;

II – shall be noncumulative, the tax owed in each transaction shall be offset by the amount charged in previous transactions;

III – shall not be levied on industrialized products intended for export;

IV – shall have a reduced impact on the acquisition of capital goods by the taxpayers, as provided by law. (CA 42, 2003)

Paragraph 4. The tax established in item VI of the head provision: (CA 42, 2003)

I – shall be progressive and have rates determined in such a manner as to discourage the retention of unproductive properties; (CA 42, 2003)

II – shall not be levied on small rural property, as defined by law, when a proprietor who owns no other real estate exploits it; (CA 42, 2003)

III – shall be controlled and collected by the municipalities which opt to do so, as provided by law, if they do not reduce this tax or introduce any other type of fiscal waiver. (CA 42, 2003)

Paragraph 5. Gold, when defined by law as a financial asset or an exchange instrument, is subject exclusively to the tax established in item V of the head provision, due on the original transaction; the minimum rate shall be one percent, and the transference of the amount collected is ensured under the following terms:

I – thirty percent to the state, the Federal District or the Territory, depending on the origin;

II – seventy percent to the municipality of origin.

Article 154. The Union may levy:

I – by supplementary law, taxes not levied in the preceding Article, as long as they are noncumulative and have a specific taxable event or tax base other than those specified in this Constitution;

II – in the case of foreign war or its imminent threat, extraordinary taxes, included or not in its taxing power, which shall be gradually suppressed when the causes for their institution have ceased.

SECTION IV

State and Federal District Taxes

Article 155. The states and the Federal District have the power to enact taxes on: (CA 3, 1993)

I – transfer by death and donation of any property or rights; (CA 3, 1993)

II – transactions related to circulation of goods and the performance of services of interstate and inter-municipal transportation and communication, even when the transactions and the performances begin abroad; (CA 3, 1993)

III – ownership of automotive vehicles. (CA 3, 1993)

Paragraph 1. The tax established in item I: (CA 3, 1993)

I – regarding real estate and the respective rights, is within the competence of the state where the property is located, or of the Federal District;

II – regarding bonds, titles and credits, is within the competence of the Federal District or of the state where the inventory and partition of succession proceeding, or where the donor is domiciled;

III – a supplementary law shall regulate the competence for the institution of such duty:

a) if the donor is domiciled or residing abroad;

b) if the deceased owned property, was resident or domiciled or had their inventory and partition of succession proceeding abroad;

IV – the Federal Senate shall establish the maximum rates for such tax.

Paragraph 2. The tax established in item II shall observe the following: (CA 3, 1993)

I – it shall be noncumulative, the tax owned in each transaction concerning the circulation of goods or provision of services shall be offset by the amount charged in the previous transactions by the same or by another state or by the Federal District;

II – exemption or waiver, except as otherwise determined by law:

a) shall not imply a credit to offset the amount owned in the subsequent transactions or renderings of services;

b) shall cause the annulment of the credit for the previous transactions;

III – it may be selective, based on the essentiality of the goods or services;

IV – a resolution of the Federal Senate, on the initiative of the president of the Republic or of one-third of the senators, approved by an absolute majority of its members, shall establish the rates that apply to interstate and export transactions and provision of services;

V – the Federal Senate may:

a) establish minimum rates for domestic transactions, by means of a resolution on the initiative of one-third and approved by an absolute majority of its members;

b) establish maximum rates for the same transactions to settle a specific conflict involving the interest of the states, by means of a resolution on the initiative of an absolute majority and approved by two-thirds of its members;

VI – unless otherwise determined by the states and the Federal District, as provided by item XII, subitem *g*, the domestic rates for transactions concerning the circulation of goods and the provision of services may not be lower than those established for interstate transactions;

VII – for transactions and instalments that send goods and services to a final consumer located in another state, whether or not such consumer is the taxpayer, the interstate rate is adopted; and it shall be up to the state where the recipient is located to collect the difference between the internal rate of the state and the interstate rate; (CA 87, 2015)

a) (Repealed) (CA 87, 2015)

b) (Repealed) (CA 87, 2015)

VIII – the responsibility for the tax collection corresponding to the difference between the internal rate and the interstate rate referred to in item VII will be assigned to: (CA 87, 2015)

a) the recipient, when it is incumbent upon the recipient to pay that tax; (CA 87, 2015)

b) the remitter, when it is not incumbent upon the recipient to pay that tax; (CA 87, 2015)

IX – it shall also be levied:

a) on the entry of goods or products imported from abroad by an individual or corporate body, even in the case of a taxpayer who does not pay such tax on a regular basis, regardless of its purpose, as well as on services provided abroad, and the tax shall be attributed to the state where the domicile or the establishment of the recipient of the product, good, or service is; (CA 33, 2001)

b) on the total value of the transaction, when goods are supplied with services not included in the taxing power of the municipalities;

X – it shall not be levied:

a) on transactions involving goods to be shipped abroad, nor on services to be delivered to parties abroad, and tax charges and credits in preceding transactions involving such goods or services shall continue in effect; (CA 42, 2003)

b) on transactions transferring petroleum, including lubricants, liquid and gaseous fuels derived therefrom, and electric energy to other states;

c) on gold, in the cases defined in Article 153, paragraph 5;

d) on communication services in the modes of sound broadcasting and sound and image broadcasting which are available for reception by the public free of charge; (CA 42, 2003)

XI – its tax base shall not include the amount of the tax on industrialized products when the transaction carried out between taxpayers that concerns a product intended for industrialization or sale represents a taxable event for both taxes;

XII – a supplementary law shall:

a) define its taxpayers;

b) provide for tax substitution;

c) regulate the system for offsetting taxes;

d) establish, for purposes of tax collection and definition of the responsible establishment, the location of the transactions concerning the circulation of goods and the provision of services;

e) exclude from levy of the tax, in exports to other countries, services and other products other than those mentioned in item X, subitem *a*;

f) provide for the event of maintenance of a credit for services and goods shipping to another state and exported to other countries;

g) regulate the manner in which, through deliberation by the states and the Federal District, tax exemptions, incentives and benefits shall be granted and repealed;

h) define the fuels and lubricants on which this tax shall be levied only once, regardless of its purpose, in which case the provision of item X, subitem *b*, shall not apply; (CA 33, 2001)

i) stipulate the tax base to include the amount of the tax, also in the event of importation of goods, products or services from abroad. (CA 33, 2001)

Paragraph 3. Except for the taxes mentioned in item II of the head provision, and Article 153, items I and II, no other tax may be levied on transactions concerning electric energy, telecommunication services, petroleum products, fuels, and minerals of the country. (CA 33, 2001)

Paragraph 4. In the event of item XII, subitem *h*, the following shall apply: (CA 33, 2001)

I – in transactions involving lubricants and petroleum derived fuels, the tax shall be attributed to the state where consumption occurs; (CA 33, 2001)

II – in interstate transactions among taxpayers involving natural gas and its by products, and lubricants and fuels not included in item I of this paragraph, the tax shall be shared by the state of origin and the state of destination, and the proportion existing in transactions involving other goods shall be observed; (CA 33, 2001)

III – in interstate transactions involving natural gas and its by-products, and lubricants and fuels not included in item I of this paragraph, when it is not incumbent upon the recipient to pay the tax, such tax shall be attributed to the state of origin; (CA 33, 2001)

IV – the tax rates shall be defined by a joint decision of states and the Federal District, under the terms of paragraph 2, item XII, subitem *g*, with due regard for the following: (CA 33, 2001)

a) they shall be uniform throughout the national territory, and they may be different for each product; (CA 33, 2001)

b) they may be specific, according to the unit of measurement adopted, or *ad valorem*, levied on the value of the transaction or on the price of the product or a similar product would be sold for in free competition circumstances; (CA 33, 2001)

c) they may be lowered and restored to their original levels, and the provision of Article 150, item III, subitem *b*, shall not apply thereto. (CA 33, 2001)

Paragraph 5. The rules for the enforcement of the provisions of paragraph 4, including those concerning the collection and assignment of the tax, shall be established by a joint decision of the states and the Federal District, under the terms of paragraph 2, item XII, subitem *g*. (CA 33, 2001)

Paragraph 6. The tax established in item III: (CA 42, 2003)

I – shall have its minimum rates stipulated by the Federal Senate; (CA 42, 2003)

II – may have different rates according to type and utilization. (CA 42, 2003)

SECTION V
Municipal Taxes

Article 156. The municipalities have the power to enact taxes on:

I – urban buildings and urban land property;

II – onerous *inter vivos* transfer of real estate, by any instrument, whether by natural or physical accession, and of interest in property, except for real security, as well as the assignment of rights to the purchase thereof;

III – services of any nature not included in Article 155, item II, as defined in a supplementary law. (CA 3, 1993)

IV – (Repealed) (CA 3, 1993)

Paragraph 1. Without prejudice to the progressiveness in time mentioned in Article 182, paragraph 4, item II, the tax referred to in item I may: (CA 29, 2000)

I – have progressive rates according to the value of the property; and (CA 29, 2000)

II – have different rates according to the location and utilization of the property. (CA 29, 2000)

Paragraph 2. The tax set forth in item II:

I – shall not be levied on the transfer of properties or rights incorporated into the assets of a legal entity to pay-in its capital, nor on the transfer of property or rights resulting from the merger, incorporation, division or dissolution of a legal entity, unless, in such cases, the predominant activity of the purchaser is the purchase and sale of such property or rights, the lease of real estate or leasing;

II – goes to the municipality where the property is.

Paragraph 3. Regarding the tax established in item III of the head provision, a supplementary law shall: (CA 37, 2002)

I – establish its maximum and minimum rates; (CA 37, 2002)

II – exclude exports of services from its application; (CA 3, 1993)

III – regulate the manner and conditions that fiscal exemptions, incentives and benefits shall be granted and repealed. (CA 37, 2002)

Paragraph 4. (Repealed) (CA 3, 1993)

SECTION VI
Sharing of Tax Revenue

Article 157. The following shall be assigned to the states and to the Federal District:

I – the proceeds from collection of the federal taxes on income and earnings of any nature, withheld from income paid, by whatever instrument, by them and the agencies and foundations they institute and maintain;

II – twenty percent of the proceeds from the collection of the taxes that the Union enacts in the exercise of the power conferred to it by Article 154, item I.

Article 158. The following shall be assigned to the municipalities:

I – the proceeds from the collection of the federal taxes on income and earnings of any nature, withheld from income paid, by whatever instrument, by them and the agencies and foundations they institute and maintain;

II – fifty percent of the proceeds from the collection of the federal taxes on rural property, concerning real estate in the municipalities, or the total amount of the proceeds in case the municipalities choose the option referred to in Article 153, paragraph 4, item III; (CA 42, 2003)

III – fifty percent of the proceeds from the collection of the state tax on ownership of automotive vehicles licensed in their territory;

IV – twenty-five percent of the proceeds from the collection of the state tax on transactions of circulation of goods and on the provision of interstate and inter-municipal transportation services and services of communication.

Single paragraph. The revenue portions assigned to the municipalities, referred to in item IV, shall be credited in accordance with the following criteria:

I – at least three-fourths, in proportion to the value added in the transactions of circulation of goods and rendering of services carried out in their territory;

II – up to one-quarter, in accordance with the provisions of a state law or, in the case of the Territories, of a federal law.

Article 159. The Union shall remit:

I – of the proceeds from the tax collection on income and earnings of any nature and of taxes on industrialized products, forty-nine percent (49%) as follows: (CA 84, 2014)

a) twenty-one and one-half percent to the Revenue Sharing Fund of the States and of the Federal District;

b) twenty-two and one-half percent to the Revenue Sharing Fund of the Municipalities;

c) three percent, for application in programs to finance the productive sector of the North, Northeast and Centre-West Regions, through their regional financial institutions,

in accordance with regional development plans, the semi-arid area of the Northeast being ensured half of the funds intended for that Region, as provided by law;

d) one percent to the Revenue Sharing Fund of the Municipalities, to be remitted within the first ten days of the month of December of each year; (CA 55, 2007)

e) one percent to the Revenue Sharing Fund of the Municipalities, to be remitted within the first ten days of the month of July of each year; (CA 84, 2014)

II – of the proceeds from the tax collection on industrialized products, ten percent to the states and to the Federal District, in proportion to the value of the respective exports of industrialized products;

III – of the proceeds from the collection of the contribution for intervention in the economic domain set forth in Article 177, paragraph 4, twenty-nine percent to the states and to the Federal District, distributed in accordance with the law, with due regard for the allocation referred to in item II, subitem *c*, of the mentioned paragraph. (CA 44, 2004)

Paragraph 1. For purposes of calculating the amount to be transferred in accordance with the provisions in item I, the portion of the tax collected on income and earnings of any nature assigned to the states, to the Federal District and to the municipalities shall be excluded, as provided by Articles 157, item I, and 158, item I.

Paragraph 2. No federal entity may have allocated a portion in excess of twenty percent of the amount referred to in item II, and any excess shall be distributed among the other participants, maintaining, for the latter, the apportionment criterion established therein.

Paragraph 3. The states shall transfer twenty-five percent of the funds they may receive as provided by item II to the respective municipalities, observing the criteria established in Article 158, single paragraph, items I and II.

Paragraph 4. Twenty-five percent of the amount of funds referred to in item III and allocated to each state shall be assigned to its municipalities, in accordance with the law referred to in the mentioned item. (CA 42, 2003)

Article 160. It is forbidden to withhold or to make any restriction to the remittance and use of the funds assigned in this section to the states, to the Federal District and to the municipalities, including any tax additions and increases.

Single paragraph. The prohibition mentioned in the present Article does not prevent the Union and the states from remitting the funds, on the following conditions: (CA 29, 2000)

I – payment of their credits, including those of the government agencies; (CA 29, 2000)

II – compliance with the provisions of Article 198, paragraph 2, items II and III. (CA 29, 2000)

Article 161. A supplementary law shall:

I – define the value added for purposes provided by Article 158, single paragraph, item I;

II – establish rules for remittance of funds referred to in Article 159, especially the criteria for sharing of funds set forth in its item I, seeking to promote social and economic balance among states and municipalities;

III – provide for monitoring, by the beneficiaries, of the calculation of the quotas and release of the participation funds set forth in Articles 157, 158 and 159.

Single paragraph. The Federal Accounting Court shall calculate the quotas referring to the participation funds mentioned in item II.

Article 162. The Union, states, Federal District and municipalities shall disclose, by the last day of the month following collection, the amounts of all taxation collected, the funds received, the tax sums remitted and to be remitted and the numerical expression of the apportionment criteria.

Single paragraph. The data disclosed by the Union shall be detailed by state and by municipality; the data released by the states will be broken down by municipality.

CHAPTER II
Public Finances

SECTION I
General Rules

Article 163. A supplementary law shall make provisions for:

I – public finances;

II – foreign and domestic public debt, including the debt of the government agencies, foundations and other entities controlled by the government;

III – granting of guarantees by public entities;

IV – issuance and redemption of public debt bonds;

V – financial supervision of government bodies and associated entities; (CA 40, 2003)

VI – foreign exchange transactions carried out by bodies and agencies of the Union, of the states, of the Federal District and of the municipalities;

VII – compatibility of the functions of the official credit institutions of the Union, safeguarding all the characteristics and full operational conditions of those intended for regional development.

Article 164. The power of the Union to issue currency shall be exercised exclusively through the Central Bank.

Paragraph 1. The Central Bank is forbidden to grant directly or indirectly loans to the National Treasury and to a body or agency which is not a financial institution.

Paragraph 2. The Central Bank may purchase and sell bonds issued by the National Treasury, for the purpose of regulating the money supply or the interest rate.

Paragraph 3. The cash assets of the Union shall be deposited at the Central Bank; those of the states, of the Federal District, of the municipalities and of the government bodies or agencies and government-controlled companies, at official financial institutions, except for the cases established by law.

SECTION II
Budgets

Article 165. Laws proposed on the initiative of the Executive branch shall establish:

I – the multiyear plan;

II – the budget directives;

III – the annual budgets.

Paragraph 1. The law which institutes the pluriannual plan shall establish, on a regional basis, the directives, objectives and targets of the federal administration for the capital expenditures and other expenses resulting therefrom and for those regarding continuous programs.

Paragraph 2. The budget directives law shall comprise the targets and priorities of the federal administration, including the capital expenditures for the subsequent fiscal year, guide the elaboration of the annual budget law, make provisions for alterations in tax legislation and establish the investment policy for the official development of financing agencies.

Paragraph 3. The executive branch shall, within thirty days after the closing of each two-month period, publish a summarized report on budget implementation.

Paragraph 4. The national, regional and sectorial plans and programs set forth in this Constitution shall be drawn up in compliance with the pluriannual plan and shall be examined by the National Congress.

Paragraph 5. The annual budget law shall include:

I – the fiscal budget regarding the branches of the Union, their funds, bodies and entities of the direct administration and associated entities, including foundations instituted and maintained by the government;

II – the investment budget of companies in which the Union directly or indirectly holds the majority of the voting capital;

III – the social welfare budget, comprising all the government bodies and associated entities connected with social security, as well as funds and foundations instituted and maintained by the government.

Paragraph 6. The budget bill shall be accompanied by a regionalized statement on the effect on revenues and expenses, deriving from exemptions, amnesties, remissions, subsidies and benefits of a financial, tax and credit nature.

Paragraph 7. The functions of the budgets set forth in paragraph 5, items I and II, of the present Article, compatible with the pluriannual plan, shall include the function of reducing interregional inequalities, according to population criteria.

Paragraph 8. The annual budget law shall not contain any provision other than a forecast of revenues and the establishment of expenses, such prohibition not including authorization to open supplementary credits and to contract credit transactions, even if by deferred revenue, as provided by law.

Paragraph 9. A supplementary law shall:

I – establish the fiscal year, the effectiveness, the terms, the preparation and organization of the multiyear plan, of the budget directives law and of the annual budget law;

II – establish rules of financial and property management of the government bodies and associated entities, as well as conditions for the institution and operation of funds;

III – provide criteria for the equitable execution, in addition to the procedures that shall be adopted in the event of legal and technical impediments, pay of carryovers and limitations on mandatory programming to accomplish the provisions under paragraphs 11 and 12 of Article 166. (CA 100, 2019)

Paragraph 10. The administration has the duty to execute the budget programming, adopting the means and the necessary measures in order to ensure that goods and services are indeed rendered to society. (CA 100, 2019)

Paragraph 11. Under the budget directives law, the provisions of paragraph 10 of this Article: (CA 102, 2019)

I – are subject to compliance with constitutional and legal provisions that establish fiscal target or limits of expenditure and do not prevent the necessary cancellation to open additional credits; (CA 102, 2019)

II – do not apply in cases of justified technical impediments; (CA 102, 2019)

III – apply exclusively to discretionary primary spending. (CA 102, 2019)

Paragraph 12. It shall integrate into the budget directives law of the fiscal year to which it refers and, at least, of the following two (2) years, attached with an estimate of fiscal aggregates and the proportion of resources for investments to be earmarked from the annual budget law to maintain those in progress. (CA 102, 2019)

Paragraph 13. The provisions of item III of paragraph 9 and paragraphs 10, 11 and 12 of this Article apply exclusively to the fiscal and social welfare budgets of the Union. (CA 102, 2019)

Paragraph 14. The annual budget law may provide an estimate of the amount of expenditure for subsequent fiscal years, specifying multiyear investments and those that are in progress. (CA 102, 2019)

Paragraph 15. The Union shall organize and maintain a record of the investment projects per state or the Federal District, which shall include, at least, a feasibility study, cost estimate, and information on the financial and practical execution of the project. (CA 102, 2019)

Article 166. The bills regarding the multiyear plan, the budget directives, the annual budget and the additional credits shall be examined by both Houses of the National Congress, in accordance with their common regulations.

Paragraph 1. A permanent joint committee of senators and deputies shall:

I – examine and issue its opinion on the bills referred to in the present Article and on the accounts submitted annually by the president of the Republic;

II – examine and issue its opinion on the national, regional and sectorial plans and programs established in this Constitution, and exercise budget monitoring and supervision, without affecting the operation of the other committees of the National Congress and of its Houses, created in accordance with Article 58.

Paragraph 2. Amendments shall be submitted to the joint committee, which shall report on them; and they shall be examined, according to internal rules, by the Plenary of both Houses of the National Congress.

Paragraph 3. Amendments to the bill of the annual budget or to the bills which modify it may only be approved if:

I – they are compatible with the multiyear plan and with the budget directives law;

II – they specify the necessary funds, allowing only those resulting from the annulment of expenses, and excluding those which apply to:

a) personnel expenses and social security contributions;

b) debt servicing;

c) constitutional tax transfers to the states, the municipalities and the Federal District; or

III – they are related:

a) to the correction of errors or omissions; or

b) to the provisions of the text of the bill of law.

Paragraph 4. Amendments to the bill of budget directives may not be approved if they are incompatible with the pluriannual plan.

Paragraph 5. The president of the Republic may send a message to the National Congress to propose modifications in the bills referred to in the present Article as long as the joint committee has not started to vote on the part for which an alteration is being proposed.

Paragraph 6. The bills of the multiyear plan law, of the budget directives law and of the annual budget law shall be forwarded by the president of the Republic to the National Congress, under the terms of the supplementary law referred to in Article 165, paragraph 9.

Paragraph 7. The other rules regarding legislative procedure shall apply to the bills mentioned in this Article, as long they are not contrary to the provisions of this section.

Paragraph 8. Any funds which, as a result of a veto, amendment or rejection of the annual budget bill, have no corresponding expenses, may be allocated, as the case may be, by means of special or supplementary credits, with prior and specific legislative authorization.

Paragraph 9. Individual amendments to the budget bill shall be approved up to the limit of one and two tenths percent (1.2%) of the net current revenue set forth in the bill forwarded by the Executive branch, and half of such percentage shall be assigned for public health actions and services. (CA 86, 2015)

Paragraph 10. The amount spent in public health actions and services as set forth in paragraph 9, including current spending, shall be taken into account for purposes of compliance with Article 198, paragraph 2, item I, however it is forbidden to assign such amount to pay personnel expenditures or social security contributions. (CA 86, 2015)

Paragraph 11. Execution of the budget and financial appropriation referred to in paragraph 9 of this Article is mandatory, in an amount corresponding to one and two tenths percent (1.2%) of the net current revenue collected in the prior fiscal year, according to the criteria for equitable execution of appropriation defined in the supplementary law set forth in paragraph 9 of Article 165. (CA 86, 2015)

Paragraph 12. Execution guarantee referred to in paragraph 11 of this Article shall also apply to appropriations stipulated by all the amendments presented by a parliamentarian state or District bench, at the maximum amount of one percent of the net current revenue in the prior fiscal year. (CA 100, 2019)

Paragraph 13. The budget appropriations set forth in paragraphs 11 and 12 of this Article shall not be subject to mandatory execution in cases of technical impediments. (CA 100, 2019)

Paragraph 14. For the purpose of compliance with the provisions of paragraphs 11 and 12 of this Article, government bodies responsible for execution shall comply, as provided by the budget directives law, with the schedule to analyze and verify any impediments to the appropriations and other necessary procedures to execute the respective amounts. (CA 100, 2019)

I – (Repealed) (CA 100, 2019)

II – (Repealed) (CA 100, 2019)

III – (Repealed) (CA 100, 2019)

IV – (Repealed) (CA 100, 2019)

Paragraph 15. (Repealed) (CA 100, 2019)

Paragraph 16. When the mandatory transfer from the Union, to execute the appropriations set forth in paragraphs 11 and 12 of this Article, is allocated for states, the Federal District, and municipalities, it shall not depend upon the fulfillment of obligations by the recipient federal entity, and it shall not be included in the tax base of the net current revenue for purposes of application of the limits of expenditure on personnel referred to in the head provision of Article 169. (CA 100, 2019)

Paragraph 17. Pay up of carryovers from appropriations provided in paragraphs 11 and 12, may be considered, in order to comply with the financial execution, until the limit of six tenths of one percent (0.6%) of the net current revenue realized in the previous fiscal year, for the appropriations of individual amendments, and, until the limit of five tenths of one percent (0.5%), for the appropriations of amendments established by state or District parliamentarian bench. (CA 100, 2019)

Paragraph 18. Should it be found that a new estimate of the revenue and the expenditure leads to the noncompliance with the targeted fiscal result established in the budget directives law, the amount set forth in paragraphs 11 and 12 of this Article may be reduced by up to the same proportion of the limit applicable to all discretionary spending. (CA 100, 2019)

Paragraph 19. The implementation of appropriations of a mandatory nature is deemed equitable if it meets objective and impartial criteria and if it complies with the presented Amendments on an equal and impersonal basis, regardless of the proponents. (CA 100, 2019)

Paragraph 20. The appropriations referred to in paragraph 12 of this Article, when related to the beginning of investments lasting more than one (1) fiscal year or whose implementation has already begun, shall be amended by the same state parliamentary bench, in each fiscal year, until the end of the construction or the project. (CA 100, 2019)

Article 166-A. Individual amendments of mandatory execution submitted to the annual budget bill may allocate funds to states, the Federal District and municipalities by means of: (CA 105, 2019)

I – a special remittance; or (CA 105, 2019)

II – a defined-purpose remittance. (CA 105, 2019)

Paragraph 1. The funds remitted as provided by the head provision shall not incorporate the revenue of states, of the Federal District and of the municipalities for the purpose of sharing expenditures and calculating the expenditure limits with active and retired personnel, as provided by paragraph 16 of Article 166, and of the federal entity indebtedness; also it is forbidden, in any case, to use the funds referred to in the head provision to pay off: (CA 105, 2019)

I – personnel expenses and social security contributions of active and retired civil servants, and of pensioners; and (CA 105, 2019)

II – expenses related to the public debt servicing. (CA 105, 2019)

Paragraph 2. In the special remittance referred to in item I of the head provision, the funds: (CA 105, 2019)

I – shall be transferred directly to the federal entity, regardless of the signature of an agreement or any other similar instrument; (CA 105, 2019)

II – shall belong to the federal entity upon the moment of the actual financial transfer; and (CA 105, 2019)

III – shall be employed in core activities of the areas of competence of the Executive branch of the benefited federal entity, subject to the provisions of paragraph 5 of this Article. (CA 105, 2019)

Paragraph 3. The federal entity benefiting from the special remittance provided in item I of the head provision may sign technical cooperation contracts to support the monitoring of the budget execution in the use of funds. (CA 105, 2019)

Paragraph 4. In the defined-purpose transfer referred to in item II of the head provision, funds shall be: (CA 105, 2019)

I – subject to the appropriations established in a parliamentary amendment; and (CA 105, 2019)

II – applied in areas of constitutional competence of the Union. (CA 105, 2019)

Paragraph 5. At least seventy percent (70%) of the special remittance referred to in item I of the head provision shall be applied in capital expenditures, while observing the restriction referred to in item II of paragraph 1 of this Article. (CA 105, 2019)

Article 167. The following are forbidden:

I – begin programs or projects not included in the annual budget law;

II – expend funds or assume direct obligations that exceed budget or additional credits;

III – borrow funds in excess of the amount of capital expenditures, except for those which were authorized by supplementary or special credits with a specific purpose and approved by an absolute majority of the legislative branch;

IV – bind tax revenues to a government body, fund or expense; except for sharing of the proceeds from the taxes collected as referred to in Articles 158 and 159; allocation of funds for public health activities and services for the maintenance and development of education and for carrying out tax administration activities, as determined respectively in Article 198, paragraph 2, Article 212, and Article 37, item XXII; and for the provision of guarantees to loans by advance of revenues, as established in Article 165, paragraph 8, as well as in paragraph 4 of the present Article; (CA 42, 2003)

V – open of supplementary or special credit without prior legislative authorization and without indication of the corresponding funds;

VI – reassign, reallocate or remit funds from one appropriation category to another or from one government body to another without prior legislative authorization;

VII – grant or use of unlimited credits;

VIII – use, without specific legislative authorization, funds from the fiscal and social security budgets to supply a necessity or to pay off debt of companies, foundations and funds, including those mentioned in Article 165, paragraph 5;

IX – institute funds of any nature without prior legislative authorization;

X – voluntary remittance of funds and grant of loans, including by deferred revenues, by the Union, the government of the states and their financial institutions, to pay off expenditures related to active and retired personnel and pensioners of the states, of the Federal District, and of the municipalities; (CA 19, 1998)

XI – use the funds from the social security contributions set forth in Article 195, items I, subitem a, and II, to defray expenses other than the payment of benefits of the General Social Security Scheme referred to in Article 201; (CA 20, 1998)

XII – under the supplementary law referred to in paragraph 22 of Article 40, use revenues from the Special social security scheme, including the amounts incorporated into the funds described in Article 249, to pay off expenses other than the benefits of the social security fund subordinated to that scheme and expenses necessary for its organization and operation; (CA 103, 2019)

XIII – voluntary remittance of resources, granting of endorsements, guarantees and subsidies by the Union, and granting of loans and financing by federal financial institutions to the states, the Federal District and the municipalities in the event of non-compliance with the general rules of organization and operation of the Special social security scheme. (CA 103, 2019)

Paragraph 1. No investment whose execution exceeds one fiscal year may be executed without prior inclusion in the multiyear plan, or without a law to authorize such inclusion, under penalty of crime of responsibility.

Paragraph 2. Special and extraordinary credits shall be in force in the fiscal year in which they are authorized, unless the authorization act is enacted during the last four months of that fiscal year, in which case, reopened within the limits of their balances, such credits shall be incorporated into the budget of the subsequent fiscal year.

Paragraph 3. The opening of extraordinary credit may only be allowed to meet unforeseeable and urgent expenses, such as those resulting from war, internal commotion or public calamity, while observing the provisions in Article 62.

Paragraph 4. It is permitted to bind proper revenues generated by the taxes referred to in Articles 155 and 156, and the funds mentioned in Articles 157, 158 and 159, items I, subitems *a* and *b*, and II, to the granting of a guarantee or a collateral to the Union, and to the payment of debits owed to the same. (CA 3, 1993)

Paragraph 5. Reassigning, reallocating, or transferring funds from one programming category to another may eventually be permitted, within science, technology, and innovation activities, with a view to enabling the outcomes of projects restricted to these functions, by means of an act of the executive branch, regardless of the prior legislative authorization set forth in item VI of this Article. (CA 85, 2015)

Article 168. Funds corresponding to budget allocations, including supplementary and special credits, intended for the bodies of the Legislative and Judicial branches, the Prosecution Office, and the Office of the Public Defender, shall be remitted to them by the twentieth of each month, in twelfths, as provided by the supplementary law referred to in Article 165, paragraph 9. (CA 45, 2004)

Article 169. Expenditures on active and retired personnel of the Union, states, the Federal District and municipalities may not exceed the limits established by supplementary law.

Paragraph 1. Granting any advantage or increase in remuneration, creation of posts, positions or functions, or alteration of career structures, as well as admission or hiring of personnel, on any account, by government bodies and entities, or entities owned by the government, including foundations instituted and maintained by the government, may only happen: (Numbered paragraph 1 by CA 19, 1998)

I – if there is a prior budget allocation sufficient to cover the estimated personnel expenditure and the increases resulting therefrom; (CA 19, 1998)

II – if there is a specific authorization in the budget directives law, except for state-owned and mixed-capital companies. (CA 19, 1998)

Paragraph 2. Once the time limit established in the supplementary law referred to in this Article for the adaptation of the standards therein stipulated has run, all remittances of federal or state funds to the states, Federal District, and municipalities which do not obey the referred limits shall be immediately suspended. (CA 19, 1998)

Paragraph 3. For compliance with the limits established as the basis of this Article, during the period stipulated in the supplementary law referred to in the head provision, the Union, states, Federal District, and municipalities shall adopt the following measures: (CA 19, 1998)

I – reduction of at least twenty percent in expenditures with commission offices and positions of trust; (CA 19, 1998)

II – discharge of non-tenured civil servants. (CA 19, 1998)

Paragraph 4. If the measures adopted according to the preceding paragraph are insufficient to guarantee compliance with the provision of the supplementary law referred to in this Article, tenured servants may be dismissed, provided that a regulatory act grounded by each of the Branches specifies the activity, the body, or the administrative unit where reduction of personnel must be carried out. (CA 19, 1998)

Paragraph 5. A servant who is dismissed according to the preceding paragraph shall be entitled to compensation equivalent to one month of remuneration per year of service. (CA 19, 1998)

Paragraph 6. The post affected by the reduction mentioned in the preceding paragraphs shall be considered as no longer existing, and the creation of a post, position, or function with equal or similar duties shall be forbidden for a period of four years. (CA 19, 1998)

Paragraph 7. A federal act shall provide for the general rules to be complied with in carrying out the provision of paragraph 4. (CA 19, 1998)

TITLE VII

The Economic and Financial Order

CHAPTER I

The General Principles of the Economic Activity

Article 170. The economic order, founded on the appreciation of the value of human labor and on free enterprise, is intended to ensure everyone a dignified existence, according to the imperative of social justice, with due regard for the following principles:

I – national sovereignty;

II – private property;

III – social function of property;

IV – free competition;

V – consumer protection;

VI – environment protection, which may include differentiated treatment in accordance with the environmental impact of goods and services and of their respective production and delivery processes; (CA 42, 2003)

VII – reduction in regional and social inequalities;

VIII – pursuit of full employment;

IX – preferential treatment for small-sized enterprises organized under Brazilian law and having their headquarter and management in Brazil. (CA 6, 1995)

Single paragraph. Free exercise of any economic activity is ensured to everyone, regardless of authorization from government bodies, except in the cases set forth by law.

Article 171. (Repealed) (CA 6, 1995)

Article 172. Based on national interests, the law shall regulate the foreign capital investments, encourage reinvestments and regulate the remittance of profits.

Article 173. Except for the cases set forth in this Constitution, direct exploitation of an economic activity by the State shall only be allowed when required by imperatives of national security or by relevant collective interest, as defined by law.

Paragraph 1. The law shall establish the legal system for state-owned companies, mixed-capital companies and their subsidiaries that engage in economic activities of production or trading of goods, or with the provision of services, by establishing: (CA 19, 1998)

I – their social function and the forms of supervision by the State and by society; (CA 19, 1998)

II – compliance with the specific legal system governing private companies, including civil, commercial, labor, and tax rights and liabilities; (CA 19, 1998)

III – competitive bidding and contracting of works, services, purchases, and disposal of assets, with due regard for the principles of government services; (CA 19, 1998)

IV – establishment and operation of boards of directors and of boards of supervisors, with participation of minority shareholders; (CA 19, 1998)

V – terms of office, performance evaluation, and liability of administrators. (CA 19, 1998)

Paragraph 2. State-owned and mixed-capital companies may not enjoy fiscal privileges which are not extended to companies of the private sector.

Paragraph 3. The law shall regulate the relation of state-owned companies with the State and the society.

Paragraph 4. The law shall repress the abuse of economic power aiming at dominating markets, eliminating competition and increasing profits arbitrarily.

Paragraph 5. Without prejudice to the personal liability of the managing officers of a legal entity, the law shall provide the liability of the latter, subjecting it to punishments compatible with the nature of the acts that contravene the economic and financial order and the popular economy.

Article 174. As the normative and regulating agent of the economic activity, the State shall perform, in the manner set forth by law, the functions of supervision, incentive and planning, the latter being binding for the public sector and indicative for the private sector.

Paragraph 1. The law shall establish the guidelines and bases for planning of the balanced national development, which shall incorporate and make compatible the national and regional development plans.

Paragraph 2. The law shall support and encourage cooperative activity and other forms of association.

Paragraph 3. The State shall favor the organization of placer mining activity in cooperatives, considering the protection of the environment and the social-economic development of the placer miners.

Paragraph 4. The cooperatives referred to in the preceding paragraph shall have priority in obtaining authorization or grants for prospecting and mining of placer resources and deposits in the areas where they are operating and in those established in accordance with Article 21, XXV, as set forth by law.

Article 175. The government is responsible for providing public utility services, either directly or by concession or permission, always through a bidding process, as established by law.

Single paragraph. The law shall provide for:

I – the operating rules for companies holding public service concession or permission, the special nature of their contract and of the extension thereof, as well as conditions of forfeiture, supervision and termination of concession or permission;

II – rights of users;

III – tariff policy;

IV – the obligation of maintaining adequate service.

Article 176. Mineral deposits, under exploitation or not, and other mineral resources and the hydraulic energy potentials form, for the purpose of exploitation or use, a property separate from that of the ground and belong to the Union, the concessionaire being guaranteed the ownership of the mined product.

Paragraph 1. The prospecting and mining of mineral resources and the utilization of the potentials mentioned in the head provision may only take place with authorization or concession by the Union, in the national interest, by Brazilians or by a company organized under Brazilian law and having its headquarters and management in Brazil, in the manner set forth by law, which law shall establish specific conditions when such activities are to be conducted in the boundary zone or on indigenous lands. (CA 6, 1995)

Paragraph 2. The owner of the land is ensured participation in the results of the mining operation, in the manner and amount as the law shall establish.

Paragraph 3. Authorization for prospecting shall always be for a set period and the authorization and concession set forth in this Article may not be assigned or transferred, either in full or in part, without the prior consent of the conceding authority.

Paragraph 4. Exploitation of a renewable energy potential of small capacity shall not require an authorization or concession.

Article 177. The Union has a monopoly on the following:

I – prospecting and exploitation of deposits of petroleum; natural gas and of other fluid hydrocarbons;

II – refining of domestic or foreign petroleum;

III – import and export of the products and basic by-products resulting from the activities set forth in the preceding items;

IV – ocean transportation of crude petroleum of domestic origin or of basic petroleum by products produced in the country, as well as pipeline transportation of crude petroleum, its by-products and natural gas of any origin;

V – prospecting, mining, enrichment, reprocessing, industrialization, and trading of nuclear mineral ores and minerals and their by-products, with the exception of radioisotopes whose production, sale, and use may be authorized under permission, in accordance with subitems *b* and *c* of item XXIII of the heading of Article 21 of this Federal Constitution. (CA 49, 2006)

Paragraph 1. The Union may contract with a state-owned company or a private enterprise to perform the activities provided for in items I through IV of this Article, with due regard for the conditions set forth by law. (CA 9, 1995)

Paragraph 2. The law referred to in paragraph 1 shall provide for: (CA 9, 1995)

I – guarantee of supply of petroleum products in the entire national territory; (CA 9, 1995)

II – the conditions of contracting; (CA 9, 1995)

III – the structure and duties of the regulatory body of the monopoly of the Union. (CA 9, 1995)

Paragraph 3. The law shall provide for the transportation and use of radioactive materials within the national territory. (CA 9, 1995)

Paragraph 4. The law which institutes a social contribution for intervention in the economic domain regarding activities of importation or sale of petroleum and petroleum products, natural gas and its by-products, and fuel alcohol shall include the following requirements: (CA 33, 2001)

I – the contribution rate may be: (CA 33, 2001)

a) different for each product or use; (CA 33, 2001)

b) lowered and restored to its original level by an act of the Executive branch, and the provision of Article 150, item III, subitem *b*, shall not apply thereto; (CA 33, 2001)

II – the proceeds from the collection of the contribution shall be allocated: (CA 33, 2001)

a) to the payment of price or transportation subsidies for fuel alcohol, natural gas and its by-products, and petroleum products; (CA 33, 2001)

b) to the financing of environmental projects related to the petroleum and gas industry; (CA 33, 2001)

c) to the financing of transportation infrastructure programs. (CA 33, 2001)

Article 178. The law shall provide for the regulation of air, water and ground transportation, and it shall, in respect to the regulation of international transportation, comply with the agreements entered by the Union, with due regard to the principle of reciprocity. (CA 7, 1995)

Single paragraph. In regulating water transportation, the law shall set forth the conditions in which the transportation of goods in coastal and internal navigational waterways will be permitted to foreign vessels. (CA 7, 1995)

Article 179. The Union, states, Federal District and municipalities shall grant micro-enterprises and small-sized enterprises, as defined by law, differentiated legal treatment, seeking to develop them through simplification of their administration, tax, social security and credit obligations or through elimination or reduction thereof by means of law.

Article 180. The Union, states, Federal District and municipalities shall promote and foster tourism as a factor of social and economic development.

Article 181. Compliance with request for a document or for information of commercial nature, made by a foreign administrative or judicial authority to an individual or legal entity residing or domiciled in the country shall depend upon authorization from the competent authority.

CHAPTER II

Urban Policy

Article 182. The urban development policy carried out by the municipal government, according to general guidelines set forth by law, is aimed at ordaining the full development of the social functions of cities and ensuring the wellbeing of its inhabitants.

Paragraph 1. The master plan, approved by the municipal council, which is compulsory for cities with over twenty thousand inhabitants, is the basic tool of the urban development and expansion policy.

Paragraph 2. Urban property performs its social function when it meets the fundamental requirements for the arrangement of the city as set forth in the master plan.

Paragraph 3. Expropriation of urban property shall be implemented subject to prior and fair compensation in cash.

Paragraph 4. The municipal government may, by means of a specific law, for an area included in the master plan, demand, according to federal law, that the owner of undeveloped, underused or unused urban land provide for adequate use thereof, subject, successively, to:

I – compulsory parceling or construction;

II – rates of urban property and land tax that are progressive in time;

III – expropriation with payment in public debt bonds issued with the prior approval of the Federal Senate, redeemable within up to ten years, in equal and successive annual instalments, ensuring the real value of the compensation and the legal interest.

Article 183. People who possess an urban area of up to two hundred and fifty square meters, for five years, without interruption or opposition, using it as theirs or as their family's home, shall acquire domain of it, provided that they do not own any other urban or rural property.

Paragraph 1. The title of ownership and concession of use shall be granted to the man or woman, or both, regardless of their marital status.

Paragraph 2. This right shall not be recognized for the same holder more than once.

Paragraph 3. Public real estate shall not be acquired by prescription.

CHAPTER III

Agricultural and Land Policy and Agrarian Reform

Article 184. The Union has the power to expropriate on account of social interest, for purposes of agrarian reform, the rural property which is not performing its social function, against prior and fair compensation in agrarian debt bonds with a clause providing for maintenance of the real value, redeemable within a period of up to twenty years computed from the second year of issue, and the use of which shall be defined in the law.

Paragraph 1. Useful and necessary improvements shall be compensated in cash.

Paragraph 2. The decree declaring the property as being of social interest for agrarian reform purposes authorizes the Union to start expropriation action.

Paragraph 3. A supplementary law shall establish a special summary adversary proceeding for expropriation action.

Paragraph 4. The budget shall determine each year the total volume of agrarian debt bonds, as well as the total amount of funds to meet the agrarian reform program in the fiscal year.

Paragraph 5. The transactions of transfer of property expropriated for agrarian reform purposes are exempt from federal, state and municipal taxes.

Article 185. Expropriation of the following for agrarian reform purposes is not permitted:

I – small and medium-size rural property, as defined by law, provided its owner does not own other property;

II – productive property.

Single paragraph. The law shall guarantee special treatment for the productive property and shall establish rules for the fulfilment of the requirements regarding its social function.

Article 186. The social function is met when the rural property complies simultaneously with, according to the criteria and standards prescribed by law, the following requirements:

I – rational and adequate use;

II – adequate use of available natural resources and preservation of the environment;

III – compliance with the provisions that regulate labor relations;

IV – exploitation that favors the well-being of the owners and laborers.

Article 187. The agricultural policy shall be planned and carried out as established by law, with the effective participation of the production sector, comprising producers and rural workers, as well as the marketing, storage and transportation sectors, with especial consideration for:

I – the credit and fiscal mechanisms;

II – prices compatible with production costs and the guarantee of commercialization;

III – research and technology incentives;

IV – technical assistance and rural extension;

V – agricultural insurance;

VI – cooperative activities;

VII – rural electricity and irrigation systems;

VIII – housing for the rural workers.

Paragraph 1. Agricultural planning includes agroindustrial, stock raising, fishing and forestry activities.

Paragraph 2. Agricultural policy and agrarian reform actions shall be made compatible.

Article 188. The destination given to public and unoccupied lands shall be made compatible with the agricultural policy and the national agrarian reform plan.

Paragraph 1. The alienation or concession in any way of public lands with an area of more than two thousand and five hundred hectares to a person or legal entity, even if through an intermediary, shall depend on the prior approval of the National Congress.

Paragraph 2. Alienations or concessions of public lands for agrarian reform purposes are excluded from the provisions of the preceding paragraph.

Article 189. The beneficiaries of distribution of rural land through agrarian reform shall receive ownership titles or concession of use which may not be transacted for a period of ten years.

Single paragraph. The ownership title and the concession of use shall be granted to the man or the woman, or to both, irrespective of their marital status, according to the terms and conditions set forth by law.

Article 190. The law shall regulate and limit the acquisition or lease of rural property by a foreign individual or legal entity and shall establish the cases that shall depend on authorization by the National Congress.

Article 191. People who, not being the owner of rural or urban property, holds as their own, for five uninterrupted years, without opposition, an area of land in the rural zone, not exceeding fifty hectares, making it productive with their labor or that of their family, and having their dwelling thereon, shall acquire ownership of the land.

Single paragraph. The public real estate shall not be acquired by prescription.

CHAPTER IV
The National Financial System

Article 192. The national financial system, structured to promote the balanced development of the country and to serve the collective interests, in all of the component elements of the system, including credit cooperatives, shall be regulated by supplementary laws which shall also provide for the participation of foreign capital in the institutions that make up the above-mentioned system. (CA 40, 2003)

I – (Repealed) (CA 40, 2003)

II – (Repealed) (CA 40, 2003)

II – (Repealed) (CA 40, 2003)

a) (Repealed) (CA 40, 2003)

b) (Repealed) (CA 40, 2003)

IV – (Repealed) (CA 40, 2003)

V – (Repealed) (CA 40, 2003)

VI – (Repealed) (CA 40, 2003)

VII – (Repealed) (CA 40, 2003)

VIII – (Repealed) (CA 40, 2003)

Paragraph 1. (Repealed) (CA 40, 2003)

Paragraph 2. (Repealed) (CA 40, 2003)

Paragraph 3. (Repealed) (CA 40, 2003)

TITLE VIII
The Social Order

CHAPTER I
General Provision

Article 193. The social order is based on the primacy of labor and aimed at social well-being and justice.

CHAPTER II
Social Welfare

SECTION I
General Provisions

Article 194. Social welfare comprises an integrated series of actions initiated by the government and by society, with the purpose of ensuring the rights to health, social security and assistance.

Single paragraph. The government is responsible for organizing social welfare, as provided by law, based on the following objectives:

I – universality of coverage and service;

II – uniformity and equivalence of benefits and services for urban and rural populations;

III – selectivity and distributiveness in the provision of benefits and services;

IV – irreducibility of the value of the benefits;

V – equitable participation in funding;

VI – diversity in the basis of financing, identifying, in specific accounting items for each area, the revenues and expenses linked to health, pension and social assistance actions, preserving the contributory basis of social security; (CA 103, 2019)

VII – democratic and decentralized character of administration, by means of a quadripartite management system, with the participation of workers, employers, retirees, and the government in the collegiate bodies. (CA 20, 1998)

Article 195. Social welfare shall be financed by the entire society, directly or indirectly, as provided by law, with funds from the budgets of the Union, states, Federal District and municipalities and from the following social contributions:

I – of employers, companies, and entities defined by law as being comparable to companies, assessed on: (CA 20, 1998)

a) the payroll and other labor earnings paid or credited, on any account, to people who provide services to them, even when there is no employment bond; (CA 20, 1998)

b) income or revenues; (CA 20, 1998)

c) profits; (CA 20, 1998)

II – of workers and other people insured by social security, progressive rates may be adopted according to the amount of the contribution salary, no contribution being charged on retirement benefits and pensions granted by the general social security regime; (CA 103, 2019)

III – on the revenues of lotteries;

IV – of importers of goods or services from other countries, or of other parties defined by law as being comparable to such importers. (CA 42, 2003)

Paragraph 1. The revenues of the states, Federal District and municipalities allocated to social welfare shall be included in the respective budgets, not being part of the budget of the Union.

Paragraph 2. The proposal for the social welfare budget shall be elaborated jointly by the government bodies responsible for health, social security and social assistance, in accordance with the goals and priorities established in the budget directives law, ensuring each area of the management of its funds.

Paragraph 3. A legal entity indebted to the social welfare system, as established in law, may not contract with the government nor receive benefits or fiscal or credit incentives therefrom.

Paragraph 4. The law may establish other sources intended to guarantee the maintenance or expansion of social welfare, with due regard to the provisions of Article 154, item I.

Paragraph 5. No social welfare benefit or service may be created, increased or extended without a corresponding source of full funding.

Paragraph 6. The social contributions referred to in this Article may only be collected ninety days after the publication of the law which instituted or modified them, the provisions of Article 150, item III, subitem *b*, not applying thereto.

Paragraph 7. Beneficent entities of social assistance which meet the requirements established by law shall be exempt from contribution to social welfare.

Paragraph 8. Rural producers, sharecroppers, tenant farmers, and self-employed fishermen, as well as their spouses, who exercise their activities within a household system and without permanent employees shall contribute to social welfare by applying a rate to the proceeds from the sale of their production and shall be entitled to the benefits provided by law. (CA 20, 1998)

Paragraph 9. The social contributions set forth in item I of the head of this Article may have differentiated rates according to the economic activity, the intensive use of labor, the size of the company, or the structural situation of the labor market; differentiated tax bases may be adopted only in the case of subitem *b* and *c* of item I of the heading. (CA 103, 2019)

Paragraph 10. The law shall define the criteria for the transfer of funds allocated to the unified health system and for social assistance initiatives, from the Union to the states, to the Federal District, and to the municipalities, and from the states to the municipalities, with due regard for the respective transfer of funds. (CA 20, 1998)

Paragraph 11. It is forbidden to grant moratorium and payments on instalment basis for over sixty (60) months and, according to supplementary law, the remission and pardon of social contributions referred to in subitem *a* of item I and item II of the head of the Article. (CA 103, 2019)

Paragraph 12. The law shall define the sectors of the economic activity for which the contributions provided in items I, subitem *b*; and IV of the heading, shall be noncumulative. (CA 42, 2003)

Paragraph 13. (Repealed) (CA 103, 2019)

Paragraph 14. The insured shall only incorporate the contribution period to the general social security regime the month which the contribution is equal to or greater than the minimum monthly contribution required for its category, the grouping of contributions being ensured. (CA 103, 2019)

SECTION II
Health

Article 196. Health is a right of all and a duty of the State and shall be guaranteed by means of social and economic policies aimed at reducing the risk of illness and other hazards and at the universal and equal access to actions and services for its promotion, protection and recovery.

Article 197. Health actions and services are of public importance, and the government is responsible, according to the law, for providing their regulation, supervision and control, and they shall be carried out directly or by third parties and also by people or private legal entities.

Article 198. Health actions and public services integrate a regionalized and hierarchical network and constitute a single system, organized according to the following directives:

I – decentralization, with a single management in each sphere of government;

II – full service, priority being given to preventive activities, without prejudice to assistance services;

III – participation of the community.

Paragraph 1. The unified health system shall be financed, as set forth in Article 195, with funds from the social welfare budget of the Union, states, Federal District and municipalities, as well as from other sources. (CA 29, 2000)

Paragraph 2. The Union, states, Federal District, and municipalities shall provide annually to public health activities and services a minimum amount of funds from the application of percentages calculated upon the following: (CA 29, 2000)

I – in the case of the Union, the net current revenue from the respective fiscal year, which may not be lower than fifteen percent (15%); (CA 86, 2015)

II – in the case of the states and of the Federal District, the proceeds from the tax collection mentioned in Article 155 and of the funds mentioned in Articles 157 and 159, items I, subitem *a*, and II, after deducting the portions remitted to the respective municipalities; (CA 29, 2000)

III – in the case of the municipalities and of the Federal District, the proceeds from the tax collection mentioned in Article 156 and of the funds mentioned in Articles 158 and 159, item I, subitem *b*, and paragraph 3. (CA 29, 2000)

Paragraph 3. A supplementary law to be revised at least every five years shall establish: (CA 29, 2000)

I – the percentages referred to in items II and III of paragraph 2; (CA 86, 2015)

II – the criteria for sharing the resources of the Union earmarked to health and assigned to the states, the Federal District, and the municipalities, and of funds of the states assigned to their respective municipalities, with a view to a progressive reduction of regional disparities; (CA 29, 2000)

III – the rules for supervision, assessment, and control of expenditures on health at the level of the Union, the states, the Federal District, and the municipalities. (CA 29, 2000)

IV – (Repealed) (CA 86, 2015)

Paragraph 4. The local managers of the unified health system may hire community health workers and endemic disease control agents by means of a public selection process, considering the nature and complexity of their duties and the specific requirements of their activity. (CA 51, 2006)

Paragraph 5. Federal legislation shall provide for the legal regime, a nationwide professional minimum salary, the guidelines for career schemes, and the regulation of activities of community health workers and endemic disease control agents, and the federal government shall be responsible, as established by law, to provide supplementary financial support to the states, the Federal District, and municipalities, to achieve compliance with the minimum salary referred to. (CA 63, 2010)

Paragraph 6. In addition to the cases set forth in paragraph 1 of Article 41 and in paragraph 4 of Article 169 of the Federal Constitution, employees whose activities are equivalent to those of a community health worker or an endemic disease control agent may be dismissed if they do not comply with the specific requirements stipulated by law for such activities. (CA 51, 2006)

Article 199. Health assistance is open to private enterprise.

Paragraph 1. Private institutions may participate in a supplementary manner in the unified health system, in accordance with the directives established by the latter, by means of public law contracts or agreements, preference being given to philanthropic and non-profit entities.

Paragraph 2. The allocation of public funds to aid or subsidize profit-oriented private institutions is forbidden.

Paragraph 3. Direct or indirect participation of foreign companies or capital in health assistance in the country is forbidden, except in cases provided by law.

Paragraph 4. The law shall provide for the conditions and requirements which facilitate the removal of organs, tissues and human substances for the purpose of transplants,

research and treatment, as well as the collection, processing and transfusion of blood and its byproducts, all kinds of sale being forbidden.

Article 200. The unified health system has the responsibility to, in addition to other duties, as provided by law:

I – control and supervise proceedings, products and substances of interest to health and to participating in the production of drugs, equipment, immunobiological products, blood products and other inputs;

II – carry out actions of sanitary and epidemiologic vigilance as well as those related to the health of workers;

III – organize the training of personnel in the area of health;

IV – participate in the definition of the policy and executing basic sanitation actions;

V – foster, within its scope of action, scientific and technological development, as well as innovation; (CA 85, 2015)

VI – supervise and inspect foodstuffs, including their nutritional contents, as well as drinks and water for human consumption;

VII – participate in the supervision and control of the production, transportation, storage and use of psychoactive, toxic and radioactive substances and products;

VIII – cooperate in the preservation of the environment, including that of the workplace.

SECTION III

Social Security

Article 201. The social security system shall be organized in the form of a general social security regime, based on contributions and mandatory affiliation, with due regard for criteria that preserve financial and actuarial balance, and shall provide for, in accordance with the law: (CA 103, 2019)

I – coverage resulting from the events of temporary or permanent disability due to work and old age; (CA 103, 2019)

II – maternity protection, especially for pregnant women; (CA 20, 1998)

III – protection to workers in situations of involuntary unemployment; (CA 20, 1998)

IV – family allowance and imprisonment allowance for dependents of insured people with low-income; (CA 20, 1998)

V – pension for death of an insured, man or woman, to the spouse or companion, and dependents, complying with the provision of paragraph 2. (CA 20, 1998)

Paragraph 1. Adoption of differentiated requirements or criteria for the concession of benefits is forbidden, except for, as defined by a supplementary law, the possibility to determine age and period of contribution other than the general rule for granting retirement exclusively in favor of the beneficiaries: (CA 103, 2019)

I – with disabilities, previously submitted to biopsychosocial evaluation performed by a multidisciplinary and interdisciplinary team; (CA 103, 2019)

II – whose activities are performed with effective exposure to chemical, physical and biological agents harmful to health, or association of these agents; the characterization by professional or occupation category, in that case, is forbidden. (CA 103, 2019)

Paragraph 2. No benefit which replaces the contribution salary or labor earnings of the beneficiaries shall have a monthly amount lower than the minimum monthly wage. (CA 20, 1998)

Paragraph 3. All contribution salaries included in the calculation of the benefit shall be duly updated, as provided by law. (CA 20, 1998)

Paragraph 4. Readjustment of the benefits is ensured, so that their real value is permanently maintained, in accordance with criteria defined by law. (CA 20, 1998)

Paragraph 5. Participation in the general social security regime, in the position of an optional beneficiary, is forbidden for a person who participates in a special social security scheme. (CA 20, 1998)

Paragraph 6. The Christmas bonus for retirees and pensioners shall be based on the amount of the earnings in the month of December of each year. (CA 20, 1998)

Paragraph 7. Retirement is ensured under the general social security regime, in accordance with the law, upon compliance with the following conditions: (CA 20, 1998)

I – 65 (sixty-five) years old, if male, and 62 (sixty-two) years old, if female, subject to minimum period of contribution; (CA 103, 2019)

II – 60 (sixty) years old, if male and 55 (fifty-five) years old, if female, for rural workers and for those who carry out their activities in a family economy regime, including the rural producer, the mining prospector and the artisan fisherman. (CA 103, 2019)

Paragraph 8. The age requirement referred to in item I of paragraph 7 shall be reduced by five (5) years for teachers who document effective period performing teaching roles in early childhood and in elementary and secondary education as established by supplementary law. (CA 103, 2019)

Paragraph 9. For retirement purposes, the reciprocal computation period of contribution in the general social security regime and in the special social security schemes, and between them, shall be ensured, in which case the various social security schemes

shall offset each other financially, in accordance with the criteria established by law. (CA 103, 2019)

Paragraph 9-A. The period of military active duty in the activities referred to in Articles 42, 142 and 143 and the period of contribution to the general social security regime or the special social security scheme will have reciprocal computation for purposes of military inactiveness or retirement, in which case military contribution income and contribution income to other schemes shall offset each other financially. (CA 103, 2019)

Paragraph 10. A supplementary law may regulate the coverage of unscheduled benefits, including those stemming from occupation injuries, which the general social security regime and the private sector will provide concurrently. (CA 103, 2019)

Paragraph 11. The amounts regularly earned by employees, on any account, shall be incorporated into their monthly salary for purposes of social security contribution and the resulting effects on benefits, in the cases and in the manner provided by law. (CA 20, 1998)

Paragraph 12. The law shall provide for a special system of social security, with differentiated rates, to include low-income workers, including those who are in an informal situation, and those without their own income who are engaged exclusively in household chores within their own homes, as long as they belong to low-income families. (CA 103, 2019)

Paragraph 13. The retirement benefit granted to the insured person referred to in paragraph 12 shall amount to 1 (one) nationally unified minimum monthly wage. (CA 103, 2019)

Paragraph 14. The methods of computation of fictitious periods of contribution for the purposes of granting social security benefits and reciprocal computation are forbidden. (CA 103, 2019)

Paragraph 15. A supplementary law will establish prohibitions, rules and conditions to accumulate social security benefits. (CA 103, 2019)

Paragraph 16. Employees of public consortia, state-owned and mixed-capital companies and their subsidiaries shall be compulsorily retired, subject to the compliance with the minimum period of contribution, upon reaching the maximum age referred to in item II of paragraph 1 of Article 40, as provided by law. (CA 103, 2019)

Article 202. The private social security scheme, of a complementary nature and organized on an autonomous basis as regards the general social security regime, shall be optional, based on the formation of reserves which guarantee the contracted benefit, and regulated by a supplementary law. (CA 20, 1998)

Paragraph 1. The supplementary law referred to in this Article shall ensure that the participant in benefit plans of private pension plan companies is provided with full access to information regarding the management of their respective plans. (CA 20, 1998)

Paragraph 2. The contributions of employers, the benefits, and the terms of contracts set forth in the by-laws, regulations, and benefit plans of the private pension plan companies are neither an integral part of the employment contract of participants, nor, with the exception of the benefits granted, an integral part of the remuneration of participants, as provided by law. (CA 20, 1998)

Paragraph 3. The Union, states, Federal District, and municipalities, their agencies, foundations, companies, mixed-capital companies, and other public entities are forbidden to contribute funds to private pension plan companies, except in the position of sponsors, in which case their standard contribution may not, under any circumstances, exceed that of the beneficiary. (CA 20, 1998)

Paragraph 4. A supplementary law shall regulate the relation between the Union, states, Federal District, or municipalities, including their agencies, foundations, mixed-capital companies, and companies controlled either directly or indirectly, in the position of sponsors of pension plans companies, and the supplementary private pension entities. (CA 103, 2019)

Paragraph 5. The supplementary law referred to in paragraph 4 shall apply, insofar as pertinent, to private companies holding a permission or concession to render public services, when such companies sponsor benefit plans in supplementary private pension entities. (CA 103, 2019)

Paragraph 6. A supplementary law shall establish the requirements for the appointment of board members of the closed private pension plan companies founded by the sponsors referred to in paragraph 4 and shall regulate the inclusion of participants in the collegiate bodies and decision-making bodies in which their interest are subject to discussion and decision. (CA 103, 2019)

SECTION IV

Social Assistance

Article 203. Social assistance shall be rendered to whomever may need it, regardless of contribution to social welfare and shall have as objectives:

I – the protection of the family, maternity, childhood, adolescence and old age;

II – the assistance to needy children and adolescents;

III – the promotion of the integration into the labor force;

IV – the habilitation and rehabilitation of the handicapped and their integration into community;

V – the guarantee of a monthly benefit of one minimum wage to handicapped people and to the elderly who prove they do not have the means to provide for their own support or having it provided by their families, as set forth by law.

Article 204. Government actions in the area of social assistance shall be implemented with funds from the social welfare budget, as provided for in Article 195, in addition to other sources, and organized based on the following directives:

I – political and administrative decentralization, the coordination and the general rules being incumbent upon the federal level, and the coordination and implementation of the respective programs, upon the state and municipal leves, as well as upon beneficent and social assistance entities;

II – participation of the population, by means of organizations representing them in the formulation of policies and in the control of actions taken at all levels.

Single paragraph. The states and the Federal District may earmark up to five tenths percent of their net tax revenues to programs of social inclusion and promotion, the utilization of such funds for the payment of the following items being forbidden: (CA 42, 2003)

I – personnel expenses and social security contributions; (CA 42, 2003)

II – debt servicing; (CA 42, 2003)

III – any other current expense not directly related to the investments or actions supported by the programs referred to. (CA 42, 2003)

CHAPTER III
Education, Culture and Sports

SECTION I
Education

Article 205. Education, which is the right of all and duty of the State and of the family, shall be promoted and fostered with the cooperation of society, with a view to the full development of people, their preparation for the exercise of citizenship and their qualification for work.

Article 206. Education shall be provided based on the following principles:

I – equal conditions of access and permanence in school;

II – freedom to learn, teach, research and express thought, art and knowledge;

III – pluralism of pedagogic ideas and conceptions and coexistence of public and private teaching institutions;

IV – free public education in official schools;

V – valorization of school education professionals, guaranteeing, in accordance with the law, career schemes for public school teachers, with admittance exclusively by means of competitive civil-servant examinations consisting of tests and presentation of academic and professional credentials; (CA 53, 2006)

VI – democratic administration of public education, in the manner prescribed by law;

VII – guarantee of standards of quality;

VIII – a nationwide professional minimum salary for public school teachers, as provided by federal law. (CA 53, 2006)

Single paragraph. The law shall provide for the classes of workers to be considered basic education professionals, as well as for the deadline for the preparation or adaptation of their career schemes, within the federal government, states, Federal District, and municipalities. (CA 53, 2006)

Article 207. Universities shall have didactic, scientific, administrative, financial and property management autonomy and shall comply with the principle of inseparability of teaching, research and extension activities.

Paragraph 1. Universities are permitted to hire foreign professors, technicians and scientists as provided by law. (CA 11, 1996)

Paragraph 2. The provisions of this Article apply to scientific and technological research institutions. (CA 11, 1996)

Article 208. The duty of the State towards education shall be fulfilled by ensuring the following:

I – mandatory basic education, free of charge, for everyone from 4 (four) to 17 (seventeen) years of age, including assurance that it will be offered gratuitously for all who did not have access to it at the proper age; (CA 59, 2009)

II – progressive universalization of free of charge secondary school education; (CA 14, 1996)

III – specialized schooling for handicapped people, preferably within the regular school system;

IV – early childhood education to children of up to 5 (five) years of age in day-care centers and pre-schools; (CA 53, 2006)

V – access to higher levels of education, research and artistic creation according to personal capacity;

VI – provision of regular night courses adequate to the conditions of the student;

VII – assistance to students in all grades of basic education, by means of supplementary programs providing school materials, transportation, food, and health care. (CA 59, 2009)

Paragraph 1. The access to compulsory and free education is a subjective public right.

Paragraph 2. In case the compulsory education is not provided, or it is not regularly provided by the government, the competent authority shall be held liable.

Paragraph 3. The government has the power to take a census of elementary school students, call them for enrollment and ensure that parents or guardians guarantee that their children attend school.

Article 209. Teaching is open to private enterprise, provided that the following conditions are met:

I – compliance with the general rules of national education;

I – authorization and evaluation of quality by the government.

Article 210. Minimum curricula shall be established for elementary schools in order to ensure a common basic education and respect for national and regional cultural and artistic values.

Paragraph 1. The teaching of religion is optional and shall be offered during the regular school hours of public elementary schools.

Paragraph 2. Regular elementary education shall be given in the Portuguese language and indigenous communities shall also be ensured the use of their native tongues and their own learning methods.

Article 211. The Union, states, Federal District, and municipalities shall cooperate in the organization of their educational systems.

Paragraph 1. The Union shall organize the federal educational system and that of the territories, finance the federal public educational institutions and exercise, in educational matters, a redistributive and supplementary function, so as to guarantee the equalization of the educational opportunities and a minimum standard of quality of education, through technical and financial assistance to the states, the Federal District and the municipalities. (CA 14, 1996)

Paragraph 2. Municipalities shall mainly address their actions in education to early childhood and elementary school. (CA 14, 1996)

Paragraph 3. The states and the Federal District shall address their actions in education to elementary and secondary school. (CA 14, 1996)

Paragraph 4. In the organization of the respective educational systems, the federal government, the states, the Federal District, and the municipalities shall establish forms of cooperation, to guarantee the universalization of mandatory education. (CA 59, 2009)

Paragraph 5. Public basic education shall give priority to regular education. (CA 53, 2006)

Article 212. The Union shall apply, annually, never less than eighteen percent, and the states, the Federal District, and the municipalities, at least twenty-five percent of the tax revenues, including those resulting from transfers, in the maintenance and development of education.

Paragraph 1. The share of tax revenues, transferred by the Union to the states, the Federal District and the municipalities, or by the states to the respective municipalities, shall not be considered for purposes of the calculation provided by this Article, as revenues of the government which transfers it.

Paragraph 2. For purposes of compliance with the heading of this Article, the federal, State and municipal educational systems, as well as the funds applied in accordance with Article 213 shall be taken into consideration.

Paragraph 3. In the distribution of public funds, priority shall be given to meeting the needs of compulsory education, with regard to universalization, assurance of quality standards, and equality, as set forth in the national education plan. (CA 59, 2009)

Paragraph 4. The supplementary food and health assistance programs provided by Article 208, item VII, shall be financed with funds derived from social contributions and other budgetary funds.

Paragraph 5. Public basic education shall have, as an additional source of financing, the social contribution levied on employee wages, collected as a payroll tax by companies, as provided by law. (CA 53, 2006)

Paragraph 6. State and municipal quotas of the proceeds from the collection of the social contribution for education shall be distributed in proportion to the number of students enrolled in basic education in the respective public-school systems. (CA 53, 2006)

Article 213. Public funds shall be allocated to public schools, and may be channeled to community, religious or philanthropic schools, as defined by law, which:

I – prove that they do not seek profit and that they apply their surplus funds in education;

II – ensure that their assets shall be assigned to another community, religious or philanthropic schools, or to the government in case they cease their activities.

Paragraph 1. The funds provided by this Article may be allocated to elementary and secondary school scholarships, as provided by law, for those who prove insufficiency of means, when no vacancies or no regular courses are offered in the public school system

of the place where the student lives, the government being placed under the obligation to invest, on a priority basis, in the expansion of the public system of the local community.

Paragraph 2. Research and extension activities, as well as activities aimed at encouraging and fostering innovation, carried out by universities and/or professional and technological education institutions, may receive financial support from the government. (CA 85, 2015)

Article 214. The law shall establish a ten-year national education plan, with a view to organizing the national education system with the cooperation of states and municipalities, as well as to defining implementation directives, objectives, targets, and strategies so as to ensure maintenance and development of teaching, at its various levels, stages, and modalities, by means of integrated federal, state, and municipal government actions leading to: (CA 59, 2009)

I – eradication of illiteracy;

II – universalization of school assistance;

III – improvement of the quality of education;

IV – professional training;

V – humanistic, scientific and technological development of the country;

VI – stipulation of an amount of public funds to be invested in education as a proportion of the gross domestic product. (CA 59, 2009)

SECTION II

Culture

Article 215. The State shall ensure to everyone the full exercise of cultural rights and access to sources of national culture and shall support and foster the appreciation and diffusion of cultural expressions.

Paragraph 1. The State shall protect the expressions of popular, Indian and Afro-Brazilian cultures, as well as those of other groups participating in the national civilization process.

Paragraph 2. The law shall provide for the establishment of commemorative dates of high significance for the various national ethnic segments.

Paragraph 3. The law shall establish the national culture plan, of multiyear duration aimed at the cultural development of the country and the integration of government initiatives to attain the following: (CA 48, 2005)

I – defense and valorization of Brazilian cultural patrimony; (CA 48, 2005)

II – the production, promotion, and diffusion of cultural goods; (CA 48, 2005)

III – training of qualified personnel to manage culture in its multiple dimensions; (CA 48, 2005)

IV – democratization of access to cultural goods; (CA 48, 2005)

V – valorization of ethnic and regional diversity. (CA 48, 2005)

Article 216. Brazilian cultural heritage consists of assets of material and immaterial nature, taken individually or as a whole, which bear reference to the identity, action and memory of the various groups that form Brazilian society, therein included:

I – forms of expression;

II – ways of creating, making and living;

III – scientific, artistic and technological creations;

IV – works, objects, documents, buildings and other spaces intended for artistic and cultural expressions;

V – urban complexes and sites of historical, natural, artistic, archaeological, paleontological, ecological and scientific value.

Paragraph 1. The government shall, with the cooperation of the community, promote and protect Brazilian cultural heritage by inventories, registers, surveillance, monument protection decrees, expropriation and other forms of precaution and preservation.

Paragraph 2. The administration is responsible for managing governmental documents and providing measures to ensure they are available for consultation to whomever may need access to them.

Paragraph 3. The law shall establish incentives for the production and knowledge of cultural assets and values.

Paragraph 4. Damage and threats to the cultural heritage shall be punished in accordance with the law.

Paragraph 5. All documents and sites bearing historical reminiscence to the ancient communities of runaway slaves are protected as national heritage.

Paragraph 6. The states and the Federal District may assign up to five tenths percent of their net tax revenues to a state fund for the promotion of culture, for funding cultural programs and projects; the use of such funds are forbidden to pay the following: (CA 42, 2003)

I – personnel expenses and social security contributions; (CA 42, 2003)

II – debt servicing; (CA 42, 2003)

III – any other current expense not directly related to the investments or actions supported by the programs referred to. (CA 42, 2003)

Article 216-A. The national culture system, organized as a collaborative regime, in a decentralized and participative manner, institutes a process of joint management and promotion of cultural policies, which shall be democratic and permanent, and agreed upon by the federal entities and society, aiming at fostering human, social, and economic development, with full exercise of cultural rights. (CA 71, 2012)

Paragraph 1. The national culture system is founded on the national cultural policy and on its guidelines, established in the national culture plan, and shall obey the following principles: (CA 71, 2012)

I – diversity of cultural expressions; (CA 71, 2012)

II – universal access to cultural goods and services; (CA 71, 2012)

III – promotion of production, diffusion, and circulation of cultural knowledge and goods; (CA 71, 2012)

IV – cooperation among the federal entities, and the public and private agents working in the cultural area; (CA 71, 2012)

V – integration and interaction in the implementation of policies, programs, projects, and actions developed; (CA 71, 2012)

VI – complementary roles for cultural agents; (CA 71, 2012)

VII – crosscutting cultural policies; (CA 71, 2012)

VIII – autonomy for the federal entities and for civil society institutions; (CA 71, 2012)

IX – transparency and sharing of information; (CA 71, 2012)

X – democratized decision-making processes, with social participation and control; (CA 71, 2012)

XI – coordinated and agreed-upon decentralization of management, resources, and actions; (CA 71, 2012)

XII – gradual increase of funds earmarked for culture in public budgets. (CA 71, 2012)

Paragraph 2. The following entities form the structure of the national culture system in the respective levels of the Federation: (CA 71, 2012)

I – cultural managing bodies; (CA 71, 2012)

II– cultural policy boards; (CA 71, 2012)

III – cultural conferences; (CA 71, 2012)

IV – inter-manager committees; (CA 71, 2012)

V – cultural plans; (CA 71, 2012)

VI – cultural funding systems; (CA 71, 2012)

VII – cultural information and indicator systems; (CA 71, 2012)

VIII – training programs in the area of culture; and (CA 71, 2012)

IX – sectoral culture systems. (CA 71, 2012)

Paragraph 3. A federal law shall provide for the regulation of the national culture system, as well as of its coordination with other national systems or sectorial policies of the government. (CA 71, 2012)

Paragraph 4. The states, Federal District, and municipalities shall organize their respective cultural systems in their own legislation. (CA 71, 2012)

SECTION III
Sports

Article 217. The State has the duty to foster the practice of formal and informal sports, as a right of each person, with due regard for:

I – the autonomy of the directing sports entities and associations, as to their organization and operation;

II – the allocation of public funds with a view to promoting, on a priority basis, educational sports and, in specific cases, high performance sports;

III – differentiated treatment for professional and nonprofessional sports;

IV – the protection and fostering of sports created in the country.

Paragraph 1. The judicial branch shall only accept legal actions related to sports discipline and competitions after the instances of the athletic courts, as regulated by law, have been exhausted.

Paragraph 2. The athletic courts shall render final decisions within sixty days, at the most, counted from the date of filing of the action.

Paragraph 3. The government shall encourage leisure as a form of social promotion.

CHAPTER IV
Science, Technology, and Innovation (CA 85, 2015)

Article 218. The State shall promote and foster scientific development, research, scientific and technological expertise, as well as innovation. (CA 85, 2015)

Paragraph 1. Basic and technological scientific research shall receive preferential treatment from the State, with a view to public well-being and the progress of science, technology, and innovation. (CA 85, 2015)

Paragraph 2. Technological research shall be directed mainly to the solution of Brazilian problems and to the development of the national and regional productive system.

Paragraph 3. The State shall support the training of human resources in the areas of science, research, technology and innovation, including support for technological extension activities, and shall offer special work means and conditions to those engaged in such activities. (CA 85, 2015)

Paragraph 4. The law shall support and foster the companies which invest in research, creation of technology appropriate for the country, training and improvement of their human resources and those which adopt remuneration systems that ensure employees a share of the economic earnings resulting from the productivity of their work, apart from their salary.

Paragraph 5. The states and the Federal District may allocate a share of their budget revenues to public entities which foster scientific and technological education and research.

Paragraph 6. The State, in the implementation of the activities set forth in the head provision, shall encourage coordination among entities, both public and private, in the various levels of government. (CA 85, 2015)

Paragraph 7. The State shall promote and foster a strong presence abroad of public institutions devoted to science, technology, and innovation, aiming at the implementation of the activities set forth in the head provision. (CA 85, 2015)

Article 219. The domestic market is part of the national patrimony and shall be supported with a view to permitting cultural and socioeconomic development, the wellbeing of the population and the technological autonomy of the country, as set forth in federal law.

Single paragraph. The State shall encourage the development and strengthening of innovation in companies, as well as in other entities, either public or private, the establishment and maintenance of technology parks and hubs and of other environments conducive to innovation, the participation of independent inventors, and the creation, absorption, dissemination, and transfer of technology. (CA 85, 2015)

Article 219-A. The Union, states, Federal District, and municipalities may sign cooperation instruments with government bodies and entities and with private entities, including for the purpose of sharing specialized human resources and installed capacity, aimed at the implementation of research, scientific and technological development, and innovation projects, upon a financial counterpart or non-financial commitment by the beneficiary entity, as provided by law. (CA 85, 2015)

Article 219-B. The National System of Science, Technology and Innovation shall be organized within a framework of cooperation among entities, both public and private,

with a view to promoting scientific and technological development and innovation. (CA 85, 2015)

Paragraph 1. A federal law shall establish the general rules for the national science, technology, and innovation system. (CA 85, 2015)

Paragraph 2. The states, Federal District, and municipalities shall legislate concurrently on their own peculiarities. (CA 85, 2015)

CHAPTER V
Social Communication

Article 220. The manifestation of thought, the creation, the expression and the information, in any form, process or medium shall not be subject to any restriction, with due regard to the provisions of this Constitution.

Paragraph 1. No law shall contain any provision which may represent a hindrance to full freedom of press in any medium of social communication, with due regard to the provisions of Article 5, items IV, V, X, XIII and XIV.

Paragraph 2. Any kind of censorship of a political, ideological and artistic nature is forbidden.

Paragraph 3. It is within the competence of federal laws to:

I – regulate public entertainment and shows, it being the responsibility of the government to inform on their nature, the age for which they are not recommended and places and times unsuitable for their exhibition;

II – establish legal means which enable people and families the possibility of defending themselves against radio and television programs and schedules which go contrary to the provisions of Article 221, as well as against publicity of products, practices and services which may be harmful to health or to the environment.

Paragraph 4. Commercial advertising of tobacco, alcoholic beverages, pesticides, medicines and therapies shall be subject to legal restrictions, in accordance with item II of the preceding paragraph and shall contain, whenever necessary, a warning concerning the harm which may be caused by their use.

Paragraph 5. Social communication media may not, directly or indirectly, be subject to monopoly or oligopoly.

Paragraph 6. The publication of a printed social communication medium shall not depend on license from authorities.

Article 221. The production and programming of radio and television stations shall comply with the following principles:

I – preference to educational, artistic, cultural and informative purposes;

II – promotion of national and regional culture and fostering of independent productions aimed at their diffusion;

III – regional differentiation of cultural, artistic and press production, according to percentages established in law;

IV – respect for the ethical and social values of the person and the family.

Article 222. Newspaper companies, sound broadcasting companies, or sound and image broadcasting companies, shall be owned exclusively by native Brazilians or those naturalized for more than ten years, or by legal entities incorporated under Brazilian laws and headquartered in Brazil. (CA 36, 2002)

Paragraph 1. In all circumstances, at least seventy percent of the total capital stock and of the voting capital of newspaper companies, sound broadcasting companies, or sound and image broadcasting companies, shall be owned directly or indirectly by native Brazilians or those naturalized for more than ten years, who shall mandatorily exercise the management of activities and shall define the content of programming. (CA 36, 2002)

Paragraph 2. Editorial responsibility and the activities regarding selection and management of the programming to be disseminated shall be carried out exclusively by native Brazilians or those naturalized for more than ten years, in any social communication medium. (CA 36, 2002)

Paragraph 3. Electronic social communication media, regardless of the technology used to deliver the service, shall comply with the principles stipulated in Article 221, as provided by specific legislation, which shall also ensure priority to Brazilian professionals in the production of Brazilian programs. (CA 36, 2002)

Paragraph 4. Specific legislation shall regulate the participation of foreign capital in the companies mentioned in paragraph 1. (CA 36, 2002)

Paragraph 5. Changes in the corporate control of the companies mentioned in paragraph 1 must be informed to the National Congress. (CA 36, 2002)

Article 223. The executive branch has the authority to grant and renew concession, permission and authorization for radio broadcasting and sound and image broadcasting services with due regard to the principle of the complementary roles of private, public and State systems.

Paragraph 1. The National Congress shall consider such proposition in the period set forth in Article 64, paragraphs 2 and 4, counted from the date of receipt of the message.

Paragraph 2. The nonrenewal of the concession or permission shall depend on approval by at least two fifths of the National Congress, in nominal voting.

Paragraph 3. The granting or renewal shall only produce legal effects after approval by the National Congress, as set forth in the preceding paragraphs.

Paragraph 4. Cancellation of a concession or permission prior to its expiring date shall depend on a court decision.

Paragraph 5. The term for a concession or permission shall be ten years for radio stations and fifteen years for television channels.

Article 224. For the purposes of the provisions of this chapter, the National Congress shall institute, as an auxiliary agency, the Social Communication Council, in the manner prescribed by law.

CHAPTER VI
Environment

Article 225. Everyone has the right to an ecologically balanced environment, which is an asset of common use and essential to a healthy quality of life, and both government and community shall have the duty to defend and preserve it for present and future generations.

Paragraph 1. To ensure the effectiveness of this right, the government has the responsibility to:

I – preserve and restore the essential ecological processes and provide for the ecological treatment of species and ecosystems;

II – preserve the diversity and integrity of the genetic patrimony of the country and control entities engaged in research and manipulation of genetic material;

III – define, in all federal entities, territorial spaces and their components which are to receive special protection, any alterations and suppressions being allowed only by means of law, and any use which may harm the integrity of the attributes which justify their protection being forbidden;

IV – demand, in the manner prescribed by law, a prior environment impact study, which shall be made public, for the installation of works and activities which may potentially cause significant degradation of the environment;

V – control the production, sale and use of techniques, methods or substances which represent a risk to life, the quality of life and the environment;

VI – promote environmental education in all school levels and public awareness of the need to preserve the environment;

VII – protect the fauna and the flora, with prohibition, in the manner prescribed by law, of all practices which represent a risk to their ecological function, cause the extinction of species or subject animals to cruelty.

Paragraph 2. Those who exploit mineral resources shall be required to restore the degraded environment, in accordance with the technical solutions demanded by the competent government body, as provided by law.

Paragraph 3. Procedures and activities considered as harmful to the environment shall subject the offenders, be they individuals or legal entities, to penal and administrative sanctions, without prejudice to the obligation to repair the damage caused.

Paragraph 4. The Brazilian Amazonian Forest, the Atlantic Forest, the Serra do Mar, the Pantanal Mato-Grossense and the coastal zone are part of the national patrimony, and they shall be used, as provided by law, under conditions which ensure the preservation of the environment, therein included the use of mineral resources.

Paragraph 5. The unoccupied lands or lands seized by the states through legal proceedings that are necessary to protect the natural ecosystems are inalienable.

Paragraph 6. Power plants operated by nuclear reactor shall have their location defined in federal law and may not otherwise be installed.

Paragraph 7. For the purposes of the provision under the final part of item VII of paragraph 1 of this Article, sporting practices with animals shall not be considered cruel so long as they are cultural manifestations, in compliance with paragraph 1 of Article 215 of this Federal Constitution. These activities must be registered as goods of immaterial nature that integrate the Brazilian cultural asset, which ought to be regulated under specific law that ensures the well-being of the animals involved. (CA 96, 2017)

CHAPTER VII

Family, Children, Adolescents, Young People and Senior Citizens (CA 65, 2010)

Article 226. The family, which is the foundation of society, shall enjoy special protection from the State.

Paragraph 1. Marriage is civil and the marriage ceremony is free of charge.

Paragraph 2. Religious marriage has civil effects, in accordance with the law.

Paragraph 3. For purposes of protection by the State, the stable union between a man and a woman is recognized as a family entity, and the law shall facilitate the conversion of such entity into marriage.

Paragraph 4. The community formed by either parent and their descendants is also considered as a family entity.

Paragraph 5. The rights and the duties of marital society shall be exercised equally by the man and the woman.

Paragraph 6. Civil marriage may be dissolved by divorce. (CA 66, 2010)

Paragraph 7. Based on the principles of human dignity and responsible parenthood, family planning is a free choice of the couple, it being within the competence of the State to provide educational and scientific resources for the exercise of this right, any coercion by official or private institutions being forbidden.

Paragraph 8. The State shall ensure assistance to the family in the person of each of its members, creating mechanisms to suppress violence within the family.

Article 227. It is the duty of the family, the society, and the State to ensure children, adolescents, and young people, with absolute priority, the right to life, health, nourishment, education, leisure, professional training, culture, dignity, respect, freedom, and family and community life, as well as to guard them from all forms of negligence, discrimination, exploitation, violence, cruelty, and oppression. (CA 65, 2010)

Paragraph 1. The State shall promote full health assistance programs for children, adolescents, and young people, the participation of nongovernmental entities being allowed, by means of specific policies and with due regard to the following precepts: (CA 65, 2010)

I – allocation of a percentage of public health care funds to mother and child assistance;

II – creation of preventive and specialized care programs for people with physical, sensory, or mental disabilities, as well as programs for the social integration of disabled adolescents and young people, by means of training for a profession and for community life and by means of enhancing access to communal facilities and services, including the elimination of architectural barriers and all forms of discrimination. (CA 65, 2010)

Paragraph 2. The law shall regulate construction standards for public sites and buildings and for the manufacturing of public transportation vehicles, in order to ensure adequate access to the handicapped.

Paragraph 3. The right to special protection shall include the following aspects:

I – minimum age of fourteen years for admission to work, with due regard to the provisions of Article 7, item XXXIII;

II – guarantee of social security and labor rights;

III – guarantee of access to school for adolescent and young workers; (CA 65, 2010)

IV – guarantee of full and formal knowledge of the juvenile offense charges, equal rights in the procedural relation and technical defense by a qualified professional, in accordance with the provisions of the specific protection legislation;

V – compliance with the principles of brevity, exceptionality and respect to the peculiar conditions of the developing person, when applying any measures that restrain freedom;

VI – government fostering, by means of legal assistance, tax incentives and subsidies, as provided by law, of the protection, through guardianship, of orphaned or abandoned children or adolescents;

VII – preventive and specialized care programs for children, adolescents, and young people addicted to narcotics or related drugs. (CA 65, 2010)

Paragraph 4. The law shall severely punish abuse, violence and sexual exploitation of children and adolescents.

Paragraph 5. Adoption shall be assisted by the government, as provided by law, which shall establish cases and conditions for adoption by foreigners.

Paragraph 6. Children born inside or outside wedlock or adopted shall have the same rights and qualifications, any discriminatory designation of their filiations being forbidden.

Paragraph 7. In attending to the rights of children and adolescents, the provisions of Article 204 shall be taken into consideration.

Paragraph 8. The law shall establish: (CA 65, 2010)

I – a young people's statute, for the purpose of regulating young people's rights; (CA 65, 2010)

II – a ten-year national plan for young people, aimed at coordinating the work of the various levels of government in the implementation of public policies. (CA 65, 2010)

Article 228. Minors under eighteen years of age may not be held criminally liable and shall be subject to the rules of the special legislation.

Article 229. Parents have the responsibility to assist, up bring and educate their under age children and adult children have the responsibility to help and assist their parents in old age, need or sickness.

Article 230. The family, society and the State are responsible for assisting senior citizens, ensuring their participation in the community, defending their dignity and well-being and guaranteeing their right to life.

Paragraph 1. Assistance programs for the elderly shall be carried out preferably within their homes.

Paragraph 2. Those over sixty-five years of age are guaranteed free urban public transportation.

CHAPTER VIII

Indigenous People

Article 231. Indigenous people shall have their social organization, customs, languages, creeds and traditions recognized, as well as their original rights to the lands they traditionally occupy. The Union is responsible for demarcating such lands, protecting and ensuring respect for all of their property.

Paragraph 1. Lands traditionally occupied by Indians are those on which they live on a permanent basis, those used for their productive activities, those indispensable to the preservation of the environmental resources necessary for their wellbeing and for their physical and cultural reproduction, according to their habits, customs and traditions.

Paragraph 2. The lands traditionally occupied by indigenous are intended for their permanent possession and they shall have the exclusive usufruct of the riches of the land, the rivers and the lakes existing therein.

Paragraph 3. Water resources, including energetic potentials, may only be exploited, and mineral riches in indigenous land may only be prospected and mined with the authorization of the National Congress, after hearing the communities involved, and the participation in the results of such mining shall be ensured to them, as set forth by law.

Paragraph 4. The lands referred to in this Article are inalienable and nontransferable and the rights thereto are not subject to statute of limitation.

Paragraph 5. The removal of indegenous groups from their lands is forbidden, except *ad referendum* of the National Congress, in case of a catastrophe or an epidemic which represents a risk to their population, or in the interest of the sovereignty of the country, after decision by the National Congress, it being guaranteed that, under any circumstances, the return shall be immediate as soon as the risk ceases.

Paragraph 6. Acts with a view to occupation, domain and possession of the lands referred to in this Article or to the exploitation of the natural riches of the land, rivers and lakes existing therein, are null and void, producing no legal effects, except in case of relevant public interest of the Union, as provided by a supplementary law and such

nullity shall not create a right to indemnity or to sue the Union, except in what concerns improvements derived from occupation in good faith, in the manner prescribed by law.

Paragraph 7. The provisions of Article 174, paragraphs 3 and 4, shall not apply to indigenous lands.

Article 232. The Indians, their communities and organizations have standing under the law to file suits to defend their rights and interests, the Prosecution Office intervening in all the procedural acts.

TITLE IX
General Constitutional Provisions

Article 233. (Repealed) (CA 28, 2000)

Article 234. It is forbidden for the Union to assume, directly or indirectly, as a result of the creation of a state, burdens related to expenses with retired or pensioner personnel and with charges and repayments of internal or foreign debt of the public administration, including those of the associated entities.

Article 235. During the first ten years after the creation of a state, the following basic rules shall be observed:

I – the State Legislature shall be composed of seventeen deputies if the population of the state is less than six hundred thousand inhabitants, and of twenty-four deputies if it is equal to or greater than this number, up to one million and five hundred thousand inhabitants;

II – the government shall have at most ten Secretariats;

III– the Accounting Court shall have three members, appointed by the elected governor, among Brazilians of proven trustworthiness and renowned legal knowledge;

IV – the Court of Appeal shall have seven judges;

V – the first judges shall be appointed by the elected governor, chosen in the following manner:

a) five from among judges at thirty-five years of age at minimum, in exercise within the area of the new state or of the original one;

b) two from among prosecutors, under the same conditions, and from among attorneys of proven the highest moral reputation and renowned legal knowledge, with at least ten years of professional practice, complying with the procedures set forth in this Constitution;

VI – in the case of a state which originated from a federal territory, the first five appellate judges may be chosen from among judges from any part of the country;

VII – in each judicial district the first state judge, the first state prosecutor and the first public defender shall be appointed by the elected governor after a competitive civil-servant examination of tests and presentation of academic and professional credentials;

VIII – until the promulgation of the State Constitution, the Offices of the State Attorney General, of the General Counsel to the States and of the Public Defender of the States shall be held by lawyers of renowned legal knowledge, at age of thirty-five years at minimum, appointed by the elected governor and removable *ad nutum*;

IX – if the new state results from the transformation of a federal territory, the transfer of financial burden from the Union to pay opting civil servants who belonged to the federal administration, shall take place as follows:

a) in the sixth year after its creation, the state shall assume twenty percent of the financial obligation to pay opting civil servants, the remainder continuing as a responsibility of the Union;

b) in the seventh year, thirty percent shall be added to the amount charged to the state and, in the eighth year, the remaining fifty percent;

X – the appointments subsequent to the first ones, for the offices mentioned in this Article, shall be regulated by the State Constitution;

XI – the budget expenses with personnel shall not exceed fifty percent of the revenues of the state.

Article 236. Notary and registration services shall be exercised by private entities by government delegation.

Paragraph 1. The law shall regulate the activities, discipline the civil and criminal liability of notaries, registrars and their officials and define the supervision of their acts by the judiciary.

Paragraph 2. Federal law shall set forth general rules for the establishment of fees for the acts performed by notary and registration services.

Paragraph 3. The entrance in notary and registration activities shall depend on a competitive civil-servant examination of tests and presentation of academic and professional credentials, and an office shall not be permitted to remain vacant for more than six months, without the opening of a public examination to fill it, either by appointment or transference.

Article 237. The supervision and control of foreign trade, which are essential to the defense of national financial interests, shall be exercised by the Ministry of Finance.

Article 238. The law shall organize the sale and resale of petroleum-derived fuels, fuel alcohol and other fuels derived from renewable raw materials, respecting the principles of this Constitution.

Article 239. The revenues from contributions to the Social Integration Program, created by the Supplementary Law nº 7 of September 7, 1970, and to the Civil Servants Asset Development Program, created by the Supplementary Law nº 8, of December 3, 1970, shall, from the date of the promulgation of this Constitution, fund the unemployment insurance program, other social security actions and the allowance referred to in paragraph 3 of this Article. (CA 103, 2019)

Paragraph 1. At least twenty-eight percent (28%) of the funds mentioned in the head of this Article shall be allocated to finance economic development programs through the National Bank for Economic and Social Development, with remuneration criteria that preserve their value. (CA 103, 2019)

Paragraph 2. Accrued assets of the Social Integration Program and of the Civil Servants Asset Development Program shall be preserved, maintaining the criteria for withdrawal in the situations provided by specific legislation, except for withdrawal by reason of marriage, it being forbidden the distribution of the revenues referred to in the head of this Article, for deposit in the personal accounts of the participants.

Paragraph 3. Employees who receive monthly remuneration of up to two minimum wages from employers who contribute to the Social Integration Program and to the Civil Servants Asset Development Program shall be ensured the annual payment of one minimum wage, in which value the income of the individual accounts shall be computed, in the case of those who already participated in such programs before the date of promulgation of this Constitution.

Paragraph 4. Funding of the unemployment insurance program shall receive an additional contribution from companies in which employee turnover exceeds the average turnover rate of the sector, in the manner established by law.

Paragraph 5. The economic development programs financed in accordance with paragraph 1 and their results shall be annually evaluated and disclosed in electronic media and presented at the meeting of the permanent joint committee referred to in paragraph 1 of Article 166. (CA 103, 2019)

Article 240. The present compulsory contributions calculated on the payroll, made by employers, intended for private social service and professional training entities linked to the labor union system, are excluded from the provisions of Article 195.

Article 241. The Union, states, Federal District, and municipalities shall issue legislation to regulate public consortia and cooperation agreements between members of the Federation, authorizing the joint management of public services, as well as the transfer, in whole or in part, of charges, services, personnel, and goods essential to the continued provision of the services transferred. (CA 19, 1998)

Article 242. The principle of Article 206, item IV, shall not apply to the official educational institutions created by state or municipal law and in existence on the date of promulgation of this Constitution, which are not totally or predominantly maintained with public funds.

Paragraph 1. The teaching of Brazilian history shall consider the contribution of the different cultures and ethnic groups to the formation of the Brazilian people.

Paragraph 2. The Pedro II School, in the City of Rio de Janeiro, shall be maintained in the federal sphere.

Article 243. Rural and urban properties in any region of the country where illegal plantations of psychotropic plants are found or the exploitation of slave labor is uncovered, as defined by law, shall be expropriated and assigned to agrarian reform and to low-income housing programs, with no indemnity to the owner and without prejudice to other sanctions set forth by law, with due regard, when appropriate, for the provisions of Article 5. (CA 81, 2014)

Single paragraph. Any and all goods of economic value seized as a result of illegal traffic of narcotics and similar drugs and of the exploitation of slave labor shall be confiscated and shall revert to a special fund for a specific purpose, as the law provides. (CA 81, 2014)

Article 244. The law shall provide for the adaptation of presently existing sites and buildings of public use and of the public transportation vehicles in order to guarantee adequate access to the handicapped, as set forth in Article 227, paragraph 2.

Article 245. The law shall provide for the cases and conditions in which the government shall give assistance to the needy heirs and dependents of victims of willful crimes, without prejudice to the civil liability of the perpetrator of the offence.

Article 246. The adoption of provisional presidential decree to regulate any Article of the Constitution which the wording has been altered by means of an Amendment enacted between January 1st, 1995 and the date of enactment of this Amendment is forbidden. (CA 32, 2001)

Article 247. The laws provided for in item III of paragraph 1 of Article 41, and in paragraph 7 of Article 169, shall establish special criteria and guarantees for the loss of

office of a tenured civil servant who, by virtue of the duties of his effective post, performs exclusive activities of State. (CA 19, 1998)

Single paragraph. In the event of insufficient performance, the loss of office shall only take place by means of an administrative proceeding in which the adversary system and full defense are ensured. (CA 19, 1998)

Article 248. The benefits paid, under any instrument, by the government body in charge of the general social security regime, even if they are financed by the National Treasury, and those benefits not subject to the maximum amount stipulated for benefits granted by such scheme shall comply with the limits set forth in Article 37, item XI. (CA 20, 1998)

Article 249. For the purpose of securing resources for the payment of retirement benefits and other pensions granted to their respective employees and their dependents, in addition to the funds of their respective Treasuries, the Union, states, Federal District, and municipalities may establish funds, made up of funds arising from contributions, and of property, rights, and assets of any kind, by means of a law that shall provide for the nature and the management of such funds. (CA 20, 1998)

Article 250. For the purpose of securing resources for the payment of benefits granted by the general social security regime, in addition to the funds arising from taxation, the Union may establish a fund made up of property, rights, and assets of any kind, by means of a law that shall provide for the nature and the management of such a fund. (CA 20, 1998)

Brasília, October 5, 1988.

Ulysses Guimarães, President – Mauro Benevides, First Vice-President – Jorge Arbage, Second Vice-President – Marcelo Cordeiro, First Secretary – Mário Maia, Second Secretary – Arnaldo Faria de Sá, Third Secretary – Benedita da Silva, First Substitute Secretary – Luiz Soyer, Second Substitute Secretary – Sotero Cunha, Third Substitute Secretary – Bernardo Cabral, Reporter--General – Adolfo Oliveira, Adjunct Reporter – Antonio Carlos Konder Reis, Adjunct Reporter – José Fogaça, Adjunct Reporter.

Abigail Feitosa – Acival Gomes – Adauto Pereira – Ademir Andrade – Adhemar de Barros Filho – Adroaldo Streck – Adylson Motta – Aécio de Borba – Aécio Neves – Affonso Camargo – Afif Domingos – Afonso Arinos – Afonso Sancho – Agassiz Almeida – Agripino de Oliveira Lima – Airton Cordeiro – Airton Sandoval – Alarico Abib – Albano Franco – Albérico Cordeiro – Albérico Filho – Alceni Guerra – Alcides Saldanha – Aldo Arantes – Alércio Dias – Alexandre Costa – Alexandre Puzyna – Alfredo Campos – Almir Gabriel – Aloisio Vasconcelos – Aloysio Chaves – Aloysio Teixeira – Aluizio Bezerra – Aluízio Campos – Álvaro Antônio – Álvaro Pacheco

– Álvaro Valle – Alysson Paulinelli – Amaral Netto – Amaury Müller – Amilcar Moreira – Ângelo Magalhães – Anna Maria Rattes – Annibal Barcellos – Antero de Barros – Antônio Câmara – Antônio Carlos Franco – Antonio Carlos Mendes Thame – Antônio de Jesus – Antonio Ferreira – Antonio Gaspar – Antonio Mariz – Antonio Perosa – Antônio Salim Curiati – Antonio Ueno – Arnaldo Martins – Arnaldo Moraes – Arnaldo Prieto – Arnold Fioravante – Arolde de Oliveira – Artenir Werner – Artur da Távola – Asdrubal Bentes – Assis Canuto – Átila Lira – Augusto Carvalho – Áureo Mello – Basílio Villani – Benedicto Monteiro – Benito Gama – Beth Azize – Bezerra de Melo – Bocayuva Cunha – Bonifácio de Andrada – Bosco França – Brandão Monteiro – Caio Pompeu – Carlos Alberto – Carlos Alberto Caó – Carlos Benevides – Carlos Cardinal – Carlos Chiarelli – Carlos Cotta – Carlos De'Carli – Carlos Mosconi – Carlos Sant'Anna – Carlos Vinagre – Carlos Virgílio – Carrel Benevides – Cássio Cunha Lima – Célio de Castro – Celso Dourado – César Cals Neto – César Maia – Chagas Duarte – Chagas Neto – Chagas Rodrigues – Chico Humberto – Christóvam Chiaradia – Cid Carvalho – Cid Sabóia de Carvalho – Cláudio Ávila – Cleonâncio Fonseca – Costa Ferreira – Cristina Tavares – Cunha Bueno – Dálton Canabrava – Darcy Deitos – Darcy Pozza – Daso Coimbra – Davi Alves Silva – Del Bosco Amaral – Delfim Netto – Délio Braz – Denisar Arneiro – Dionisio Dal Prá – Dionísio Hage – Dirce Tutu Quadros – Dirceu Carneiro – Divaldo Suruagy – Djenal Gonçalves – Domingos Juvenil – Domingos Leonelli – Doreto Campanari – Edésio Frias – Edison Lobão – Edivaldo Motta – Edme Tavares – Edmilson Valentim – Eduardo Bonfim – Eduardo Jorge – Eduardo Moreira – Egídio Ferreira Lima – Elias Murad – Eliel Rodrigues – Eliézer Moreira – Enoc Vieira – Eraldo Tinoco – Eraldo Trindade – Erico Pegoraro – Ervin Bonkoski – Etevaldo Nogueira – Euclides Scalco – Eunice Michiles – Evaldo Gonçalves – Expedito Machado – Ézio Ferreira – Fábio Feldmann – Fábio Raunheitti – Farabulini Júnior – Fausto Fernandes – Fausto Rocha – Felipe Mendes – Feres Nader – Fernando Bezerra Coelho – Fernando Cunha – Fernando Gasparian – Fernando Gomes – Fernando Henrique Cardoso – Fernando Lyra – Fernando Santana – Fernando Velasco – Firmo de Castro – Flavio Palmier da Veiga – Flávio Rocha – Florestan Fernandes – Floriceno Paixão – França Teixeira – Francisco Amaral – Francisco Benjamim – Francisco Carneiro – Francisco Coelho – Francisco Diógenes – Francisco Dornelles – Francisco Küster – Francisco Pinto – Francisco Rollemberg – Francisco Rossi – Francisco Sales – Furtado Leite – Gabriel Guerreiro – Gandi Jamil – Gastone Righi – Genebaldo Correia – Genésio Bernardino – Geovani Borges – Geraldo Alckmin Filho – Geraldo Bulhões – Geraldo Campos – Geraldo Fleming – Geraldo Melo – Gerson Camata – Gerson Marcondes – Gerson Peres – Gidel Dantas – Gil César – Gilson Machado – Gonzaga Patriota – Guilherme Palmeira – Gumercindo Milhomem – Gustavo de Faria – Harlan Gadelha – Haroldo Lima – Haroldo Sabóia – Hélio Costa – Hélio Duque – Hélio Manhães – Hélio Rosas – Henrique Córdova – Henrique Eduardo Alves – Heráclito Fortes –

Hermes Zaneti – Hilário Braun – Homero Santos – Humberto Lucena – Humberto Souto – Iberê Ferreira – Ibsen Pinheiro – Inocêncio Oliveira – Irajá Rodrigues – Iram Saraiva – Irapuan Costa Júnior – Irma Passoni – Ismael Wanderley – Israel Pinheiro – Itamar Franco – Ivo Cersósimo – Ivo Lech – Ivo Mainardi – Ivo Vanderlinde – Jacy Scanagatta – Jairo Azi – Jairo Carneiro – Jalles Fontoura – Jamil Haddad – Jarbas Passarinho – Jayme Paliarin – Jayme Santana – Jesualdo Cavalcanti – Jesus Tajra – Joaci Góes – João Agripino – João Alves – João Calmon – João Carlos Bacelar – João Castelo – João Cunha – João da Mata – João de Deus Antunes – João Herrmann Neto – João Lobo – João Machado Rollemberg – João Menezes – João Natal – João Paulo – João Rezek – Joaquim Bevilácqua – Joaquim Francisco – Joaquim Hayckel – Joaquim Sucena – Jofran Frejat – Jonas Pinheiro – Jonival Lucas – Jorge Bornhausen – Jorge Hage – Jorge Leite – Jorge Uequed – Jorge Vianna – José Agripino – José Camargo – José Carlos Coutinho – José Carlos Grecco – José Carlos Martinez – José Carlos Sabóia – José Carlos Vasconcelos – José Costa – José da Conceição – José Dutra – José Egreja – José Elias – José Fernandes – José Freire – José Genoíno – José Geraldo – José Guedes – José Ignácio Ferreira – José Jorge – José Lins – José Lourenço – José Luiz de Sá – José Luiz Maia – José Maranhão – José Maria Eymael – José Maurício – José Melo – José Mendonça Bezerra – José Moura – José Paulo Bisol – José Queiroz – José Richa – José Santana de Vasconcellos – José Serra – José Tavares – José Teixeira – José Thomaz Nonô – José Tinoco – José Ulísses de Oliveira – José Viana – José Yunes – Jovanni Masini – Juarez Antunes – Júlio Campos – Júlio Costamilan – Jutahy Júnior – Jutahy Magalhães – Koyu Iha – Lael Varella – Lavoisier Maia – Leite Chaves – Lélio Souza – Leopoldo Peres – Leur Lomanto – Levy Dias – Lézio Sathler – Lídice da Mata – Louremberg Nunes Rocha – Lourival Baptista – Lúcia Braga – Lúcia Vânia – Lúcio Alcântara – Luís Eduardo – Luís Roberto Ponte – Luiz Alberto Rodrigues – Luiz Freire – Luiz Gushiken – Luiz Henrique – Luiz Inácio Lula da Silva – Luiz Leal – Luiz Marques – Luiz Salomão – Luiz Viana – Luiz Viana Neto – Lysâneas Maciel – Maguito Vilela – Maluly Neto – Manoel Castro – Manoel Moreira – Manoel Ribeiro – Mansueto de Lavor – Manuel Viana – Márcia Kubitschek – Márcio Braga – Márcio Lacerda – Marco Maciel – Marcondes Gadelha – Marcos Lima – Marcos Queiroz – Maria de Lourdes Abadia – Maria Lúcia – Mário Assad – Mário Covas – Mário de Oliveira – Mário Lima – Marluce Pinto – Matheus Iensen – Mattos Leão – Maurício Campos – Maurício Correa – Maurício Fruet – Maurício Nasser – Maurício Pádua – Maurílio Ferreira Lima – Mauro Borges – Mauro Campos – Mauro Miranda – Mauro Sampaio – Max Rosenmann – Meira Filho – Melo Freire – Mello Reis – Mendes Botelho – Mendes Canale – Mendes Ribeiro – Messias Góis – Messias Soares – Michel Temer – Milton Barbosa – Milton Lima – Milton Reis – Miraldo Gomes – Miro Teixeira – Moema São Thiago – Moysés Pimentel – Mozarildo Cavalcanti – Mussa Demes – Myrian Portella – Nabor Júnior – Naphtali Alves de Souza – Narciso Mendes – Nelson Aguiar – Nelson Carneiro – Nelson Jobim

– Nelson Sabrá – Nelson Seixas – Nelson Wedekin – Nelton Friedrich – Nestor Duarte – Ney Maranhão – Nilso Sguarezi – Nilson Gibson – Nion Albernaz – Noel de Carvalho – Nyder Barbosa – Octávio Elísio – Odacir Soares – Olavo Pires – Olívio Dutra – Onofre Corrêa – Orlando Bezerra – Orlando Pacheco – Oscar Corrêa – Osmar Leitão – Osmir Lima – Osmundo Rebouças – Osvaldo Bender – Osvaldo Coelho – Osvaldo Macedo – Osvaldo Sobrinho – Oswaldo Almeida – Oswaldo Trevisan – Ottomar Pinto – Paes de Andrade – Paes Landim – Paulo Delgado – Paulo Macarini – Paulo Marques – Paulo Mincarone – Paulo Paim – Paulo Pimentel – Paulo Ramos – Paulo Roberto – Paulo Roberto Cunha – Paulo Silva – Paulo Zarzur – Pedro Canedo – Pedro Ceolin – Percival Muniz – Pimenta da Veiga – Plínio Arruda Sampaio – Plínio Martins – Pompeu de Sousa – Rachid Saldanha Derzi – Raimundo Bezerra – Raimundo Lira – Raimundo Rezende – Raquel Cândido – Raquel Capiberibe – Raul Belém – Raul Ferraz – Renan Calheiros – Renato Bernardi – Renato Johnsson – Renato Vianna – Ricardo Fiuza – Ricardo Izar – Rita Camata – Rita Furtado – Roberto Augusto – Roberto Balestra – Roberto Brant – Roberto Campos – Roberto D'Ávila – Roberto Freire – Roberto Jefferson – Roberto Rollemberg – Roberto Torres – Roberto Vital – Robson Marinho – Rodrigues Palma – Ronaldo Aragão – Ronaldo Carvalho – Ronaldo Cezar Coelho – Ronan Tito – Ronaro Corrêa – Rosa Prata – Rose de Freitas – Rospide Netto – Rubem Branquinho – Rubem Medina – Ruben Figueiró – Ruberval Pilotto – Ruy Bacelar – Ruy Nedel – Sadie Hauache – Salatiel Carvalho – Samir Achôa – Sandra Cavalcanti – Santinho Furtado – Sarney Filho – Saulo Queiroz – Sérgio Brito – Sérgio Spada – Sérgio Werneck – Severo Gomes – Sigmaringa Seixas – Sílvio Abreu – Simão Sessim – Siqueira Campos – Sólon Borges dos Reis – Stélio Dias – Tadeu França – Telmo Kirst – Teotonio Vilela Filho – Theodoro Mendes – Tito Costa – Ubiratan Aguiar – Ubiratan Spinelli – Uldurico Pinto – Valmir Campelo – Valter Pereira – Vasco Alves – Vicente Bogo – Victor Faccioni – Victor Fontana – Victor Trovão – Vieira da Silva – Vilson Souza – Vingt Rosado – Vinicius Cansanção – Virgildásio de Senna – Virgílio Galassi – Virgílio Guimarães – Vitor Buaiz – Vivaldo Barbosa – Vladimir Palmeira – Wagner Lago – Waldec Ornélas – Waldyr Pugliesi – Walmor de Luca – Wilma Maia – Wilson Campos – Wilson Martins – Ziza Valadares.

PARTICIPANTS: Álvaro Dias – Antônio Britto – Bete Mendes – Borges da Silveira – Cardoso Alves – Edivaldo Holanda – Expedito Júnior – Fadah Gattass – Francisco Dias – Geovah Amarante – Hélio Gueiros – Horácio Ferraz – Hugo Napoleão – Iturival Nascimento – Ivan Bonato – Jorge Medauar – José Mendonça de Morais – Leopoldo Bessone – Marcelo Miranda – Mauro Fecury – Neuto de Conto – Nivaldo Machado – Oswaldo Lima Filho – Paulo Almada – Prisco Viana – Ralph Biasi – Rosário Congro Neto – Sérgio Naya – Tidei de Lima.

IN MEMORIAM: Alair Ferreira – Antônio Farias – Fábio Lucena – Norberto Schwantes – Virgílio Távora.

TEMPORARY CONSTITUTIONAL PROVISIONS ACT

Article 1. The president of the Republic, the president of the Federal Supreme Court and the members of the National Congress shall take an oath to maintain, defend and comply with the Constitution, upon and on the date of the promulgation thereof.

Article 2. On September 7, 1993, the voters shall define, through a plebiscite, the form (Republic or Constitutional Monarchy) and system of government (parliamentary or presidential) to be in force in Brazil.

Paragraph 1. The free diffusion of these forms and systems through public utility mass communication vehicles shall be free of charge.

Paragraph 2. The Superior Electoral Court shall, upon promulgation of the Constitution, issue the regulatory rules for this Article.

Article 3. The revision of the Constitution shall take place after five years as of its promulgation, by vote of an absolute majority of the members of the National Congress in a unicameral session.

Article 4. The term of office of the incumbent president of the Republic shall end on March 15, 1990.

Paragraph 1. The first election for president of the Republic after promulgation of the Constitution shall be held on November 15, 1989, and the provisions of Article 16 of the Constitution shall not apply thereto.

Paragraph 2. The impossibility to reduce the present representation of the states and the Federal District in the Chamber of Deputies is ensured.

Paragraph 3. The terms of office of the governors and of the vice-governors elected on November 15, 1986 shall end on March 15, 1991.

Paragraph 4. The terms of office of the present mayors, vice-mayors and municipal councilors shall end on January 1st, 1989, with the inauguration of those elected.

Article 5. The provisions of Article 16 and the rules of Article 77 of the Constitution do not apply to the elections scheduled for November 15, 1988.

Paragraph 1. For the elections of November 15, 1988, an electoral domicile in the electoral district of at least four months prior to the election shall be required, and the candidates who fulfill this requirement and satisfy the other legal requisites may register with the Electoral Courts after the Constitution is promulgated.

Paragraph 2. In the absence of a specific legal rule, the Superior Electoral Court is competent to issue the rules required to hold the 1988 elections, with due regard for the laws in force.

Paragraph 3. Present federal and state parliamentarians elected for vice-mayor, if called to exercise the office of mayor, shall not lose their parliamentary office.

Paragraph 4. The number of councilors per municipality shall be determined, for the representation to be elected in 1988, by the respective Regional Electoral Court, with due regard for the limits established in Article 29, item IV, of the Constitution.

Paragraph 5. For the elections to be held on November 15, 1988, except for those who already hold an elective office, the spouse and relatives by blood or marriage up to the second degree or relatives by adoption of the president of the Republic, of a state governor, or the governor of the Federal District and of a mayor who have served more than half of their term of office, are ineligible for any office within the jurisdiction of the office holder.

Article 6. A group of at least thirty congress members may, during the six months following the promulgation of the Constitution request from the Superior Electoral Court the registration of a new political party, the petition to be accompanied by the respective manifest, the by-laws and the program duly signed by the petitioners.

Paragraph 1. The provisional registration, which shall be promptly granted by the Superior Electoral Court, according to this Article, grants to the new party all rights, duties and prerogatives of the existing parties, among which the right to take part, under its own name, in the elections to be held during the twelve months following its formation.

Paragraph 2. The new party shall automatically lose its provisional registration if, within twenty-four months of its formation, it fails to obtain the final registration at the Superior Electoral Court, as established by law.

Article 7. Brazil shall strive for the creation of an international court of human rights.

Article 8. Amnesty is granted to those who, during the period from September 18, 1946, to the date the Constitution is promulgated, have been affected, exclusively for political reasons, by institutional or supplementary acts of exception, to those encompassed in Legislative Decree nº 18, of December 15, 1961, and to those affected by Decree-Law nº

846, of September 12, 1969, ensuring the promotions, in their inactivity, to the office, position or rank to which they would be entitled if they were in active service, with due regard for the periods of continuous activity set forth in laws and regulations in force, respecting the characteristics and peculiarities of the careers of civil and military public servants and complying with the respective legal regimes.

Paragraph 1. The provisions of this Article shall only generate financial effects as from the promulgation of the Constitution, any kind of retroactive compensation being forbidden.

Paragraph 2. The benefits established in this Article are ensured to workers of the private sector, union officers and representatives who, for exclusively political reasons, have been punished, dismissed or compelled to leave the remunerated activities they had been performing, as well as to those who have been prevented from performing their professional activities due to ostensive pressures or secret official procedures.

Paragraph 3. Reparation of economic nature shall be granted, as set forth by a law to be proposed by the National Congress and to become effective within twelve months counted from the promulgation of the Constitution, to citizens who were prevented from performing, as civilians, a specific professional activity as a result of Reserved Ordinances of the Ministry of the Air Force S50GM5 of June 19, 1964, and S285GM5.

Paragraph 4. To those who, due to institutional acts, have gratuitously exercised elective offices of municipal councilors, the respective periods shall be computed for purposes of social security and retirement from civil service.

Paragraph 5. The amnesty granted under this Article applies to civil servants and to employees at all levels of government or at its foundations, state-owned or mixed-capital companies under state control, except in the military Ministries, who have been punished or dismissed from professional activities interrupted by decision of their employees, as well as a result of Decree Law nº 1,632, of August 4, 1978, or for exclusively political reasons, the readmission of those affected as from 1979 being ensured, with due regard for the provisions of paragraph 1.

Article 9. Those who, for exclusively political reasons, were disfranchised or had their political rights suspended during the period from July 15 to December 31, 1969, by an act of the then president of the Republic, may request the Federal Supreme Court to acknowledge the rights and advantages interrupted by the punitive acts, as long as they prove that such acts were marked by gross flaws.

Single paragraph. The Federal Supreme Court shall render the decision within one hundred and twenty days from the request of the interested party.

Article 10. Until the supplementary law referred to in Article 7, item I, of the Constitution is promulgated:

I – the protection referred to therein is limited to the increase, to four times, of the percentage set forth in Article 6, heading and paragraph 1, of the Law nº 5,107 of September 13, 1966;

II – arbitrary dismissal or dismissal without cause is prohibited:

a) of employees elected to an executive office of internal accident prevention committees, from the date of registration of their candidacy to one year after the end of their term of office;

b) of pregnant employees, from the date the pregnancy is confirmed to five months after birth.

Paragraph 1. Until such time as the law shall regulate the provisions of Article 7, item XIX, of the Constitution, the period of paternity leave referred to in the item is of five days.

Paragraph 2. Until further legal provisions are established, the contributions to fund the activities of rural unions shall be collected with the rural property tax, by the same collecting body.

Paragraph 3. Upon the first proof of fulfilment of labor obligations by rural employers, as established by Article 2337, after the promulgation of the Constitution, the conformity of the contract to the law and of the correction of the labor obligations over the entire period shall be certified before the Labor Courts.

Article 11. Each State Legislature endowed with constituent powers, shall draft the State Constitution within one year as from the promulgation of the Federal Constitution, with due regard for the principles of the latter.

Single paragraph. After the promulgation of the State Constitution, the municipal council is responsible for, within six months, voting the respective Organic Law, in two rounds of discussion and voting, with due regard for the provisions of the Federal and State Constitutions.

Article 12. Within ninety days of the promulgation of the Constitution, a land studies committee shall be created, with ten members nominated by the National Congress and five members by the executive branch , for the purpose of submitting studies concerning the national territory and draft bills regarding new territorial units, particularly in the Legal Amazonian Region and in areas pending solution.

Paragraph 1. Within one year the committee shall submit the results of its studies to the National Congress so that, in accordance with the Constitution, such studies may be

examined during the twelve subsequent months, the committee being dissolved shortly thereafter.

Paragraph 2. The states and municipalities shall, within three years of the promulgation of the Constitution, provide, by agreement or adjustment, for the demarcation of their borders presently in litigation, and they may for such purpose effect area alterations and compensations which allow for natural features, historical criteria, administrative ease and convenience of the bordering populations.

Paragraph 3. At the request of the interested states and municipalities, the Union may undertake the demarcation work.

Paragraph 4. If, three years after the promulgation of the Constitution, the demarcation work has not been completed, the Union shall determine the borders of the areas under litigation.

Paragraph 5. The present borders of the State of Acre with the States of Amazonas and Rondônia are hereby recognized and ratified according to cartographic and geodesic surveys conducted by the tripartite committee formed by representatives of the states and of the specialized technical services of the Brazilian Institute of Geography and Statistics.

Article 13. The State of Tocantins is created by separation of the area described in this Article and its installation shall occur on the forty-sixth day after the election provided for in paragraph 3, but not before January 1, 1989.

Paragraph 1. The State of Tocantins is part of the Northern Region and borders with the State of Goiás along the northern boundaries of the municipalities of São Miguel do Araguaia, Porangatu, Formoso, Minaçu, Cavalcante, Monte Alegre de Goiás and Campos Belos, maintaining the present eastern, northern and western borders of Goiás with the States of Bahia, Piauí, Maranhão, Pará and Mato Grosso.

Paragraph 2. The executive branch shall designate one of the cities of the State as its provisional capital until such time as the final seat of government is approved by the Constituent Assembly.

Paragraph 3. The governor, the vice-governor, the senators and the federal and state deputies shall be elected, in a single voting, within seventy five days after the promulgation of the Constitution, but not before November 15, 1988, at the discretion of the Superior Electoral Court, with due regard, among others, for the following rules:

I – the deadline for affiliation of the candidates to the parties shall end seventy-five days prior to the date of the elections;

II – the dates for the regional party conventions for the purpose of deciding upon coalitions and choice of candidates, for the presentation of the application for registration of the candidates chosen and for the other legal procedures shall be determined by the Electoral Courts in a special schedule;

III – the holders of state or municipal offices who have not left such offices on a definitive basis seventy-five days prior to the date of the elections provided for in this paragraph shall be ineligible;

IV – the present regional committees of the political parties of the State of Goiás are maintained, and the national executive committees have the duty to appoint provisional committees for the State of Tocantins, in accordance with and for the purposes established by law.

Paragraph 4. The terms of office of governor, vice-governor and federal and state deputies elected in accordance with the preceding paragraph shall end concurrently with those of the other federal entities; the term of office of the least voted elected senator shall end on the same occasion and the terms of office of the other two senators shall end together with those of the senators elected in 1986 in the other states.

Paragraph 5. The State Constituent Assembly shall be installed on the forty-sixth day as from the election of its members, but not before January 1, 1989, under the chairmanship of the president of the Regional Electoral Court of the State of Goiás, and shall on the same date inaugurate the elected governor and vice-governor.

Paragraph 6. The legal rules regulating the division of the State of Mato Grosso shall apply, where appropriate, to the creation and installation of the State of Tocantins with due regard for the provisions of Article 234 of the Constitution.

Paragraph 7. The State of Goiás shall be released from debts and burdens resulting from undertakings within the territory of the new state, and the Union is authorized, at its discretion, to take over such debts.

Article 14. The Federal Territories of Roraima and of Amapá are transformed into federated states, their present geographic borders being maintained.

Paragraph 1. The installation of the states shall occur upon the inauguration of the governors elected in 1990.

Paragraph 2. The rules and criteria adopted for the creation of the State of Rondônia shall apply to the transformation and installation of the States of Roraima and Amapá, with due regard for the provisions of the Constitution and of this Act.

Paragraph 3. The president of the Republic shall, within forty-five days of the promulgation of the Constitution, submit for examination by the Federal Senate the names

of the governors of the States of Roraima and Amapá who shall exercise the executive branch until the new states are installed with the inauguration of the elected governors.

Paragraph 4. Until the transformation into states is implemented according to this Article, the Federal Territories of Roraima and Amapá shall enjoy the benefits of transfer of funds provided for in Article 159, item I, subitem *a*, of the Constitution and Article 34, paragraph 2, item II, of this Act.

Article 15. The Federal Territory of Fernando de Noronha is extinguished, and its area reincorporated into the State of Pernambuco.

Article 16. Until the provisions of Article 32, paragraph 2, of the Constitution are implemented, the present of the Republic is responsible for, with the approval of the Federal Senate, appointing the governor and vice-governor of the Federal District.

Paragraph 1. The authority of the Legislative Chamber of the Federal District shall, until such time as it is installed, be exercised by the Federal Senate.

Paragraph 2. The accounting, financial, budget, operational and property supervision of the Federal District shall, until such time as the Legislative Chamber is installed, be carried out by the Federal Senate, by means of external control, with the assistance of the Accounting Court of the Federal District, with due regard for the provisions of Article 72 of the Constitution.

Paragraph 3. The assets of the Federal District shall include those which may be assigned to it by the Union as established by law.

Article 17. Earnings, compensation, advantages and additional pay, as well as retirement benefits which are being received in disagreement with this Constitution, shall be reduced immediately to the limits arising therefrom, it not being allowed, in this case, to invoke a vested right or receipt of excess on any account.

Paragraph 1. It is ensured the cumulative occupation of two medical offices or jobs that are held by a military physician in the government bodies or associated entities.

Paragraph 2. Cumulative occupation of two offices or jobs reserved for health professionals is ensured if the positions are in the government bodies or associated entities.

Article 18. The legal effects of any legislative or administrative act drawn up as of the installation of the National Constituent Assembly, with the objective of granting tenure to a public servant admitted without a competitive civil-servant examination into government bodies or associated entities, including the foundations instituted and maintained by the government, shall be extinguished.

Article 19. Civil servants of the Union, states, Federal District and municipalities, of the government bodies, agencies and foundations, who, on the date of promulgation

of the Constitution, have been in office for at least five continuous years, and who have not been admitted as established in Article 37 of the Constitution, are deemed to have tenure in the public service.

Paragraph 1. The period of service of the civil servants referred to in this Article shall be considered as a credential when they take a competitive examination for the purpose of acquiring tenure, as set forth by law.

Paragraph 2. The provisions of this Article do not apply to the holders of trust or commission functions and jobs nor to those who are legally subject to free discharge, whose period of service shall not be computed for the purposes of the head of this Article, except for public servants.

Paragraph 3. The provisions of this Article shall not apply to higher education professors as set forth by law.

Article 20. Within one hundred and eighty days, the rights of retired servants and pensioners shall be revised, and the income and pensions owed to them shall be updated in order to adjust them to the provisions of the Constitution.

Article 21. Judges vested in office for a limited period, who have been admitted by means of a competitive public examination of tests and presentation of academic and professional credentials and who are in office on the date this Constitution is promulgated, shall achieve tenure with due regard for the probation period and they shall be included in a special job class to be terminated, maintaining the authority, prerogatives and restrictions of the legislations which they were subject to, except for those inherent to the temporary nature of their investiture.

Single paragraph. The retirement of the judges referred to in this Article shall be regulated by the rules established for other state judges.

Article 22. Public defenders vested in office before the date of installation of the National Constituent Assembly are ensured the right to opt for the career, complying with the guarantees and prohibitions set forth in Article 134, single paragraph, of the Constitution.

Article 23. Until such time as the regulations of Article 21, item XVI, of the Constitution are issued, the present holders of the office of federal censor shall continue to exercise functions compatible with such office in the Federal Police, with due regard for the constitutional provisions.

Single paragraph. Such law shall provide for the reassignment of the Federal Censors as set forth in this Article.

Article 24. The Union, states, Federal District and municipalities shall issue laws establishing criteria to make their staffs compatible with the provisions of Article 39 of

the Constitution and with the administrative reorganization resulting therefrom, within eighteen months as from the promulgation of the Constitution.

Article 25. As of one hundred and eighty days after the promulgation of the Constitution, such period being subject to extension by law, all legal provisions which confer on or delegate to a body of the Executive branch authority assigned to the National Congress by the Constitution shall be repealed, especially those referring to:

I – normative action;

II – allocation or transfer of funds of any kind.

Paragraph 1. The decree laws pending before the National Congress and not examined by it before the Constitution is promulgated shall have their effects regulated as follows:

I – if issued up to September 2, 1988, they shall be examined by the National Congress within one hundred and eighty days as from the date of the promulgation of the Constitution, not counting the parliamentary recess;

II – if the time limit defined in the preceding item elapses without the decree-laws mentioned therein having been examined, they shall be considered rejected;

III – in the cases defined in items I and II, the acts performed during the effectiveness of the respective decree-laws shall be fully valid and the National Congress may, if necessary, legislate on their remaining effects.

Paragraph 2. The decree-laws issued between September 3, 1988 and the date of the promulgation of the Constitution shall be converted on such date into provisional presidential decree, with the rules established in Article 62, single paragraph, being applied thereto.

Article 26. Within one year of promulgation of the Constitution, the National Congress shall conduct, through a joint committee, an analytical and expert examination of the acts and facts which generate the Brazilian foreign indebtedness.

Paragraph 1. The committee shall have the legal authority of a parliamentary investigation committee for purposes of requisition and summons and shall act with the assistance of the Federal Accounting Court.

Paragraph 2. If irregularities are found, the National Congress shall propose that the executive branch declare the act null and void and shall forward the case to the Federal Prosecution Office, which shall take the appropriate action within sixty days.

Article 27. The Superior Court of Justice shall be installed under the Presidency of the Federal Supreme Court.

Paragraph 1. Until such time as the Superior Court of Justice is installed, the Federal Supreme Court shall perform the duties and responsibilities defined in the previous constitutional order.

Paragraph 2. The initial composition of the Superior Court of Justice shall be obtained:

I – by reassignment of justices of the Federal Court of Appeal;

II – by appointment of the justices required to complete the number established in the Constitution.

Paragraph 3. For the purposes of the Constitution, the present justices of the Federal Court of Appeals shall be considered as belonging to the class they came from at the time of their appointment.

Paragraph 4. Once the Court has been installed, the retired justices of the Federal Court of Appeal shall automatically become retired justices of the Superior Court of Justice.

Paragraph 5. The justices referred to in paragraph 2, item II, shall be nominated in a triple list by the Federal Court of Appeal, with due regard for the provisions of Article 104, single paragraph, of the Constitution.

Paragraph 6. Five Federal Regional Courts of Justice are hereby created, to be installed within six months of the promulgation of the Constitution, with the jurisdiction and seat assigned to them by the Federal Court of Appeal, taking into account the number of lawsuits and their geographical location.

Paragraph 7. Until such time as the Regional Federal Courts of Appeal are installed, the Federal Court of Appeal shall exercise the authority attributed to them throughout the national territory, it being incumbent upon it their installation and nomination of candidates for all initial offices by means of a triple list which may include federal judges of any region, with due regard for the provisions of paragraph 9.

Paragraph 8. As from promulgation of the Constitution, it is forbidden to fill vacant offices of justices of the Federal Court of Appeal.

Paragraph 9. If there is no federal judge with the minimum period of service set forth in Article 107, item II, of the Constitution, the promotion may be granted to a judge with less than five years of office.

Paragraph 10. Federal Courts have jurisdiction to try lawsuits filed therein until such time as the Constitution is promulgated, and the Regional Federal Courts of Appeal as well as the Superior Court of Justice shall try the actions to overrule the final decision rendered until then by the Federal Courts, including those which refer to matters for which competence has been transferred to another branch of the Judiciary.

Paragraph 11. The following Regional Federal Courts of Appeal are also hereby created: the Regional Federal Court of the 6th Region, with seat in Curitiba, State of Paraná, and jurisdiction over the States of Paraná, Santa Catarina, and Mato Grosso do Sul; the Regional Federal Court of the 7th Region, with seat in Belo Horizonte, State of Minas Gerais, and jurisdiction over the State of Minas Gerais; the Regional Federal Court of the 8th Region, with seat in Salvador, State of Bahia, and jurisdiction over the States of Bahia and Sergipe; and the Regional Federal Court of the 9th Region, with seat in Manaus, State of Amazonas, and jurisdiction over the States of Amazonas, Acre, Rondônia, and Roraima. (CA 73, 2013)

Article 28. The federal judges referred to in Article 123, paragraph 2, of the Constitution of 1967, with the wording given by Constitutional Amendment 7 of 1977, shall be vested in office in courts of the judiciary section for which they were appointed or designated; if there are no vacancies, the existing courts shall be divided.

Single paragraph. For purposes of promotion for seniority, the period of service of such judges shall be computed as from the day of their taking of office.

Article 29. Until such time as the supplementary laws relating to the Prosecution Office and to the Office of the General Counsel to the Federal Government are approved, the Federal Prosecution Office, the Office of the General Counsel to the National Treasury, the Legal Consultancies of the Ministries, the Prosecution and Legal Departments of the federal government agencies, and the members of the Prosecution Offices of public foundation universities shall continue to conduct their activities within their respective incumbencies.

Paragraph 1. The president of the Republic shall, within one hundred and twenty days, submit to the National Congress a bill of supplementary law dealing with the organization and operation of the Office of the General Counsel to the Federal Government.

Paragraph 2. The present federal prosecutors may, in accordance with the supplementary law, opt irrevocably between the careers of the Federal Prosecution Office and of the Office of the General Counsel to the Federal Government.

Paragraph 3. A member of the Prosecution Office admitted prior to the promulgation of the Constitution may opt for the previous regime insofar as guarantees and advantages are concerned, with due regard, as to prohibitions, for the legal status on the date of such promulgation.

Paragraph 4. The present members of the supplementary staff of the Labor and Military Prosecution Offices, who have acquired tenure in these functions, shall belong to the staff of the respective career.

Paragraph 5. The present Office of the General Counsel to the National Treasury is responsible, directly or by delegation, which may be made to the State Prosecution Office, for representing the Union in court in fiscal lawsuits, in their respective spheres of authority, until such time as the supplementary laws set forth in this Article are promulgated.

Article 30. The legislation which creates the justiceship of the peace shall maintain the present judges of the peace until the new judges take office, ensuring them the rights and duties conferred on the latter and shall establish the date for the election provided for in Article 98, item II, of this Constitution.

Article 31. The clerical offices of the judicial courts, as defined in law, shall be brought under State control, with due regard for the rights of the present clerks.

Article 32. The provisions of Article 236 shall not apply to notary and registration services which have already been made official by the government, with due regard for the rights of their servants.

Article 33. With the exception of credits for alimony, the amount due by virtue of court orders for which payment is outstanding on the date of the promulgation of the Constitution, therein included remaining interests and adjustment for inflation, may be paid in legal tender, with readjustments, in equal and successive annual instalments, within eight years at the most, counted from July 1, 1989, in accordance with a decision by the Executive branch within one hundred and eighty days of the promulgation of the Constitution.

Single paragraph. In order to comply with the provisions of this Article, the debtor entities may issue, each year, for the exact amount of the expenditure, public debt bonds which shall not be computed for purposes of determining the total limit of indebtedness.

Article 34. The national tax system shall become effective on the first day of the fifth month following the promulgation of the Constitution, and until then, the system set forth in the 1967 Constitution, with the wording provided by Amendment 1 of 1969 and by the subsequent ones, shall be maintained.

Paragraph 1. With the promulgation of this Constitution, Articles 148, 149, 150, 154, item I, 156, item III, and 159, item I, subitem *c*, shall enter in force, with all provisions to the contrary in the 1967 Constitution and in the amendments which modified it, especially its Article 25, item III, being repealed.

Paragraph 2. The Participation Fund of the States and the Federal District, and the Participation Fund of the Municipalities shall obey the following determinations:

I – from the date of promulgation of the Constitution, the percentages shall be, respectively, of eighteen and twenty percent, calculated on the proceeds from the tax collection

referred to in Article 153, items III and IV, the present apportionment criteria being maintained until the supplementary law referred to in Article 161, item II, enters in force;

II – the percentage referring to the Participation Fund of the States and the Federal District shall be increased by one percent in the fiscal year of 1989 and, as from and including 1990, by one half of one percent per fiscal year until and including 1992, reaching in 1993 the percentage established in Article 159, item I, subitem *a*;

III – the percentage referring to the Participation Fund of the Municipalities, as from and including 1989 shall be increased by one half of one percent per fiscal year until it reaches the limit established in Article 159, item I, subitem *b*.

Paragraph 3. Upon the promulgation of this Constitution, the Union, states, Federal District and municipalities may issue the laws which are necessary for the application of the national tax system established therein.

Paragraph 4. The laws issued in accordance with the preceding paragraph produce effects as from the date the national tax system set forth in the Constitution enters in force.

Paragraph 5. Once the new national tax system is in force, the application of the preceding legislation shall be ensured in that in which it is not incompatible with the new system and with the legislation referred to in paragraphs 3 and 4.

Paragraph 6. Until December 31, 1989, the provisions of Article 150, item III, subitem *b*, shall not apply to the taxes referred to in Articles 155, item I, subitems *a* and *b*, and 156, items II and III, which may be collected thirty days after the publication of the law which has instituted or increased them.

Paragraph 7. Until the maximum rates of the municipal tax on retail sales of liquid and gaseous fuels have been established in a supplementary law, such rates shall not exceed three percent.

Paragraph 8. If, within sixty days counted from the promulgation of the Constitution, the supplementary law required for the institution of the tax referred to in Article 155, item I, subitem *b*, has not been issued, the states and the Federal District, by means of an agreement concluded in the manner set forth in Supplementary Law 24 of January 7, 1975, shall establish the rules to regulate the matter provisionally.

Paragraph 9. Until a supplementary law provides for the matter, electric power distribution companies, as taxpayers or substitute taxpayers, shall be liable, when the product leaves their facilities, even if the destination is another federal entity, for the payment of the tax on the circulation of goods levied on electric power, from production or importation to the last operation, such tax being calculated on the price charged on the occasion

of the final operation, its collection being ensured to the state or the Federal District, depending on the place where such operation occurs.

Paragraph 10. Until the law provided by Article 159, item I, subitem *c*, which shall be promulgated by December 31, 1989, enters in force, the application of the funds set forth in that provision shall be ensured in the following manner:

I – sixtenths of one percent in the Northern Region, through the Bank of the Amazon;

II – one and eight-tenths percent in the Northeastern Region, through the Northeastern Bank of Brazil;

III – six-tenths of one percent in the Centre-West Region, through the Bank of Brazil.

Paragraph 11. The Centre-West Development Bank is hereby created, in the manner established by law, in order to comply, within that region, with the provisions of Articles 159, item I, subitem *c*, and 192, paragraph 2, of the Constitution.

Paragraph 12. The urgency provided by Article 148, item II, shall not preclude the collection of the compulsory loan instituted for the benefit of the Brazilian Power Plants corporation (*Eletrobrás*) by Law 4.156 of November 28, 1962, with the subsequent amendments.

Article 35. The provisions of Article 165, paragraph 7, shall be complied with progressively, over a period of ten years, the funds being distributed among the macroeconomic regions in proportion to their population, based on the situation verified for the 1986-87 period.

Paragraph 1. In the application of the criteria referred to in this Article, the total expenses shall exclude expenses for:

I – projects considered as priorities in the multiyear plan;

II – national security and defense;

III – maintenance of the federal bodies in the Federal District;

IV – the National Congress, the Federal Accounting Court and the judiciary;

V – the servicing of the debt of the government bodies and associated entities, including foundations instituted and maintained by the federal government.

Paragraph 2. Until the supplementary law referred to in Article 165, paragraph 9, items I and II, comes into force, the following rules shall be complied with:

I – the project of the multiyear plan, to be in force until the end of the first fiscal year of the subsequent presidential term of office, shall be forwarded not less than four months before the end of the first fiscal year and returned for sanction before the end of the legislative session;

II – the bill of budget directives shall be forwarded not less than eight and a half months before the end of the fiscal year and returned for sanction before the end of the first period of the legislative session;

III – the budget bill of the Union shall be forwarded not less than four months before the end of the fiscal year and returned for sanction before the end of the legislative session.

Article 36. The funds existing on the day the Constitution is promulgated, except for those resulting from tax exemptions which become private property and those which are of interest to national defense, shall be extinguished if they are not ratified by the National Congress within two years.

Article 37. Adaptation to the provisions of Article 167, item III, shall be made within the period of five years, the excess being reduced at a rate of at least one fifth per year.

Article 38. Until the promulgation of the supplementary law referred to in Article 169, the Union, states, Federal District and municipalities shall not spend more than sixty-five percent of the amount of the respective current revenues on personnel.

Single paragraph. The Union, states, Federal District and municipalities, whenever the respective expenditure with personnel exceeds the limit established in this Article, shall return to such limit, reducing the excess percentage at a rate of one fifth per year.

Article 39. For purposes of compliance with the constitutional provisions which involve variations of expenses and revenues of the Union, after the promulgation of the Constitution, the executive branch shall elaborate and the legislative branch shall examine a bill of review of the budget law referring to the fiscal year of 1989.

Single paragraph. The National Congress shall vote within twelve months the supplementary law provided by Article 161, item II.

Article 40. The Free-Trade Zone of Manaus, with its characteristics of free-trade, export and import and fiscal benefits, shall be maintained for a period of twenty-five years as from the promulgation of the Constitution.

Single paragraph. The criteria which regulated or may come to regulate the approval of projects in the Free-Trade Zone of Manaus may only be modified by a federal law.

Article 41. The executive branches of the Union, states, Federal District and municipalities shall reassess all sectorial tax incentives now in force and shall propose the appropriate measures to the respective legislative branches.

Paragraph 1. The incentives which are not confirmed by law within two years of the promulgation of the Constitution shall be considered repealed.

Paragraph 2. Revocation shall not preclude any rights which have become vested before that date, in relation to incentives granted under conditions and for a set period.

Paragraph 3. Incentives granted by means of agreements concluded between states, in accordance with Article 23, paragraph 6 of the 1967 Constitution, with the wording of Amendment 1, of October 17, 1969, shall also be reassessed and reconfirmed within the time limits set forth in this Article.

Article 42. For 40 (forty) years, the Federation shall allocate the funds intended for irrigation: (CA 89, 2015)

I – 20% (twenty percent) for the Central-West Region; (CA 89, 2015)

II – 50% (fifty percent) for the Northeast Region, preferably in the semiarid region. (CA 89, 2015)

Single paragraph. Of the percentages provided for in items I and II of the head of the Article, a minimum of 50% (fifty percent) will be allocated to projects of irrigation that benefit family farming that meets the requirements provided for in specific legislation. (CA 89, 2015)

Article 43. On the date of the promulgation of the law regulating the prospecting and mining of mineral resources and beds of ore, or within one year counted from the date of the promulgation of the Constitution, the authorizations, grants and other deeds over mining rights shall become ineffective, in case the prospecting or mining works have not provenly started in the legal time limits or are inactive.

Article 44. Brazilian companies which presently hold valid prospecting authorizations and permits for the mining of mineral resources and the exploitation of hydraulic energy shall have four years, counted from the date of the promulgation of the Constitution, to comply with the requirements of Article 176, paragraph 1.

Paragraph 1. Except for the provisions of national interest set forth in the constitutional text, Brazilian companies shall be exempt from compliance with the provisions of Article 176, paragraph 1, provided that, within four years counted from the date of promulgation of the Constitution, they have destined the product of their mining and processing activities to industrialization within the national territory, in their own facilities or in a controlling or controlled industrial company.

Paragraph 2. Brazilian companies which hold a hydraulic energy concession for use in their industrial processes shall also be exempted from compliance with the provisions of Article 176, paragraph 1.

Paragraph 3. The Brazilian companies referred to in paragraph 1 may only be granted prospecting authorizations or concessions to mine or exploit hydraulic energy potentials provided that the energy and the mining product are used in their respective industrial processes.

Article 45. Refineries which operate in the country under Article 43 and under the conditions of Article 45 of Law 2,004 of October 3, 1953, are excluded from the monopoly established by Article 177, item II, of the Constitution.

Single paragraph. Contracts entered into by Brazilian Petroleum corporation (Petrobrás) for petroleum exploration which allocated all risk to the party hired and that were in force on the date of promulgation of the Constitution are exempted from the prohibition of Article 177, paragraph 1.

Article 46. Credits with institutions under intervention or extrajudicial liquidation, even when such proceedings are converted into bankruptcy, are subject to adjustment for inflation from the date of maturity to the date of actual payment, with no interruption or suspension.

Single paragraph. The provisions of this Article shall also apply to:

I – transactions made after the proceedings referred to in the head of this Article have been decreed;

II – loan, financing and refinancing transactions, transactions of financial assistance for liquidity purposes, assignment or subrogation of credits or mortgage bonds, guarantee of deposits made by the public, or of purchase of liabilities, including those carried out with funds intended for such purposes;

III – credits existing prior to the promulgation of this Constitution;

IV – credits held by public administration entities before the promulgation of this Constitution and not settled by January 1, 1988.

Article 47. In the settlement of debts, including their subsequent renegotiation and composition, even when taken to court, arising out of any loans granted by banks and by financial institutions, there shall be no adjustment for inflation, provided that the loan has been granted:

I – to micro and small businessmen or to their businesses in the period from February 28, 1986, to February 28, 1987;

II – to mini, small and medium rural producers in the period from February 28, 1986, to December 31, 1987, if it refers to rural credit.

Paragraph 1. For the purposes of this Article, microenterprises shall be considered as the legal entities and individual firms with annual income of up to ten thousand National Treasury Bonds, and small-sized enterprises as the legal entities and individual firms with annual income of up to twenty-five thousand National Treasury Bonds.

Paragraph 2. Classification as a mini, small or medium rural producer shall be made in accordance with the rural credit rules in force at the time of the contract.

Paragraph 3. Exemption from adjustment for inflation referred to in this Article shall only be granted in the following cases:

I – if the initial debt, plus legal interests and judicial fees, are settled within ninety days of promulgation of this Constitution;

II – if the application of the funds is not contrary to the purpose of the financing, the burden of proof lying with the creditor institution;

III – if the creditor institution does not show that the borrower has the means to pay the debt, such means excluding the business, house and work of the borrower and production instruments;

IV – if the initial financing does not exceed the limit of five thousand National Treasury Bonds;

V – if the beneficiary is not the owner of more than five rural modules.

Paragraph 4. The benefits referred to in this Article shall not be extended to the debts which have already been paid and to debtors who are members of the Constituent Assembly.

Paragraph 5. In the event of transactions due after the deadline for settlement of the debt, should the borrower be interested, the banks and the financial institutions shall propose, by a specific instrument, an amendment to the original conditions of the contract so as to adjust them to this benefit.

Paragraph 6. The granting of this benefit by private commercial banks shall not, under any circumstances, entail a burden to the government, even if made by refinancing funds from the Central Bank.

Paragraph 7. In the case of funds to official financial agents or credit cooperatives, the burden shall fall upon the original source of funds.

Article 48. The National Congress, within one hundred and twenty days of the promulgation of this Constitution, shall draw up a consumer defense code.

Article 49. The law shall provide for the institution of emphyteusis concerning urban real property, the tenants having the option, in the event of extinction, of redemption of the emphyteusis, by acquisition of direct title in accordance with the provisions contained in the respective contracts.

Paragraph 1. In the absence of a contractual clause, the criteria and bases currently in force in the special legislation on real estate of the Union shall be adopted.

Paragraph 2. The rights of presently registered occupants shall be ensured by application of another kind of contract.

Paragraph 3. Emphyteusis shall continue to be applied to tidal lands and those lands added to them, which are located within the security strip extending from the coastline.

Paragraph 4. After redemption of the emphyteusis, the former holder of direct title shall, within ninety days, subject to liability, entrust all documents related to such title to the custody of the competent real estate registry.

Article 50. An agricultural law to be promulgated within one year shall provide, in accordance with this Constitution, for the objectives and instruments of agricultural policy, priorities, crop planning, marketing, internal supply, foreign market and institution of agrarian credit.

Article 51. All donations, sales and concessions of public land with an area of more than three thousand hectares, made in the period from January 1st, 1962, to December 31, 1987, shall be reviewed by the National Congress, by a joint committee, during the three years following the promulgation of the Constitution.

Paragraph 1. Insofar as sales are concerned, the review shall be based exclusively on the criterion of lawfulness of the transaction.

Paragraph 2. In the case of concessions and donations, the review shall comply with the criteria of lawfulness and of convenience of public interest.

Paragraph 3. In the cases set forth in the preceding paragraphs, if illegality is proven or if there is public interest, the lands shall revert to the ownership of the Union, states, Federal District or municipalities.

Article 52. Until such time as the conditions referred to in Article 192 are established, the following are forbidden: (CA 40, 2003)

I – the installation, in the country, of new branches of financial institutions domiciled abroad;

II – the increase in the percentage of participation of people or legal entities resident or domiciled abroad in the capital of financial institutions with headquarters in Brazil.

Single paragraph. The prohibition referred to in this Article does not apply to the authorizations resulting from international agreements, from reciprocity or from interest of the Brazilian government.

Article 53. Veterans who have participated in war operations during the second world war, in accordance with Law 5,315 of September 12, 1967, shall be ensured the following rights:

I – admission to public service without being required to undergo a competitive civil-service examination, with tenure;

II – special pension corresponding to that of Second Lieutenant of the Armed Forces, which may be applied for at any time and may not be accumulated with any other earn-

ings received from the Treasury, except for social security benefits, the right to opt being ensured;

III – in case of death, proportional pension to the widow, companion or dependent, in an amount equal to that of the preceding item;

IV – free medical, hospital and educational assistance extending to dependents;

V – retirement with full pay after twenty-five years of actual service, under any juridical system;

VI – priority in the acquisition of a home for those who do not own one or for their widows or companions.

Single paragraph. The concession of the special pension referred to in item II replaces, for all legal effects, any other pension already granted to the veteran.

Article 54. Rubber tappers recruited in accordance with Decree Law nº 5,813 of September 14, 1943, and protected by Decree Law nº 9,882 of September 16, 1946, shall receive, when needy, a monthly pension for life in the amount of two minimum wages.

Paragraph 1. The benefit extends to rubber tappers who, at the request of the Brazilian government, contributed to the war effort by working in rubber production in the Amazonian Region during the second world war.

Paragraph 2. The benefits established in this Article may be transferred to dependents who are provenly needy.

Paragraph 3. The concession of the benefit shall be done in accordance with the law to be proposed by the Executive branch within one hundred and fifty days of the promulgation of the Constitution.

Article 54-A. The rubber tappers referred to in Article 54 of this Temporary Constitutional Provisions Act shall receive a compensation, in a single instalment, of R$ 25,000.00 (twenty-five thousand reals). (CA 78, 2014)

Article 55. Until such time as the budget directives law is approved, at least thirty percent of the social welfare budget, excluding unemployment insurance, shall be allocated to the health sector.

Article 56. Until such time as the law regulates Article 195, item I, the revenues resulting from at least five of the six tenths of one percent corresponding to the rate of the contribution referred to in Decree Law nº 1,940 of May 25, 1982, as amended by Decree Law nº 2,049 of August 1, 1983, by Decree nº 91,236 of May 8, 1985, and by Law nº 7,611 of July 8, 1987, shall become part of the social welfare revenues, excepting, exclusively in the fiscal year of 1988, commitments assumed for ongoing programs and projects.

Article 57. The debts of the states and municipalities related to social security contributions up to June 30, 1988, shall be settled, with adjustment for inflation, in one hundred and twenty monthly instalments, with the waiver of the interests and penalties applicable thereto, provided the debtors request instalment payment and begin such payment within one hundred and eighty days of the promulgation of this Constitution.

Paragraph 1. The amount to be paid in each of the first two years shall not be less than five percent of the total consolidated and updated debt, the balance to be divided into equal monthly instalments.

Paragraph 2. Settlement may include payments by assignment of assets and rendering of services, as set forth in Law nº 7,578 of December 23, 1986.

Paragraph 3. As guarantee for the payment of the instalments, the states and municipalities shall each year include in their respective budgets the appropriations required for the payment of their debts.

Paragraph 4. If any of the conditions established for the concession of instalment payment are not met, the debt shall be considered as due and payable in full and liable for default interest; in such case, the portion of the funds corresponding to the Participation Funds intended for the debtor states and municipalities shall be blocked and transferred to the social security for payment of their debts.

Article 58. Benefits paid on a continuous basis and maintained by social security on the date of the promulgation of the Constitution shall have their values reviewed so as to reestablish their purchasing power expressed in terms of the numbers of minimum wages they represented on the date on which they were granted, such updating criterion to be adopted until the plan of funding and benefits referred to in the following Article is implemented.

Single paragraph. The monthly benefit payments updated in accordance with this Article shall be due and paid as from the seventh month after the promulgation of the Constitution.

Article 59. The bills of law for the organization of social welfare and for the plan of funding and benefits shall be submitted, not more than six months after the promulgation of the Constitution, to the National Congress, which shall have six months to examine them.

Single paragraph. Upon approval by the National Congress, the plans shall be implemented progressively in the following eighteen months.

Article 60. In the 14 (fourteen) years following the promulgation of this Constitutional Amendment, the states, Federal District, and municipalities shall allocate a portion of the funds referred to in the head of Article 212 of the Federal Constitution, to the main-

tenance and development of basic education and to the payment of appropriate salaries to education workers, with due regard for the following provisions: (CA 53, 2006)

I – the distribution of resources and responsibilities among the Federal District, states, and their municipalities is assured through the establishment, within each state and the Federal District, of a Fund for the Maintenance and Development of Basic Education and for the Appreciation of Education Professionals (FUNDEB), of a financial nature; (CA 53, 2006)

II – the Funds referred to in item I of the head of this Article shall be made up of 20% (twenty percent) of the resources referred to in items I, II, and III of Article 155; item II of the head of Article 157; items II, III, and IV of the head provision of Article 158; and subitems *a* and *b* of item I, and item II of the head of Article 159, of the Federal Constitution, and shall be distributed among each state and its municipalities, in proportion to the number of students in the various grades and modalities of on-site basic education, enrolled in the respective school systems, within the respective scope of priority action as established by paragraphs 2 and 3 of Article 211 of the Federal Constitution; (CA 53, 2006)

III – with due regard for the guarantees established in items I, II, III, and IV of the head of Article 208 of the Federal Constitution, as well as for the basic education universalization goals established in the National Education Plan, the law shall provide for: (CA 53, 2006)

a) the organization of the Funds, the proportional distribution of their resources, the differences and weightings regarding the annual value per student among the various grades and modalities of basic education and types of schools; (CA 53, 2006)

b) the form of calculation of the minimum annual value per student; (CA 53, 2006)

c) the maximum percentages for the allocation of fund resources to the various grades and modalities of basic education, with due regard for Articles 208 and 214 of the Federal Constitution, as well as for the National Education Plan goals; (CA 53, 2006)

d) oversight and control of the Funds; (CA 53, 2006)

e) a deadline to stipulate, by means of a specific law, a nationwide professional minimum salary for public school teachers of basic education; (CA 53, 2006)

IV – the resources transferred to the Funds established under the terms of item I of the head of this Article shall be applied by the states and municipalities exclusively within the scope of their priority actions, as established by paragraphs 2 and 3 of Article 211 of the Federal Constitution; (CA 53, 2006)

V – the federal government shall supplement the resources of the Funds referred to in item II of the head of this Article, whenever in the Federal District and in each state, the value per student does not reach the nationally set minimum value, stipulated in accordance with

the provisions of item VII of the head of this Article, and use of the resources referred to in paragraph 5 of Article 212 of the Federal Constitution is forbidden; (CA 53, 2006)

VI – up to 10% (ten percent) of the resources supplemented by the Union as set forth in item V of the head of this Article may be distributed to the Funds by means of programs aimed at improving the quality of education, under the terms of law referred to in item III of the head of this Article; (CA 53, 2006)

VII – the minimum amount of resources supplemented by the Union as set forth in item V of the head of this Article shall be equal to: (CA 53, 2006)

a) R$ 2,000,000,000.00 (two billion reals), in the first year the Funds are in force; (CA 53, 2006)

b) R$ 3,000,000,000.00 (three billion reals), in the second year the Funds are in force; (CA 53, 2006)

c) R$ 4,500,000,000.00 (four billion and five hundred million reals), in the third year the Funds are in force; (CA 53, 2006)

d) 10% (ten percent) of the total amount of resources referred to in item II of the heading of this Article, as from the fourth year the Funds are in force; (CA 53, 2006)

VIII – the resources earmarked for the maintenance and development of education as established in Article 212 of the Union may cover a maximum amount of 30% (thirty percent) of the resources supplemented by the Union, taking into consideration, for the purposes of this item, the amounts set forth in item VII of the head provision; (CA 53, 2006)

IX – the amounts referred to in subitems *a*, *b*, and *c* of item VII of the head provision shall be adjusted every year from the promulgation of this Constitutional Amendment, so that the real value of the supplementation provided by the Union is permanently preserved; (CA 53, 2006)

X – the supplementation provided by the Union shall comply with the provisions of Article 160 of the Federal Constitution; (CA 53, 2006)

XI – the competent authority shall be held liable for crime of responsibility in case of non-compliance with the provisions of items V and VII of the head provision; (CA 53, 2006)

XII – a share of not less than 60% (sixty percent) of the resources of each Fund referred to in item I of the head provision shall be used for the payment of basic education teachers who are actively engaged in the practice of teaching. (CA 53, 2006)

Paragraph 1. When financing basic education, the Union, states, Federal District, and municipalities shall ensure that the quality of education will be improved, to guarantee a nationally set minimum standard. (CA 53, 2006)

Paragraph 2. The value per elementary school student, within each state Fund and the Federal District Fund, may not be lower than the value prescribed by the Fund for the Maintenance and Development of Elementary Education and for the Appreciation of the Teaching Profession (FUNDEF), in the year preceding the coming into force of this Constitutional Amendment. (CA 53, 2006)

Paragraph 3. The minimum annual value per elementary school student, within the Fund for the Maintenance and Development of Basic Education and for the Appreciation of Education Professionals (FUNDEB), may not be lower than the minimum value stipulated for the entire country in the year preceding the year in which this Constitutional Amendment comes into force. (CA 53, 2006)

Paragraph 4. For the purposes of distribution of the resources of the Funds referred to in item I of the head provision, the total number of students enrolled in elementary education will be taken into account, and, regarding early childhood education, high school, and the education of young people and adults, 1/3 (one third) of the total number of students enrolled in the first year, 2/3 (two thirds) in the second year, and the total number from the third year shall be taken into consideration. (CA 53, 2006)

Paragraph 5. The percentage of resources to constitute the Funds, in accordance with item II of the head of this Article, shall be gradually achieved over the first 3 (three) years the Funds are in force, as follows: (CA 53, 2006)

I – as to the taxes and transfers mentioned in item II of the heading of Article 155; item IV of the heading of Article 158; and subitems *a* and *b* of item I and item II of the head of Article 159 of the Federal Constitution: (CA 53, 2006)

a) 16.66% (sixteen and sixty-six hundredths of one percent), in the first year; (CA 53, 2006)

b) 18.33% (eighteen and thirty-three hundredths of one percent), in the second year; (CA 53, 2006)

c) 20% (twenty percent), as from the third year; (CA 53, 2006)

II – as to the taxes and transfers mentioned in items I and III of the heading of Article 155; item II of the heading of Article 157; and items II and III of the heading of Article 158 of the Federal Constitution: (CA 53, 2006)

a) 6.66% (six and sixty-six hundredths of one percent), in the first year; (CA 53, 2006)

b) 13.33% (thirteen and thirty-three hundredths of one percent), in the second year; (CA 53, 2006)

c) 20% (twenty percent), from the third year. (CA 53, 2006)

Paragraph 6. (Repealed) (CA 53, 2006)

Paragraph 7. (Repealed) (CA 53, 2006)

Article 61. The educational entities referred to in Article 213, as well as the educational and research foundations whose creation has been authorized by law, which meet the requirements of items I and II of such Article and which have, in the last three years, received public funds, may continue to receive such funds, unless otherwise established by law.

Article 62. The law shall create the National Rural Apprenticeship Service (SENAR), based on the legislation for the National Industrial Apprenticeship Service (SENAI), and the National Commercial Apprenticeship Service (SENAC), without prejudice to the incumbencies of the government bodies engaged in the area.

Article 63. A committee composed of nine members is hereby created, three from the legislative branch, three from the judiciary and three from the executive branch, to promote the commemorations of the centennial of the proclamation of the Republic and of the promulgation of the first republican Constitution of the country, and such committee may, at its discretion, be subdivided into as many subcommittees as may be necessary.

Single paragraph. In carrying out its duties the committee shall conduct studies, debates and assessments of the political, social, economic and cultural development of the country, and may join efforts with state and municipal governments and with public and private institutions desiring to take part in the events.

Article 64. The national press and other printing departments of the Union, states, Federal District and municipalities, of the direct bodies and associated entities, including foundations instituted and maintained by the government, shall provide for a popular edition of the full text of the Constitution, which shall be made available free of charge, to schools and public registry offices, to unions, military barracks, churches and other community organizations, in order that each Brazilian citizen may receive from the State a copy of the Brazilian Constitution.

Article 65. The legislative branch shall, within twelve months, regulate the Article 220, paragraph 4.

Article 66. The public telecommunications utility concessions presently in force shall be maintained, as established by law.

Article 67. The Union shall conclude the demarcation of the Indian lands within five years of the promulgation of the Constitution.

Article 68. Final ownership shall be recognized for the remaining members of the ancient runaway slave communities who are occupying their lands and the State shall grant them the respective title deeds.

Article 69. The states shall be allowed to maintain legal consultancy offices independent from their General Counsels Offices or Attorney General Offices, if they have separate agencies for the respective functions on the date of promulgation of this Constitution.

Article 70. The present competence of the Court of Appeal shall be maintained until it is defined in the State Constitution, as established in Article 125, paragraph 1, of the Constitution.

Article 71. The Emergency Social Fund is hereby instituted for the fiscal years of 1994 and 1995, as well as for the periods from January 1st, 1996 through June 30, 1997, and from July 1st, 1997 through December 31, 1999, aiming at the financial recuperation of the Federal Public Finances and the economic stabilization, the resources of which shall be applied primarily to the actions of the health and education systems, including the supplementation of resources set forth in paragraph 3 of Article 60 of the Temporary Constitutional Provisions Act, the welfare benefits and welfare assistance of a permanent nature, including the payment of welfare debts and budget expenditures associated to programs of great economic and social interest. (CA 17, 1997)

Paragraph 1. The provision of the final part of item II of paragraph 9 of Article 165 of the Constitution shall not apply to the Fund established by this Article. (CA 10, 1996)

Paragraph 2. From the beginning of the 1996 fiscal year on, the Fund established by this Article shall be called Fiscal Stabilization Fund. (CA 10, 1996)

Paragraph 3. The Executive branch shall publish, on a bimonthly basis, a budget execution statement, which shall list the sources and applications of the Fund established by this Article. (CA 10, 1996)

Article 72. The Emergency Social Fund is comprised of: (RCA 1, 1994)

I – the proceeds from the collection of the tax on income and earnings of any nature to be levied at source on payments of any nature made by the Union, including its autonomous government agencies and foundations; (RCA 1, 1994)

II – the part of the proceeds from the collection of the tax on income and earnings of any nature, and of the tax on credit, foreign exchange and insurance transactions, or transactions relating to bonds and securities, resulting from the changes generated by Law nº 8,894 of June 21, 1994, and by Laws nº 8,849 and nº 8,848, both dated January 28, 1994 and further modifications; (CA 10, 1996)

III – the part of the proceeds from the collection due to the increase of the rate of welfare contribution on the profit of taxpayers mentioned in paragraph 1 of Article 22 of Law nº 8,212 of July 24, 1991, which, in the fiscal years of 1994 and 1995, as well as in the period from January 1, 1996 through June 30, 1997, shall be of 30 percent, subject to

modification by ordinary law, the other stipulations of Law nº 7,869 of December 15, 1988 remaining unchanged; (CA 10, 1996)

IV – twenty percent of the proceeds from the collection of all taxes and contributions to the Union, already instituted or to be instituted, except those provided by items I, II and III, with due regard to the provisions of paragraphs 3 and 4; (CA 10, 1996)

V – the part of the proceeds from the collection of the contribution mentioned in Supplementary Law nº 7, of September 7, 1970, owed by the legal entities referred to in item III of this Article, which will be calculated, in the fiscal years of 1994 and 1995, as well as in the periods from January 1st, 1996 through June 30, 1997, and from July 1st, 1997 through December 31, 1999, through the employment of a rate of seventy-five hundredths of one percent, subject to modification by subsequent ordinary law, on the gross operating income, as defined in the legislation of income tax and earnings of any nature; (CA 17, 1997)

VI – other incomes defined in specific legislation. (RCA 1, 1994)

Paragraph 1. The rates and calculation base defined in items III and V shall be applied as from the first day of the month following the ninetieth day after the promulgation of this Amendment. (RCA 1, 1994)

Paragraph 2. The parts referred to in items I, II, III and V shall be previously deducted from the calculation base of any legal or constitutional designation or participation, and the provisions of Articles 159, 212 and 239 of the Constitution shall not apply to them. (CA 10, 1996)

Paragraph 3. The part referred to in item IV shall be previously deducted from the calculation base of any constitutional or legal designation or participation stipulated by Articles 153, paragraph 5, 157, item II, 212 and 239 of the Constitution. (CA 10, 1996)

Paragraph 4. The provision of the former paragraph shall not apply to the resources provided by Articles 158, item II, and 159 of the Constitution. (CA 10, 1996)

Paragraph 5. The part of the resources originating from the tax on income and earnings of any nature, designated for the Emergency Social Fund, as provided by item II of this Article, shall not exceed five and six-tenths of one percent of the total proceeds from its collection. (CA 10, 1996)

Article 73. In the regulation of the Emergency Social Fund, the instrument provided by item V of Article 59 of the Constitution may not be applied. (RCA 1, 1994)

Article 74. The Union may establish provisional contribution on the movement or transmission of funds and of credits and rights of financial nature. (CA 12, 1996)

Paragraph 1. The rate of the contribution mentioned in this Article shall not exceed twenty-five hundredths of one percent, and the executive branch may reduce it or reestablish it, in whole or in part, in the conditions and limits provided for by law. (CA 12, 1996)

Paragraph 2. The provisions of Articles 153, paragraph 5, and 154, item I, of the Constitution shall not apply to the contribution mentioned in this Article. (CA 12, 1996)

Paragraph 3. The total amount of the proceeds from the collection of the contribution mentioned in this Article shall be allocated to the National Health Foundation to finance health actions and services. (CA 12, 1996)

Paragraph 4. The liability for the contribution mentioned in this Article shall be governed by the provisions of Article 195, paragraph 6, of the Constitution, and it shall not be collected for longer than two years. (CA 12, 1996)

Article 75. The collection of the provisional contribution on the movement or transmission of funds and of credits and rights of financial nature mentioned in Article 74, established by Law nº 9,311, of October 24, 1996, is extended for thirty-six months, and the same extension applies to the enforcement of Law nº 9,539, of December 12, 1997, which modified Law nº 9,311. (CA 21, 1999)

Paragraph 1. With due regard for paragraph 6 of Article 195 of the Federal Constitution, the rate of the contribution shall be thirty-eight hundredths of one percent, in the first twelve months, and thirty hundredths in the subsequent months, and the executive branch may reduce it, in whole or in part, in the limits hereby stipulated. (CA 21, 1999)

Paragraph 2. The proceeds from increased collection of the contribution, resulting from the alteration of the rate, during the financial years of 1999, 2000, and 2001, shall be allocated to the financing of social security. (CA 21, 1999)

Paragraph 3. The Union is authorized to issue domestic public debt bonds, whose resources shall be allocated to the financing of health services and social security, in an amount equivalent to the proceeds of the collection of the contribution, estimated but not achieved in 1999. (CA 21, 1999)

Article 76. Until December 31, 2023, 30% (thirty percent) of the Union collection with social contributions, contributions for intervention in the economic domain, and fees, already instituted or instituted by the referred date, shall not be earmarked to any government body, fund or expense, irrespective of expenses with the General social security regime. (CA 93, 2016)

Paragraph 1. (Repealed) (CA 93, 2016)

Paragraph 2. The proceeds from the collection of the social contribution on employee-wage for education mentioned in paragraph 5 of Article 212 of the Federal Constitution shall be excepted from the provision of the head of this Article. (CA 68, 2011)

Paragraph 3. (Repealed) (CA 93, 2016)

Paragraph 4. The detachment established under the head of this Article does not apply on revenues from social contributions earmarked to fund the social welfare. (CA 103, 2019)

Article 76-A. Until December 31, 2023, 30% (thirty percent) of the states' collection and that of the Federal District with taxes, fees, and fines, already instituted or instituted by the referred date, as well as their surcharges, legal increments and other current receipts, shall not be earmarked to any government body, fund, or expense. (CA 93, 2016)

Single paragraph. The following are excepted from the precept dealt with in the head provision: (CA 93, 2016)

I – receipts appropriated to fund public health activities and services and to maintain and develop education dealt with respectively under items II and III of paragraph 2 of Article 198 and Article 212 of the Federal Constitution; (CA 93, 2016)

II – revenues of the municipalities from the remittance provided for in the Federal Constitution; (CA 93, 2016)

III – revenues from social security contributions and health assistance from servants; (CA 93, 2016)

IV – other mandatory or voluntary remittances among federal entities provided by law to a specified allocation; (CA 93, 2016)

V – funds instituted by the judiciary, Accounting Courts, Prosecution Office, Office of the Public Defender, and prosecutors of the states and the Federal District. (CA 93, 2016)

Article 76-B. Until December 31, 2023, 30% (thirty percent) of the municipalities' revenues from taxes, fees and fines, already instituted or instituted by the referred date, as well as their surcharges, legal increments and other current revenues, shall not be earmarked to any government body, fund, or expense. (CA 93, 2016)

Single paragraph. The following are excepted from the earmark dealt with in the head provision: (CA 93, 2016)

I – resources allocated to finance public health actions and services and to maintain and develop education programs dealt with respectively under items II and III of paragraph 2 of Article 198 and Article 212 of the Federal Constitution; (CA 93, 2016)

II – receipts from social security contributions and health assistance from servants; (CA 93, 2016)

III – mandatory or voluntary remittance among federal entities provided by law to a specified allocation; (CA 93, 2016)

IV – funds instituted by the Municipal Accounting Court. (CA 93, 2016)

Article 77. Until the fiscal year 2004, the minimum amount of funds applied to health activities and public services shall be equivalent to: (CA 29, 2000)

I – in the case of the Union: (CA 29, 2000)

a) in the year 2000, the amount of checks issued to health activities and public services during the fiscal year 1999, plus at least five percent; (CA 29, 2000)

b) from the year 2001 through the year 2004, the amount expended in the previous year, restated according to the nominal changes of the Gross Domestic Product – GDP; (CA 29, 2000)

II – in the case of the states and of the Federal District, twelve percent of the proceeds from the tax collection referred to in Article 155 and of the funds mentioned in Articles 157 and 159, item I, subitem *a*, and item II, after deducting the portions transferred to the respective municipalities; (CA 29, 2000)

III – in the case of the municipalities and of the Federal District, fifteen percent of the proceeds from the tax collection mentioned in Article 156 and of the funds mentioned in Articles 158 and 159, item I, subitem *b*, and paragraph 3. (CA 29, 2000)

Paragraph 1. The states, Federal District, and municipalities which apply percentages lower than those stipulated in items II and III shall raise them gradually, until the fiscal year 2004, the difference being reduced at the rate of at least one fifth per year, and the application shall consist of at least seven percent as of the year 2000. (CA 29, 2000)

Paragraph 2. At least fifteen percent of the Union funds expended under the terms of this Article shall be applied in the municipalities, according to the populational criterion, to health activities and public services, in accordance with the law. (CA 29, 2000)

Paragraph 3. The funds of the states, Federal District, and municipalities assigned for health activities and public services, as well as those transferred by the Union for the same purpose, shall be applied by means of the Health Fund, to be monitored and supervised by the Health Board, without prejudice to the provisions of Article 74 of the Federal Constitution. (CA 29, 2000)

Paragraph 4. In the absence of the supplementary law referred to in Article 198, paragraph 3, the provisions of this Article shall apply to the Union, states, Federal District, and municipalities as of the fiscal year 2005. (CA 29, 2000)

Article 78. Except for credits defined by law as being of small amount, those for alimony, and those referred to in Article 33 of this Temporary Constitutional Provisions Act and their supplementations, as well as those credits whose respective funds have already been released or paid into court, the court order debts for which payment is outstanding on the date of promulgation of this Amendment and those stemming from actions filed by December 31st, 1999, shall be settled in currency according to their real value includ-

ing legal interests, in equal and successive annual instalments, within ten years at the most, the assignment of credits being permitted. (CA 30, 2000)

Paragraph 1. The division of instalments is permitted, at the discretion of the creditor. (CA 30, 2000)

Paragraph 2. In the event the annual instalments referred to in the head provision have not been paid before the end of the relevant fiscal year, they shall be deducted from taxation owed to the debtor entity. (CA 30, 2000)

Paragraph 3. The period referred to in the head provision is reduced to two years, in the case of court order debts deriving from the expropriation of a creditor's residential property, provided that such property is proven to be the creditor's only residential property at the time of emission of a writ of ejectment. (CA 30, 2000)

Paragraph 4. If the time limit has elapsed, or in the case of omission in the budget, or in the event the right of precedence is not respected, the president of the appropriate court shall, upon petition of a creditor, requisition or order the seizure of funds of the debtor entity, at an amount sufficient to pay the instalment. (CA 30, 2000)

Article 79. The Fund to Fight and Eradicate Poverty, hereby instituted within the sphere of the Federal Executive branch, shall be in force through the year 2010 and shall be regulated by a supplementary law, aiming at enabling all Brazilians to have access to adequate subsistence levels, and its resources shall be applied to supplementary initiatives regarding nutrition, housing, education, health, a complementary family income, and other programs of relevant social interest oriented towards the improvement of quality of life. (CA 31, 2000)

Single paragraph. The Fund set forth in this Article shall have an advisory and monitoring board that must include representatives of civil society, under the terms of law. (CA 31, 2000)

Article 80. The Fund to Fight and Eradicate Poverty is comprised of: (CA 31, 2000)

I – the part of the proceeds from the collection corresponding to additional eight hundredths of one percent, applicable from June 18, 2000, through June 17, 2002, to the rate of the social contribution referred to in Article 75 of the Temporary Constitutional Provisions Act; (CA 31, 2000)

II – the part of the proceeds from the collection corresponding to additional five percent on the rate of the federal VAT [IPI], or of the tax that may eventually replace it, levied on luxury goods and applicable while the Fund is in force; (CA 31, 2000)

III – the proceeds from the tax collection referred to in Article 153, item VII, of the Constitution; (CA 31, 2000)

IV – budget appropriations; (CA 31, 2000)

V – donations, of any nature, by people or corporations established in Brazil or abroad; (CA 31, 2000)

VI – other revenues, to be defined by the legislation that regulates the Fund. (CA 31, 2000)

Paragraph 1. The provisions of Articles 159 and 167, item IV, of the Constitution are not applicable to the resources that make up the Fund, neither is any disconnection of budget resources. (CA 31, 2000)

Paragraph 2. The proceeds from the collection of the resources referred to in item I of this Article, during the period from June 18, 2000 through the date the supplementary law mentioned in Article 79 enters in force, shall be remitted in full to the Fund, their real value being preserved, in federal government securities, progressively redeemable after June 18, 2002, under the terms of law. (CA 31, 2000)

Article 81. A Fund is hereby instituted, to be comprised of the resources received by the Union as a result of privatization of mixed-capital companies and state-owned companies controlled directly or indirectly by the federal government, when such operation involves the divestment of the respective controlling interest to an individual or entity not belonging to the government bodies, or of any remaining equity interest following such divestment, and the income thereof, generated as from June 18, 2002, shall be transferred to the Fund to Fight and Eradicate Poverty. (CA 31, 2000)

Paragraph 1. In case the yearly amount of income to be transferred to the Fund to Fight and Eradicate Poverty, as set forth in this Article, does not add up to the total of four billion reals, it shall be supplemented according to Article 80, item IV, of the Temporary Constitutional Provisions Act. (CA 31, 2000)

Paragraph 2. Without prejudice to the provision of paragraph 1, the Executive branch may allocate other revenues deriving from the sale of Union assets to the Fund mentioned in this Article. (CA 31, 2000)

Paragraph 3. The resources that make up the Fund referred to in the head provision, the transfer of said resources to the Fund to Fight and Eradicate Poverty, and the other provisions concerning paragraph 1 of this Article shall be regulated by law, and the provision of Article 165, paragraph 9, item II, of the Constitution shall not be applicable. (CA 31, 2000)

Article 82. The states, Federal District, and municipalities shall institute funds to fight poverty, comprised of the resources referred to in this Article and other resources that may eventually be allocated for this purpose, and the referred to funds shall be managed by entities which include the participation of civil society. (CA 31, 2000)

Paragraph 1. With a view to financing the State Funds and the Federal District Fund, an additional tax of up to two percent may be created, to raise the rate of the State VAT (ICMS), due on luxury goods and services and observing the conditions defined in the supplementary law referred to in Article 155, paragraph 2, item XII, of the Constitution, and the provision of Article 158, item IV, of the Constitution shall not be applicable to such percentage. (CA 42, 2003)

Paragraph 2. With a view to financing the municipal funds, an additional tax of up to half of one percent may be created, to raise the rate of the local service tax [ISS], or the rate of the tax that may eventually replace it, levied on luxury services. (CA 31, 2000)

Article 83. A federal law shall define the luxury goods and services referred to in Articles 80, item II, and 82, paragraph 2. (CA 42, 2003)

Article 84. The provisional contribution on the movement or transmission of funds and of credits and rights of a financial nature, set forth in Articles 74, 75, and 80, item I, of this Temporary Constitutional Provisions Act, shall be collected through December 31, 2004. (CA 37, 2002)

Paragraph 1. The effect of Law 9,311, of October 24, 1996, as well as of its alterations, is hereby extended through the date mentioned in the head provision. (CA 37, 2002)

Paragraph 2. Of the proceeds from collection of the social contribution mentioned in this Article, the portion corresponding to the following rates shall be allocated to the purposes herein stated: (CA 37, 2002)

I – twenty hundredths of one percent to the National Health Fund, for the financing of health activities and services; (CA 37, 2002)

II – ten hundredths of one percent to the financing of social security; (CA 37, 2002)

III – eight hundredths of one percent to the Fund to Fight and Eradicate Poverty, set forth in Articles 80 and 81 of this Temporary Constitutional Provisions Act. (CA 37, 2002)

Paragraph 3. The rate of the contribution mentioned in this Article shall be equal to: (CA 37, 2002)

I – thirty-eight hundredths of one percent in the fiscal years 2002 and 2003. (CA 37, 2002)

II – (Repealed) (CA 42, 2003)

Article 85. The contribution mentioned in Article 84 of this Temporary Constitutional Provisions Act shall not be levied, from the thirtieth day after the publication of this Constitutional Amendment, on entries concerning: (CA 37, 2002)

I – current deposit accounts especially opened and exclusively used for transactions carried out by: (CA 37, 2002)

a) clearinghouses and providers of clearing and settlement services referred to in Article 2, single paragraph, of Law nº 10,214, of March 27, 2001; (CA 37, 2002)

b) securitization companies referred to in Law nº 9,514, of November 20, 1997; (CA 37, 2002)

c) business corporations whose exclusive purpose is to purchase credits originating from transactions carried out in the financial market; (CA 37, 2002)

II – current deposit accounts, when such entries are related to: (CA 37, 2002)

a) stock purchase and sale transactions, made within stock exchange trading floors or electronic systems, and in the organized over the counter market; (CA 37, 2002)

b) contracts written on stocks or stock indices, in their various modes, negotiated in stock exchanges, commodities and futures exchanges; (CA 37, 2002)

III – foreign investors' accounts, regarding entries into and remittances from Brazil of funds employed exclusively in transactions and contracts referred to in item II of this Article. (CA 37, 2002)

Paragraph 1. The executive branch shall regulate the provisions of this Article within thirty days as of the date of publication of this Constitutional Amendment. (CA 37, 2002)

Paragraph 2. The provisions of item I of this Article apply only to the transactions specified in an act issued by the executive branch, from among the transactions that constitute the purpose of the entities referred to. (CA 37, 2002)

Paragraph 3. The provisions of item II of this Article apply only to transactions and contracts made through financial institutions, securities brokerage houses, securities distribution companies, and commodities brokerage houses. (CA 37, 2002)

Article 86. Debts that must be paid by the federal, state, Federal District, or municipal tax authorities due to final and unappealable judicial decisions shall be paid in accordance with the provisions of Article 100 of the Federal Constitution, the parceling rule established in the head provision of Article 78 of this Temporary Constitutional Provisions Act not being applicable, if such debts meet the following cumulative conditions: (CA 37, 2002)

I – having been the subject of a court order; (CA 37, 2002)

II – having been defined as small amount debts by the law referred to in paragraph 3 of Article 100 of the Federal Constitution, or by Article 87 of this Temporary Constitutional Provisions Act; (CA 37, 2002)

III – their payment being outstanding, in whole or in part, on the date of publication of this Constitutional Amendment. (CA 37, 2002)

Paragraph 1. The debts referred to in the head provision, or their respective balances, shall be paid in chronological order of presentation of the respective court orders, with precedence over debts of a higher amount. (CA 37, 2002)

Paragraph 2. If the debts referred to in the head provision have not been subject to partial payment yet, under the terms of Article 78 of this Temporary Constitutional Provisions Act, they may be paid in two annual instalments, as the law provides. (CA 37, 2002)

Paragraph 3. The payment of the alimony debts referred to in this Article, with due respect for the chronological order of their presentation, shall take precedence over the payment of all other debts. (CA 37, 2002)

Article 87. For purposes of the provisions set forth in paragraph 3 of Article 100 of the Federal Constitution, and in Article 78 of this Temporary Constitutional Provisions Act, and until such time as the official publication of the respective defining acts by the federal entities is carried out, the debts or bonds stated in court orders shall be considered as being of a small amount, with due regard for paragraph 4 of Article 100 of the Federal Constitution, if their amount is equal to or lesser than: (CA 37, 2002)

I – forty minimum monthly wages, in the case of debts owed by the tax authorities of the states and of the Federal District; (CA 37, 2002)

II – thirty minimum monthly wages, in the case of debts owed by the tax authorities of the municipalities. (CA 37, 2002)

Single paragraph. Should the amount under execution exceed the amount stipulated in this Article, payment shall always be made by means of a court order, the execution creditor being entitled to waiving the credit of the excess amount, so that one may opt to receive the balance without the emission of a court order, in the manner set forth in paragraph 3 of Article 100. (CA 37, 2002)

Article 88. Until such time as a supplementary law regulates the provisions of items I and III of paragraph 3 of Article 156 of the Federal Constitution, the tax referred to in item III of the head provision of said Article shall: (CA 37, 2002)

I – have a minimum rate of two percent, except for the services referred to in items 32, 33, and 34 of the List of Services appended to Decree Law nº 406, of December 31, 1968; (CA 37, 2002)

II – not be subject to the granting of fiscal exemptions, incentives, and benefits, should the direct or indirect result of such granting be the reduction of the minimum rate stipulated in item I. (CA 37, 2002)

Article 89. Members of the military police and employees of the Federal Territory of Rondônia who were provenly working to the administration at the time it was transformed

into a state; in addition to member of the military police and employees subject to the provisions of Article 36 of supplementary law nº 41, December 22, 1981; and those who were legally included in the government staff of the State of Rondônia by March 15, 1987, when the first elected governor took office; shall be included, at their option, in a special job class to be eventually terminated within the federal government. They are ensured the correspondent rights and advantages and the payment of differences in their wage, under any circumstance, shall be forbidden. (CA 60, 2009)

Paragraph 1. The members military police shall continue working for the State of Rondônia, as detailed personnel, subject to their respective Military Police corporations, with due regard for the compatibility between the duties of their function and their rank in the hierarchy. (CA 60, 2009)

Paragraph 2. The employees referred to in the head provision shall continue working for the State of Rondônia, as detailed personnel, up until they are placed in a federal government body, entity, agency or foundation. (CA 60, 2009)

Article 90. The time limit set forth in the heading of Article 84 of this Temporary Constitutional Provisions Act is hereby extended through December 31, 2007. (CA 42, 2003)

Paragraph 1. The enforcement of Law nº 9,311, of October 24, 1996, as well as of its alterations, is hereby extended through the date mentioned in the head provision. (CA 42, 2003)

Paragraph 2. The rate of the contribution referred to in Article 84 of this Temporary Constitutional Provisions Act shall be equal to thirty-eight hundredths of one percent through the date referred to in the head provision. (CA 42, 2003)

Article 91. The Union shall remit to the states and to the Federal District the amount defined by a supplementary law, in accordance with the criteria, time limits, and terms therein determined, taking into consideration exports of primary commodities and semi manufactured products to other countries, the import-export ratio, credits deriving from purchases intended for the permanent assets, and the effective maintenance and utilization of the tax credits referred to in Article 155, paragraph 2, item X, subitem *a*. (CA 42, 2003)

Paragraph 1. As to the amount of funds to be remitted to each state, seventy-five percent of such amount shall be assigned to the state itself, and twenty-five percent to its municipalities, such percentage being distributed in accordance with the criteria referred to in Article 158, single paragraph, of the Constitution. (CA 42, 2003)

Paragraph 2. The remittance of funds set forth in this Article shall prevail, as defined by supplementary law, until such time as the proceeds from the tax collection referred

to in Article 155, item II, are predominantly assigned, in a proportion not below eighty percent, to the state where consumption of the products, goods, or services takes place. (CA 42, 2003)

Paragraph 3. Until such time as the supplementary law referred to in the head provision is enacted, and so as to replace the system of remittance of funds set forth therein, there shall remain in force the system of remittance of funds set forth in Article 31 and Schedule of Supplementary Law nº 87, of September 13, 1996, with the wording provided by Supplementary Law nº 115, of December 26, 2002. (CA 42, 2003)

Paragraph 4. The states and the Federal District shall present to the federal government, under the terms of instructions issued by the Ministry of Finance, information regarding the tax referred to in Article 155, item II, supplied by the taxpayers who carry out transactions involving goods to be shipped abroad or services to be delivered to foreign parties. (CA 42, 2003)

Article 92. A period of ten years shall be added to the period set forth in Article 40 of this Temporary Constitutional Provisions Act. (CA 42, 2003)

Article 92-A. A period of fifty (50) years shall be added to the period set forth in Article 92 of this Temporary Constitutional Provisions Act. (CA 83, 2014)

Article 93. The provisions of Article 159, item III, and paragraph 4, shall only come into force after the promulgation of the law referred to in said item III. (CA 42, 2003)

Article 94. The special tax regimes for micro and small-sized enterprises which are specific of the Union, states, Federal District, and municipalities shall be discontinued from the date the regime set forth in Article 146, item III, subitem *d*, of the Constitution comes into force. (CA 42, 2003)

Article 95. People born abroad between June 7, 1994, and the date of enactment of this Constitutional Amendment, to a Brazilian father or a Brazilian mother, may be registered with a Brazilian diplomatic or consular authority, or with an official registry if they come to reside in the Federative Republic of Brazil. (CA 54, 2007)

Article 96. Acts aimed at the establishment, fusion, merger, and dismemberment of municipalities, whose act of creation was published by December 31, 2006, are hereby confirmed, provided that the requirements set forth in the legislation of the respective state at the time of establishment of the municipalities referred to have been fulfilled. (CA 57, 2008)

Article 97. Up until the supplementary law referred to in paragraph 15 of Article 100 of the Federal Constitution is enacted, the states, Federal District, and municipalities which, on the date of enactment of Constitutional Amendment 62, have not yet made

payments of past due court-ordered debts regarding their respective government bodies and associated entities, including court orders issued during the period the special regime instituted by this Article is in force, shall make such payments in accordance with the rules set forth in this Article, whereas the provisions of Article 100 of this Federal Constitution shall not be applicable, except for its paragraphs 2, 3, 9, 10, 11, 12, 13, and 14, and without prejudice to agreements already formalized by the date of publication of Constitutional Amendment 62. (CA 62, 2009)

Paragraph 1. The states, Federal District, and municipalities subject to the special regime set forth in this Article shall, by means of an act of the Executive branch, opt for either: (CA 62, 2009)

I – depositing the amount referred to in paragraph 2 of this Article into a special account; or (CA 62, 2009)

II – adopting the special regime for a period of up to 15 (fifteen) years, in which case the percentage to be deposited into the special account referred to in paragraph 2 of this Article shall be equivalent to the total yearly balance of court-ordered debts, increased by the official rate applied to savings accounts and by simple interest applied at the same percentage of interest applied to savings accounts for the purpose of compensation for delay in the payment – the employment of compensatory interest being excluded, reduced by any paid amount, and divided by the remaining number of years in the special regime of payment. (CA 62, 2009)

Paragraph 2. In order to pay up both its past due and future accruing court-ordered debts through the special regime, the states, Federal District, and municipalities in debt shall make a monthly deposit into a special account created for such purpose, of 1/12 (one twelfth) of the amount calculated as a percentage of the respective net current revenues, as computed in the second month preceding the month of payment, whereas such percentage, calculated at the time of opting for the special regime and kept unchanged through the end of the period referred to in paragraph 14 of this Article, shall be equal to: (CA 62, 2009)

I – in the case of the states and of the Federal District: (CA 62, 2009)

a) at least 1.5% (one whole and five tenths percent), for the states of the North, Northeast, and Centre-West regions, in addition to the Federal District, or for those states where the backlog of court orders owed by their respective bodies and associated entities corresponds to up to 35% (thirty-five percent) of the total net current revenues; (CA 62, 2009)

b) at least 2% (two percent), for the states of the South and Southeast Regions, where the backlog of court orders owed by their respective bodies and associated entities corresponds to over 35% (thirty-five percent) of the net current revenues; (CA 62, 2009)

II – in the case of municipalities: (CA 62, 2009)

a) at least 1% (one percent), for municipalities of the North, Northeast, and Centre West regions, or for those municipalities where the backlog of court orders owed by their respective bodies and associated entities corresponds to up to 35% (thirty-five percent) of the net current revenues; (CA 62, 2009)

b) at least 1.5% (one whole and five tenths percent), for municipalities of the South and Southeast Regions, where the backlog of court orders owed by their respective bodies and associated entities corresponds to over 35% (thirty-five percent) of the net current revenues. (CA 62, 2009)

Paragraph 3. For the purposes of this Article, net current revenues mean the total sum of tax, industry, and agriculture revenues, property income, revenues from contributions and from services, current transfers, and other current revenues, including those deriving from paragraph 1 of Article 20 of the Federal Constitution, such total sum being computed in the period including the reference month and the 11 (eleven) preceding months, excluding any double counting but at the same time deducting: (CA 62, 2009)

I – in the case of the states, the portions remitted to the municipalities as set forth by the Constitution; (CA 62, 2009)

II – in the case of the states, Federal District, and municipalities, the contribution paid by respective employees to fund their own social security and social assistance system, as well as revenues deriving from the financial offsetting referred to in paragraph 9 of Article 201 of the Federal Constitution. (CA 62, 2009)

Paragraph 4. The special accounts referred to in paragraphs 1 and 2 shall be managed by the respective Court of Appeals, for payment of judicial orders issued by courts. (CA 62, 2009)

Paragraph 5. The funds deposited into the special accounts referred to in paragraphs 1 and 2 of this Article may not be returned to the states, Federal District, and municipalities in debt. (CA 62, 2009)

Paragraph 6. At least 50% (fifty percent) of the funds referred to in paragraphs 1 and 2 of this Article shall be used to pay court orders according to their chronological order of submission, with due regard for the priorities defined in paragraph 1 of Article 100 – in the case of court orders of one same year – and in paragraph 2 – in the case of court orders of all years. (CA 62, 2009)

Paragraph 7. If it is not possible to ascertain the chronological priority between 2 (two) court orders, the court order stating the smallest amount shall be paid first. (CA 62, 2009)

Paragraph 8. The employment of the remaining funds shall depend on decision by the states, Federal District, and municipalities in debt, through an act of the Executive branch, in accordance with the following modes, which may be applied either separately or simultaneously: (CA 62, 2009)

I – payment of court orders by means of auctions; (CA 62, 2009)

II – payment in cash of court orders not paid up under the terms of paragraph 6 and of item I, in a single, increasing order of respective amounts; (CA 62, 2009)

III – payment through direct agreement with creditors, under the terms of specific law to each federal entity in debt, which may provide for the establishment and mode of operation of conciliation panels. (CA 62, 2009)

Paragraph 9. The following shall apply to the auctions referred to in item I of paragraph 8 of this Article: (CA 62, 2009)

I – auctions shall be carried out through an electronic system managed by an entity authorized by the Brazilian Securities and Exchange Commission (CVM) or by the Central Bank of Brazil; (CA 62, 2009)

II – court orders – or an instalment of a court order amount as designated by its holder – with respect to which no appeal or challenge of any nature whatsoever is pending within the Judiciary shall be qualified to take part in an auction, whereas, at the initiative of the Executive branch, it will be permitted to offset court-order debt payments against clear legal debits, either registered or not under debts in execution and attributed to the original debtor by the Treasury in debt up to the date of issuance of respective court order, except for those whose enforceability has been stayed under the terms of law, or which have already been subject to deduction under the terms of paragraph 9 of Article 100 of the Federal Constitution; (CA 62, 2009)

III – auctions will be carried out through public offer to all creditors qualified by the respective federal entity in debt; (CA 62, 2009)

IV – any creditor who meets the requirements of item II shall be considered automatically qualified; (CA 62, 2009)

V – auctions shall be carried out as many times as necessary to meet the available amount; (CA 62, 2009)

VI – inclusion of an instalment of the total amount in an auction will be carried out the discretion of respective creditor, at an abatement in the amount of the instalment; (CA 62, 2009)

VII – auctions shall take the form of debt abatement, associated with the largest volume offered – either cumulated or not with the highest percentage of abatement,

according to the highest percentage of abatement, in which case the maximum amount per creditor may be stipulated, or according to another criterion to be defined in a public call notice; (CA 62, 2009)

VIII – the price formation mechanism shall be stated in the public call notices issued for each auction; (CA 62, 2009)

IX – the payment in part of a court order shall be ratified by the court which issued said court order. (CA 62, 2009)

Paragraph 10. Should the funds referred to in item II of paragraph 1 and in paragraphs 2 and 6 of this Article not be made available in due time: (CA 62, 2009)

I – there shall be decision of the amount in the accounts belonging to the states, Federal District, and municipalities in debt, by order of the presiding judge of the court referred to in paragraph 4, up to the limit of the amount not made available; (CA 62, 2009)

II – there shall be established, as an alternative, by order of the presiding judge of the relevant court, in favor of creditors of court orders, against the states, Federal District, and municipalities in debt, a clear legal right – self-enforceable and irrespective of regulation – to automatic offsetting against clear debits attributed to abovementioned creditors by such debtors, whereas, there being a balance in favor of a creditor, such amount shall automatically be deductible from the taxation owed to the states, Federal District, and municipalities in debt, up to the offsetting limits; (CA 62, 2009)

III – the head of the respective Executive branch shall be held liable under the terms of the legislation on fiscal responsibility and administrative improbity; (CA 62, 2009)

IV – for as long as non-compliance prevails, the federal entity in debt: (CA 62, 2009)

a) shall not be allowed to raise loans at home or abroad; (CA 62, 2009)

b) shall not be entitled to receive voluntary transfers; (CA 62, 2009)

V – the federal government shall retain the payments related to the Participation Fund of the States and Federal District and the Participation Fund of Municipalities, depositing them instead into the special accounts referred to in paragraph 1 of this Article, whereas the employment of such amounts must comply with paragraph 5 of this Article. (CA 62, 2009)

Paragraph 11. Regarding a court order concerning several creditors in a group of parties, the court of origin of the court order referred to may dismember the total amount per creditor, and each creditor may participate in an auction with the total amount such creditor is entitled to, the rule set forth in paragraph 3 of Article 100 of the Federal Constitution not being applicable to such case. (CA 62, 2009)

Paragraph 12. Should the legislation referred to in paragraph 4 of Article 100 not be enacted within 180 (one hundred and eighty) days from the date of enactment of Constitutional Amendment 62, the following amounts shall prevail for the relevant purposes, for the states, Federal District, and municipalities in debt which have failed to regulate the matter: (CA 62, 2009)

I – 40 (forty) monthly minimum wages in case of the states and Federal District; (CA 62, 2009)

II – 30 (thirty) monthly minimum wages in case of the municipalities. (CA 62, 2009)

Paragraph 13. During the period in which the states, Federal District, and municipalities in debt are paying court orders through the special regime, they may not be subject to seizure of funds, except when the funds referred to in item II of paragraph 1 and in paragraph 2 of this Article are not made available in due time. (CA 62, 2009)

Paragraph 14. The special regime for payment of court orders set forth in item I of paragraph 1 of this Article shall be in force for as long as the amount of court-ordered debts is higher than the amount of funds earmarked under the terms of paragraph 2 of this Article, or for a fixed period of 15 (fifteen) years in the case of the option referred to in item II of paragraph 1. (CA 62, 2009)

Paragraph 15. Court-ordered debts divided into instalments under the terms of Article 33 or Article 78 of this Temporary Constitutional Provisions Act and whose payment is still pending shall be included in the special regime with the amount of all pending instalments being updated, whereas the balance of any judicial and extrajudicial agreements shall also be included in the special regime. (CA 62, 2009)

Paragraph 16. From the date Constitutional Amendment 62 is enacted, the amounts stated in court orders, up until effective payment, irrespective of their nature, shall be adjusted according to the official rate applied to savings accounts, whereas, for the purpose of compensation of delay in the payment, simple interest will be applied at the same percentage of interest applied to savings accounts, the employment of compensatory interest being excluded. (CA 62, 2009)

Paragraph 17. While the special regime is in force, any amount in excess of the limit set forth in paragraph 2 of Article 100 of the Federal Constitution shall be paid in accordance with paragraphs 6 and 7 or with items I, II, and III of paragraph 8 of this Article, whereas the amounts used to meet the provision of paragraph 2 of Article 100 of the Federal Constitution shall be computed for the purposes of paragraph 6 of this Article. (CA 62, 2009)

Paragraph 18. While the special regime referred to in this Article is in force, the original holders of court orders who have reached the age of 60 (sixty) years old by the

date of enactment of Constitutional Amendment 62 shall also be entitled to the priority referred to in paragraph 6. (CA 62, 2009)

Article 98. The number of public defenders in each judicial district shall be proportional to the effective demand for the service of the Office of the Public Defender and to the respective population. (CA 80, 2014)

Paragraph 1. Within 8 (eight) years, the Union, states, and Federal District shall have public defenders in all of their judicial districts, with due regard for the head provision. (CA 80, 2014)

Paragraph 2. During the period set forth in paragraph 1 of this Article, the assignment of public defenders shall be carried out to serve, on a priority basis, those regions with higher levels of social exclusion and higher population density. (CA 80, 2014)

Article 99. For the purposes of the provisions in item VII of paragraph 2 of Article 155, in the case of transactions and provision of goods and services to end-users located in another state who are not taxpayers, the taxes corresponding to the difference between the internal rate and the inter-state rate will be shared between the state of origin and the state of destination, in the following proportions: (CA 87, 2015)

I – for the year 2015: 20% (twenty percent) for the state of destination and 80% (eighty percent) for the state of origin;

II – for the year 2016: 40% (forty percent) for the state of destination and 60% (sixty percent) for the State of origin;

III – for the year 2017: 60% (sixty percent) for the state of destination and 40% (forty percent) for the state of origin;

IV – for the year 2018: 80% (eighty percent) for the state of destination and 20% (twenty percent) for the state of origin;

V – as of year 2019: 100% (one hundred percent) for the state of destination.

Article 100. Until the supplementary law mentioned in item II of paragraph 1 of Article 40 of the Federal Constitution comes into force, the justices of the Federal Supreme Court, of the Higher Courts and of the Federal Accounting Court will retire, compulsorily, at the age of 75 (seventy five), under the conditions of Article 52 of the Federal Constitution. (CA 88, 2015)

Article 101. The states, Federal District, and municipalities that were in default on their court-ordered debts on March 25, 2015, shall pay off these overdue debts by December 31, 2024, as well as those debts that become due during this period, adjusted by the National Extended Consumer Price Index (IPCA-E), or by another index that replaces it, depositing monthly in a special account in the Court of Appeal of the State, under the court's sole and

exclusive administration, 1/12 (one-twelfth) of the amount calculated on a percentage basis over their respective net current receipts, determined in the second month prior to the month of payment, in a percentage sufficient for the satisfaction of their debts and, even though variable, never inferior than, in each fiscal year, to the percentage applied on the date the special regime referred to in this Article comes into force, in accordance with a payment plan to be submitted annually to the Court of Appeal of the State. (CA 99, 2017)

Paragraph 1. For the purposes of this Article, net current revenues means the sum of revenue collected from taxes, assets, industry, agriculture, and livestock; from contributions and services; from current transfers and other current receipts, including those stemming from paragraph 1 of Article 20 of the Federal Constitution, assessed in the period including the second month immediately prior to the referred month and the 11 (eleven) preceding months, excluding duplicities, and deductions: (CA 94, 2016)

I – from the states, the amounts transferred to the municipalities by constitutional precept; (CA 94, 2016)

II – from the states, the Federal District and the municipalities, the employees' contribution to fund their social security system and social assistance and the receipts from the financial compensation referred to in paragraph 9 of Article 201 of the Federal Constitution. (CA 94, 2016)

Paragraph 2. Court-ordered debts shall be paid with the governments' own budget stemming from the net current receipts referred to in paragraph 1 of this Article and, in addition, it may be paid by the following means: (CA 99, 2017)

I – up to 75% (seventy-five percent) of the amount of the judicial and administrative cash deposits from judicial or administrative proceedings, having them tax or non-tax nature, in which the state, the Federal District, or the municipalities are parties, or their government agencies, foundations, or dependent associated state-owned companies, through the institution of a guarantee fund in an amount equivalent to 1/3 (one third) of the resources collected, consisting of the remaining portion of judicial deposits and adjusted by the reference rate of the Special Settlement and Custody System (Selic) for federal securities, being it never lower than the rates and criteria applied to the deposits collected; (CA 99, 2017)

II – up to 30% (thirty percent) of other judicial deposits in the locality under the jurisdiction of the respective Court of Appeal, through the institution of a guarantee fund in an amount equivalent to the resources collected, consisting of the remaining portion of judicial deposits and adjusted by the reference rate of the Special Settlement

and Custody System (Selic) for federal securities, being it never lower than the rates and criteria applied to the deposits collected, for: (CA 99, 2017)

a) in the case of the Federal District, 100% (one hundred percent) of these resources shall be assigned to the Federal District itself; (CA 94, 2016)

b) in the case of the states, 50% (fifty percent) of the resources of the state itself and 50% (fifty percent) of its municipalities, according to the judicial district where the funds are deposited, and if there is more than one municipality in the same judicial district, the resources will be shared among the municipalities involved, proportionally to the respective populations, used as reference the last census survey or the most recent population estimate of the Brazilian Institute of Geography and Statistics Foundation (IBGE); (CA 99, 2017)

III – contracting of loans, exempted from such end the indebtedness limits dealt with in items VI and VII of the head provision of Article 52 of the Federal Constitution and any other indebtedness limits provided for by law, being inapplicable to these loans the prohibition to earmark tax revenues established in item IV of the head provision of Article 167 of the Federal Constitution; (CA 99, 2017)

IV – the total deposit made to settle court-ordered debts and direct requisitions of payment of small amount obligations up to December 31, 2009 and still not drawn out, being cancelled the respective bond and set aside the obligations. The bond shall be revalidated before the court of the proceeding at the request of the creditor and after hearing the debtor entity. The original chronological position and the indexes adjustments for the entire period shall be maintained. (CA 99, 2017)

Paragraph 3. The additional resources provided for in items I, II and IV of paragraph 2 of this Article shall be transferred directly by the depositary financial institution to the special account referred to in the head provision of this Article, under the sole and exclusive administration of the Court of Appeal of the State, and such transfer shall be carried out within sixty days after this paragraph comes into force, under penalty of personal liability of the head of the financial institution for improbity. (CA 99, 2017)

Paragraph 4. Within a period of up to six months after the special regime referred to in this Article comes into force, the federal government, directly or through the official financial institutions under its control, shall make available to the states, Federal District and municipalities, as well as to their associate agencies, foundations and companies, a credit line facility to settle court-ordered debts submitted to the special payment system referred to in this Article, subject to the following conditions. (CA 99, 2017)

I – as to financing the remaining court-ordered debts referred to in this paragraph, the criteria of rates and adjustments for inflation applied to court-ordered debts shall be applied, pursuant to paragraph 12 of Article 100 of the Federal Constitution; (CA 99, 2017)

II – the remaining court-ordered debts finance referred to in this paragraph shall be settled in monthly instalments sufficient to repay the constituted debt; (CA 99, 2017)

III – the amount of each installment referred to in item II of this paragraph shall be calculated on the net current revenue, respectively, of the state, Federal District and municipality, in the second month prior to payment, in a percentage equivalent to the 2012 average of monthly percentage commitment until the end of the period referred to in the head provision, being it considered for such purposes solely each federative entities' own resources to settle court-ordered debts; (CA 99, 2017)

IV – in the loans referred to in this paragraph, the indebtedness limits dealt with in items VI and VII of the head provision of Article 52 of the Federal Constitution and any other limits of indebtedness provided by law do not apply. (CA 99, 2017)

Article 102. So long as the special regime provided for in this Constitutional Amendment is in force, at least 50% (fifty percent) of the resources which, under Article 101 of this Temporary Constitutional Provisions Act, are assigned to settle court-ordered debts in default shall be used for payment in accordance with the chronological order of submission, respecting the priority of support-related credits and those with respect to age, state of health, and disabilities pursuant to paragraph 2 of Article 100 of the Federal Constitution, over all the other credits of all years. (CA 94, 2016)

Paragraph 1. The employment of the remaining resources, at discretion of the states, Federal District and municipalities, by act of their respective executives Branches, abiding by the priority order of creditors, may be assigned for payment through direct settlements, via the Auxiliaries Courts for Settlement of Judgement Debt of the government, reducing 40% (forty percent) of the updated credit value at most, so long as no appeal or judicial defense is pending with respect to the credit and that the requirements set forth in the regulations issued by the federal entity are observed. (CA 99, 2017)

Paragraph 2. Pursuant to the special regime foreseen in Article 101 of this Temporary Constitutional Provisions Act, priorities regarding age, health status and disability shall be met up to the amount equivalent to five times the one established by law for the purposes of the provision of paragraph 3 of Article 100 of the Federal Constitution. Payment of a fractional amount is permitted for such end and the remaining shall be paid in chronological order of submission of the court-ordered debts. (CA 99, 2017)

Article 103. So long as the states, Federal District and municipalities are making the payment of the monthly amounts owed as provided in the head provision of Article 101 of this Temporary Constitutional Provisions Act, neither they, nor their respective dependent associated agencies, foundations, or companies may suffer seizure of assets, except for failing to release resources on time. (CA 94, 2016)

Single paragraph. Under the special regime established in Article 101 of this Temporary Constitutional Provisions Act, expropriations are prohibited by the states, Federal District and municipalities, whose court-ordered debts still due, including the ones to be paid by their associated entities, are higher than 70% (seventy percent) of net current revenue, except for expropriations motivated by the public interest in the areas of health, education, public safety, public transportation, basic sanitation and housing of social interest. (CA 99, 2017)

Article 104. If the resources referred to under Article 101 of this Temporary Constitutional Provisions Act to settle court-ordered debts are not released on time, either in whole or in part: (CA 94, 2016)

I – the chief judge of the Court of Appeal of the State shall order the accounts seizure of the noncompliant-federal entity, up to the limit of the unreleased amounts; (CA 94, 2016)

II – the head of the respective executive branch of the non-compliant federal entity shall be held liable under the terms of the legislation on fiscal responsibility and administrative improbity; (CA 94, 2016)

III – the federal government shall retain the resources transferred to the Participation Fund of the states, the Federal District and the municipalities and shall deposit them in a special account referred to in Article 101 of this Temporary Constitutional Provisions Act, to be used as provided therein; (CA 94, 2016)

IV – the states shall retain the remittances provided for in the single paragraph of Article 158 of the Federal Constitution and deposit them in a special account referred to in Article 101 of this Temporary Constitutional Provisions Act, to be used as provided therein. (CA 94, 2016)

Single paragraph. So long as the omission persists, the federal entity may not negotiate foreign or domestic loans, except for the purposes provided for in paragraph 2 of Article 101 of this Temporary Constitutional Provisions Act and shall be prohibited from receiving voluntary transfers. (CA 94, 2016)

Article 105. So long as the regime to settle court-ordered debts provided for in Article 101 of this Temporary Constitutional Provisions Act remains in force, creditors of the judgement debt of the government, whether their own or of third parties, may offset debts

of tax or non-tax nature that by March 25, 2015, have been registered as an overdue tax liability of the states, Federal District or municipalities, abiding by the requirements established under the own law of the federal entity. (CA 94, 2016)

Paragraph 1. No sort of earmark, such as transferences to other entities and those assigned to education, health or other purposes, shall apply to the offset referred to in the head provision of this Article. (CA 99, 2017)

Paragraph 2. The states, Federal District and municipalities shall regulate in their respective laws the provisions of the head provision up to one hundred and twenty days from January 1st, 2018. (CA 99, 2017)

Paragraph 3. Having passed the time established in paragraph 2 of this Article without the regulation provided therein, creditors of court-ordered debts are authorized to exercise the choice referred to in the head provision of this Article. (CA 99, 2017)

Article 106. The New Fiscal Regime is hereby instituted to the fiscal budget and the social welfare budget of the Union and it shall be in force for twenty fiscal years, according to Articles 107 to 114 of this Temporary Constitutional Provisions Act. (CA 95, 2016)

Article 107. Individualized limits to primary public spending are hereby established for each fiscal year: (CA 95, 2016)

I – of the executive branch; (CA 95, 2016)

II – of the Federal Supreme Court, the Superior Court of Justice, the National Council of Justice, Labor Courts, Federal Courts, Federal Military Courts, Electoral Courts and the Court of the Federal District and Territories, within the judiciary; (CA 95, 2016)

III – of the Federal Senate, the Chamber of Deputies and the Federal Accounting Court, within the legislative branch; (CA 95, 2016)

IV – of the Prosecution Office of the Union and the National Council of the Prosecution Office; (CA 95, 2016)

V – of the Office of the Federal Public Defender. (CA 95, 2016)

Paragraph 1. Each of the limits referred to on the head provision shall be equivalent to: (CA 95, 2016)

I – fiscal year 2017, the primary spending paid in the fiscal year 2016, including expenses incurred but not yet paid, payments, and other operations that affect the primary operating result, corrected by 7.2% (seven and two tenths percent); and (CA 95, 2016)

II – for subsequent fiscal years, the value of the immediately preceding fiscal year limit, adjusted by the National Ample Consumer Price Index (IPCA) variation, published by the Brazilian Institute of Geography and Statistics, or another index that may replace

it, for the twelve-month period ending in June of the prior fiscal year to which the budget law refers to. (CA 95, 2016)

Paragraph 2. The limits under item IV of the heading of Article 51, item XIII of the heading of Article 52, paragraph 3 of Article 127 and paragraph 3 of Article 134 of the Federal Constitution shall not be higher than those established under this Article. (CA 95, 2016)

Paragraph 3. The message that forwards the budget bill shall demonstrate the programmed maximum values compatible with the individualized limits calculated according to the method under paragraph 1 of this Article and abide by paragraphs 7 and 9 of this Article. (CA 95, 2016)

Paragraph 4. The primary spending authorized in the annual budget law subject to the limits dealt with under this Article shall not exceed the maximum values demonstrated according to paragraph 3 of this Article. (CA 95, 2016)

Paragraph 5. It is forbidden to open supplementary or special credits that increase the authorized amount of primary spending subject to the limits dealt with in this Article. (CA 95, 2016)

Paragraph 6. The following shall not be included in the tax base and in the limits established under this Article: (CA 95, 2016)

I – constitutional transfers established under paragraph 1 of Article 20, item III of the single paragraph of Article 146, paragraph 5 of Article 153, Article 157, items I and II of Article 158, Article 159 and paragraph 6 of Article 212, the expenses referred to in item XIV of the heading of Article 21, all of the Federal Constitution, and the supplementary sources dealt with in items V and VII of the heading of Article 60 of this Temporary Constitutional Provisions Act; (CA 95, 2016)

II – extraordinary credits referred to in paragraph 3 of Article 167 of the Federal Constitution; (CA 95, 2016)

III – non-recurring expenses to hold elections of Electoral Courts; and (CA 95, 2016)

IV – expenses with capital increase of independent state-owned companies; (CA 95, 2016)

V – transfers to the Federal District, states, and municipalities of part of the amount collected through auctions of the exceeding limits referred to in paragraph 2 of Article 1 of Law 12,276, of June 30, 2010, and the expenses resulting from the revision of the onerous assignment contract provided for under the same law. (CA 102, 2019)

Paragraph 7. In the first three fiscal years the New Fiscal Regime is in force, the executive may even the amount exceeding the limits dealt with under items II to V of the head provision, according to the values established in the budget bill sent by the Executive in

the respective fiscal year, by reducing the equivalent amount of its primary spending. (CA 95, 2016)

Paragraph 8. The compensation dealt with in paragraph 7 of this Article shall not exceed 0.25% (twenty-five hundreds of one percent) of the executive branch 's limit. (CA 95, 2016)

Paragraph 9. With respect to the sum of each one of items II to IV of the head provision, the budget directives law may provide for the compensation among the individualized limits of the entities set forth in each item. (CA 95, 2016)

Paragraph 10. For the purposes of assessing the compliance with the limits dealt with under this Article, the primary spending carried out, including paid carryovers and other operations that affect the primary operating result in the fiscal year shall be considered. (CA 95, 2016)

Paragraph 11. Payment of carryovers incurred by December 31, 2015, may be excluded from the assessment of compliance with the limits dealt with in this Article, up to the amount of the primary result of the fiscal budget and the social welfare budget that exceeds the target set forth under the budget directives law. (CA 95, 2016)

Article 108. Starting in the tenth fiscal year of the New Fiscal Regime, the president of the Republic may propose a supplementary law bill to alter the correction method of the limits referred to in item II of paragraph 1 of Article 107 of this Temporary Constitutional Provisions Act. (CA 95, 2016)

Single paragraph. Only one correction method alteration of the limits shall be permitted per presidential office. (CA 95, 2016)

Article 109. In case of noncompliance with an individualized limit, the following prohibitions shall apply, until the end of the fiscal year the limit is complied with, to the executive or to the entities set forth under items II to V of the heading of Article 107 of this Temporary Constitutional Provisions Act that failed to comply with, without prejudice to other measures: (CA 95, 2016)

I – concession, on any account, of an advantage, increase, readjustment or adjustment of remuneration of members of the branch or associated entities, of civil servants or public employees and military, except those derived from a final and unappealable decision or a legal precept stemming from acts prior to this Constitutional Amendment entry in force; (CA 95, 2016)

II – creation of a position, job or function that implies an expense increase; (CA 95, 2016)

III – alteration of a career structure that implies spending increase; (CA 95, 2016)

IV – admission or employment of personnel, on any account, except for replacement of command or management positions that do not imply spending increase or those stemming from vacancies in permanent or life-tenured offices; (CA 95, 2016)

V – holding of competitive civil-service examinations, except for replacement of vacancies provided for in item IV; (CA 95, 2016)

VI – creation or increase in assistances, advantages, bonuses, allowances, reimbursement payments for union representation or benefits on any account in favor of members of the branches, the Prosecution Office or the Office of the Public Defender, civil servants, public employees and military; (CA 95, 2016)

VII – creation of obligatory spending items; (CA 95, 2016)

VIII – adoption of measures that imply a readjustment of obligatory spending above inflation rate, excluding the obligation to maintain the purchasing power referred to in item IV of the heading of Article 7 of the Federal Constitution. (CA 95, 2016)

Paragraph 1. Whenever any of the individualized limits of the entities set forth under items II, III, and IV of the heading of Article 107 of the Temporary Constitutional Provisions Act is not complied with, the prohibitions provided for in items I, III and VI of the heading apply to the entire group of entities referred to in each item. (CA 95, 2016)

Paragraph 2. In addition to what is provided in the head provision, in case of noncompliance with the limits established in item I of the heading of Article 107 of this Temporary Constitutional Provisions Act, the following acts are prohibited: (CA 95, 2016)

I – creation or expansion of credit programs and financing facilities, as well as the remission, renegotiation, or refinance of debts that imply increasing of expenses with subsidies; and (CA 95, 2016)

II – concession or increase in incentives or benefits of tax nature. (CA 95, 2016)

Paragraph 3. In case of noncompliance with any of the individualized limits set out in the heading of Article 107 of this Temporary Constitutional Provisions Act, granting the general review provided for in item X of the heading of Article 37 of the Federal Constitution is prohibited. (CA 95, 2016)

Paragraph 4. The prohibitions provided for in this Article also apply to legislative proposals. (CA 95, 2016)

Article 110. While the New Fiscal Regime is in force, the minimum spending in public health activities and services and in the maintenance and development of education shall be equal to: (CA 95, 2016)

I – in fiscal year 2017, the minimum spending calculated under item I of paragraph 2 of Article 198 and the heading of Article 212 of the Federal Constitution; and (CA 95, 2016)

II – in subsequent fiscal years, the values calculated for minimum spending in the immediately prior fiscal year, corrected according to the method established under item II of paragraph 1 of Article 107 of this Temporary Constitutional Provisions Act. (CA 95, 2016)

Article 111. From fiscal year 2018 until the last fiscal year the New Fiscal Regime is in force, the approval and execution provided for in paragraphs 9 and 11 of Article 166 of the Federal Constitution shall correspond to the amount for mandatory execution for fiscal year 2017, with the inflation adjusted in accordance to the method under item II of paragraph 1 of Article 107 of this Temporary Constitutional Provisions Act. (CA 95, 2016)

Article 112. The provisions introduced by the New Fiscal Regime: (CA 95, 2016)

I – do not constitute an obligation for future payment by the federal government or rights of others on the Treasury; and (CA 95, 2016)

II – do not repeal, disregard or suspend the compliance with constitutional and legal provisions that deal with fiscal goals or maximum limits on expenses. (CA 95, 2016)

Article 113. A legislative proposal that creates or alters mandatory expenses or waiver of revenues shall be accompanied by an estimate of its budget and financial effects. (CA 95, 2016)

Article 114. The proceeding of the legislative proposal under the heading of Article 59 of the Federal Constitution, with the exception of the one referred to in item V, whenever they imply spending increase or waiver of revenues, shall be suspended for up to twenty days, at the request of one fifth of the members of the Nacional Congress respective organ, according internal regulations, for analysis of its compatibility with the New Fiscal Regime. (CA 95, 2016)

Brasília, October 5, 1988.

Ulysses Guimarães, President – Mauro Benevides, First VicePresident – Jorge Arbage, Second VicePresident – Marcelo Cordeiro, First Secretary – Mário Maia, Second Secretary – Arnaldo Faria de Sá, Third Secretary – Benedita da Silva, First Substitute Secretary – Luiz Soyer, Second Substitute Secretary – Sotero Cunha, Third Substitute Secretary – Bernardo Cabral, Reporter-General – Adolfo Oliveira, Adjunct Reporter – Antonio Carlos Konder Reis, Adjunct Reporter – José Fogaça, Adjunct Reporter – Abigail Feitosa – Acival Gomes – Adauto Pereira – Ademir Andrade – Adhemar de Barros Filho – Adroaldo Streck – Adylson Motta – Aécio de Borba – Aécio Neves – Affonso Camargo – Afif Domingos – Afonso Arinos – Afonso Sancho – Agassiz Almeida – Agripino de Oliveira Lima – Airton Cordeiro – Airton Sandoval – Alarico Abib – Albano Franco – Albérico Cordeiro – Albérico

Filho – Alceni Guerra – Alcides Saldanha – Aldo Arantes – Alércio Dias – Alexandre Costa – Alexandre Puzyna – Alfredo Campos – Almir Gabriel – Aloisio Vasconcelos – Aloysio Chaves – Aloysio Teixeira – Aluizio Bezerra – Aluízio Campos – Álvaro Antônio – Álvaro Pacheco – Álvaro Valle – Alysson Paulinelli – Amaral Netto – Amaury Müller – Amilcar Moreira – Ângelo Magalhães – Anna Maria Rattes – Annibal Barcellos – Antero de Barros – Antônio Câmara – Antônio Carlos Franco – Antonio Carlos Mendes Thame – Antônio de Jesus – Antonio Ferreira – Antonio Gaspar – Antonio Mariz – Antonio Perosa – Antônio Salim Curiati – Antonio Ueno – Arnaldo Martins – Arnaldo Moraes – Arnaldo Prieto – Arnold Fioravante – Arolde de Oliveira – Artenir Werner – Artur da Távola – Asdrubal Bentes – Assis Canuto – Átila Lira – Augusto Carvalho – Áureo Mello – Basílio Villani – Benedicto Monteiro – Benito Gama – Beth Azize – Bezerra de Melo – Bocayuva Cunha – Bonifácio de Andrada – Bosco França – Brandão Monteiro – Caio Pompeu – Carlos Alberto – Carlos Alberto Caó – Carlos Benevides – Carlos Cardinal – Carlos Chiarelli – Carlos Cotta – Carlos De'Carli – Carlos Mosconi – Carlos Sant'Anna – Carlos Vinagre – Carlos Virgílio – Carrel Benevides – Cássio Cunha Lima – Célio de Castro – Celso Dourado – César Cals Neto – César Maia – Chagas Duarte – Chagas Neto – Chagas Rodrigues – Chico Humberto – Christóvam Chiaradia – Cid Carvalho – Cid Sabóia de Carvalho – Cláudio Ávila – Cleonâncio Fonseca – Costa Ferreira – Cristina Tavares – Cunha Bueno – Dálton Canabrava – Darcy Deitos – Darcy Pozza – Daso Coimbra – Davi Alves Silva – Del Bosco Amaral – Delfim Netto – Délio Braz – Denisar Arneiro – Dionisio Dal Prá – Dionísio Hage – Dirce Tutu Quadros – Dirceu Carneiro – Divaldo Suruagy – Djenal Gonçalves – Domingos Juvenil – Domingos Leonelli – Doreto Campanari – Edésio Frias – Edison Lobão – Edivaldo Motta – Edme Tavares – Edmilson Valentim – Eduardo Bonfim – Eduardo Jorge – Eduardo Moreira – Egídio Ferreira Lima – Elias Murad – Eliel Rodrigues – Eliézer Moreira – Enoc Vieira – Eraldo Tinoco – Eraldo Trindade – Erico Pegoraro – Ervin Bonkoski – Etevaldo Nogueira – Euclides Scalco – Eunice Michiles – Evaldo Gonçalves – Expedito Machado – Ézio Ferreira – Fábio Feldmann – Fábio Raunheitti – Farabulini Júnior – Fausto Fernandes – Fausto Rocha – Felipe Mendes – Feres Nader – Fernando Bezerra Coelho – Fernando Cunha – Fernando Gasparian – Fernando Gomes – Fernando Henrique Cardoso – Fernando Lyra – Fernando Santana – Fernando Velasco – Firmo de Castro – Flavio Palmier da Veiga – Flávio Rocha – Florestan Fernandes – Floriceno Paixão – França Teixeira – Francisco Amaral – Francisco Benjamim – Francisco Carneiro – Francisco Coelho – Francisco Diógenes – Francisco Dornelles – Francisco Küster – Francisco Pinto – Francisco Rollemberg – Francisco Rossi – Francisco Sales – Furtado Leite – Gabriel Guerreiro – Gandi Jamil – Gastone Righi – Genebaldo

Correia – Genésio Bernardino – Geovani Borges – Geraldo Alckmin Filho – Geraldo Bulhões – Geraldo Campos – Geraldo Fleming – Geraldo Melo – Gerson Camata – Gerson Marcondes – Gerson Peres – Gidel Dantas – Gil César – Gilson Machado – Gonzaga Patriota – Guilherme Palmeira – Gumercindo Milhomem – Gustavo de Faria – Harlan Gadelha – Haroldo Lima – Haroldo Sabóia – Hélio Costa – Hélio Duque – Hélio Manhães – Hélio Rosas – Henrique Córdova – Henrique Eduardo Alves – Heráclito Fortes – Hermes Zaneti – Hilário Braun – Homero Santos – Humberto Lucena – Humberto Souto – Iberê Ferreira – Ibsen Pinheiro – Inocêncio Oliveira – Irajá Rodrigues – Iram Saraiva – Irapuan Costa Júnior – Irma Passoni – Ismael Wanderley – Israel Pinheiro – Itamar Franco – Ivo Cersósimo – Ivo Lech – Ivo Mainardi – Ivo Vanderlinde – Jacy Scanagatta – Jairo Azi – Jairo Carneiro – Jalles Fontoura – Jamil Haddad – Jarbas Passarinho – Jayme Paliarin – Jayme Santana – Jesualdo Cavalcanti – Jesus Tajra – Joaci Góes – João Agripino – João Alves – João Calmon – João Carlos Bacelar – João Castelo – João Cunha – João da Mata – João de Deus Antunes – João Herrmann Neto – João Lobo – João Machado Rollemberg – João Menezes – João Natal – João Paulo – João Rezek – Joaquim Bevilácqua – Joaquim Francisco – Joaquim Hayckel – Joaquim Sucena – Jofran Frejat – Jonas Pinheiro – Jonival Lucas – Jorge Bornhausen – Jorge Hage – Jorge Leite – Jorge Uequed – Jorge Vianna – José Agripino – José Camargo – José Carlos Coutinho – José Carlos Grecco – José Carlos Martinez – José Carlos Sabóia – José Carlos Vasconcelos – José Costa – José da Conceição – José Dutra – José Egreja – José Elias – José Fernandes – José Freire – José Genoíno – José Geraldo – José Guedes – José Ignácio Ferreira – José Jorge – José Lins – José Lourenço – José Luiz de Sá – José Luiz Maia – José Maranhão – José Maria Eymael – José Maurício – José Melo – José Mendonça Bezerra – José Moura – José Paulo Bisol – José Queiroz – José Richa – José Santana de Vasconcellos – José Serra – José Tavares – José Teixeira – José Thomaz Nonô – José Tinoco – José Ulísses de Oliveira – José Viana – José Yunes – Jovanni Masini – Juarez Antunes – Júlio Campos – Júlio Costamilan – Jutahy Júnior – Jutahy Magalhães – Koyu Iha – Lael Varella – Lavoisier Maia – Leite Chaves – Lélio Souza – Leopoldo Peres – Leur Lomanto – Levy Dias – Lézio Sathler – Lídice da Mata – Louremberg Nunes Rocha – Lourival Baptista – Lúcia Braga – Lúcia Vânia – Lúcio Alcântara – Luís Eduardo – Luís Roberto Ponte – Luiz Alberto Rodrigues – Luiz Freire – Luiz Gushiken – Luiz Henrique – Luiz Inácio Lula da Silva – Luiz Leal – Luiz Marques – Luiz Salomão – Luiz Viana – Luiz Viana Neto – Lysâneas Maciel – Maguito Vilela – Maluly Neto – Manoel Castro – Manoel Moreira – Manoel Ribeiro – Mansueto de Lavor – Manuel Viana – Márcia Kubitschek – Márcio Braga – Márcio Lacerda – Marco Maciel – Marcondes Gadelha – Marcos Lima – Marcos Queiroz – Maria de Lourdes Abadia – Maria Lúcia – Mário Assad

– Mário Covas – Mário de Oliveira – Mário Lima – Marluce Pinto – Matheus Iensen – Mattos Leão – Maurício Campos – Maurício Correa – Maurício Fruet – Maurício Nasser – Maurício Pádua – Maurílio Ferreira Lima – Mauro Borges – Mauro Campos – Mauro Miranda – Mauro Sampaio – Max Rosenmann – Meira Filho – Melo Freire – Mello Reis – Mendes Botelho – Mendes Canale – Mendes Ribeiro – Messias Góis – Messias Soares – Michel Temer – Milton Barbosa – Milton Lima – Milton Reis – Miraldo Gomes – Miro Teixeira – Moema São Thiago – Moysés Pimentel – Mozarildo Cavalcanti – Mussa Demes – Myrian Portella – Nabor Júnior – Naphtali Alves de Souza – Narciso Mendes – Nelson Aguiar – Nelson Carneiro – Nelson Jobim – Nelson Sabrá – Nelson Seixas – Nelson Wedekin – Nelton Friedrich – Nestor Duarte – Ney Maranhão – Nilso Sguarezi – Nilson Gibson – Nion Albernaz – Noel de Carvalho – Nyder Barbosa – Octávio Elísio – Odacir Soares – Olavo Pires – Olívio Dutra – Onofre Corrêa – Orlando Bezerra – Orlando Pacheco – Oscar Corrêa – Osmar Leitão – Osmir Lima – Osmundo Rebouças – Osvaldo Bender – Osvaldo Coelho – Osvaldo Macedo – Osvaldo Sobrinho – Oswaldo Almeida – Oswaldo Trevisan – Ottomar Pinto – Paes de Andrade – Paes Landim – Paulo Delgado – Paulo Macarini – Paulo Marques – Paulo Mincarone – Paulo Paim – Paulo Pimentel – Paulo Ramos – Paulo Roberto – Paulo Roberto Cunha – Paulo Silva – Paulo Zarzur – Pedro Canedo – Pedro Ceolin – Percival Muniz – Pimenta da Veiga – Plínio Arruda Sampaio – Plínio Martins – Pompeu de Sousa – Rachid Saldanha Derzi – Raimundo Bezerra – Raimundo Lira – Raimundo Rezende – Raquel Cândido – Raquel Capiberibe – Raul Belém – Raul Ferraz – Renan Calheiros – Renato Bernardi – Renato Johnsson – Renato Vianna – Ricardo Fiuza – Ricardo Izar – Rita Camata – Rita Furtado – Roberto Augusto – Roberto Balestra – Roberto Brant – Roberto Campos – Roberto D'Ávila – Roberto Freire – Roberto Jefferson – Roberto Rollemberg – Roberto Torres – Roberto Vital – Robson Marinho – Rodrigues Palma – Ronaldo Aragão – Ronaldo Carvalho – Ronaldo Cezar Coelho – Ronan Tito – Ronaro Corrêa – Rosa Prata – Rose de Freitas – Rospide Netto – Rubem Branquinho – Rubem Medina – Ruben Figueiró – Ruberval Pilotto – Ruy Bacelar – Ruy Nedel – Sadie Hauache – Salatiel Carvalho – Samir Achôa – Sandra Cavalcanti – Santinho Furtado – Sarney Filho – Saulo Queiroz – Sérgio Brito – Sérgio Spada – Sérgio Werneck – Severo Gomes – Sigmaringa Seixas – Sílvio Abreu – Simão Sessim – Siqueira Campos – Sólon Borges dos Reis – Stélio Dias – Tadeu França – Telmo Kirst – Teotonio Vilela Filho – Theodoro Mendes – Tito Costa – Ubiratan Aguiar – Ubiratan Spinelli – Uldurico Pinto – Valmir Campelo – Valter Pereira – Vasco Alves – Vicente Bogo – Victor Faccioni – Victor Fontana – Victor Trovão – Vieira da Silva – Vilson Souza – Vingt Rosado – Vinicius Cansanção – Virgildásio de Senna – Virgílio Galassi – Virgílio Guimarães – Vitor Buaiz

– Vivaldo Barbosa – Vladimir Palmeira – Wagner Lago – Waldec Ornélas – Waldyr Pugliesi – Walmor de Luca – Wilma Maia – Wilson Campos – Wilson Martins – Ziza Valadares.

Participants: Álvaro Dias – Antônio Britto – Bete Mendes – Borges da Silveira – Cardoso Alves – Edivaldo Holanda – Expedito Júnior – Fadah Gattass – Francisco Dias – Geovah Amarante – Hélio Gueiros – Horácio Ferraz – Hugo Napoleão – Iturival Nascimento – Ivan Bonato – Jorge Medauar – José Mendonça de Morais – Leopoldo Bessone – Marcelo Miranda – Mauro Fecury – Neuto de Conto – Nivaldo Machado – Oswaldo Lima Filho – Paulo Almada – Prisco Viana – Ralph Biasi – Rosário Congro Neto – Sérgio Naya – Tidei de Lima.

In Memoriam: Alair Ferreira – Antônio Farias – Fábio Lucena – Norberto Schwantes – Virgílio Távora.

Published in the Official Journal, October 5, 1988.

REVISION CONSTITUTIONAL AMENDMENTS

REVISION CONSTITUTIONAL AMENDMENT 1, 1994
(Published in the Official Journal, March 3, 1994)
Amends the Temporary Constitutional Provisions Act to include rules about the Emergency Social Fund and establishes other provisions.

REVISION CONSTITUTIONAL AMENDMENT 2, 1994
(Published in the Official Journal, June 9, 1994)
Amends precepts of the Federal Constitution, adding expressions to its Article 50.

REVISION CONSTITUTIONAL AMENDMENT 3, 1994
(Published in the Official Journal, June 9, 1994)
Amends subitem c of item I, subitem b *of item II, paragraph 1 and item II of paragraph 4 of Article 12 of the Federal Constitution.*

REVISION CONSTITUTIONAL AMENDMENT 4, 1994
(Published in the Official Journal, June 9, 1994)
Adds expressions to paragraph 9 of Article 14 of the Constitution.

REVISION CONSTITUTIONAL AMENDMENT 5, 1994
(Published in the Official Journal, June 9, 1994)
Amends Article 82 of the Federal Constitution. [...]
Article 2. This Constitutional Amendment shall come into force on January 1, 1995.

REVISION CONSTITUTIONAL AMENDMENT 6, 1994
(Published in the Official Journal, June 9, 1994)
Adds paragraph 4 to Article 55 of the Federal Constitution.

CONSTITUTIONAL AMENDMENTS

CONSTITUTIONAL AMENDMENT 1, 1992
(Published in the Official Journal, April 6, 1992)
Provides for the remuneration of stte deputies and municipal councilors.

CONSTITUTIONAL AMENDMENT 2, 1992
(Published in the Official Journal, September 1, 1992)
Provides for the plebiscite set forth in Article 2 of the Temporary Constitutional Provisions Act.

CONSTITUTIONAL AMENDMENT 3, 1993
(Published in the Official Journal, March 18, 1993)
Alters Articles 40, 42, 102, 103, 155, 156, 160, 167 of the Federal Constitution.

CONSTITUTIONAL AMENDMENT 4, 1993
(Published in the Official Journal, September 15, 1993)
Gives new wording to Article 16 of the Federal Constitution.

CONSTITUTIONAL AMENDMENT 5, 1995
(Published in the Official Journal, August 16, 1995)
Alters paragraph 2 of Article 25 of the Federal Constitution.

CONSTITUTIONAL AMENDMENT 6, 1995
(Published in the Official Journal, August 16, 1995)
Alters item IX of Article 170, Article 171, and paragraph 1 of Article 176 of the Federal Constitution.

CONSTITUTIONAL AMENDMENT 7, 1995
(Published in the Official Journal, August 16, 1995)
Alters Article 178 of the Federal Constitution and provides for the adoption of provisional presidential decrees.

CONSTITUTIONAL AMENDMENT 8, 1995
(Published in the Official Journal, August 16, 1995)
Alters item XI and subitem a *of item XII of Article 21 of the Federal Constitution.*

CONSTITUTIONAL AMENDMENT 9, 1995
(Published in the Official Journal, November 10, 1995)
Gives new wording to Article 177 of the Federal Constitution, altering and inserting paragraphs.

CONSTITUTIONAL AMENDMENT 10, 1996
(Published in the Official Journal, March 7, 1996)
Alters Articles 71 and 72 of the Temporary Constitutional Provisions Act, introduced by the Revision Constitutional Amendment 1 of 1994.

CONSTITUTIONAL AMENDMENT 11, 1996
(Published in the Official Journal, May 2, 1996)
Allows hiring foreign professors, technicians and scientists by Brazilian universities and grants autonomy to scientific and technological research institutions.

CONSTITUTIONAL AMENDMENT 12, 1996
(Published in the Official Journal, August 16, 1996)
Grants power to the Union to levy: provisional contribution on the movement or transmission of resources and of credits and financial rights.

CONSTITUTIONAL AMENDMENT 13, 1996
(Published in the Official Journal, August 22, 1996)
Gives new wording to item II of Article 192 of the Federal Constitution.

CONSTITUTIONAL AMENDMENT 14, 1996
(Published in the Official Journal, September 13, 1996)
Alters Articles 34, 208, 211 and 212 of the Federal Constitution and gives new wording to Article 60 of the Temporary Constitutional Provisions Act.

CONSTITUTIONAL AMENDMENT 15, 1996
(Published in the Official Journal, September 13, 1996)
Gives new wording to paragraph 4 of Article 18 of the Federal Constitution.

CONSTITUTIONAL AMENDMENT 16, 1997
(Published in the Official Journal, June 5, 1997)
Gives new wording to paragraph 5 of Article 14, to the heading of Article 28, to item II of Article 29, to the heading of Article 77, and to Article 82 of the Federal Constitution.

CONSTITUTIONAL AMENDMENT 17, 1997
(Published in the Official Journal, November 25, 1997)
Alters provisions of Articles 71 and 72 of the Temporary Constitutional Provisions Act, introduced by the Revision Constitutional Amendment 1 of 1994.

CONSTITUTIONAL AMENDMENT 18, 1998
(Published in the Official Journal, February 6, 1998, rectified on February 16, 1998)
Establishes constitutional rules for militaries.

CONSTITUTIONAL AMENDMENT 19, 1998
(Published in the Official Journal, June 5, 1998)
Alters the regime of and provides for the principles and rules of the public administration, servants, and political agents, control of expenditures and public finance, and costing of activities within the responsibility of the Federal District and established other provisions.

CONSTITUTIONAL AMENDMENT 20, 1998
(Published in the Official Journal, December 16, 1998)
Alters the social security system, establishes transitional precepts and other provisions.

CONSTITUTIONAL AMENDMENT 21, 1999
(Published in the Official Journal, March 19, 1999)
Extends the provisional contribution on the movement or transmission of revenues and of credits and financial rights, referred to in Article 74 of the Temporary Constitutional Provisions Act, and alters its rate.

CONSTITUTIONAL AMENDMENT 22, 1999
(Published in the Official Journal, March 19, 1999)
Adds a single paragraph to Article 98 and alters subitem i *of item I of Article 102, and subitem c of item I of Article 105 of the Federal Constitution.*

CONSTITUTIONAL AMENDMENT 23, 1999
(Published in the Official Journal, September 3, 1999)
Alters Articles 12, 52, 84, 91, 102, and 105 of the Federal Constitution (establishment of the Ministry of Defense).

CONSTITUTIONAL AMENDMENT 24, 1999
(Published in the Official Journal, December 10, 1999)
Alters provisions of the Federal Constitution regarding the composition of class judges representing employees and employers in Labor Courts.

CONSTITUTIONAL AMENDMENT 25, 2000
(Published in the Official Journal, February 15, 2000)
Alters item VI of Article 29 and adds Article 29-A to the Federal Constitution, regarding limits on expenditures with the municipal legislative branch.

CONSTITUTIONAL AMENDMENT 26, 2000
(Published in the Official Journal, February 15, 2000)
Alters the wording of Article 6 of the Federal Constitution.

CONSTITUTIONAL AMENDMENT 27, 2000
(Published in the Official Journal, March 22, 2000)
Adds Article 76 to the Temporary Constitutional Provisions Act, providing that a certain amount of the proceeds from the collection of federal taxes and social contributions shall be earmarked.

CONSTITUTIONAL AMENDMENT 28, 2000

(Published in the Official Journal, May 26, 2000, rectified on May 29, 2000)

Gives new wording to item XXIX of Article 7 and repeals Article 233 of the Federal Constitution.

CONSTITUTIONAL AMENDMENT 29, 2000

(Published in the Official Journal, September 14, 2000)

Alters Articles 34, 35, 156, 160, 167, and 198 of the Federal Constitution, and adds an Article to the Temporary Constitutional Provisions Act, to guarantee a minimum amount of funds to finance health activities and public services.

CONSTITUTIONAL AMENDMENT 30, 2000

(Published in the Official Journal, September 14, 2000)

Alters the wording of Article 100 of the Federal Constitution and adds Article 78 to the Temporary Constitutional Provisions Act, regarding payment of court order debts.

CONSTITUTIONAL AMENDMENT 31, 2000

(Published in the Official Journal, December 18, 2000)

Alters the Temporary Constitutional Provisions Act, introducing Articles that establish the Fund to Fight and Eradicate Poverty.

CONSTITUTIONAL AMENDMENT 32, 2001

(Published in the Official Journal, September 12, 2001)

Alters provisions of Articles 48, 57, 61, 62, 64, 66, 84, 88, and 246 of the Federal Constitution, and establishes other provisions.

CONSTITUTIONAL AMENDMENT 33, 2001

(Published in the Official Journal, December 12, 2001)

Alters Articles 149, 155, and 177 of the Federal Constitution.

CONSTITUTIONAL AMENDMENT 34, 2001

(Published in the Official Journal, December 14, 2001)

Gives new wording to subitem c *of item XVI of Article 37 of the Federal Constitution.*

CONSTITUTIONAL AMENDMENT 35, 2001
(Published in the Official Journal, December 21, 2001)
Gives new wording to Article 53 of the Federal Constitution.

CONSTITUTIONAL AMENDMENT 36, 2002
(Published in the Official Journal, May 29, 2002)
Gives new wording to Article 222 of the Federal Constitution to allow the participation of legal entities in the capital stock of newspaper companies, sound broadcasting companies, and sound and image broadcasting companies, under the conditions herein stipulated.

CONSTITUTIONAL AMENDMENT 37, 2002
(Published in the Official Journal, June 13, 2002)
Alters Articles 100 and 156 of the Federal Constitution and adds Articles 84, 85, 86, 87, and 88 to the Temporary Constitutional Provisions Act.

CONSTITUTIONAL AMENDMENT 38, 2002
(Published in the Official Journal, June 13, 2002)
Adds Article 89 to the Temporary Constitutional Provisions Act, to include the Military Police of the former Federal Territory of Rondônia into the Union staff.

CONSTITUTIONAL AMENDMENT 39, 2002
(Published in the Official Journal, December 20, 2002)
Adds Article 149-A to the Federal Constitution (creates a contribution to finance public lighting services in municipalities and the Federal District).

CONSTITUTIONAL AMENDMENT 40, 2003
(Published in the Official Journal, May 30, 2003)
Alters item V of Article 163 and Article 192 of the Federal Constitution, and the heading of Article 52 of the Temporary Constitutional Provisions Act.

CONSTITUTIONAL AMENDMENT 41, 2003
(Published in the Official Journal, December 31, 2003)
Alters Articles 37, 40, 42, 48, 96, 149, and 201 of the Federal Constitution, repeals item IX of paragraph 3 of Article 142 of the Federal Constitution and provisions of Constitutional Amendment 20, of December 15, 1998, and establishes other provisions.

CONSTITUTIONAL AMENDMENT 42, 2003
(Published in the Official Journal, December 31, 2003)
Alters the National Tax System and establishes other provisions.

CONSTITUTIONAL AMENDMENT 43, 2004
(Published in the Official Journal, April 16, 2004)
Alters Article 42 of the Temporary Constitutional Provisions Act, extending, for 10 (ten) years, application by the Union of minimum percentages of the total amount of funds intended for irrigation in the Centre West and Northeast Regions.

CONSTITUTIONAL AMENDMENT 44, 2004
(Published in the Official Journal, July 1, 2004)
Alters the National Tax System and makes further provisions.

CONSTITUTIONAL AMENDMENT 45, 2004
(Published in the Official Journal, December 31, 2004)
Alters provisions of Articles 5, 36, 52, 92, 93, 95, 98, 99, 102, 103, 104, 105, 107, 109, 111, 112, 114, 115, 125, 126, 127, 128, 129, 134, and 168 of the Federal Constitution, and adds Articles 103 A, 103 B, 111 A, and 130 A, and establishes other provisions.

CONSTITUTIONAL AMENDMENT 46, 2005
(Published in the Official Journal, May 6, 2005)
Alters item IV of Article 20 of the Federal Constitution.

CONSTITUTIONAL AMENDMENT 47, 2005
(Published in the Official Journal, July 6, 2005)
Alters Articles 37, 40, 195, and 201 of the Federal Constitution, to set forth on social security matters, and establishes other provisions.

CONSTITUTIONAL AMENDMENT 48, 2005
(Published in the Official Journal, August 11, 2005)
Adds paragraph 3 to Article 215 of the Federal Constitution to institute the National Culture Plan.

CONSTITUTIONAL AMENDMENT 49, 2006
(Published in the Official Journal, February 9, 2006)
Alters the wording of subitem b *and adds subitem* c *to item XXIII of the heading of Article 21, and alters the wording of item V of the heading of Article 177 of the Federal Constitution to exclude the production, sale, and use of short lived radioisotopes for medical, agricultural, and industrial purposes from the monopoly of the Union.*

CONSTITUTIONAL AMENDMENT 50, 2006
(Published in the Official Journal, February 15, 2006)
Alters Article 57 of the Federal Constitution.

CONSTITUTIONAL AMENDMENT 51, 2006
(Published in the Official Journal, February 15, 2006)
Adds paragraphs 4, 5, and 6 to Article 198 of the Federal Constitution.

CONSTITUTIONAL AMENDMENT 52, 2006
(Published in the Official Journal, March 9, 2006)
Gives new wording to paragraph 1 of Article 17 of the Federal Constitution to regulate electoral coalitions.

CONSTITUTIONAL AMENDMENT 53, 2006
(Published in the Official Journal, December 20, 2006)
Gives new wording to Articles 7, 23, 30, 206, 208, 211, and 212 of the Federal Constitution and to Article 60 of the Temporary Constitutional Provisions Act.

CONSTITUTIONAL AMENDMENT 54, 2007
(Published in the Official Journal, September 21, 2007)
Gives new wording to subitem c *of item I of Article 12 of the Federal Constitution and adds Article 95 to the Temporary Constitutional Provisions Act, to ensure that Brazilians born abroad may be registered with Brazilian consulates.*

CONSTITUTIONAL AMENDMENT 55, 2007
(Published in the Official Journal, September 21, 2007)
Alters Article 159 of the Federal Constitution, to increase the amount of funds remitted by the federal government to the Revenue Sharing Fund of the Municipalities.

CONSTITUTIONAL AMENDMENT 56, 2007
(Published in the Official Journal, December 21, 2007)
Extends the period stipulated in the heading of Article 76 of the Temporary Constitutional Provisions Act and establish other provisions.

CONSTITUTIONAL AMENDMENT 57, 2008
(Published in the Official Journal, December 18, 2008 – Extra Edition)
Adds an Article to the Temporary Constitutional Provisions Act to validate the acts of creation, fusion, merger, and dismemberment of municipalities.

CONSTITUTIONAL AMENDMENT 58, 2009
(Published in the Official Journal, September 24, 2009)
Alters the wording of item IV of the heading of Article 29 and the wording of Article 29-A of the Federal Constitution, establishing provisions for the composition of municipal chambers.

CONSTITUTIONAL AMENDMENT 59, 2009
(Published in the Official Journal, November 12, 2009)
Adds paragraph 3 to Article 76 of the Temporary Constitutional Provisions Act, to reduce, annually, starting in fiscal year 2009, the percentage of Detachment of the Federal Revenue levied on resources earmarked to the maintenance and development of education referred to in Article 212 of the Federal Constitution; gives new wording to items I and VII of Article 208, to provide for mandatory education from four to seventeen years and expend the scope of supplementary programs for all stages of basic education; and gives new wording to paragraph 4 of Article 211, to paragraph 3 of Article 212, and to the heading of Article 214, also adding item VI to Article 214.

CONSTITUTIONAL AMENDMENT 60, 2009
(Published in the Official Journal, November 12, 2009)
Alters Article 89 of the Temporary Constitutional Provisions Act to set forth the staff of civil and military servants of the former Federal Territory of Rondônia.

CONSTITUTIONAL AMENDMENT 61, 2009
(Published in the Official Journal, November 12, 2009)
Alters Article 103-B of the Federal Constitution, to change the composition of the National Council of Justice.

CONSTITUTIONAL AMENDMENT 62, 2009
(Published in the Official Journal, December 10, 2009)
Alters Article 100 of the Federal Constitution and adds Article 97 to the Temporary Constitutional Provisions Act, to establish a special regime to pay up court ordered debts for the states, Federal District, and municipalities.

CONSTITUTIONAL AMENDMENT 63, 2010
(Published in the Official Journal, February 5, 2010)
Alters paragraph 5 of Article 198 of the Federal Constitution to provide for a nationwide minimum salary and guidelines for the Career Schemes of community health workers and endemic disease control agents.

CONSTITUTIONAL AMENDMENT 64, 2010
(Published in the Official Journal, February 5, 2010)
Changes the wording of Article 6 of the Federal Constitution to include food as a social right.

CONSTITUTIONAL AMENDMENT 65, 2010
(Published in the Official Journal, July 14, 2010)
Changes the name of Chapter VII of Title VIII of the Federal Constitution and alters Article 227 to protect the youth interests.

CONSTITUTIONAL AMENDMENT 66, 2010
(Published in the Official Journal, July 14, 2010)
Gives new wording to paragraph 6 of Article 226 of the Federal Constitution, which provides for the dissolution of civil marriage by divorce, thus ending the prior requirement of legal separation for more than 1 (one) year or of proven de facto *separation for more than 2 (two) years.*

CONSTITUTIONAL AMENDMENT 67, 2010
(Published in the Official Journal, December 23, 2010)
Extends, indefinitely, the period of enforcement for the Fund to Fight and Eradicate Poverty.

CONSTITUTIONAL AMENDMENT 68, 2011
(Published in the Official Journal, December 22, 2011)
Alters Article 76 of the Temporary Constitutional Provisions Act.

CONSTITUTIONAL AMENDMENT 69, 2012
(Published in the Official Journal, March 30, 2012)
Alters Articles 21, 22, and 48 of the Federal Constitution, with a view to transferring from the Federal Government to the Federal District the duties to organize and maintain the Office of the Public Defender of the Federal District.

CONSTITUTIONAL AMENDMENT 70, 2012
(Published in the Official Journal, March 30, 2012)
Alters Constitutional Amendment 41, of 2003.

CONSTITUTIONAL AMENDMENT 71, 2012
(Published in the Official Journal, November 30, 2012)
Adds Article 216-A to the Federal Constitution with a view to creating the National Culture System.

CONSTITUTIONAL AMENDMENT 72, 2013
(Published in the Official Journal, April 3, 2013)
Alters the wording of the single paragraph of Article 7 of the Federal Constitution to establish equality of labor rights among domestic workers and other urban and rural workers.

CONSTITUTIONAL AMENDMENT 73, 2013
(Published in the Official Journal, June 7, 2013)
Creates the Regional Federal Courts of Appeal of the 6th, 7th, 8th, and 9th Regions.

CONSTITUTIONAL AMENDMENT 74, 2013
(Published in the Official Journal, August 7, 2013)
Alters Article 134 of the Federal Constitution.

CONSTITUTIONAL AMENDMENT 75, 2013
(Published in the Official Journal, October 16, 2013)
Adds subitem e *to item VI of Article 150 of the Federal Constitution, establishing tax exemption for musical phonograms and videophonograms produced in Brazil containing musical works or literary musical works by Brazilian authors and/or works in general interpreted by Brazilian artists, as well as the physical media or digital files containing such works.*

CONSTITUTIONAL AMENDMENT 76, 2013

(Published in the Official Journal, November 29, 2013)

Alters paragraph 2 of Article 55 and paragraph 4 of Article 66 of the Federal Constitution, to end secret voting in the case of loss of office of a deputy or senator and of examination of a veto.

CONSTITUTIONAL AMENDMENT 77, 2014

(Published in the Official Journal, February 12, 2014)

Alters items II, III, and VIII of paragraph 3 of Article 142 of the Federal Constitution, to extend to healthcare professionals of the Armed Forces the possibility to accumulate offices referred to in Article 37, item XVI, subitem c.

CONSTITUTIONAL AMENDMENT 78, 2014

(Published in the Official Journal, May 15, 2014)

Adds Article 54-A to the Temporary Constitutional Provisions Act, to provide for the compensation due to rubber tappers referred to in Article 54 of this Act.

CONSTITUTIONAL AMENDMENT 79, 2014

(Published in the Official Journal, May 28, 2014)

Alters Constitutional Amendment 19, of June 4th, 1998.

CONSTITUTIONAL AMENDMENT 80, 2014

(Published in the Official Journal, June 5, 2014)

Alters Chapter IV – The Functions Essential to Justice, of Title IV – The Organization of the Branches, and adds an Article to the Temporary Constitutional Provisions Act of the Federal Constitution.

CONSTITUTIONAL AMENDMENT 81, 2014

(Published in the Official Journal, June 6, 2014)

Gives new wording to Article 243 of the Federal Constitution.

CONSTITUTIONAL AMENDMENT 82, 2014

(Published in the Official Journal, July 17, 2014)

Includes paragraph 10 in Article 144 of the Federal Constitution, to regulate road safety within the states, Federal District, and municipalities.

CONSTITUTIONAL AMENDMENT 83, 2014
(Published in the Official Journal, August 6, 2014)
Adds Article 92 A to the Temporary Constitutional Provisions Act.

CONSTITUTIONAL AMENDMENT 84, 2014
(Published in the Official Journal, December 3, 2014)
Alters Article 159 of the Federal Constitution to increase the amount of funds to be remitted by the Union to the Revenue Sharing Fund of the Municipalities.

CONSTITUTIONAL AMENDMENT 85, 2015
(Published in the Official Journal, February 27, 2015 and republished in the Official Journal, March 3, 2015)
Alters and adds provisions to the Federal Constitution to update the treatment given to science, technology, and innovation activities.

CONSTITUTIONAL AMENDMENT 86, 2015
(Published in the Official Journal, March 18, 2015)
Alters Articles 165, 166, and 198 of the Federal Constitution to make the execution of the budget appropriations it specifies mandatory.

CONSTITUTIONAL AMENDMENT 87, 2015
(Published in the Official Journal, April 17, 2015)
Alters paragraph 2 of Article 155 of the Federal Constitution and adds Article 99 to the Act of the Temporary Constitutional Provisions to provide about tax collection from transactions relating to the circulation of goods and rendering of interstate and intermunicipal transportation of services and of communication levied on the transactions and rendering of goods and services to end users located in another state, whether taxpayers or not.

CONSTITUTIONAL AMENDMENT 88, 2015
(Published in the Official Journal, May 8, 2015)
Alters Article 40 of the Federal Constitution, concerning the age limit for compulsory retirement of civil servants and adds a provision to the Act of the Temporary Constitutional Provisions.

CONSTITUTIONAL AMENDMENT 89, 2015

(Published in the Official Journal, September 16, 2015)

Gives new wording to Article 42 of the Temporary Constitutional Provisions Act, increasing the period in which the Federation shall allocate to the Central West and Northeast Regions the minimum percentages of the total amount of funds intended for irrigation.

CONSTITUTIONAL AMENDMENT 90, 2015

(Published in the Official Journal, September 16, 2015)

Gives new wording to Article 6 of the Federal Constitution to establish transportation as a social right.

CONSTITUTIONAL AMENDMENT 91, 2016

(Published in the Official Journal, February 19, 2016)

Alters the Federal Constitution to establish the possibility, exceptional and within limited period, of disaffiliating from a political party, without prejudice to the politician's office.

CONSTITUTIONAL AMENDMENT 92, 2016

(Published in the Official Journal, July 13, 2016)

Alters Articles 92 and 111-A of the Federal Constitution to specify the Superior Labor Court as a body of the Judiciary, to alter the requirements for filling the positions of justices of that court, and to change its jurisdiction.

CONSTITUTIONAL AMENDMENT 93, 2016

(Published in the Official Journal, September 9, 2016 – Extra Edition)

Alters the Temporary Constitutional Provisions Act to extend the detachment of revenues of the Union and establish the detachment of revenues of the states, Federal District and municipalities.

CONSTITUTIONAL AMENDMENT 94, 2016

(Published in the Official Journal, December 16, 2016)

Alters Article 100 of the Federal Constitution to provide for the regime to pay up public debts due to court orders; and includes provisions to the Temporary Constitutional Provisions Act, to establish a special regime of payment for the cases in default.

CONSTITUTIONAL AMENDMENT 95, 2016

(Published in the Official Journal, December 16, 2016)

Alters the Temporary Constitutional Provisions Act to establish the New Fiscal Regime and sets forth other provisions.

CONSTITUTIONAL AMENDMENT 96, 2016

(Published in the Official Journal, June 7, 2017)

Adds paragraph 7 to Article 225 of the Federal Constitution to establish that sporting activities that use animals are not considered cruel, under the conditions that it specifies.

CONSTITUTIONAL AMENDMENT 97, 2016

(Published in the Official Journal, October 5, 2017)

Alters the Federal Constitution to prohibit party coalitions in proportional elections, to establish rules on political party access to party fund resources and to free-of-charge propaganda time on radio and on television and to provide for transitional rules.

CONSTITUTIONAL AMENDMENT 98, 2016

(Published in the Official Journal, December 11, 2017)

Alters Constitutional Amendment 19, of June 4th, 1998.

CONSTITUTIONAL AMENDMENT 99, 2017

(Published in the Official Journal, December 15, 2017)

Alters Article 101 of the Temporary Constitutional Provisions Act, to establish a new special regime to pay up court-ordered debts, and Articles 102, 103 and 105 of the Temporary Constitutional Provisions Act.

CONSTITUTIONAL AMENDMENT 100, 2019

(Published in the Official Journal, June 27, 2019)

Alters Articles 165 and 166 of the Federal Constitution to establish that the execution of budget appropriations from amendments of state or district parliamentarian bench is mandatory.

CONSTITUTIONAL AMENDMENT 101, 2019
(Published in the Official Journal, July 4, 2019)
Adds paragraph 3 to Article 42 of the Federal Constitution to extend to militaries of the states, of the Federal District and of the Territories the right to accumulate public offices provided by Article 37, item XVI.

CONSTITUTIONAL AMENDMENT 102, 2019
(Published in the Official Journal, September 27, 2019)
Gives new wording to Article 20 of the Federal Constitution and alters Article 165 of the Federal Constitution and Article 107 of the Temporary Constitutional Provisions Act.

CONSTITUTIONAL AMENDMENT 103, 2019
(Published in the Official Journal, November 13, 2019)
Alters the social security system and establishes transitional rules and temporary provisions.

CONSTITUTIONAL AMENDMENT 104, 2019
(Published in the Official Journal, December 5, 2019)
Alters item XIV of the heading of article 21, paragraph 4 of Article 32 and Article 144 of the Federal Constitution, to create the federal, state and district correctional police.

CONSTITUTIONAL AMENDMENT 105, 2019
(Published in the Official Journal, December 13, 2019)
Adds article 166-A to the Federal Constitution to authorize the funds transfer to the states, the Federal District, and municipalities by means of amendments to the annual budget bill.

CONSTITUTIONAL AMENDMENT 106, 2020
(Published in the Official Journal, May 8, 2020)
Establishes an extraordinary fiscal, financial and of contracting regime to counter a national calamity resulting from a pandemic.

This book was designed by Eduardo Franco Dias and composed by Camila Penha Soares in the Office of Documentation of the Federal Supreme Court.

The book is set in Kepler Std, a typeface designed by Robert Slimbach and edited by Adobe Systems in 2003.
